# From Reagent to Catalyst: Dispersion-Driven Design of a General Asymmetric Transfer Hydrogenation Catalyst

**DOI:** 10.1021/jacs.5c00641

**Published:** 2025-05-06

**Authors:** Wencke Leinung, Benjamin Mitschke, Markus Leutzsch, Vijay N. Wakchaure, Rajat Maji, Benjamin List

**Affiliations:** Max-Planck-Institut für Kohlenforschung, Mülheim an der Ruhr, 45470 Germany

## Abstract

Even though chemists have long underappreciated the role of London dispersion in catalysis, its importance in determining a reaction course is now well recognized. Dispersion interactions have been shown to stabilize transition states and govern the stereoselectivity. In this context, the transfer hydrogenation of α,β-unsaturated aldehydes reported by our group via asymmetric counteranion-directed catalysis (ACDC) was revisited mechanistically. Previously, the use of an engineered Hantzsch ester featuring an isopropyl group was crucial for high enantioselectivity, suggesting London dispersion as an important stereocontrolling factor. Based on this hypothesis and the method’s drawbacks (commercially unavailable Hantzsch ester, limited substrate scope, high catalyst loadings), we designed a broadly applicable second-generation catalyst system by introducing dispersion energy donors into the catalyst instead of the Hantzsch ester reagent. With the help of computational analysis, noncovalent interactions contributing to stereocontrol in the two systems were elucidated.

## Graphical abstract

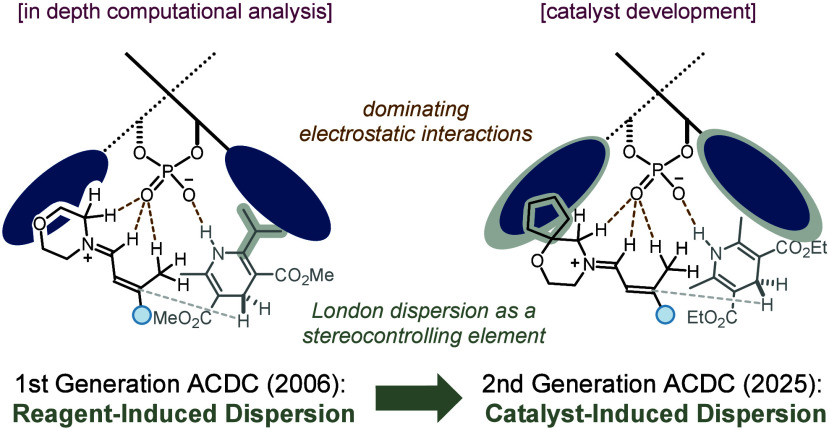

The origin of enantioselectivity in an asymmetric catalytic reaction lies in energetically different diastereomeric transition states evolving to opposite isoenergetic enantiomers.^1^ One transition state may be stabilized by attractive interactions between the chiral catalyst and substrate(s), lowering the kinetic barrier, or destabilized by steric repulsion, raising the transition state energy.^2^ In recent years, a reconsideration of dispersion interactions has transformed the way organic chemists think about sterically demanding residues from energetically repulsive moieties to stabilizing design elements.^3–16^ In this context, we now revisited our enantioselective transfer hydrogenation of β,β-disubstituted enals^17–23^ via asymmetric counteranion-directed catalysis (ACDC) (Figure 1A).^24^ Excellent enantioselectivities have been achieved, suspected as early as 2006 to arise not solely from ion pairing but also from a nonclassical CH···O hydrogen bond between the iminium cation and the chiral phosphate anion. Nevertheless, our conjugate reduction faced several drawbacks: (I) a substrate scope limited to aromatic and sterically nonhindered aliphatic enals, (II) high catalyst loadings, and (III) the utilization of an engineered, commercially unavailable Hantzsch ester (**2a**). When the latter was replaced with commercially available Hantzsch ester **2b** lacking the isopropyl group, a significant drop in selectivity was found (Figure 1A). This preliminary finding prompted a new hypothesis: additional dispersion interactions constitute an important stereocontrollability factor (Figure 1B). We aimed to design a superior catalyst system that would enable the efficient asymmetric conjugate reduction of α,β-unsaturated aldehydes in the presence of commercially available Hantzsch ester **2b**. By introducing dispersion energy donors (DEDs) into the catalyst, an alternative interaction handle may be provided. Here, we report the successful realization of this concept with a broadly applicable second generation catalyst system for the transformation of various β-methyl enals with Hantzsch ester **2b**.

**Figure 1:**
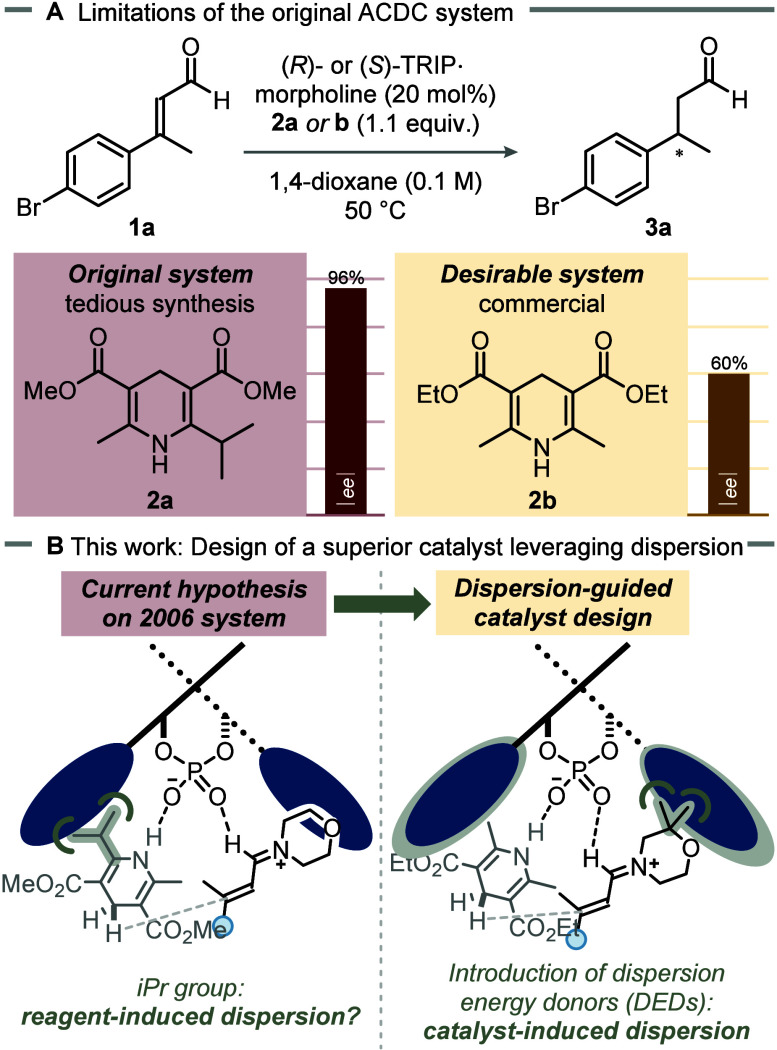
(A) Comparison of enantioselectivities in the transfer hydrogenation of enals with Hantzsch esters **2a** and **2b**. (B) This work: New catalyst design harnesses dispersion interactions.

We began by investigating stereodetermining factors and the role of London dispersion in the original 2006 system. Using density functional theory (DFT) and *ab initio* studies, we performed a transition state analysis (Figure 2). In the transition state leading to the major (*R*)-enantiomer, **TS-1**, the iminium ion coordinates to the phosphate via three CH···O hydrogen bonds, similar to our original proposal. We suggest this polarity-matched orientation to be a substrate recognition element of the catalyst and an explanation for the observed stereoconvergence through deprotonative dienamine formation (see SI for details). We identified two distinct transition states leading to the minor (*S*)-enantiomer, **TS-2** and **TS-2b** (see SI for **TS-2b**), uncovering several energetic trade-offs required to accommodate them. **TS-2** involves a complete enantiofacial flip of the iminium, reducing electrostatic interactions with the phosphate, somewhat mitigated by a shorter CH···O contact (2.30 vs 2.04 Å). In **TS-2b**, the iminium adopts a (*Z*)-configuration, partially restoring electrostatic interactions while the phenyl ring is oriented away from the 3,3′-substituents of the phosphate. Most importantly, our computational analysis is in good agreement with the experimentally observed enantioselectivity (ΔΔ*G* ^‡^ = 2.6 kcal/mol between **TS-1** and **TS-2**, matching the enantiomeric ratio of 98.5:1.5), and showed **TS-2b** to be inconsequential (ΔΔ*G* ^‡^ = 4.6 kcal/mol) at the CPCM­(1,4-dioxane)-DLPNO–CCSD­(T)/cc-pVTZ//PBE-D3­(BJ)/def2-SVP level of theory. Local energy decomposition (LED)^26–33^ analysis revealed a predominance of nondispersion interactions (ΔΔ*E*_no‑disp_ = 7.4 kcal/mol) in **TS-1**, while **TS-2** exhibited a greater contribution from dispersion interactions (ΔΔ*E*_disp_ = – 6.6 kcal/mol, see SI for further details). We attribute these findings to the polarity-matched orientation of the iminium ion in **TS-1**, promoting favorable electrostatic interactions, while enhanced dispersion in **TS-2** likely arises from proximity of the iminium phenyl fragment to the 3,3′-substituents of the phosphate anion, consistent with our structural analysis. Despite the significant dispersion contributions in **TS-2**, we focused on the investigation of stereocontrolling noncovalent interactions favoring **TS-1**, with particular emphasis on the isopropyl group of the Hantzsch ester. Using the Independent Gradient Model based on Hirshfeld partition (IGMH^25^) in conjunction with the Multiwfn program,^34,35^ we quantitatively analyzed noncovalent interfragment interactions between the phosphate anion and the combined Hantzsch ester-iminium ion system at the B3LYP-D3­(BJ)/def2-TZVPP//PBE-D3­(BJ)/def2-SVP level of theory. The isopropyl group contributed 21.8% to interfragment interactions in **TS-1** but only 11.5% in **TS-2** (Figure 2B, with color-coded atomic contributions to the interfragment interaction). In summary, we identified three distinct transition state models, two of which (**TS-1** and **TS-2**) are in alignment with the experimental data. Moreover, LED analysis highlighted the balance of electrostatic and dispersion interactions in **TS-1** vs **TS-2**. Importantly, IGMH analysis deciphered critical stereodifferentiating noncovalent interactions involving the isopropyl group of the Hantzsch ester, supporting our initial hypothesis.

**Figure 2:**
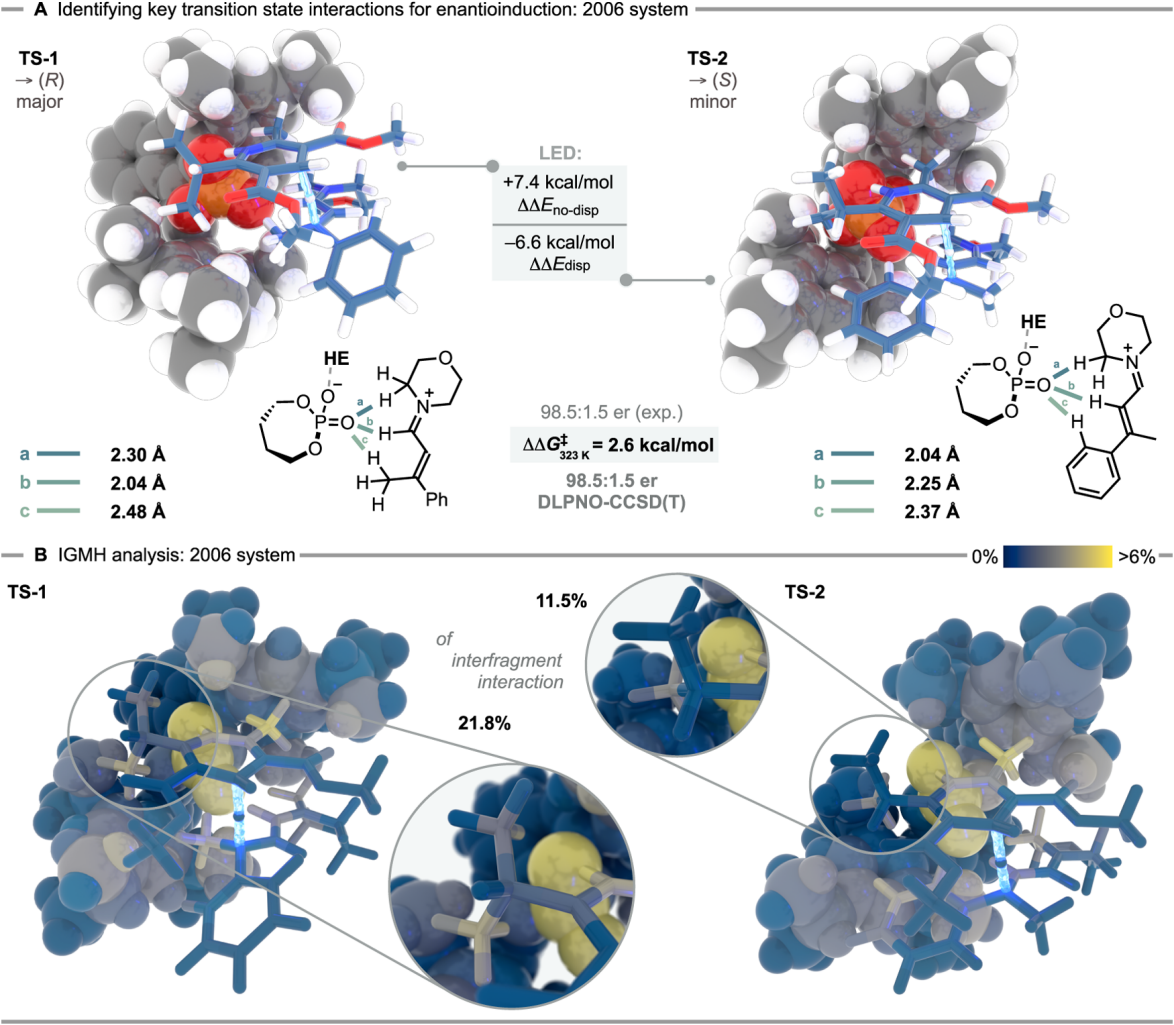
Computations for the 2006 system using (*S*)-TRIP·morpholine (**4a**): A) DFT transition state evaluation with Gibbs free enthalpies computed at the CPCM­(1,4-dioxane)-DLPNO–CCSD­(T)/cc-pVTZ//PBE-D3­(BJ)/def2-SVP level of theory at 323 K/1 M as well as key CH contacts. Energies are given in kcal mol^–1^. B) IGMH analysis^25^ using B3LYP-D3­(BJ)/def2-TZVPP densities, highlighting key noncovalent interactions.

Building on our understanding of the 2006 transfer hydrogenation system, we sought to harness specific dispersion interactions to develop a second-generation catalyst addressing its limitations. We initiated our studies by exploring the transfer hydrogenation of (*E*)-3-(4-bromophenyl)­but-2-enal (**1a**) as our model substrate using commercially available Hantzsch ester **2b** as a reductant under previously established conditions (Table 1). The morpholinium salt of (*S*)-TRIP (**4a**) resulted in full conversion but yielded an unsatisfying enantiomeric ratio (er) of 80:20 (entry 1), unaffected by reduced temperature (entry 2). Testing different chiral phosphoric acids (CPAs) revealed that introducing a second aryl substituent at the 3,3′-aryl wing of the BINOL backbone improved enantioselectivity (er = 9.5:90.5), without compromising reactivity (entry 3). Incorporating isopropyl groups at the 2,4,6-positions of the distal aromatic ring, which may facilitate beneficial dispersion interactions as demonstrated in previous studies,^36,37^ was found to enhance enantioselectivity (entry 4). Different morphologies featuring DEDs were also investigated. Introducing a *gem*-dimethyl group in the 3-position led not only to a severe decrease in reactivity, probably due to steric constraints, but also to reduced enantioselectivity (entry 5). Remarkably, however, we found geminal alkyl substituents in the 2-position to significantly improve the enantiomeric ratio (entries 6–8). Ultimately, with spiro-morpholinium salt **6e**, the catalyst loading was reduced to 5 mol % with excellent enantioselectivity (entry 9).

**Table 1:**
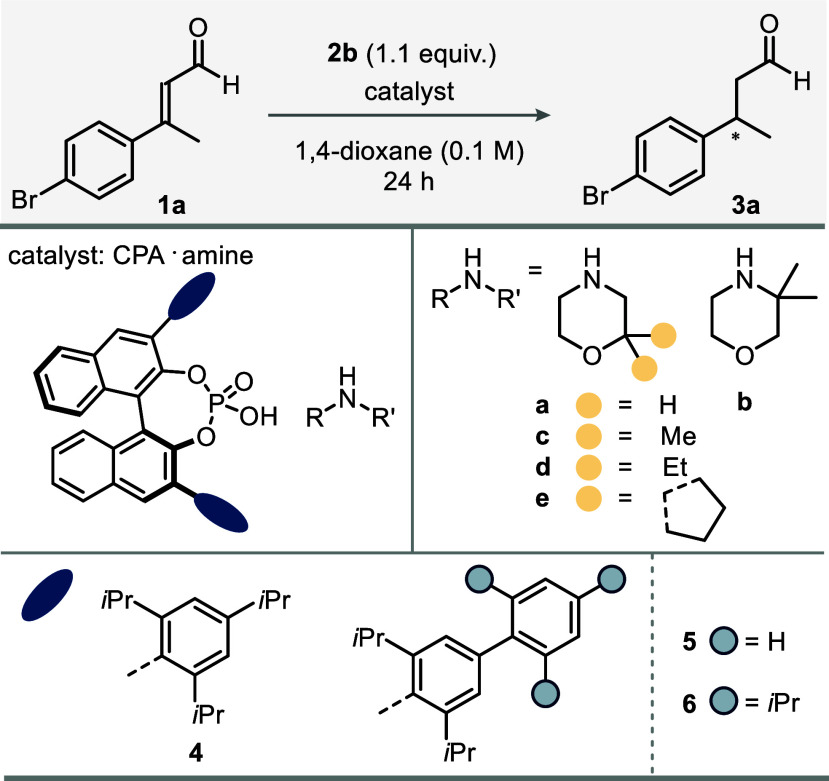
Reaction Development

entry^a^	catalyst	catalyst loading(mol %)	*T* (°C)	conv.^b^ (%)	er^c^
1	**4a**	20	50	100	80:20
2	**4a**	20	30	100	79.5:20.5
3	(*R*)-**5a**	20	50	100	9.5:90.5
4	**6a**	20	50	100	93:7
5	**6b**	20	50	45	62:38
6	**6c**	20	50	100	96:4
7	**6d**	20	50	100	97.5:2.5
8	**6e**	20	50	100	98:2
9	**6e**	5	50	100	98:2

a Reactions were performed on a 0.025–0.030 mmol scale with the indicated catalyst.

b Determined via ^1^H NMR analysis using Ph_3_CH as internal standard.

c Determined via GC.

We next explored the substrate scope of the reaction (Figure 3). β-Aryl-β-methyl-disubstituted enals with diverse substituents were, irrespective of their electronic and steric nature, well-tolerated. For example, β-branched aldehydes **3a**–**c** with electron-deficient groups (Br, CF_3_) were obtained in very good yields and with exceptional enantioselectivities. β-Aryl enals having electron-neutral or electron-donating groups at the *ortho*, *meta*- or *para*-positions were effectively reduced with excellent enantioselectivities (**3c**–**h**). Styrene-derivative **1i** also proved to be a suitable substrate for our methodology. Furthermore, disubstituted β-aryl enal **1j**, featuring a nitro and a methoxy group, furnished the desired aldehyde in very good yield and enantioselectivity. Remarkably, heteroaromatic aldehydes **3k** and **3l** were obtained in high yields with excellent enantioselectivities.

**Figure 3:**
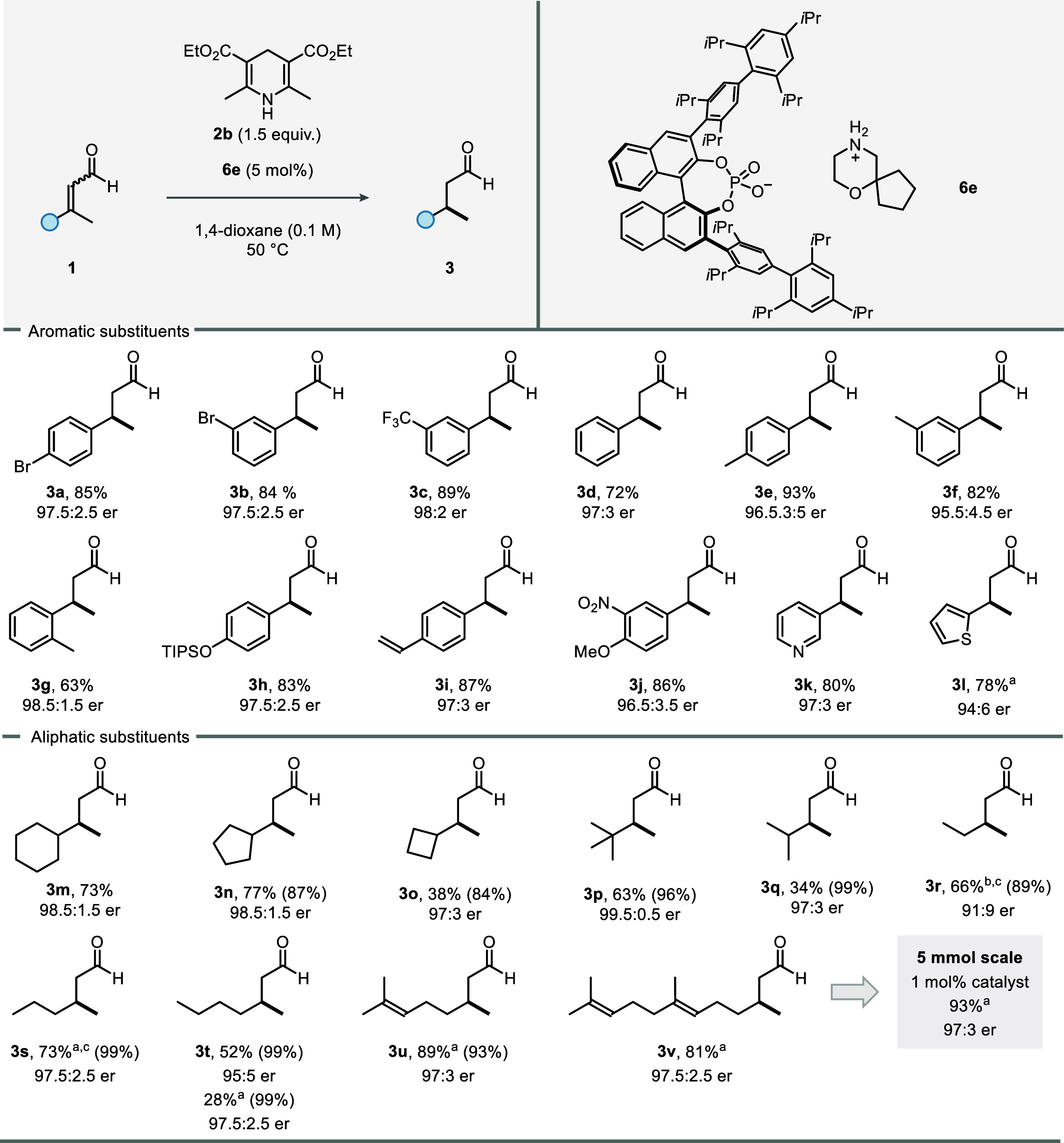
All reactions were conducted on a 0.2 mmol scale. Yields are reported as isolated yields after column chromatography. For some volatile substrates, ^1^H NMR yields, reported in parentheses, were determined using mesitylene as an internal standard. *E*/*Z* ratios for all starting materials were between 2:1 and >20:1. ^a^Reaction was performed in CyH at rt. ^b^Reaction was performed in CyH/*n*-pentane (9:1) at – 10 °C. ^c^Product was isolated after in situ derivatization with 2,4-dinitrophenylhydrazine as the corresponding hydrazone. See SI for detailed reaction conditions.

Finally, we explored aliphatic substrates, including sterically hindered ones, which had posed a significant challenge in our previous system. To our delight, enals bearing β-cycloalkyl substituents of differing ring sizes were effectively reduced to aldehydes **3m**–**o** in very good yields with excellent enantioselectivities. Sterically hindered substrates **1p–q** afforded almost quantitative yields and a very high enantioselectivity. Notably, 3-methylpent-2-enal (**1r**), an especially challenging substrate considering the notoriously difficult methyl–ethyl differentiation that is required to be controlled for high enantioselectivity, was converted to aldehyde **3r** with a satisfying er of 91:9. Prolonging the β-ethyl chain by one or two methylene groups (**3s**–**t**) restored the excellent enantioselectivity observed for all other substrates. Lastly, citral (*E*/*Z* = 3:1) and farnesal were subjected to our transfer hydrogenation protocol, furnishing (*S*)-citronellal (**3u**) and (*S*)- dihydrofarnesal (**3v**) in very high yields and enantioselectivities. Scaling the reaction with farnesal to 5 mmol allowed the catalyst loading to be reduced to 1 mol %, yielding (*S*)-dihydrofarnesal in improved yield and excellent enantiomeric ratio of 97:3.

Following the successful development of a second-generation transfer hydrogenation catalyst, we sought to elucidate the reasons behind its exceptional scope and stereocontrol. Visual inspection of the enantiodetermining optimized structures **TS-3** and **TS-4** shows that the Hantzsch ester methyl groups are deeply embedded within the phosphate pocket, therefore compensating for the lack of the isopropyl group (Figure 4). In general, **TS-3** shares similar characteristics as **TS-1** (see SI for more details and distortion-interaction analyses of all transition states), with a significant difference being the corresponding (*E*)- or (*Z*)-configuration of the C=N bond. We found the transition states with an *endo*-configuration of the spiro functionality to be energetically lower than their *exo*-counterparts (for a full description see SI); consequently, the following discussion focuses on the configuration of the C=C bond. In the 2006 system, the (*E*)-iminium configuration was identified as the lowest-lying transition state leading to the minor enantiomer (**TS-2**). In our new system, however, the (*Z*)-configured iminium ion transition state (**TS-4**) exhibited significant stabilization over the (*E*)-configuration (**TS-4b**), driven by dispersion interactions with the phosphate anion. This stabilization resulted in an energetic preference for **TS-4** over **TS-4b** at the CPCM­(1,4-dioxane)-DLPNO–CCSD­(T)/cc-pVTZ//PBE-D3­(BJ)/def2-SVP level of theory (ΔΔ*G* ^‡^ = 2.2 kcal/mol for **TS-4** vs 2.5 kcal/mol for **TS-4b**). Interestingly, LED analysis indicated that the (*Z*)-configuration allows both the α-CH_2_ of the morpholine as well as the formyl CH functionalities to retain some electrostatic interactions (ΔΔ*E*_no‑disp_ = 2.9 kcal/mol), contributing to the observed energetic difference. Once again, we employed the IGMH method to partition potential stereodetermining noncovalent interactions responsible for stereoinduction. In **TS-3**, a series of tighter CH···H contacts originating from the spiro-functionality of the morpholine fragment were observed. These contacts are significantly weakened in **TS-4**, leading to a stronger stabilization in **TS-3** (Figure 4B). Dispersion-driven close contact between the phenyl fragment and the 3,3′-substituents of the phosphate in **TS-4** (ΔΔ*E*_disp_ = – 6.9 kcal/mol), causes the morpholine fragment to protrude from the catalyst pocket. Consequently, only in **TS-3** does the spiro-moiety interact closely with the catalyst, accounting for the observed interfragment interaction differences (23.9% in **TS-3** vs 19.5% in **TS-4**). The importance of the catalyst design becomes evident upon inspection of the individual contributions of the extended triisopropylphenyl fragment (12.4% in **TS-3** vs 8.3% in **TS-4** for one interacting isopropyl group in the southern catalyst fragment, Figure 4B), whereas the dispersion stabilization of the iminium ion in **TS-4b** mainly originates from the 2,6-diisopropylphenyl fragment closest to the BINOL backbone. Furthermore, the methyl groups of the Hantzsch ester were also found to contribute to stereodifferentiation via noncovalent interactions, albeit to a lesser extent (for more details, see SI).

**Figure 4:**
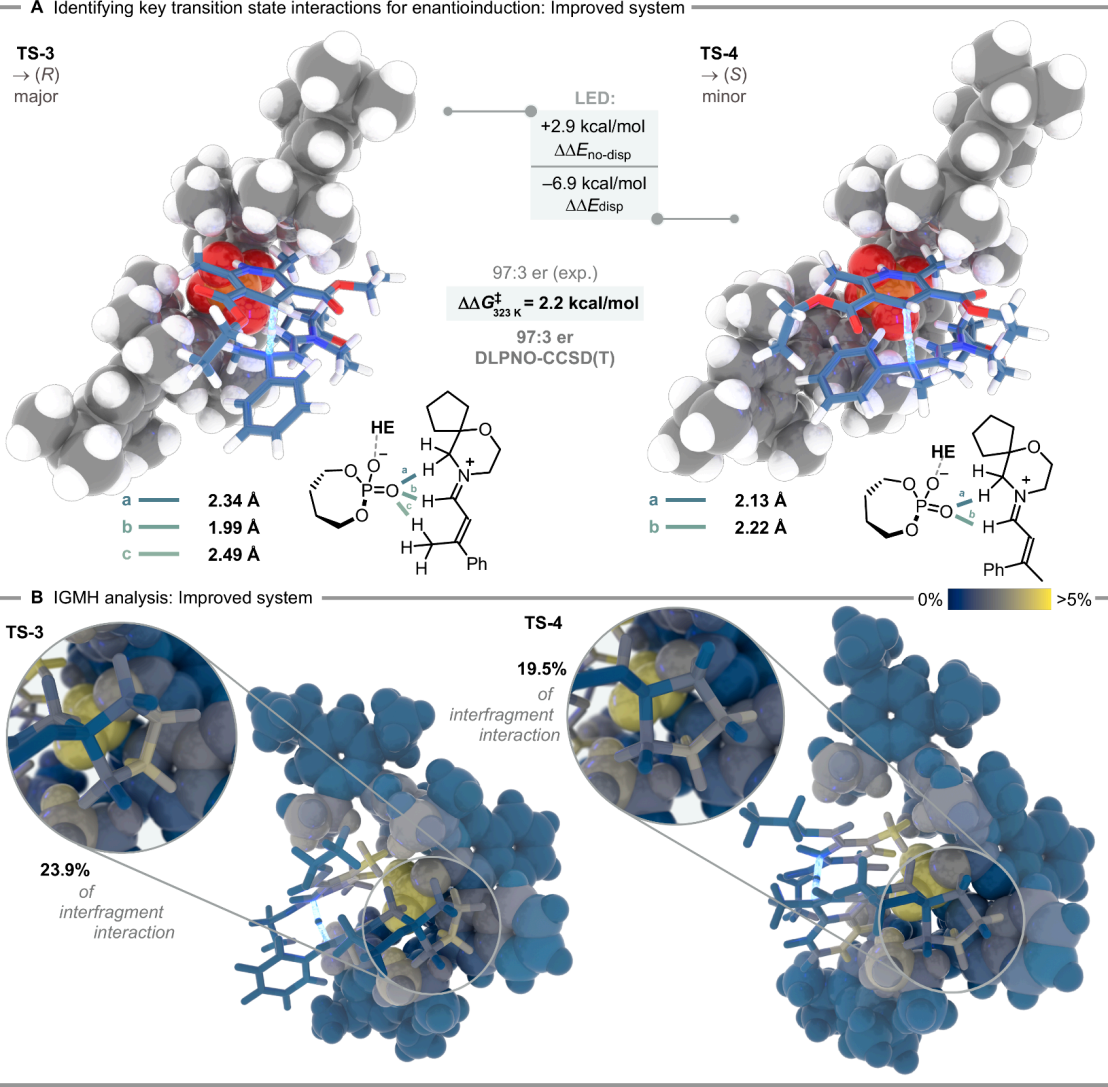
Computations for the new system using **6e**: A) DFT transition state evaluation with Gibbs free enthalpies computed at the CPCM­(1,4-dioxane)-DLPNO–CCSD­(T)/cc-pVTZ//PBE-D3­(BJ)/def2-SVP level of theory at 323 K/1 M as well as key CH contacts. Energies are given in kcal mol^–1^. B) IGMH analysis^25^ using B3LYP-D3­(BJ)/def2-TZVPP densities, highlighting key noncovalent interactions.

With the help of a thorough computational analysis of the first generation transfer hydrogenation system, we identified the polarity-match/-mismatch orientation of the iminium relative to the phosphate as a key stereodetermining element. Furthermore, dispersion interactions originating from the isopropyl group of the unsymmetrical Hantzsch ester **2a** were found to contribute to stereocontrol, substantiating our hypothesis. On this basis, we have developed a state-of-the-art, broadly applicable second-generation catalyst system for the transfer hydrogenation of β-methyl enals using the simpler and commercially available Hantzsch ester **2b**. While the enantioselectivity is, likewise, primarily driven by electrostatic interactions in the relevant transition states, crucial dispersion interactions arise from the spiro-moiety on the iminium’s morpholine fragment and the phosphate’s 3,3′ substituents, that were both conceptually introduced as DEDs into the catalyst. Our findings demonstrate that, even alongside electrostatic interactions, London dispersion may significantly contribute to the attractive forces stabilizing a transition state and should therefore be considered a critical element in catalyst design.

## Supplementary Material



## Notes

Additional experimental details, compound synthesis and characterization, computational data (PDF)

## §

Benjamin Mitschke – Laboratorium für Organische Chemie, ETH Zürich, 8093 Zürich, Switzerland

## ∥

Rajat Maji - Department of Chemistry and Biochemistry, University of California Los Angeles, Los Angeles, California 90095, United States

## ABBREVIATIONS

ACDC: asymmetric counteranion-directed catalysis
BINOL: 1,1′-bi-2-naphtol, CPA, chiral phosphoric acid
Cy: cyclohexyl
DFT: density functional theory
er: enantiomeric ratio
Et: ethyl
HE: Hantzsch ester
IM: iminium
*i*Pr: isopropyl
Me: methyl
NCI: noncovalent interactions
P: phosphate
SI: Supporting Information
TIPS: triisopropylsilyl
TRIP: 3,3′-bis­(2,4,6-triisopropylphenyl)-1,1′-binaphthyl-2,2′-diyl hydrogenphosphate
TS: transition state
