# Supplementary material for: From Reagent to Catalyst: Dispersion-Driven Design of a General Asymmetric Transfer Hydrogenation Catalyst
Source: J Am Chem Soc. 2025 May 6;147(20):16722–8. doi: 10.1021/jacs.5c00641 (PMC12100657; doi:10.1021/jacs.5c00641)
Supplement: Supplementary file 1 [file ja5c00641_si_001.pdf]

From Reagent to Catalyst: Dispersion-Driven Design of a General Asymmetric Transfer Hydrogenation Catalyst

Wencke Leinung[‡], Benjamin Mitschke[‡], Markus Leutzsch, Vijay N. Wakchaure, Rajat Maji, Benjamin List*

Table of Contents

1. **General Information** 2
2. **Reaction Optimization** 3
3. **Synthesis of α,β-Unsaturated Esters (Horner-Wadsworth-Emmons Reaction)** 6
4. **Synthesis of α,β-Unsaturated Aldehydes** 13
5. **Transfer Hydrogenation of α,β-Unsaturated Aldehydes** 23
6. **Catalyst Characterization (6e)** 33
7. **Mechanistic Experiments** 34
8. **Computational Details** 38
9. **Comparison of catalysts 4a, 4e, 6a and 6e** 137
10. **NMR Spectra** 140
11. **GC Traces** 186
12. **References** 210

1. General Information

Unless otherwise stated, all reagents were purchased from commercial suppliers and used without further purification. All solvents used in the reactions were distilled from appropriate drying agents prior to use. Reactions were monitored by thin layer chromatography (TLC) on silica gel pre-coated plastic sheets (0.2 mm, Macherey-Nagel). Visualization was performed by irradiation with UV light at 254 nm and/or phosphomolybdic acid (PMA) or $KMnO_4$ staining. Column chromatography was conducted using Merck silica gel (60 Å, 230–400 mesh, particle size 0.040–0.063 mm) using technical grade solvents. ^{1}H and ^{13}C NMR spectra were recorded on a Bruker Avance III 500 spectrometer in deuterated solvents. ^{1}H chemical shifts (δ) are reported in ppm relative to the protonated solvent resonance employed as the internal standard ($CDCl_3$ δ = 7.26, CD_2Cl_2 δ = 5.32). Data are reported as follows: chemical shift, multiplicity (s = singlet, d = doublet, t = triplet, q = quartet, p = pentet, s = sextet, h = heptet, m = multiplet, b = broad), coupling constants (Hz) and integration. When data from a mixture of *E*/*Z* isomers were reported, signals of the minor isomer were assigned only if they were unambiguously resolved. ^{13}C chemical shifts are reported in ppm with the solvent resonance as the internal standard ($CDCl_3$ δ = 77.16, CD_2Cl_2 δ = 54.00). High-resolution mass spectra were obtained using a Bruker APEX III FTMS (7 T magnet). Optical rotations were determined with an Autopol IV polarimeter (Rudolph Research Analytical) at 589 nm (sodium D line) and 25 °C. Data are reported as follows: $[\alpha]_D^T$, concentration c (g/100 mL) and solvent. Enantiomeric ratios (er) were determined by GC or HPLC analysis using a chiral stationary phase column, indicated in each experiment, by comparing the samples with the corresponding racemic mixtures.

2. Reaction Optimization

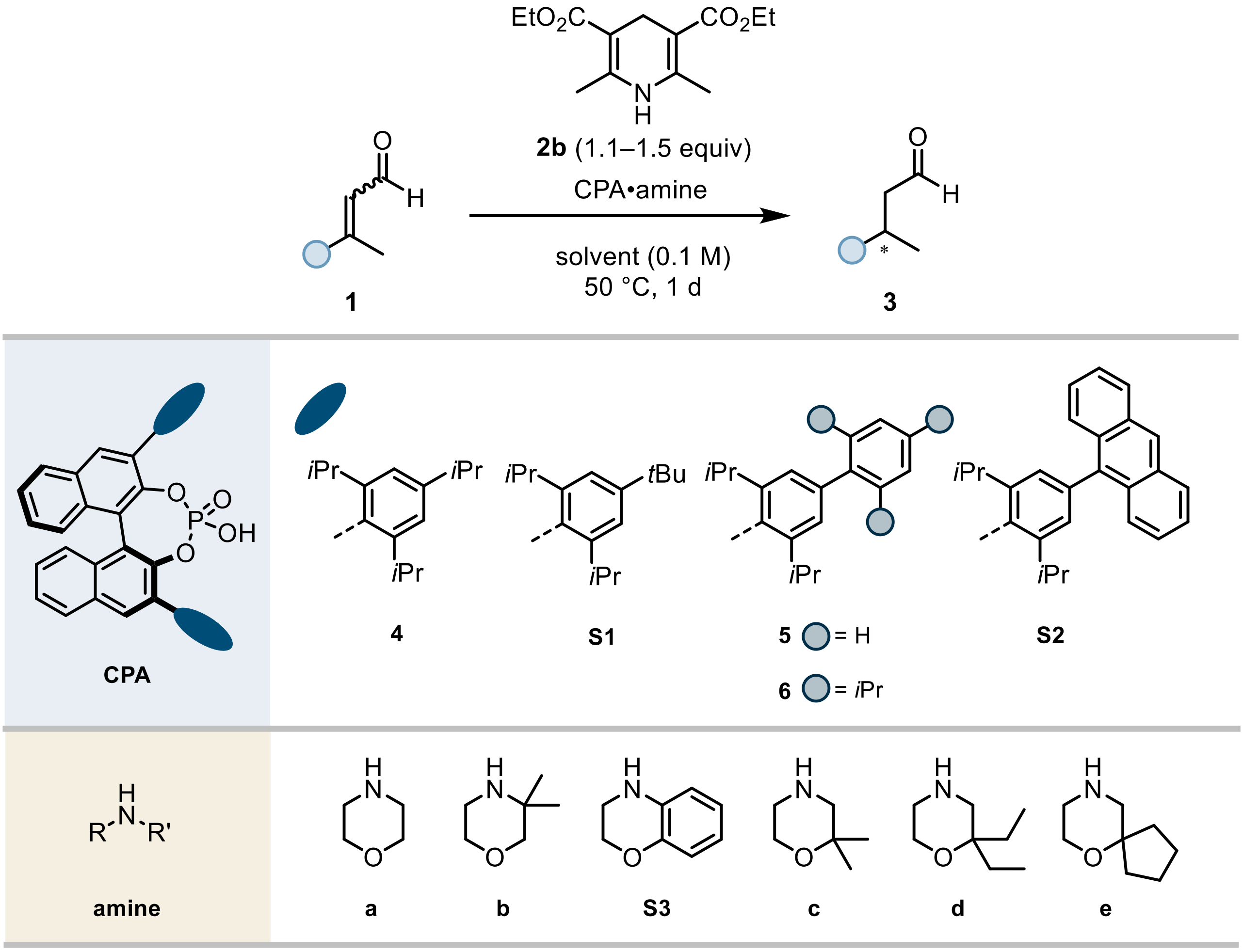

An oven-dried GC vial under argon, equipped with a magnetic stirring bar, was charged with the chiral phosphoric acid (CPA) and the amine of choice (2.5–20 mol%). Substrate **1** (0.025–0.03 mmol) was added and dissolved in the respective dry solvent. The reaction mixture was stirred for five minutes at room temperature. After addition of Hantzsch ester **2b** (1.1–1.5 equiv) the reaction mixture was heated to the desired temperature and stirred overnight (for reduced temperatures the reaction was set up at –78°C and warmed to the reaction temperature). The reaction was quenched by addition of NEt_3 (10 µL) followed by addition of Ph_3CH (1.0 M in PhMe) or mesitylene as internal standard. An aliquot of the mixture was taken and diluted with $CDCl_3$ for subsequent ^{1}H NMR analysis. The remaining solution was used for preparative thin layer chromatography to purify the chiral product **3**. Chiral GC analysis was performed to give the corresponding enantiomeric ratio.

***Table S1**: Reaction Optimization for* 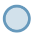 *= 4-Br-C_6H_4 (**1a/3a**).*

entry	CPA	amine	catalyst loading [mol%]	solvent	temperature	conversion	yield	er
1	4	a	20	1,4-dioxane	50 °C	100%	73%	80:20
2	4	a	20	1,4-dioxane	30 °C	92%	68%	79.5:20.5
3	4	a	10	1,4-dioxane	50 °C	100%	70%	80:20
4	4	a	5	1,4-dioxane	50 °C	100%	82%	80:20

entry	CPA	amine	catalyst loading [mol%]	solvent	temperature	conversion	yield	er
5	4	a	2.5	1,4-dioxane	50 °C	100%	78%	80:20
6	4	b	20	1,4-dioxane	50 °C	22%	7%	57:43
7	4	S3	20	1,4-dioxane	50 °C	100%	72%	53.5:46.5
8	(*R*)-S1	a	20	1,4-dioxane	50 °C	100%	69%	21:79
9	(*R*)-5	a	20	1,4-dioxane	50 °C	100%	74%	9.5:90.5
10	6	a	20	1,4-dioxane	50 °C	100%	58%	93:7
11	6	b	20	1,4-dioxane	50 °C	45%	28%	62:38
12	6	S3	20	1,4-dioxane	50 °C	100%	68%	83:17
13	6	c	20	1,4-dioxane	50 °C	100%	58%	96:4
14	6	d	20	1,4-dioxane	50 °C	96%	78%	97.5:2.5
15	6	e	20	1,4-dioxane	50 °C	94%	66%	98:2
16	6	e	5	1,4-dioxane	50 °C	92%	57%	98:2
17	6	e	2.5	1,4-dioxane	50 °C	100%	81%	98:2
18	6	e	5	$CHCl_3$	50 °C	60%	n.d.	88:12
19	6	e	5	THF	50 °C	92%	n.d.	93.5:6.5
20	6	e	5	Et_2O	rt	100%	n.d.	97.5:2.5
21	6	e	5	PhMe	50 °C	100%	n.d.	96.5:3.5
22	6	e	5	CyH	50 °C	100%	n.d.	97.5:2.5

***Table S2**: Reoptimization for other substrates.*

	CPA	amine	catalyst loading [mol%]	solvent	temperature	conversion	yield	er
	6	e	2.5	1,4-dioxane	50 °C	95%	51%	92:8
	(*R*)-S1	e	2.5	1,4-dioxane	50 °C	85%	40%	31.5:68.5
	(*R*)-5	e	2.5	1,4-dioxane	50 °C	87%	52%	27:73
	(*R*)-S2	e	2.5	1,4-dioxane	50 °C	86%	31%	16:84
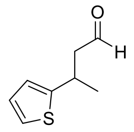	6	e	2.5	Et_2O	rt	88%	72%	92:8
	6	e	2.5	PhMe	rt	100%	87%	93:7
	6	e	2.5	*n*-pentane	rt	100%	70%	92:8
	6	e	2.5	CyH	rt	92%	72%	95:5
	6	e	2.5	CyMe	rt	64%	30%	93.5:6.5
	6	e	2.5	1,4-dioxane	50 °C	100%	57%	86.5:13.5
	(*R*)-S1	e	2.5	1,4-dioxane	50 °C	100%	67%	18:82
	(*R*)-5	e	2.5	1,4-dioxane	50 °C	97%	67%	17.5:82.5
	(*R*)-S2	e	2.5	1,4-dioxane	50 °C	97%	62%	17:83
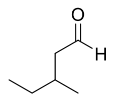	6	e	2.5	Et_2O	rt	100%	84%	86:14
	6	e	2.5	PhMe	rt	100%	87%	86.5:13.5
	6	e	2.5	*n*-pentane	rt	100%	83%	88:12

	CPA	amine	catalyst loading [mol%]	solvent	temperature	conversion	yield	er
	6	e	2.5	CyH	rt	100%	84%	90.5:9.5
	6	e	2.5	CyMe	rt	100%	85%	89.5:10.5
	6	e	2.5	CyH/*n*-pentane 9:1	0	75%[1]	59%	92:8
	6	e	2.5	CyH/*n*-pentane 9:1	–10	67%[1]	39%	92.5:7.5
	6	e	2.5	CyH/*n*-pentane 9:1	–20	35%[1]	20%	93.5:6.5
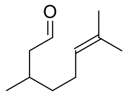	6	e	2.5	1,4-dioxane	50 °C	100%	83%	94:6
	(*R*)-S1	e	2.5	1,4-dioxane	50 °C	100%	40%	10:90
	(*R*)-5	e	2.5	1,4-dioxane	50 °C	98%	58%	9.5:90.5
	(*R*)-S2	e	2.5	1,4-dioxane	50 °C	80%	43%	11:89
	6	e	2.5	CyH	rt	100%	88%	97:3
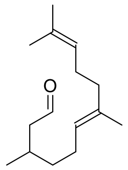	6	e	2.5	1,4-dioxane	50 °C	100%	56%	94.5:5.5
	6	e	2.5	CyH	rt	100%	61%	97.5:2.5

[1] Reaction was run for 2 days.

3. Synthesis of α,β-Unsaturated Esters (Horner-Wadsworth-Emmons Reaction)

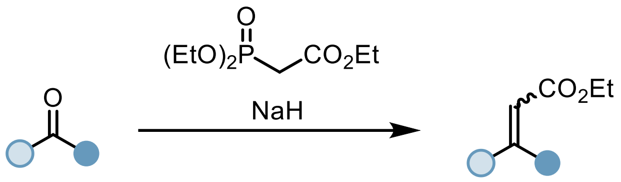

General procedure 1 (GP1)

In a flame-dried Schlenk flask under argon, NaH (60% in mineral oil, 1.5 equiv) was suspended in dry THF (0.2 M). After drowise addition of triethyl phosphonoacetate (1.7 equiv) at 0 °C, the reaction mixture was allowed to reach room temperature. The respective ketone (1.0 equiv) was then slowly added and the resulting mixture was stirred at room temperature overnight. The reaction was quenched by the addition of water and extracted with DCM (3x). The combined organic layers were washed with water (2x), dried over anhydrous Na_2CO_3 and concentrated under reduced pressure. The residue was purified *via* flash chromatography on silica gel to obtain the corresponding α,β-unsaturated ester.

ethyl 3-(4-bromophenyl)but-2-enoate (Sa)

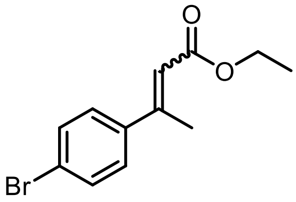

Following GP1 the reaction was performed using 4’-bromoacetophenone (2.00 g, 10.0 mmol, 1.00 equiv). Purification by silica gel flash chromatography (5% Et_2O in pentane) afforded the product as a mixture of *E*/*Z* isomers (2.63 g, 9.78 mmol, 97%, *E*/*Z*≈76:24) as a colorless oil. The NMR-spectroscopic data are in agreement with the literature.[1]

^{1}H NMR (501 MHz, $CDCl_3$): δ = 7.52–7.48 (m, $2H_{maj}$), 7.47 (d, J = 8.4 Hz, $2H_{min}$), 7.37–7.32 (m, $2H_{maj}$), 7.11–7.05 (m, $2H_{min}$), 6.11 (q, J = 1.3 Hz, $1H_{maj}$), 5.92 (q, J = 1.5 Hz, $1H_{min}$), 4.22 (q, J = 7.1 Hz, $2H_{maj}$), 4.01 (q, J = 7.2 Hz, $2H_{min}$), 2.54 (d, J = 1.3 Hz, $3H_{maj}$), 2.15 (d, J = 1.5 Hz, $3H_{min}$), 1.32 (t, J = 7.1 Hz, $3H_{maj}$), 1.12 (t, J = 7.1 Hz, $3H_{min}$).

ethyl 3-(3-bromophenyl)but-2-enoate (Sb)

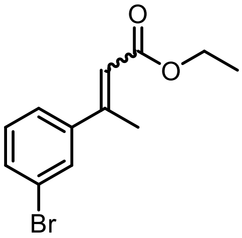

Following GP1 the reaction was performed using 3’-bromoacetophenone (2.0 mL, 15 mmol, 1.0 equiv). Purification by silica gel flash chromatography (5% Et_2O in pentane) afforded the product as a mixture of *E*/*Z* isomers (3.87 g, 14.4 mmol, 96%, *E*/*Z*≈89:11) as a colorless oil. The NMR-spectroscopic data are in agreement with the literature.[2]

^{1}H NMR (501 MHz, $CDCl_3$): δ = 7.61 (t, J = 1.9 Hz, $1H_{maj}$), 7.48 (ddd, J = 7.9, 2.0, 1.0 Hz, $1H_{maj}$), 7.43 (ddd, J = 8.0, 2.0, 1.1 Hz, $1H_{min}$), 7.39 (ddd, J = 7.8, 1.8, 1.0 Hz, $1H_{maj}$), 7.34 (t, J = 1.8 Hz, $1H_{min}$), 7.26 (d, J = 0.9 Hz, $1H_{maj}$), 7.24 (s, $1H_{min}$), 7.13 (dt, J = 7.7, 1.3 Hz, $1H_{min}$), 6.11 (q, J = 1.3 Hz, $1H_{maj}$), 5.92 (q, J = 1.5 Hz, $1H_{min}$), 4.22 (q, J = 7.1 Hz, $2H_{maj}$), 4.01 (q, J = 7.1 Hz, $2H_{min}$), 2.54 (d, J = 1.4 Hz, $3H_{maj}$), 2.15 (d, J = 1.4 Hz, $3H_{min}$), 1.32 (t, J = 7.1 Hz, $3H_{maj}$), 1.09 (t, J = 7.1 Hz, $3H_{min}$).

ethyl 3-(3-(trifluoromethyl)phenyl)but-2-enoate (Sc)

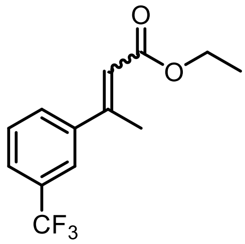

Following GP1 the reaction was performed using 3'-(trifluoromethyl)acetophenone (1.0 mL, 6.6 mmol, 1.0 equiv). Purification by silica gel flash chromatography (10% Et_2O in pentane) afforded the product as a mixture of *E*/*Z* isomers (1.41 g, 5.47 mmol, 83%, *E*/*Z*≈97:3) as a light yellow oil.

^{1}H NMR (501 MHz, $CDCl_3$): δ = 7.70 (d, *J* = 1.9 Hz, $1H_{maj}$), 7.67–7.59 (m, $2H_{maj}$), 7.50 (t, *J* = 7.8 Hz, $1H_{maj}$), 6.15 (q, *J* = 1.3 Hz, $1H_{maj}$), 4.23 (q, *J* = 7.1 Hz, $2H_{maj}$), 2.59 (d, *J* = 1.3 Hz, $3H_{maj}$), 1.33 (t, *J* = 7.1 Hz, $3H_{maj}$).

^{19}F{^{1}H} NMR (471 MHz, $CDCl_3$): δ = –62.6 (s, $3F_{min}$), –62.73 (s, $3F_{maj}$).

(*E*)- and (*Z*)-ethyl 3-phenylbut-2-enoate ((*E*)-Sd, (*Z*)-Sd)

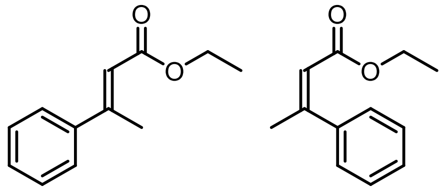

Following GP1 the reaction was performed using acetophenone (2.0 mL, 17 mmol, 1.0 equiv). Purification by silica gel flash chromatography (10% Et_2O in pentane) afforded the separated *E* (2.50 g, 13.2 mmol, 77%) and *Z* isomer (0.45 g, 2.4 mmol, 14%) of the product as light yellow oils. The NMR-spectroscopic data are in agreement with the literature.[1]

E isomer: **^{1}H NMR** (501 MHz, $CDCl_3$): δ = 7.51–7.43 (m, 2H), 7.41–7.33 (m, 3H), 6.13 (q, *J* = 1.4 Hz, 1H), 4.22 (q, *J* = 7.1 Hz, 2H), 2.58 (d, *J* = 1.3 Hz, 3H), 1.32 (t, *J* = 7.1 Hz, 3H).

Z isomer: **^{1}H NMR** (501 MHz, $CDCl_3$): δ = 7.38–7.28 (m, 3H), 7.23–7.17 (m, 2H), 5.91 (q, *J* = 1.5 Hz, 1H), 4.00 (q, *J* = 7.1 Hz, 2H), 2.18 (d, *J* = 1.5 Hz, 3H), 1.08 (t, *J* = 7.1 Hz, 3H).

ethyl 3-(*p*-tolyl)but-2-enoate (Se)

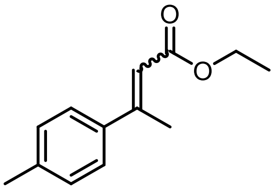

Following GP1 the reaction was performed using 4'-methylacetophenone (2.2 mL, 16 mmol, 1.0 equiv). Purification by silica gel flash chromatography (10% Et_2O in pentane) afforded the product as a mixture of *E*/*Z* isomers (3.15 g, 15.4 mmol, 94%, *E*/*Z*≈83:17) as a colorless oil. The NMR-spectroscopic data are in agreement with the literature.[1]

^{1}H NMR (501 MHz, $CDCl_3$): δ = 7.39 (d, *J* = 8.3 Hz, $2H_{maj}$), 7.20–7.10 (m, $2H_{maj}$, $4H_{min}$), 6.13 (q, *J* = 1.4 Hz, $1H_{maj}$), 5.89 (q, *J* = 1.5 Hz, $1H_{min}$), 4.21 (q, *J* = 7.1 Hz, $2H_{maj}$), 4.02 (q, *J* = 7.1 Hz, $1H_{min}$), 2.57 (d, *J* = 1.3 Hz, $3H_{maj}$), 2.16 (d, *J* = 1.5 Hz, $1H_{min}$), 1.32 (t, *J* = 7.1 Hz, $3H_{maj}$), 1.12 (t, *J* = 7.1 Hz, $3H_{min}$).

ethyl 3-(*m*-tolyl)but-2-enoate (Sf)

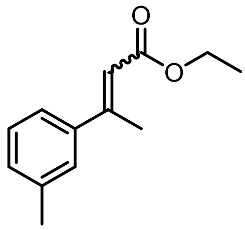

Following GP1 the reaction was performed using 3'-methylacetophenone (3.0 mL, 22 mmol, 1.0 equiv). Purification by silica gel flash chromatography (10% Et_2O in pentane) afforded the product as a mixture of *E*/*Z* isomers (4.42 g, 21.6 mmol, 98%, *E*/*Z*≈85:15) as a colorless oil. The NMR-spectroscopic data are in agreement with the literature.[3]

^{1}H NMR (501 MHz, $CDCl_3$): δ = 7.30–7.25 (m, $3H_{maj}$), 7.25–7.21 (m, $1H_{min}$), 7.20–7.15 (m, $1H_{maj}$), 7.13–7.10 (m, $1H_{min}$), 7.02–6.98 (m, $2H_{min}$), 6.12 (d, *J* = 1.4 Hz, $1H_{maj}$), 5.89 (d, *J* = 1.4 Hz, $1H_{min}$), 4.22 (q, *J* = 7.1 Hz, $2H_{maj}$), 4.00 (q, *J* = 7.1 Hz, $2H_{min}$), 2.57 (d, *J* = 1.4 Hz, $3H_{maj}$), 2.16 (d, *J* = 1.4 Hz, $3H_{min}$), 1.32 (t, *J* = 7.1 Hz, $3H_{maj}$), 1.08 (t, *J* = 7.1 Hz, $1H_{min}$).

ethyl (*E*)-3-(o-tolyl)but-2-enoate (Sg)

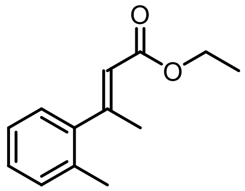

Following GP1 the reaction was performed using 2'-methylacetophenone (1.0 mL, 7.6 mmol, 1.0 equiv). Purification by silica gel flash chromatography (10–50% DCM in hexanes) afforded the product (612 mg, 3.00 mmol, 39%, *E*/*Z*>99:1) as a colorless oil. The NMR-spectroscopic data are in agreement with the literature.[4]

^{1}H NMR (501 MHz, $CDCl_3$): δ = 7.23–7.13 (m, 3H), 7.07 (dd, *J* = 7.4, 1.5 Hz, 1H), 5.76 (q, *J* = 1.4 Hz, 1H), 4.22 (q, *J* = 7.1 Hz, 2H), 2.45 (d, *J* = 1.4 Hz, 3H), 2.29 (s, 3H), 1.31 (t, *J* = 7.1 Hz, 3H).

ethyl 3-(4-((triisopropylsilyl)oxy)phenyl)but-2-enoate (Sh)

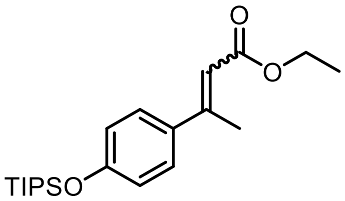

Following GP1 the reaction was performed using ethyl 3-(4-((triisopropylsilyl)oxy)phenyl)but-2-enoate[2] (4.59 g, 15.7 mmol, 1.00 equiv). The reaction mixture was additionally stirred at 80 °C overnight. Purification by silica gel flash chromatography (10% Et_2O in pentane) afforded the product (3.70 g, 10.2 mmol, 65%, *E*/*Z*≈98:2) as a colorless oil. The NMR-spectroscopic data are in agreement with the literature.[5]

^{1}H NMR (501 MHz, CD_2Cl_2): δ = 7.43–7.38 (m, $2H_{maj}$), 6.90–6.85 (m, $2H_{maj}$), 6.09 (q, *J* = 1.3 Hz, $1H_{maj}$), 4.17 (q, *J* = 7.1 Hz, $2H_{maj}$), 2.53 (d, *J* = 1.2 Hz, $3H_{maj}$), 1.31–1.24 (m, $6H_{maj}$), 1.11 (d, *J* = 7.4 Hz, $18H_{maj}$).

ethyl 3-(4-methoxy-3-nitrophenyl)but-2-enoate (Sj)

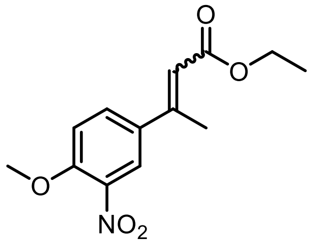

Following GP1 the reaction was performed using 4'-methoxy-3'-nitroacetophenone (2.00 g, 10.2 mmol, 1.00 equiv). Purification by silica gel flash chromatography (30% Et_2O in pentane) afforded the product as a mixture of *E*/*Z* isomers (1.99 g, 7.48 mmol, 73%, *E*/*Z*≈74:26) as an off-white solid.

^{1}H NMR (501 MHz, $CDCl_3$): δ = 7.99 (d, *J* = 2.5 Hz, $1H_{maj}$), 7.76 (d, *J* = 2.3 Hz, $1H_{min}$), 7.67 (dd, *J* = 8.8, 2.4 Hz, $1H_{maj}$), 7.44 (dd, *J* = 8.7, 2.2 Hz, $1H_{min}$), 7.09 (d, *J* = 8.8 Hz, $1H_{maj}$), 7.06 (d, *J* = 8.7 Hz, $1H_{min}$), 6.14 (q, *J* = 1.3 Hz, $1H_{maj}$), 5.95 (q, *J* = 1.5 Hz, $1H_{min}$), 4.22 (q, *J* = 7.1 Hz, $2H_{maj}$), 4.04 (q, *J* = 7.1 Hz, $2H_{min}$), 3.99 (s, $3H_{maj}$), 3.97 (s, $3H_{min}$), 2.56 (d, *J* = 1.3 Hz, $3H_{maj}$), 2.18 (d, *J* = 1.5 Hz, $3H_{min}$), 1.32 (t, *J* = 7.1 Hz, $3H_{maj}$), 1.15 (t, *J* = 7.1 Hz, $3H_{min}$).

^{13}C NMR (126 MHz, $CDCl_3$): δ = 166.5, 165.5, 153.4, 152.8, 152.4, 152.1, 139.6, 139.1, 134.4, 133.6, 132.7, 131.9, 124.9, 123.7, 119.0, 117.7, 113.7, 113.0, 60.2, 60.2, 56.8, 56.7, 26.9, 17.6, 14.4, 14.2.

HRMS *m*/*z* (GC-EI): calcd. for $C_{13}H_{15}N_1O_5$ ($[M]^+$): 265.094474; found: 265.094600.

ethyl 3-(pyridin-3-yl)but-2-enoate (Sk)

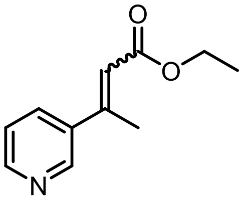

Following GP1 the reaction was performed using 3-acetylpyridine (2.0 mL, 18 mmol, 1.0 equiv). Purification by silica gel flash chromatography (5–30% EtOAc in hexanes) afforded the product as a mixture of *E*/*Z* isomers (1.68 g, 8.77 mmol, 48%, *E*/*Z*≈97:3) as a yellow oil. The NMR-spectroscopic data are in agreement with the literature.[1]

^{1}H NMR (501 MHz, $CDCl_3$): δ = 8.72 (d, *J* = 2.4 Hz, $1H_{maj}$), 8.59 (dd, *J* = 4.8, 1.6 Hz, $1H_{maj}$), 7.75 (dt, *J* = 7.9, 2.2 Hz, $1H_{maj}$), 7.30 (dd, *J* = 8.0, 4.8 Hz, $1H_{maj}$), 6.14 (q, *J* = 1.4 Hz, $1H_{maj}$), 4.22 (qd, *J* = 7.1, 1.1 Hz, $2H_{maj}$), 2.58 (d, *J* = 1.4 Hz, $3H_{maj}$), 1.32 (td, *J* = 7.2, 1.1 Hz, $3H_{maj}$).

ethyl 3-(thiophen-2-yl)but-2-enoate (Sl)

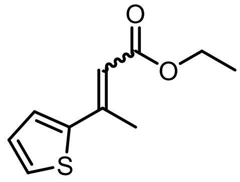

Following GP1 the reaction was performed using 2-acetylthiophene (2.0 mL, 19 mmol, 1.0 equiv). Purification by silica gel flash chromatography (2–5% EtOAc in hexanes) afforded the product as a mixture of *E*/*Z* isomers (3.64 g, 16.3 mmol, 88%, *E*/*Z*≈87:13) as a light yellow oil. The NMR-spectroscopic data are in agreement with the literature.[1]

^{1}H NMR (501 MHz, $CDCl_3$): δ = 7.49–7.44 (m, $1H_{min}$), 7.38 (d, *J* = 4.8 Hz, $1H_{min}$), 7.34–7.29 (m, $2H_{maj}$), 7.07–7.01 (m, $1H_{maj}$, $1H_{min}$), 6.27–6.24 (m, $1H_{maj}$), 5.89–5.82 (m, $1H_{min}$), 4.20 (q, *J* = 2.4 Hz, $2H_{maj}$), 4.16–4.07 (m, $2H_{min}$), 2.61 (s, $3H_{maj}$), 2.29 (s, $3H_{min}$), 1.31 (t, *J* = 7.2 Hz, $3H_{maj}$), 1.25 (t, *J* = 7.2 Hz, $3H_{min}$).

ethyl 3-cyclohexylbut-2-enoate (Sm)

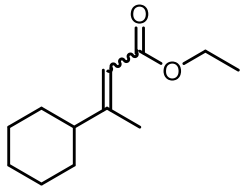

Following GP1 the reaction was performed using cyclohexyl methyl ketone (1.8 mL, 13 mmol, 1.0 equiv). Purification by silica gel flash chromatography (20–30% DCM in hexanes) afforded the product as a mixture of *E*/*Z* isomers (1.64 g, 8.37 mmol, 64%, *E*/*Z*≈89:11) as a colorless oil. The NMR-spectroscopic data are in agreement with the literature.[6]

^{1}H NMR (501 MHz, $CDCl_3$): δ = 5.64 (d, *J* = 1.5 Hz, $1H_{maj}$), 5.57 (d, *J* = 1.5 Hz, $1H_{min}$), 4.13 (q, *J* = 7.1 Hz, $2H_{maj}$, $2H_{min}$), 3.60 (tt, *J* = 11.5, 3.5 Hz, $1H_{min}$), 2.13 (d, *J* = 1.1 Hz, $3H_{maj}$), 1.96 (tt, *J* = 11.3, 3.2 Hz, $1H_{maj}$), 1.82–1.51 (m, $8H_{min}$,$5H_{maj}$), 1.41–1.10 (m, $8H_{maj}$, $8H_{min}$).

ethyl 3-cyclopentylbut-2-enoate (Sn)

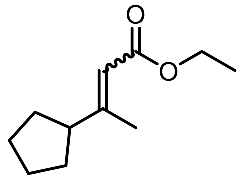

Following GP1 the reaction was performed using cyclopentyl methyl ketone (2.0 mL, 16 mmol, 1.0 equiv). Purification by silica gel flash chromatography (5% Et_2O in pentane) afforded the product as a mixture of *E*/*Z* isomers (2.75 g, 15.1 mmol, 93%, *E*/*Z*≈86:14) as a colorless oil. The NMR-spectroscopic data are in agreement with the literature.[7]

^{1}H NMR (501 MHz, $CDCl_3$): δ = 5.70 (s, $1H_{maj}$), 5.64 (s, $1H_{min}$), 4.14 (q, *J* = 7.2 Hz, $2H_{maj}$, $2H_{min}$), 4.07–3.98 (m, $1H_{min}$), 2.59–2.46 (m, $1H_{maj}$), 2.15 (d, *J* = 1.2 Hz, $3H_{maj}$), 1.87–1.77 (m, $2H_{maj}$, $5H_{min}$), 1.74–1.55 (m, $4H_{maj}$, $4H_{min}$), 1.49–1.39 (m, $2H_{maj}$, $2H_{min}$), 1.31–1.23 (m, $3H_{maj}$, $3H_{min}$).

ethyl 3-cyclobutylbut-2-enoate (So)

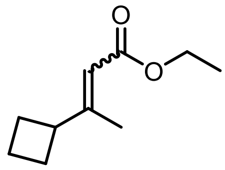

Following GP1 the reaction was performed using cyclobutyl methyl ketone (2.5 mL, 23 mmol, 1.0 equiv). Purification by silica gel flash chromatography (4% Et_2O in pentane) afforded the product (3.33 g, 19.8 mmol, 86%) as a colorless oil. The exact *E*/*Z* ratio could not be determined by NMR analysis due to significant signal overlap.

^{1}H NMR (501 MHz, $CDCl_3$): δ = 5.59 (t, *J* = 1.5 Hz, 1H), 4.18–4.09 (m, 2H), 3.05–2.94 (m, 1H), 2.17–2.09 (m, 2H), 2.08–2.05 (m, 3H), 1.99–1.85 (m, 3H), 1.75–1.67 (m, 1H), 1.28 (t, *J* = 7.2 Hz, 3H).

^{13}C NMR (126 MHz, $CDCl_3$): δ = 167.4, 162.8, 113.2, 59.6, 44.3, 27.3, 17.6, 16.4, 14.5. (Signals of major (*E*)-isomer given.)

HRMS *m*/*z* (GC-EI): calcd. for $C_{10}H_{16}O_2$ ($[M]^+$): 168.114480; found: 168.114550.

ethyl (*E*)-3,4,4-trimethylpent-2-enoate (Sp)

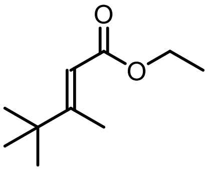

Following GP1 the reaction was performed using 3,3-dimethylbutan-2-one (3.7 mL, 30 mmol, 1.0 equiv). Purification by silica gel flash chromatography (20% DCM in pentane) afforded the product (2.42 g, 14.2 mmol, 48%) as a colorless oil. The NMR-spectroscopic data are in agreement with the literature.[8]

^{1}H NMR (501 MHz, $CDCl_3$): δ = 5.73 (d, *J* = 1.5 Hz, 1H), 4.14 (q, *J* = 7.1 Hz, 2H), 2.16 (d, *J* = 1.1 Hz, 3H), 1.28 (t, *J* = 7.1 Hz, 3H), 1.10 (s, 9H).

ethyl 3,4-dimethylpent-2-enoate (Sq)

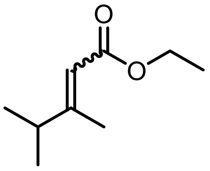

Following GP1 the reaction was performed using 3-methylbutan-2-one (5.0 mL, 47 mmol, 1.0 equiv). Purification by silica gel flash chromatography (1–5% Et_2O in pentane) afforded the product as a mixture of *E*/*Z* isomers (6.81 g, 43.6 mmol, 93%, *E*/*Z*≈85:15) as a colorless oil. The NMR-spectroscopic data are in agreement with the literature.[9]

^{1}H NMR (501 MHz, $CDCl_3$): δ = 5.67 (s, $1H_{maj}$), 5.58 (d, *J* = 1.6 Hz, $1H_{min}$), 4.14 (q, *J* = 7.2 Hz, $2H_{maj}$, $2H_{min}$), 3.97 (hept, *J* = 7.1 Hz, $1H_{min}$), 2.35 (hept, *J* = 6.7 Hz, $1H_{maj}$), 2.13 (d, *J* = 1.3 Hz, $3H_{maj}$), 1.79 (d, *J* = 1.4 Hz, $3H_{min}$), 1.27 (t, *J* = 7.2 Hz, $3H_{maj}$, $3H_{min}$), 1.06 (d, *J* = 6.8 Hz, $6H_{maj}$), 1.03 (d, *J* = 6.8 Hz, $6H_{min}$).

ethyl 3-methylpent-2-enoate (Sr)

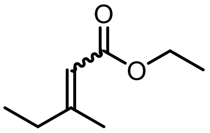

Following GP1 the reaction was performed using butan-2-one (3.7 mL, 41 mmol, 1.0 equiv). Purification by silica gel flash chromatography (1–5% Et_2O in pentane) afforded the product as a mixture of *E*/*Z* isomers (5.28 g, 37.1 mmol, 90%, *E*/*Z*≈75:25) as a colorless oil. The NMR-spectroscopic data are in agreement with the literature.[10]

^{1}H NMR (501 MHz, $CDCl_3$): δ = 5.66–5.63 (m, $1H_{maj}$), 5.62–5.60 (m, $1H_{min}$), 4.18–4.07 (m, $2H_{maj}$, $2H_{min}$), 2.62 (q, *J* = 7.6 Hz, $2H_{min}$), 2.18–2.11 (m, $5H_{maj}$), 1.86 (s, $3H_{min}$), 1.29–1.23 (m, $3H_{maj}$, $3H_{min}$), 1.03–1.08 (m, $3H_{maj}$, $3H_{min}$).

ethyl 3-methylhex-2-enoate (Ss)

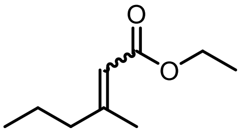

Following GP1 the reaction was performed using pentan-2-one (2.0 mL, 19 mmol, 1.0 equiv). Purification by silica gel flash chromatography (2% Et_2O in pentane) afforded the product as a mixture of *E*/*Z* isomers (2.58 g, 16.5 mmol, 88%, *E*/*Z*≈73:27) as a colorless oil. The NMR-spectroscopic data are in agreement with the literature.[11]

^{1}H NMR (501 MHz, $CDCl_3$): δ = 5.65 (q, *J* = 1.3 Hz, $1H_{maj}$, $1H_{min}$), 4.17–4.11 (m, $2H_{maj}$, $2H_{min}$), 2.63–2.57 (m, $2H_{min}$), 2.14 (d, *J* = 1.2 Hz, $3H_{maj}$), 2.11 (td, *J* = 7.4, 1.2 Hz, $2H_{maj}$), 1.87 (s, $3H_{min}$), 1.55–1.46 (m, $2H_{maj}$, $2H_{min}$), 1.29–1.25 (m, $3H_{maj}$, $3H_{min}$), 0.95 (t, *J* = 7.4 Hz, $3H_{min}$), 0.91 (t, *J* = 7.4 Hz, $3H_{maj}$).

ethyl 3-methylhept-2-enoate (St)

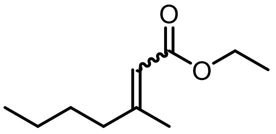

Following GP1 the reaction was performed using hexan-2-one (2.0 mL, 16 mmol, 1.0 equiv). Purification by silica gel flash chromatography (2% Et_2O in pentane) afforded the product as a mixture of *E*/*Z* isomers (2.46 g, 14.5 mmol, 89%, *E*/*Z*≈78:22) as a colorless oil. The NMR-spectroscopic data are in agreement with the literature.[12]

^{1}H NMR (501 MHz, $CDCl_3$): δ = 5.66 (q, *J* = 1.3 Hz, $1H_{maj}$),), 5.64 (d, *J* = 1.5 Hz, $1H_{min}$), 4.17–4.11 (m, $2H_{maj}$, $2H_{min}$), 2.65–2.59 (m, $2H_{min}$), 2.15 (d, *J* = 1.4 Hz, $3H_{maj}$), 2.14–2.11 (m, $2H_{maj}$), 1.88 (s, $3H_{min}$), 1.47–1.42 (m, $2H_{maj}$, $2H_{min}$), 1.38–1.22 (m, $5H_{maj}$, $5H_{min}$), 0.94–0.89 (m, $3H_{maj}$, $3H_{min}$).

(*E*)- and (*Z*)-ethyl 4,4,4-trifluoro-3-phenylbut-2-enoate ((*E*)-Sw, (*Z*)-Sw)

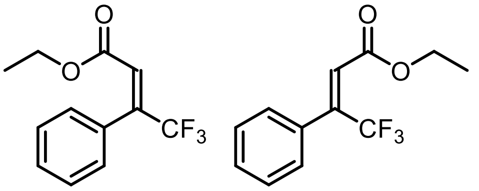

Following GP1 the reaction was performed using trifluoroacetyl benzene (560 µL, 4.0 mmol, 1.0 equiv). Purification by silica gel flash chromatography (5% Et_2O in pentane) afforded the separated *E* (761 mg, 3.12 mmol, 78%) and *Z* isomer (70.0 mg, 0.287 mmol, 7%) of the product as light yellow oils. The NMR-spectroscopic data are in agreement with the literature.[1]

E isomer: **^{1}H NMR** (501 MHz, $CDCl_3$): δ = 7.44–7.38 (m, 3H), 7.30–7.27 (m, 2H), 6.61 (q, *J* = 1.4 Hz, 1H), 4.04 (q, *J* = 7.1 Hz, 2H), 1.06 (t, *J* = 7.1 Hz, 3H).

Z isomer: **^{1}H NMR** (501 MHz, $CDCl_3$): δ = 7.41–7.41 (m, 5H), 6.34 (s, 1H), 4.31 (q, *J* = 7.1 Hz, 2H), 1.35 (t, *J* = 7.1 Hz, 3H).

ethyl 3-phenylpent-2-enoate (Sx)

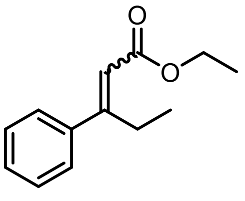

Following GP1 the reaction was performed using propiophenone (3.0 mL, 37 mmol, 1.0 equiv). Purification by silica gel flash chromatography (4% EtOAc in hexanes) afforded the product as a mixture of *E*/*Z* isomers (1.38 g, 4.37 mmol, 64%, *E*/*Z*≈78:22) as a colorless oil. The NMR-spectroscopic data are in agreement with the literature.[13]

^{1}H NMR (501 MHz, $CDCl_3$): δ = 7.45–7.29 (m, $5H_{maj}$, $5H_{min}$), 7.17–7.12 (m, $2H_{min}$), 6.02 (s, $1H_{maj}$), 5.87 (s, $1H_{min}$), 4.22 (q, *J* = 7.1 Hz, $2H_{maj}$), 3.98 (q, *J* = 7.2 Hz, $2H_{min}$), 3.11 (q, *J* = 7.4 Hz, $2H_{maj}$), 2.46 (qd, *J* = 7.4, 1.5 Hz, $2H_{min}$), 1.32 (t, *J* = 7.1 Hz, $3H_{maj}$), 1.10–1.04 (m, $6H_{maj}$, $3H_{min}$).

(*E*)- and (*Z*)-ethyl 3-phenylhept-2-enoate ((*E*)-Sy, (*Z*)-Sy)

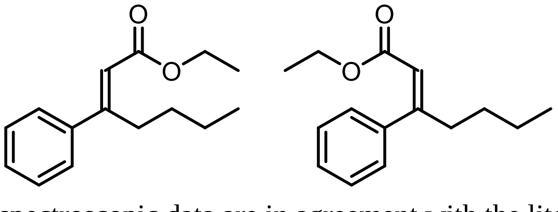

Following GP1 the reaction was performed using 1-phenylpentan-1-one (5.1 mL, 31 mmol, 1.0 equiv). Purification by silica gel flash chromatography (1–5% Et_2O in pentane) afforded the separated *E* (3.8 g, 16 mmol, 53%) and *Z* isomer (3.3 g, 14 mmol, 46%) of the product as colorless oils. The NMR-spectroscopic data are in agreement with the literature.[1]

E isomer: **^{1}H NMR** (501 MHz, $CDCl_3$): δ = 7.45–7.40 (m, 2H), 7.40–7.33 (m, 3H), 6.01 (s, 1H), 4.21 (q, *J* = 7.1 Hz, 2H), 3.13–3.05 (m, 2H), 1.44–1.34 (m, 4H), 1.31 (t, *J* = 7.1 Hz, 3H), 0.88 (t, *J* = 7.0 Hz, 3H).

Z isomer: **^{1}H NMR** (501 MHz, $CDCl_3$): δ 7.36–7.27 (m, 3H), 7.17–7.13 (m, 2H), 5.87 (s, 1H), 3.98 (q, *J* = 7.1 Hz, 2H), 2.44 (ddd, *J* = 8.2, 6.9, 1.3 Hz, 2H), 1.41–1.28 (m, 4H), 1.06 (t, *J* = 7.1 Hz, 3H), 0.87 (t, *J* = 7.1 Hz, 3H).

4. Synthesis of α,β-Unsaturated Aldehydes

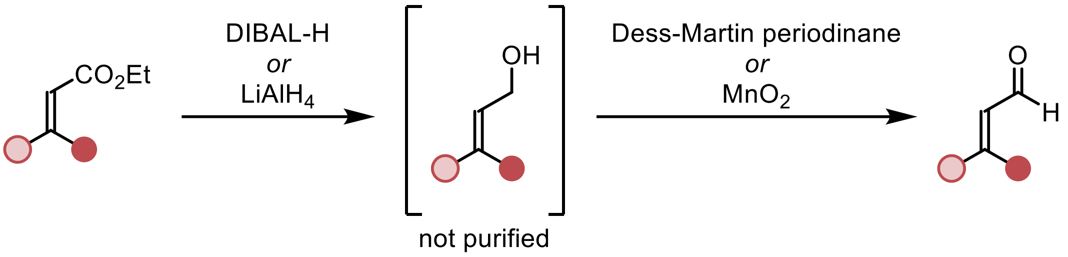

General procedure 2 (GP2)

a:In a flame-dried Schlenk flask under argon, the respective unsaturated ester (1.0 equiv) dissolved in dry DCM (0.2 M) was cooled to –78 °C. Then a solution of DIBAL-H (1 M in DCM, 2.5–3.0 equiv) was added dropwise. The reaction mixture was stirred at that temperature overnight until the reaction was judged to be complete by TLC. After warming the mixture to 0 °C, water was added dropwise to quench the reaction, followed by aqueous NaOH (10%). After separation of the organic layer, the aqueous phase was extracted with DCM (3x). The combined organic layers were washed with water, dried over anhydrous Na_2SO_4 and concentrated under reduced pressure. The residue was taken on to the next step without further purification.

b:In a flame-dried Schlenk flask under argon, the respective unsaturated ester (1.0 equiv) dissolved in dry THF (0.4 M) was cooled to 0 °C. Then a solution of $LiAlH_4$ (1 M in THF, 1.0 equiv) was added dropwise. The reaction mixture was stirred at that temperature for 1–3 h until the reaction was judged to be complete by TLC. After warming the mixture to 0 °C, water was added dropwise to quench the reaction, followed by aqueous NaOH (10%). After separation of the organic layer, the aqueous phase was extracted with DCM (3x). The combined organic layers were washed with water, dried over anhydrous Na_2SO_4 and concentrated under reduced pressure. The residue was taken on to the next step without further purification.

General procedure 3 (GP3)

a:A flame-dried Schlenk flask under argon, was charged with the crude allylic alcohol (1.0 equiv) dissolved in dry DCM (0.2 M). After cooling down to 0 °C, Dess-Martin periodinane (1.1 equiv) was added in one portion. The reaction mixture was stirred at room temperature for 3–6 h, until TLC analysis indicated complete conversion of the starting material. Subsequent addition of aqueous $NaHCO_3$ was followed by extraction of the aqueous phase with DCM (3x). The combined organic layers were washed with water, dried over anhydrous Na_2SO_4 and concentrated under reduced pressure. The residue was purified *via* flash chromatography on silica gel to obtain an *E*/*Z*-mixture of the corresponding α,β-unsaturated aldehyde.

b:A flame-dried Schlenk flask under argon was charged with the crude allylic alcohol (1.0 equiv) dissolved in dry DCM (0.4 M). MnO_2 (10 equiv) was added and the resulting reaction mixture was stirred at room temperature for 1–3 days until TLC analysis indicated complete conversion of the starting material. The suspension was filtered over a short pad of Celite and washed with DCM. Subsequently, the filtrate was concentrated under reduced pressure. The residue was purified *via* flash chromatography on silica gel to obtain an *E*/*Z*-mixture of the corresponding α,β-unsaturated aldehyde.

3-(4-bromophenyl)but-2-enal (1a)

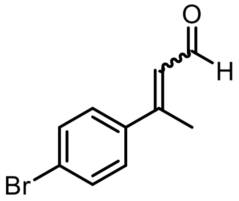

Preparation according to GP2a and GP3a followed by silica gel flash chromatography (10% Et_2O in pentane) afforded the product as a mixture of *E*/*Z* isomers as a yellow oil (1234 mg, 5.48 mmol, 72%, *E*/*Z*≈77:23).

^{1}H NMR (501 MHz, CD_2Cl_2): δ = 10.15 (d, *J* = 7.7 Hz, $1H_{maj}$), 9.44 (d, *J* = 8.2 Hz, $1H_{min}$), 7.58–7.50 (m, $2H_{maj}$, $2H_{min}$), 7.47–7.39 (m, $2H_{maj}$), 7.20 (d, *J* = 8.4 Hz, $2H_{min}$), 6.32 (dq, *J* = 7.8, 1.4 Hz, $1H_{maj}$), 6.10 (dd, *J* = 8.1, 1.4 Hz, $1H_{min}$), 2.52 (d, *J* = 1.4 Hz, $3H_{maj}$), 2.27 (d, *J* = 1.4 Hz, $3H_{min}$).

^{13}C NMR (126 MHz, CD_2Cl_2): δ = 192.7, 191.2, 160.7, 156.2, 139.9, 137.8, 132.1, 131.9, 130.4, 129.8, 128.2, 127.7, 124.5, 123.5, 26.4, 16.4.

HRMS *m*/*z* (GC-EI): calcd. for $C_{10}H_9O_1Br_1$ ($[M]^+$): 223.983140; found: 223.983140.

3-(3-bromophenyl)but-2-enal (1b)

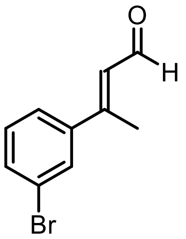

Preparation according to GP2a and GP3a followed by silica gel flash chromatography (20% Et_2O in pentane) afforded the product as a white solid (571 mg, 2.54 mmol, 77%, *E*/*Z*>99:1).

^{1}H NMR (501 MHz, CD_2Cl_2): δ = 10.16 (d, *J* = 7.7 Hz, 1H), 7.70 (t, *J* = 1.9 Hz, 1H), 7.56 (ddd, *J* = 8.0, 2.0, 1.0 Hz, 1H), 7.50 (ddd, *J* = 7.9, 1.9, 1.0 Hz, 1H), 7.31 (t, *J* = 7.9 Hz, 1H), 6.31 (dq, *J* = 7.7, 1.3 Hz, 1H), 2.53 (d, *J* = 1.3 Hz, 3H).

^{13}C NMR (126 MHz, CD_2Cl_2): δ = 191.2, 156.0, 143.4, 133.1, 130.7, 129.7, 128.3, 125.4, 123.2, 16.6.

HRMS *m*/*z* (GC-EI): calcd. for $C_{10}H_9O_1Br_1$ ($[M]^+$): 223.983140; found: 223.983160.

(*E*)-3-(3-(trifluoromethyl)phenyl)but-2-enal (1c)

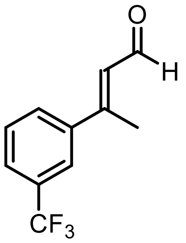

Preparation according to GP2a and GP3a followed by silica gel flash chromatography (15% Et_2O in pentane) afforded the product as a mixture of *E*/*Z* isomers as a light yellow oil (358 mg, 1.67 mmol, 89%, *E*/*Z*≈99:1).

^{1}H NMR (501 MHz, CD_2Cl_2): δ = 10.18 (d, *J* = 7.6 Hz, 1H), 7.80 (d, *J* = 1.9 Hz, 1H), 7.76 (dt, *J* = 7.9, 1.5 Hz, 1H), 7.71–7.66 (m, 1H), 7.58 (t, *J* = 7.8 Hz, 1H), 6.36 (dt, *J* = 7.6, 1.3 Hz, 1H), 2.58 (d, *J* = 1.3 Hz, 3H).

^{13}C NMR (126 MHz, CD_2Cl_2): δ = 191.2, 156.0, 142.1, 131.3 (q, *J* = 32.4 Hz), 130.1, 129.8, 128.7, 126.8 (q, *J* = 3.7 Hz), 124.5 (q, *J* = 272.4 Hz), 123.5 (q, *J* = 3.9 Hz), 16.7.

^{19}F{^{1}H} NMR (471 MHz, CD_2Cl_2): δ = –63.1.

HRMS *m*/*z* (GC-CI): calcd. for $C_{11}H_{10}O_1F_3$ ($[M+H]^+$): 215.067826; found: 215.067890.

(*E*)-3-phenylbut-2-enal ((*E*)-1d)

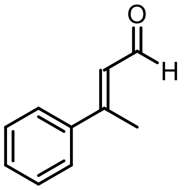

Preparation according to GP2a and GP3a followed by silica gel flash chromatography (5–10% Et_2O in pentane) afforded the product as a light yellow oil (728 mg, 4.98 mmol, 88%, *E*/*Z*>99:1).

^{1}H NMR (501 MHz, CD_2Cl_2): δ = 10.17 (d, *J* = 7.8 Hz, 1H), 7.61–7.54 (m, 2H), 7.47–7.39 (m, 2H), 6.36 (dd, *J* = 7.8, 1.3 Hz, 1H), 2.56 (d, *J* = 1.3 Hz, 3H).

^{13}C NMR (126 MHz, CD_2Cl_2): δ = 191.4, 157.9, 141.1, 130.3, 129.1, 127.6, 126.7, 16.6.

HRMS *m*/*z* (GC-EI): calcd. for $C_{10}H_{10}O_1$ ($[M]^+$): 146.072615; found: 146.072690.

(*Z*)-3-phenylbut-2-enal ((*Z*)-1d)

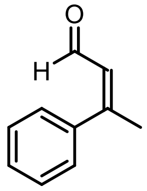

Preparation according to GP2a and GP3a followed by silica gel flash chromatography (5–10% Et_2O in pentane) afforded the product as a light yellow oil (339 mg, 2.32 mmol, 65%, *E*/*Z*<1:99).

^{1}H NMR (501 MHz, CD_2Cl_2): δ = 9.45 (d, *J* = 8.1 Hz, 1H), 7.46–7.38 (m, 3H), 7.36–7.29 (m, 2H), 6.10 (dq, *J* = 8.2, 1.4 Hz, 1H), 2.31 (d, *J* = 1.4 Hz, 3H).

^{13}C NMR (126 MHz, CD_2Cl_2): δ = 193.4, 162.5, 139.0, 129.5, 129.4, 128.8, 128.7, 26.6.

HRMS *m*/*z* (GC-EI): calcd. for $C_{10}H_{10}O_1$ ($[M]^+$): 146.072615; found: 146.072740.

(*E*)-3-(p-tolyl)but-2-enal (1e)

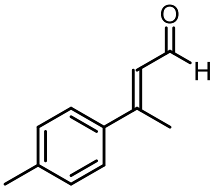

Preparation according to GP2a and GP3a followed by silica gel flash chromatography (10% Et_2O in pentane) afforded the product as a light yellow oil (364 mg, 2.27 mmol, 59%, *E*/*Z*>99:1).

^{1}H NMR (501 MHz, CD_2Cl_2): δ = 10.16 (d, *J* = 7.9 Hz, 1H), 7.52–7.45 (m, 2H), 7.26–7.20 (m, 3H), 6.35 (dt, *J* = 7.9, 1.3 Hz, 1H), 2.54 (d, *J* = 1.3 Hz, 3H), 2.38 (s, 3H).

^{13}C NMR (126 MHz, CD_2Cl_2): δ = 191.4, 157.7, 141.0, 138.0, 129.8, 126.8, 126.6, 21.4, 16.4.

HRMS *m*/*z* (GC-EI): calcd. for $C_{11}H_{13}O_1$ ($[M+H]^+$): 161.096090; found: 161.096250.

(*E*)-3-(m-tolyl)but-2-enal (1f)

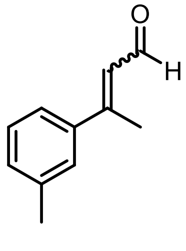

Preparation according to GP2a and GP3b followed by silica gel flash chromatography (20% Et_2O in pentane) afforded the product as a mixture of *E*/*Z* isomers as a light yellow oil (736 mg, 4.59 mmol, 69%, *E*/*Z*≈94:6).

^{1}H NMR (501 MHz, CD_2Cl_2): δ = 10.16 (d, *J* = 7.9 Hz, $1H_{maj}$), 9.45 (d, *J* = 8.1 Hz, $1H_{min}$), 7.40–7.34 (m, $2H_{maj}$), 7.30 (t, *J* = 7.6 Hz, $1H_{maj}$, $1H_{min}$), 7.26–7.21 (m, $1H_{maj}$, $1H_{min}$), 7.15–7.10 (m, $2H_{min}$), 6.34 (dq, *J* = 7.9, 1.3 Hz, $1H_{maj}$), 6.08 (dq, *J* = 8.1, 1.4 Hz, $1H_{min}$), 2.55 (d, *J* = 1.3 Hz, $3H_{maj}$), 2.39 (d, *J* = 0.8 Hz, $3H_{maj}$, $3H_{min}$), 2.29 (d, *J* = 1.4 Hz, $3H_{min}$).

^{13}C NMR (126 MHz, CD_2Cl_2): δ = 193.5, 191.5, 158.1, 141.1, 138.9, 131.1, 130.1, 129.4, 129.3, 128.9, 128.6, 127.5, 127.3, 125.9, 123.8, 26.7, 21.6, 21.5, 16.6.

HRMS *m*/*z* (EI): calcd. for $C_{11}H_{12}O_1$ ($[M]^+$): 160.088265; found: 160.088310.

(*E*)-3-(o-tolyl)but-2-enal (1g)

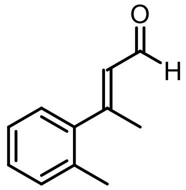

Preparation according to GP2b and GP3a followed by silica gel flash chromatography (5% Et_2O in pentane) afforded the product as a light yellow oil (377 mg, 2.35 mmol, 62%, *E*/*Z*>99:1).

^{1}H NMR (501 MHz, CD_2Cl_2): δ = 10.15 (d, *J* = 7.9 Hz, 1H), 7.29–7.15 (m, 3H), 7.11 (dd, *J* = 7.3, 1.2 Hz, 1H), 5.90 (dq, *J* = 7.9, 1.4 Hz, 1H), 2.45 (d, *J* = 1.4 Hz, 3H), 2.31 (s, 3H).

^{13}C NMR (126 MHz, CD_2Cl_2): δ = 191.3, 161.2, 143.3, 134.2, 131.0, 130.5, 128.5, 127.1, 126.2, 19.9, 19.5.

HRMS *m/z* (GC-EI): calcd. for $C_{11}H_{12}O_1$ ($[M]^+$): 160.088265; found: 160.088540.

(*E*)-3-(4-((triisopropylsilyl)oxy)phenyl)but-2-enal (1h)

Preparation according to GP2b and GP3a followed by silica gel flash chromatography (10% Et_2O in pentane) afforded the product as a yellow solid (354 mg, 1.11 mmol, 65%, *E*/*Z*≈99:1).

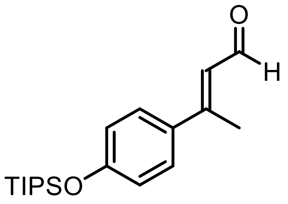

^{1}H NMR (501 MHz, CD_2Cl_2): δ = 10.14 (d, *J* = 7.9 Hz, 1H), 7.54–7.47 (m, 2H), 6.95–6.88 (m, 2H), 6.34 (dq, *J* = 7.9, 1.2 Hz, 1H), 2.53 (d, *J* = 1.2 Hz, 3H), 1.35–1.21 (m, 3H), 1.11 (d, *J* = 7.4 Hz, 18H).

^{13}C NMR (126 MHz, CD_2Cl_2): δ = 191.4, 158.6, 157.2, 133.2, 128.2, 125.9, 120.4, 18.1, 16.2, 13.1.

HRMS *m/z* (ESI): calcd. for $C_{19}H_{30}O_2Si_1Na_1$ ($[M+Na]^+$): 341.19073; found: 341.19064.

3-(4-vinylphenyl)but-2-enal (1i)

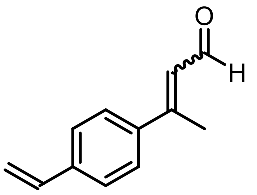

A flame-dried Schlenk flask under argon was charged with 3-(4-bromophenyl)but-2-enal (504 mg, 2.24 mmol, 1.00 equiv), trimethyl orthoformate (0.29 mL, 2.69 mmol, 1.20 equiv), anhydrous camphorsulfonic acid (5.2 mg, 22 µmol, 1.0 mol%) and anhydrous methanol (2 mL). The reaction mixture was stirred overnight until TLC analysis indicated full conversion of the starting material. Addition of aqueous $NaHCO_3$ (20 mL) was followed by extraction with Et_2O (3x 20 mL). The combined organic phases were dried over anhydrous Na_2SO_4 and concentrated under reduced pressure. The residue was transferred into a flame-dried flask under argon and dissolved in dry toluene (7.5 mL). After addition of tributylvinyltin (1.07 g, 3.36 mmol, 1.50 equiv) and tetrakis(triphenylphosphine)palladium(0) (52 mg, 45 µmol, 2.0 mol%) the reaction mixture was heated to 120 °C and stirred for 22 h. Subsequent cooling down to ambient temperature was followed by addition of a saturated KF solution (5 mL). The formed precipitate was filtered off over a short pad of Celite. The aqueous phase was extracted with DCM (4 x 30 mL). The combined organic phases were washed with water (2 x 20 mL), dried over anhydrous Na_2SO_4 and concentrated under reduced pressure. The crude oil was then dissolved in 4 mL THF and 2 mL HCl (10%) and stirred for 1 h. Dilution with water (30 mL) was followed by extraction with DCM (3 x 30 mL). The combined organic phases were dried over anhydrous Na_2SO_4 and concentrated under reduced pressure. After purification via column chromatography (60% DCM, hexanes) an *E*/*Z* mixture of the product (262 mg, 2.24 mmol, 68%, *E*/*Z*≈84:16) was obtained as a yellow creamy solid.

^{1}H NMR (501 MHz, CD_2Cl_2): δ = 10.17 (d, *J* = 7.8 Hz, $1H_{maj}$), 9.48 (d, *J* = 8.2 Hz, $1H_{min}$), 7.59–7.52 (m, $2H_{maj}$), 7.51–7.44 (m, $2H_{maj}$, $2H_{min}$), 7.32–7.27 (m, $2H_{min}$), 6.80–6.71 (m, $1H_{maj}$, $1H_{min}$), 6.38 (dq, *J* = 7.9, 1.3 Hz, $1H_{maj}$), 6.09 (dq, *J* = 8.2, 1.4 Hz, $1H_{min}$), 5.88–5.79 (m, $1H_{maj}$, $1H_{min}$), 5.37–5.31 (m, $1H_{maj}$, $1H_{min}$), 2.55 (d, *J* = 1.2 Hz, $3H_{maj}$), 2.30 (d, *J* = 1.4 Hz, $3H_{min}$).

^{13}C NMR (126 MHz, CD_2Cl_2): δ = 193.3, 191.4, 161.9, 157.1, 140.2, 139.7, 138.8, 138.4, 136.4, 136.4, 129.5, 129.2, 127.2, 126.9, 126.8, 126.5, 115.5, 115.3, 26.4, 16.4.

HRMS *m/z* (EI): calcd. for $C_{12}H_{12}O_1$ ($[M]^+$): 172.088265; found: 172.088440.

3-(4-methoxy-3-nitrophenyl)but-2-enal (1j)

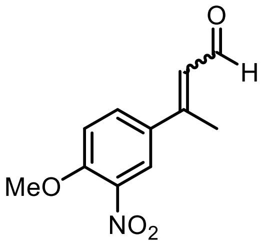

Preparation according to GP2a and GP3b followed by silica gel flash chromatography (3% Et_2O in DCM) afforded the product as a mixture of *E*/*Z* isomers as a yellow solid (145 mg, 0.66 mmol, 33%, *E*/*Z*≈82:18).

^{1}H NMR (501 MHz, CD_2Cl_2): δ = 10.16 (d, *J* = 7.6 Hz, $1H_{maj}$), 9.48 (d, *J* = 8.1 Hz, $1H_{maj}$), 8.06 (d, *J* = 2.5 Hz, $1H_{maj}$), 7.83–7.76 (m, $1H_{maj}$, $1H_{min}$), 7.52 (dd, *J* = 8.6, 2.3 Hz, $1H_{min}$), 7.17 (d, *J* = 9.0 Hz, $1H_{maj}$, $1H_{min}$), 6.37 (d, *J* = 1.3 Hz, $1H_{maj}$), 6.14 (dq, *J* = 8.1, 1.5 Hz, $1H_{min}$), 4.00 (d, *J* = 1.4 Hz, $3H_{maj}$, $3H_{min}$), 2.55 (d, *J* = 1.3 Hz, $3H_{maj}$), 2.30 (d, *J* = 1.5 Hz, $3H_{min}$).

^{13}C NMR (126 MHz, CD_2Cl_2): δ = 192.4, 191.1, 158.9, 154.3, 153.7, 140.0, 134.7, 133.1, 132.2, 131.0, 130.3, 127.5, 125.6, 123.9, 114.3, 114.1, 57.2, 57.2, 26.3, 16.3.

HRMS *m*/*z* (GC-EI): calcd. for $C_{11}H_{11}O_4N_1$ ($[M]^+$): 221.068259; found: 221.068310.

(*E*)-3-(pyridin-3-yl)but-2-enal (1k)

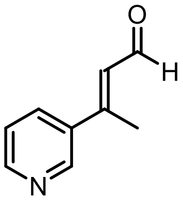

Preparation according to GP2a and GP3a followed by silica gel flash chromatography (70% EtOAc, hexanes) afforded the product as a yellow solid (1046 mg, 7.11 mmol, 81%, *E*/*Z*≈98:2).

^{1}H NMR (501 MHz, CD_2Cl_2): δ = 10.18 (d, *J* = 7.7 Hz, 1H), 8.61 (dd, *J* = 4.8, 1.6 Hz, 1H), 7.84 (ddd, *J* = 8.0, 2.5, 1.6 Hz, 1H), 7.35 (ddd, *J* = 8.0, 4.8, 0.9 Hz, 1H), 6.35 (dq, *J* = 7.6, 1.3 Hz, 1H), 2.57 (d, *J* = 1.3 Hz, 3H).

^{13}C NMR (126 MHz, CD_2Cl_2): δ = 191.0, 154.7, 151.2, 147.9, 136.6, 133.8, 128.5, 123.8, 16.4.

HRMS *m*/*z* (GC-EI): calcd. for $C_9H_9O_1N_1$ ($[M]^+$): 147.067864; found: 147.067810.

(*E*)-3-(thiophen-2-yl)but-2-enal (1l)

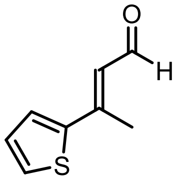

Preparation according to GP2b and GP3b followed by silica gel flash chromatography (10% EtOAc, hexanes) afforded the product as a yellow oil (414 mg, 2.72 mmol, 42%, *E*/*Z*≈98:2).

^{1}H NMR (501 MHz, CD_2Cl_2): δ = 10.11 (d, *J* = 7.8 Hz, 1H), 7.50–7.44 (m, 2H), 7.12 (dd, *J* = 4.9, 4.0 Hz, 1H), 6.42 (dd, *J* = 7.7, 1.2 Hz, 1H), 2.57 (d, *J* = 1.2 Hz, 3H).

^{13}C NMR (126 MHz, CD_2Cl_2): δ = 190.8, 150.1, 145.0, 129.2, 128.7, 128.3, 124.8, 16.3.

HRMS *m*/*z* (GC-EI): calcd. for $C_8H_8O_1S_1$ ($[M]^+$): 152.029037; found: 152.029150.

(*E*)-3-cyclohexylbut-2-enal (1m)

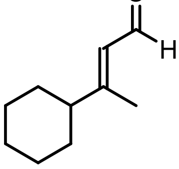

Preparation according to GP2a and GP3a followed by silica gel flash chromatography (10% Et_2O in pentane) afforded the product as a light yellow oil (546 mg, 3.69 mmol, 43%, *E*/*Z*>99:1).

^{1}H NMR (501 MHz, CD_2Cl_2): δ = 10.00 (d, *J* = 8.0 Hz, 1H), 5.82 (ddq, *J* = 8.0, 1.3, 1.3 Hz, 1H), 2.14 (d, *J* = 1.3 Hz, 3H), 2.04 (tt, *J* = 11.3, 3.2 Hz, 1H), 1.86–1.65 (m, 5H), 1.38–1.13 (m, 5H).

^{13}C NMR (126 MHz, CD_2Cl_2): δ = 191.9, 169.2, 126.0, 48.8, 31.6, 26.7, 26.5, 16.2.

HRMS *m/z* (GC-EI): calcd. for $C_{10}H_{16}O_1$ ([M]$^+$): 152.119565; found: 152.119540.

3-cyclopentylbut-2-enal (1n)

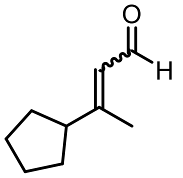

Preparation according to GP2a and GP3a followed by silica gel flash chromatography (5% Et_2O in pentane) afforded the product as a mixture of *E*/*Z* isomers as a light yellow oil (430 mg, 3.11 mmol, 38%, *E*/*Z*≈85:15).

^{1}H NMR (501 MHz, CD_2Cl_2): δ = 10.03 (d, *J* = 8.2 Hz, $1H_{min}$), 10.00 (d, *J* = 8.0 Hz, $1H_{maj}$), 5.86 (ddq, *J* = 8.0, 1.2, 1.2 Hz, $1H_{maj}$), 5.83–5.79 (m, $1H_{min}$), 3.71–3.62 (m, $1H_{min}$), 2.63–2.53 (m, $1H_{maj}$), 2.15 (d, *J* = 1.2 Hz, $3H_{maj}$), 1.91 (d, *J* = 1.2 Hz, $3H_{min}$), 1.90–1.82 (m, $2H_{maj}$), 1.76–1.68 (m, $2H_{maj}$), 1.68–1.60 (m, $2H_{maj}$), 1.53–1.43 (m, $2H_{maj}$).

^{13}C NMR (126 MHz, CD_2Cl_2): δ = 191.8, 190.4, 167.6, 129.1, 125.9, 50.2, 41.6, 32.2, 31.4, 26.5, 25.7, 20.5, 16.3.

HRMS *m/z* (GC-EI): calcd. for $C_9H_{14}O_1$ ([M]$^+$): 138.103915; found: 138.104000.

3-cyclobutylbut-2-enal (1o)

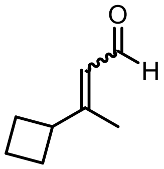

Preparation according to GP2a and GP3a followed by silica gel flash chromatography (10% Et_2O in pentane) afforded the product as a mixture of *E*/*Z* isomers as a light yellow oil (766 mg, 6.17 mmol, 67%, *E*/*Z*≈92:8).

^{1}H NMR (501 MHz, CD_2Cl_2): δ = 9.99 (d, *J* = 8.1 Hz, $1H_{maj}$), 9.93 (d, *J* = 8.4 Hz, $1H_{min}$), 5.78 (ddq, *J* = 8.2, 1.3, 1.3 Hz, $1H_{maj}$), 5.72 (ddq, *J* = 8.3, 1.4, 1.4 Hz, $1H_{min}$), 3.93 (p, *J* = 9.0 Hz, $1H_{min}$), 3.07 (p, *J* = 8.5 Hz, $1H_{maj}$), 2.25–2.10 (m, $2H_{maj}$), 2.08–2.05 (m, $3H_{maj}$), 2.04–1.89 (m, $3H_{maj}$), 1.82–1.69 (m, $1H_{maj}$).

^{13}C NMR (126 MHz, CD_2Cl_2): δ = 191.7, 190.9, 167.1, 127.8, 124.9, 44.4, 38.3, 28.8, 27.5, 22.0, 19.2, 17.9, 15.2.

HRMS *m/z* (GC-CI): calcd. for $C_8H_{13}O_1$ ([M+H]$^+$): 125.096090; found: 125.096180.

(*E*)-3,4,4-trimethylpent-2-enal (1p)

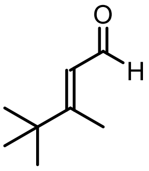

Preparation according to GP2a and GP3b followed by silica gel flash chromatography (5% Et_2O in pentane) afforded the product as a light yellow oil (468 mg, 3.71 mmol, 26%, *E*/*Z*>99:1).

^{1}H NMR (501 MHz, CD_2Cl_2): δ = 10.04 (d, *J* = 7.8 Hz, 1H), 5.91 (dq, *J* = 7.7, 1.2 Hz, 1H), 2.17 (d, *J* = 1.2 Hz, 3H), 1.13 (s, 9H).

^{13}C NMR (126 MHz, CD_2Cl_2): δ = 192.7, 171.4, 124.9, 38.1, 28.5, 13.9.

HRMS *m/z* (ESI): calcd. for $C_8H_{15}O_1$ ([M+H]$^+$): 127.111740; found: 127.111900.

3,4-dimethylpent-2-enal (1q)

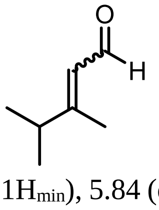

Preparation according to GP2a and GP3b followed by silica gel flash chromatography (5% Et_2O in pentane) afforded the product as a mixture of *E*/*Z* isomers as a light yellow oil (617 mg, 5.50 mmol, 52%, *E*/*Z*≈87:13).

^{1}H NMR (501 MHz, CD_2Cl_2): δ = 10.04 (d, *J* = 8.2 Hz, $1H_{min}$), 10.00 (d, *J* = 8.0 Hz, $1H_{min}$), 5.84 (dq, *J* = 8.0, 1.1 Hz, $1H_{maj}$), 5.74 (dq, *J* = 8.2, 1.4 Hz, $1H_{min}$), 3.63 (hept, *J* = 6.8 Hz, $1H_{min}$),

2.41 (hept, J = 6.9 Hz, $1H_{maj}$), 2.14 (d, J = 1.3 Hz, $3H_{maj}$), 1.88 (d, J = 1.3 Hz, $3H_{min}$), 1.13 (d, J = 6.9 Hz, $6H_{min}$), 1.10 (d, J = 6.9 Hz, $6H_{maj}$).

^{13}C NMR (126 MHz, CD_2Cl_2): δ = 191.9, 190.1, 169.8, 127.8, 125.6, 38.3, 29.8, 21.2, 20.9, 19.5, 15.4.

HRMS *m*/*z* (ESI): calcd. for $C_7H_{13}O_1$ ($[M+H]^+$): 113.096090; found: 113.096230.

3-methylpent-2-enal (1r)

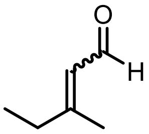

Preparation according to GP2a and GP3a followed by silica gel flash chromatography (5% Et_2O in pentane) afforded the product as a mixture of *E*/*Z* isomers as a colorless oil (517 mg, 5.27 mmol, 38%, *E*/*Z*≈78:22).

^{1}H NMR (501 MHz, CD_2Cl_2): δ = 9.99 (d, J = 8.0 Hz, $1H_{maj}$), 9.95 (d, J = 8.2 Hz, $1H_{min}$), 5.83 (dp, J = 8.1, 1.4 Hz, $1H_{maj}$), 5.79 (dd, J = 8.1, 1.4 Hz, $1H_{min}$), 2.59 (q, J = 7.6 Hz, $2H_{min}$), 2.24 (qd, J = 7.4, 1.3 Hz, $2H_{maj}$), 2.16 (d, J = 1.3 Hz, $3H_{maj}$), 1.97 (d, J = 1.3 Hz, $3H_{min}$), 1.16 (t, J = 7.6 Hz, $3H_{min}$), 1.10 (t, J = 7.4 Hz, $3H_{maj}$).

^{13}C NMR (126 MHz, CD_2Cl_2): δ = 191.6, 190.8, 166.7, 166.0, 127.8, 126.5, 33.8, 26.1, 24.6, 17.6, 13.8, 11.8.

HRMS *m*/*z* (ESI): calcd. for $C_6H_{11}O_1$ ($[M+H]^+$): 99.080440; found: 99.080550.

3-methylhex-2-enal (1s)

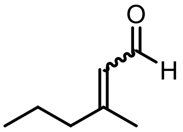

Preparation according to GP2a and GP3b followed by silica gel flash chromatography (5% Et_2O in pentane) afforded the product as a mixture of *E*/*Z* isomers as a light yellow oil (347 mg, 3.09 mmol, 47%, *E*/*Z*≈75:25).

^{1}H NMR (501 MHz, CD_2Cl_2): δ = 9.98 (d, J = 8.0 Hz, $1H_{maj}$), 9.94 (d, J = 8.3 Hz, $1H_{min}$), 5.87–5.79 (m, $1H_{maj}$, $1H_{min}$), 2.58–2.53 (m, $2H_{min}$), 2.19 (td, J = 7.6, 1.2 Hz, $2H_{maj}$), 2.14 (d, J = 1.3 Hz, $3H_{maj}$), 1.96 (d, J = 1.3 Hz, $3H_{min}$), 1.64–1.49 (m, $2H_{maj}$, $2H_{min}$), 0.99–0.89 (m, $3H_{maj}$, $3H_{min}$).

^{13}C NMR (126 MHz, CD_2Cl_2): δ = 191.5, 190.9, 164.9, 164.5, 128.9, 127.7, 43.0, 34.7, 25.0, 22.3, 20.8, 17.6, 13.9, 13.8.

HRMS *m*/*z* (ESI): calcd. for $C_7H_{13}O_1$ ($[M+H]^+$): 113.096090; found: 113.096150.

3-methylhept-2-enal (1t)

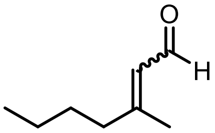

Preparation according to GP2a and GP3b followed by silica gel flash chromatography (5% Et_2O in pentane) afforded the product as a mixture of *E*/*Z* isomers as a light yellow oil (599 mg, 4.74 mmol, 62%, *E*/*Z*≈74:26).

^{1}H NMR (501 MHz, CD_2Cl_2): δ = 9.97 (d, J = 8.1 Hz, $1H_{maj}$), 9.94 (d, J = 8.2 Hz, $1H_{min}$), 5.86–5.79 (m, $1H_{maj}$, $1H_{min}$), 2.61–2.54 (m, $2H_{min}$), 2.24–2.18 (m, $2H_{maj}$), 2.15 (d, J = 1.3 Hz, $3H_{maj}$), 1.96 (d, J = 1.3 Hz, $3H_{min}$), 1.58–1.44 (m, $2H_{maj}$, $2H_{min}$), 1.43–1.28 (m, $2H_{maj}$, $2H_{min}$), 0.97–0.88 (m, $3H_{maj}$, $3H_{min}$).

^{13}C NMR (126 MHz, CD_2Cl_2): δ = 191.5, 190.9, 165.3, 164.8, 128.6, 127.5, 40.7, 32.7, 31.4, 29.7, 25.1, 23.0, 22.7, 17.6, 14.0.

HRMS *m*/*z* (ESI): calcd. for $C_8H_{15}O_1$ ($[M+H]^+$): 127.111740; found: 127.111880.

(*E*)-4,4,4-trifluoro-3-phenylbut-2-enal ((*E*)-1w)

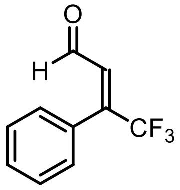

Preparation according to GP2a and GP3a followed by silica gel flash chromatography (5% Et_2O in pentane) afforded the product as a light yellow oil (562 mg, 2.81 mmol, 90%, *E*/*Z*≈98:2).

^{1}H NMR (501 MHz, CD_2Cl_2): δ = 9.55 (dd, *J* = 7.5, 0.7 Hz, 1H), 7.58–7.48 (m, 3H), 7.45 – 7.40 (m, 2H), 6.64 (dq, *J* = 7.6, 1.3 Hz, 1H).

^{19}F{^{1}H} NMR (471 MHz, CD_2Cl_2): δ = –67.69.

^{13}C NMR (126 MHz, CD_2Cl_2): δ = 192.0, 147.9 (q, *J* = 31.6 Hz), 131.5 (q, *J* = 4.9 Hz), 130.9, 130.4, 129.2, 123.34 (q, *J* = 274.7 Hz).

HRMS *m/z* (GC-EI): calcd. for $C_{10}H_7O_1F_3$ ($[M]^+$): 200.044351; found: 200.044270.

(*Z*)-4,4,4-trifluoro-3-phenylbut-2-enal ((*Z*)-1w)

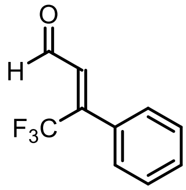

Preparation according to GP2a and GP3a followed by silica gel flash chromatography (5% Et_2O in pentane) afforded the product as a light yellow oil (48 mg, 0.24 mmol, 84%, *E*/*Z*≈4:96).

^{1}H NMR (501 MHz, CD_2Cl_2): δ = 10.20 (dq, *J* = 7.4, 2.2 Hz, 1H), 7.53–7.42 (m, 5H), 6.38 (d, *J* = 7.4 Hz, 1H).

^{19}F{^{1}H} NMR (471 MHz, CD_2Cl_2): δ = –55.61.

^{13}C NMR (126 MHz, CD_2Cl_2): δ = 190.0, 135.4, 135.4, 133.6, 130.8, 129.2, 128.3.

HRMS *m/z* (GC-EI): calcd. for $C_{10}H_7O_1F_3$ ($[M]^+$): 200.044351; found: 200.044440.

3-phenylpent-2-enal (1x)

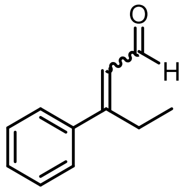

Preparation according to GP2a and GP3b followed by silica gel flash chromatography (5% Et_2O in pentane) afforded the product as a light yellow oil (693 mg, 4.33 mmol, 65%, *E*/*Z*≈82:18). The NMR-spectroscopic data are in agreement with the literature.[14]

^{1}H NMR (501 MHz, $CDCl_3$): δ = 10.16 (d, *J* = 8.0 Hz, $1H_{maj}$), 9.45 (d, *J* = 8.1 Hz, $1H_{min}$), 7.55–7.25 (m, $5H_{maj,}$ $5H_{maj}$), 6.25 (d, *J* = 8.0 Hz, $1H_{maj}$), 6.11 (dt, *J* = 8.0, 1.4 Hz, $1H_{min}$), 3.07 (q, *J* = 7.6 Hz, $2H_{maj}$), 2.60 (qd, *J* = 7.4, 1.4 Hz, $2H_{min}$), 1.18 (t, *J* = 7.6 Hz, $3H_{maj}$), 1.11 (t, *J* = 7.4 Hz, $3H_{min}$).

HRMS *m/z* (EI): calcd. for $C_{11}H_{12}O_1$ ($[M]^+$): 160.088265; found: 160.088360.

(*E*)-3-phenylhept-2-enal (1y)

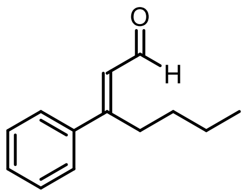

Preparation according to GP2a and GP3b followed by silica gel flash chromatography (2–5% Et_2O in pentane) afforded the product as a light yellow oil (1.40 g, 7.43 mmol, 86%, *E*/*Z*>99:1).The NMR-spectroscopic data are in agreement with the literature.[15]

^{1}H NMR (501 MHz, $CDCl_3$): δ = 10.15 (d, *J* = 8.0 Hz, 1H), 7.52–7.46 (m, 2H), 7.44–7.38 (m, 3H), 6.28 (d, *J* = 8.0 Hz, 1H), 3.08–3.00 (m, 2H), 1.54–1.45 (m, 2H), 1.44–1.31 (m, 2H), 0.90 (t, *J* = 7.3 Hz, 3H).

HRMS *m/z* (ESI): calcd. for $C_{13}H_{16}O_1Na_1$ ($[M+Na]^+$): 211.109334; found: 211.109550.

(*E*)-3-phenylbut-2-enal-4,4,4-*d*$_3$ ((*E*)-1d')

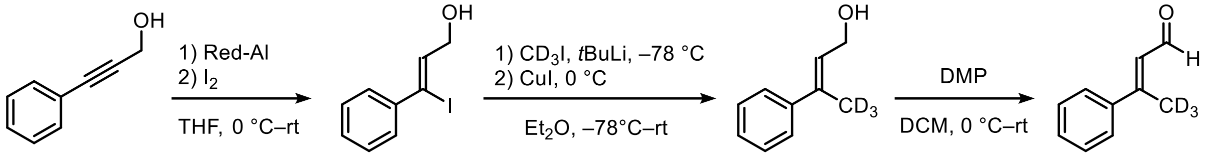

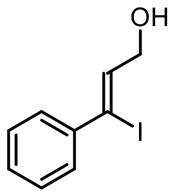

In a flame-dried Schlenk under argon 3-phenylprop-2-yn-1-ol (0.93 mL, 7.5 mmol, 1.0 equiv) was dissolved in dry THF (30 mL) and cooled to 0 °C. After dropwise addition of Red-Al (2.7 mL, 8.2 mmol, 1.1 equiv, 60% in toluene) the reaction mixture was stirred for 4 h at 0 °C. Then iodine (2.08 g, 8.21 mmol, 1.10 equiv) was added and the reaction mixture was stirred for 15 minutes while slowly warming to room temperature. The reaction was terminated by addition of an aqueous Rochelle's salt solution followed by saturated Na_2SO_3 solution. After separation of the organic phase, the aqueous layer was extracted with EtOAc (3x). The combined organic phases were washed with water, dried over anhydrous Na_2SO_4 and then concentrated under reduced pressure. Purification via column chromatography (10–20% EtOAc in hexanes) furnished (*Z*)-3-iodo-3-phenylprop-2-en-1-ol (1.54 g, 5.91 mmol, 79%). The NMR-spectroscopic data are in agreement with the literature.[16]

^{1}H NMR (501 MHz, $CDCl_3$): δ = 7.49–7.45 (m, 2H), 7.34–7.27 (m, 3H), 6.26 (t, *J* = 5.7 Hz, 1H), 4.40 (d, *J* = 5.7 Hz, 2H), 1.71 (s, 1H).

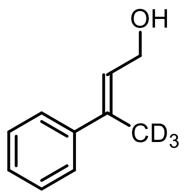

In a flame-dried Schlenk under argon CD_3I (0.68 mL, 11 mmol, 5.0 equiv) was dissolved in dry Et_2O (20 mL) and cooled to –78 °C. After dropwise addition of *t*BuLi (12.9 mL, 21.9 mmol, 10.0 equiv) the solution was stirred for 30 minutes and then warmed to 0 °C. CuI (1.04 g, 5.48 mmol, 2.50 equiv) was added and the reaction mixture was stirred for additional 30 minutes and then cooled down to –78 °C. (*Z*)-3-iodo-3-phenylprop-2-en-1-ol (570 mg, 2.19 mmol, 1.00 equiv) dissolved in dry Et_2O (7 mL) was added dropwise. The reaction was gradually warmed to 0 °C and stirred for 1 h. Saturated NH_4Cl solution was added, followed by extraction with EtOAc (3x). The combined organic phases were washed with water, dried over anhydrous Na_2SO_4 and concentrated under reduced pressure. Purification via column chromatography (10–20% EtOAc in hexanes) yielded the product as an inseparable mixture of desired (*E*)-3-phenylbut-2-en-4,4,4-*d*$_3$-1-ol (85% purity, 283 mg, 1.59 mmol, 73%) and deiodinated side product (*E*)-3-phenylprop-2-en-1-ol. The NMR-spectroscopic data are in agreement with the literature.[17]

^{1}H NMR (501 MHz, $CDCl_3$): δ = 7.43–7.38 (m, 2H), 7.36–7.31 (m, 2H), 7.28–7.24 (m, 1H), 5.99 (t, *J* = 6.7 Hz, 1H), 4.37 (d, *J* = 6.5 Hz, 2H).

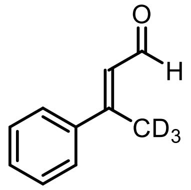

In a flame-dried Schlenk under argon (*E*)-3-phenylbut-2-en-4,4,4-*d*$_3$-1-ol (85% purity, 283 mg, 1.59 mmol, 1.00 equiv) was dissolved in dichloromethane (9 mL) and cooled to 0 °C. Dess-Martin periodinane (873 mg, 2.06 mmol, 1.29 equiv) was added in one portion and the reaction mixture was stirred overnight while warming to room temperature. The organic phase was sequentially washed with saturated $NaHCO_3$ solution and water (2x). The organic layer was dried over anhydrous Na_2SO_4 and concentrated under reduced pressure. Purification via column chromatography (3–5% Et_2O, hexanes) furnished the desired (*E*)-3-phenylbut-2-enal-4,4,4-*d*$_3$ (68 mg, 0.456 mmol, 29%). The NMR-spectroscopic data are in agreement with the literature.[18]

^{1}H NMR (501 MHz, CD_2Cl_2): δ = 10.16 (d, *J* = 7.9 Hz, 1H), 7.61–7.52 (m, 2H), 7.45–7.39 (m, 3H), 6.36 (d, *J* = 7.8 Hz, 1H).

5. Transfer Hydrogenation of α,β-Unsaturated Aldehydes

General procedure 4 (GP4)

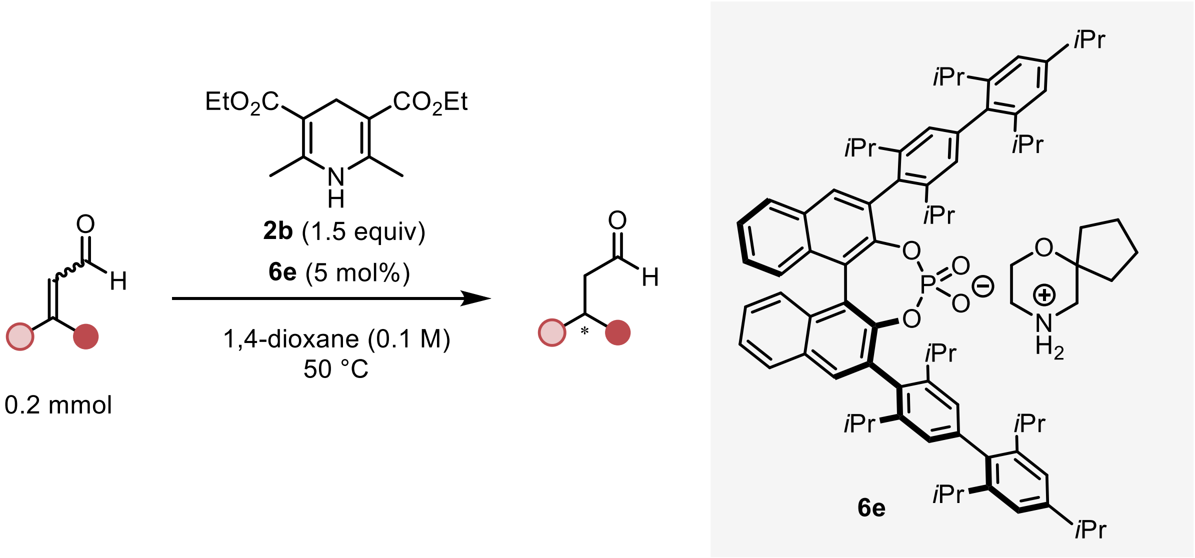

In a flame-dried Schlenk flask under argon the respective α,β-unsaturated aldehyde (1.00 equiv, 0.20 mmol) and the preformed catalyst **6e** (5 mol%, 0.01 mmol) were dissolved in dry 1,4-dioxane (2 mL) and heated to 50 °C. After five minutes Hantzsch ester **2b** (1.5 equiv, 0.30 mmol) was added and the resulting reaction mixture was stirred at 50 °C for 24 h. After cooling to ambient temperature, the mixture was directly loaded onto a column packed with silica and eluted with DCM/pentane to give the β-chiral aldehyde products. For deviations from the general procedure, see the respective entries.

Racemate syntheses

For the racemate syntheses, the respective α,β-unsaturated aldehyde (0.03 mmol, 1.00 equiv) was reacted with Hantzsch ester **2b** (45.0 µmol, 1.50 equiv) and morpholinium diphenyl phosphate (20 mol%, 6 µmol) in dry 1,4-dioxane (0.3 mL) at 50 °C for 10–24 h. Subsequent thin-layer chromatography gave the isolated racemic products that were used for the determination of the enantiomeric ratio by GC-analysis.

(*R*)-3-(4-bromophenyl)butanal (3a)

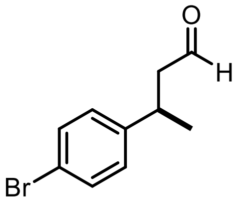

Following GP4 the reaction was performed using enal **1a** (45.2 mg, 0.20 mmol, 1.00 equiv). Purification by silica gel flash chromatography (20–50% DCM in pentane) afforded the product **3a** (38.7 mg, 0.17 mmol, 85%) as a light yellow oil.

^{1}H NMR (501 MHz, CD_2Cl_2): δ = 9.67 (t, *J* = 1.8 Hz, 1H), 7.47–7.40 (m, 2H), 7.16–7.09 (m, 2H), 3.32 (h, *J* = 7.1 Hz, 1H), 2.72 (ddd, *J* = 16.9, 7.1, 1.8 Hz, 1H), 2.65 (ddd, *J* = 17.0, 7.4, 1.9 Hz, 1H), 1.28 (d, *J* = 7.0 Hz, 3H).

^{13}C NMR (126 MHz, CD_2Cl_2): δ = 201.5, 145.3, 132.0, 129.1, 120.3, 51.9, 34.1, 22.2.

HRMS *m/z* (EI): calcd. for $C_{10}H_{11}O_1Br_1$ ($[M]^+$): 225.998790; found: 225.998770.

GC (30 m Ivadex-1, injection temperature: 220 °C, 110 °C iso 5 min, 2 °C/min, 150 °C iso 5 min, 5 °C/min, 200 °C iso 30 min, 5 °C/min, 220 °C iso 5 min, 0.5 bar H_2): t_{R1} = 30.1 min (major), t_{R2} = 30.6 min (minor), er = 97.5:2.5 (95% ee).

$[\alpha]_D^{25}$ = –28.5 (c= 0.20, $CHCl_3$)

(*R*)-3-(3-bromophenyl)butanal (3b)

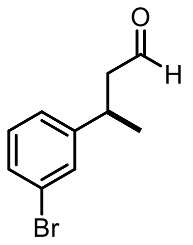

Following GP4 the reaction was performed using enal **1b** (44.9 mg, 0.20 mmol, 1.00 equiv). Purification by silica gel flash chromatography (20–50% DCM in pentane) afforded the product **3b** (38.0 mg, 0.17 mmol, 84%) as a yellow oil.

^{1}H NMR (501 MHz, CD_2Cl_2): δ = 9.68 (t, *J* = 1.8 Hz, 1H), 7.39 (t, *J* = 1.8 Hz, 1H), 7.35 (dt, *J* = 7.1, 2.0 Hz, 1H), 7.23–7.15 (m, 2H), 3.33 (h, *J* = 7.0 Hz, 1H), 2.74 (ddd, *J* = 17.0, 7.0, 1.7 Hz, 1H), 2.66 (ddd, *J* = 17.0, 7.4, 1.9 Hz, 1H), 1.28 (d, *J* = 7.0 Hz, 3H).

^{13}C NMR (126 MHz, CD_2Cl_2): δ = 201.4, 148.7, 130.6, 130.4, 129.9, 126.1, 122.9, 51.8, 34.3, 22.2.

HRMS *m/z* (EI): calcd. for $C_{10}H_{11}O_1Br_1$ ($[M]^+$): 225.998790; found: 225.998640.

GC (30 m Ivadex-1, injection temperature: 220 °C, 110 °C iso 5 min, 2 °C/min, 150 °C iso 5 min, 8 °C/min, 220 °C iso 5 min, 0.5 bar H_2): t_{R1} = 26.8 min (major), t_{R2} = 27.2 min (minor), er = 97.5:2.5 (95% ee).

$[\alpha]_D^{25}$ = –25.3 (c= 0.17, $CHCl_3$)

(*R*)-3-(3-(trifluoromethyl)phenyl)butanal (3c)

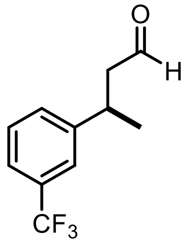

Following GP4 the reaction was performed using enal **1c** (35 µL, 0.20 mmol, 1.00 equiv). Purification by silica gel flash chromatography (20–50% DCM in pentane) afforded the product **3c** (38.5 mg, 0.18 mmol, 89%) as a light yellow oil.

^{1}H NMR (501 MHz, CD_2Cl_2): δ = 9.70 (t, *J* = 1.7 Hz, 1H), 7.52–7.41 (m, 4H), 3.44 (h, *J* = 7.1 Hz, 1H), 2.78 (ddd, *J* = 17.1, 6.9, 1.7 Hz, 1H), 2.71 (ddd, *J* = 17.1, 7.4, 1.8 Hz, 1H), 1.32 (d, *J* = 7.0 Hz, 3H).

^{13}C NMR (126 MHz, CD_2Cl_2): δ = 201.2, 147.3, 131.0 (q, *J* = 32.0 Hz), 131.0, 129.6, 128.0, 125.8, 124.8 (q, *J* = 274.7 Hz), 124.0 (q, *J* = 3.9 Hz), 123.7 (q, *J* = 3.9 Hz), 51.8, 34.4, 22.2.

^{19}F{^{1}H} NMR (471 MHz, CD_2Cl_2): δ = –62.9.

HRMS *m/z* (EI): calcd. for $C_{11}H_{11}O_1F_3$ ($[M]^+$): 216.075650; found: 216.075790.

GC (30 m Ivadex-1, injection temperature: 220 °C, 110 °C iso 5 min, 2 °C/min, 150 °C iso 5 min, 8 °C/min, 220 °C iso 5 min, 0.5 bar H_2): t_{R1} = 10.9 min (major), t_{R2} = 11.3 min (minor), er = 98:2 (96% ee).

$[\alpha]_D^{25}$ = –24.3 (c= 0.32, $CHCl_3$)

(*R*)-3-phenylbutanal (3d)

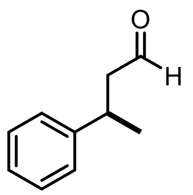

Following GP4 the reaction was performed using enal **1d** (28 µL, 0.20 mmol, 1.00 equiv). Purification by silica gel flash chromatography (20–50% DCM in pentane) afforded the product **3d** (21.6 mg, 0.15 mmol, 72%) as a light yellow oil.

^{1}H NMR (501 MHz, CD_2Cl_2): δ = 9.68 (t, *J* = 2.0 Hz, 1H), 7.33–7.28 (m, 2H), 7.26–7.18 (m, 3H), 3.35 (h, *J* = 7.1 Hz, 1H), 2.74 (ddd, *J* = 16.7, 7.1, 1.9 Hz, 1H), 2.65 (ddd, *J* = 16.7, 7.5, 2.1 Hz, 1H), 1.30 (d, *J* = 7.0 Hz, 3H).

^{13}**C NMR** (126 MHz, CD_2Cl_2): δ = 202.0, 146.2, 129.0, 127.2, 126.8, 52.1, 34.7, 22.4.

HRMS *m/z* (GC-EI): calcd. for $C_{10}H_{12}O_1$ ($[M]^+$): 148.088265; found: 148.088520.

GC (30 m Ivadex-1, injection temperature: 220 °C, 110 °C iso 5 min, 2 °C/min, 150 °C iso 5 min, 8 °C/min, 220 °C iso 5 min, 0.5 bar H_2): t_{R1} = 11.5 min (major), t_{R2} = 12.0 min (minor), er = 97:3 (94% ee).

$[\boldsymbol{\alpha}]_{\boldsymbol{D}}^{\mathbf{25}}$ = –37.0 (c= 0.15, $CHCl_3$)

The absolute configuration was determined by comparison with the previously reported optical rotation $[\boldsymbol{\alpha}]_{\boldsymbol{D}}^{\mathbf{20}}$ = –33.2 (c= 0.74, $CHCl_3$) of the (*R*) enantiomer (90% ee).[19] The absolute stereochemistry of further substrates was assigned as (*R*) by analogy.

(*R*)-3-(p-tolyl)butanal (3e)

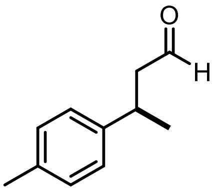

Following GP4 the reaction was performed using enal **1e** (32 µL, 0.20 mmol, 1.00 equiv). Purification by silica gel flash chromatography (20–50% DCM in pentane) afforded the product **3e** (30.6 mg, 0.19 mmol, 93%) as a light yellow oil.

^{1}**H NMR** (501 MHz, CD_2Cl_2): δ = 9.67 (t, *J* = 2.1 Hz, 1H), 7.12 (s, 4H), 3.30 (h, *J* = 7.1 Hz, 1H), 2.70 (ddd, *J* = 16.6, 7.2, 2.0 Hz, 1H), 2.62 (ddd, *J* = 16.6, 7.4, 2.2 Hz, 1H), 2.31 (s, 3H), 1.28 (d, *J* = 7.0 Hz, 4H).

^{13}**C NMR** (126 MHz, CD_2Cl_2): δ = 202.2, 143.1, 136.4, 129.6, 127.0, 52.1, 34.3, 22.5, 21.1.

HRMS *m/z* (EI): calcd. for $C_{11}H_{14}O_1$ ($[M]^+$): 162.103915; found: 162.104070.

GC (30 m Ivadex-1, injection temperature: 220 °C, 110 °C iso 5 min, 2 °C/min, 150 °C iso 5 min, 8 °C/min, 220 °C iso 5 min, 0.5 bar H_2): t_{R1} = 15.9 min (major), t_{R2} = 16.3 min (minor), er = 96.5:3.5 (93% ee).

$[\boldsymbol{\alpha}]_{\boldsymbol{D}}^{\mathbf{25}}$ = –33.1 (c= 0.33, $CHCl_3$)

(*R*)-3-(m-tolyl)butanal (3f)

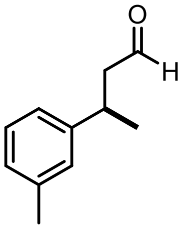

Following GP4 the reaction was performed using enal **1f** (31 µL, 0.20 mmol, 1.00 equiv). Purification by silica gel flash chromatography (20–50% DCM in pentane) afforded the product **3f** (26.7 mg, 0.17 mmol, 82%) as a light yellow oil.

^{1}**H NMR** (501 MHz, CD_2Cl_2): δ = 9.67 (t, *J* = 2.0 Hz, 1H), 7.19 (t, *J* = 7.5 Hz, 1H), 7.06–6.99 (m, 3H), 3.30 (h, *J* = 7.1 Hz, 1H), 2.72 (ddd, *J* = 16.6, 7.1, 1.9 Hz, 1H), 2.63 (ddd, *J* = 16.6, 7.5, 2.1 Hz, 1H), 2.33 (s, 3H), 1.28 (d, *J* = 7.0 Hz, 4H).

^{13}**C NMR** (126 MHz, CD_2Cl_2): δ = 202.2, 146.1, 138.7, 128.8, 128.0, 127.5, 124.2, 52.0, 34.7, 22.5, 21.6.

HRMS *m/z* (EI): calcd. for $C_{11}H_{14}O_1$ ($[M]^+$): 162.103915; found: 162.104170.

GC (30 m Ivadex-1, injection temperature: 220 °C, 110 °C iso 5 min, 2 °C/min, 150 °C iso 5 min, 8 °C/min, 220 °C iso 5 min, 0.5 bar H_2): t_{R1} = 15.0 min (major), t_{R2} = 15.3 min (minor), er = 95.5:4.5 (91% ee).

$[\boldsymbol{\alpha}]_{\boldsymbol{D}}^{\mathbf{25}}$ = –31.9 (c= 0.18, $CHCl_3$)

(*R*)-3-(o-tolyl)butanal (3g)

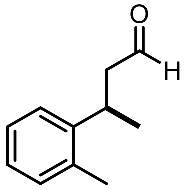

Following GP4 the reaction was performed using enal **1g** (32 µL, 0.20 mmol, 1.00 equiv). Purification by silica gel flash chromatography (20–50% DCM in pentane) afforded the product **3g** (20.6 mg, 0.13 mmol, 63%) as a light yellow oil.

^{1}H NMR (501 MHz, CD_2Cl_2): δ = 9.69 (t, *J* = 1.9 Hz, 1H), 7.19–7.13 (m, 3H), 7.12–7.07 (m, 1H), 3.64–3.54 (m, 1H), 2.76 (ddd, *J* = 16.8, 6.7, 1.8 Hz, 1H), 2.66 (ddd, *J* = 16.8, 7.8, 2.1 Hz, 1H), 2.37 (s, 3H), 1.25 (d, *J* = 6.9 Hz, 3H).

^{13}C NMR (126 MHz, CD_2Cl_2): δ = 202.0, 144.3, 135.6, 130.8, 126.7, 126.5, 125.6, 51.5, 29.6, 21.8, 19.6.

HRMS *m/z* (EI): calcd. for $C_{11}H_{14}O_1$ ($[M]^+$): 162.103915; found: 162.103960.

GC (30 m Ivadex-1, injection temperature: 220 °C, 110 °C iso 30 min, 2 °C/min, 130 °C iso 2 min, 8 °C/min, 220 °C iso 5 min, 0.5 bar H_2): t_{R1} = 19.8 min (major), t_{R2} = 20.5 min (minor), er = 98.5:1.5 (97% ee).

$[\alpha]_D^{25}$ = –35.3 (c= 0.17, $CHCl_3$)

(*R*)-3-(4-((triisopropylsilyl)oxy)phenyl)butanal (3h)

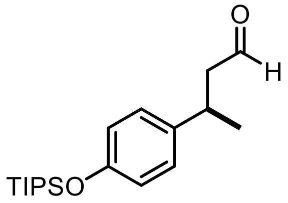

Following GP4 the reaction was performed using enal **1h** (63.4 mg, 0.20 mmol, 1.00 equiv). Purification by silica gel flash chromatography (20–50% DCM in pentane) afforded the product **3h** (52.8 mg, 0.17 mmol, 83%) as a light yellow oil.

^{1}H NMR (501 MHz, CD_2Cl_2): δ = 9.66 (t, *J* = 2.1 Hz, 1H), 7.10–7.03 (m, 2H), 6.85–6.78 (m, 2H), 3.28 (h, *J* = 7.1 Hz, 1H), 2.67 (ddd, *J* = 16.5, 7.0, 2.0 Hz, 1H), 2.59 (ddd, *J* = 16.5, 7.5, 2.3 Hz, 1H), 1.28–1.19 (m, 6H), 1.09 (d, *J* = 7.4 Hz, 18H).

^{13}C NMR (126 MHz, CD_2Cl_2): δ = 202.3, 154.9, 138.5, 128.0, 120.2, 52.3, 34.0, 22.5, 18.1, 13.1.

HRMS *m/z* (GC-EI): calcd. for $C_{19}H_{32}O_2Si_1$ ($[M]^+$): 320.216609; found: 320.216360.

GC (25 m Ivadex-1, injection temperature: 220 °C, 140 °C iso 150 min, 8 °C/min, 220 °C iso 3 min, 0.5 bar H_2): t_{R1} = 135.9 min (major), t_{R2} = 137.8 min (minor), er = 97.5:2.5 (95% ee).

$[\alpha]_D^{25}$ = –10.2 (c= 0.20, $CHCl_3$)

(*R*)-3-(4-vinylphenyl)butanal (3i)

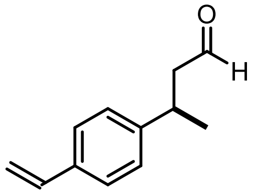

Following GP4 the reaction was performed using enal **1i** (34.2 mg, 0.20 mmol, 1.00 equiv). Purification by silica gel flash chromatography (20–50% DCM in pentane) afforded the product **3i** (30.1 mg, 0.17 mmol, 87%) as a light yellow oil.

^{1}H NMR (501 MHz, CD_2Cl_2): δ = 9.68 (t, *J* = 2.0 Hz, 1H), 7.40–7.33 (m, 2H), 7.23–7.16 (m, 2H), 6.70 (dd, *J* = 17.6, 10.9 Hz, 1H), 5.72 (dd, *J* = 17.5, 0.9 Hz, 1H), 5.21 (dd, *J* = 11.0, 0.9 Hz, 1H), 3.34 (h, *J* = 7.1 Hz, 1H), 2.73 (ddd, *J* = 16.7, 7.1, 1.9 Hz, 1H), 2.65 (ddd, *J* = 16.7, 7.5, 2.1 Hz, 1H), 1.29 (d, *J* = 7.0 Hz, 3H).

^{13}C NMR (126 MHz, CD_2Cl_2): δ = 201.9, 145.9, 136.9, 136.3, 127.4, 126.8, 113.5, 52.0, 34.4, 22.3.

HRMS *m/z* (EI): calcd. for $C_{12}H_{14}O_1$ ($[M]^+$): 174.103915; found: 174.104050.

GC (30 m Ivadex-1, injection temperature: 220 °C, 110 °C iso 5 min, 2 °C/min, 150 °C iso 5 min, 8 °C/min, 220 °C iso 5 min, 0.5 bar H_2): t_{R1} = 24.2 min (major), t_{R2} = 24,6 min (minor), er = 97:3 (94% ee).

$[\alpha]_D^{25}$ = –45.5 (c= 0.22, $CHCl_3$)

(*R*)-3-(4-methoxy-3-nitrophenyl)butanal (3j)

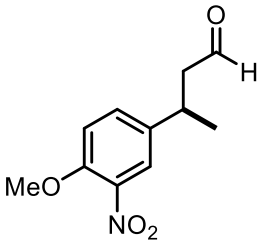

Following GP4 the reaction was performed using enal **1j** (44.0 mg, 0.20 mmol, 1.00 equiv). Purification by silica gel flash chromatography (5–20% EtOAc in hexanes) afforded the product **3j** (38.2 mg, 0.17 mmol, 86%) as a yellow oil.

^{1}H NMR (501 MHz, CD_2Cl_2): δ = 9.69 (t, *J* = 1.7 Hz, 1H), 7.69 (d, *J* = 2.4 Hz, 1H), 7.43 (dd, *J* = 8.7, 2.4 Hz, 1H), 7.06 (d, *J* = 8.7 Hz, 1H), 3.92 (s, 3H), 3.38 (h, *J* = 7.1 Hz, 1H), 2.75 (ddd, *J* = 17.2, 7.1, 1.6 Hz, 1H), 2.69 (ddd, *J* = 17.2, 7.2, 1.7 Hz, 1H), 1.30 (d, *J* = 7.0 Hz, 3H).

^{13}C NMR (126 MHz, CD_2Cl_2): δ = 201.1, 151.8, 140.0, 138.6, 133.2, 124.0, 114.3, 57.0, 51.9, 33.4, 22.2.

HRMS *m/z* (GC-EI): calcd. for $C_{11}H_{13}N_1O_4$ ($[M]^+$): 223.083909; found: 223.083860.

GC (30 m BGB-176, injection temperature: 220 °C, 130 °C iso 410 min, 8 °C/min, 240 °C, 0.6 bar H_2): t_{R1} = 380.8 min (major), t_{R2} = 390.5 min (minor), er = 96.5:3.5 (93% ee).

$[\alpha]_D^{25}$ = –18.2 (c= 0.20, $CHCl_3$)

(*R*)-3-(pyridin-3-yl)butanal (3k)

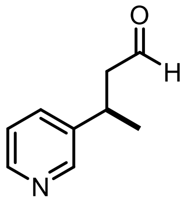

Following GP4 the reaction was performed using enal **1k** (30.4 mg, 0.21 mmol, 1.00 equiv). Purification by silica gel flash chromatography (20–50% EtOAc in hexanes) afforded the product **3k** (24.7 mg, 0.17 mmol, 80%) as a light yellow oil.

^{1}H NMR (501 MHz, CD_2Cl_2): δ = 9.70 (t, *J* = 1.7 Hz, 1H), 8.49 (d, *J* = 2.4 Hz, 1H), 8.43 (dd, *J* = 4.8, 1.7 Hz, 1H), 7.55 (dt, *J* = 7.8, 2.0 Hz, 1H), 7.24 (ddd, *J* = 7.9, 4.8, 0.9 Hz, 1H), 3.38 (h, *J* = 7.1 Hz, 1H), 2.77 (ddd, *J* = 17.2, 7.0, 1.6 Hz, 1H), 2.71 (ddd, *J* = 17.2, 7.4, 1.8 Hz, 1H), 1.32 (d, *J* = 7.0 Hz, 3H).

^{13}C NMR (126 MHz, CD_2Cl_2): δ = 201.1, 149.2, 148.2, 141.4, 134.5, 123.9, 51.7, 32.1, 22.1.

HRMS *m/z* (ESI): calcd. for $C_9H_{12}N_1O_1$ ($[M+H]^+$): 150.091339; found: 150.091310.

GC (30 m Ivadex-1, injection temperature: 220 °C, 110 °C iso 5 min, 2 °C/min, 150 °C iso 5 min, 8 °C/min, 220 °C iso 5 min, 0.5 bar H_2): t_{R1} = 19.0 min (major), t_{R2} = 19.5 min (minor), er = 97:3 (94% ee).

$[\alpha]_D^{25}$ = –15.8 (c= 0.20, $CHCl_3$)

(*R*)-3-(thiophen-2-yl)butanal (3l)

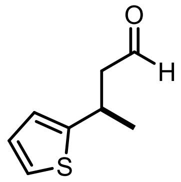

Following GP4 the reaction was performed using enal **1l** (26 μL, 0.20 mmol, 1.00 equiv) in CyH at room temperature for 4 days. Purification by silica gel flash chromatography (50% DCM in pentane) afforded the product **3l** (24.2 mg, 0.16 mmol, 78%) as a yellow oil.

^{1}H NMR (501 MHz, CD_2Cl_2): δ = 9.71 (t, *J* = 1.8 Hz, 1H), 7.16 (dd, *J* = 5.1, 1.2 Hz, 1H), 6.93 (dd, *J* = 5.1, 3.5 Hz, 1H), 6.85 (dt, *J* = 3.5, 1.1 Hz, 1H), 3.67 (hd, *J* = 7.0, 0.8 Hz, 1H), 2.79 (ddd, *J* = 16.9, 6.9, 1.8 Hz, 1H), 2.68 (ddd, *J* = 17.0, 7.2, 1.9 Hz, 1H), 1.38 (d, *J* = 7.0 Hz, 4H).

^{13}C NMR (126 MHz, CD_2Cl_2): δ = 201.4, 150.2, 127.1, 123.5, 123.4, 52.7, 30.2, 23.2.

HRMS *m/z* (GC-EI): calcd. for $C_8H_{10}O_1S_1$ ($[M]^+$): 154.044687; found: 154.044850.

GC (30 m Ivadex-1, injection temperature: 220 °C, 110 °C iso 5 min, 2 °C/min, 150 °C iso 5 min, 8 °C/min, 220 °C iso 5 min, 0.5 bar H_2): t_{R1} = 11.7 min (major), t_{R2} = 12.1 min (minor), er = 94:6 (88% ee).

$[\boldsymbol{\alpha}]_{\boldsymbol{D}}^{\boldsymbol{25}}$ = –27.8 (c= 0.23, $CHCl_3$)

(*R*)-3-cyclohexylbutanal (3m)

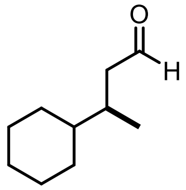

Following GP4 the reaction was performed using enal **1m** (31 μL, 0.20 mmol, 1.00 equiv). Purification by silica gel flash chromatography (20–50% DCM in pentane) afforded the product **3m** (22.9 mg, 0.15 mmol, 73%) as a colorless oil.

^{1}H NMR (501 MHz, CD_2Cl_2): δ = 9.72 (dd, *J* = 2.9, 1.8 Hz, 1H), 2.44 (ddd, *J* = 16.0, 4.8, 1.9 Hz, 1H), 2.16 (ddd, *J* = 16.0, 8.9, 2.9 Hz, 1H), 1.99–1.88 (m, 1H), 1.80–1.70 (m, 2H), 1.69–1.59 (m, 3H), 1.29–1.07 (m, 4H), 1.07–0.93 (m, 2H), 0.90 (d, *J* = 6.9 Hz, 3H).

^{13}C NMR (126 MHz, CD_2Cl_2): δ = 203.6, 48.9, 43.2, 33.5, 30.7, 29.6, 27.1, 27.1, 27.0, 17.0.

HRMS *m/z* (ESI): calcd. for $C_{10}H_{18}Na_1O_1$ ($[M+Na]^+$): 177.12499; found: 177.12493.

GC (30 m Ivadex-1, injection temperature: 220 °C, 110 °C iso 5 min, 2 °C/min, 150 °C iso 5 min, 8 °C/min, 220 °C iso 5 min, 0.5 bar H_2): t_{R1} = 11.3 min (major), t_{R2} = 11.6 min (minor), er = 98.5:1.5 (97% ee).

$[\boldsymbol{\alpha}]_{\boldsymbol{D}}^{\boldsymbol{25}}$ = –20.6 (c= 0.24, $CHCl_3$)

(*R*)-3-cyclopentylbutanal (3n)

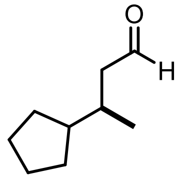

Following GP4 the reaction was performed using enal **1n** (30 μL, 0.20 mmol, 1.00 equiv). ^{1}H NMR yield was determined to be 87% using mesitylene as internal standard. Purification by silica gel flash chromatography (20–50% DCM in pentane) afforded the product **3n** (21.8 mg, 0.16 mmol, 77%) as a light yellow oil.

^{1}H NMR (501 MHz, CD_2Cl_2): δ = 9.73 (dd, *J* = 3.0, 1.8 Hz, 1H), 2.48 (ddd, *J* = 15.9, 4.3, 1.7 Hz, 1H), 2.19 (ddd, *J* = 16.0, 9.0, 3.0 Hz, 1H), 1.95–1.85 (m, 1H), 1.80–1.70 (m, 2H), 1.70–1.57 (m, 3H), 1.57–1.49 (m, 2H), 1.20–1.08 (m, 2H), 0.95 (d, *J* = 6.8 Hz, 3H).

^{13}C NMR (126 MHz, CD_2Cl_2): δ = 203.5, 50.6, 46.7, 33.9, 31.2, 30.6, 25.8, 18.9.

HRMS *m/z* (ESI): calcd. for $C_9H_{17}O_1$ ($[M+H]^+$): 141.127390; found: 141.127310.

GC (30 m Ivadex-1, injection temperature: 220 °C, 40 °C, 1 °C/min, 80 °C iso 5 min, 8 °C/min, 220 °C iso 5 min, 0.5 bar H_2): t_{R1} = 49.1 min (major), t_{R2} = 49.5 min (minor), er = 98.5:1.5 (97% ee).

$[\alpha]_D^{25}$ = –17.7 (c= 0.23, $CHCl_3$)

(*R*)-3-cyclobutylbutanal (3o)

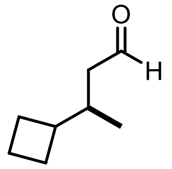

Following GP4 the reaction was performed using enal **1o** (27 µL, 0.20 mmol, 1.00 equiv). ^{1}H NMR yield was determined to be 84% using mesitylene as internal standard. Purification by silica gel flash chromatography (20–50% DCM in pentane) afforded the product **3o** (9.7 mg, 0.08 mmol, 38%) as a light yellow oil.

^{1}H NMR (501 MHz, CD_2Cl_2): δ = 9.72 (dd, *J* = 2.9, 2.0 Hz, 1H), 2.34 (ddd, *J* = 15.8, 4.5, 1.9 Hz, 1H), 2.10–2.02 (m, 2H), 2.02–1.87 (m, 3H), 1.85–1.76 (m, 1H), 1.75–1.60 (m, 3H), 0.85 (d, *J* = 6.6 Hz, 3H).

^{13}C NMR (126 MHz, CD_2Cl_2): δ = 203.3, 48.9, 42.5, 35.4, 27.4, 27.1, 17.7, 17.1.

HRMS *m/z* (ESI): calcd. for $C_8H_{14}Na_1O_1$ ($[M+Na]^+$): 149.09369; found: 149.09376.

GC (30 m Ivadex-1, injection temperature: 220 °C, 40 °C, 1 °C/min, 80 °C iso 5 min, 8 °C/min, 220 °C iso 5 min, 0.5 bar H_2): t_{R1} = 31.5 min (major), t_{R2} = 32.9 min (minor), er = 97:3 (94% ee).

$[\alpha]_D^{25}$ = –28.6 (c= 0.14, $CHCl_3$)

(*R*)-3,4,4-trimethylpentanal (3p)

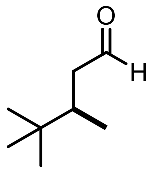

Following GP4 the reaction was performed using enal **1p** (29 µL, 0.20 mmol, 1.00 equiv). ^{1}H NMR yield was determined to be 96% using mesitylene as internal standard. Purification by silica gel flash chromatography (20–50% DCM in pentane) afforded the product **3p** (16.1 mg, 0.13 mmol, 63%) as a colorless oil.

^{1}H NMR (501 MHz, CD_2Cl_2): δ = 9.72 (dd, *J* = 3.3, 1.3 Hz, 1H), 2.57–2.50 (m, 1H), 2.06 (ddd, *J* = 16.0, 10.2, 3.3 Hz, 1H), 1.93–1.81 (m, 1H), 0.89–0.87 (m, 12H).

^{13}C NMR (126 MHz, CD_2Cl_2): δ = 203.7, 47.4, 37.8, 33.0, 27.3, 15.5.

HRMS *m/z* (GC-EI): calcd. for $C_8H_{16}O_1$ ($[M]^+$): 128.119565; found: 128.119770.

GC (30 m Ivadex-1, injection temperature: 220 °C, 40 °C, 1 °C/min, 80 °C iso 5 min, 8 °C/min, 220 °C iso 5 min, 0.5 bar H_2): t_{R1} = 21.9 min (major), t_{R2} = 24.0 min (minor), er = 99.5:0.5 (99% ee).

$[\alpha]_D^{25}$ = –30.1 (c= 0.07, $CHCl_3$)

(*R*)-3,4-dimethylpentanal (3q)

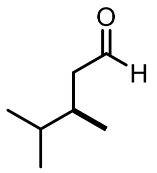

Following GP4 the reaction was performed using enal **1q** (26 µL, 0.20 mmol, 1.00 equiv). ^{1}H NMR yield was determined to be 84% using mesitylene as internal standard. Purification by silica gel flash chromatography (20–50% DCM in pentane) afforded the product **3q** (7.7 mg, 0.07 mmol, 34%) as a colorless oil.

^{1}H NMR (501 MHz, CD_2Cl_2): δ = 9.73 (dd, *J* = 2.9, 1.8 Hz, 1H), 2.41 (ddd, *J* = 16.0, 4.8, 1.9 Hz, 1H), 2.16 (ddd, *J* = 16.0, 9.0, 2.9 Hz, 1H), 2.01–1.90 (m, 1H), 1.64–1.55 (m, 1H), 0.89 (d, *J* = 6.8 Hz, 6H), 0.86 (d, *J* = 6.9 Hz, 3H).

^{13}C NMR (126 MHz, CD_2Cl_2): δ = 203.5, 48.8, 34.0, 32.7, 19.8, 18.7, 16.4.

HRMS *m/z* (GC-CI): calcd. for $C_7H_{15}O_1$ ($[M+H]^+$): 115.111740; found: 115.111850.

GC (30 m Ivadex-1, injection temperature: 220 °C, 40 °C, 1 °C/min, 80 °C iso 5 min, 8 °C/min, 220 °C iso 5 min, 0.5 bar H_2): t_{R1} = 14.9 min (major), t_{R2} = 16.1 min (minor), er = 97:3 (94% ee).

$[\alpha]_D^{25}$ = –9.2 (c= 0.06, $CHCl_3$)

(*S*)-3-methylpentanal (3r), (*S*)-1-(2,4-dinitrophenyl)-2-(3-methylpentylidene)hydrazine (3ra)

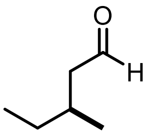

Following GP4 the reaction was performed using enal **1r** (22µL, 0.20 mmol, 1.00 equiv) in CyH/*n*-pentane (9:1) at –10 °C for 8 days. After addition of mesitylene as an internal standard ^1H NMR analysis of the crude reaction mixture showed 89% yield of product **3r**.

^{1}H NMR (501 MHz, CD_2Cl_2): δ 9.73 (t, *J* = 2.3 Hz, 1H), 2.38 (ddd, *J* = 16.1, 5.8, 2.1 Hz, 1H), 2.19 (ddd, *J* = 16.1, 7.9, 2.5 Hz, 1H), 2.01–1.91 (m, 1H), 1.42–1.31 (m, 1H), 1.31–1.21 (m, 1H), 0.94 (d, *J* = 6.8 Hz, 3H), 0.90 (t, *J* = 7.4 Hz, 3H).

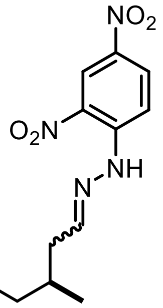

For *in situ* derivatization of the volatile aldehyde **3r**, 2,4-dinitrophenylhydrazine (43 mg, 0.22 mmol, 1.10 equiv), 1 mL EtOH and a drop of concentrated H_2SO_4 were added to the crude mixture. The reaction was stirred at room temperature for 1 h. Subsequent silica gel flash chromatography (0–10% EtOAc in hexanes) afforded an *E*/*Z* mixture of the corresponding hydrazone **3ra** (36.3 mg, 0.13 mmol, 66%, *E*/*Z*≈77:23) as an orange solid.

^{1}H NMR (501 MHz, $CDCl_3$): δ = 11.20 (s, $1H_{min}$), 11.03 (s, $1H_{maj}$), 9.13 (d, *J* = 2.6 Hz, $1H_{min}$), 9.12 (d, *J* = 2.6 Hz, $1H_{maj}$), 8.33 (dd, *J* = 9.6, 2.6 Hz, $1H_{min}$), 8.29 (dd, *J* = 9.6, 2.6 Hz, $1H_{maj}$), 7.96 (d, *J* = 9.5 Hz, $1H_{min}$), 7.93 (d, *J* = 9.6 Hz, $1H_{maj}$), 7.53 (t, *J* = 5.8 Hz, $1H_{maj}$), 7.00 (t, *J* = 5.6 Hz, $1H_{min}$), 2.48–2.32 (m, $1H_{maj}$, $1H_{min}$), 2.32–2.17 (m, $1H_{maj}$, $1H_{min}$), 1.87–1.69 (m, $1H_{maj}$, $1H_{min}$), 1.54–1.39 (m, $1H_{maj}$, $1H_{min}$), 1.38–1.23 (m, $1H_{maj}$, $1H_{min}$), 1.04 (d, *J* = 6.7 Hz, $3H_{min}$), 0.99 (d, *J* = 6.7 Hz, $3H_{maj}$), 0.98–0.92 (m, $3H_{maj}$, $3H_{min}$).

^{13}C NMR (126 MHz, $CDCl_3$): δ = 152.3, 150.8, 145.4, 145.3, 137.9, 130.2, 130.1, 128.9, 123.7, 123.5, 116.7, 116.6, 39.3, 34.4, 33.2, 33.0, 29.6, 29.5, 19.7, 19.4, 11.6, 11.5.

HRMS *m/z* (GC-EI): calcd. for $C_{12}H_{16}N_4O_4$ ($[M]^+$): 280.116605; found: 280.116700.

HPLC (OD-3, *n*-heptane/*i*-PrOH 95:5, 298 K, 340 nm): t_{R1} = 13.273 min (major), t_{R2} = 15.590 min (minor), er = 91:9 (82% ee).

$[\alpha]_D^{25}$ = 2.4 (c= 0.17, $CHCl_3$)

(*S*)-3-methylhexanal (3s), (*S*)-1-(2,4-dinitrophenyl)-2-(3-methylhexylidene)hydrazine (3sa)

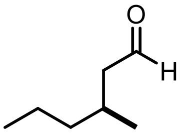

Following GP4 the reaction was performed using enal **1s** (26µL, 0.20 mmol, 1.00 equiv) in CyH at room temperature for 17 h. After addition of mesitylene as an internal standard ^1H NMR analysis of the crude reaction mixture showed 99% yield of product **3s**.

^{1}H NMR (501 MHz, CD_2Cl_2): δ = 9.72 (t, *J* = 2.3 Hz, 1H), 2.37 (ddd, *J* = 16.0, 5.7, 2.1 Hz, 1H), 2.19 (ddd, *J* = 16.0, 7.8, 2.6 Hz, 1H), 2.09–2.01 (m, 1H), 1.37–1.20 (m, 4H), 0.94 (d, *J* = 6.7 Hz, 3H), 0.90 (t, *J* = 7.1 Hz, 3H).

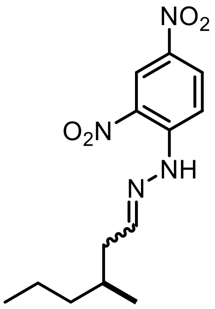

For *in situ* derivatization of the volatile aldehyde **3s**, 2,4-dinitrophenylhydrazine (44 mg, 0.22 mmol, 1.10 equiv), 1 mL EtOH and a drop of concentrated H_2SO_4 were added to the crude mixture. The reaction was stirred at room temperature for 1 h. Subsequent silica gel flash chromatography (0–10% EtOAc in hexanes) afforded an *E*/*Z* mixture of the corresponding hydrazone **3sa** (43.9 mg, 0.15 mmol, 73%, *E*/*Z*≈77:23) as an orange solid.

^{1}H NMR (501 MHz, $CDCl_3$): δ = 11.19 (s, $1H_{min}$), 11.02 (s, $1H_{maj}$), 9.12 (d, *J* = 2.6 Hz, $1H_{min}$), 9.10 (d, *J* = 2.6 Hz, $1H_{maj}$), 8.32 (dd, *J* = 9.6, 2.6 Hz, $1H_{min}$), 8.29 (dd, *J* = 9.6, 2.6 Hz, $1H_{maj}$), 7.95 (d, *J* = 9.6 Hz, $1H_{min}$), 7.93 (d, *J* = 9.7 Hz, $1H_{maj}$), 7.53 (t, *J* = 5.8 Hz, $1H_{maj}$), 6.99 (t, *J* = 5.6 Hz, $1H_{min}$), 2.42 (dt, *J* = 14.6, 5.7 Hz, $1H_{maj}$), 2.36 (dt, *J* = 16.1, 5.7 Hz, $1H_{min}$), 2.31–2.17 (m, $1H_{maj}$, $1H_{min}$), 1.94–1.88 (m, $1H_{min}$), 1.83 (hept, *J* = 6.8 Hz, $1H_{maj}$), 1.46–1.17 (m, $4H_{maj}$, $4H_{min}$), 1.04 (d, *J* = 6.7 Hz, $3H_{min}$), 0.99 (d, *J* = 6.7 Hz, $3H_{maj}$), 0.95–0.88 (m, $3H_{maj}$, $3H_{min}$).

^{13}C NMR (126 MHz, $CDCl_3$): δ = 152.3, 150.8, 145.4, 145.3, 138.4, 137.9, 130.2, 130.1, 129.6, 128.9, 123.6, 123.5, 116.7, 116.6, 39.7, 39.2, 39.1, 34.7, 31.3, 31.1, 20.2, 20.2, 20.0, 19.8, 14.3, 14.3.

HRMS *m*/*z* (GC-EI): calcd. for $C_{13}H_{18}N_4O_4$ ($[M]^+$): 294.132255; found: 294.132420.

HPLC (OD-3, *n*-heptane/*i*-PrOH 95:5, 298 K, 340 nm): t_{R1} = 12.716 min (major), t_{R2} = 15.413 min (minor), er = 97.5:2.5 (95% ee).

$[\alpha]_D^{25}$ = –1.4 (c= 0.14, $CHCl_3$)

(*S*)-3-methylheptanal (3t)

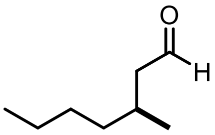

Following GP4 the reaction was performed using enal **1t** (29 µL, 0.20 mmol, 1.00 equiv). ^{1}H NMR yield was determined to be 84% using mesitylene as internal standard. Purification by silica gel flash chromatography (20–50% DCM in pentane) afforded the product **3t** (13.4 mg, 0.11 mmol, 52%) as a colorless oil.

^{1}H NMR (501 MHz, CD_2Cl_2): δ = 9.73 (t, *J* = 2.4 Hz, 1H), 2.37 (ddd, *J* = 16.1, 5.7, 2.1 Hz, 1H), 2.20 (ddd, *J* = 16.1, 7.9, 2.6 Hz, 1H), 2.07–1.98 (m, 1H), 1.32–1.23 (m, 6H), 0.94 (d, *J* = 6.7 Hz, 3H), 0.91–0.87 (m, 3H).

^{13}C NMR (126 MHz, CD_2Cl_2): δ = 203.3, 51.4, 37.0, 29.5, 28.5, 23.2, 20.1, 14.2.

HRMS *m*/*z* (ESI): calcd. for $C_8H_{16}Na_1O_1$ ($[M+Na]^+$): 151.10934; found: 151.10946.

GC (30 m Ivadex-1, injection temperature: 220 °C, 45 °C iso 40 min, 1 °C/min, 65 °C, 8 °C/min, 220 °C iso 5 min, 0.5 bar H_2): t_{R1} = 44.3 min (major), t_{R2} = 46.4 min (minor), er = 95:5 (90% ee).

$[\alpha]_D^{25}$ = –17.4 (c= 0.23, $CHCl_3$)

Following GP4 the reaction was performed using enal **1t** (29 µL, 0.20 mmol, 1.00 equiv) in CyH at room temperature. ^{1}H NMR yield was determined to be 84% using mesitylene as internal standard. Purification by silica gel flash chromatography (20–50% DCM in pentane) afforded the product **3t** (7.1 mg, 0.06 mmol, 28%) as a colorless oil.

GC (25 m Hydrodex-beta-TBDAc-CD, injection temperature: 220 °C, 70 °C iso 44 min, 8 °C/min, 220 °C, 0.5 bar H_2): t_{R1} = 37.5 min (major), t_{R2} = 40.6 min (minor), er = 97.5:2.5 (95% ee).

$[\alpha]_D^{25}$ = –23.5 (c= 0.03, $CHCl_3$)

(*S*)-3,7-dimethyloct-6-enal/ (*S*)-citronellal (3u)

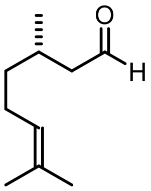

Following GP4 the reaction was performed using citral (34 µL, 0.20 mmol, 1.00 equiv) in CyH at room temperature. ^{1}H NMR yield was determined to be 84% using mesitylene as internal standard. Purification by silica gel flash chromatography (20–50% DCM in pentane) afforded the product **3u** (27.3 mg, 0.18 mmol, 89%) as a light yellow oil.

^{1}H NMR (501 MHz, CD_2Cl_2): δ = 9.72 (t, *J* = 2.3 Hz, 1H), 5.13–5.07 (m, 1H), 2.38 (ddd, *J* = 16.1, 5.6, 2.0 Hz, 1H), 2.20 (ddd, *J* = 16.1, 8.0, 2.6 Hz, 1H), 2.10–1.92 (m, 3H), 1.68 (d, *J* = 1.4 Hz, 3H), 1.60 (d, *J* = 1.3 Hz, 3H), 1.41–1.30 (m, 1H), 1.30–1.22 (m, 1H), 0.95 (d, *J* = 6.7 Hz, 3H).

^{13}C NMR (126 MHz, CD_2Cl_2): δ = 203.2, 132.0, 124.5, 51.4, 37.3, 28.2, 25.8, 20.0, 17.7.

HRMS *m/z* (ESI): calcd. for $C_{10}H_{19}O_1$ ($[M+H]^+$): 155.143040; found: 155.143110.

GC (30 m BGB-174, injection temperature: 220 °C, 70 °C iso 120 min, 8 °C/min, 240 °C iso 3 min, 0.6 bar H_2): t_{R1} = 95.0 min (major), t_{R2} = 102.3 min (minor), er = 97:3 (94% ee).

$[\alpha]_D^{25}$ = –19.1 (c= 0.34, $CHCl_3$)

(*S*,*E*)-3,7,11-trimethyldodeca-6,10-dienal/ (*S*,*E*)-dihydrofarnesal (3v)

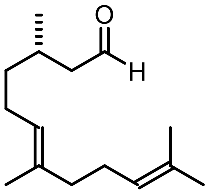

Following GP4 the reaction was performed using farnesal (96%, 50 µL, 0.20 mmol, 1.00 equiv) in CyH at room temperature for 7 days. Purification by silica gel flash chromatography (20–50% DCM in pentane) afforded the product **3v** (35.4 mg, 0.16 mmol, 81%) as a colorless oil.

^{1}H NMR (501 MHz, CD_2Cl_2): δ = 9.72 (t, *J* = 2.2 Hz, 1H), 5.14–5.07 (m, 2H), 2.39 (ddd, *J* = 16.0, 5.6, 2.1 Hz, 1H), 2.20 (ddd, *J* = 16.0, 8.0, 2.6 Hz, 1H), 2.11–1.93 (m, 8H), 1.67 (d, *J* = 1.4 Hz, 3H), 1.60 (d, *J* = 1.3 Hz, 6H), 1.41–1.23 (m, 3H), 0.96 (d, *J* = 6.7 Hz, 3H).

^{13}C NMR (126 MHz, CD_2Cl_2): δ = 203.2, 135.7, 131.7, 124.7, 124.5, 51.4, 40.1, 37.3, 28.1, 27.1, 25.8, 25.7, 20.0, 17.8, 16.1.

HRMS *m/z* (ESI): calcd. for $C_{15}H_{26}O_1$ ($[M]^+$): 222.197815; found: 222.197800.

GC (30 m BGB-174, injection temperature: 220 °C, 110 °C iso 160 min, 8 °C/min, 220 °C iso 3 min, 0.6 bar H_2): t_{R1} = 123.8 min (major), t_{R2} = 127.2 min (minor), er = 97.5:2.5 (95% ee).

$[\alpha]_D^{25}$ = –14.3 (c= 0.27, $CHCl_3$)

5 mmol scale (3v)

Following GP4 the reaction was performed using farnesal (96%, 1.15 g, 5.00 mmol, 1.00 equiv) in CyH at room temperature for 19 h. Cyclohexane was removed under reduced pressure prior to purification by silica gel flash chromatography (20–50% DCM in pentane). Product **3v** (1.03 g, 4.63 mmol, 93%) was obtained as a colorless oil.

GC (30 m BGB-174, injection temperature: 220 °C, 110 °C iso 160 min, 8 °C/min, 220 °C iso 3 min, 0.6 bar H_2): t_{R1} = 123.6 min (major), t_{R2} = 127.1 min (minor), er = 97:3 (94% ee).

$[\alpha]_D^{25}$ = –14.7 (c= 0.37, $CHCl_3$)

6. Catalyst Characterization (6e)

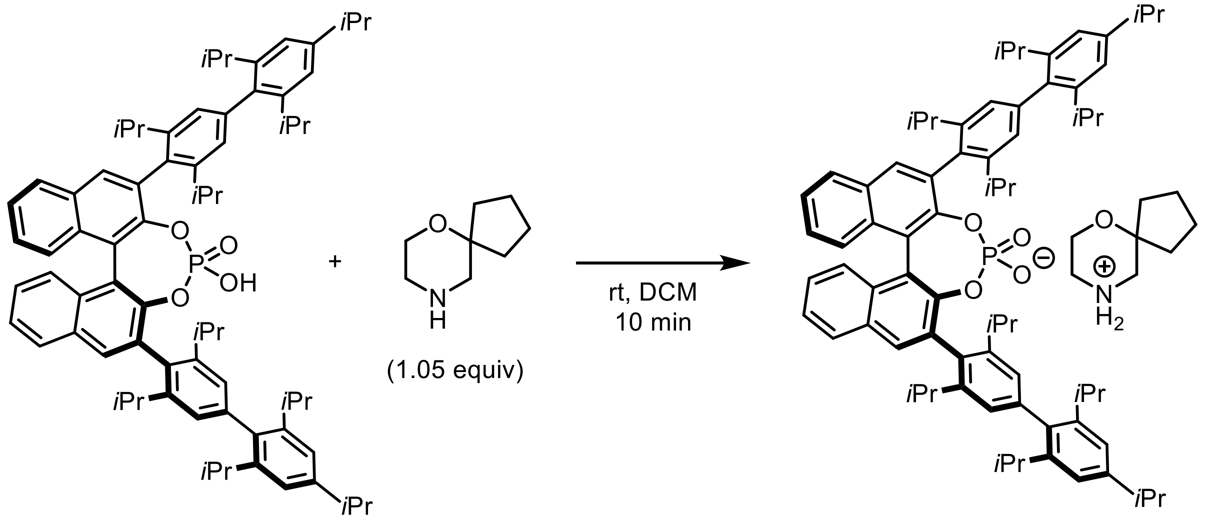

An oven-dried vial, equipped with a magnetic stirring bar, was charged with chiral phosphoric acid **6** (790 mg, 0.74 mmol, 1.00 equiv) and amine **e** (109 mg, 0.77 mmol, 1.05 equiv). After addition of DCM (2 mL), the reaction mixture was stirred for ten minutes at room temperature. The solvent was removed under reduced pressure. The remaining salt was washed four times with *n*-pentane and then dried under high vacuum overnight to afford **6e** as an off-white solid (881 mg, 0.73 mmol, 99%).

^{1}H NMR (501 MHz, CD_2Cl_2): δ = 7.94 (d, *J* = 8.2 Hz, 2H), 7.84 (s, 2H), 7.46 (ddd, *J* = 8.0, 6.3, 1.4 Hz, 2H), 7.36–7.28 (m, 4H), 7.15–7.09 (m, 6H), 7.03 (d, *J* = 1.7 Hz, 2H), 3.69–3.44 (m, 2H), 3.13–3.03 (m, 2H), 3.01–2.94 (m, 2H), 2.93–2.86 (m, 2H), 2.86–2.77 (m, 3H), 2.76–2.61 (m, 5H), 1.80–1.61 (m, 4H), 1.55–1.42 (m, 4H), 1.34 (d, *J* = 6.9 Hz, 12H), 1.25–1.12 (m, 42H), 0.92 (d, *J* = 6.8 Hz, 6H).

^{31}P NMR (203 MHz, $CDCl_3$): δ = 6.1.

^{13}C NMR (126 MHz, CD_2Cl_2): δ = 148.2, 147.8, 147.4, 147.3, 146.2, 140.0, 138.1, 133.0, 132.8, 132.5, 130.7, 128.2, 127.5, 126.0, 125.0, 123.8, 123.0, 121.0, 120.7, 81.3, 58.9, 49.7, 42.5, 35.7, 34.4, 31.0, 30.8, 30.6, 30.4, 26.3, 25.1, 24.9, 24.6, 24.6, 24.5, 24.3, 24.3, 24.0, 23.9, 23.8, 23.7.

HRMS *m*/*z* (ESI): calcd. for $C_8H_{16}N_1O_1$ ([M– $C_{74}H_{88}O_1P_1$]$^+$): 142.12264; found: 142.12263.

HRMS *m*/*z* (ESI): calcd. for $C_{74}H_{88}O_1P_1$ ([M– $C_8H_{16}N_1O_1$]$^-$): 1071.64257; found: 1071.64271.

7. Mechanistic Experiments

a. Enantioconvergence Experiment

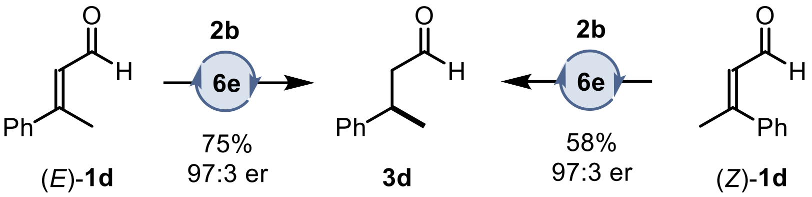

An oven-dried GC vial under argon, equipped with a magnetic stirring bar, was charged with the chiral catalyst **6e** (2.5 mol%). The respective (*E*)- or (*Z*)-isomer of **1d** (0.03 mmol) was added and dissolved in dry 1,4-dioxane (0.1 M). The reaction mixture was stirred for five minutes at room temperature. After addition of Hantzsch ester **2b** (1.1 equiv) the reaction mixture was heated to 50 °C and stirred for 24 h. The reaction was quenched by addition of NEt_3 (10 µL) followed by addition of Ph_3CH (1.0 M in PhMe) as internal standard. An aliquot of the mixture was taken and diluted with $CDCl_3$ for subsequent ^1H NMR analysis. The remaining solution was used for preparative thin layer chromatography to purify the chiral product **3d**. Chiral GC analysis was performed to give the corresponding enantiomeric ratio.

Regardless of which geometric isomer was used as the starting material, the same enantiomeric ratio of the product (97:3 er) was obtained.

b. Deuterium Scrambling Experiment

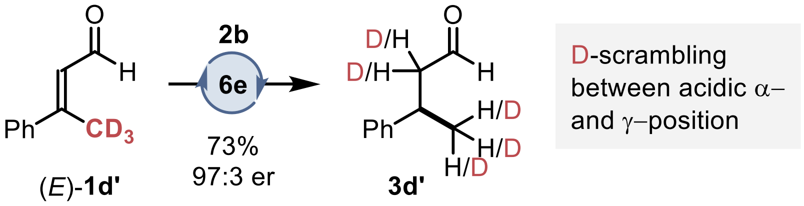

An oven-dried GC vial under argon, equipped with a magnetic stirring bar, was charged with the chiral catalyst **6e** (2.5 mol%). The (*E*)-isomer of the deuterated substrate **1d'** (0.03 mmol) was added and dissolved in dry 1,4-dioxane (0.1 M). The reaction mixture was stirred for five minutes at room temperature. After addition of Hantzsch ester **2b** (1.1 equiv) the reaction mixture was heated to 50 °C and stirred for 24 h. The reaction was quenched by addition of NEt_3 (10 µL) followed by addition of Ph_3CH (1.0 M in PhMe) as internal standard. An aliquot of the mixture was taken and diluted with $CDCl_3$ for subsequent ^1H NMR analysis. The remaining solution was used for preparative thin layer chromatography to purify the chiral product **3d'**. Chiral GC analysis was performed to give the corresponding enantiomeric ratio.

Mass spectrometry as well as ^1H and ^2H NMR analyses (in CD_2Cl_2) were performed. Based on the C**H**O NMR signal, **3d'** was found to be a mixture of isotopomers with the following average D distribution:

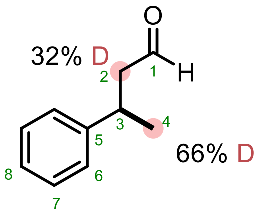

1H{off},1D, 600.20 MHz,CD2Cl2,298.0K, pulse sequence: zg30

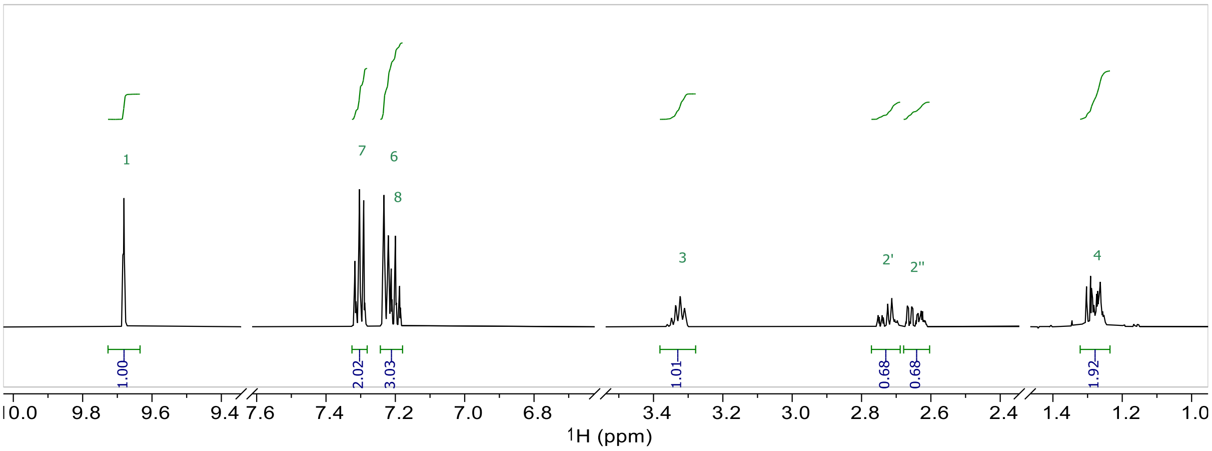

2H{off},1D, 92.13 MHz,CD2Cl2,298.0K, pulse sequence: zg2h.2

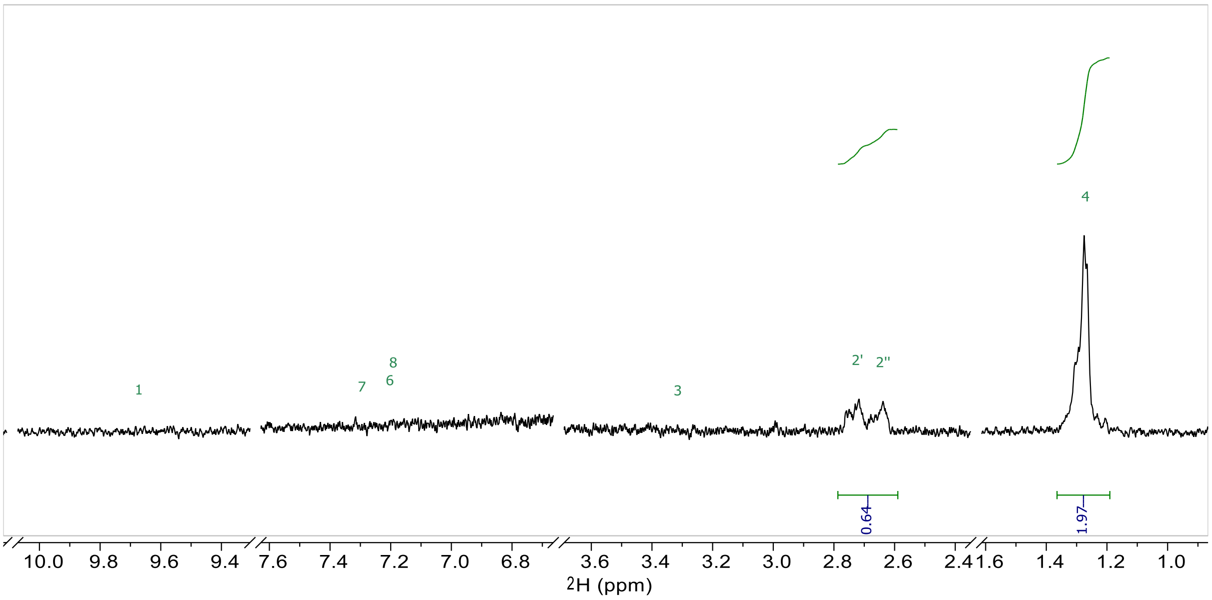

GC-EI-MS analysis revealed the presence of D0 to D5 species with the following D distribution:

D0	4.5% ± 0.6%
D1	13.9% ± 0.8%
D2	23.5% ± 0.8%
D3	31.2% ± 0.8%
D4	21.6% ± 0.8%
D5	5.0% ± 0.6%
	**99.7%**

c. Transfer Hydrogenation of 4,4,4-trifluoro-3-phenylbut-2-enal

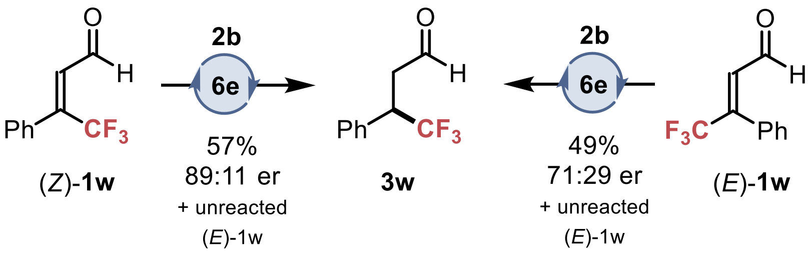

An oven-dried GC vial under argon, equipped with a magnetic stirring bar, was charged with the chiral catalyst **6e** (2.5 mol%). The respective (*E*)- or (*Z*)-isomer of **S4** (0.03 mmol) was added and dissolved in dry 1,4-dioxane (0.1 M). The reaction mixture was stirred for five minutes at room temperature. After addition of Hantzsch ester **2b** (1.1 equiv) the reaction mixture was heated to 50 °C and stirred for 24 h. The reaction was quenched by addition of NEt_3 (10 µL) followed by addition of Ph_3CH (1.0 M in PhMe) as internal standard. An aliquot of the mixture was taken and diluted with $CDCl_3$ for subsequent ^1H NMR analysis. The remaining solution was used for preparative thin layer chromatography to purify the chiral product **S5**. Chiral GC analysis was performed to give the corresponding enantiomeric ratio.

Regardless of which isomer was reacted, the same product enantiomer is favored and exclusively the *E*-isomer remains unreacted. However, different enantiomeric ratios are obtained depending on the geometrical purity of the starting material. These observations indicate a different and slower isomerization pathway taking place.

d. Transfer Hydrogenation of β-CH_2R Cinnamaldehydes

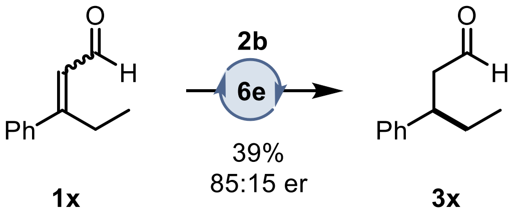

An oven-dried GC vial under argon, equipped with a magnetic stirring bar, was charged with the chiral catalyst **6e** (2.5 mol%). Substrate **1x** (0.03 mmol) was added and dissolved in dry 1,4-dioxane (0.1 M). The reaction mixture was stirred for five minutes at room temperature. After addition of Hantzsch ester **2b** (1.1 equiv) the reaction mixture was heated to 50 °C and stirred for 24 h. The reaction was quenched by addition of NEt_3 (10 µL) followed by addition of Ph_3CH (1.0 M in PhMe) as internal standard. An aliquot of the mixture was taken and diluted with $CDCl_3$ for subsequent ^1H NMR analysis. The remaining solution was used for preparative thin layer chromatography to purify the chiral product **3x**. Chiral GC analysis was performed to give the corresponding enantiomeric ratio.

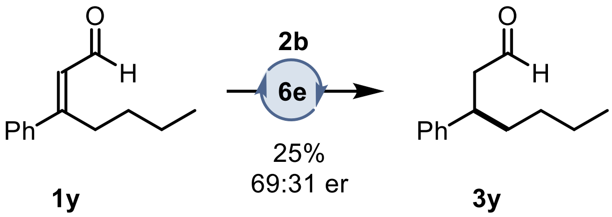

An oven-dried GC vial under argon, equipped with a magnetic stirring bar, was charged with the chiral catalyst **6e** (2.5 mol%). Substrate **1y** (0.03 mmol) was added and dissolved in dry 1,4-dioxane (0.1 M). The reaction mixture was stirred for five minutes at room temperature. After addition of Hantzsch ester **2b** (1.1 equiv) the reaction mixture was heated to 50 °C and stirred for 24 h. The reaction was quenched

by addition of NEt_3 (10 µL) followed by addition of Ph_3CH (1.0 M in PhMe) as internal standard. An aliquot of the mixture was taken and diluted with $CDCl_3$ for subsequent ^{1}H NMR analysis. The remaining solution was used for preparative thin layer chromatography to purify the chiral product **3y**. Chiral GC analysis was performed to give the corresponding enantiomeric ratio.

The enantioselectivity decreases with longer β-alkyl chains, possibly due to a weakened CH···O interaction between the β-CH_2R group and the phosphate (see transition state analysis in Section 8). This could be due to the reduced polarity of the CH bond and potential steric clashes introduced by the bulkier substituents. Current optimization studies of related compounds are ongoing in our laboratory.

e. Reaction Mechanism

In agreement with our above mentioned experiments and previous mechanistic proposals[20,21] we suggest the following mechanism:

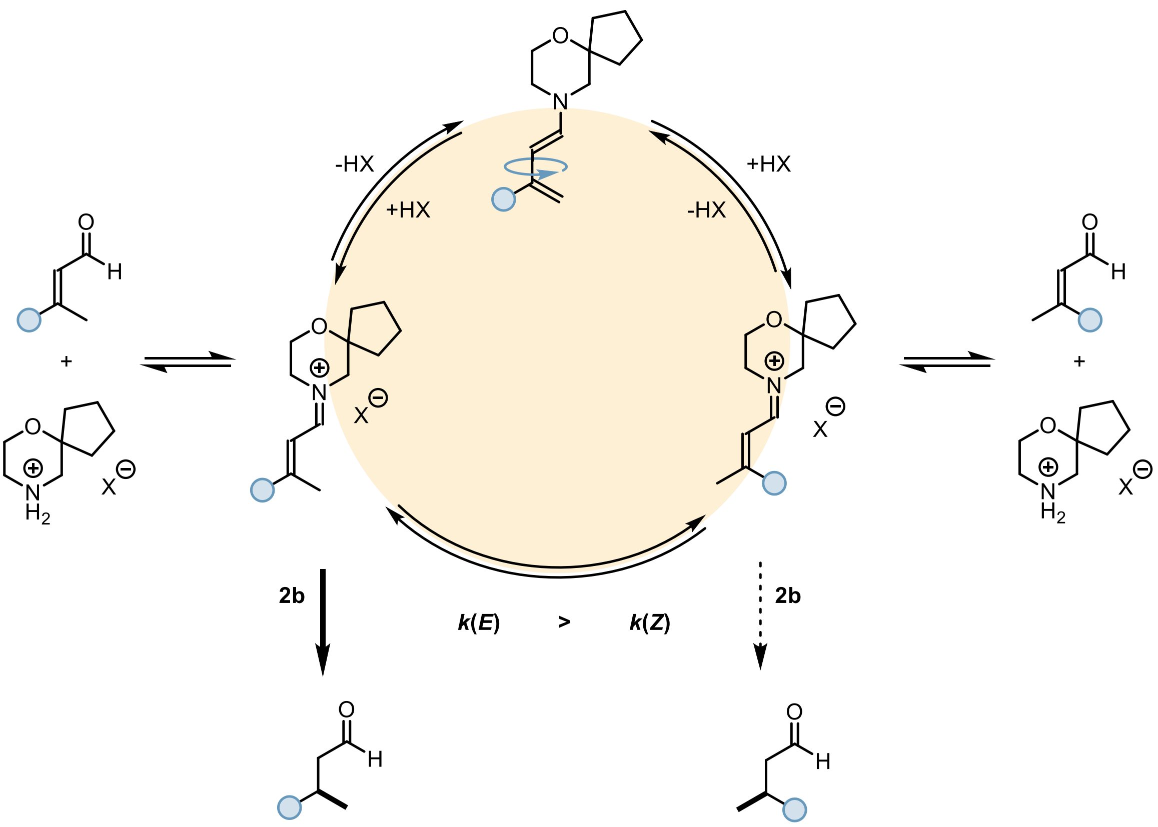

8. Computational Details

Preliminary transition state structures were generated at the GFN2-xTB[22] level using the xtb program version 6.6.0, followed by exploration of the conformational landscape at the same level of theory using Grimme's Conformer–Rotamer Ensemble Sampling Tool (CREST)[23], version 2.12 with the `mdlen = 50` setting. All resulting conformers were subsequently optimized using constraints at the GFN2-xTB level. Following structure reduction through RMSD evaluation, conformers that met the elimination criteria were subsequently subjected to constrained optimizations using DFT.

All DFT calculations were conducted using ORCA version 5.0.3.[24] The GFN2-xTB conformers were optimized using the PBE exchange-correlation functional[25] and Grimme's DFT-D3[26] scheme with Becke–Johnson damping[27] along with the def2-SVP[28] basis set (verytightscf convergence criterion). This computational protocol for geometry optimization has proven successful in the literature.(ref) The Resolution of Identity (RI) approximation[29] in the Split-RI-J[30] variant using a corresponding auxiliary basis set[31] was used. After successful constrained geometry optimizations, another round of RMSD elimination was performed (see below for exact criteria), followed by transition state optimizations (OptTS keyword). True transition state structures were verified by subsequent vibrational frequency calculation at the same level of theory. Solvent effect were included implicitly with the CPCM model[32] in 1,4-dioxane (`epsilon 2.25` and `refrac 1.42` options in the `%cpcm` block). Gibbs free energies were calculated using refined electronic energies at various levels of theory in combination with thermochemical corrections from the vibrational frequency calculations acquired by using Duarte's `otherm.py`[33] in a 1 M solution standard state at 323 K.

Non-covalent interactions were studied using the Independent Gradient Model based on Hirshfeld partition (IGMH)[34] in conjunction with the Multiwfn program, version 3.8.[35,36]

The following input was used for the DLPNO-CCSD(T) calculations:

```
! DLPNO-CCSD(T) cc-pVTZ cc-pVTZ/C cc-pVTZ/JK verytightscf TightPNO keepdens
```

a. 2006 System: Transition State Overview

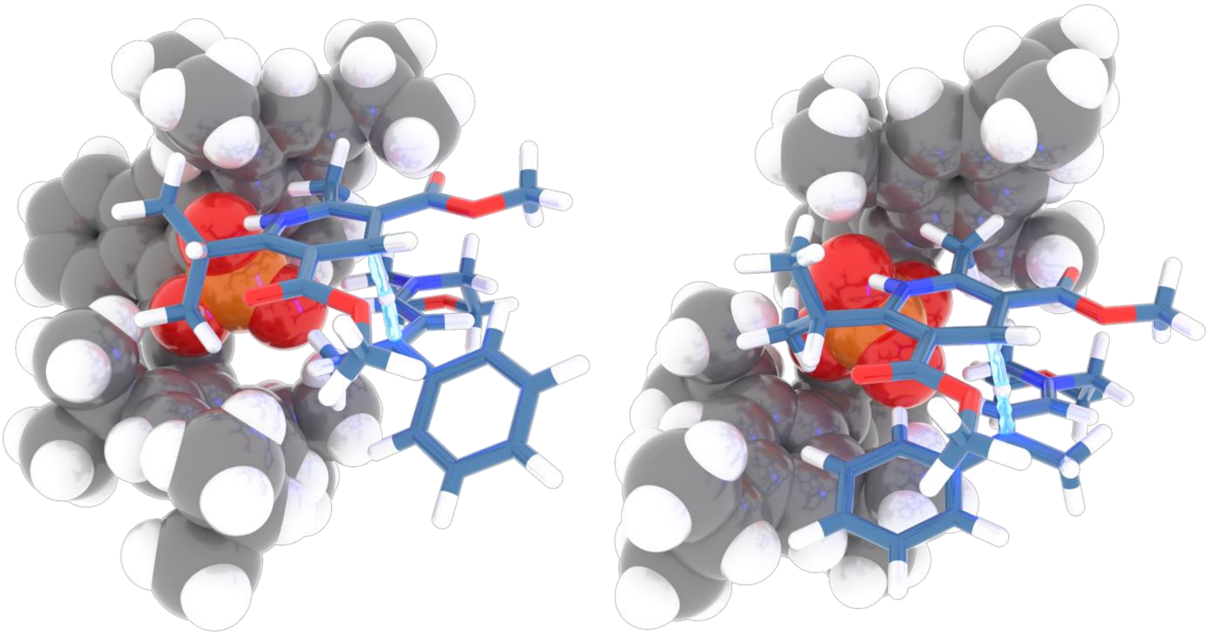

Figure S1: *Structural overview of* ***TS-1*** *(left) and* ***TS-2*** *(right).*

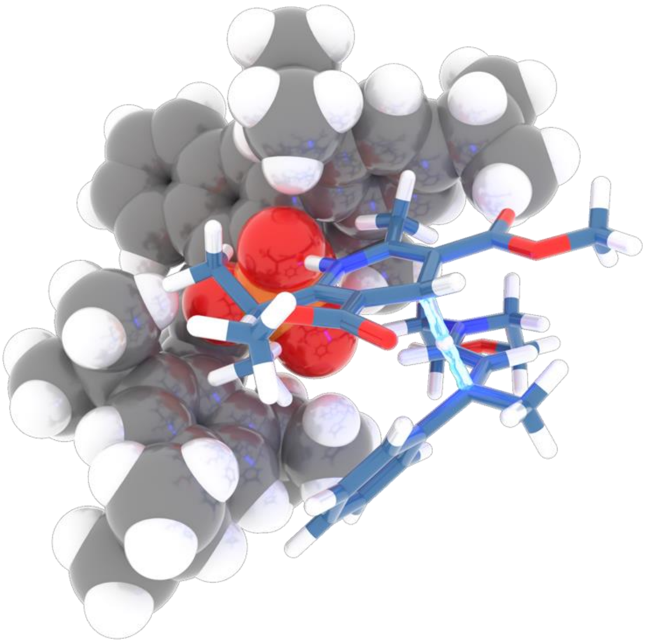

Figure S2: *Structural overview of* ***TS-2b****.*

b. Method Screening

In order to gain access to a computationally verifiable data point, we conducted the transfer hydrogenation of enal **1d** according to the following procedure:

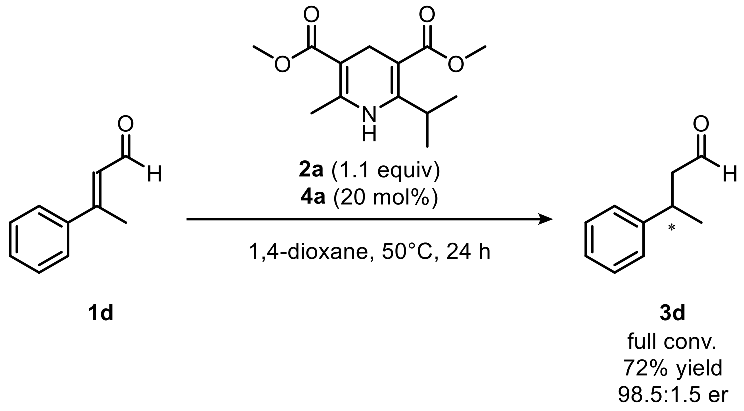

An oven dried GC vial under argon, equipped with a magnetic stirring bar, was charged with the chiral phosphoric acid (*S*)-**4** and morpholine **a** (20 mol%). Substrate **1d** (0.025 mmol) was added and dissolved in dry 1,4-dioxane (0.1 M). The reaction mixture was stirred for five minutes at room temperature. After addition of Hantzsch ester **2a** (1.1 equiv.) the reaction mixture was heated to 50 °C and stirred overnight. The reaction was terminated by addition of NEt_3 (10 µL) followed by addition of mesitylene as internal standard. An aliquot of the mixture was taken and diluted with $CDCl_3$ for subsequent ^{1}H NMR analysis. The remaining solution was used for preparative thin layer chromatography to purify the chiral product **3d**. Chiral GC analysis was performed to give the corresponding enantiomeric ratio (98.5:1.5).

According to the following equation, this enantiomeric ratio at 323 K corresponds to an energy difference of 2.7 kcal/mol.

$$ee = \frac{e^{\frac{-\Delta\Delta G^{\neq}}{RT}} - 1}{e^{\frac{-\Delta\Delta G^{\neq}}{RT}} + 1}$$

Subsequently, the three transition states **TS-1**, **TS-2**, and **TS-2b** were evaluated by single point calculation using distinct functionals, and eventually the corresponding Gibbs energies were compared by comparing with the thermochemical corrections obtained at the PBE-D3(BJ)/def2-SVP level of theory.

The same protocol was followed for catalyst **6e** (97:3 er in the reaction to form **3d**), corresponding to an energy difference of 2.2 kcal/mol.

Table S3: *Gibbs energies (in kcal/mol) of the transition states (double bond configurations below) as calculated by combining single point energies of different functionals and basis sets with the PBE-D3(BJ)/def2-SVP thermochemical corrections; solvent effects taken into account by using the CPCM model (epsilon = 2.25, refrac = 1.42).*

	**B3LYP-D3(BJ)/def2-TZVPP**[37,38]	**B3LYP-D3(BJ)/def2-TZVPPD**[37,38]	**B3LYP/def2-TZVPP**[37,38]	**M06-2X/def2-TZVPP**[39]	**B2PLYP-D3(BJ)/def2-TZVPP**[40]	**ωB97X-V/def2-TZVPP**[41]	**ωB97M-D3(BJ)/def2-TZVPP**[42]	**ωB97M-V/def2-TZVPP**[42]	**DLPNO-CCSD(T)/cc-pVTZ**[43–50]
**TS-1** (*E*)	0.0	0.0	0.0	0.0	0.0	0.0	0.0	0.0	**0.0**
**TS-2** (*E*)	2.7	3.3	8.7	2.1	2.2	3.9	3.2	2.7	**2.6**
**TS-2b** (*Z*)	3.8	4.0	2.1	4.2	4.0	5.5	5.2	5.6	**4.6**
**TS-3** (*E*,*E*)	0.0	–	0.0	0.0	–	0.0	0.0	0.0	**0.0**
**TS-4** (*E*,*Z*)	2.5	–	9.0	2.7	–	3.9	2.8	3.0	**2.2**
**TS-4b** (*Z*,*E*)	1.2	–	8.8	3.5	–	3.2	2.6	2.5	**2.5**
**TS-3b** (*Z*,*E*)	4.1	–	–5.5	3.3	0.0	4.5	4.1	4.7	**3.7**
**TS-4c** (*Z*,*Z*)	6.5	–	5.7	4.6	1.9	8.1	6.6	7.2	**5.8**
**TS-4d** (*E*,*E*)	6.0	–	5.4	4.4	1.3	8.3	6.6	7.2	**6.1**

In light of the good agreement between the experimental values and computational benchmark, the DLPNO-CCSD(T) method in conjunction with a cc-pVTZ basis set was used for all further calculations reported in this study, unless stated otherwise.

c. 2006 System: Distortion–Interaction Analysis

Table S4: *Overview of the transition states at the CPCM(1,4-dioxane)-DLPNO-CCSD(T)/cc-pVTZ//PBE-D3(BJ)def2-SVP level of theory as well as computed enantiomeric ratios at 323 K.*

	**$\Delta\Delta G^{\ddagger}$ (323 K)**	**Computed er (323 K)**
**TS-1**	0.0 kcal/mol	–
**TS-2**	2.6 kcal/mol	98.5:1.5
**TS-2b**	4.6 kcal/mol	>99.5:0.5

In addition to the Gibbs free energy comparison of the lowest-lying transition states, Distortion–Interaction Analysis of the final single point energies was conducted[51]:

***Table S5:** Distortion–Interaction analysis between **TS-1**, **TS-2**, and **TS-2b** at the DLPNO-CCSD(T)/cc-pVTZ//PBE-D3(BJ)def2-SVP level of theory. Positive values: favored in **TS-1**, negative values: favored in **TS-2** or **TS-2b**.*

**Component**	**Δ*E*(SP), TS-2 – TS-1**	**Δ*E*(SP), TS-2b – TS-1**
total	1.7 kcal/mol	4.4 kcal/mol
IM + H	–1.5 kcal/mol	–0.8 kcal/mol
P	1.1 kcal/mol	–1.1 kcal/mol
distortion	–0.4 kcal/mol	–1.8 kcal/mol
interaction	2.1 kcal/mol	6.2 kcal/mol

Table S5 shows that the substrates in **TS-1** undergo a significant amount of distortion, most likely due to maximized electrostatic interactions of the iminium to the phosphate fragment. However, the phosphate in **TS-2** experiences a higher degree of distortion, which might be related to the increased dispersion interactions. For further discussion, please see table S33 and the LED calculations (Tables S9 and S10). The comparison between **TS-1** and **TS-2b** highlights the dominance of interaction as a stereodetermining element. Not only are electrostatic interactions due to an unfavorable orientation of the iminium in **TS-2b** expected to contribute to this observation, but further dispersion interactions between the fragments as well.

d. 2006 System: Local Energy Decomposition

In order to calculate the interaction between the three fragments within the system, we chose to perform a Local Energy Decomposition analysis at the DLPNO-CCSD(T)/cc-pVTZ level of theory for **TS-1**, **TS-2** and **TS-2b**.[43–50]

The resulting terms, corresponding to the decomposition of the HF interaction energy as well as the correlation interaction energy, can be found below. The sum of the individual elements is given at the top left corner of each matrix. The overall non-dispersion fragment interaction was calculated by adding the non-dispersion correlation contribution to the sum of electrostatic E_{elstat} and exchange E_{exch} interactions. All energies are given in kcal/mol.

***Table S6:** Results of the LED analysis of **TS-1** at the DLPNO-CCSD(T)/cc-pVTZ level of theory.*

Electronic Preparation and Binding Energy

–149.9	H	IM	P
H	584.6	–793.2	–204.8
IM	–	307.7	–163.1
P	–	–	118.9

Overall Non-Dispersion

–75.2	H	IM	P
H	565.5	–714.3	–169.7
IM	–	272.7	–140.6
P	–	–	111.1

Electrostatics

82.6	H	IM	P
H	565.5	–594.5	–144.3
IM	–	272.7	–127.9
P	–	–	111.1

Exchange

–157.8	H	IM	P
H	–	–119.8	–25.4
IM	–	–	–12.7
P	–	–	–

Dispersion

–78.5	H	IM	P
H	–	–35.1	–25.4
IM	–	–	–18.0
P	–	–	–

The non-dispersion correlation contribution was found to be 3.6 kcal/mol, resulting in a total $\Delta E_{\text{non-disp}}$ of –71.7 kcal/mol.

Table S7: *Results of the LED analysis of* ***TS-2*** *at the DLPNO-CCSD(T)/cc-pVTZ level of theory.*

Electronic Preparation and Binding Energy

–149.0	H	IM	P
H	590.1	–824.0	–177.3
IM	–	327.4	–177.4
P	–	–	112.1

Overall Non-Dispersion

–66.6	H	IM	P
H	571.2	–741.6	–144.4
IM	–	292.0	–148.0
P	–	–	104.3

Electrostatics

95.4	H	IM	P
H	571.2	–617.8	–122.4
IM	–	292.0	–131.9
P	–	–	104.3

Exchange

–162.0	H	IM	P
H	–	–123.8	–22.1
IM	–	–	–16.1
P	–	–	–

Dispersion

–85.1	H	IM	P
H	–	–37.4	–24.2
IM	–	–	–23.5
P	–	–	–

The non-dispersion correlation contribution was found to be 2.3 kcal/mol, resulting in a total $\Delta E_{\text{non-disp}}$ of –64.3 kcal/mol.

Table S8: *Results of the LED analysis of* ***TS-2b*** *at the DLPNO-CCSD(T)/cc-pVTZ level of theory.*

Electronic Preparation and Binding Energy

–142.2	H	IM	P
H	510.4	–678.4	–217.0
IM	–	261.2	–132.3
P	–	–	113.9

Overall Non-Dispersion

–69.1	H	IM	P
H	492.0	–602.4	–182.5
IM	–	229.9	–112.7
P	–	–	106.6

Electrostatics

79.0	H	IM	P
H	492.0	–490.6	–156.6
IM	–	229.9	–102.3
P	–	–	106.6

Exchange

–148.2	H	IM	P
H	–	–111.9	–25.9
IM	–	–	–10.4
P	–	–	–

Dispersion

–74.9	H	IM	P
H	–	–34.1	–24.7
IM	–	–	–16.2
P	–	–	–

The non-dispersion correlation contribution was found to be 2.1 kcal/mol, resulting in a total $\Delta E_{\text{non-disp}}$ of –67.0 kcal/mol.

Ultimately, we used the individual LED analyses of **TS-1**, **TS-2**, and **TS-2b** to calculate their corresponding difference.

Table S9: *Difference of LED analyses between* ***TS-1*** *and* ***TS-2*** *at the DLPNO-CCSD(T)/cc-pVTZ level of theory. Positive values: favored in* ***TS-1****, negative values: favored in* ***TS-2****.*

Electronic Preparation and Binding Energy

0.8	**H**	**IM**	**P**
**H**	5.5	–30.8	27.5
**IM**	–	19.7	–14.3
**P**	–	–	–6.8

Overall Non-Dispersion

8.6	**H**	**IM**	**P**
**H**	5.7	–27.3	25.2
**IM**	–	19.3	–7.4
**P**	–	–	–6.8

Electrostatics

12.8	**H**	**IM**	**P**
**H**	5.7	–23.3	21.9
**IM**	–	19.3	–4.0
**P**	–	–	–6.8

Exchange

–4.2	**H**	**IM**	**P**
**H**	–	–4.1	3.3
**IM**	–	–	–3.4
**P**	–	–	–

Dispersion

–6.6	**H**	**IM**	**P**
**H**	–	–2.3	1.1
**IM**	–	–	–5.5
**P**	–	–	–

Taking into account the individual non-dispersion correlation contributions, an overall difference in $\Delta E_{\text{non-disp}}$ between both transition states of 7.4 kcal/mol was obtained.

Table S10: *Difference of LED analyses between* ***TS-1*** *and* ***TS-2b*** *at the DLPNO-CCSD(T)/cc-pVTZ level of theory. Positive values: favored in* ***TS-1****, negative values: favored in* ***TS-2b****.*

Electronic Preparation and Binding Energy

7.6	**H**	**IM**	**P**
**H**	–74.2	114.8	–12.2
**IM**	–	–46.6	30.8
**P**	–	–	–5.0

Overall Non-Dispersion

6.1	**H**	**IM**	**P**
**H**	–73.5	111.8	–12.9
**IM**	–	–42.8	28.0
**P**	–	–	–4.5

Electrostatics

–3.6	**H**	**IM**	**P**
**H**	–73.5	104.0	–12.3
**IM**	–	–42.8	25.6
**P**	–	–	–4.5

Exchange

9.7	**H**	**IM**	**P**
**H**	–	7.9	–0.6
**IM**	–	–	2.4
**P**	–	–	–

Dispersion

3.5	**H**	**IM**	**P**
**H**	–	1.1	0.7
**IM**	–	–	1.8
**P**	–	–	–

Taking into account the individual non-dispersion correlation contributions, an overall difference in $\Delta E_{\text{non-disp}}$ between both transition states of 4.7 kcal/mol was obtained.

e. 2006 System: IGMH Analysis

An analysis according to the Idependent Gradient Model based on Hirshfeld partition[34] was conducted with the Multiwfn program, version 3.8, using the B3LYP-D3(BJ)/def2-TZVPP densities.(ref) Non-covalent interactions were subsequently plotted as an isosurface of δg^{inter} with an isovalue of 0.004. In a direct comparison, the atoms were colored by their respective δG^{atom}(%).

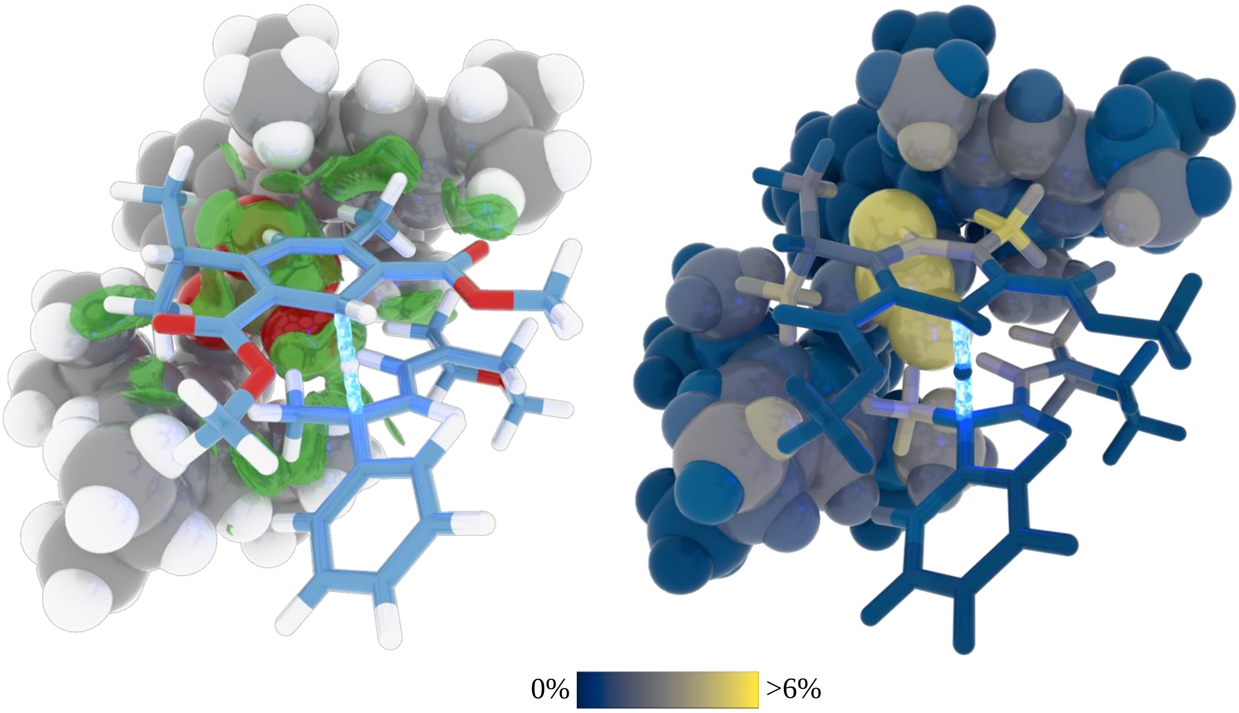

Figure S3: *δg^{inter} isosurface (left) and color-coded atoms according to their contribution to the overall interfragment interaction δG^{atom}(%) for **TS-1**.*

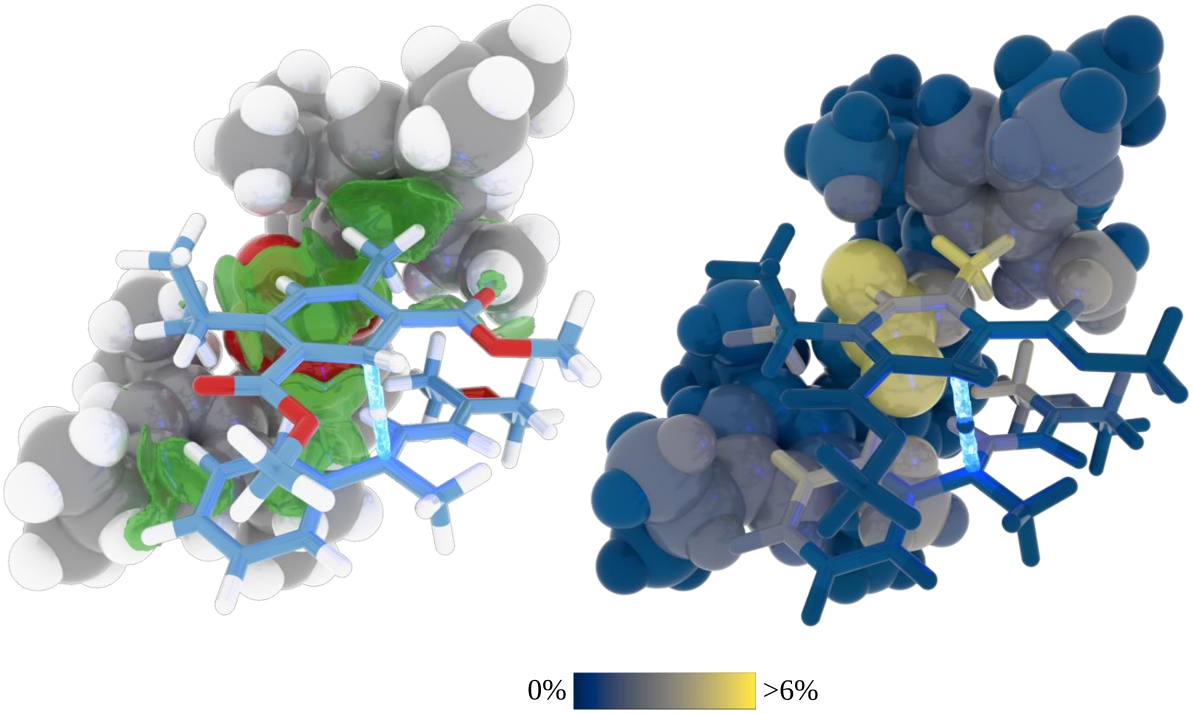

Figure S4: *δg^{inter} isosurface (left) and color-coded atoms according to their contribution to the overall interfragment interaction δG^{atom}(%) for **TS-2**.*

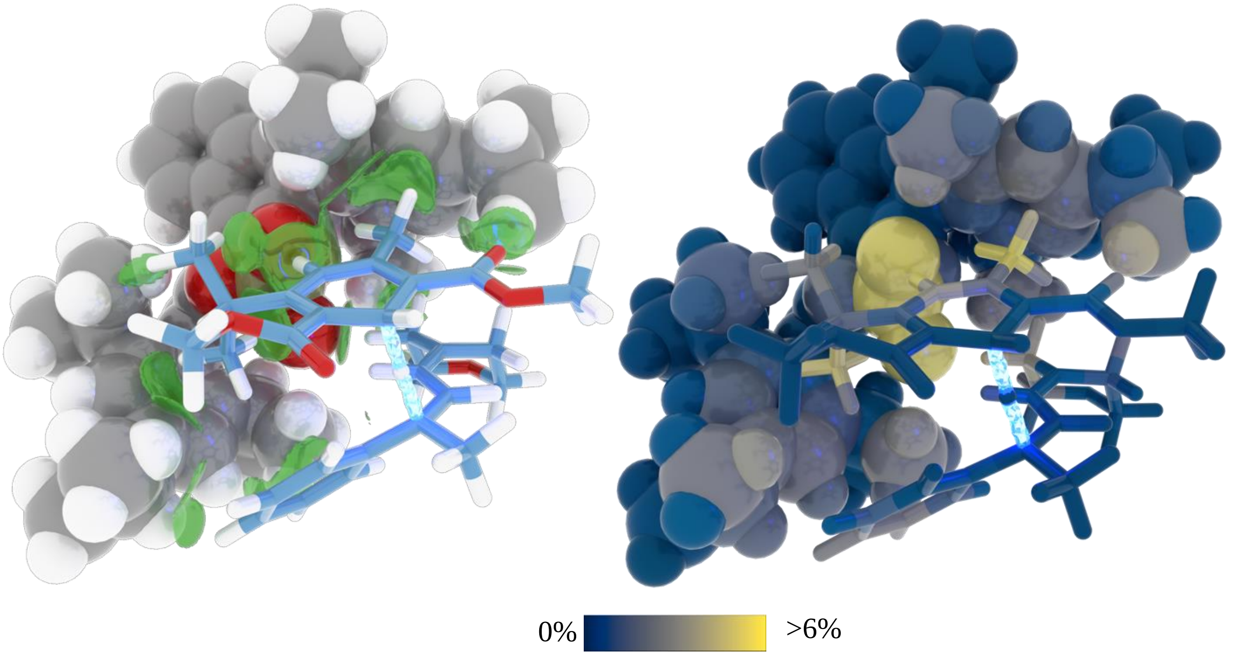

Figure S5: *δg^{inter} isosurface (left) and color-coded atoms according to their contribution to the overall interfragment interaction δG^{atom}(%) for* ***TS-2b****.*

Visual inspection reveals three hotspots for non-covalent interaction: the isopropyl group of the Hantzsch ester, the methyl group of the Hantzsch ester, as well as the α-CH_2 group of the morpholine fragment. The following quantitative analysis was made based on the percentage contribution of the individual atoms δG^{atom}(%) of these groups to the overall interfragment interaction:

Table S11: *Comparison of δG^{atom}(%) of the Hantzsch ester's isopropyl fragment between* ***TS-1****,* ***TS-2****, and* ***TS-2b****.*

**TS-1**		**TS-2**		**TS-2b**	
#	δG^{atom}(%)	#	δG^{atom}(%)	#	δG^{atom}(%)
62 (C)	1.15%	62 (C)	0.63%	62 (C)	1.43%
179 (H)	0.22%	179 (H)	0.26%	179 (H)	0.38%
178 (C)	4.28%	178 (C)	3.04%	178 (C)	5.20%
180 (H)	3.12%	180 (H)	2.97%	180 (H)	5.17%
181 (H)	4.37%	181 (H)	2.76%	181 (H)	2.16%
182 (H)	2.65%	182 (H)	1.32%	182 (H)	5.21%
174 (C)	1.87%	174 (C)	0.19%	174 (C)	2.30%
175 (H)	0.39%	175 (H)	0.23%	175 (H)	1.55%
176 (H)	2.94%	176 (H)	0.02%	176 (H)	3.28%
177 (H)	0.80%	177 (H)	0.07%	177 (H)	0.39%
**Σ**	**21.79%**	**Σ**	**11.49%**	**Σ**	**27.07%**

Table S12: Comparison of δG^{atom}(%) of the Hantzsch ester's methyl fragment between ***TS-1****,* ***TS-2****, and* ***TS-2b****.*

**TS-1**		**TS-2**		**TS-2b**	
#	δG^{atom}(%)	#	δG^{atom}(%)	#	δG^{atom}(%)
63 (C)	5.92%	63 (C)	7.81%	63 (C)	6.16%
64 (H)	6.87%	64 (H)	5.04%	64 (H)	2.76%
65 (H)	2.54%	65 (H)	4.87%	65 (H)	4.13%
66 (H)	3.26%	66 (H)	6.96%	66 (H)	6.71%
**Σ**	**18.59%**	**Σ**	**24.68%**	**Σ**	**19.76%**

Table S13: Comparison of δG^{atom}(%) of the α-CH_2 morpholine fragment between ***TS-1****,* ***TS-2****, and* ***TS-2b****.*

**TS-1**		**TS-2**		**TS-2b**	
#	δG^{atom}(%)	#	δG^{atom}(%)	#	δG^{atom}(%)
21 (C)	3.17%	29 (C)	4.01%	21 (C)	3.78%
23 (H)	2.36%	30 (H)	3.98%	23 (H)	3.44%
24 (H)	3.90%	31 (H)	4.36%	24 (H)	3.66%
**Σ**	**9.43%**	**Σ**	**12.35%**	**Σ**	**11.88%**

Table S14: Comparison of δG^{atom}(%) of the iminium phenyl fragment between ***TS-1****,* ***TS-2****, and* ***TS-2b****.*

**TS-1**		**TS-2**		**TS-2b**	
#	δG^{atom}(%)	#	δG^{atom}(%)	#	δG^{atom}(%)
39 (C)	0.27%	39 (C)	1.03%	39 (C)	0.22%
40 (C)	0.03%	40 (C)	2.99%	40 (C)	1.06%
41 (C)	0.62%	41 (C)	0.31%	41 (C)	0.21%
43 (C)	0.17%	43 (C)	0.36%	43 (C)	0.91%
44 (C)	0.01%	44 (C)	3.76%	44 (C)	2.48%
45 (C)	0.02%	45 (C)	1.74%	45 (C)	2.40%
46 (H)	1.25%	46 (H)	0.04%	46 (H)	0.03%
47 (H)	0.11%	47 (H)	0.07%	47 (H)	0.49%
48 (H)	0.00%	48 (H)	1.31%	48 (H)	2.42%
49 (H)	0.01%	49 (H)	3.16%	49 (H)	0.65%
50 (H)	0.00%	50 (H)	4.24%	50 (H)	2.52%
**Σ**	**2.49%**	**Σ**	**19.01%**	**Σ**	**13.39%**

Table S15: *Comparison of δG^{atom}(%) of the western 2-isopropyl fragment between* ***TS-1****,* ***TS-2****, and* ***TS-2b****.*

**TS-1**		**TS-2**		**TS-2b**	
#	δG^{atom}(%)	#	δG^{atom}(%)	#	δG^{atom}(%)
133 (C)	0.63%	133 (C)	0.56%	133 (C)	0.63%
139 (H)	0.24%	139 (H)	0.29%	139 (H)	0.37%
134 (C)	2.87%	134 (C)	3.11%	134 (C)	2.42%
135 (H)	1.35%	135 (H)	3.89%	135 (H)	1.18%
136 (H)	1.56%	136 (H)	1.16%	136 (H)	1.55%
137 (H)	3.99%	137 (H)	2.27%	137 (H)	3.14%
138 (C)	0.10%	138 (C)	0.10%	138 (C)	0.10%
140 (H)	0.04%	140 (H)	0.01%	140 (H)	0.01%
141 (H)	0.01%	141 (H)	0.06%	141 (H)	0.05%
142 (H)	0.05%	142 (H)	0.04%	142 (H)	0.05%
**Σ**	**10.84%**	**Σ**	**11.49%**	**Σ**	**9.51%**

Table S16: *Comparison of δG^{atom}(%) of the western 4-isopropyl fragment between* ***TS-1****,* ***TS-2****, and* ***TS-2b****.*

**TS-1**		**TS-2**		**TS-2b**	
#	δG^{atom}(%)	#	δG^{atom}(%)	#	δG^{atom}(%)
153 (C)	1.19%	153 (C)	1.40%	153 (C)	1.41%
168 (H)	1.05%	168 (H)	1.55%	168 (H)	1.47%
163 (C)	2.37%	163 (C)	1.42%	163 (C)	2.11%
164 (H)	0.65%	164 (H)	2.10%	164 (H)	0.60%
165 (H)	0.94%	165 (H)	0.37%	165 (H)	0.51%
166 (H)	3.84%	166 (H)	0.28%	166 (H)	3.26%
167 (C)	0.13%	167 (C)	0.14%	167 (C)	0.16%
169 (H)	0.04%	169 (H)	0.04%	169 (H)	0.06%
170 (H)	0.04%	170 (H)	0.03%	170 (H)	0.04%
171 (H)	0.03%	171 (H)	0.05%	171 (H)	0.04%
**Σ**	**10.28%**	**Σ**	**7.38%**	**Σ**	**9.66%**

Table S17: *Comparison of δG^{atom}(%) of the western 6-isopropyl fragment between* ***TS-1***, ***TS-2***, *and* ***TS-2b***.

**TS-1**		**TS-2**		**TS-2b**	
#	δG^{atom}(%)	#	δG^{atom}(%)	#	δG^{atom}(%)
143 (C)	0.42%	143 (C)	0.16%	143 (C)	0.31%
149 (H)	0.09%	149 (H)	0.04%	149 (H)	0.07%
144 (C)	0.06%	144 (C)	0.02%	144 (C)	0.05%
145 (H)	0.04%	145 (H)	0.00%	145 (H)	0.03%
146 (H)	0.01%	146 (H)	0.01%	146 (H)	0.01%
147 (H)	0.01%	147 (H)	0.00%	147 (H)	0.01%
148 (C)	1.42%	148 (C)	0.48%	148 (C)	1.10%
150 (H)	1.22%	150 (H)	0.06%	150 (H)	0.84%
151 (H)	0.18%	151 (H)	0.82%	151 (H)	0.18%
152 (H)	1.68%	152 (H)	0.34%	152 (H)	1.56%
**Σ**	**5.14%**	**Σ**	**1.93%**	**Σ**	**4.16%**

Table S18: *Comparison of δG^{atom}(%) of the eastern 2-isopropyl fragment between* ***TS-1***, ***TS-2***, *and* ***TS-2b***.

**TS-1**		**TS-2**		**TS-2b**	
#	δG^{atom}(%)	#	δG^{atom}(%)	#	δG^{atom}(%)
104 (C)	0.32%	104 (C)	1.66%	104 (C)	0.42%
110 (H)	0.08%	110 (H)	1.74%	110 (H)	0.10%
105 (C)	1.73%	105 (C)	2.98%	105 (C)	2.13%
106 (H)	0.57%	106 (H)	2.56%	106 (H)	1.86%
107 (H)	0.42%	107 (H)	3.50%	107 (H)	0.48%
108 (H)	1.89%	108 (H)	0.39%	108 (H)	2.39%
109 (C)	0.06%	109 (C)	0.22%	109 (C)	0.07%
111 (H)	0.01%	111 (H)	0.04%	111 (H)	0.01%
112 (H)	0.02%	112 (H)	0.06%	112 (H)	0.02%
113 (H)	0.04%	113 (H)	0.14%	113 (H)	0.04%
**Σ**	**5.14%**	**Σ**	**13.29%**	**Σ**	**7.51%**

Table S19: *Comparison of δG^{atom}(%) of the eastern 4-isopropyl fragment between **TS-1**, **TS-2**, and **TS-2b**.*

**TS-1**		**TS-2**		**TS-2b**	
#	δG^{atom}(%)	#	δG^{atom}(%)	#	δG^{atom}(%)
124 (C)	0.73%	124 (C)	0.49%	124 (C)	0.84%
159 (H)	0.37%	159 (H)	0.09%	159 (H)	0.42%
154 (C)	0.07%	154 (C)	0.08%	154 (C)	0.08%
155 (H)	0.02%	155 (H)	0.01%	155 (H)	0.02%
156 (H)	0.03%	156 (H)	0.01%	156 (H)	0.02%
157 (H)	0.01%	157 (H)	0.05%	157 (H)	0.03%
158 (C)	1.64%	158 (C)	1.16%	158 (C)	2.21%
160 (H)	0.80%	160 (H)	0.13%	160 (H)	0.45%
161 (H)	3.02%	161 (H)	1.29%	161 (H)	1.15%
162 (H)	0.30%	162 (H)	1.10%	162 (H)	3.94%
**Σ**	**6.99%**	**Σ**	**4.41%**	**Σ**	**9.16%**

Table S20: *Comparison of δG^{atom}(%) of the eastern 6-isopropyl fragment between **TS-1**, **TS-2**, and **TS-2b**.*

**TS-1**		**TS-2**		**TS-2b**	
#	δG^{atom}(%)	#	δG^{atom}(%)	#	δG^{atom}(%)
114 (C)	0.87%	114 (C)	0.50%	114 (C)	0.66%
120 (H)	0.47%	120 (H)	0.23%	120 (H)	0.20%
115 (C)	2.15%	115 (C)	0.76%	115 (C)	1.85%
116 (H)	0.82%	116 (H)	1.46%	116 (H)	0.30%
117 (H)	0.54%	117 (H)	0.14%	117 (H)	1.12%
118 (H)	3.63%	118 (H)	0.22%	118 (H)	2.80%
119 (C)	0.09%	119 (C)	0.05%	119 (C)	0.08%
121 (H)	0.01%	121 (H)	0.01%	121 (H)	0.02%
122 (H)	0.03%	122 (H)	0.02%	122 (H)	0.01%
123 (H)	0.03%	123 (H)	0.01%	123 (H)	0.05%
**Σ**	**8.64%**	**Σ**	**3.40%**	**Σ**	**7.09%**

f. 2006 System: Activation Energies

In order to investigate the activation energy computationally, we optimized the structure of the pre-transition state complex (**1d2a4a**).

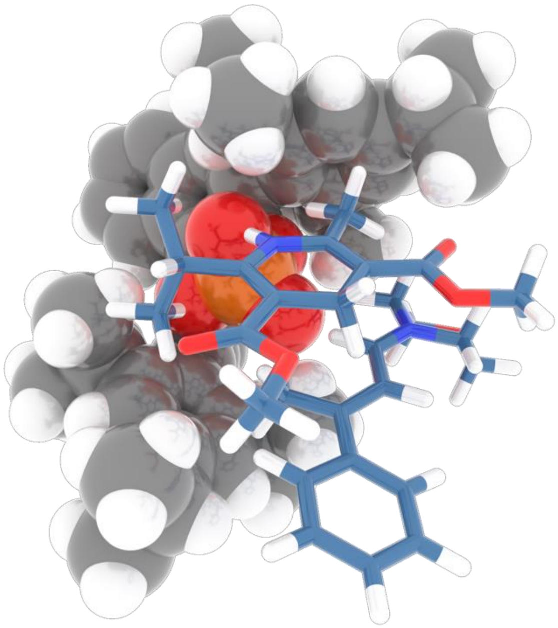

Figure S6: *Structure of the pre-transition state complex* ***1d2a4a****, obtained by relaxation of* ***TS-1****.*

Table S21: *Obtained thermochemical corrections at the PBE-D3(BJ)/def2-SVP level (323 K, 1 M) as well as single point energies for the pre-transition state complex* ***1d2a4a****; Gibbs free energies were calculated with respect to* ***TS-1****.*

TC 1.380282790774	E^{SP} / a.u.	**ΔΔ*G*(323 K)**
B3LYP-D3(BJ)/def2-TZVPP	–4117.81165	10.1 kcal/mol
B3LYP/def2-TZVPP	–4117.29809	12.1 kcal/mol
ωB97M-V/def2-TZVPP	–4118.27199	12.7 kcal/mol

We additionally conducted an IGMH analysis with the B3LYP-D3(BJ)/def2-TZVPP densities.

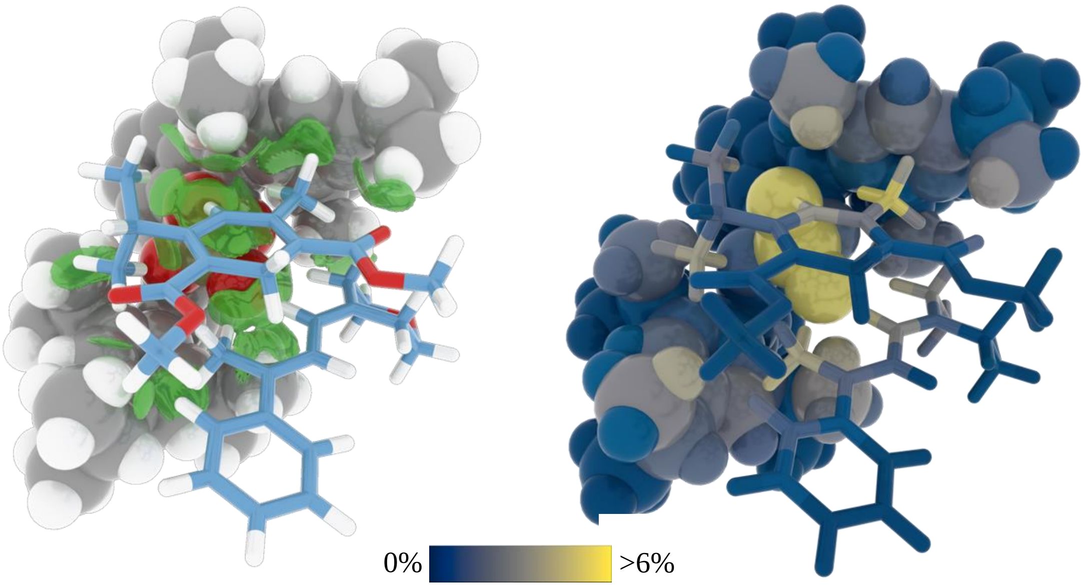

***Figure S7:** δg^{inter} isosurface (left) and color-coded atoms according to their contribution to the overall interfragment interaction δG^{atom}(%) for pre-transition state complex **1d2a4a**.*

Table S22: *Comparison of δG^{atom}(%) of the Hantzsch ester's isopropyl fragment between* ***1d2a4a*** *and* ***TS-1****.*

**1d2a4a**		**TS-2**	
#	δG^{atom}(%)	#	δG^{atom}(%)
62 (C)	1.04%	62 (C)	1.15%
179 (H)	0.19%	179 (H)	0.22%
178 (C)	3.79%	178 (C)	4.28%
180 (H)	2.68%	180 (H)	3.12%
181 (H)	4.02%	181 (H)	4.37%
182 (H)	2.32%	182 (H)	2.65%
174 (C)	1.88%	174 (C)	1.87%
175 (H)	0.38%	175 (H)	0.39%
176 (H)	2.98%	176 (H)	2.94%
177 (H)	0.87%	177 (H)	0.80%
**Σ**	**20.15%**	**Σ**	**21.79%**

Table S23: *Comparison of δG^{atom}(%) of the Hantzsch ester's methyl fragment between* ***1d2a4a*** *and* ***TS-1****.*

**1d2a4a**		**TS-1**	
#	δG^{atom}(%)	#	δG^{atom}(%)
63 (C)	5.90%	63 (C)	5.92%
64 (H)	3.55%	64 (H)	6.87%
65 (H)	6.69%	65 (H)	2.54%
66 (H)	2.52%	66 (H)	3.26%
**Σ**	**18.66%**	**Σ**	**18.59%**

Table S24: *Comparison of δG^{atom}(%) of the α-CH_2 morpholine fragment between* ***1d2a4a*** *and* ***TS-1****.*

**1d2a4a**		**TS-1**	
#	δG^{atom}(%)	#	δG^{atom}(%)
21 (C)	3.03%	21 (C)	3.17%
23 (H)	1.96%	23 (H)	2.36%
24 (H)	3.96%	24 (H)	3.90%
**Σ**	**8.95%**	**Σ**	**9.43%**

Table S25: *Comparison of δG^{atom}(%) of the iminium phenyl fragment between* ***1d2a4a*** *and* ***TS-1****.*

**1d2a4a**		**TS-1**	
#	δG^{atom}(%)	#	δG^{atom}(%)
39 (C)	0.49%	39 (C)	0.49%
40 (C)	0.12%	40 (C)	0.12%
41 (C)	0.95%	41 (C)	0.95%
43 (C)	0.34%	43 (C)	0.34%
44 (C)	0.03%	44 (C)	0.03%
45 (C)	0.05%	45 (C)	0.05%
46 (H)	1.46%	46 (H)	1.46%
47 (H)	0.21%	47 (H)	0.21%
48 (H)	0.01%	48 (H)	0.01%
49 (H)	0.05%	49 (H)	0.05%
50 (H)	0.00%	50 (H)	0.00%
**Σ**	**3.71%**	**Σ**	**3.71%**

Table S26: *Comparison of δG^{atom}(%) of the western 2-isopropyl fragment between* ***1d2a4a*** *and* ***TS-1****.*

**1d2a4a**		**TS-1**	
#	δG^{atom}(%)	#	δG^{atom}(%)
133 (C)	0.57%	133 (C)	0.63%
139 (H)	0.19%	139 (H)	0.24%
134 (C)	3.00%	134 (C)	2.87%
135 (H)	1.25%	135 (H)	1.35%
136 (H)	2.02%	136 (H)	1.56%
137 (H)	3.79%	137 (H)	3.99%
138 (C)	0.10%	138 (C)	0.10%
140 (H)	0.04%	140 (H)	0.04%
141 (H)	0.01%	141 (H)	0.01%
142 (H)	0.06%	142 (H)	0.05%
**Σ**	**11.03%**	**Σ**	**10.84%**

***Table S27:** Comparison of δG^{atom}(%) of the western 4-isopropyl fragment between **1d2a4a** and **TS-1**.*

**1d2a4a**		**TS-1**	
#	δG^{atom}(%)	#	δG^{atom}(%)
153 (C)	1.38%	153 (C)	1.19%
168 (H)	1.23%	168 (H)	1.05%
163 (C)	2.36%	163 (C)	2.37%
164 (H)	0.69%	164 (H)	0.65%
165 (H)	0.72%	165 (H)	0.94%
166 (H)	3.86%	166 (H)	3.84%
167 (C)	0.14%	167 (C)	0.13%
169 (H)	0.04%	169 (H)	0.04%
170 (H)	0.04%	170 (H)	0.04%
171 (H)	0.05%	171 (H)	0.03%
**Σ**	**10.51%**	**Σ**	**10.28%**

***Table S28:** Comparison of δG^{atom}(%) of the western 6-isopropyl fragment between **1d2a4a** and **TS-1**.*

**1d2a4a**		**TS-1**	
#	δG^{atom}(%)	#	δG^{atom}(%)
143 (C)	0.37%	143 (C)	0.42%
149 (H)	0.08%	149 (H)	0.09%
144 (C)	0.05%	144 (C)	0.06%
145 (H)	0.03%	145 (H)	0.04%
146 (H)	0.01%	146 (H)	0.01%
147 (H)	0.01%	147 (H)	0.01%
148 (C)	1.38%	148 (C)	1.42%
150 (H)	0.18%	150 (H)	1.22%
151 (H)	1.73%	151 (H)	0.18%
152 (H)	1.17%	152 (H)	1.68%
**Σ**	**5.01%**	**Σ**	**5.14%**

Table S29: *Comparison of δG^{atom}(%) of the eastern 2-isopropyl fragment between* ***1d2a4a*** *and* ***TS-1****.*

**1d2a4a**		**TS-1**	
#	δG^{atom}(%)	#	δG^{atom}(%)
104 (C)	0.30%	104 (C)	0.32%
110 (H)	0.07%	110 (H)	0.08%
105 (C)	1.63%	105 (C)	1.73%
106 (H)	1.55%	106 (H)	0.57%
107 (H)	0.40%	107 (H)	0.42%
108 (H)	1.71%	108 (H)	1.89%
109 (C)	0.06%	109 (C)	0.06%
111 (H)	0.01%	111 (H)	0.01%
112 (H)	0.02%	112 (H)	0.02%
113 (H)	0.04%	113 (H)	0.04%
**Σ**	**5.79%**	**Σ**	**5.14%**

Table S30: *Comparison of δG^{atom}(%) of the eastern 4-isopropyl fragment between* ***1d2a4a*** *and* ***TS-1****.*

**1d2a4a**		**TS-1**	
#	δG^{atom}(%)	#	δG^{atom}(%)
124 (C)	0.70%	124 (C)	0.73%
159 (H)	0.36%	159 (H)	0.37%
154 (C)	0.06%	154 (C)	0.07%
155 (H)	0.01%	155 (H)	0.02%
156 (H)	0.01%	156 (H)	0.03%
157 (H)	0.02%	157 (H)	0.01%
158 (C)	1.60%	158 (C)	1.64%
160 (H)	0.73%	160 (H)	0.80%
161 (H)	3.00%	161 (H)	3.02%
162 (H)	0.28%	162 (H)	0.30%
**Σ**	**6.77%**	**Σ**	**6.99%**

Table S31: *Comparison of δG^{atom}(%) of the eastern 6-isopropyl fragment between **1d2a4a** and **TS-1**.*

**1d2a4a**		**TS-1**	
#	δG^{atom}(%)	#	δG^{atom}(%)
114 (C)	0.91%	114 (C)	0.87%
120 (H)	0.52%	120 (H)	0.47%
115 (C)	2.33%	115 (C)	2.15%
116 (H)	1.00%	116 (H)	0.82%
117 (H)	0.55%	117 (H)	0.54%
118 (H)	3.87%	118 (H)	3.63%
119 (C)	0.10%	119 (C)	0.09%
121 (H)	0.03%	121 (H)	0.01%
122 (H)	0.03%	122 (H)	0.03%
123 (H)	0.02%	123 (H)	0.03%
**Σ**	**9.36%**	**Σ**	**8.64%**

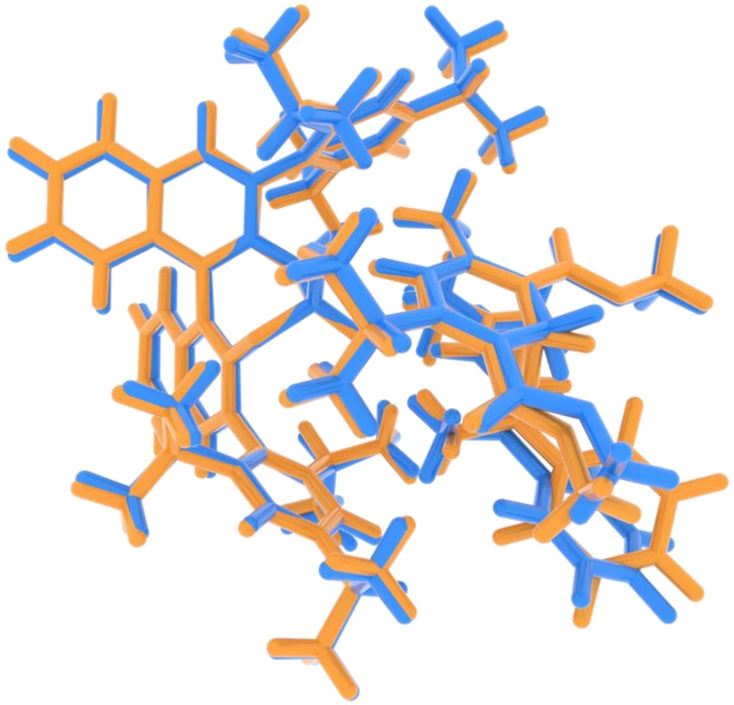

Figure S8: *Structural comparison between **1d2a4a** (blue) and **TS-1** (orange).*

Structural comparison of **1d2a4a** and **TS-1** indicates no observable qualitative differences.

g. Improved System: Transition State Overview

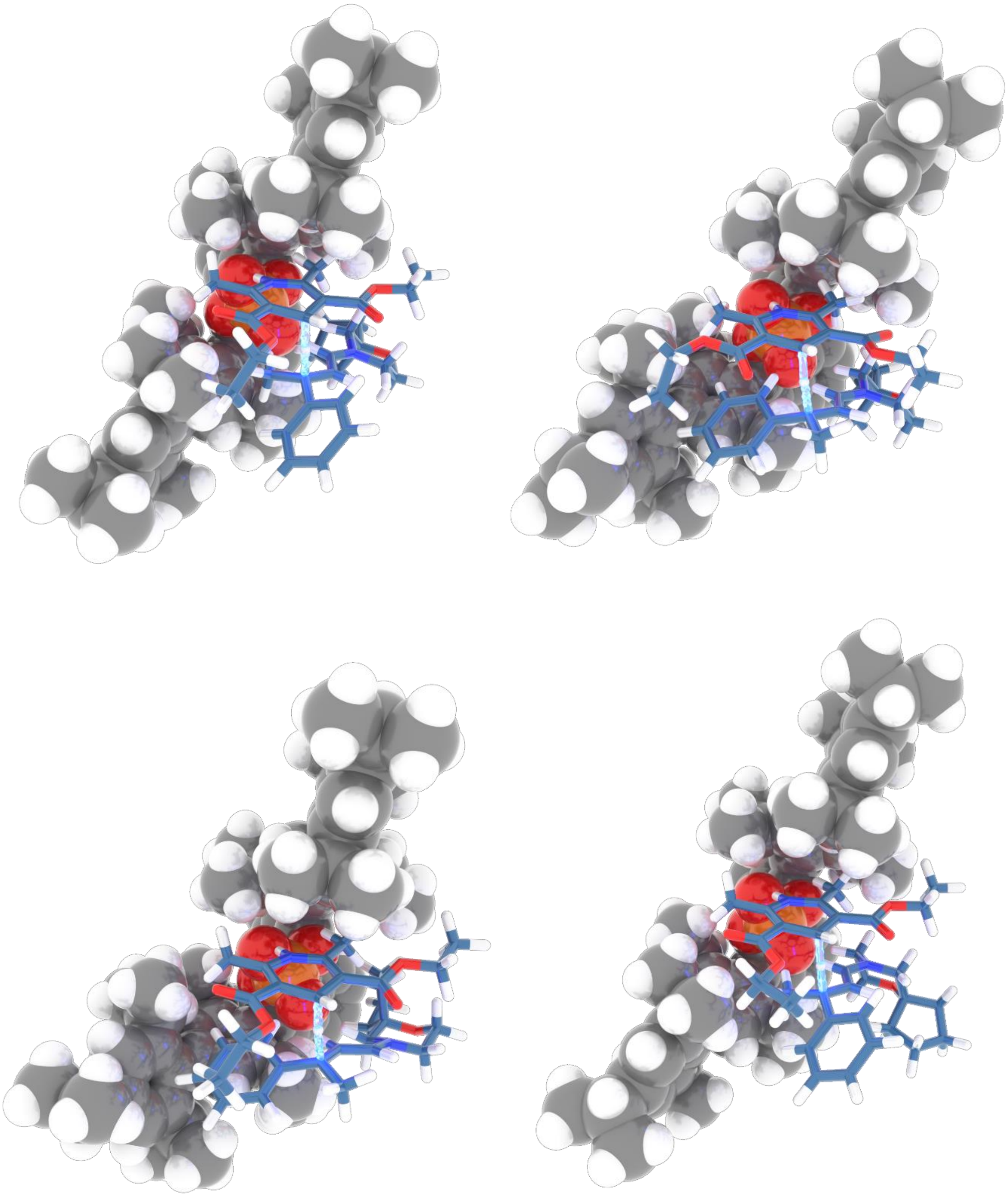

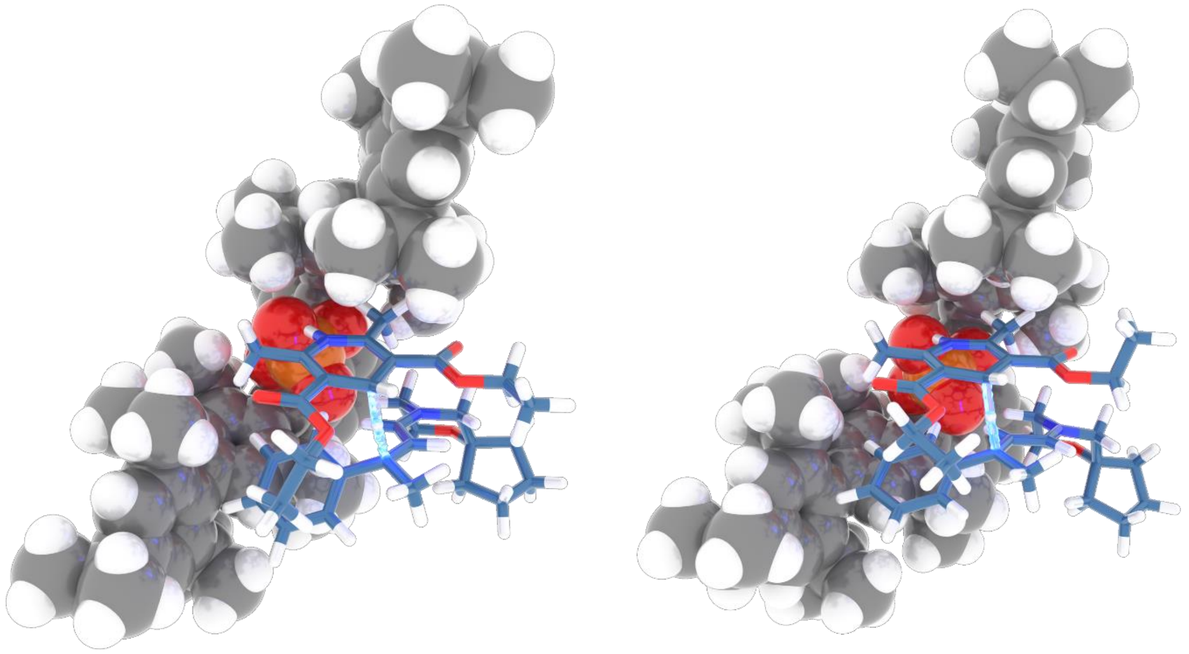

Figure S9: *Structural overview of* ***TS-3*** *(top left),* ***TS-4*** *(top right),* ***TS-4b*** *(middle left),* ***TS-3b*** *(middle right),* ***TS-4c*** *(bottom left), and* ***TS-4d*** *(bottom right).*

h. Improved System: Distortion–Interaction Analysis

Table S32: *Overview of the transition states at the CPCM(1,4-dioxane)-DLPNO-CCSD(T)/cc-pVTZ//PBE-D3(BJ)def2-SVP level of theory as well as computed enantiomeric ratios at 323 K.*

	$\Delta\Delta G^{\ddagger}$ (323 K)	Computed er (323 K)
**TS-3**	0.0 kcal/mol	–
**TS-4**	2.2 kcal/mol	97:3
**TS-4b**	2.5 kcal/mol	98:2
**TS-3b**	3.7 kcal/mol	>99:1
**TS-4c**	5.8 kcal/mol	>99:1
**TS-4d**	6.1 kcal/mol	>99:1

In analogy to the 2006 system, we could also locate transition states leading to the minor enantiomer similar to **TS-2** (**TS-4b** and **TS-4d**). However, it appears as though the extended size of the phosphate (**6**) stabilizes both phenyl fragments of the iminium ion in **TS-4** and **TS-4b** (and **TS-4c** and **TS-4d**, respectively) to a similar extent, eventually resulting in an energetic preference of **TS-4** over **TS-4b** (and **TS-4c** and **TS-4d**, respectively; please also see the LED calculations).

In addition to the Gibbs free energy comparison of the lowest-lying transition states, Distortion–Interaction Analysis[51] of the final single point energies was conducted:

Table S33: *Distortion–Interaction analysis between* ***TS-3****,* ***TS-4****, and* ***TS-4b*** *at the DLPNO-CCSD(T)/cc-pVTZ//PBE-D3(BJ)def2-SVP level of theory. Positive values: favored in* ***TS-3****, negative values: favored in* ***TS-4*** *or* ***TS-4b****.*

Component	Δ*E*(SP), TS-4 –TS-3	Δ*E*(SP), TS-4b – TS-3
total	0.6 kcal/mol	2.1 kcal/mol
IM + H	–1.3 kcal/mol	–1.7 kcal/mol
P	3.0 kcal/mol	4.2 kcal/mol
distortion	1.7 kcal/mol	2.5 kcal/mol
interaction	–1.1 kcal/mol	–0.4 kcal/mol

As has already been discussed for **TS-1** vs **TS-2** (see Table S5), the same arguments are valid for the improved system (catalyst distorted in the minor transition state to maximize London dispersion interactions leading to a lack of electrostatic interactions). Whereas interaction was found the driving force in the DIA between **TS-1** and **TS-2**, we now find distortion to be the main contributor for stereodifferentiation. It is plausible that, in order to maximize the London dispersion interactions, the phosphate anion in **TS-4** adapts a higher-energy conformation.

Table S34: *Distortion–Interaction analysis between* ***TS-3b****,* ***TS-4c****, and* ***TS-4d*** *at the DLPNO-CCSD(T)/cc-pVTZ//PBE-D3(BJ)def2-SVP level of theory. Positive values: favored in* ***TS-3****, negative values: favored in* ***TS-4*** *or* ***TS-4b****.*

Component	Δ*E*(SP), TS-4c –TS-3b	Δ*E*(SP), TS-4d – TS-3b
total	0.6 kcal/mol	1.4 kcal/mol
IM + H	–0.2 kcal/mol	–0.6 kcal/mol
P	2.6 kcal/mol	3.9 kcal/mol
distortion	2.4 kcal/mol	3.3 kcal/mol
interaction	–1.7 kcal/mol	–1.9 kcal/mol

For the sake of completeness, the same analysis was conducted for the three transition states corresponding to an *exo*-orientation of the spiro fragment (Table S34).

i. Improved System: Local Energy Decomposition

In order to calculate the interaction between the three fragments within the system, we chose to perform a Local Energy Decomposition analysis at the DLPNO-CCSD(T)/cc-pVTZ level of theory for all transition states.[43–50]

The resulting terms, corresponding to the decomposition of the HF interaction energy as well as the correlation interaction energy, can be found below. The sum of the individual elements is given at the top left corner of each matrix. The overall non-dispersion fragment interaction was calculated by adding the non-dispersion correlation contribution to the sum of electrostatic E_{elstat} and exchange E_{exch} interactions. All energies are given in kcal/mol.

Table S35: *Results of the LED analysis of* ***TS-3*** *at the DLPNO-CCSD(T)/cc-pVTZ level of theory.*

Electronic Preparation and Binding Energy

–157.8	H	IM	P
H	587.9	–769.9	–207.3
IM	–	322.9	–198.5
P	–	–	134.1

Overall Non-Dispersion

–66.7	H	IM	P
H	568.8	–717.7	–166.8
IM	–	286.3	–162.1
P	–	–	124.8

Electrostatics

97.0	H	IM	P
H	568.8	–599.3	–140.5
IM	–	286.3	–143.1
P	–	–	124.8

Exchange

–163.7	H	IM	P
H	–	–118.4	–26.3
IM	–	–	–19.0
P	–	–	–

Dispersion

–95.8	H	IM	P
H	–	–36.0	–30.4
IM	–	–	–29.4
P	–	–	–

The non-dispersion correlation contribution was found to be 4.2 kcal/mol, resulting in a total $\Delta E_{\text{non-disp}}$ of –62.6 kcal/mol.

Table S36: *Results of the LED analysis of* ***TS-4*** *at the DLPNO-CCSD(T)/cc-pVTZ level of theory.*

Electronic Preparation and Binding Energy

–161.9	H	IM	P
H	577.7	–800.1	–202.6
IM	–	335.0	–208.9
P	–	–	136.9

Overall Non-Dispersion

–63.7	H	IM	P
H	557.9	–717.0	–164.0
IM	–	297.5	–165.2
P	–	–	127.1

Electrostatics

106.3	H	IM	P
H	557.9	–594.5	–138.2
IM	–	297.5	–143.5
P	–	–	127.1

Exchange

–170.0	H	IM	P
H	–	–122.5	–25.9
IM	–	–	–21.7
P	–	–	–

Dispersion

–102.7	H	IM	P
H	–	–38.3	–28.7
IM	–	–	–35.6
P	–	–	–

The non-dispersion correlation contribution was found to be 4.0 kcal/mol, resulting in a total $\Delta E_{\text{non-disp}}$ of –59.7 kcal/mol.

Table S37: *Results of the LED analysis of* ***TS-4b*** *at the DLPNO-CCSD(T)/cc-pVTZ level of theory.*

Electronic Preparation and Binding Energy

–155.2	H	IM	P
H	501.4	–680.7	–196.7
IM	–	298.4	–211.4
P	–	–	133.8

Overall Non-Dispersion

–56.6	H	IM	P
H	483.7	–604.3	–158.3
IM	–	264.1	–165.6
P	–	–	123.8

Electrostatics

102.4	H	IM	P
H	483.7	–493.1	–133.2
IM	–	264.1	–143.0
P	–	–	123.8

Exchange

–158.9	H	IM	P
H	–	–111.2	–25.1
IM	–	–	–22.6
P	–	–	–

Dispersion

–101.5	H	IM	P
H	–	–35.5	–28.7
IM	–	–	–37.2
P	–	–	–

The non-dispersion correlation contribution was found to be 2.5 kcal/mol, resulting in a total $\Delta E_{\text{non-disp}}$ of –54.1 kcal/mol.

Table S38: *Results of the LED analysis of* ***TS-3b*** *at the DLPNO-CCSD(T)/cc-pVTZ level of theory.*

Electronic Preparation and Binding Energy

–149.4	H	IM	P
H	572.1	–782.7	–202.6
IM	–	310.8	–168.6
P	–	–	121.5

Overall Non-Dispersion

–69.6	H	IM	P
H	553.0	–703.7	–162.3
IM	–	275.3	–145.6
P	–	–	113.3

Electrostatics

88.6	H	IM	P
H	553.0	–584.9	–136.1
IM	–	275.3	–132.1
P	–	–	113.3

Exchange

–158.5	H	IM	P
H	–	–118.8	–26.2
IM	–	–	–13.5
P	–	–	–

Dispersion

–84.2	H	IM	P
H	–	–35.6	–30.3
IM	–	–	–18.3
P	–	–	–

The non-dispersion correlation contribution was found to be 4.2 kcal/mol, resulting in a total $\Delta E_{\text{non-disp}}$ of –65.7 kcal/mol.

Table S39: *Results of the LED analysis of* ***TS-4c*** *at the DLPNO-CCSD(T)/cc-pVTZ level of theory.*

Electronic Preparation and Binding Energy

–150.6	H	IM	P
H	543.5	–748.2	–196.0
IM	–	311.6	–188.6
P	–	–	127.0

Overall Non-Dispersion

–61.6	H	IM	P
H	525.1	–667.9	–157.5
IM	–	276.2	–155.4
P	–	–	118.0

Electrostatics

100.5	H	IM	P
H	525.1	–549.1	–132.1
IM	–	276.2	–137.6
P	–	–	118.0

Exchange

–162.1	H	IM	P
H	–	–118.9	–25.5
IM	–	–	–17.8
P	–	–	–

Dispersion

–92.0	H	IM	P
H	–	–36.8	–28.7
IM	–	–	–26.6
P	–	–	–

The non-dispersion correlation contribution was found to be 2.9 kcal/mol, resulting in a total $\Delta E_{\text{non-disp}}$ of –58.8 kcal/mol.

Table S40: *Results of the LED analysis of **TS-4d** at the DLPNO-CCSD(T)/cc-pVTZ level of theory.*

Electronic Preparation and Binding Energy

–149.8	H	IM	P
H	556.2	–766.1	–192.9
IM	–	318.2	–192.0
P	–	–	126.7

Overall Non-Dispersion

–58.9	H	IM	P
H	538.0	–685.8	–154.8
IM	–	283.0	–156.8
P	–	–	117.5

Electrostatics

105.2	H	IM	P
H	538.0	–565.5	–129.8
IM	–	283.0	–138.0
P	–	–	117.5

Exchange

–164.1	H	IM	P
H	–	–120.3	–25.0
IM	–	–	–18.8
P	–	–	–

Dispersion

–93.0	H	IM	P
H	–	–36.4	–28.4
IM	–	–	–28.2
P	–	–	–

The non-dispersion correlation contribution was found to be 1.9 kcal/mol, resulting in a total $\Delta E_{\text{non-disp}}$ of –57.0 kcal/mol.

Table S41: *Difference of LED analyses between **TS-3** and **TS-4** at the DLPNO-CCSD(T)/cc-pVTZ level of theory. Positive values: favored in **TS-3**, negative values: favored in **TS-4**.*

Electronic Preparation and Binding Energy

–4.1	H	IM	P
H	–10.2	–3.2	4.7
IM	–	12.1	–10.4
P	–	–	2.9

Overall Non-Dispersion

3.0	H	IM	P
H	–10.9	0.7	2.7
IM	–	11.2	–3.1
P	–	–	2.3

Electrostatics

9.4	H	IM	P
H	–10.9	4.8	2.3
IM	–	11.2	–0.4
P	–	–	2.3

Exchange

–6.3	H	IM	P
H	–	–4.1	0.4
IM	–	–	–2.7
P	–	–	–

Dispersion

–6.9	H	IM	P
H	–	–2.3	1.7
IM	–	–	–6.3
P	–	–	–

Taking into account the individual non-dispersion correlation contributions, an overall difference in $\Delta E_{\text{non-disp}}$ between both transition states of 2.9 kcal/mol was obtained.

Table S42: *Difference of LED analyses between* ***TS-3*** *and* ***TS-4b*** *at the DLPNO-CCSD(T)/cc-pVTZ level of theory. Positive values: favored in* ***TS-3****, negative values: favored in* ***TS-4b****.*

Electronic Preparation and Binding Energy

2.7	H	IM	P
H	–86.6	116.2	10.6
IM	–	–24.5	–12.9
P	–	–	–0.3

Overall Non-Dispersion

10.2	H	IM	P
H	–85.0	113.4	8.5
IM	–	–22.2	–3.5
P	–	–	–1.0

Electrostatics

5.4	H	IM	P
H	–85.0	106.2	7.3
IM	–	–22.2	0.1
P	–	–	–1.0

Exchange

4.8	H	IM	P
H	–	7.2	1.2
IM	–	–	–3.6
P	–	–	–

Dispersion

–5.7	H	IM	P
H	–	0.5	1.7
IM	–	–	–7.9
P	–	–	–

Taking into account the individual non-dispersion correlation contributions, an overall difference in $\Delta E_{\text{non-disp}}$ between both transition states of 8.5 kcal/mol was obtained.

Table S43: *Difference of LED analyses between* ***TS-3b*** *and* ***TS-4c*** *at the DLPNO-CCSD(T)/cc-pVTZ level of theory. Positive values: favored in* ***TS-3b****, negative values: favored in* ***TS-4c****.*

Electronic Preparation and Binding Energy

–1.2	H	IM	P
H	–28.6	34.5	6.6
IM	–	0.8	–20.0
P	–	–	5.5

Overall Non-Dispersion

8.3	H	IM	P
H	–27.9	35.7	4.7
IM	–	0.9	–9.7
P	–	–	4.6

Electrostatics

11.9	H	IM	P
H	–27.9	35.8	4.0
IM	–	0.9	–5.5
P	–	–	4.6

Exchange

–3.6	H	IM	P
H	–	–0.1	0.7
IM	–	–	–4.3
P	–	–	–

Dispersion

–7.9	H	IM	P
H	–	–1.1	1.7
IM	–	–	–8.4
P	–	–	–

Taking into account the individual non-dispersion correlation contributions, an overall difference in $\Delta E_{\text{non-disp}}$ between both transition states of 6.9 kcal/mol was obtained.

Table S44: *Difference of LED analyses between* ***TS-3b*** *and* ***TS-4d*** *at the DLPNO-CCSD(T)/cc-pVTZ level of theory. Positive values: favored in* ***TS-3b****, negative values: favored in* ***TS-4d****.*

Electronic Preparation and Binding Energy

–0.4	H	IM	P
**H**	–16.0	16.6	9.7
**IM**	–	7.4	–23.4
**P**	–	–	5.3

Overall Non-Dispersion

11.0	H	IM	P
**H**	–15.0	17.9	7.5
**IM**	–	7.7	–11.2
**P**	–	–	4.2

Electrostatics

16.6	H	IM	P
**H**	–15.0	19.4	6.3
**IM**	–	7.7	–5.9
**P**	–	–	4.2

Exchange

–5.6	H	IM	P
**H**	–	–1.5	1.2
**IM**	–	–	–5.3
**P**	–	–	–

Dispersion

–8.8	H	IM	P
**H**	–	–0.8	1.9
**IM**	–	–	–9.9
**P**	–	–	–

Taking into account the individual non-dispersion correlation contributions, an overall difference in $\Delta E_{\text{non-disp}}$ between both transition states of 8.7 kcal/mol was obtained.

j. Improved System: IGMH Analysis

An analysis according to the Independent Gradient Model based on Hirshfeld partition[34] was conducted with the Multiwfn program, version 3.8, using the B3LYP-D3(BJ)/def2-TZVPP densities.[35,36] Non-covalent interactions were subsequently plotted as an isosurface of δg^{inter} with an isovalue of 0.004. In a direct comparison, the atoms were colored by their respective δG^{atom}(%).

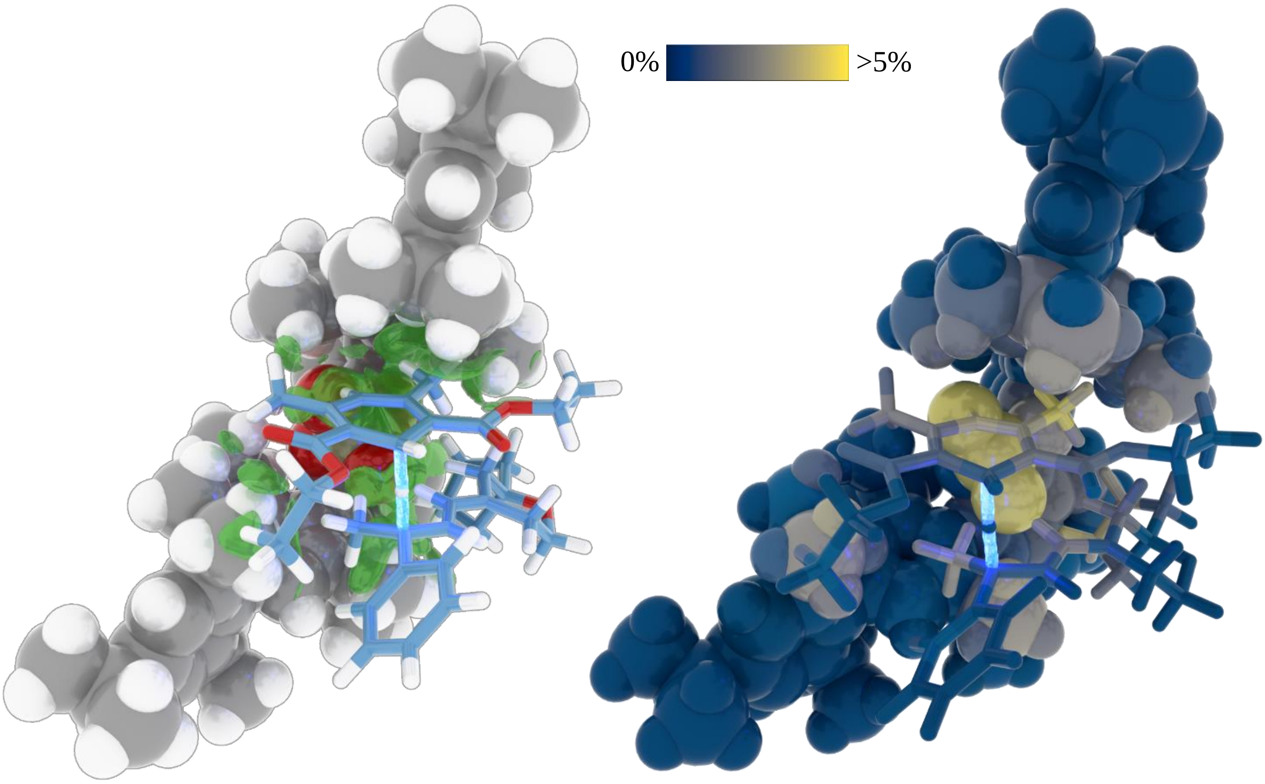

Figure S10: *δg^{inter} isosurface (left) and color-coded atoms according to their contribution to the overall interfragment interaction δG^{atom}(%) for* ***TS-3****.*

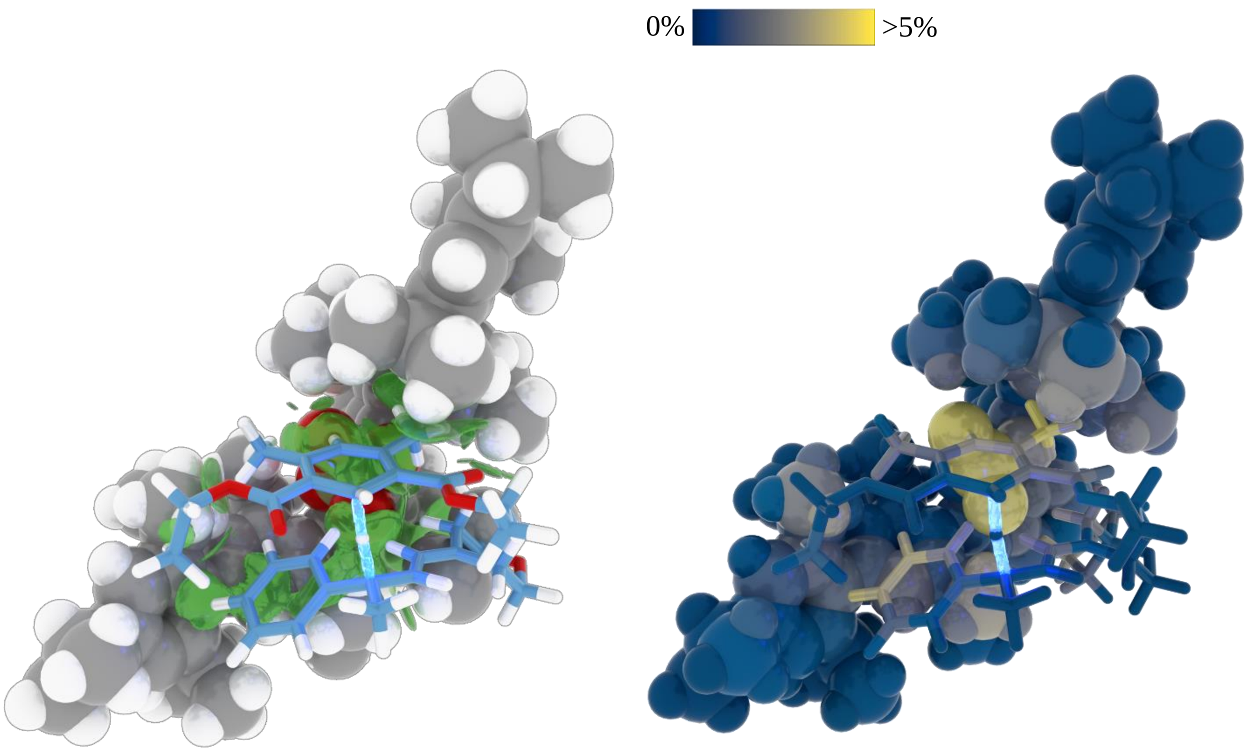

Figure S11: *δg^{inter} isosurface (left) and color-coded atoms according to their contribution to the overall interfragment interaction δG^{atom}(%) for **TS-4**.*

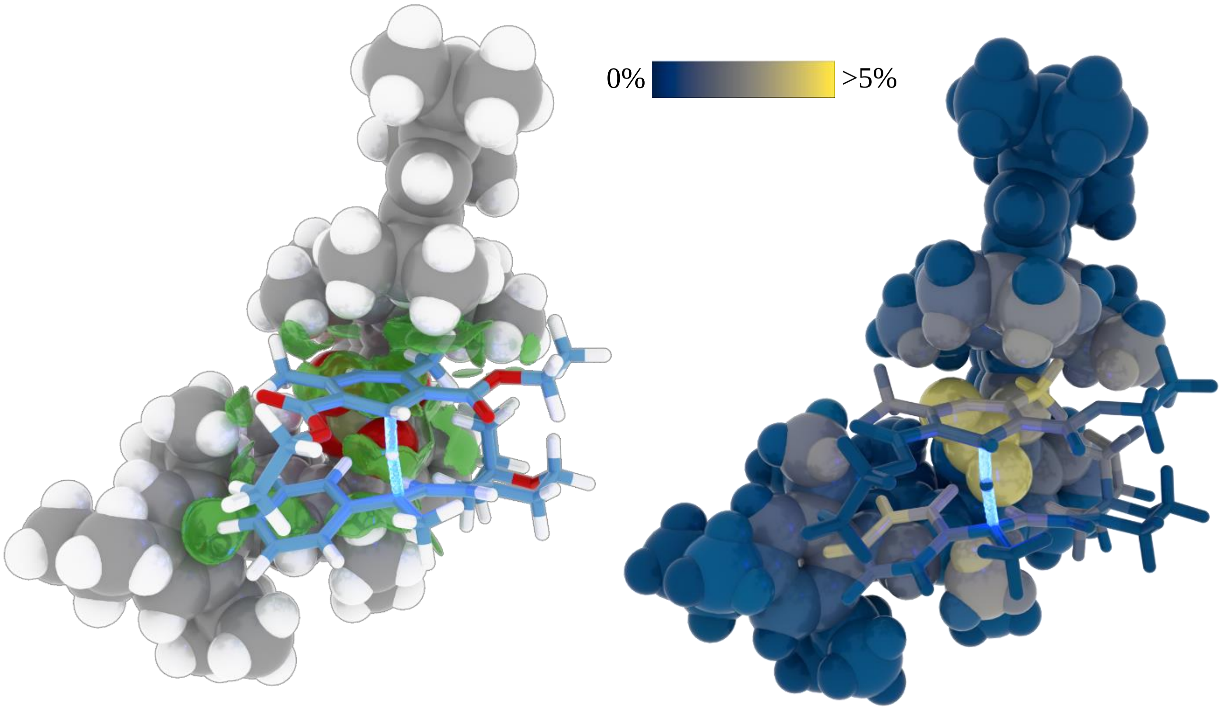

Figure S12: *δg^{inter} isosurface (left) and color-coded atoms according to their contribution to the overall interfragment interaction δG^{atom}(%) for **TS-4b**.*

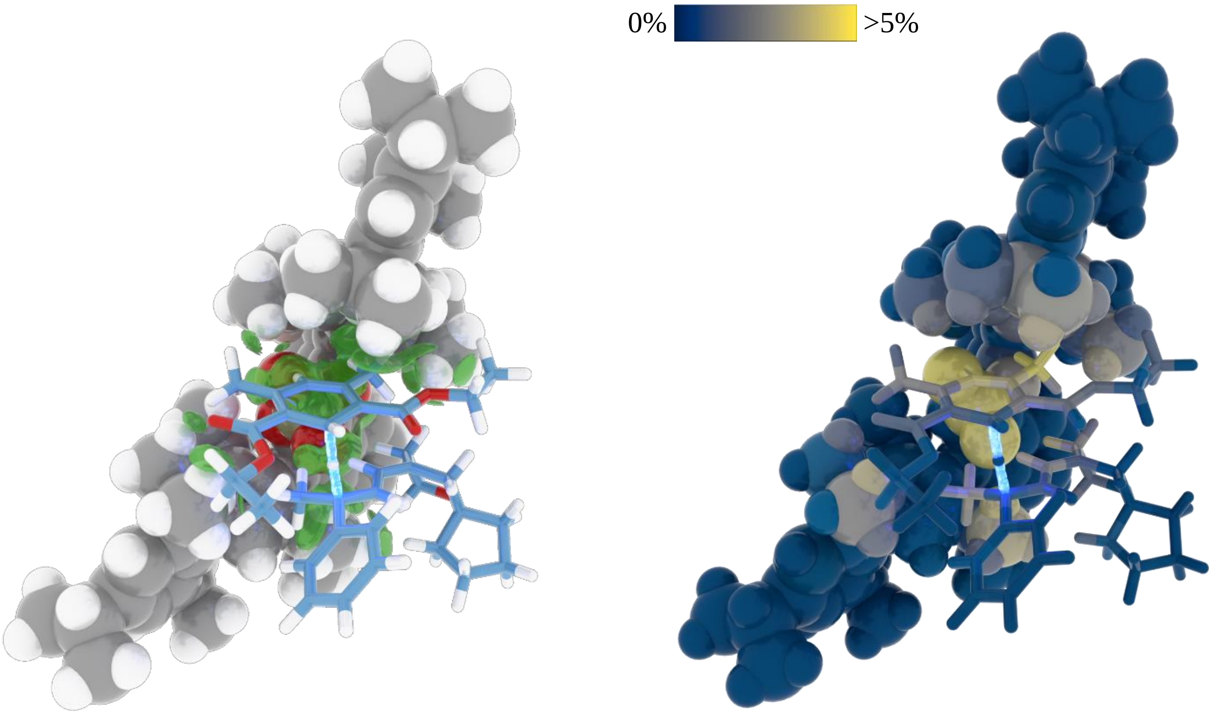

Figure S13: *δg^{inter} isosurface (left) and color-coded atoms according to their contribution to the overall interfragment interaction δG^{atom}(%) for* ***TS-3b****.*

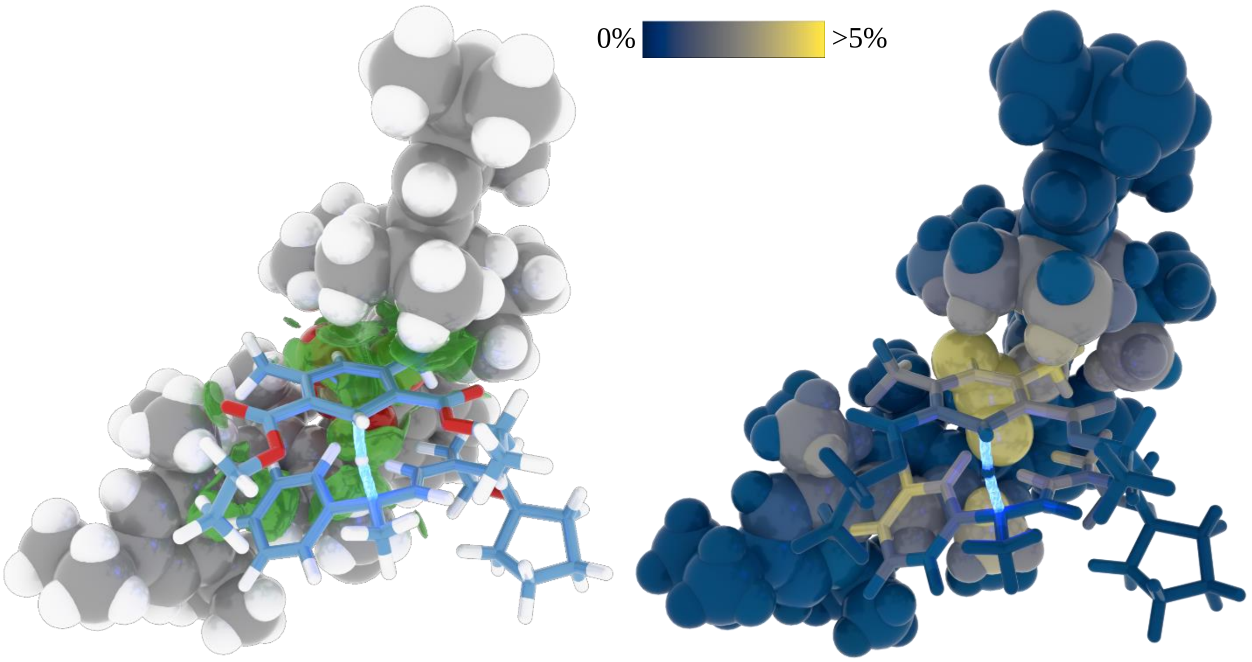

Figure S14: *δg^{inter} isosurface (left) and color-coded atoms according to their contribution to the overall interfragment interaction δG^{atom}(%) for* ***TS-4c****.*

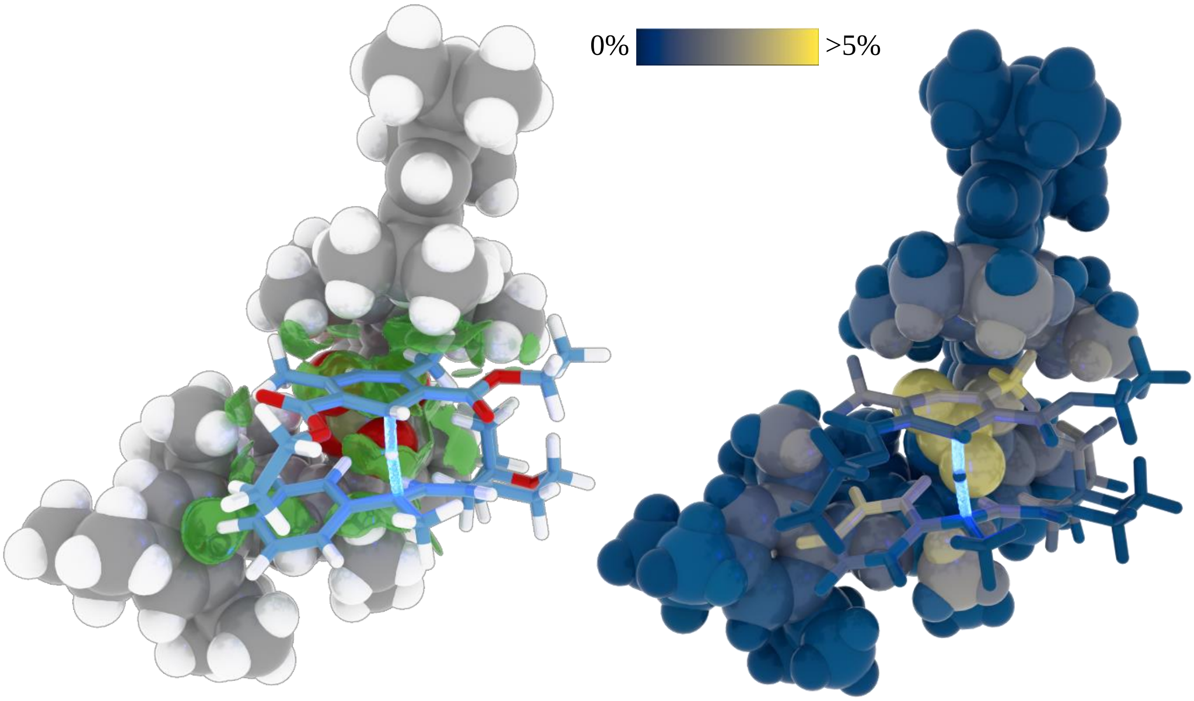

Figure S15: *δg^{inter} isosurface (left) and color-coded atoms according to their contribution to the overall interfragment interaction δG^{atom}(%) for **TS-4d**.*

Visual inspection reveals three hotspots for non-covalent interaction: the two methyl groups of the Hantzsch ester, the α-CH_2 group, as well as the spirocyclopentane ring of the morpholine fragment. The following quantitative analysis was made based on the percentage contribution of the individual atoms δG^{atom}(%) of these groups to the overall interfragment interaction:

Table S45: *Comparison of δG^{atom}(%) of the Hantzsch ester's western methyl fragment between **TS-3**, **TS-4**, and **TS-4b**.*

**TS-3**		**TS-4**		**TS-4b**	
#	δG^{atom}(%)	#	δG^{atom}(%)	#	δG^{atom}(%)
58 (C)	2.06%	59 (C)	1.76%	59 (C)	2.08%
59 (H)	1.95%	60 (H)	1.82%	60 (H)	0.88%
60 (H)	1.07%	61 (H)	0.51%	61 (H)	2.30%
61 (H)	1.19%	62 (H)	1.58%	62 (H)	1.08%
**Σ**	**6.27%**	**Σ**	**5.67%**	**Σ**	**6.34%**

Table S46: *Comparison of δG^{atom}(%) of the Hantzsch ester's eastern methyl fragment between **TS-3**, **TS-4**, and **TS-4b**.*

**TS-3**		**TS-4**		**TS-4b**	
#	δG^{atom}(%)	#	δG^{atom}(%)	#	δG^{atom}(%)
62 (C)	5.85%	63 (C)	5.89%	63 (C)	5.16%
63 (H)	2.36%	64 (H)	3.17%	64 (H)	3.54%
64 (H)	3.53%	65 (H)	2.94%	65 (H)	5.41%
65 (H)	6.03%	66 (H)	6.05%	66 (H)	1.74%
**Σ**	**17.07%**	**Σ**	**18.05%**	**Σ**	**15.85%**

Table S47: *Comparison of δG^{atom}(%) of the α-CH_2 morpholine fragment between **TS-3**, **TS-4**, and **TS-4b**.*

**TS-3**		**TS-4**		**TS-4b**	
#	δG^{atom}(%)	#	δG^{atom}(%)	#	δG^{atom}(%)
21 (C)	1.51%	29 (C)	1.83%	29 (C)	1.65%
23 (H)	1.01%	30 (H)	1.43%	30 (H)	2.66%
24 (H)	2.34%	31 (H)	2.81%	31 (H)	1.05%
**Σ**	**4.86%**	**Σ**	**5.36%**	**Σ**	**6.07%**

Table S48: *Comparison of δG^{atom}(%) of the spiro-cyclopentane morpholine fragment between **TS-3**, **TS-4**, and **TS-4b**.*

**TS-3**		**TS-4**		**TS-4b**	
#	δG^{atom}(%)	#	δG^{atom}(%)	#	δG^{atom}(%)
238 (C)	1.26%	233 (C)	1.24%	233 (C)	2.12%
247 (H)	1.71%	239 (H)	0.54%	239 (H)	1.19%
248 (H)	0.25%	240 (H)	1.81%	240 (H)	2.73%
242 (C)	3.74%	238 (C)	1.81%	238 (C)	2.32%
245 (H)	2.67%	241 (H)	0.35%	241 (H)	0.99%
246 (H)	4.33%	242 (H)	2.76%	242 (H)	3.01%
239 (C)	2.74%	235 (C)	2.85%	235 (C)	1.60%
243 (H)	1.75%	243 (H)	3.59%	243 (H)	2.45%
244 (H)	3.11%	244 (H)	1.72%	244 (H)	0.21%
237 (C)	0.88%	234 (C)	1.12%	234 (C)	1.42%
240 (H)	0.15%	236 (H)	1.51%	236 (H)	2.26%
241 (H)	1.29%	237 (H)	0.20%	237 (H)	0.42%
**Σ**	**23.88%**	**Σ**	**19.50%**	**Σ**	**20.72%**

Table S49: *Comparison of δG^{atom}(%) of the iminium phenyl fragment between **TS-3b**, **TS-4c**, and **TS-4d**.*

**TS-3b**		**TS-4c**		**TS-4d**	
#	δG^{atom}(%)	#	δG^{atom}(%)	#	δG^{atom}(%)
37 (C)	0.27%	38 (C)	0.71%	38 (C)	1.01%
38 (C)	0.51%	39 (C)	2.30%	39 (C)	2.58%
42 (C)	0.14%	43 (C)	3.96%	43 (C)	3.99%
43 (C)	0.02%	44 (C)	3.72%	44 (C)	3.35%
41 (C)	0.01%	42 (C)	1.01%	42 (C)	0.92%
39 (C)	0.04%	40 (C)	0.40%	40 (C)	0.44%
47 (H)	0.73%	48 (H)	1.93%	48 (H)	2.27%
48 (H)	0.07%	49 (H)	3.27%	49 (H)	3.80%
46 (H)	0.00%	47 (H)	4.36%	47 (H)	3.95%
45 (H)	0.00%	46 (H)	0.49%	46 (H)	0.42%
44 (H)	0.01%	45 (H)	0.06%	45 (H)	0.07%
**Σ**	**1.80%**	**Σ**	**22.21%**	**Σ**	**22.80%**

Table S50: *Comparison of δG^{atom}(%) of the western 2-isopropyl fragment (first arene) between* ***TS-3****,* ***TS-4****, and* ***TS-4b****.*

**TS-3**		**TS-4**		**TS-4b**	
#	δG^{atom}(%)	#	δG^{atom}(%)	#	δG^{atom}(%)
140 (C)	1.11%	141 (C)	1.22%	141 (C)	1.05%
146 (H)	1.56%	147 (H)	3.05%	147 (H)	2.69%
141 (C)	2.91%	142 (C)	3.48%	142 (C)	3.86%
142 (H)	3.60%	143 (H)	1.67%	143 (H)	1.67%
143 (H)	1.80%	144 (H)	1.59%	144 (H)	0.66%
144 (H)	1.10%	145 (H)	0.33%	145 (H)	40.26%
145 (C)	0.25%	146 (C)	1.63%	146 (C)	1.35%
147 (H)	0.06%	245 (H)	0.25%	245 (H)	0.09%
148 (H)	0.05%	246 (H)	0.09%	246 (H)	0.04%
149 (H)	0.18%	247 (H)	7.00%	247 (H)	0.22%
**Σ**	**12.62%**	**Σ**	**20.31%**	**Σ**	**11.89%**

Table S51: *Comparison of δG^{atom}(%) of the western 6-isopropyl fragment (first arene) between* ***TS-3****,* ***TS-4****, and* ***TS-4b****.*

**TS-3**		**TS-4**		**TS-4b**	
#	δG^{atom}(%)	#	δG^{atom}(%)	#	δG^{atom}(%)
150 (C)	0.02%	148 (C)	0.02%	148 (C)	0.01%
156 (H)	0.01%	154 (H)	0.00%	154 (H)	0.00%
151 (C)	0.00%	149 (C)	0.00%	149 (C)	0.00%
152 (H)	0.00%	150 (H)	0.00%	150 (H)	0.00%
153 (H)	0.00%	151 (H)	0.00%	151 (H)	0.00%
154 (H)	0.00%	152 (H)	0.03%	152 (H)	0.01%
155 (C)	0.04%	153 (C)	0.01%	153 (C)	0.01%
157 (H)	0.02%	155 (H)	0.00%	155 (H)	0.00%
158 (H)	0.01%	156 (H)	0.08%	156 (H)	0.02%
159 (H)	0.12%	157 (H)	0.02%	157 (H)	0.01%
**Σ**	**0.22%**	**Σ**	**0.16%**	**Σ**	**0.06%**

Table S52: *Comparison of δG^{atom}(%) of the western 2-isopropyl fragment (second arene) between* ***TS-3****,* ***TS-4****, and* ***TS-4b****.*

**TS-3**		**TS-4**		**TS-4b**	
#	δG^{atom}(%)	#	δG^{atom}(%)	#	δG^{atom}(%)
205 (C)	0.00%	203 (C)	0.14%	203 (C)	0.17%
211 (H)	0.00%	209 (H)	0.01%	209 (H)	0.02%
206 (C)	0.00%	204 (C)	0.00%	204 (C)	0.01%
207 (H)	0.00%	205 (H)	0.00%	205 (H)	0.00%
208 (H)	0.00%	206 (H)	0.00%	206 (H)	0.00%
209 (H)	0.00%	207 (H)	0.16%	207 (H)	0.28%
210 (C)	0.00%	208 (C)	0.06%	208 (C)	0.06%
212 (H)	0.00%	210 (H)	0.01%	210 (H)	0.02%
213 (H)	0.00%	211 (H)	0.06%	211 (H)	0.12%
214 (H)	0.01%	212 (H)	0.29%	212 (H)	0.49%
**Σ**	**0.01%**	**Σ**	**0.73%**	**Σ**	**1.17%**

Table S53: *Comparison of δG^{atom}(%) of the western 4-isopropyl fragment (second arene) between* ***TS-3****,* ***TS-4****, and* ***TS-4b****.*

**TS-3**		**TS-4**		**TS-4**	
#	δG^{atom}(%)	#	δG^{atom}(%)	#	δG^{atom}(%)
225 (C)	0.00%	223 (C)	0.13%	223 (C)	0.20%
231 (H)	0.00%	229 (H)	0.40%	229 (H)	0.03%
226 (C)	0.00%	224 (C)	0.27%	224 (C)	0.02%
227 (H)	0.00%	225 (H)	0.07%	225 (H)	0.00%
228 (H)	0.00%	226 (H)	0.71%	226 (H)	0.01%
229 (H)	0.00%	227 (H)	0.02%	227 (H)	0.38%
230 (C)	1.00%	228 (C)	0.04%	228 (C)	0.05%
232 (H)	0.01%	230 (H)	0.00%	230 (H)	0.51%
233 (H)	0.00%	231 (H)	0.00%	231 (H)	0.04%
234 (H)	0.02%	232 (H)	0.01%	232 (H)	0.27%
**Σ**	**1.03%**	**Σ**	**1.65%**	**Σ**	**1.51%**

Table S54: *Comparison of δG^{atom}(%) of the western 6-isopropyl fragment (second arene) between* ***TS-3****,* ***TS-4****, and* ***TS-4b****.*

**TS-3**		**TS-4**		**TS-4b**	
#	δG^{atom}(%)	#	δG^{atom}(%)	#	δG^{atom}(%)
215 (C)	0.93%	213 (C)	0.86%	213 (C)	0.78%
221 (H)	1.02%	219 (H)	2.02%	219 (H)	1.43%
216 (C)	2.60%	214 (C)	1.03%	214 (C)	0.62%
217 (H)	0.73%	215 (H)	3.04%	215 (H)	2.29%
218 (H)	3.30%	216 (H)	0.52%	216 (H)	0.25%
219 (H)	2.00%	217 (H)	0.10%	217 (H)	0.08%
220 (C)	0.56%	218 (C)	0.64%	218 (C)	0.54%
222 (H)	0.12%	220 (H)	0.03%	220 (H)	0.02%
223 (H)	1.00%	221 (H)	0.02%	221 (H)	0.02%
224 (H)	0.11%	222 (H)	0.04%	222 (H)	0.03%
**Σ**	**12.37%**	**Σ**	**8.30%**	**Σ**	**6.06%**

Table S55: *Comparison of δG^{atom}(%) of the eastern 2-isopropyl fragment (first arene) between* ***TS-3****,* ***TS-4****, and* ***TS-4b****.*

**TS-3**		**TS-4**		**TS-4b**	
#	δG^{atom}(%)	#	δG^{atom}(%)	#	δG^{atom}(%)
111 (C)	0.86%	112 (C)	0.91%	112 (C)	1.16%
117 (H)	0.74%	118 (H)	1.44%	118 (H)	2.03%
112 (C)	2.04%	113 (C)	2.02%	113 (C)	0.49%
113 (H)	0.68%	114 (H)	0.21%	114 (H)	1.15%
114 (H)	1.10%	115 (H)	0.92%	115 (H)	2.74%
115 (H)	2.92%	116 (H)	0.12%	116 (H)	0.18%
116 (C)	0.12%	117 (C)	0.90%	117 (C)	1.28%
118 (H)	0.02%	119 (H)	0.02%	119 (H)	0.11%
119 (H)	0.06%	120 (H)	0.03%	120 (H)	0.04%
120 (H)	0.03%	121 (H)	0.07%	121 (H)	0.05%
**Σ**	**8.57%**	**Σ**	**6.64%**	**Σ**	**9.23%**

Table S56: *Comparison of δG^{atom}(%) of the eastern 6-isopropyl fragment (first arene) between* ***TS-3****,* ***TS-4****, and* ***TS-4b****.*

**TS-3**		**TS-4**		**TS-4b**	
#	δG^{atom}(%)	#	δG^{atom}(%)	#	δG^{atom}(%)
121 (C)	0.41%	122 (C)	0.45%	122 (C)	0.40%
127 (H)	0.24%	128 (H)	0.76%	128 (H)	0.86%
122 (C)	0.81%	123 (C)	1.43%	123 (C)	1.68%
123 (H)	0.24%	124 (H)	0.21%	124 (H)	0.26%
124 (H)	0.19%	125 (H)	0.15%	125 (H)	0.20%
125 (H)	1.58%	126 (H)	0.04%	126 (H)	0.03%
126 (C)	0.04%	127 (C)	0.26%	127 (C)	0.25%
128 (H)	0.01%	129 (H)	0.01%	129 (H)	0.01%
129 (H)	0.01%	130 (H)	0.01%	130 (H)	0.01%
130 (H)	0.01%	131 (H)	0.01%	131 (H)	0.01%
**Σ**	**3.54%**	**Σ**	**3.33%**	**Σ**	**3.71%**

Table S57: *Comparison of δG^{atom}(%) of the eastern 2-isopropyl fragment (second arene) between* ***TS-3****,* ***TS-4****, and* ***TS-4b****.*

**TS-3**		**TS-4**		**TS-4b**	
#	δG^{atom}(%)	#	δG^{atom}(%)	#	δG^{atom}(%)
170 (C)	0.01%	168 (C)	0.01%	168 (C)	0.01%
176 (H)	0.01%	174 (H)	0.00%	174 (H)	0.00%
171 (C)	0.00%	169 (C)	0.00%	169 (C)	0.00%
172 (H)	0.00%	170 (H)	0.00%	170 (H)	0.00%
173 (H)	0.00%	171 (H)	0.00%	171 (H)	0.00%
174 (H)	0.00%	172 (H)	0.00%	172 (H)	0.00%
175 (C)	0.00%	173 (C)	0.01%	173 (C)	0.01%
177 (H)	0.00%	175 (H)	0.00%	175 (H)	0.01%
178 (H)	0.01%	176 (H)	0.00%	176 (H)	0.00%
179 (H)	0.00%	177 (H)	0.01%	177 (H)	0.00%
**Σ**	**0.03%**	**Σ**	**0.03%**	**Σ**	**0.03%**

Table S58: *Comparison of δG^{atom}(%) of the eastern 4-isopropyl fragment (second arene) between **TS-3**, **TS-4**, and **TS-4b**.*

**TS-3**		**TS-4**		**TS-4b**	
#	δG^{atom}(%)	#	δG^{atom}(%)	#	δG^{atom}(%)
190 (C)	0.00%	188 (C)	0.00%	188 (C)	0.00%
196 (H)	0.00%	194 (H)	0.00%	194 (H)	0.00%
191 (C)	0.00%	189 (C)	0.00%	189 (C)	0.00%
192 (H)	0.00%	190 (H)	0.00%	190 (H)	0.00%
193 (H)	0.00%	191 (H)	0.00%	191 (H)	0.00%
194 (H)	0.00%	192 (H)	0.00%	192 (H)	0.00%
195 (C)	0.00%	193 (C)	0.00%	193 (C)	0.00%
197 (H)	0.00%	195 (H)	0.00%	195 (H)	0.00%
198 (H)	0.00%	196 (H)	0.00%	196 (H)	0.00%
199 (H)	0.00%	197 (H)	0.00%	197 (H)	0.00%
**Σ**	**0.00%**	**Σ**	**0.00%**	**Σ**	**0.00%**

Table S59: *Comparison of δG^{atom}(%) of the eastern 6-isopropyl fragment (second arene) between **TS-3**, **TS-4**, and **TS-4b**.*

**TS-3**		**TS-4**		**TS-4b**	
#	δG^{atom}(%)	#	δG^{atom}(%)	#	δG^{atom}(%)
180 (C)	1.55%	178 (C)	1.31%	178 (C)	1.40%
186 (H)	2.35%	184 (H)	0.71%	184 (H)	1.11%
181 (C)	1.04%	179 (C)	0.24%	179 (C)	0.13%
182 (H)	0.38%	180 (H)	0.07%	180 (H)	1.77%
183 (H)	0.12%	181 (H)	0.96%	181 (H)	0.41%
184 (H)	1.57%	182 (H)	1.90%	182 (H)	1.88%
185 (C)	2.09%	183 (C)	2.12%	183 (C)	2.09%
187 (H)	2.76%	185 (H)	0.22%	185 (H)	2.53%
188 (H)	0.23%	186 (H)	1.19%	186 (H)	0.20%
189 (H)	1.32%	187 (H)	2.55%	187 (H)	1.17%
**Σ**	**13.41%**	**Σ**	**11.27%**	**Σ**	**12.69%**

Table S60: *Comparison of δG^{atom}(%) of the Hantzsch ester's western methyl fragment between* ***TS-3b****,* ***TS-4c****, and* ***TS-4d****.*

**TS-3b**		**TS-4c**		**TS-4d**	
#	δG^{atom}(%)	#	δG^{atom}(%)	#	δG^{atom}(%)
60 (C)	2.38%	59 (C)	1.96%	59 (C)	2.00%
61 (H)	1.09%	60 (H)	2.17%	60 (H)	2.08%
62 (C)	1.40%	61 (C)	0.64%	61 (C)	0.61%
63 (H)	2.47%	62 (H)	1.40%	62 (H)	1.67%
**Σ**	**7.34%**	**Σ**	**6.17%**	**Σ**	**6.36%**

Table S61: *Comparison of δG^{atom}(%) of the Hantzsch ester's eastern methyl fragment between* ***TS-3b****,* ***TS-4c****, and* ***TS-4d****.*

**TS-3b**		**TS-4c**		**TS-4d**	
#	δG^{atom}(%)	#	δG^{atom}(%)	#	δG^{atom}(%)
64 (C)	7.46%	63 (C)	7.13%	63 (C)	7.11%
65 (H)	5.42%	64 (H)	4.24%	64 (H)	3.55%
66 (H)	7.57%	65 (H)	7.26%	65 (H)	3.88%
67 (H)	2.62%	66 (H)	3.04%	66 (H)	6.99%
**Σ**	**23.07%**	**Σ**	**21.67%**	**Σ**	**21.53%**

Table S62: *Comparison of δG^{atom}(%) of the α-CH_2 morpholine fragment between* ***TS-3b****,* ***TS-4c****, and* ***TS-4d****.*

**TS-3b**		**TS-4c**		**TS-4d**	
#	δG^{atom}(%)	#	δG^{atom}(%)	#	δG^{atom}(%)
21 (C)	2.69%	21 (C)	2.87%	21 (C)	2.62%
23 (H)	1.66%	23 (H)	2.10%	23 (H)	2.12%
24 (H)	4.00%	24 (H)	4.01%	24 (H)	3.62%
**Σ**	**8.35%**	**Σ**	**8.98%**	**Σ**	**8.36%**

Table S63: *Comparison of δG^{atom}(%) of the spiro-cyclopentane morpholine fragment between* ***TS-3b****,* ***TS-4c****, and* ***TS-4d****.*

**TS-3b**		**TS-4c**		**TS-4d**	
#	δG^{atom}(%)	#	δG^{atom}(%)	#	δG^{atom}(%)
237 (C)	0.65%	233 (C)	0.18%	233 (C)	0.18%
244 (H)	1.05%	240 (H)	0.31%	240 (H)	0.27%
243 (H)	0.42%	239 (H)	0.10%	239 (H)	0.15%
245 (H)	0.04%	238 (C)	0.02%	238 (C)	0.02%
246 (H)	0.03%	241 (H)	0.01%	241 (H)	0.01%
242 (C)	0.08%	242 (H)	0.01%	242 (H)	0.00%
239 (C)	0.02%	235 (C)	0.00%	235 (C)	0.00%
247 (H)	0.00%	243 (H)	0.00%	243 (H)	0.00%
248 (H)	0.01%	244 (H)	0.00%	244 (H)	0.00%
238 (C)	0.03%	234 (C)	0.02%	234 (C)	0.02%
240 (H)	0.01%	236 (H)	0.01%	236 (H)	0.01%
241 (H)	0.01%	237 (H)	0.01%	237 (H)	0.02%
**Σ**	**2.35%**	**Σ**	**0.67%**	**Σ**	**0.68%**

Table S64: *Comparison of δG^{atom}(%) of the iminium phenyl fragment between* ***TS-3b****,* ***TS-4c****, and* ***TS-4d****.*

**TS-3b**		**TS-4c**		**TS-4d**	
#	δG^{atom}(%)	#	δG^{atom}(%)	#	δG^{atom}(%)
39 (C)	0.22%	38 (C)	0.97%	38 (C)	1.24%
40 (C)	0.36%	39 (C)	2.77%	39 (C)	3.11%
41 (C)	0.03%	40 (C)	0.55%	40 (C)	0.50%
43 (C)	0.01%	42 (C)	1.11%	42 (C)	0.88%
44 (C)	0.08%	43 (C)	4.76%	43 (C)	4.96%
45 (C)	0.01%	44 (C)	4.22%	44 (C)	3.68%
46 (H)	0.02%	45 (H)	0.08%	45 (H)	0.08%
47 (H)	0.00%	46 (H)	0.40%	46 (H)	0.32%
48 (H)	0.00%	47 (H)	4.59%	47 (H)	4.06%
49 (H)	0.60%	48 (H)	2.18%	48 (H)	2.81%
50 (H)	0.04%	49 (H)	3.92%	49 (H)	5.06%
**Σ**	**1.37%**	**Σ**	**25.55%**	**Σ**	**26.70%**

Table S65: *Comparison of δG^{atom}(%) of the western 2-isopropyl fragment (first arene) between* ***TS-3b****,* ***TS-4c****, and* ***TS-4d****.*

**TS-3b**		**TS-4c**		**TS-4d**	
#	δG^{atom}(%)	#	δG^{atom}(%)	#	δG^{atom}(%)
142 (C)	0.76%	141 (C)	0.93%	141 (C)	0.79%
148 (H)	0.63%	147 (H)	0.76%	147 (H)	0.61%
143 (C)	3.56%	142 (C)	3.48%	142 (C)	3.37%
144 (H)	1.35%	143 (H)	1.48%	143 (H)	1.69%
145 (H)	4.33%	144 (H)	4.65%	144 (H)	1.78%
146 (H)	2.88%	145 (H)	2.09%	145 (H)	4.37%
147 (C)	0.17%	146 (C)	0.18%	146 (C)	0.15%
149 (H)	0.15%	245 (H)	0.03%	245 (H)	0.02%
150 (H)	0.02%	246 (H)	0.06%	246 (H)	0.06%
151 (H)	0.05%	247 (H)	0.15%	247 (H)	0.10%
**Σ**	**13.90%**	**Σ**	**13.81%**	**Σ**	**12.94%**

Table S66: *Comparison of δG^{atom}(%) of the western 6-isopropyl fragment (first arene) between* ***TS-3b****,* ***TS-4c****, and* ***TS-4d****.*

**TS-3b**		**TS-4c**		**TS-4d**	
#	δG^{atom}(%)	#	δG^{atom}(%)	#	δG^{atom}(%)
152 (C)	0.01%	148 (C)	0.02%	148 (C)	0.02%
158 (H)	0.01%	154 (H)	0.01%	154 (H)	0.01%
153 (C)	0.00%	149 (C)	0.00%	149 (C)	0.00%
153 (H)	0.00%	150 (H)	0.00%	150 (H)	0.00%
155 (H)	0.00%	151 (H)	0.00%	151 (H)	0.00%
156 (H)	0.00%	152 (H)	0.00%	152 (H)	0.00%
157 (C)	0.05%	153 (C)	0.04%	153 (C)	0.04%
159 (H)	0.02%	155 (H)	0.02%	155 (H)	0.02%
160 (H)	0.01%	156 (H)	0.00%	156 (H)	0.00%
161 (H)	0.14%	157 (H)	0.09%	157 (H)	0.09%
**Σ**	**0.24%**	**Σ**	**0.18%**	**Σ**	**0.18%**

Table S67: Comparison of δG^{atom}(%) of the western 2-isopropyl fragment (second arene) between ***TS-3b****,* ***TS-4c****, and* ***TS-4d****.*

**TS-3b**		**TS-4c**		**TS-4d**	
#	δG^{atom}(%)	#	δG^{atom}(%)	#	δG^{atom}(%)
207 (C)	0.00%	203 (C)	0.13%	203 (C)	0.16%
213 (H)	0.00%	209 (H)	0.06%	209 (H)	0.06%
208 (C)	0.00%	204 (C)	0.01%	204 (C)	0.01%
209 (H)	0.00%	205 (H)	0.00%	205 (H)	0.00%
210 (H)	0.00%	206 (H)	0.00%	206 (H)	0.00%
211 (H)	0.00%	207 (H)	0.00%	207 (H)	0.00%
212 (C)	0.00%	208 (C)	0.15%	208 (C)	0.20%
214 (H)	0.00%	210 (H)	0.28%	210 (H)	0.01%
215 (H)	0.00%	211 (H)	0.01%	211 (H)	0.07%
216 (H)	0.00%	212 (H)	0.05%	212 (H)	0.36%
**Σ**	**0.00%**	**Σ**	**0.69%**	**Σ**	**0.87%**

Table S68: Comparison of δG^{atom}(%) of the western 4-isopropyl fragment (second arene) between ***TS-3b****,* ***TS-4c****, and* ***TS-4d****.*

**TS-3b**		**TS-4c**		**TS-4d**	
#	δG^{atom}(%)	#	δG^{atom}(%)	#	δG^{atom}(%)
227 (C)	0.00%	223 (C)	0.06%	223 (C)	0.14%
233 (H)	0.00%	229 (H)	0.01%	229 (H)	0.03%
228 (C)	0.00%	221 (C)	0.03%	221 (C)	0.03%
229 (H)	0.00%	225 (H)	0.01%	225 (H)	0.01%
230 (H)	0.00%	226 (H)	0.00%	226 (H)	0.00%
231 (H)	0.00%	227 (H)	0.00%	227 (H)	0.00%
232 (C)	0.00%	228 (C)	0.12%	228 (C)	0.24%
234 (H)	0.00%	230 (H)	0.16%	230 (H)	0.32%
235 (H)	0.00%	231 (H)	0.01%	231 (H)	0.02%
236 (H)	0.00%	232 (H)	0.09%	232 (H)	0.18%
**Σ**	**0.00%**	**Σ**	**0.49%**	**Σ**	**0.97%**

Table S69: *Comparison of δG^{atom}(%) of the western 6-isopropyl fragment (second arene) between **TS-3b**, **TS-4c**, and **TS-4d**.*

**TS-3b**		**TS-4c**		**TS-4d**	
#	δG^{atom}(%)	#	δG^{atom}(%)	#	δG^{atom}(%)
217 (C)	0.85%	213 (C)	1.05%	213 (C)	1.26%
223 (H)	0.90%	219 (H)	0.90%	219 (H)	1.04%
218 (C)	2.37%	214 (C)	1.84%	214 (C)	1.96%
219 (H)	0.52%	215 (H)	0.28%	215 (H)	1.01%
220 (H)	3.53%	216 (H)	1.09%	216 (H)	2.76%
221 (H)	1.61%	217 (H)	2.62%	217 (H)	0.28%
222 (C)	0.65%	218 (C)	0.12%	218 (C)	0.14%
224 (H)	1.26%	220 (H)	0.03%	220 (H)	0.05%
225 (H)	0.13%	221 (H)	0.03%	221 (H)	0.03%
226 (H)	0.14%	222 (H)	0.06%	222 (H)	0.03%
**Σ**	**11.96%**	**Σ**	**8.02%**	**Σ**	**8.56%**

Table S70: *Comparison of δG^{atom}(%) of the eastern 2-isopropyl fragment (first arene) between **TS-3b**, **TS-4c**, and **TS-4d**.*

**TS-3b**		**TS-4c**		**TS-4d**	
#	δG^{atom}(%)	#	δG^{atom}(%)	#	δG^{atom}(%)
113 (C)	0.89%	112 (C)	0.57%	112 (C)	0.54%
119 (H)	0.48%	118 (H)	0.12%	118 (H)	0.12%
114 (C)	2.13%	113 (C)	2.00%	113 (C)	2.05%
115 (H)	0.64%	114 (H)	1.55%	114 (H)	1.62%
116 (H)	3.36%	115 (H)	0.52%	115 (H)	0.55%
117 (H)	0.98%	116 (H)	2.37%	116 (H)	2.36%
118 (C)	0.09%	117 (C)	0.10%	117 (C)	0.10%
120 (H)	0.03%	119 (H)	0.02%	119 (H)	0.02%
121 (H)	0.04%	120 (H)	0.07%	120 (H)	0.02%
122 (H)	0.02%	121 (H)	0.02%	121 (H)	0.07%
**Σ**	**8.66%**	**Σ**	**7.34%**	**Σ**	**7.45%**

Table S71: *Comparison of δG^{atom}(%) of the eastern 6-isopropyl fragment (first arene) between* ***TS-3b****,* ***TS-4c****, and* ***TS-4d****.*

**TS-3b**		**TS-4c**		**TS-4d**	
#	δG^{atom}(%)	#	δG^{atom}(%)	#	δG^{atom}(%)
123 (C)	0.55%	122 (C)	0.54%	122 (C)	0.48%
129 (H)	0.35%	128 (H)	0.35%	128 (H)	0.30%
124 (C)	1.01%	123 (C)	0.82%	123 (C)	0.66%
125 (H)	0.31%	124 (H)	1.56%	124 (H)	0.18%
126 (H)	0.21%	125 (H)	0.24%	125 (H)	0.11%
127 (H)	1.95%	126 (H)	0.14%	126 (H)	1.28%
128 (C)	0.05%	127 (C)	0.05%	127 (C)	0.04%
130 (H)	0.01%	129 (H)	0.01%	129 (H)	0.01%
131 (H)	0.01%	130 (H)	0.01%	130 (H)	0.01%
132 (H)	0.01%	131 (H)	0.01%	131 (H)	0.01%
**Σ**	**4.46%**	**Σ**	**3.73%**	**Σ**	**3.08%**

Table S72: *Comparison of δG^{atom}(%) of the eastern 2-isopropyl fragment (second arene) between* ***TS-3b****,* ***TS-4c****, and* ***TS-4d****.*

**TS-3b**		**TS-4c**		**TS-4d**	
#	δG^{atom}(%)	#	δG^{atom}(%)	#	δG^{atom}(%)
172 (C)	0.01%	168 (C)	0.01%	168 (C)	0.01%
178 (H)	0.02%	174 (H)	0.02%	174 (H)	0.01%
173 (C)	0.00%	169 (C)	0.00%	169 (C)	0.00%
174 (H)	0.00%	170 (H)	0.00%	170 (H)	0.00%
175 (H)	0.00%	171 (H)	0.00%	171 (H)	0.00%
176 (H)	0.00%	172 (H)	0.00%	172 (H)	0.00%
177 (C)	0.01%	173 (C)	0.00%	173 (C)	0.00%
179 (H)	0.02%	175 (H)	0.00%	175 (H)	0.01%
180 (H)	0.00%	176 (H)	0.00%	176 (H)	0.00%
181 (H)	0.00%	177 (H)	0.01%	177 (H)	0.00%
**Σ**	**0.06%**	**Σ**	**0.04%**	**Σ**	**0.03%**

Table S73: *Comparison of δG^{atom}(%) of the eastern 4-isopropyl fragment (second arene) between* ***TS-3b****,* ***TS-4c****, and* ***TS-4d****.*

**TS-3b**		**TS-4c**		**TS-4d**	
#	δG^{atom}(%)	#	δG^{atom}(%)	#	δG^{atom}(%)
192 (C)	0.00%	188 (C)	0.00%	188 (C)	0.00%
198 (H)	0.00%	194 (H)	0.00%	194 (H)	0.00%
193 (C)	0.00%	189 (C)	0.00%	189 (C)	0.00%
194 (H)	0.00%	190 (H)	0.00%	190 (H)	0.00%
195 (H)	0.00%	191 (H)	0.00%	191 (H)	0.00%
196 (H)	0.00%	192 (H)	0.00%	192 (H)	0.00%
197 (C)	0.00%	193 (C)	0.00%	193 (C)	0.00%
199 (H)	0.00%	195 (H)	0.00%	195 (H)	0.00%
200 (H)	0.00%	196 (H)	0.00%	196 (H)	0.00%
201 (H)	0.00%	197 (H)	0.00%	197 (H)	0.00%
**Σ**	**0.00%**	**Σ**	**0.00%**	**Σ**	**0.00%**

Table S74: *Comparison of δG^{atom}(%) of the eastern 6-isopropyl fragment (second arene) between* ***TS-3b****,* ***TS-4c****, and* ***TS-4d****.*

**TS-3b**		**TS-4c**		**TS-4d**	
#	δG^{atom}(%)	#	δG^{atom}(%)	#	δG^{atom}(%)
182 (C)	1.91%	178 (C)	1.85%	178 (C)	1.70%
188 (H)	3.01%	184 (H)	2.79%	184 (H)	2.64%
183 (C)	1.07%	179 (C)	1.55%	179 (C)	1.18%
184 (H)	0.11%	180 (H)	0.59%	180 (H)	0.13%
185 (H)	1.52%	181 (H)	0.17%	181 (H)	1.73%
186 (H)	0.38%	182 (H)	2.33%	182 (H)	0.41%
187 (C)	2.63%	183 (C)	2.25%	183 (C)	2.30%
189 (H)	3.28%	185 (H)	0.26%	185 (H)	0.34%
190 (H)	0.28%	186 (H)	1.12%	186 (H)	1.37%
191 (H)	1.88%	187 (H)	3.23%	187 (H)	3.07%
**Σ**	**16.07%**	**Σ**	**16.14%**	**Σ**	**14.87%**

k. Improved System: Activation Energies

In order to investigate the activation energy computationally, we optimized the structure of the pre-transition state complex (**1d2a6a**).

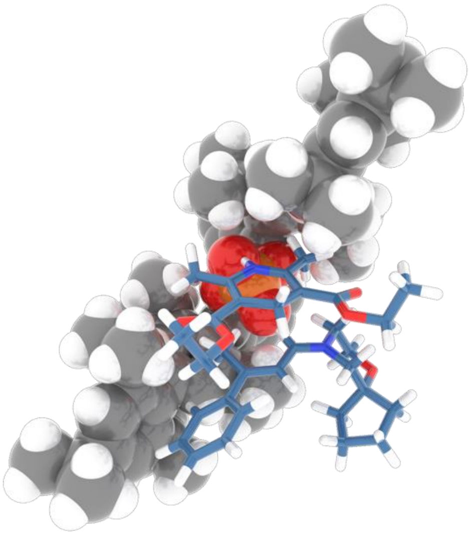

Figure S16: *Structure of the pre-transition state complex* ***1d2b6e****, obtained by relaxation of* ***TS-3b****.*

Table S75: *Obtained thermochemical corrections at the PBE-D3(BJ)/def2-SVP level (323 K, 1 M) as well as single point energies for the pre-transition state complex* ***1d2b6e****; Gibbs free energies were calculated with respect to* ***TS-3****.*

TC 1.921211795535	**E^{SP} / a.u.**	**ΔΔ*G*(323 K)**
B3LYP-D3(BJ)/def2-TZVPP	–5207.54815	11.0 kcal/mol
B3LYP/def2-TZVPP	–5206.85244	6.5 kcal/mol
ωB97M-V/def2-TZVPP	–5208.11048	14.7 kcal/mol

We additionally conducted an IGMH analysis with the B3LYP-D3(BJ)/def2-TZVPP densities.

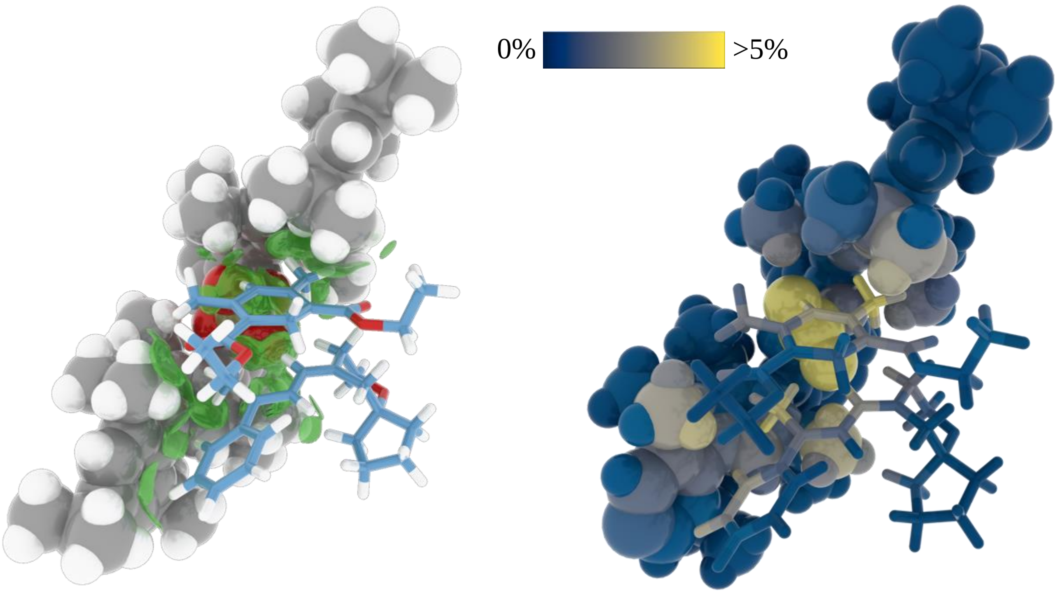

Figure S17: *δg^{inter} isosurface (left) and color-coded atoms according to their contribution to the overall interfragment interaction δG^{atom}(%) for pre-transition state complex* ***1d2b4e****.*

Table S76: *Comparison of δG^{atom}(%) of the Hantzsch ester's western methyl fragment between* ***1d2b6e*** *and* ***TS-3b****.*

**1d2b6e**		**TS-3b**	
#	#	#	δG^{atom}(%)
60 (C)	1.85%	60 (C)	2.38%
61 (H)	1.19%	61 (H)	1.09%
62 (C)	1.86%	62 (C)	1.40%
63 (H)	0.99%	63 (H)	2.47%
**Σ**	**5.89%**	**Σ**	**7.34%**

Table S77: *Comparison of δG^{atom}(%) of the Hantzsch ester's eastern methyl fragment between* ***1d2b6e*** *and* ***TS-3b****.*

**1d2b6e**		**TS-3b**	
#	δG^{atom}(%)	#	δG^{atom}(%)
64 (C)	6.31%	64 (C)	7.46%
65 (H)	3.00%	65 (H)	5.42%
66 (H)	6.50%	66 (H)	7.57%
67 (H)	3.65%	67 (H)	2.62%
**Σ**	**19.46%**	**Σ**	**23.07%**

Table S78: *Comparison of δG^{atom}(%) of the α-CH_2 morpholine fragment between* ***1d2b6e*** *and* ***TS-3b****.*

**1d2b6e**		**TS-3b**	
#	δG^{atom}(%)	#	δG^{atom}(%)
21 (C)	1.66%	21 (C)	2.69%
23 (H)	0.66%	23 (H)	1.66%
24 (H)	2.55%	24 (H)	4.00%
**Σ**	**4.87%**	**Σ**	**8.35%**

Table S79: *Comparison of δG^{atom}(%) of the spiro-cyclopentane morpholine fragment between* ***1d2b6e*** *and* ***TS-3b****.*

**1d2b6e**		**TS-3b**	
#	δG^{atom}(%)	#	δG^{atom}(%)
237 (C)	0.66%	237 (C)	0.65%
244 (H)	1.33%	244 (H)	1.05%
243 (H)	0.27%	243 (H)	0.42%
245 (H)	0.04%	245 (H)	0.04%
246 (H)	0.07%	246 (H)	0.03%
242 (C)	0.11%	242 (C)	0.08%
239 (C)	0.02%	239 (C)	0.02%
247 (H)	0.00%	247 (H)	0.00%
248 (H)	0.00%	248 (H)	0.01%
238 (C)	0.02%	238 (C)	0.03%
240 (H)	0.00%	240 (H)	0.01%
241 (H)	0.01%	241 (H)	0.01%
**Σ**	**2.53%**	**Σ**	**2.35%**

Table S80: *Comparison of δG^{atom}(%) of the iminium phenyl fragment between* ***1d2b6e*** *and* ***TS-3b****.*

**1d2b6e**		**TS-3b**	
#	δG^{atom}(%)	#	δG^{atom}(%)
39 (C)	0.98%	39 (C)	0.22%
40 (C)	3.00%	40 (C)	0.36%
41 (C)	0.21%	41 (C)	0.03%
43 (C)	0.14%	43 (C)	0.01%
44 (C)	2.41%	44 (C)	0.08%
45 (C)	0.55%	45 (C)	0.01%
46 (H)	0.03%	46 (H)	0.02%
47 (H)	0.01%	47 (H)	0.00%
48 (H)	0.27%	48 (H)	0.00%
49 (H)	3.84%	49 (H)	0.60%
50 (H)	3.09%	50 (H)	0.04%
**Σ**	**14.53%**	**Σ**	**1.37%**

Table S81: *Comparison of δG^{atom}(%) of the western 2-isopropyl fragment (first arene) between* ***1d2b6e*** *and* ***TS-3b****.*

**1d2b6e**		**TS-3b**	
#	δG^{atom}(%)	#	δG^{atom}(%)
142 (C)	0.64%	142 (C)	0.76%
148 (H)	0.23%	148 (H)	0.63%
143 (C)	3.52%	143 (C)	3.56%
144 (H)	2.10%	144 (H)	1.35%
145 (H)	3.82%	145 (H)	4.33%
146 (H)	2.21%	146 (H)	2.88%
147 (C)	0.14%	147 (C)	0.17%
149 (H)	0.07%	149 (H)	0.15%
150 (H)	0.02%	150 (H)	0.02%
151 (H)	0.07%	151 (H)	0.05%
**Σ**	**12.62%**	**Σ**	**13.90%**

Table S82: *Comparison of δG^{atom}(%) of the western 6-isopropyl fragment (first arene) between* ***1d2b6e*** *and* ***TS-3b****.*

**1d2b6e**		**TS-3b**	
#	δG^{atom}(%)	#	δG^{atom}(%)
152 (C)	0.05%	152 (C)	0.01%
158 (H)	0.01%	158 (H)	0.01%
153 (C)	0.00%	153 (C)	0.00%
153 (H)	0.00%	153 (H)	0.00%
155 (H)	0.00%	155 (H)	0.00%
156 (H)	0.00%	156 (H)	0.00%
157 (C)	0.11%	157 (C)	0.05%
159 (H)	0.07%	159 (H)	0.02%
160 (H)	0.01%	160 (H)	0.01%
161 (H)	0.24%	161 (H)	0.14%
**Σ**	**0.49%**	**Σ**	**0.24%**

Table S83: *Comparison of δG^{atom}(%) of the western 2-isopropyl fragment (second arene) between* ***1d2b6e*** *and* ***TS-3b****.*

**1d2b6e**		**TS-3b**	
#	δG^{atom}(%)	#	δG^{atom}(%)
207 (C)	0.11%	207 (C)	0.00%
213 (H)	0.05%	213 (H)	0.00%
208 (C)	0.01%	208 (C)	0.00%
209 (H)	0.00%	209 (H)	0.00%
210 (H)	0.00%	210 (H)	0.00%
211 (H)	0.00%	211 (H)	0.00%
212 (C)	0.11%	212 (C)	0.00%
214 (H)	0.04%	214 (H)	0.00%
215 (H)	0.17%	215 (H)	0.00%
216 (H)	0.01%	216 (H)	0.00%
**Σ**	**0.50%**	**Σ**	**0.00%**

Table S84: *Comparison of δG^{atom}(%) of the western 4-isopropyl fragment (second arene) between* ***1d2b6e*** *and* ***TS-3b****.*

**1d2b6e**		**TS-3b**	
#	δG^{atom}(%)	#	δG^{atom}(%)
227 (C)	0.29%	227 (C)	0.00%
233 (H)	0.06%	233 (H)	0.00%
228 (C)	0.04%	228 (C)	0.00%
229 (H)	0.02%	229 (H)	0.00%
230 (H)	0.01%	230 (H)	0.00%
231 (H)	0.01%	231 (H)	0.00%
232 (C)	0.75%	232 (C)	0.00%
234 (H)	0.62%	234 (H)	0.00%
235 (H)	0.87%	235 (H)	0.00%
236 (H)	0.09%	236 (H)	0.00%
**Σ**	**2.76%**	**Σ**	**0.00%**

Table S85: *Comparison of δG^{atom}(%) of the western 6-isopropyl fragment (second arene) between* ***1d2b6e*** *and* ***TS-3b****.*

**1d2b6e**		**TS-3b**	
#	δG^{atom}(%)	#	δG^{atom}(%)
217 (C)	1.45%	217 (C)	0.85%
223 (H)	1.38%	223 (H)	0.90%
218 (C)	3.01%	218 (C)	2.37%
219 (H)	0.56%	219 (H)	0.52%
220 (H)	2.01%	220 (H)	3.53%
221 (H)	3.98%	221 (H)	1.61%
222 (C)	0.19%	222 (C)	0.65%
224 (H)	0.13%	224 (H)	1.26%
225 (H)	0.04%	225 (H)	0.13%
226 (H)	0.04%	226 (H)	0.14%
**Σ**	**12.79%**	**Σ**	**11.96%**

Table S86: *Comparison of δG^{atom}(%) of the eastern 2-isopropyl fragment (first arene) between* ***1d2b6e*** *and* ***TS-3b****.*

**1d2b6e**		**TS-3b**	
#	δG^{atom}(%)	#	δG^{atom}(%)
113 (C)	0.42%	113 (C)	0.89%
119 (H)	0.11%	119 (H)	0.48%
114 (C)	1.35%	114 (C)	2.13%
115 (H)	0.30%	115 (H)	0.64%
116 (H)	1.88%	116 (H)	3.36%
117 (H)	0.91%	117 (H)	0.98%
118 (C)	0.06%	118 (C)	0.09%
120 (H)	0.03%	120 (H)	0.03%
121 (H)	0.01%	121 (H)	0.04%
122 (H)	0.01%	122 (H)	0.02%
**Σ**	**5.08%**	**Σ**	**8.66%**

__Table S87:__ Comparison of δG^{atom}(%) of the eastern 6-isopropyl fragment (first arene) between __1d2b6e__ and __TS-3b__.

**1d2b6e**		**TS-3b**	
#	δG^{atom}(%)	#	δG^{atom}(%)
123 (C)	0.47%	123 (C)	0.55%
129 (H)	0.26%	129 (H)	0.35%
124 (C)	1.03%	124 (C)	1.01%
125 (H)	0.34%	125 (H)	0.31%
126 (H)	0.21%	126 (H)	0.21%
127 (H)	1.98%	127 (H)	1.95%
128 (C)	0.04%	128 (C)	0.05%
130 (H)	0.01%	130 (H)	0.01%
131 (H)	0.01%	131 (H)	0.01%
132 (H)	0.01%	132 (H)	0.01%
**Σ**	**4.36%**	**Σ**	**4.46%**

__Table S88:__ Comparison of δG^{atom}(%) of the eastern 2-isopropyl fragment (second arene) between __1d2b6e__ and __TS-3b__.

**1d2b6e**		**TS-3b**	
#	δG^{atom}(%)	#	δG^{atom}(%)
172 (C)	0.01%	172 (C)	0.01%
178 (H)	0.02%	178 (H)	0.02%
173 (C)	0.00%	173 (C)	0.00%
174 (H)	0.00%	174 (H)	0.00%
175 (H)	0.00%	175 (H)	0.00%
176 (H)	0.00%	176 (H)	0.00%
177 (C)	0.00%	177 (C)	0.01%
179 (H)	0.01%	179 (H)	0.02%
180 (H)	0.00%	180 (H)	0.00%
181 (H)	0.00%	181 (H)	0.00%
**Σ**	**0.04%**	**Σ**	**0.06%**

*Table S89: Comparison of δG^{atom}(%) of the eastern 4-isopropyl fragment (second arene) between **1d2b6e** and **TS-3b**.*

**1d2b6e**		**TS-3b**	
#	δG^{atom}(%)	#	δG^{atom}(%)
192 (C)	0.00%	192 (C)	0.00%
198 (H)	0.00%	198 (H)	0.00%
193 (C)	0.00%	193 (C)	0.00%
194 (H)	0.00%	194 (H)	0.00%
195 (H)	0.00%	195 (H)	0.00%
196 (H)	0.00%	196 (H)	0.00%
197 (C)	0.00%	197 (C)	0.00%
199 (H)	0.00%	199 (H)	0.00%
200 (H)	0.00%	200 (H)	0.00%
201 (H)	0.00%	201 (H)	0.00%
**Σ**	**0.00%**	**Σ**	**0.00%**

*Table S90: Comparison of δG^{atom}(%) of the eastern 6-isopropyl fragment (second arene) between **1d2b6e** and **TS-3b**.*

**1d2b6e**		**TS-3b**	
#	δG^{atom}(%)	#	δG^{atom}(%)
182 (C)	1.53%	182 (C)	1.91%
188 (H)	2.41%	188 (H)	3.01%
183 (C)	0.68%	183 (C)	1.07%
184 (H)	0.08%	184 (H)	0.11%
185 (H)	0.86%	185 (H)	1.52%
186 (H)	0.20%	186 (H)	0.38%
187 (C)	2.78%	187 (C)	2.63%
189 (H)	3.43%	189 (H)	3.28%
190 (H)	0.49%	190 (H)	0.28%
191 (H)	2.03%	191 (H)	1.88%
**Σ**	**14.49%**	**Σ**	**16.07%**

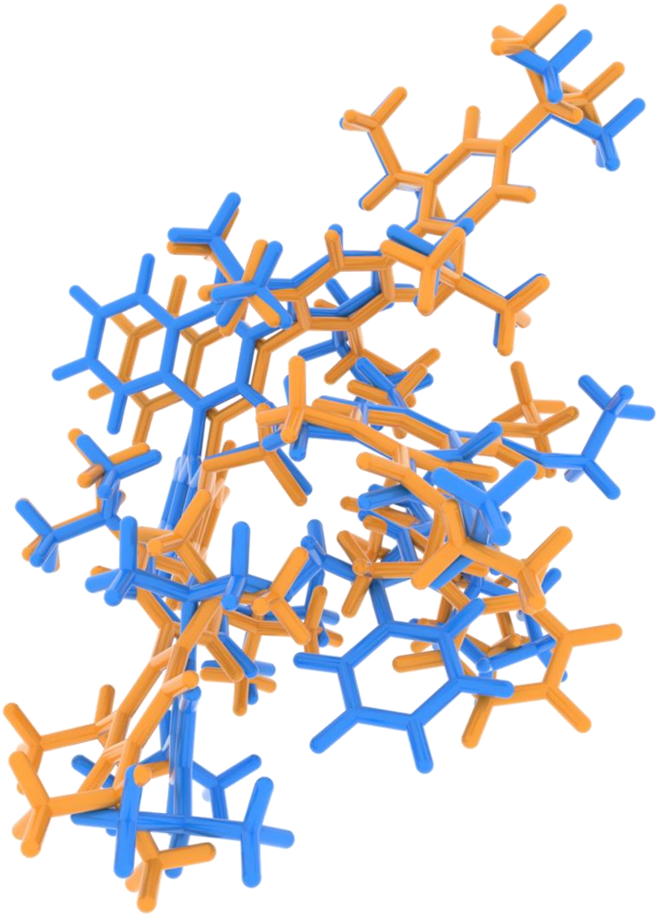

Figure S18: *Structural comparison between* ***1d2b6e*** *(blue) and* ***TS-3b*** *(orange).*

The structural overlay of both **1d2b6e** and **TS-3b** indicates a substantial difference, most of which is being caused by a closer orientation of the phenyl fragment to the phosphate anion. This eventually leads to the calculated difference in activation energies with and without dispersion correction (11.0 kcal/mol vs. 6.5 kcal/mol), and is further supported by the larger contribution to the interfragment interaction as calculated by the IGMH analysis. These effects are expected to be transposable to the *endo*-rotated **TS-3**.

XYZ Coordinates

TS-1

54 conformers were generated by CREST, all of which were used for constrained DFT optimizations followed by transition state and frequency calculations.

Electronic energy (a. u.) | DLPNO-CCSD(T)/cc-pVTZ: –4111.23553028788
Thermochemical Corrections (a. u.) | PBE-D3(BJ)/def2-SVP: 1.377326615848
Imaginary Frequencies: one (–587.05 cm^{-1})
Gibbs Free Energy (a. u.): –4116.415342903240

```
182

  C   3.66916939159229     -2.16779132812554      0.46704356259096
  C   5.51422098630449     -1.21005863490217     -1.03211868577402
  C   6.13635157519248     -0.04700797259461     -1.61606009461278
  C   5.51591875492624      1.22651969520515     -1.46950747607233
  C   4.28569774464126      1.37653566500592     -0.84296290083396
  C   3.67391201708912      0.20806989394791     -0.28497381135580
  H   6.02789248153986      2.11341677147031     -1.87200616162969
  C   4.40245995709047     -2.88115955657019      1.48368279575297
  C   2.33312756539934     -2.51805923345353      0.23403386371157
  C   3.78499634013339     -4.01379949032619      2.12944103647421
  C   2.45022331334453     -4.36939284209497      1.78737883768940
  C   1.70153413853831     -3.62675403648145      0.88363962882473
  H   1.99131245093996     -5.24290839856985      2.27561071336398
  O   2.45734895029572      0.35563507587984      0.35001256331843
  O   1.60260255944772     -1.82510643947587     -0.70512878850483
  P   1.09349736235602     -0.25194383720057     -0.41981942394066
  O  -0.03112089343170     -0.17315443415070      0.59157501177372
  O   0.95951578079090      0.35945982521705     -1.81530977838032
  N  -1.73693561008059      2.39763767506177      2.57772883353844
  C  -2.37570308051657      1.48425800132703      1.81555214927576
  C  -0.28982705157165      2.31043100862385      2.79700388726562
  C  -0.03193732864340      2.01314084951569      4.27737399779960
  H   0.17680773894732      3.28348977498643      2.52758936683118
  H   0.12883411020209      1.52008644023830      2.14175010816290
  O  -0.64672400963202      2.98349236007490      5.11527353760768
  H   1.05273369952982      2.03972564708348      4.49392192404463
  H  -0.41576797278255      0.99070997187260      4.51531936106720
  C  -2.04648984085980      3.02310911780157      4.91530233043615
  C  -2.39201740233740      3.35760138865356      3.45761741399446
  H  -3.48506860057064      3.34293723368426      3.29578694897906
  H  -2.02190157004319      4.37384855523291      3.20356673966694
  C  -3.74740566327703      1.30553107745461      1.67884953710384
  H  -1.67465541538254      0.82719229278678      1.27012714459380
  C  -4.32631817437380      0.33455348620613      0.77694536752064
  C  -3.47705661622335     -0.83199898324525      0.31366258785535
  H  -3.89955884011335     -1.29838410248180     -0.59588864152838
  H  -3.43629548410133     -1.61599981704013      1.09425050986369
  H  -2.42729623088201     -0.55723899949941      0.10084274622395
  C  -5.80028748277270      0.06985139881692      0.92091606870706
  C  -6.71290896621549      1.14652522228262      0.99370054478498
  C  -6.31567064127169     -1.24410233666306      0.93659712072522
  H  -4.44677980430233      1.94904759833716      2.23254707317600
  C  -7.69565772640002     -1.47365472433783      1.04397072717693
  C  -8.09095657402840      0.91776982147364      1.09372720224915
```

C	-8.59036107992030	-0.39558505073217	1.12297054517368
H	-5.63225911779755	-2.10271386630522	0.87300827479650
H	-8.07332959217966	-2.50747676391754	1.06471965338164
H	-9.67304068594082	-0.57653852888479	1.20198192842148
H	-6.33045949000923	2.17653956682858	0.92819675441020
H	-8.78408918804877	1.77187138928458	1.13898682366252
H	-2.51245640889745	2.04111009487886	5.18131914501599
H	-2.45153922506060	3.79873585388862	5.59627546834835
N	-1.49581181408165	1.27142529354855	-2.17364440996369
C	-2.44498640810865	0.51315092476582	-2.81963843249766
C	-3.78576430972647	0.73807030209701	-2.51813957975505
C	-4.12937633393170	1.72326736856341	-1.49433700335351
C	-3.08494088627107	2.63226188852330	-1.04278802745192
H	-4.36890911278864	1.01880607973443	-0.39353486077374
H	-5.14953914041179	2.13533600151422	-1.54144687005327
C	-1.75332179879941	2.31885453457289	-1.33848786176926
H	-0.47483081974002	0.92636075113551	-2.17797344408564
C	-1.93260360553234	-0.54662529662517	-3.77119676761976
C	-0.56274397464319	3.07150438044071	-0.84273581431311
H	0.37465193684911	2.55626715341577	-1.10864003561990
H	-0.62127907512102	3.22326468822521	0.24793652055569
H	-0.55032652317815	4.08869887126249	-1.28083809213398
C	-3.41953140704677	3.79567206185447	-0.20187845275876
O	-2.63868366688041	4.58888997332807	0.31166950000392
O	-4.77763438926652	3.92619179592516	-0.04946327836706
C	-5.19080643963190	5.04415381771702	0.73717502354229
C	-4.88123534438619	-0.11444531914712	-3.05068304845986
O	-6.03068889146736	0.12822158516512	-2.35707978781148
C	-7.14336059157068	-0.70961795239300	-2.67856890888599
O	-4.82316532319750	-0.96145338077656	-3.93154383003256
H	-4.77227491523196	5.98939958660282	0.33754672384420
H	-4.85071148596561	4.93899978985889	1.78871334638116
H	-7.95209926553403	-0.41343541663017	-1.98728209429842
H	-6.88337059570558	-1.77709983021209	-2.52802314692768
C	7.35968187855267	-0.19779902619055	-2.33320686715776
C	6.12826680096771	-2.48096277456097	-1.23961008651041
C	7.31084947818726	-2.59589768708327	-1.95626185791574
C	7.94139707547342	-1.44527863378440	-2.49888757241773
H	5.64382383877287	-3.37835735861655	-0.83074959079475
H	7.76036175301172	-3.58873446306757	-2.10955678952357
H	8.88251389733876	-1.54780108417538	-3.06003878047444
H	7.82781541154834	0.70052636731037	-2.76540284733598
C	4.28812469265563	-1.04901551795350	-0.29263663833919
C	4.51359414508461	-4.73677472126725	3.11966299467987
C	5.70793744993506	-2.49331547374261	1.91131032019441
C	6.38425717809053	-3.20817549432186	2.88954976259163
C	5.79139615946979	-4.34916505990969	3.49150163284710
H	4.03085453551000	-5.60496472589750	3.59531154249884
H	6.34021444623713	-4.91270054006730	4.26112447028842
H	6.17524333268969	-1.60662145523950	1.46183574194908
H	7.38745075710391	-2.88313857571200	3.20469287887240
C	3.62510118578457	2.70650084676279	-0.66328299923226
C	3.60571204051371	3.29083874620192	0.63273452368379
C	2.92605240139668	4.50532932131746	0.82412012465090
C	2.28751210419530	5.17709271809837	-0.23284823920445
C	2.36243492167106	4.60380847628320	-1.51254103180329
C	3.00420975747201	3.37353743367068	-1.75332764854662
H	1.87612293403975	5.12715122644201	-2.35115171051162
H	2.90702796927510	4.94779557958532	1.83345357915403

C	4.34418760134618	2.65896471080787	1.80985347088685
C	3.39185792946389	2.24974697618864	2.94347537419015
H	2.86728922215794	3.13267074937384	3.36588714881979
H	3.95245807279786	1.76885899155710	3.77160654694162
H	2.63653826162866	1.52987130345391	2.57372981030649
C	5.46306199834172	3.58470835576737	2.31857159853581
H	4.83225801106590	1.73426507095629	1.44279956473160
H	6.16411296623420	3.85395478649977	1.50319272775008
H	6.04423949860271	3.08997250113453	3.12394678321564
H	5.05305279036648	4.52901988005374	2.73381063725958
C	3.04196656119741	2.79513803821200	-3.16577790642933
C	1.71519977758755	2.93772251598820	-3.92566778656343
H	1.77095963471436	2.39827536660724	-4.89292123787343
H	1.47605259248563	3.99668276505293	-4.15679920553425
H	0.87590363741373	2.50569616675567	-3.35212028007592
C	4.18753914326781	3.42531184005497	-3.97998181585578
H	3.24154753300730	1.70924021404366	-3.06314749323812
H	5.16986343473902	3.28729129924533	-3.48820299531191
H	4.02724009157165	4.51730643965711	-4.10108585751951
H	4.24600770048836	2.97512982304421	-4.99258219993832
C	1.56441010919590	6.49763466030960	-0.00218250632206
C	0.27472866278745	-3.95320508765501	0.58117162703385
C	-0.73781091272151	-3.64924867846960	1.52826191214087
C	-0.06096918449358	-4.57166192426121	-0.65308951347919
C	-2.07089376504993	-3.96986779092204	1.21133295276475
C	-1.41241443756842	-4.83907861129331	-0.93541445299652
C	-2.43635971380246	-4.54662088911591	-0.01582035387525
H	-2.86053116348428	-3.76588848608105	1.95346982862956
H	-1.66918095348398	-5.31430887371825	-1.89607410968474
C	-0.41059336686588	-3.03978468373110	2.88920668836908
C	-1.27424797750469	-1.81512785929783	3.22215620701525
H	-0.95859901776090	-1.37876710093288	4.19291458185543
H	-2.35023374991823	-2.07097976029287	3.31829356116568
H	-1.15613420365884	-1.04873055061946	2.43480782069984
C	-0.50183135110081	-4.10554054817308	3.99572116760106
H	0.63917478622929	-2.68643019773191	2.84540136976747
H	-0.22048675295245	-3.67793988683618	4.98041998963352
H	0.16712994975785	-4.96554017959802	3.79219605758721
H	-1.53562588060472	-4.50142463976674	4.08056378699999
C	1.02588449872249	-5.02697594579733	-1.62464439834314
C	1.02054522369601	-6.56008708214440	-1.75890040923433
H	0.07965321899141	-6.92204478490676	-2.22374870284145
H	1.11506517622056	-7.05066281228383	-0.76919848169022
H	1.86203787137928	-6.90213126575078	-2.39616264299583
C	0.94609344002088	-4.33990138159262	-2.99533640365481
H	2.00113334892301	-4.74490420081734	-1.17904978870756
H	-0.00294871643490	-4.57990249389468	-3.51903704616542
H	1.77773375857647	-4.68032557457710	-3.64616308787494
H	1.01923527509543	-3.24108936488223	-2.88978644358573
C	-3.89019029305108	-4.91158247416602	-0.29365255115089
C	2.55823300605877	7.61465845760809	0.36249738333144
H	3.34801148761903	7.71947603240908	-0.40808716325993
H	3.06113431626270	7.40046483572698	1.32874711710070
H	2.04022740459838	8.59087312069837	0.46348195469813
C	0.44908558173609	6.37949524439905	1.05166880482032
H	1.08720901711265	6.77611116915922	-0.96777553215087
H	0.86069609941629	6.07098714469286	2.03579770851095
H	-0.32764824101802	5.64716925160549	0.75667506152177
H	-0.05304946082675	7.35800819221118	1.19930242873894

C	-4.42518545062386	-4.32549461432008	-1.60928325429390
H	-5.48709794184792	-4.60815406376185	-1.76304412499256
H	-3.85436342806850	-4.69689605246963	-2.48467591113751
H	-4.36448412391800	-3.21951425004347	-1.62314494698091
C	-4.08369249003600	-6.43828781053797	-0.24889944801101
H	-4.49070067225979	-4.47658778129348	0.53717578896714
H	-3.50587535087821	-6.93302535105859	-1.05705300256111
H	-5.15130517587886	-6.71147459642421	-0.38086272054651
H	-3.73402977308328	-6.85778164415601	0.71538389019989
H	-7.45129757334798	-0.57155150396347	-3.73463228116287
H	-6.29477114606098	5.05940513534504	0.69464892676575
C	-0.69327105877810	-0.11367815635937	-4.56911451185941
H	-0.47586677621097	-0.88217600452691	-5.33716624909936
H	0.20515007371111	-0.02181058408353	-3.92781673791395
H	-0.85776073025563	0.84959834544973	-5.09319416740199
C	-1.68981093150785	-1.85870107985935	-2.99917844305751
H	-2.76460061275491	-0.73979453542195	-4.47230440366316
H	-2.60701601322616	-2.20018882893789	-2.48661215514117
H	-0.88576179387039	-1.75707983100763	-2.24485399457714
H	-1.38788869892172	-2.65462758915138	-3.70644258326858

TS-2

385 conformers were generated by CREST, all of which were used for constrained optimizations at the GFN2-xTB level of theory. Subsequently, the number of conformers was reduced to 191 by RMSD calculation and cutoff following RMSD ≥ 0.20 Å^2. After constrained DFT optimizations, the number of conformers was again reduced from 191 to 143 by RMSD ≥ 0.25 Å^2. All 143 conformers were subjected to transition state optimizations and frequency calculations.

Electronic energy (a. u.) | DLPNO-CCSD(T)/cc-pVTZ: –4111.23227588186
Thermochemical Corrections (a. u.) | PBE-D3(BJ)/def2-SVP: 1.378167177460
Imaginary Frequencies: one (–664.24 cm^{-1})
Gibbs Free Energy (a. u.): –4109.854108704400

182

C	3.12757822667729	-2.07516375554245	0.94051806765247
C	5.21838461541356	-1.28807449910797	-0.26623472480098
C	5.93605597141385	-0.23659188552705	-0.94030375504962
C	5.27100862402927	0.98939222884652	-1.20850418628551
C	3.93314256120608	1.19951011368444	-0.89007188517950
C	3.23576426127698	0.15701042053297	-0.19842645912107
H	5.82726619120903	1.79357788988848	-1.71411799499328
C	3.65497922160241	-2.62275467540376	2.16335366610159
C	1.90914544533508	-2.56501928040315	0.45505917537864
C	3.01284914440342	-3.77489266998886	2.74570504276077
C	1.83963499522027	-4.29919115361030	2.13524457650179
C	1.24057281455894	-3.69018065228212	1.03635490332787
H	1.36626728052412	-5.19027150124514	2.57638211715566
O	1.92447364059415	0.37017244074491	0.17772687606433
O	1.36491802949214	-1.99310988052965	-0.67286861739785
P	0.66336813554519	-0.47819227645945	-0.57609781287953
O	-0.50176080570269	-0.44834877254492	0.39526763955598
O	0.49506742135663	-0.00851927207667	-2.01651475903366
N	-0.87321708268768	2.19869838469389	3.41018398381380
C	-2.18026753418980	2.10719552402814	3.08326554151266
C	-0.39761134765470	3.15005936219615	4.40827138640279
C	0.56076070101289	2.44898709252533	5.37944753469737

H	-1.26379602195565	3.57368361739773	4.95491011617920
H	0.13494321819847	3.98207050929323	3.89607131191730
O	1.62587858134404	1.82111041050906	4.69373831648808
H	-0.01370999687774	1.69992745818465	5.98091682338602
H	1.00747519955382	3.18828247261969	6.07435631477560
C	1.13449444895459	0.84221946037841	3.78071211936886
C	0.19923300676400	1.45942974476015	2.74482096391053
H	0.77254075833483	2.17314381128497	2.11793993657166
H	-0.19963688880511	0.68116265237906	2.06676445603848
C	-2.75718810558176	1.26130887484463	2.15166854760188
H	-2.81780225268201	2.79216974398807	3.66580214639534
C	-4.17277545763603	1.24971955590346	1.83649087269765
C	-5.11891084131353	2.09227914378591	2.67803409411630
H	-5.35990002730583	1.58680587421043	3.63663220238507
H	-6.06737531625105	2.27879354195496	2.14040182163899
H	-4.68441609625184	3.08277130259300	2.90178845659957
C	-4.74107120137576	0.00341133001619	1.22812082857926
C	-3.94171741433026	-0.84017606730238	0.41866278793229
C	-6.11173356573364	-0.32238152327090	1.36806909152502
H	-2.09927382042559	0.57958912387533	1.59428295030263
C	-6.66462092064178	-1.43454900793812	0.72078070985245
C	-4.49996944958397	-1.94897406082291	-0.23164991115788
C	-5.86241129122640	-2.24735042731652	-0.09643994296270
H	-6.76371759966654	0.30159349843434	1.99391562729453
H	-7.73204262415864	-1.66759319866385	0.85624681856935
H	-6.29579500270737	-3.11149537332059	-0.62221564649298
H	-2.86449372265264	-0.65032543299818	0.29549322373685
H	-3.85056683780376	-2.58755792378385	-0.84452776729710
H	2.01070080440007	0.39468129702967	3.27287509609882
H	0.59986473765585	0.03308380964376	4.33709269675481
N	-1.86359971002173	1.37174881659414	-1.78577725318857
C	-3.13046696749110	0.93890325316921	-2.08154869086378
C	-4.20817981620465	1.61447560734891	-1.49745505199857
C	-3.95688467681062	2.61646102989669	-0.46810730677561
C	-2.58644966542899	3.08099561720987	-0.29128833604838
H	-4.18320458754373	1.96959705368088	0.67789327549892
H	-4.75167275750627	3.36159798139978	-0.30813694815335
C	-1.55583026868411	2.42713607376595	-0.96748926788965
H	-1.02106866346568	0.79548241596996	-2.08643552449577
C	-3.25726507994485	-0.19798231859627	-3.07551351819880
C	-0.12051891233276	2.81467063120469	-0.89972228473066
H	0.32887621667729	2.46742344981173	0.04953055981078
H	-0.01203855897932	3.91045084566905	-0.89886552886486
H	0.45844909238774	2.36569347321062	-1.72148954117028
C	-2.29680825050733	4.17778609181850	0.65468830036842
O	-1.19988294598503	4.60120537116032	1.00125349938834
O	-3.45888641019982	4.71115610015849	1.15302167516305
C	-3.27955342673256	5.77409406644083	2.08854436139107
C	-5.61822475352835	1.26600833456462	-1.79941282923088
O	-6.46980098720929	1.93805601973914	-0.96642069455629
C	-7.85454891967743	1.63782592133589	-1.15243989188122
O	-6.03119988038529	0.50219736716552	-2.66079551947964
H	-2.70727806329194	6.61032094768486	1.63856077904218
H	-2.72264902370133	5.42708584086304	2.98419102214722
H	-8.40755790400879	2.30431434157486	-0.46645604489079
H	-8.05460086229856	0.57576273681302	-0.90401172961523
C	7.28444399211661	-0.45556194436081	-1.35059257299094
C	5.87658101222062	-2.54321866409913	-0.09205674314212
C	7.18347142736365	-2.73199305335118	-0.51742413517046

C	7.90319398053894	-1.67716000428835	-1.13883652565938
H	5.33070586359452	-3.37321021090408	0.37564646318321
H	7.66371636933197	-3.71234298085477	-0.37740736203694
H	8.94191637486561	-1.83770877902066	-1.46498162013812
H	7.81844820142129	0.36502089662233	-1.85492827954275
C	3.86447732516810	-1.04320305416306	0.16626418828287
C	3.55190893850484	-4.34371244007123	3.93779661330740
C	4.77905221901987	-2.05981576939826	2.83827520016578
C	5.26635092993996	-2.62453963801799	4.00828806418025
C	4.65897526677404	-3.78490520039576	4.55758477942589
H	3.05783782974493	-5.23006387141441	4.36602576204060
H	5.05954047358324	-4.22938250896324	5.48123288892145
H	5.25401024667453	-1.16027607266810	2.42206715627370
H	6.12854572119954	-2.16680542701738	4.51654855507869
C	3.30083583086468	2.49674280534167	-1.28925242670272
C	3.10235864283727	3.52817652600429	-0.32932953238667
C	2.63281022418444	4.77829939049579	-0.76407415600690
C	2.33982022054443	5.03851973299147	-2.11503385553090
C	2.51965314604781	3.99844726320133	-3.03794452471574
C	2.99123779405566	2.72700949566579	-2.65608499478336
H	2.27525293315509	4.18357066988747	-4.09657705057784
H	2.47162767751052	5.57262241144749	-0.02145610830136
C	3.41233615484166	3.29265148774402	1.14709370892687
C	2.60175888979844	4.18883839921876	2.09658342475936
H	2.66828156555156	3.79453306340743	3.13014778626470
H	1.53114463498272	4.23454449128381	1.80963868396341
H	2.98685095715374	5.22984574871618	2.10519890560291
C	4.91806177063466	3.43793922713442	1.43639267112548
H	3.13058019372247	2.24078111462140	1.36548442295468
H	5.25652624115849	4.47014473694597	1.20840329770432
H	5.52237332053507	2.73794501372443	0.82786270077888
H	5.13192089667658	3.23457427903251	2.50601639773086
C	3.16346614962773	1.64283844448966	-3.71800451731944
C	1.90914107450675	1.46337940859696	-4.58836390392221
H	1.03895333199995	1.19800516266447	-3.95939841547609
H	2.06451578178989	0.63714894717345	-5.31215647504604
H	1.67430098804573	2.37528505881887	-5.17591221405519
C	4.40320376320090	1.91896899530502	-4.58722507340200
H	3.32346141277879	0.68322883502966	-3.18683709073880
H	5.32544170574381	1.98519679668182	-3.97683104157280
H	4.29635183321207	2.87777586616702	-5.13674589248520
H	4.54714461902094	1.11299334096081	-5.33610221797110
C	1.76893721491826	6.38055048703770	-2.55306061926159
C	-0.07733128520545	-4.15468811596315	0.51219805238064
C	-1.22424221823158	-4.10128336392517	1.35593605870413
C	-0.19938308830419	-4.64701135514634	-0.81675995039777
C	-2.46011303326959	-4.53700091987465	0.84482209836164
C	-1.46058207651432	-5.06471310839798	-1.27696112336064
C	-2.60587279139904	-5.02079450734892	-0.46547670560725
H	-3.35374936728074	-4.48081583667587	1.48771176542452
H	-1.54192099165012	-5.45105341851675	-2.30477227776989
C	-1.17883748747525	-3.55852090755604	2.78355532207678
C	-1.99129141828703	-2.26191634379162	2.91565926209239
H	-1.62146164299941	-1.51695616764286	2.18713434466251
H	-1.89169572271495	-1.83779144625014	3.93727342721390
H	-3.07206708386597	-2.42261584695850	2.72260596231950
C	-1.61005381602169	-4.61699237206770	3.81092306657532
H	-0.12867439192061	-3.29185072429486	3.00831768667712
H	-0.98983106766815	-5.53377297491421	3.73670071351956

H	-2.66796751551777	-4.91979483245483	3.66635396449962
H	-1.51587407006053	-4.22109987243345	4.84334019560263
C	1.00344757935713	-4.81204874024169	-1.74157206439594
C	1.29900587372134	-6.30386314786518	-1.98148482511318
H	2.20905213446656	-6.42785043239541	-2.60421399659873
H	0.45982919749155	-6.80270416467938	-2.50986134755090
H	1.45938261550873	-6.84162196678544	-1.02521225685689
C	0.83300818203335	-4.04864887416841	-3.06490978938244
H	1.88878620643438	-4.38757240571218	-1.22905502253898
H	1.74020244345868	-4.15928399981247	-3.69389881253861
H	0.67511680301070	-2.96932443148224	-2.87929893841775
H	-0.02558830988741	-4.43604355229670	-3.65252233649684
C	-3.95599670010215	-5.53269705184148	-0.95097403319545
C	2.57802127999068	7.57299250986868	-2.02132067060553
H	2.18721066879635	8.52732424629723	-2.43011935877273
H	3.64829054801936	7.49065888163287	-2.29715167614745
H	2.51977086010490	7.64179593726751	-0.91525631029843
C	0.28412897546929	6.49461546640168	-2.15795621621380
H	1.82261832913290	6.40496923763374	-3.66382009637474
H	-0.14475517290247	7.46207385882030	-2.49276454737106
H	0.15795098990800	6.42700927906185	-1.05695461017584
H	-0.31440525190066	5.67919902857980	-2.61138457668662
C	-4.28751339380669	-5.11193913840151	-2.39043217044490
H	-4.20752943162210	-4.01460570659617	-2.52783318353901
H	-5.31888443901986	-5.41717758191560	-2.66125196887858
H	-3.60434120881729	-5.58921910926923	-3.12273109913759
C	-4.03959994374263	-7.06182019607549	-0.78966394297521
H	-4.72319971911573	-5.08469273602130	-0.28127121881928
H	-3.84774158907160	-7.36470291838119	0.25890248845557
H	-3.28151764541058	-7.56353009211935	-1.42659759907573
H	-5.03976904066331	-7.44264938789444	-1.08422159094610
H	-8.16229346980222	1.81500358876556	-2.20232517774149
H	-4.29344352642524	6.10827571513804	2.37311231692440
C	-3.30348515799250	0.38092301934813	-4.50525414579816
H	-2.36185954607710	0.91433617551887	-4.75096193048540
H	-4.15430299865191	1.07901188099828	-4.62419840175399
H	-3.42946579682154	-0.44164952306180	-5.23749586272004
C	-2.18294418549525	-1.28807948984257	-2.94642306054098
H	-4.24730021395921	-0.64941251658853	-2.88235722071288
H	-2.05415697534800	-1.63639423257855	-1.90324845379459
H	-1.18648371598559	-0.96156319236475	-3.29962849423169
H	-2.48760614435924	-2.16203278929516	-3.55596757103010

TS-2b

250 conformers were generated by CREST, all of which were used for constrained optimizations at the GFN2-xTB level of theory. Subsequently, the number of conformers was reduced to 156 by RMSD calculation and cutoff following RMSD ≥ 0.15 Å^2. After constrained DFT optimizations, the number of conformers was again reduced from 156 to 137 by RMSD ≥ 0.20 Å^2. All 137 conformers were subjected to transition state optimizations and frequency calculations.

Electronic energy (a. u.) | DLPNO-CCSD(T)/cc-pVTZ: –4111.22933365562
Thermochemical Corrections (a. u.) | PBE-D3(BJ)/def2-SVP: 1.378416051103
Imaginary Frequencies: one (–656.95 cm^{-1})
Gibbs Free Energy (a. u.) –4109.850917604520

182

C	4.22811595333883	-1.01973738451625	-0.13363963435567
C	5.39564187097862	0.74130064823584	-1.57722090892940
C	5.50488192774828	2.12265455709381	-1.97804140203002
C	4.52436059459083	3.05564769164473	-1.53883547597793
C	3.42086484167038	2.66568313261964	-0.78913962506269
C	3.32215609132698	1.28958504732061	-0.40741671708584
H	4.63479647292184	4.11454863910241	-1.81960310934390
C	5.31221674739612	-1.57766382956899	0.63620773259306
C	3.06688497463681	-1.78330861030687	-0.30898731163690
C	5.22701382710843	-2.94935402675384	1.07313524404965
C	4.05420813959355	-3.70099205425780	0.78244096550530
C	2.95901278189711	-3.13691678301560	0.14006365121557
H	4.00941891572798	-4.75703731269430	1.08958823605869
O	2.24395514156635	0.91111896100088	0.36123855827225
O	2.00679910175686	-1.24949486563108	-1.00782749532190
P	1.04966604195744	-0.07236293522079	-0.29267845023426
O	0.24505947672097	-0.60066844107244	0.87353870115297
O	0.36702659603182	0.63104300669194	-1.46804303578910
N	-2.47084568299990	1.62892740556015	2.77398959306635
C	-3.27901402294271	0.78314357338724	2.10703301760728
C	-1.02244701515726	1.41333076539140	2.84712441245047
C	-0.64897171428143	1.07499224986362	4.29046237351706
H	-0.50846950591253	2.35187717498425	2.54707186360067
H	-0.70434592484333	0.61327864790694	2.14397476011621
O	-1.07395687278439	2.09522913030968	5.18673062996081
H	0.44936990048315	0.98956783032818	4.39015638709341
H	-1.10624438571253	0.09284731551695	4.56968960292660
C	-2.47274378376923	2.28726202737749	5.12207592115068
C	-2.92751148275112	2.65969762332809	3.70231764013149
H	-4.02435641553691	2.77816655848990	3.65149105367838
H	-2.46625113022504	3.62404835347202	3.40256925951790
C	-4.67341656066436	0.78901403436946	2.08371762051708
H	-2.72847119291767	0.02589356011496	1.52383119790481
C	-5.49090305867577	-0.12376850851491	1.33097693566579
C	-6.99619236701005	0.08304176863046	1.45909871365656
H	-7.24864247398410	1.16019898854947	1.43734382276518
H	-7.35555045990354	-0.35106602207166	2.41619551011883
H	-7.54668052196462	-0.41539870244418	0.63925621890690
C	-5.07523850393751	-1.55090687429771	1.11326958992104
C	-4.18497243619797	-2.21788909890670	1.98612481475429
C	-5.65362669910959	-2.29544441017462	0.05718907648479
H	-5.20417007380876	1.61107578715646	2.58730524838345
C	-5.34073608616123	-3.64782751419101	-0.12880225283613
C	-3.88197967513574	-3.57426535939052	1.80447743654832
C	-4.45385414311476	-4.29475568670406	0.74571954713236
H	-6.36077346026210	-1.80484045480074	-0.62650261949701
H	-5.79623340767575	-4.20217880117678	-0.96372785073461
H	-4.21202746907217	-5.35832895894162	0.60594245317572
H	-3.75544118760412	-1.67853486582240	2.84201050104387
H	-3.19610581473443	-4.07216168465748	2.50448458809810
H	-3.01514614518771	1.36193174414951	5.44253359663040
H	-2.72468399627167	3.10253395652800	5.82983267338551
N	-2.29649953565864	0.72169799837735	-1.44996254419240
C	-3.10055368126178	-0.20218817681069	-2.07993414107420
C	-4.48135995165064	-0.00817543552810	-2.03612477860200
C	-5.02951373404545	0.98665825602090	-1.10746978165635
C	-4.11303249625463	2.02134752656741	-0.63111556791302

H	-5.32808143087631	0.36689126850834	-0.00898661083357
H	-6.05532860906449	1.31795433490587	-1.34115506879014
C	-2.73712108484946	1.84463953010277	-0.80928343033140
H	-1.22577016594378	0.59768348148150	-1.48098245076867
C	-2.38301353874374	-1.39661784623734	-2.67189913794626
C	-1.68780516710266	2.81027856014945	-0.36445876636691
H	-1.82559821649718	3.07664215933542	0.69682372493463
H	-1.79296437713011	3.76435335355020	-0.91565891967149
H	-0.67554599055932	2.40909745786386	-0.53956547929500
C	-4.64124905497498	3.18335458561389	0.10660614963661
O	-4.00021882348358	4.08463679786282	0.63520827379785
O	-6.00945164139042	3.15389104681286	0.15791198802985
C	-6.61108239738700	4.23924422612952	0.86512671963099
C	-5.51099632441983	-0.72856169302419	-2.82074220623452
O	-5.04890700375916	-1.39621179107825	-3.90821705397757
C	-6.04339478470557	-2.09675378184997	-4.66358742963109
O	-6.70774855420112	-0.68611452470132	-2.54676461064323
H	-7.70281146537757	4.09201677770992	0.78309828171026
H	-6.31585999523708	5.21327072874943	0.42584949690771
H	-5.50598722496678	-2.57558511774026	-5.50115655184539
H	-6.81484705621778	-1.39810704903743	-5.04436544253627
C	6.58721031164461	2.52247308901769	-2.81644373712574
C	6.35423633410094	-0.18563286531265	-2.08520410131890
C	7.38662464189409	0.23239411731669	-2.91263066367226
C	7.51548019655479	1.59979634277384	-3.27303647742115
H	6.25915082475957	-1.24756955525805	-1.82015399425880
H	8.10818802034393	-0.50445208282271	-3.29696829270444
H	8.34244210487956	1.92098056140295	-3.92426588988894
H	6.66214160874421	3.58271461279017	-3.10498298749285
C	4.31245156588112	0.34747923084626	-0.71186161683324
C	6.30899063117582	-3.51776994733230	1.80817531334782
C	6.46191258086743	-0.82151921365132	1.01543286696743
C	7.49003734896348	-1.39847113904508	1.74706714998475
C	7.42390266781760	-2.76236913302538	2.13630650658769
H	6.23186798279374	-4.57015801699608	2.12365432482137
H	8.24912452799217	-3.21118345353482	2.70964274859803
H	6.52229394069994	0.23688601748333	0.72788620957203
H	8.36213624821110	-0.79058149916463	2.03217646417317
C	2.38129774203351	3.64775403825660	-0.35861606755544
C	2.25003954418184	3.97300565448303	1.02100081877233
C	1.27206112742782	4.90273720395255	1.41261890821771
C	0.44389414229089	5.55442172563073	0.48098420515348
C	0.60697676187413	5.23133586218010	-0.87490059780635
C	1.54512741698658	4.27875614520704	-1.31876530149508
H	-0.03247835352595	5.73853898090102	-1.61662122370924
H	1.17941883896577	5.15253938069444	2.48200690110449
C	3.19356360695803	3.40440458193146	2.07909198226514
C	2.45322486258021	2.57499039822525	3.13884530424402
H	1.71194805324231	3.18472523641626	3.69789703916267
H	3.16791397118367	2.16194882440249	3.88052507506248
H	1.93057047079659	1.72644396991773	2.65810662616716
C	4.03510184192753	4.52298112851829	2.71837435866856
H	3.89954746725421	2.72119218695376	1.56687618206185
H	4.58804608995914	5.09954604339328	1.94972691997501
H	4.77409842866120	4.09980748088355	3.42972352514884
H	3.40015462020254	5.23850061249265	3.28141108164151
C	1.63203769239432	3.96493497299634	-2.81117234504305
C	0.30449598406238	3.42874717929920	-3.37231846608834
H	0.40249766688751	3.22015886111523	-4.45774353963616

H	-0.52353646378047	4.15808464680359	-3.24776070784505
H	0.03279548693660	2.48441813184419	-2.86476846385617
C	2.13186250666270	5.18366250820406	-3.60568545480040
H	2.37612854654783	3.15417149874217	-2.93703060691872
H	2.25248103973973	4.93024316121680	-4.67915130849192
H	3.10940576699697	5.54537584793923	-3.22656638950405
H	1.41786661648801	6.03117979820115	-3.53929834445997
C	-0.54827432269105	6.62304723931706	0.92048929187494
C	1.72404293826703	-3.94263969112995	-0.11562460267039
C	0.77205561161779	-4.13615982272185	0.92030787395807
C	1.53426903621086	-4.54894713379773	-1.38479332164131
C	-0.32594925203609	-4.98124357617708	0.67357755874535
C	0.41507462822774	-5.37597679465677	-1.58332371029406
C	-0.51981070160164	-5.61888791720009	-0.56274508189824
H	-1.06527572201454	-5.14920437114954	1.47199613016050
H	0.27389005439092	-5.84909519785894	-2.56836876492460
C	0.93786631523252	-3.45852539311221	2.27845859032674
C	-0.38888138871747	-2.95921394469175	2.86730161697883
H	-0.19222763797804	-2.32829845790254	3.75907534596373
H	-1.03597650826356	-3.79752404642756	3.20165549581509
H	-0.93706536486946	-2.34688995283105	2.12745125133517
C	1.66197616997057	-4.37589338794561	3.27994932199779
H	1.56512225102742	-2.56017463675113	2.10802256161411
H	2.66701870090125	-4.66655166521949	2.91912930207782
H	1.08267619348572	-5.30734977871081	3.45331606312703
H	1.78820170723699	-3.86661535139148	4.25799703369635
C	2.50146862862988	-4.30652283385877	-2.53893469542186
C	3.06637823555106	-5.61714259971544	-3.10920551222544
H	2.27050186504299	-6.24451548736111	-3.56218476234505
H	3.56073857177641	-6.22001968829147	-2.32092584679199
H	3.81320381562706	-5.40774584163404	-3.90238836968769
C	1.84345330260228	-3.44400479744030	-3.63053294162128
H	3.35874111962259	-3.72909267466380	-2.13729958440686
H	0.97644471864749	-3.96775265331407	-4.08667070100451
H	2.56574851467158	-3.21929387480228	-4.44241933350689
H	1.48665141302449	-2.48489766736756	-3.20841728918332
C	-1.70085210308517	-6.55309151174111	-0.78677329048331
C	0.19251255396031	7.88113863476075	1.40897426180181
H	-0.52284345069742	8.69047687092332	1.66376177837925
H	0.88863952652829	8.26385612642837	0.63611765361732
H	0.79087893878806	7.66185387787052	2.31795386498729
C	-1.54405543750790	6.11713758970210	1.97715182226977
H	-1.13469189113903	6.90826894187112	0.01943399569127
H	-2.23240247676229	6.93212331393049	2.28307084899719
H	-1.01878176135704	5.77203803668406	2.89290455831382
H	-2.16535292245563	5.28844657219447	1.58628407634223
C	-2.60262397882025	-6.08687470656119	-1.94197470923824
H	-3.48602770055279	-6.75091747402514	-2.04669931479340
H	-2.05818936721398	-6.10617326814900	-2.90894198967967
H	-2.96223318335417	-5.05193999846707	-1.77970131390517
C	-1.23434438698654	-8.00418704569630	-0.99463580934740
H	-2.30747546425845	-6.52816421338081	0.14561260199035
H	-2.09996249049384	-8.69077078226073	-1.10033296817442
H	-0.61645452715628	-8.35309139126915	-0.14336183632229
H	-0.61865858919376	-8.09554320233000	-1.91353123841608
H	-6.54853577252893	-2.86159620534011	-4.03920571352686
H	-6.30161040988296	4.23913275231183	1.93113948683659
C	-1.31493301819482	-1.01222702381488	-3.71012972696727
H	-0.89949891375458	-1.93384385646508	-4.16326639329117

```
H   -0.47196771530129     -0.45566185305952     -3.25528341766086
H   -1.75459018132732     -0.39907357440364     -4.52243204546244
C   -1.82476352524583     -2.25622134315558     -1.51662948655538
H   -3.15221143138270     -1.99311633580189     -3.18827273563096
H   -1.28944071164782     -3.13665703192125     -1.92257782116856
H   -2.64562256556483     -2.62161866553739     -0.86795125607797
H   -1.11109255964662     -1.70921017230570     -0.87139724800555
```

TS-3

174 conformers were generated by GOAT. Subsequently, the number of conformers was reduced to 109 by RMSD calculation and cutoff following RMSD ≥ 0.10 Å^2. All 109 conformers were subjected to constrained DFT optimizations followed by transition state optimizations and frequency calculations.

Electronic energy (a. u.) | DLPNO-CCSD(T)/cc-pVTZ: –5199.14938975462
Thermochemical Corrections (a. u.) | PBE-D3(BJ)/def2-SVP: 1.918448315521
Imaginary Frequencies: one (–566.00 cm^{-1})
Gibbs Free Energy (a. u.) –5197.230941439100

```
248

C   -0.15196725590091     -4.64094817236051      1.07078252128286
C    1.90517217134921     -5.85319940892892      0.18142304135856
C    3.28857927525065     -5.80927604082442     -0.22179157309134
C    3.94443509379975     -4.55118515685713     -0.31581831098644
C    3.28667861831521     -3.35387470101199     -0.06104953096450
C    1.93875708673517     -3.41512239255865      0.41830384234365
H    4.99554403107120     -4.52379511262758     -0.64184783534726
C   -0.57660128257273     -5.47012521435770      2.16976460501546
C   -1.10989006155191     -3.84087419454471      0.43598737298288
C   -1.98351051590689     -5.56212182073789      2.47317108214173
C   -2.91709799159553     -4.80565503883742      1.71001148179152
C   -2.51021588282839     -3.91547606544143      0.72336805459682
H   -3.98986577587851     -4.90948283641055      1.93195577532737
O    1.33525620969435     -2.23622827085901      0.79832153687338
O   -0.70691607285902     -2.98058859046797     -0.56120760943825
P    0.07533805489268     -1.57466022633276     -0.11540140931166
O   -0.72763443915661     -0.73545861818675      0.85735952468231
O    0.62070580609384     -1.00760430413201     -1.42450928443623
N   -0.32763019194516      1.39470641501698      3.77918739302757
C   -0.89903445784079      1.77726752091571      2.61343400248303
C    0.50125874743864      0.19178896431722      3.83930780430294
C    0.22347317055420     -0.63564751624815      5.10617162480615
H    1.57178275824126      0.49722809114976      3.85795341088664
H    0.31903917655495     -0.40089366097630      2.92154644942265
O    0.34921590162129      0.22277686141888      6.25125225397585
C   -0.54281713328711      1.31882764970049      6.21298875026529
C   -0.30774552501165      2.20701085958806      4.98688310637942
H   -1.09426641397392      2.98239089368186      4.92893349946191
H    0.67611604102072      2.72097548092332      5.07833654131445
C   -1.55271823117171      2.97601184663150      2.36417855038907
H   -0.83255864869882      1.00026648895369      1.83069685856833
C   -2.18458075567700      3.30114509596440      1.10320412130841
C   -2.59971914767073      2.18190510445775      0.16707660026110
H   -2.74688994041022      2.56153000479281     -0.86086228528442
H   -3.56013584992103      1.73711325040799      0.49646924152471
H   -1.86767050216057      1.35282638530146      0.13792231289935
C   -3.04823592825295      4.53419757601762      1.10998090370330
```

C	-4.37656546904851	4.50763768209336	0.63684476664504
C	-2.52372890242720	5.75920600014983	1.58302200068264
H	-1.55360904785600	3.76689269447862	3.12842183752481
C	-3.30929260742470	6.91883529290687	1.58735899047327
C	-5.16645752293762	5.66778674433783	0.65442235175492
C	-4.63621759298459	6.87821265357386	1.12598659936126
H	-1.47595344722099	5.80293133849322	1.92323181176004
H	-2.87942625887925	7.86559321750525	1.94875314151013
H	-5.25337634446656	7.78943457022891	1.13053917579618
H	-4.80759216159413	3.56914468132265	0.26023812617168
H	-6.20451641143823	5.62278320008916	0.29095555386332
N	0.84611434057085	1.60124849686657	-1.17232970415471
C	0.12462773598910	2.35780100874609	-2.06169230550913
C	-0.26806555819523	3.63513660580692	-1.67336828456206
C	-0.03146093785544	4.04292444105819	-0.29327770445016
C	0.94101488779824	3.29011705000153	0.49005535937936
H	-1.15928739797823	3.78999745984804	0.37344589983683
H	-0.05798661987887	5.12513571951068	-0.08749450478100
C	1.32350644903087	2.02546726121484	0.03478018552778
H	0.90269148198947	0.54328395548829	-1.36585731842170
C	-0.13063262730036	1.68546079831298	-3.37483753262730
H	-0.37799876229760	0.61892516200610	-3.19915066428601
H	0.79603615980095	1.70402130299811	-3.98743385102172
H	-0.91722264511532	2.19899172067766	-3.94910667888473
C	2.24381175292053	1.06264553865771	0.71496831290682
H	1.78565112379997	0.66772552627419	1.63813407191547
H	3.17891107470002	1.56356629881944	1.01726221177509
H	2.48350962855211	0.21220728853423	0.05518120222299
C	1.40265995945068	3.95827388427807	1.72566065777177
O	1.05288386494992	5.08898751252401	2.05079979251737
O	2.27378357727525	3.22234665751639	2.46641060519761
C	2.83051067297494	3.87921325963385	3.62248483135294
C	-0.99728005407955	4.55287315900212	-2.58562742073882
O	-1.43373137953587	5.64646983827163	-1.90616554399105
C	-2.16533619660528	6.63525525004807	-2.66399062005930
O	-1.19844141198883	4.38552106075597	-3.78211385803004
C	3.67261797502889	2.87058855194911	4.37735044478537
H	2.00151672915342	4.28863038154898	4.23840897058302
H	3.42839197979379	4.75091188453484	3.28057163754464
H	4.16430495108759	3.36112430335360	5.24049381566113
H	4.46203310826044	2.44355446370621	3.72751064647282
H	3.05766913033819	2.03390166208529	4.76651098568002
C	-3.63316389198951	6.26772731746497	-2.80021674116307
H	-1.68596298393544	6.74499739344350	-3.65813399211226
H	-2.03443634744192	7.57089713809545	-2.08635841716779
H	-4.08853678420990	6.09680061999131	-1.80543225814886
H	-3.75344970583292	5.35435878040991	-3.41354311306650
H	-4.18092399224580	7.09295892585975	-3.29856889116506
C	3.95832865854652	-7.01930640364857	-0.57005744174501
C	1.23654334893670	-7.11403243467942	0.13775802590315
C	1.91000439264090	-8.27160540172510	-0.22480344824336
C	3.28782166511295	-8.23196769540630	-0.56497175877288
H	0.16827625246738	-7.16152707126623	0.38675154048762
H	1.36713423429372	-9.22848199177213	-0.25516958288720
H	3.81304899133148	-9.15829710691510	-0.84242710460468
H	5.01872118338324	-6.96560255772311	-0.86260061348164
C	1.25093660115652	-4.62864656310020	0.57576980589520
C	-2.40969840451557	-6.38621371576953	3.55658637698084
C	0.34192671386598	-6.17510530011679	3.00277397758243

C	-0.10317424615161	-6.95245031663693	4.06245966994874
C	-1.49135059906412	-7.07091216827072	4.33762531755461
H	-3.48781989252631	-6.45350444584558	3.77096982395959
H	-1.83371698995459	-7.69585482818695	5.17618867272488
H	1.41821159020228	-6.08016271420744	2.80384747286647
H	0.62648070152319	-7.47731403937858	4.69751777948589
C	3.99663793508729	-2.05731891149950	-0.31184547173756
C	4.69600683812678	-1.41020951933475	0.73970752663400
C	5.47203893993767	-0.27353741043022	0.44617448387398
C	5.57811975705211	0.23223646501702	-0.86176555041258
C	4.83348804926237	-0.39296417686724	-1.87780318723322
C	4.03985677391061	-1.52844901883397	-1.63260768592207
H	4.90706867572499	0.00517469985153	-2.90032082725555
H	6.03906930213070	0.22252134804838	1.24834210691067
C	4.68534021091840	-1.97880670978920	2.15486959867531
C	4.53417263032754	-0.89998380500384	3.23576872584507
H	5.40330678874079	-0.21087296689084	3.26215978455455
H	4.45883519100394	-1.36536592371925	4.23933742095057
H	3.62353640727188	-0.29222442242120	3.07051011336621
C	5.94394901849207	-2.83206987217719	2.39506664545804
H	3.80693487573057	-2.65231435450206	2.23416113553338
H	6.01200607339418	-3.66195019907006	1.66392112097897
H	5.93741141443198	-3.27286263354638	3.41340971879659
H	6.86229197744502	-2.21638531239941	2.29299591860300
C	3.29935526852165	-2.19599422967351	-2.78870110386224
C	2.72780263856832	-1.18858581255257	-3.79676288538617
H	2.05723233780288	-1.70754414115062	-4.51076438554135
H	3.52454975415325	-0.69735447414813	-4.39354395553205
H	2.13385563501741	-0.41096541565146	-3.28458203603001
C	4.20385476652216	-3.21159043337458	-3.51146972022831
H	2.43895988153353	-2.74185063371214	-2.35154449687083
H	5.09309311124282	-2.70681357556857	-3.94448681297677
H	3.65356325400147	-3.70223265050071	-4.34057339430283
H	4.56386484316599	-4.00579157570242	-2.83005669560434
C	6.52761950470813	1.34182051248982	-1.19177404125195
C	-3.47525213385914	-3.05548070064620	-0.02684575706738
C	-4.22290926116406	-2.05326944446286	0.65369732426560
C	-3.64965182192252	-3.24161959190547	-1.42679972520717
C	-5.11432289750678	-1.25374354948320	-0.08494462598797
C	-4.57313171408461	-2.43512642436721	-2.11331495934875
C	-5.31249009477400	-1.43193592156684	-1.46485885885649
H	-5.69939690734401	-0.47579698222178	0.42958934809955
H	-4.74603760573206	-2.60730848388336	-3.18646009158655
C	-4.13157761500041	-1.83534963509699	2.16175838717805
C	-3.85209891604003	-0.37109924810048	2.52435899812769
H	-2.90186561506769	-0.04173414805989	2.06711172881618
H	-3.77457254954070	-0.25026064850218	3.62422769225895
H	-4.66250919013511	0.30699669887638	2.18554905461265
C	-5.40098788839210	-2.35031544352880	2.86402681231230
H	-3.27370081017804	-2.42734967234886	2.53589793842293
H	-5.58912572772379	-3.41972641938388	2.64033734473994
H	-6.29727923503048	-1.78374237669515	2.53497406141144
H	-5.31519997097184	-2.23991952504179	3.96501829154814
C	-2.91765096852804	-4.32995699889109	-2.20646798667451
C	-3.90035913227324	-5.40942694155486	-2.69381132421262
H	-3.35832243430706	-6.22720127075116	-3.21201200513432
H	-4.64133912835361	-4.99313133420001	-3.40782088455825
H	-4.46305690765059	-5.85205020369384	-1.84709261514136
C	-2.08769066894740	-3.74860575781892	-3.36308688324345

H	-2.21327839061314	-4.82870667009752	-1.51257959898836
H	-2.73451387192824	-3.28087669984235	-4.13503419076858
H	-1.50522453399924	-4.55019906835158	-3.86225142797282
H	-1.37629975958038	-2.98541503056084	-2.99297126332912
C	-6.34100354255073	-0.62814411533185	-2.19839211982271
C	6.06513321404084	2.66091437677633	-1.44621693672308
C	7.91611128286183	1.05028129165641	-1.28218607495817
C	-7.70012641105496	-1.03299670492404	-2.14031183312993
C	-5.97477216262365	0.53035123999925	-2.93779271047558
C	6.99698976917146	3.65801400831889	-1.78397066031786
C	8.80968655146281	2.08664478967724	-1.61066357344243
C	8.37520135330585	3.39600510563929	-1.86908018029310
H	6.63201061152236	4.67856923184983	-1.98259723508181
H	9.88786311277086	1.86693144007180	-1.67858611155713
C	8.45809589981190	-0.35582425945756	-1.03470873507551
C	9.33172975348161	-0.85564029866146	-2.19653850842136
H	9.64144617725128	-1.90719390323201	-2.02592944216891
H	10.25711731442022	-0.25339757861468	-2.30726897607968
H	8.78347489345447	-0.81064156490482	-3.15883585916150
C	9.20902839701727	-0.43388744939573	0.30569401496852
H	7.58784184363801	-1.03849871953233	-0.96289393908945
H	9.56381012499515	-1.46716092824930	0.50157251417454
H	8.55713627828304	-0.13075239951502	1.14958520251514
H	10.09498231566229	0.23520443476752	0.31125924696931
C	4.58559278211361	3.02192553401497	-1.35677798913091
C	3.98692067759731	3.32073256439754	-2.74203823353889
H	4.10787375391200	2.45737906350556	-3.42701056104999
H	4.47968851267884	4.19509007108245	-3.21637404037604
H	2.90284513679773	3.54807257408225	-2.66379037289060
C	4.34037706225823	4.18314381592622	-0.37935552466041
H	4.06032486298445	2.13004225490801	-0.96274497142397
H	3.25902276663406	4.41359123667744	-0.30746265548422
H	4.85236085257827	5.11032010118403	-0.70897078905539
H	4.71109515604466	3.93965122262720	0.63693857575831
C	9.36553263535213	4.49103321101262	-2.24024507946102
C	9.34354437455318	5.64935487907879	-1.22893042439537
H	10.11151271794101	6.40963780172875	-1.48036114493069
H	9.53726116800377	5.28913512926828	-0.19881663461960
H	8.35798996788715	6.15973614184798	-1.22583168936307
C	9.13300623523996	4.99745808468766	-3.67402621371876
H	10.37826592258873	4.03265582745468	-2.20516814899189
H	9.89514404776773	5.75240400719572	-3.95769233250711
H	8.13594955985783	5.47535044695066	-3.77125583227753
H	9.17877950769578	4.16705713008941	-4.40652769444285
C	-8.67021045211413	-0.26351608241846	-2.81067102402329
C	-6.97990148568298	1.25988967003142	-3.59576261081874
C	-8.33466304455195	0.88481284511233	-3.54269856108039
H	-9.72866261741907	-0.56826588334904	-2.76264182466889
H	-6.69706281204843	2.15643835717614	-4.16791239654879
C	-8.12904321450579	-2.27026076813073	-1.35551858657047
C	-8.96873008709146	-3.23734756838260	-2.20492508979486
H	-9.19408021759168	-4.16193760741622	-1.63465386102232
H	-8.43190713362055	-3.52668343348791	-3.13056018765307
H	-9.93947371344494	-2.79034887608836	-2.50411936885244
C	-8.86057162961973	-1.87739339403422	-0.06047975771170
H	-7.20582737028740	-2.80907720243669	-1.06141573338572
H	-9.14044574457087	-2.77736078055122	0.52551948037012
H	-9.79037654632762	-1.31179335966940	-0.27985132739874
H	-8.22233804917926	-1.23699083377779	0.58096302453163

C	-4.51394344297029	0.95795937853327	-3.05761788369591
C	-4.32828949893693	2.48099366030132	-3.09764456994185
H	-4.73710780229130	2.92408404016747	-4.02915240859978
H	-3.25230224603507	2.74379892882181	-3.07924536337083
H	-4.82265460798560	2.98386802536860	-2.24239863939185
C	-3.85432924816703	0.30846886452104	-4.28930774442283
H	-3.98608772525085	0.57435886921534	-2.15795604339715
H	-3.89545734185337	-0.79600839459937	-4.23820518147015
H	-2.78811076766680	0.60339682977191	-4.36711434812472
H	-4.36768172051966	0.62687648431733	-5.22040526226288
C	-9.40515574257920	1.70098175844324	-4.25378236022649
C	-9.18962955136323	1.72158949338115	-5.77636239190821
H	-8.23624817148176	2.22518207598106	-6.03989885565310
H	-10.00839900343706	2.27045865500607	-6.28596314697084
H	-9.15079658454483	0.69479035937565	-6.19170172728223
C	-9.50167004185623	3.12673525777477	-3.68466790063049
H	-10.37612707363544	1.19475503293373	-4.05866505789697
H	-9.68647840654756	3.11172837450365	-2.59189381187845
H	-10.32494194725332	3.69277183455352	-4.16758085392241
H	-8.56160780632174	3.69092327597463	-3.85814953947993
H	-1.60436255099385	0.97491113845741	6.21864674962760
H	-0.37072345921306	1.90732447409310	7.13676935650803
C	1.23115831928464	-1.78362695800478	5.24261929350609
C	-1.12276185158734	-1.38545261203438	5.01758211461981
C	0.70741995983551	-2.89300170522364	4.30929587675714
H	1.20402390548730	-2.09633568695757	6.30680304565924
H	2.26703480004544	-1.46005105273532	5.02090561286424
C	-0.81509866369002	-2.61060997854151	4.13347797676694
H	0.89973064728020	-3.90031248290189	4.72514949319564
H	1.21413491161741	-2.85746838344148	3.32413150959393
H	-1.44234216281105	-3.48035804148321	4.40618919462821
H	-1.03594861035461	-2.38215247068306	3.07194499962520
H	-1.94232841783504	-0.76393246622447	4.60953600156919
H	-1.40152242359004	-1.68590628026377	6.04947906075626

TS-4

45 conformers were generated by GOAT, all of which were subjected to constrained DFT optimizations followed by transition state optimizations and frequency calculations.

Electronic energy (a. u.) | DLPNO-CCSD(T)/cc-pVTZ: –5199.14698169226
Thermochemical Corrections (a. u.) | PBE-D3(BJ)/def2-SVP: 1.919610586735
Imaginary Frequencies: one (–630.64 cm^{-1})
Gibbs Free Energy (a. u.) –5197.227371105520

248

C	-0.33501076724424	-4.62983214313133	0.20645211655079
C	1.41317533688767	-5.72897945491957	-1.28122741596308
C	2.70013737957584	-5.66694235985876	-1.92619141241783
C	3.44683691949347	-4.45891071252743	-1.86541856045362
C	2.96065633234087	-3.31300053481958	-1.24475349379875
C	1.69952170663480	-3.39909731041788	-0.57357621831806
H	4.43367608612619	-4.42215209986493	-2.35181806713090
C	-0.60389826600341	-5.62539156381462	1.21168901916056
C	-1.33499705790582	-3.69902618188797	-0.09838797696060
C	-1.92707507595496	-5.70660348688371	1.77996864940859
C	-2.93824194124022	-4.81238151949060	1.32667787531658

C	-2.66891428972234	-3.80296417497298	0.41089299703460
H	-3.95949206015433	-4.91953273575508	1.72315963556890
O	1.23655702173191	-2.29510530147530	0.11007363404026
O	-1.06055198497646	-2.68608856097264	-0.99140379791985
P	-0.08115161665891	-1.41946361801888	-0.49487268213160
O	-0.64994237337345	-0.67293447713305	0.69466266956441
O	0.32216328556857	-0.69715937620489	-1.77840743379217
N	0.66145690893030	0.84706842920614	3.76836448662302
C	-0.12843161585376	1.59607484990034	2.96915814385493
C	1.29986131454726	1.31566857836859	4.99302967031054
C	1.10808104855429	0.26238050269616	6.09114466656753
H	0.84933310980252	2.27321366014213	5.31396824636135
H	2.38019214651647	1.48630142517664	4.79385669957954
O	1.56700396545744	-1.01328996874674	5.69557829667700
H	0.02944085891121	0.23289568538396	6.38207137253328
H	1.69355710819207	0.54996898427741	6.98802676446294
C	0.89454479142789	-1.51145641515051	4.52025542278786
C	1.07624188891992	-0.49964944439043	3.38012411769265
H	2.15717772154841	-0.46677250770096	3.11917167029585
H	0.50461891221528	-0.80813656196829	2.48243311787857
C	-0.38950189794430	2.95331579943794	3.12889641937484
C	-1.19733570148373	3.83052419949677	2.30477366563856
C	-1.31191436065572	5.24789259019967	2.85774587753537
H	-0.32934800072173	5.57966458721931	3.24459607991785
H	-2.03822358360908	5.29372382920031	3.69626081240992
H	-1.62700106593337	5.95760959249565	2.06990868853810
C	-2.36685245654025	3.35297540431937	1.48903956759301
C	-2.27879023570812	2.25101036189194	0.60608911700266
C	-3.59612139068612	4.05423392954116	1.53943954412927
H	0.16970762723450	3.47858459066790	3.91849963909704
C	-4.69255120613867	3.65760356779784	0.76329803307805
C	-3.37399599560458	1.86249296076403	-0.17931875423108
C	-4.58729523715476	2.56227885779065	-0.10853377209848
H	-3.70565668124799	4.91982394561716	2.20611484692958
H	-5.64079249269696	4.21260829852099	0.83519888449089
H	-5.44539355855492	2.25317728922942	-0.72258492869969
H	-1.36315029873162	1.65218399200372	0.52266341615012
H	-3.27463170240086	0.98093690239704	-0.82973302750291
N	0.81111254130748	1.83321395440961	-1.08382676499553
C	-0.05116092361709	2.76714324790484	-1.59640705211760
C	-0.15785135817796	3.99402067876869	-0.94611071193411
C	0.55531899557906	4.20355315478246	0.30906182655145
C	1.53024256385540	3.19765118378312	0.72455110984660
H	-0.36650062549846	4.09411806584510	1.25505681374717
H	0.78117809727284	5.25465626931920	0.55527915109522
C	1.60523426851852	1.99781854179634	0.01597723468113
H	0.72530863234434	0.83510704180854	-1.46854867896308
C	-0.80072514524706	2.31486262479933	-2.81118249490562
H	-0.67205693618049	1.22465967952203	-2.94998035244000
H	-0.44415757644638	2.84867108561842	-3.71454436341923
H	-1.87295824911960	2.55218813048037	-2.71245487468607
C	2.49687178554646	0.84318150285825	0.32336364673375
H	1.95720303873748	0.11345773908857	0.95650967511243
H	3.38929018359587	1.16244618585395	0.88217151953765
H	2.77761494723482	0.31510762958624	-0.60388613962479
C	2.35817989271662	3.43403883690111	1.92766536934352
O	3.06264289452289	2.62116555702217	2.51727827325330
O	2.26279132776981	4.73537016229950	2.33246616308356
C	3.03319210670755	5.08408965020656	3.49649625493497

C	-1.08030673879154	5.09335170480827	-1.31684497770926
O	-1.66549734881210	4.94308548768864	-2.53107125015419
C	-2.64191486661496	5.94367073501294	-2.90174468576058
O	-1.29370232329459	6.04934667112887	-0.57913285207696
C	2.85976976235736	6.56841909664649	3.74462516115548
H	4.09393352602085	4.80594190191814	3.32192480681341
H	2.68306077080269	4.47065852399820	4.35575725336895
H	3.45187249445118	6.87721195985020	4.62865937435304
H	1.79802949841433	6.82413750376480	3.93431637133405
H	3.20525110400701	7.15825126044185	2.87272104569591
C	-4.01856061898091	5.61467979578061	-2.34748475994418
H	-2.63417284244700	5.93965853692438	-4.00929987577192
H	-2.29223229086954	6.93004213863317	-2.53622273939113
H	-4.75235333049427	6.37376486963993	-2.68688352635379
H	-4.00707420550031	5.61348956155945	-1.24079561202980
H	-4.36531224716232	4.61978713431257	-2.69053641101258
C	3.18348715271399	-6.80348343407707	-2.63958454860128
C	0.63829063147348	-6.91741765925326	-1.44184651696601
C	1.12817929203574	-8.00035916492903	-2.15730884435499
C	2.41800519516136	-7.95341824020230	-2.74971600705058
H	-0.36536552261870	-6.96401379511865	-0.99861528514968
H	0.50733438415392	-8.90188562854418	-2.27174604347465
H	2.79834166660875	-8.82197487155158	-3.30822326692836
H	4.17431729690623	-6.74076873660424	-3.11620636610403
C	0.95074857749973	-4.58428678098123	-0.53776481577154
C	-2.19350324054423	-6.68267427282176	2.78486309035086
C	0.39744120696259	-6.51900305706489	1.69746621133580
C	0.10648066851012	-7.45478747573654	2.68014585474272
C	-1.20046862700682	-7.54313881469019	3.22774475518388
H	-3.20862171077477	-6.73432168155949	3.20883205852380
H	-1.42020038691131	-8.28801541880993	4.00731046643873
H	1.41577040986235	-6.44721165970894	1.29113269878041
H	0.89876074338309	-8.12569375186202	3.04506614261602
C	3.78699913049356	-2.06371656112954	-1.29176377910977
C	4.62623534349617	-1.72810895553033	-0.19530689353700
C	5.52483651051945	-0.65484750319612	-0.33006587168417
C	5.61901640748189	0.08867635771470	-1.52083334448632
C	4.73215318314745	-0.21849343624224	-2.56709395719627
C	3.80818758694183	-1.27696187519734	-2.47624089057389
H	4.78859041099356	0.37278267068952	-3.49300018576800
H	6.19281172612997	-0.39532789762082	0.50400332012184
C	4.60773201964540	-2.55579268517699	1.08614236918334
C	4.86058313421741	-1.72617001931096	2.35250959977299
H	4.19717399706121	-0.83958712920091	2.40436281833591
H	5.90788015884770	-1.36446847738206	2.40882920630436
H	4.68019595643482	-2.34044251874044	3.25746910012111
C	5.61179410550492	-3.72011061396932	0.98705281669671
H	3.59268838428582	-2.99580449370928	1.17608786652093
H	6.64494537294919	-3.33323482353017	0.86088079445062
H	5.38613450554247	-4.37553867103641	0.12318244474196
H	5.58831812448397	-4.34330612795596	1.90490584810490
C	2.88155329858138	-1.57295110654426	-3.65245726810298
C	2.30006134249549	-0.29950538439872	-4.28550908913080
H	1.81882526908382	0.33984473761222	-3.52319061364146
H	1.52729566901253	-0.56929924977019	-5.03330110921508
H	3.07293443864896	0.29809939326921	-4.81238341697255
C	3.59017035157340	-2.42562674044286	-4.72097612160351
H	2.02543028933689	-2.15127681697356	-3.25042352659588
H	3.93621214520851	-3.39399789168436	-4.31189319181143

H	4.47606800206727	-1.89339815942117	-5.12704954563979
H	2.90490092973172	-2.64079505937061	-5.56652358509658
C	6.68648778357325	1.12214235496583	-1.70648846698855
C	-3.72110120761384	-2.86868701321770	-0.09041408285023
C	-4.25899863534667	-1.85926914873894	0.75210047164421
C	-4.17217611718161	-2.98577417604263	-1.43341390336929
C	-5.18005884945074	-0.94990747441631	0.20859056656056
C	-5.15108534405638	-2.09526308934030	-1.90648852117206
C	-5.64499088526906	-1.04045858203970	-1.11843177666771
H	-5.53893091399361	-0.12245459786232	0.83596163869748
H	-5.53174443020103	-2.22123877332112	-2.92978751150610
C	-3.90016867394527	-1.74820826101845	2.23000159263715
C	-3.44998249689880	-0.33848948346796	2.63299835372142
H	-2.55500212158037	-0.05099665809377	2.05324138876999
H	-3.19968415806630	-0.30801233881501	3.71322284011433
H	-4.23474581053518	0.42583529454975	2.45844430108327
C	-5.07628998691728	-2.23127638237948	3.09828571570091
H	-3.04518502857304	-2.42706911079938	2.41259142875415
C	-3.63417439218503	-4.04802320464258	-2.38711601937733
C	-4.73760062641277	-5.02780747701265	-2.82026194384889
H	-4.31935868078219	-5.82772480684814	-3.46540043994249
H	-5.53342100940584	-4.51526843929836	-3.40028749660037
H	-5.21744314598197	-5.50777746162398	-1.94362400283090
C	-2.93245800667112	-3.40758301251387	-3.59798654601195
H	-2.87303869620988	-4.63996421562063	-1.84068111752242
H	-2.46984107775513	-4.18684008197584	-4.23821243550709
H	-2.13562648162264	-2.71399208274252	-3.26707987228997
H	-3.65071719252015	-2.84174387418289	-4.22770237772791
C	-6.58105475420061	0.00031538886057	-1.64053917202512
C	6.39476564765422	2.50874521162884	-1.63202839823157
C	8.01311902344043	0.69139090107637	-1.99045871860873
C	-7.77871921162521	0.30352903906620	-0.92885876907394
C	-6.24009411929258	0.78305506323916	-2.78749341549184
C	7.43299733462916	3.43458473739349	-1.84930244062486
C	9.01745405223380	1.65543243892572	-2.18126862825627
C	8.75017899943475	3.03425291966416	-2.12033096321428
H	7.21165824414460	4.51329970235569	-1.80026665138632
H	10.04399769760957	1.31614399309889	-2.39343144879544
C	8.36924712232814	-0.79089979645735	-2.08534274370275
C	9.12562743623057	-1.13005572139704	-3.37966693048985
H	8.56055369672298	-0.79843982803328	-4.27373514644461
H	9.28637754750671	-2.22476774024062	-3.46052998846766
H	10.12479487519294	-0.64850267944948	-3.41481427782720
C	9.15118215621007	-1.25168550141151	-0.84274637803421
H	7.41759484575213	-1.35905515281559	-2.10456669584495
H	10.11452740530243	-0.70735438598314	-0.75202566847860
H	9.37725020152838	-2.33705387558448	-0.89586885867533
H	8.57451551385360	-1.06749502509765	0.08570596883025
C	4.98686713150701	3.01508816048675	-1.33091854146290
C	4.31058882108824	3.58584126627996	-2.59004024884690
H	4.26008265337634	2.83037546726728	-3.39952176461456
H	4.86840374750838	4.46175865324208	-2.98223788346902
H	3.27444025679446	3.91578604170373	-2.36501588728047
C	4.96995752517456	4.03411469817789	-0.18016086842986
H	4.39270382224279	2.13740540638069	-1.00974007866409
H	5.58949905995642	4.92528750332625	-0.40935439526642
H	5.35070139625326	3.58671583575392	0.75942613329965
H	3.93959500574852	4.39732663018390	0.00496440448092
C	9.85173364692375	4.06152950779433	-2.34180799241959

C	10.43652174833239	3.96904689605146	-3.76130342397094
H	10.93208440269051	2.98975838744654	-3.92663639921223
H	11.19569033045334	4.76035949505324	-3.93113113964533
H	9.64565549333670	4.07702006753852	-4.53018596267059
C	10.95397930467337	3.94906187228686	-1.27498445330493
H	9.38159186214386	5.06396257300581	-2.23604107680169
H	11.47064401174287	2.96867556161792	-1.33640975800134
H	10.53589205379473	4.04318614594523	-0.25287790843874
H	11.72127141767363	4.73926988028650	-1.40996904048781
C	-8.55388514240880	1.41257667377195	-1.31628430669560
C	-7.06192529024792	1.86702600149604	-3.13997641398989
C	-8.20382049500631	2.22525122164830	-2.40458366990777
H	-9.46419465470512	1.64419786966010	-0.74262344494070
H	-6.78947257366406	2.48179613076341	-4.01286717589204
C	-8.28872561051672	-0.56006431493559	0.22426600157108
C	-9.65007580476050	-1.19025403292177	-0.11979825373081
H	-10.43723598440573	-0.41943050333840	-0.25483811786213
H	-9.98268624446796	-1.86882248777159	0.69270036527180
H	-9.59274596261088	-1.77845999863031	-1.05732709652455
C	-8.35580161668401	0.21864231588897	1.54868838442494
H	-7.57226684989106	-1.39302824475643	0.36173430284205
H	-8.65145455936024	-0.44991272339443	2.38346052200128
H	-9.10089972043691	1.03991798001662	1.49925979672481
H	-7.37776571606204	0.67374754904694	1.80508361770630
C	-5.00538581107981	0.50665665705294	-3.64363348608237
C	-4.21936014474826	1.77922026375633	-3.99436592241518
H	-3.26765571473918	1.51488991671668	-4.49766104612513
H	-3.97872262598308	2.36543788910015	-3.08586021143940
H	-4.77944954840306	2.44068694179061	-4.68708716116273
C	-5.38729396387599	-0.25101414770552	-4.92879899850919
H	-4.32786690596887	-0.14406338132091	-3.05459204468731
H	-6.04746487768105	0.37167995507569	-5.56820315232774
H	-5.93496358104610	-1.18866435133504	-4.70922602630395
H	-4.48424732880778	-0.51049248602288	-5.51909742225543
C	-8.95909690338478	3.49863610777441	-2.75920663787777
C	-8.28006032249954	4.70761221591567	-2.08444138505260
H	-8.33092338498300	4.61269668393561	-0.97929819901902
H	-8.77544788413604	5.65884399460853	-2.36999247124317
H	-7.20910560415518	4.77690432251452	-2.36294480668386
C	-10.45543723554191	3.45210425767511	-2.42847606202935
H	-8.85897832265214	3.63336474359229	-3.85948852706524
H	-10.94509716079171	2.56350926316587	-2.87472798926299
H	-10.96754189540034	4.35746713419264	-2.81220174682575
H	-10.63085977377815	3.42130230022986	-1.33309511950347
C	1.54197355278611	-2.87146930808264	4.14227805751259
C	-0.60403767438741	-1.83467577724030	4.77772551011594
C	-0.83659203596669	-3.16831757109372	4.05602982710030
H	-1.28510541912669	-1.02580041035921	4.45026757891124
H	-0.73691435255999	-1.97402977616726	5.87159641914337
C	0.45555254799389	-3.93921197248542	4.35215790992679
H	2.45349979464969	-3.02125954065965	4.75255915373686
H	1.85250719294620	-2.84901262317331	3.07674275244206
H	0.45206655571987	-4.28300212096398	5.40879135519015
H	0.58926417166463	-4.83123634905630	3.71186276158007
H	-0.92699707432395	-2.99897340499746	2.96117871001874
H	-1.75798357536485	-3.69107439994987	4.38121805467513
H	-4.80163136364154	-2.22218142989311	4.17365293157504
H	-5.38841499230860	-3.26164517869790	2.83171520651193
H	-5.96170419587280	-1.57340415486676	2.96924510805628

H	-0.55071398199561	1.01549804151278	2.13381375758376

TS-4b

39 conformers were generated by GOAT, all of which were subjected to constrained DFT optimizations followed by transition state optimizations and frequency calculations.

Electronic energy (a. u.) | DLPNO-CCSD(T)/cc-pVTZ: –5199.14595863599
Thermochemical Corrections (a. u.) | PBE-D3(BJ)/def2-SVP: 1.919054203187
Imaginary Frequencies: one (–616.82 cm^{-1})
Gibbs Free Energy (a. u.) –5197.226904432800

248

C	-0.21085067030366	-4.51808058887981	0.83987959810129
C	1.43131084555585	-5.67162577705448	-0.72121489329549
C	2.64203631827935	-5.61809419457663	-1.50069030834452
C	3.32873070773485	-4.37961654402381	-1.62624149901421
C	2.85055453429558	-3.20392214781078	-1.05939027878684
C	1.67473193075738	-3.27855822912664	-0.24584598338140
H	4.25265211300764	-4.34403714259829	-2.22370310975907
C	-0.34789158831282	-5.45233287794988	1.92763271629598
C	-1.28022136982251	-3.66038007227446	0.55911946126987
C	-1.62291654748271	-5.57311906043996	2.59034899446797
C	-2.72041417768373	-4.78023896372441	2.14479173444967
C	-2.57346290778967	-3.81527444894828	1.15789179370554
H	-3.71041473815015	-4.95180155792420	2.59261785323254
O	1.25058128852156	-2.13959239801635	0.39758071877348
O	-1.13048797788025	-2.69074119961507	-0.40971424467944
P	-0.18609054072695	-1.35208935279519	-0.04721698977710
O	-0.65439571658652	-0.61716160135748	1.19079666363433
O	0.02774143573768	-0.63402277718195	-1.37664433567145
N	0.36724447278642	1.27952020367234	4.54889577556815
C	-0.08687399984456	2.37945858477934	3.89936904752024
C	0.99715978647205	1.42748154033861	5.85838573637471
C	1.98510812258340	0.28874575303335	6.09401515056558
H	0.21542505100907	1.39556134933699	6.65473352313877
H	1.50470194351766	2.41182667758290	5.90993884729582
O	1.35066037335172	-0.96199156853182	5.96971892876849
H	2.39256846649838	0.34785597475634	7.12351795303521
H	2.83980220318274	0.39433322225952	5.38101439445614
C	0.79391266014030	-1.19094225418563	4.64952357373430
C	-0.17275477398654	-0.05833673673789	4.27923220094214
H	-0.43023135220658	-0.15955316915542	3.20738833991324
H	-1.10564462678375	-0.17613081716300	4.87908794048995
C	-0.90497584619865	2.39565053182632	2.78238831095481
C	-1.51807847258528	3.58026593583767	2.22753111768444
C	-1.56612552991864	4.85413038011821	3.05492005187318
H	-2.34189102063687	4.78391808466335	3.84614245702474
H	-1.79694943099027	5.72882727591023	2.41757261522164
H	-0.58861687075986	5.05557724313340	3.53058722895939
C	-2.61802358116437	3.34217819606642	1.24202553534919
C	-2.57069163521182	2.22572477317993	0.36993495420811
C	-3.72124737732124	4.21904199621047	1.13128111736212
H	-1.12496634497627	1.43460834263614	2.29817199291263
C	-4.74487739726258	3.97849788101870	0.20336625006994
C	-3.59263472542649	1.99081939761324	-0.55619598344581
C	-4.68858864023079	2.86322678696031	-0.64779674379191

H	-3.79612928973892	5.09216416924815	1.79419526684844
H	-5.60100461663824	4.66864811859490	0.14884198452557
H	-5.49269965529852	2.66512954441912	-1.37200468153803
H	-1.74449903403006	1.50124018200218	0.42724939657949
H	-3.53632329063858	1.09975066805714	-1.19554011121313
N	0.73790899971676	1.88067759082432	-0.83220017221428
C	-0.04952062701223	2.82222345961891	-1.44692307013386
C	-0.17263759148960	4.07470222342402	-0.85280410549345
C	0.39905996115028	4.27520273753927	0.47880581698763
C	1.41580766865871	3.32313633553467	0.92535224095046
H	-0.56640489188414	4.02769020631669	1.28724592786847
H	0.57270396451173	5.31983272973283	0.78633222225886
C	1.51034012336356	2.09254943897320	0.27898824774491
H	0.60661117246313	0.86834771752216	-1.15325972420421
C	-0.69466306374469	2.36631432426566	-2.71606140242242
H	-1.62230837870040	2.92542916029122	-2.91772990246831
H	-0.88530820187581	1.27696700028507	-2.66035953737327
H	-0.01384030718277	2.55074919832586	-3.57408353369843
C	2.39857239052643	0.95676159587744	0.66542517537568
H	3.45814177932237	1.26913227917224	0.63102709613638
H	2.26900239001330	0.09802579903649	-0.01291680474302
H	2.19595515669900	0.63548343562261	1.70260852477981
C	2.17922637471912	3.73619269749647	2.11933263892786
O	1.94383991572987	4.76575343424429	2.74718106296756
O	3.18212498652869	2.88798339310454	2.46639270180451
C	3.96936709194612	3.27829229996634	3.60634209939257
C	-0.96682733073577	5.15290895943059	-1.48207325336765
O	-1.14729898458036	6.19382865898648	-0.61481821694223
C	-1.84964365142231	7.33962140591492	-1.14287699378693
O	-1.40595599156394	5.15917452700644	-2.62435083726815
C	5.03651500219525	2.22437386194241	3.81800220308070
H	3.30190651236006	3.38424925806848	4.48901716189475
H	4.40058176898860	4.28413101243139	3.41765744057943
H	5.67957936913866	2.50102307606981	4.67660499313129
H	5.67935649687641	2.12322487343661	2.92117345614642
H	4.58593957668614	1.23375003069484	4.02805086998053
C	-3.35758354072449	7.18990738360121	-1.02949899687742
H	-1.55065039941229	7.47765162983330	-2.20171782017377
H	-1.47805436578569	8.19438807908093	-0.54391267188148
H	-3.70980332285162	6.32544434858101	-1.62435129971412
H	-3.85404136719060	8.10385942040755	-1.41417682924757
H	-3.67015102882081	7.04220897879983	0.02269147261244
C	3.10362531917666	-6.79297448212006	-2.16432926229174
C	0.69937990213884	-6.89760420476676	-0.70444513778275
C	1.16430806100908	-8.01915710591575	-1.37591985030846
C	2.38500395747213	-7.97622114974712	-2.09992418295754
H	-0.25198520260399	-6.94486308049086	-0.15816344721829
H	0.57617291805778	-8.94908794873536	-1.35184272273722
H	2.74693030296090	-8.87481129061853	-2.62193138587814
H	4.03728025256075	-6.73376813014537	-2.74546559859922
C	0.99401820793840	-4.48487626833712	-0.02843744830837
C	-1.76166103497431	-6.49968301284327	3.66541341213752
C	0.73688385141239	-6.25408729943622	2.39232672968654
C	0.57056406365874	-7.14176439899567	3.44594391137133
C	-0.68990926891003	-7.27260959374165	4.08635654407904
H	-2.74145631535425	-6.58496878650690	4.16093007614511
H	-0.81128622149491	-7.97987748802042	4.92054916447594
H	1.71897210577632	-6.14827388182165	1.91120607972996
H	1.42540670925129	-7.74120794203430	3.79363444039993

C	3.57519872266609	-1.91945924247999	-1.32405702313234
C	4.57170723280910	-1.46542110123645	-0.42029985593355
C	5.34298543613010	-0.34181210040272	-0.76776571335050
C	5.15092383410148	0.34123657320990	-1.98235570898130
C	4.11690662411093	-0.08864653789204	-2.83233382292917
C	3.32212420184409	-1.20988599184284	-2.53043452928208
H	3.95731153803380	0.45129252386926	-3.77730048275117
H	6.13557438934104	0.00795274849183	-0.09000832855325
C	4.84877275337765	-2.21604312575500	0.87843454143401
C	5.24608445067305	-1.29388005247324	2.03924706843982
H	6.25349154949239	-0.85202002000848	1.89266332846631
H	5.27749332757069	-1.86380005745297	2.98980787268775
H	4.52607697821680	-0.46211931745721	2.16135060621978
C	5.91878737178747	-3.30227946682140	0.66052803676159
H	3.90561695114600	-2.72365706459453	1.16949508671142
H	6.10441910423592	-3.86757469233342	1.59716808808717
H	6.87767976220630	-2.84387038777735	0.33903971911479
H	5.61055757985460	-4.02552808198871	-0.11913223008319
C	2.25619030532404	-1.67290340512229	-3.52034980431626
C	1.53186330093266	-0.50743521793724	-4.20857731389601
H	1.16487513992653	0.22341655635344	-3.46709223777223
H	0.65412219820534	-0.88598904717092	-4.77006483088074
H	2.18467777600712	0.01891272406189	-4.93587104989185
C	2.85450252225890	-2.62037693745065	-4.57703773760367
H	1.49381842321684	-2.22830308234684	-2.93741926472545
H	3.29502138995461	-3.52724174739655	-4.12118541231633
H	3.65205938852071	-2.10694050257612	-5.15417963954047
H	2.07241077321576	-2.94780515396251	-5.29259189995545
C	6.08127627530882	1.43565708688766	-2.40528954568599
C	-3.69110652613564	-3.00153716280799	0.58580419841747
C	-4.11859604867778	-1.79588781155342	1.20614377647425
C	-4.22808777866238	-3.38815656545063	-0.66932679306727
C	-4.97438800014594	-0.94393807771240	0.49031150787865
C	-5.14326718578101	-2.53502185139828	-1.31117348205889
C	-5.48250289253457	-1.27893485089758	-0.78277305925557
H	-5.23798260744142	0.03350455595098	0.91319301991042
H	-5.57697905975505	-2.84211473534201	-2.27298559374157
C	-3.69338311310588	-1.45031961094128	2.62970182540002
C	-3.71996342730493	0.04865976247005	2.93772056927668
H	-3.13799264081820	0.62276703316040	2.19684142784447
H	-3.29320923735906	0.24621789627420	3.94121883679664
H	-4.75379178013409	0.45264192317939	2.94531325582933
C	-4.58024194141782	-2.19462707897054	3.64754255852303
H	-2.64653604591355	-1.80235811401732	2.74935718601015
C	-3.80621106660891	-4.67700869743099	-1.37058329406620
C	-5.01286700861750	-5.57302334175536	-1.69401277970279
H	-4.67850351426775	-6.52892570103456	-2.14695147381925
H	-5.69912624549158	-5.08725386436302	-2.41847514699755
H	-5.59854821831702	-5.80864594515003	-0.78265387172006
C	-2.97112000158602	-4.37878648856980	-2.62985735535242
H	-3.15859526210834	-5.24286860422173	-0.67053211669274
H	-2.58652104142579	-5.31929846404200	-3.07597918157600
H	-2.10844836291322	-3.72708296689618	-2.39100885854979
H	-3.58313463356339	-3.86611031510542	-3.40143074916244
C	-6.30075929608187	-0.28595774441229	-1.54329229870546
C	5.70689229539447	2.80250620515507	-2.31282759226419
C	7.35496126084526	1.08479214725553	-2.93050896876484
C	-7.48164208084676	0.26479347209837	-0.96691153257944
C	-5.86236484917249	0.18728345412482	-2.81959152184610

C	6.61153727415122	3.78863535781959	-2.74426186977008
C	8.23003161240953	2.10975815568093	-3.33504346970556
C	7.88100254656440	3.46701570021796	-3.25413675982002
H	6.31359812675957	4.84706429275043	-2.67479165158698
H	9.22118899893034	1.84460725030630	-3.73807852713386
C	7.79183342885529	-0.37257337708541	-3.05951057683982
C	8.31254145847742	-0.70653386215574	-4.46637233946458
H	8.52776880515908	-1.79139569342754	-4.55351652730635
H	9.25286267678436	-0.16622316365549	-4.70240091571493
H	7.56794562758030	-0.44081630372357	-5.24331750591249
C	8.82311415672565	-0.74027133550996	-1.97852593324428
H	6.89568535920117	-1.00203274776466	-2.88670618118235
H	8.41878032898461	-0.56421985975457	-0.96167431627055
H	9.74623904114410	-0.13238235088228	-2.08264922500909
H	9.11028176266155	-1.80986434739821	-2.05190773978794
C	4.34627956926890	3.22453932460216	-1.76629770386473
C	3.43179386743539	3.75260319421478	-2.88550215446134
H	3.86504730592197	4.65428146673441	-3.36666029398726
H	2.43438088025596	4.03067047867028	-2.48557999526208
H	3.28473731942253	2.99014454473854	-3.67657462214702
C	4.47733483084253	4.24351959977691	-0.62292956265983
H	3.86604245375748	2.31476226593503	-1.35419105728176
H	3.47778790965184	4.52819297668689	-0.23938974605657
H	4.97901148143506	5.17441838090454	-0.95821123214912
H	5.06482810192972	3.82962097944393	0.22136251703792
C	8.84870183023216	4.55229146733588	-3.70513227136576
C	9.26168447455522	5.46403308870188	-2.53716520429913
H	8.38973638245211	6.02225817152549	-2.13690247476719
H	10.01550476246995	6.21056731932551	-2.86240821606160
H	9.69385119521626	4.87652579889678	-1.70266307653644
C	8.28245877942675	5.36811741827715	-4.87929913374342
H	9.76436894390727	4.03478552116167	-4.06682025148868
H	9.02235948308950	6.11271062820559	-5.23908114848786
H	7.36968143937749	5.92367328354250	-4.57909230058167
H	8.00986509297378	4.71236802769791	-5.73015851523865
C	-8.16507498810341	1.29127484188344	-1.64465577061094
C	-6.58715222876556	1.21244649758326	-3.45274689692378
C	-7.73158333572157	1.79322120807593	-2.88023330741016
H	-9.07315209044488	1.72217527383190	-1.19124061198239
H	-6.22918510022942	1.58596070452141	-4.42434274310347
C	-8.05816844240778	-0.23572302576939	0.35585723279859
C	-9.48295164365688	-0.78744306464328	0.17603633894745
H	-10.19393449335042	0.00268690126468	-0.14355460384914
H	-9.86143544271856	-1.20925283347262	1.13003492230367
H	-9.50783403147120	-1.58978758781342	-0.58816115324193
C	-8.01343563124315	0.84995020875350	1.44475425149235
H	-7.42609182568706	-1.07839171549676	0.69805309067202
H	-8.35696423345881	0.44559341798487	2.41944848473508
H	-8.66934357952685	1.70825124963126	1.18852594915839
H	-6.98823425517810	1.25019038139387	1.58013439018032
C	-4.62716609023176	-0.36616681492531	-3.53235850101719
C	-3.72616459913937	0.72846346104022	-4.12954450696075
H	-2.76711315115757	0.28823192636923	-4.47099030928632
H	-3.49292257780472	1.51829403638208	-3.39081755093124
H	-4.19088057879826	1.21923886838778	-5.00961293908584
C	-5.03318403380168	-1.36375548740711	-4.63379740450602
H	-4.02266627606408	-0.91822827181952	-2.78387128212103
H	-5.63477512037757	-0.85696458453734	-5.41713501626016
H	-5.64677338059087	-2.19577704931970	-4.23612207738059

```
  H   -4.13673878835255     -1.80046758235734     -5.12053715990917
  C   -8.44427391281478      2.96248601332274     -3.54379448241241
  C   -8.81048641183103      2.68124655202505     -5.00894077401964
  H   -7.90462954124212      2.55240446629225     -5.63681826049848
  H   -9.39074972714087      3.52336504228977     -5.43862704457026
  H   -9.41786730135261      1.75898592276257     -5.10293595153294
  C   -7.60886800785422      4.24958917238029     -3.41189542123015
  H   -9.39226313112368      3.12069765321100     -2.98369494592708
  H   -7.38856380591897      4.47783507746578     -2.34928086292302
  H   -8.14169963212536      5.11979125571179     -3.84836371892513
  H   -6.63687988185768      4.14818422103860     -3.93844521071582
  C   1.91315179592566     -1.47567628432331      3.62114446384694
  C   0.07259465226236     -2.54231699667389      4.73707347803784
  C   1.22605186917310     -3.54955378854424      4.78012442053391
  H   -0.53230642234264     -2.67754515448204      3.81500260206552
  H   -0.60595905590529     -2.58297891428931      5.61243505415044
  C   2.23870236792549     -2.98877349511401      3.75429563515724
  H   2.79565936319182     -0.82725559501462      3.78319725021842
  H   1.53114056233261     -1.25610795086339      2.60716074727901
  H   3.28613870486308     -3.17146872734932      4.06658237206271
  H   2.10677435841047     -3.48438914538470      2.77130802081125
  H   0.90558997184735     -4.58326894857103      4.55147461518715
  H   1.66646695080129     -3.54315130274522      5.79692825358853
  H   -4.56611255630102     -3.29039325367579      3.50050098483586
  H   -5.63458205406972     -1.85868869234722      3.55604227615587
  H   -4.24427804952648     -1.98584629865438      4.68473512398667
  H   0.23650074426053      3.32869635576850      4.35673057760566
```

TS-3b

251 conformers were generated by CREST, all of which were used for constrained optimizations at the GFN2-xTB level of theory. Subsequently, the number of conformers was reduced to 102 by RMSD calculation and cutoff following RMSD ≥ 0.20 Å^2. All 102 conformers were subjected to constrained DFT optimizations followed by transition state optimizations and frequency calculations.

Electronic energy (a. u.) | DLPNO-CCSD(T)/cc-pVTZ: –5199.14153903724
Thermochemical Corrections (a. u.) | PBE-D3(BJ)/def2-SVP: 1.916419816326
Imaginary Frequencies: one (–601.04 cm^{-1})
Gibbs Free Energy (a. u.) –5197.225119220910

```
248

  C   0.09422807874680     -4.60800276859242      0.73958862658690
  C   2.20930147340619     -5.69618050810399     -0.17132035135697
  C   3.58783347690148     -5.57113902538602     -0.57291896453502
  C   4.17556422997305     -4.27812278274923     -0.63465851865661
  C   3.45491297446671     -3.12162323614265     -0.35959878254175
  C   2.10334784522949     -3.26102922790282      0.09331151331645
  H   5.22916331629789     -4.18890647547784     -0.94148579191239
  C   -0.26324665292869     -5.45034144792250      1.85215711929401
  C   -0.91402038736349     -3.87001117754379      0.10867863873195
  C   -1.65816145679465     -5.62444953189714      2.17472295170899
  C   -2.64182618439283     -4.92013795541138      1.42527905259852
  C   -2.30129200481210     -4.01000603588747      0.43060937821620
  H   -3.70356448621659     -5.07703071790734      1.66848492435934
  O   1.41917652599341     -2.12527800396372      0.46804324898848
  O   -0.56617394589572     -3.00207105691019     -0.90570029285617
  P   0.13013692375411     -1.54858635445633     -0.47024242821983
```

O	-0.74587776232116	-0.75482362345448	0.47644337914684
O	0.67309386607486	-0.94513685188905	-1.76300868191393
N	0.00805142640496	1.04660759653443	3.43366487240609
C	-0.70905041822397	1.57073343057758	2.41268562297851
C	0.35941017900336	-0.37603324207920	3.45906815663059
C	-0.40649388391962	-1.05400674757296	4.59603410218332
H	1.45230338142302	-0.47142041871767	3.64101909495793
H	0.11248918826833	-0.83051273312985	2.47930750980473
O	-0.19177079014951	-0.39795743777117	5.83992939887057
H	-0.05775057623059	-2.09839682139211	4.72216278258460
H	-1.49150948249923	-1.08338264360223	4.33918361150810
C	-0.56368096686918	0.98717873850107	5.83228249508366
C	0.22865016730216	1.70179022350406	4.71168005399297
H	-0.04824514968193	2.76986766794488	4.65186656655457
H	1.31129185836628	1.64186694183922	4.95944458114135
C	-1.30996534424295	2.82215224926961	2.36428133030230
H	-0.80529324027502	0.86823097259448	1.56494858627361
C	-2.07586507339731	3.30085716131972	1.23407876964950
C	-2.64131829111499	2.29862480437078	0.24418229906547
H	-3.55594248885114	1.82222236302848	0.65067500493562
H	-1.93409750401181	1.47881666756090	0.01623264070946
H	-2.91849132633073	2.79371890261660	-0.70443096588050
C	-2.88662235762503	4.54379307436520	1.48527037341837
C	-4.24312939617811	4.62920535191711	1.10552584827369
C	-2.29057831036829	5.66251650668685	2.11325660761481
H	-1.19569228678987	3.51851914524112	3.20747539853079
C	-3.04113145509140	6.81347042414026	2.38496846498403
C	-4.99405970895212	5.78185984953110	1.38271813210030
C	-4.39941340010477	6.87616367552750	2.02926768592726
H	-1.21572320763201	5.63647893511110	2.35520070032181
H	-2.55713709844145	7.67588382643390	2.86832379692540
H	-4.98846265163916	7.78076345680393	2.24355804543013
H	-4.72816547012797	3.77940960092643	0.60477193957329
H	-6.05476273729381	5.82178888866782	1.09120578624780
N	0.84275676923703	1.67688874376209	-1.30555881112172
C	0.03427087693525	2.42043557893782	-2.13304489876281
C	-0.39782346983815	3.66588632165172	-1.69105137225259
C	-0.06301199451539	4.08828878852195	-0.33263837092050
C	0.96814780001406	3.34447633530394	0.37809554063613
H	-1.12666920891105	3.83800647952345	0.41896116762326
H	-0.08933112892775	5.17169670570422	-0.12746593572253
C	1.34537702300596	2.08776983548779	-0.10466038677624
H	0.93653887864480	0.63792679278823	-1.55239476512391
C	-0.27536769077581	1.76477234311335	-3.44285881318005
H	0.58550966298277	1.89300194640980	-4.13370005014543
H	-1.15195069421365	2.22288497399585	-3.92741130504465
H	-0.40777820171804	0.67505500037160	-3.28910574251861
C	2.27025881360613	1.12451643156602	0.56394970433610
H	3.27036572921885	1.57292445967652	0.69927079738283
H	2.37771625183003	0.19823798907300	-0.02314582741196
H	1.90881474219071	0.88206831715586	1.57830034557923
C	1.46317126128626	3.98549032125904	1.61411637901436
O	1.07355185358259	5.08166075344171	2.00805537767180
O	2.41540176612812	3.27420418205269	2.27088084760137
C	2.98601020031040	3.90580921764822	3.43282131394957
C	-1.31263074254289	4.51034088927894	-2.49889205715959
O	-1.82828921418207	5.50570551191682	-1.72962385696169
C	-2.80230062387426	6.36701991889022	-2.35484296432833
O	-1.61747222113070	4.35405966642847	-3.67451698473853

C	4.06437618763296	2.98878473143470	3.97153883000482
H	2.18080923572878	4.09228119961735	4.17559030232239
H	3.38314367299686	4.90044745706753	3.13969810399560
H	3.64547146628255	2.00415759042117	4.26030225959932
H	4.53811098409966	3.44058657515291	4.86511329997109
H	4.85184338126071	2.81227423914849	3.21168392234353
C	-2.98549611947689	7.58335609992757	-1.47252721856609
H	-3.74728097155508	5.79217964975533	-2.46968694835987
H	-2.45179420582732	6.62093365669394	-3.37641762956423
H	-3.74864820987531	8.25540343443443	-1.91305431146820
H	-2.03820323411617	8.15076682886142	-1.37829405213598
H	-3.32100933487726	7.29329796585958	-0.45804935483376
C	4.32262492230401	-6.73794075466628	-0.93686138230538
C	1.61179995471081	-6.99188326336704	-0.22253222784552
C	2.34792623762759	-8.10623714250524	-0.59819514752739
C	3.71996696940756	-7.98580117188304	-0.94357397386653
H	0.54887783017119	-7.10127999720590	0.03083910320718
H	1.86017728625141	-9.09214064126061	-0.63496910263831
H	4.29487747074491	-8.87850847815505	-1.23255725250370
H	5.37819441799013	-6.62279967279011	-1.22922027740014
C	1.48829527940653	-4.51461137767772	0.23217135919038
C	-2.02116081489175	-6.47842708664508	3.25815994036895
C	0.70673670661892	-6.10016546254679	2.67240056366697
C	0.32126020029237	-6.90958900505605	3.73152715320081
C	-1.05394386111017	-7.11399704835503	4.02124088070303
H	-3.09062994237564	-6.61018022623983	3.48574164402937
H	-1.34791450487336	-7.76473850189225	4.85858701183640
H	1.77322912478035	-5.94441573663914	2.45834541763688
H	1.08859738177910	-7.39408827041795	4.35421158128759
C	4.12463400004405	-1.79180038139711	-0.53206025262915
C	4.77496136411793	-1.18193069957018	0.57260035072244
C	5.53823639896880	-0.01885430956985	0.35992911672325
C	5.67499907724331	0.55230051473593	-0.91887952340905
C	4.97336674578215	-0.03620804140295	-1.98605964560166
C	4.19534501022789	-1.19702474052364	-1.82227078560762
H	5.07111909696856	0.41136352446678	-2.98582158206025
H	6.06909576065587	0.44698453053520	1.20417953052725
C	4.71676185701706	-1.81209794688142	1.96074392184174
C	4.48470900689434	-0.78562616284093	3.07880983895080
H	4.33694604813435	-1.29801369527511	4.05133439130019
H	3.58866125380320	-0.16717471960131	2.87564716302929
H	5.34799372721245	-0.09930048661857	3.20025736474041
C	5.98485522664999	-2.64351172925828	2.22607492465482
H	3.85409947911298	-2.50942329327154	1.96910519961209
H	5.94386509070659	-3.12421158450627	3.22539085410570
H	6.89078240351905	-2.00249466146200	2.19008844988216
H	6.10679707807857	-3.44236464249298	1.46787457603064
C	3.49823064005649	-1.81675310677036	-3.03063085350321
C	2.93170742796154	-0.76625786386994	-3.99711690514809
H	2.29393736644318	-1.25794292547945	-4.75868374135613
H	3.73329614611774	-0.22392321354702	-4.54071893096916
H	2.30518559049212	-0.02941855546913	-3.46258666071769
C	4.44135306468843	-2.77499676946004	-3.78121601484613
H	2.63726386962994	-2.39846652024422	-2.64446253739862
H	4.79137368600478	-3.60201527559761	-3.13434355723453
H	5.33580154380146	-2.23274146271863	-4.15377895217332
H	3.92618329711699	-3.22445171389588	-4.65497688656306
C	6.60975866023909	1.69523015738739	-1.16747800808608
C	-3.33072526931852	-3.18041654095284	-0.26381055389681

C	-4.09684005092068	-2.23748389427026	0.47950015606217
C	-3.55274893450715	-3.33176830306767	-1.66044046147286
C	-5.06575891763246	-1.47091910234310	-0.19191482146810
C	-4.54525500635948	-2.55376394202084	-2.28107930600977
C	-5.31422256716191	-1.61948713828586	-1.56693757660252
H	-5.66896247135496	-0.74151397556795	0.37075368517766
H	-4.75114235629934	-2.69454732350948	-3.35315735718121
C	-3.93277671259275	-2.04119577773806	1.98474350326652
C	-3.69500926643498	-0.57164926488418	2.35519886965012
H	-4.56304608688855	0.07042238458496	2.09844405283691
H	-2.80458171070839	-0.18979183352966	1.82471161888693
H	-3.53064235068218	-0.46660579780709	3.44735865957498
C	-5.13738862869784	-2.61881300361864	2.74892264165407
H	-3.02984962242543	-2.60005666561796	2.30096608736140
H	-4.99600140711685	-2.51791007679981	3.84513774596966
H	-5.29301615747000	-3.69245835446300	2.52093034466990
H	-6.07367862218825	-2.08714712022500	2.47829076402752
C	-2.80156293035497	-4.35859183589043	-2.50274335838598
C	-3.75008094261307	-5.47507689906129	-2.97458593820233
H	-3.19267774758795	-6.25047672334517	-3.53952485108758
H	-4.54313799520953	-5.07815300798816	-3.64217766669236
H	-4.25006482645382	-5.96709123575304	-2.11587297238848
C	-2.06100064002727	-3.70366548578372	-3.67994154257992
H	-2.03861821103339	-4.83482876359919	-1.85672885837821
H	-2.76749167162999	-3.23598410803118	-4.39743207240346
H	-1.47562154644823	-4.46231296347764	-4.23915687920366
H	-1.36003216191638	-2.92543340076267	-3.32141463623445
C	-6.41845287067255	-0.85671025119713	-2.23012419334369
C	6.12754534997537	3.01111816846116	-1.39328412401246
C	8.00908283152456	1.43767466325936	-1.21374295299688
C	-7.74068982419125	-1.37493728228543	-2.18102443921397
C	-6.15962925649189	0.37052500744784	-2.89574781689593
C	7.05230446173359	4.03878180507767	-1.65913143221223
C	8.89164016263829	2.50119168420509	-1.46920752212912
C	8.43631094780945	3.81126867837045	-1.69836819373285
H	6.68301390933222	5.06185672807843	-1.83820043354445
H	9.97370312282425	2.29560651723128	-1.50071780845298
C	8.57362953319676	0.03455785993945	-1.00028369128101
C	9.48537276243106	-0.40558322237372	-2.15700239266504
H	8.95772048705866	-0.34131893401529	-3.12967420988556
H	9.81703128104521	-1.45458896748520	-2.01415697729261
H	10.39791426460127	0.22201221962805	-2.22742776756315
C	9.29254781360883	-0.07551986723821	0.35508280320042
H	7.71680733551187	-0.66807470233265	-0.97531239837017
H	8.61405355797727	0.18560770211839	1.19208764945448
H	10.16392936652985	0.61054630110115	0.40631058436628
H	9.66396420365193	-1.10758700958445	0.52447104130596
C	4.63793304422631	3.33908938314214	-1.35437570895171
C	4.07403216813816	3.59848524571404	-2.76233853984399
H	4.56422119252985	4.47436065383159	-3.23655878789799
H	2.98375138775448	3.80439679954838	-2.71824136785134
H	4.23117538659346	2.72582025908600	-3.42719577436959
C	4.33051865717789	4.51136175927447	-0.40900439321730
H	4.12090041698659	2.44289075015291	-0.95958167546286
H	3.23976408103447	4.70052979508961	-0.36332495229822
H	4.81049299505651	5.45103099770870	-0.75103969569494
H	4.69064508547645	4.30543306667257	0.61895100919362
C	9.40745848079069	4.94446363070367	-1.99856228570161
C	10.10826606451868	4.73560939866089	-3.35206785607041

H	10.73344066288832	3.81853074866019	-3.33939280993323
H	10.77251159656611	5.59126903820438	-3.59292200207654
H	9.37230940345660	4.62684481062848	-4.17350026773692
C	10.42869052061939	5.14353928103092	-0.86641387543930
H	8.80167138828698	5.87423097455138	-2.07340305039346
H	9.92513146929907	5.31454836587210	0.10600610101313
H	11.08528378418392	6.01328801077552	-1.07446769771835
H	11.08211402739168	4.25349680586361	-0.75411672785279
C	-8.78029384133773	-0.65232020516409	-2.79209485913855
C	-7.23315395646154	1.05172819059562	-3.49949468564865
C	-8.54906140849275	0.56274500868910	-3.45904619691120
H	-9.80481582957793	-1.05487977917902	-2.74588990744417
H	-7.04419486424276	2.00272621167532	-4.02203634938907
C	-8.05267672392977	-2.67895666915002	-1.45154774883861
C	-8.87703888275748	-3.65265227453758	-2.30738694973976
H	-9.00638717069703	-4.61999165464306	-1.77994912125935
H	-8.38062304346718	-3.85442802188264	-3.27779649718364
H	-9.89205049979479	-3.25923167692579	-2.52392235510523
C	-8.73667784631076	-2.39858685450581	-0.10185051950054
H	-7.08437373015745	-3.17275515989028	-1.23222047396244
H	-8.93033431847068	-3.34198125102647	0.44979578587530
H	-9.70874821350137	-1.88192315520650	-0.24605230106891
H	-8.10468068362777	-1.75148779833321	0.53903881305242
C	-4.74020671617592	0.92178562718568	-3.00005516172866
C	-4.67179832208113	2.45196698446311	-2.89115624872613
H	-5.16115265276874	2.95256371639146	-3.75170387674677
H	-3.61861841809742	2.79424942755498	-2.89569119201940
H	-5.16032476643390	2.81850536433553	-1.96540207428026
C	-4.06608978708491	0.44153113152986	-4.29919617884018
H	-4.16322681805876	0.49459509028738	-2.15189499417129
H	-3.02091421089211	0.80724711193910	-4.35937557527834
H	-4.61436706186444	0.81797751535316	-5.18779532215468
H	-4.04181575101702	-0.66370898510372	-4.35769182090914
C	-9.68969499098669	1.33790093815269	-4.10449691108923
C	-10.66729914555962	1.87807280715617	-3.04619331757538
H	-11.15870517235296	1.04748172021920	-2.49780106910378
H	-11.46579325232634	2.48944429250348	-3.51548637722799
H	-10.14290436209979	2.50709790873801	-2.29932837129299
C	-10.42318888913280	0.50576051068488	-5.16868250327299
H	-9.23333333656806	2.21300969310273	-4.61749338160896
H	-10.92320808833943	-0.37582832182949	-4.71613390596952
H	-9.72251227643166	0.13242567716397	-5.94195268694789
H	-11.20646373308675	1.10861513640887	-5.67285543646122
C	-2.09345971634559	1.21033904962861	5.67174125700428
C	-0.21438242017111	1.58582467746196	7.22214967154289
C	-1.52179595043462	2.17776473162238	7.78966623737299
H	0.56869150974818	2.36619304580222	7.11766308237651
H	0.21342079484273	0.78278632693116	7.85221812477716
C	-2.39006018889546	2.43259165051492	6.54735767625218
H	-2.60297359516103	0.31571733881991	6.08860439434736
H	-2.41894484012679	1.32540057430335	4.61943566859225
H	-3.46775249222166	2.55440088403721	6.77508149634128
H	-2.06595812686124	3.36282130566114	6.02985535666242
H	-1.35675740498879	3.08274526693883	8.40739198834788
H	-2.02516022901963	1.43056555627441	8.43853225202190

TS-4c

329 conformers were generated by CREST, all of which were used for constrained optimizations at the GFN2-xTB level of theory. Subsequently, the number of conformers was reduced to 62 by RMSD calculation and cutoff following RMSD ≥ 0.50 Å^2. All 62 conformers were subjected to constrained DFT optimizations followed by transition state optimizations and frequency calculations.

Electronic energy (a. u.) | DLPNO-CCSD(T)/cc-pVTZ: –5199.13908137966
Thermochemical Corrections (a. u.) | PBE-D3(BJ)/def2-SVP: 1.917406181065
Imaginary Frequencies: one (–724.80 cm^{-1})
Gibbs Free Energy (a. u.) –5197.221675198590

248

C	-0.51336830912119	-4.53605319174477	0.81413541408959
C	1.28239683705291	-5.92994922385491	-0.32878451608226
C	2.60257896358686	-6.01552271583651	-0.90024488000213
C	3.36887511867203	-4.82773809228438	-1.04885466102643
C	2.87463685033309	-3.57474945870303	-0.70277494151210
C	1.57570752823440	-3.50498968712038	-0.10113494018059
H	4.38233562104901	-4.89920486551549	-1.47336681751594
C	-0.84124409295830	-5.30603610927882	1.98684248351149
C	-1.47941882734575	-3.66816266134783	0.29279564573367
C	-2.19380667785380	-5.27628934848331	2.48635438309464
C	-3.16067571792578	-4.46015301475241	1.83443137293475
C	-2.82603528329657	-3.62423555773255	0.77578949616283
H	-4.19707383416313	-4.47083307195306	2.20335024339064
O	1.11393051037870	-2.28193186584744	0.33153704571214
O	-1.13942818840440	-2.84822797759048	-0.76451665190494
P	-0.20555173660241	-1.50693383219109	-0.40329900324709
O	-0.83351156445358	-0.64023575657088	0.66778817943172
O	0.23109741573877	-0.91950693957589	-1.74183776335832
N	0.48145209590935	0.80247799319961	3.57889485570907
C	-0.22655726300430	1.62599809886260	2.77881345614619
C	0.31316052101891	-0.65233568202457	3.52009154612162
C	-0.31096223246091	-1.13420721974034	4.82817708105224
H	1.31622792407904	-1.11401184049134	3.40183751139571
H	-0.29618538579327	-0.91541152503063	2.63235638905739
O	0.44243418523258	-0.70818064149648	5.95673417348813
H	-0.32575141289820	-2.24225867080122	4.85409275212945
H	-1.36680295735014	-0.77631729782246	4.89172070636827
C	0.60419646707147	0.71386515289652	6.03705551405255
C	1.26462698322454	1.21949649737949	4.73214416850507
H	1.38129912167075	2.31685941587350	4.74924079462285
H	2.28085967725428	0.78099422328374	4.64348245454245
C	-0.27285184294249	3.01589205796615	2.85126305393711
C	-1.01520944552507	3.92380290622851	2.00340695756295
C	-1.04249415604808	5.35735591774675	2.52168559662132
H	-1.34304899682187	6.06706046040646	1.72794330982876
H	-0.03596585078451	5.64793770218371	2.87863551787232
H	-1.74756283336308	5.46281685677824	3.37301250168461
C	-2.21125004771442	3.48864214338608	1.20734713089131
C	-2.17096232197320	2.37396110973470	0.33704646947241
C	-3.41940465897859	4.22224926001301	1.27710169832659
H	0.34995686718262	3.51352607942792	3.61030969568920
C	-4.54046154647427	3.84879695211570	0.52379921390840
C	-3.29097465001268	2.00470716207690	-0.41953776929628
C	-4.48246952431513	2.74042926514734	-0.33535995774293

H	-3.49510298041074	5.09149104429163	1.94498773899861
H	-5.47148083385214	4.43020836511799	0.60899154374512
H	-5.36152992796894	2.44321672972480	-0.92578009836017
H	-1.27523460389859	1.74738331656436	0.25211801262208
H	-3.22744532685128	1.11116924506071	-1.05670971410389
N	0.94422482686661	1.60679308453888	-1.16026657281009
C	0.19622028611809	2.55748581045899	-1.80635910129396
C	0.14436930975595	3.84063107679961	-1.26862097394886
C	0.79195639750668	4.11183836532551	0.01444731819571
C	1.68785789872737	3.08668171744277	0.54442948858742
H	-0.15885754905566	4.11215536197593	0.92736754710202
H	1.08866706924254	5.15871281824820	0.19034506149363
C	1.69229316439186	1.81792036127963	-0.03623447870719
H	0.80037502518173	0.59489704782978	-1.47639146480470
C	-0.50880069768056	2.07703586194460	-3.03567154162621
H	-0.54200259415281	0.97076312493356	-3.04043697261065
H	0.01722745794423	2.43160193945221	-3.94564785583098
H	-1.52734663769728	2.49834001961687	-3.09211560667210
C	2.45735821406962	0.64408688020800	0.46750910920099
H	3.48785692915989	0.92740740237161	0.73982918465561
H	2.47262404872126	-0.17193571206105	-0.27271037629180
H	1.99584538942224	0.26894638434980	1.40022586846941
C	2.50939394114838	3.37137131428658	1.73880918460694
O	3.19461130015624	2.57936226471410	2.37555395255680
O	2.44229580999050	4.69860453776940	2.07099090475495
C	3.23884127036382	5.09616856580870	3.20081379037159
C	-0.66621706578587	4.89291681700441	-1.91798284218915
O	-0.79937671875999	5.97909965607046	-1.09783192436724
C	-1.59979674404306	7.06291212469430	-1.61779134190531
O	-1.17279075236877	4.83434352054939	-3.02997503752573
C	3.11804836102230	6.59845951884782	3.35441755844078
H	4.28749808615749	4.77166617928198	3.03345626520751
H	2.88229953695464	4.55273794138405	4.10382969411388
H	2.06815773112957	6.90231258120252	3.53959286675171
H	3.47184221133686	7.11880263888155	2.44244253247882
H	3.73176400347645	6.94337551096665	4.20988969883930
C	-3.08231901328232	6.83461950584248	-1.37240880707325
H	-1.39259327934757	7.16651709319168	-2.70214390368669
H	-1.22996231146375	7.96274856295767	-1.08772855825020
H	-3.29817300154450	6.71066425660312	-0.29341862880931
H	-3.43034485198215	5.92630585280364	-1.90108507915396
H	-3.66419551954168	7.70028806370605	-1.74874424381988
C	3.10006220590735	-7.27980070865296	-1.33408330389918
C	0.49567203447979	-7.12044285661338	-0.27753475242377
C	1.00133570759274	-8.33309736863241	-0.72302745975614
C	2.31980506518402	-8.42144257270058	-1.24304653275777
H	-0.52978972804193	-7.06767179024257	0.11198334362004
H	0.37053508055343	-9.23388502051185	-0.67857688006467
H	2.71159271347192	-9.39061096564142	-1.58687066618476
H	4.11525611492223	-7.32570936877584	-1.75856574948832
C	0.80368086947267	-4.65240985069763	0.13570881932673
C	-2.52846179346393	-6.04994589088772	3.63705344565509
C	0.12669657270902	-6.07438335288299	2.70008217488402
C	-0.22691781476885	-6.80121823157720	3.82799590479473
C	-1.56778416152187	-6.80163418312717	4.29593853464957
H	-3.56771866948862	-6.02576702736562	4.00084074212890
H	-1.83819274336492	-7.38814155906435	5.18698106558537
H	1.16760298046881	-6.07535210347933	2.34787289516382
H	0.53973102681160	-7.37881673138900	4.36640904910381

C	3.72369519392697	-2.36437145659852	-0.95201682247251
C	4.67578006169839	-1.95300351441108	0.02140624611344
C	5.53566231073589	-0.87950349624152	-0.27886855713505
C	5.49566226750256	-0.22212719587319	-1.52162354494204
C	4.52919981157066	-0.63001620025828	-2.45756480479280
C	3.64372955171236	-1.69366594204332	-2.20464516673191
H	4.50647536823979	-0.12939329438953	-3.43617916346095
H	6.28771927172369	-0.56193343417578	0.45790304571202
C	4.77001784704438	-2.65287301935535	1.37528179431016
C	3.85276893909617	-1.97441760539463	2.41013589196338
H	4.16810197699619	-0.92471849852317	2.58824668756073
H	3.88726698737562	-2.51202780229338	3.38048256529316
H	2.80413936595022	-1.95924451592948	2.05702551503753
C	6.20701886253481	-2.76744627425462	1.90303029740966
H	4.38803471543625	-3.68504974065086	1.23099324712793
H	6.23296786162770	-3.40206635532551	2.81201948930507
H	6.62496316551862	-1.77953633469709	2.18737829883995
H	6.88547259242776	-3.21656278935408	1.14976670413449
C	2.68890598775830	-2.15708741442661	-3.30150882176793
C	2.18993521393342	-1.01353128726162	-4.19598639299108
H	1.80198926066944	-0.17121888121584	-3.59457161947530
H	1.36374281912785	-1.37165558069466	-4.84199954329315
H	2.98842572461782	-0.63119290134579	-4.86559792235191
C	3.34507120310245	-3.25230488893784	-4.16385314395646
H	1.79915134964227	-2.58706240500532	-2.79969602757078
H	3.62991396276588	-4.13685818305529	-3.56306405927575
H	4.26190433858248	-2.86376674936514	-4.65501703619838
H	2.64914760352582	-3.59276949648936	-4.95811138141771
C	6.53683341932642	0.78325431763530	-1.90976170676254
C	-3.81431937141445	-2.69385495323299	0.15383874668612
C	-4.31599344139669	-1.58780207010415	0.89596281391405
C	-4.24331619678952	-2.90112353380768	-1.18480864036651
C	-5.19837633631731	-0.69725000926531	0.26412184777769
C	-5.17427833516674	-2.01264834240068	-1.75013601560849
C	-5.65059389356473	-0.88696370186511	-1.05674983188051
H	-5.54286308953543	0.18908599249995	0.81395056557925
H	-5.54109388769507	-2.20455510003293	-2.76830073558441
C	-3.95820471579416	-1.34928328392514	2.36118012481565
C	-3.57319083524760	0.10622059538298	2.65492801454452
H	-4.43225240673339	0.80139821837976	2.55536082910026
H	-2.78664568065632	0.44192943990443	1.95821800891221
H	-3.19533151124505	0.20505698220421	3.69385487464551
C	-5.11204388293472	-1.79851478000325	3.27760055475311
H	-3.07228758761856	-1.97448834542295	2.59238829248423
C	-3.75172684557597	-4.07003555875520	-2.03240943080729
C	-4.89845391141992	-5.04142767891490	-2.36151041458976
H	-5.68128844856563	-4.55252629431402	-2.97834416233050
H	-5.38495465506994	-5.41833052725493	-1.43926543264074
H	-4.52016280595561	-5.91381896455030	-2.93321985495189
C	-3.03699585054173	-3.58146777008063	-3.30444359275260
H	-3.01022219560456	-4.63721149645983	-1.43489591654960
H	-3.73307479211217	-3.03911380348914	-3.97832082529798
H	-2.62515635329161	-4.44055806585297	-3.87317618678527
H	-2.20007880382093	-2.90357862896407	-3.04863468457669
C	-6.59108556284549	0.10040990586013	-1.66823345070975
C	6.27276775179384	2.17893974321554	-1.89457931752977
C	7.80417093048098	0.30894437573787	-2.34657030106269
C	-7.82621782486925	0.39200291925280	-1.01953834475028
C	-6.24104731984276	0.81598292923242	-2.85520756651870

C	7.27570919533882	3.06694192641714	-2.32156942393778
C	8.78077346489131	1.24040546533985	-2.74561696325689
C	8.53910816357532	2.62253621374951	-2.74708615661193
H	7.06189194844341	4.14743882801888	-2.31820664243452
H	9.76688896527713	0.87769623639888	-3.07907863573134
C	8.13167251103268	-1.18208905675259	-2.38658770011231
C	8.68635070395012	-1.62424575858265	-3.75013378333490
H	7.99589812823745	-1.34997200868740	-4.57283377305848
H	8.82860386256213	-2.72425402357129	-3.77321120928197
H	9.67107995986008	-1.16126242025729	-3.96857236603454
C	9.08420316936217	-1.56795795129301	-1.24167204819759
H	7.18376211766212	-1.73376584030497	-2.22659991230695
H	10.05446841186306	-1.03586523628004	-1.33108919052626
H	9.29294845968770	-2.65807415408438	-1.24964109373907
H	8.65103655748538	-1.30996274015285	-0.25475164805831
C	4.92325905804687	2.73315027087068	-1.44647967705481
C	4.06587300487443	3.16465780872280	-2.64920611436332
H	3.88895894234118	2.31878719366322	-3.34304342925491
H	4.56433643621188	3.97306566920689	-3.22383833606895
H	3.07823842276353	3.54453985539861	-2.31278963241497
C	5.07250245075286	3.87830223176006	-0.43225487983837
H	4.38730811338411	1.90581707119597	-0.94110737429122
H	5.63872991441370	4.73303691551659	-0.85587221915631
H	5.59933987866148	3.54105778188598	0.48262154950732
H	4.07989982747766	4.26912655515105	-0.13460534737192
C	9.61414520628058	3.60624847423624	-3.18797742247935
C	10.06204100914039	4.50823160834842	-2.02504995862616
H	10.88240241825552	5.18619756802418	-2.33951579318744
H	10.41948156289587	3.90745894265416	-1.16500852668680
H	9.22368078310882	5.14087423115437	-1.66587485787949
C	9.16544430997928	4.43924777940862	-4.40009505453052
H	10.49300899109481	3.00132096610821	-3.50216804201156
H	8.29626957531798	5.08109845273600	-4.14612889628088
H	8.86566679125245	3.78961125182913	-5.24648222946480
H	9.98153834374651	5.10617627437090	-4.74700474897363
C	-8.64633713583291	1.41558779457328	-1.52935834385178
C	-7.10327840352757	1.82253980572166	-3.32349656292019
C	-8.30142441349243	2.15548198432729	-2.66913466334544
H	-9.59686357380709	1.64615548851115	-1.02028435707464
H	-6.81753228360449	2.38095911321436	-4.22832446191838
C	-8.32390640552643	-0.39548757599907	0.19227902811194
C	-9.66285628770185	-1.09267297599829	-0.10515354731218
H	-9.58334074994529	-1.74956648699460	-0.99427492995315
H	-10.47477898285799	-0.36140601053050	-0.29959535361553
H	-9.97685955136859	-1.71730004690773	0.75654434027111
C	-8.42257431311580	0.48434191104814	1.44976822508392
H	-7.58353521385282	-1.19228710245603	0.39996590368846
H	-7.45314233915927	0.96809227641516	1.68557911750889
H	-8.72699850827398	-0.11870089658149	2.33026663687915
H	-9.17276173401273	1.29240287212772	1.32147105717617
C	-4.96331849890935	0.53362924390278	-3.64437420066581
C	-4.20649904402811	1.80704679120721	-4.05615472257743
H	-4.77049284149788	2.40312895314413	-4.80284612222010
H	-3.23903040812276	1.53862883317887	-4.52629599302503
H	-3.99665568684604	2.46525736057685	-3.19049611009933
C	-5.27154807336343	-0.31897890245798	-4.89040662098810
H	-4.28893419823685	-0.05810514632250	-2.99263770905732
H	-5.92273459544825	0.23934066401033	-5.59504060774680
H	-5.79911437226278	-1.25809741824602	-4.63135176637378

```
H   -4.33763477899294     -0.58673790694270     -5.42635749627142
C   -9.18190190027276      3.29298107089904     -3.16506137272681
C   -9.64893911102558      3.07639004080727     -4.61354236031127
H   -8.79091621908154      3.07172790986582     -5.31745522158930
H   -10.33593345753845      3.88677180821048     -4.93327463207099
H   -10.17868901593141      2.10937615064265     -4.72544797610249
C   -8.47310029463202      4.64919469124870     -3.00139979857849
H   -10.08614774673063      3.30544006475889     -2.51747815439333
H   -8.16906951296020      4.81840212687471     -1.94869517001087
H   -9.13411604674057      5.48545149016321     -3.30998255143136
H   -7.55600564145643      4.69668367197178     -3.62484079802030
C   -0.72866457055988      1.46283743444634      6.32073112172415
C   1.50682719040599      1.02378649140604      7.26270046711831
C   0.70064073238512      1.96926778888360      8.17773722043820
H   2.45492783807836      1.49597546436460      6.93042264849053
H   1.77688296763703      0.06712435117966      7.74895878811245
C   -0.32078480181999      2.62397408986180      7.23438770911324
H   -1.38925683690128      0.76317667601325      6.87522847430814
H   -1.26508155291272      1.78068314242267      5.40459564020913
H   -1.17867576907090      3.08833586885692      7.76069529553135
H   0.16369547601347      3.42866752245499      6.63723072098166
H   1.33657642125771      2.69922444200988      8.71689335000642
H   0.15874256367481      1.38106303104762      8.94784208461547
H   -5.38285803081252     -2.86089936007579      3.11683851240373
H   -6.02256262683233     -1.19261017516930      3.08537446962203
H   -4.84018662703263     -1.67208896819003      4.34637235701486
H   -0.78872239918602      1.07518065586514      2.01007961599568
```

TS-4d

948 conformers were generated by CREST, all of which were used for constrained optimizations at the GFN2-xTB level of theory. Subsequently, the number of conformers was reduced to 650 by RMSD calculation and cutoff following RMSD ≥ 0.15 $Å^2$. After constrained DFT optimizations, the number of conformers was again reduced from 650 to 337 by RMSD ≥ 0.20 $Å^2$. All 337 conformers were subjected to transition state optimizations and frequency calculations.

Electronic energy (a. u.) | DLPNO-CCSD(T)/cc-pVTZ: –5199.13885404644
Thermochemical Corrections (a. u.) | PBE-D3(BJ)/def2-SVP: 1.917685659392
Imaginary Frequencies: one (–715.85 cm^{-1})
Gibbs Free Energy (a. u.) –5197.221168387050

```
248

C   -0.23530307789734     -4.49685417164055      0.75214719740913
C   1.56993853921369     -5.79723108474331     -0.47833910588854
C   2.86378667722121     -5.81961155701404     -1.11249225261786
C   3.56625357431739     -4.59714929465410     -1.29153675125061
C   3.03469669624225     -3.36973090609843     -0.91069688977076
C   1.76929560565203     -3.36402779390232     -0.23702609832292
H   4.55758127024154     -4.62048711903453     -1.77036205365498
C   -0.48922192037698     -5.30341275166464      1.91853574927957
C   -1.26054067743996     -3.67118914134077      0.27640732157208
C   -1.82874883961238     -5.36194214711248      2.45035754791982
C   -2.85783682023312     -4.59346362902830      1.83604369191465
C   -2.59660846722575     -3.71853939973443      0.78806748456482
H   -3.88260062909933     -4.66956791196205      2.23001678827654
O   1.27788073633353     -2.16856869519721      0.24054504944651
```

O	-0.99607910843161	-2.80955583394725	-0.76809624648386
P	-0.09919370649107	-1.43841159124012	-0.42734192207308
O	-0.70986537574896	-0.60434298623864	0.68079481883511
O	0.25134420624316	-0.82050680459522	-1.77744517774448
N	0.75779151690378	0.87230392051855	4.10565770803936
C	0.08710728639467	1.97243653812011	3.70432549631364
C	0.74911200070544	-0.41570216529011	3.41612228672176
C	0.14942266400395	-1.48055188759080	4.33258844121113
H	1.80332911367319	-0.68471436947375	3.19460503072105
H	0.19304160004656	-0.37439952429846	2.45886235736019
O	0.81835253325002	-1.52748611850812	5.58748806995130
H	0.26396132655500	-2.48046106415423	3.86849476019926
H	-0.94219951002201	-1.28801757437150	4.46250832758761
C	0.80034811095574	-0.27912142202670	6.29020514587409
C	1.43375558058771	0.81122854274256	5.39043929288669
H	1.37842284553703	1.80050853391133	5.88620941896617
H	2.50538314816651	0.56081471563709	5.22698536096565
C	-0.70544343310727	2.10917848670649	2.57699344096777
C	-1.41379244508188	3.32151729935702	2.22664703057267
C	-1.39791614809143	4.48772765271682	3.20217786907807
H	-2.09903286771650	4.31545858555365	4.04570683448383
H	-1.69000855024573	5.42867491306367	2.69948971737219
H	-0.38481838857066	4.65011799398584	3.61207865912251
C	-2.62379830327356	3.17716231010866	1.35637247146205
C	-2.70318667101778	2.14954380579700	0.38393975857241
C	-3.70909591654042	4.08119239370424	1.45149102613915
H	-0.81024587625218	1.24057674987328	1.91145175075233
C	-4.83078486796693	3.95989427852293	0.62008021471614
C	-3.82160904360978	2.04057058200659	-0.45168587096115
C	-4.89259758755623	2.94039153674390	-0.34408508018914
H	-3.69028036452255	4.88312172703050	2.20201954437035
H	-5.66916331793985	4.66513404824672	0.73053524745586
H	-5.77075449574378	2.83473701878538	-0.99892097712784
H	-1.91224376049495	1.38934191672650	0.29484532582012
H	-3.86370915279965	1.22026857522236	-1.17960412976934
N	0.74060238186064	1.72920928466815	-1.09924110514612
C	-0.17732192174711	2.61543011986490	-1.59686959050129
C	-0.35593689838069	3.82921413940925	-0.93697990331801
C	0.33542576360101	4.06796794370486	0.32777873943278
C	1.38129433705500	3.12166245235356	0.71681946324575
H	-0.58700757621459	3.83871830370935	1.24168512539722
H	0.49850552396677	5.12207091649435	0.60386188705945
C	1.52571265430974	1.93374173223734	-0.00108225052407
H	0.69475740932440	0.73542840276182	-1.49020781198908
C	-0.90616313580196	2.15103026941279	-2.81750785947490
H	-0.76714007725766	1.06083886342801	-2.94734423006868
H	-0.53911788307790	2.68407876461275	-3.71802358026720
H	-1.97914664756663	2.39573840491461	-2.74107530401527
C	2.47840225212774	0.83454261653641	0.32104254288124
H	2.10627817711003	0.25600005427719	1.18559638664875
H	3.45945066421793	1.23352218905656	0.62560072223204
H	2.58919535613895	0.14364968191793	-0.53058977092693
C	2.21083701865547	3.38320854449942	1.91038892600210
O	3.02686396454952	2.62755694325431	2.42554715543633
O	1.95783442477005	4.63603897070217	2.41140038245324
C	2.74900326072782	5.03319081727430	3.54824572885560
C	-1.36571789108362	4.80104668227310	-1.41289074065145
O	-1.54385539233094	5.79866314760968	-0.49872228227299
C	-2.58562413263273	6.74234900985121	-0.81098553928692

O	-1.99594394672214	4.74654004072403	-2.45946849178298
C	4.09662517461596	5.59443303277059	3.12353324478689
H	2.88205929342677	4.15605370818090	4.21492061444494
H	2.13439796986806	5.79693981173031	4.06550076027628
H	4.65318081114536	5.96246182320456	4.00904427693953
H	3.96901654063612	6.43875948798459	2.41732763855033
H	4.70540750400403	4.81072064683631	2.63308028600517
C	-2.67300501575439	7.73381400602564	0.33060463434181
H	-3.53469543205341	6.18358778598707	-0.95436103888676
H	-2.35058907716648	7.23356933477586	-1.77908285305553
H	-3.45101652853948	8.49305587035277	0.11642280699527
H	-1.70855594003170	8.25907924419508	0.47987851611850
H	-2.94171239157885	7.22466543737154	1.27785809519608
C	3.39645084796248	-7.05744212563850	-1.57967064540085
C	0.83943488211808	-7.02191805081152	-0.40349942949591
C	1.37709779253524	-8.20764808469179	-0.88255704038961
C	2.67313923653415	-8.23361605611754	-1.46229612987967
H	-0.16844567418793	-7.01759725455258	0.03225930426174
H	0.78918591820229	-9.13580668833424	-0.81789152407562
H	3.09153057340937	-9.18198087116596	-1.83184912224710
H	4.39182193770394	-7.05506280317866	-2.05102062683711
C	1.05917379877417	-4.54577663015658	0.02288504566510
C	-2.08823782272255	-6.17273571368689	3.59476680357062
C	0.53954187892778	-6.02568247610891	2.59372377882750
C	0.25758511840949	-6.78963265592586	3.71727509103310
C	-1.06877338624595	-6.87657966571813	4.21717054045243
H	-3.11760473044620	-6.21542573728778	3.98403118774931
H	-1.28177224224920	-7.49158546961768	5.10454901735144
H	1.56932558288670	-5.96012091462346	2.21560518758794
H	1.07011771324812	-7.32892117854174	4.22732578475448
C	3.80908188996074	-2.11927110046288	-1.20428666792881
C	4.79381854967382	-1.66138751974585	-0.28583301930291
C	5.56937067900287	-0.53665491637294	-0.62463461881133
C	5.41719020921803	0.12563292458436	-1.85578175337269
C	4.43137994364003	-0.33905597787992	-2.74371909055766
C	3.62375046564478	-1.45267667304396	-2.44807090060805
H	4.32136780951368	0.16805045768097	-3.71316096105364
H	6.33856332434959	-0.17542520581774	0.07336969023837
C	5.01420281736532	-2.37149204744954	1.04788031171705
C	4.10603924171994	-1.78563535820777	2.14512608113283
H	4.34792805448498	-0.71804329256967	2.33190371602575
H	4.23118594068232	-2.33793294111810	3.09953907876235
H	3.04203099268020	-1.84560324914980	1.84798577917605
C	6.48254308916364	-2.38623162221046	1.49512314582877
H	4.70330028984766	-3.42697584787975	0.90112226808284
H	7.15016809275820	-2.76778439044971	0.69620590328221
H	6.60524772583819	-3.03459427357544	2.38624574906093
H	6.84144530732575	-1.37529082418107	1.77940283110668
C	2.63847875479800	-1.96895354263615	-3.49381713769547
C	2.01750471215493	-0.85658515151650	-4.35028011024841
H	1.18763500963942	-1.26890540403586	-4.95834395155602
H	2.75238738953957	-0.41457824925823	-5.05546753296602
H	1.59960870791281	-0.04929453875575	-3.72123318706811
C	3.31346978813026	-3.01706485285264	-4.39918476516786
H	1.80843097215570	-2.45898432096449	-2.94698616167151
H	2.59502350756662	-3.40365490436659	-5.15112881514994
H	3.69358777493234	-3.88013595785051	-3.82024729902229
H	4.17088743631547	-2.56821074623423	-4.94348163312510
C	6.34801594338123	1.22357467910107	-2.27161937059303

C	-3.64231865093110	-2.82347955660857	0.21056022543511
C	-4.18405250981583	-1.76584810155611	0.99394949784441
C	-4.07774337384437	-3.00409646641084	-1.12948464271385
C	-5.08837375726101	-0.87630073144376	0.39212423233324
C	-5.04986340776485	-2.13581894902452	-1.65569641395137
C	-5.55072362666891	-1.04079727288094	-0.93011590907311
H	-5.44898493081471	-0.01500077999918	0.97106987333779
H	-5.42954262407754	-2.32148515495298	-2.66941777796845
C	-3.86428183629399	-1.59495552358308	2.47708840440146
C	-3.41718347453313	-0.17592037669982	2.84234648463068
H	-3.16351211333241	-0.11375686410316	3.92163680743628
H	-4.20110460200943	0.58369391894357	2.64490575330316
H	-2.52228843821230	0.09718141984395	2.25851662021037
C	-5.06921620291824	-2.03484447898621	3.32946691573046
H	-3.01730032931983	-2.26850367082487	2.71391725605649
C	-3.55447819226883	-4.12809787602267	-2.01783763527113
C	-4.66615239685130	-5.13787036673245	-2.35156949546620
H	-5.47901271819249	-4.66696385084908	-2.94322709984108
H	-5.12079994365591	-5.55638654776261	-1.43103646655056
H	-4.26300940831297	-5.98001042624973	-2.95104487803373
C	-2.88594663607415	-3.57771734529114	-3.28981874126793
H	-2.77842056165524	-4.67914275179394	-1.44966311254872
H	-3.61796662057037	-3.05071436580014	-3.93698618645671
H	-2.44832634805398	-4.40376361339451	-3.88752513348295
H	-2.07468619612069	-2.87011379390131	-3.03198455737244
C	-6.52901533185826	-0.06703525456910	-1.50478161230870
C	5.95161634612317	2.58518202156753	-2.22697220496275
C	7.64179996714923	0.87898677525950	-2.75459771641228
C	-7.73289926834774	0.22447295811152	-0.79630805668782
C	-6.25475754098831	0.63654789238153	-2.72058528403291
C	6.85387320394223	3.57293870461945	-2.66544661303186
C	8.50992479586804	1.90292491099770	-3.17008591186291
C	8.13719241409625	3.25820335390505	-3.13710771555561
H	6.54943459044303	4.63214717385715	-2.63535072835627
H	9.51135185501269	1.63131529816705	-3.54059665825675
C	8.10886435609834	-0.57316860108033	-2.82291839437752
C	8.65086805950384	-0.94998277515180	-4.21079344510825
H	8.89670639254247	-2.03107641860957	-4.25137098175191
H	9.57806598259694	-0.39371814963690	-4.46172138871075
H	7.90621189385774	-0.73733613676470	-5.00389531103079
C	9.13651607958451	-0.87436129045871	-1.71807611282571
H	7.22477861070586	-1.21463283526368	-2.63427254688839
H	8.71831889852170	-0.66719697518595	-0.71279125418060
H	10.04706475425178	-0.25051136728625	-1.83798568108241
H	9.44852756897968	-1.93921387190117	-1.74596602808847
C	4.57558898819666	2.99899897917537	-1.71543263443168
C	3.66802072509342	3.49485559019972	-2.85387838520731
H	4.08693912977247	4.40188381839476	-3.33786713771693
H	2.65913149457204	3.75254656436302	-2.46824831327780
H	3.54701609276692	2.72137311842201	-3.63872332924931
C	4.67180200887326	4.03468886009500	-0.58351671760978
H	4.10614191741502	2.08872252752627	-1.29636216219104
H	5.16610699079157	4.96863711236188	-0.92187117620392
H	5.24920506549592	3.63826565502331	0.27559937017690
H	3.66166287417664	4.31139779113352	-0.22070392730211
C	9.08841084836123	4.34910940780269	-3.60942681272075
C	9.35781048113549	4.24276003513975	-5.12044197869647
H	9.86558962109621	3.28759560095404	-5.36926446336568
H	10.01010948160155	5.07038873041399	-5.46835056105375

H	8.41382063559799	4.27885596376272	-5.70001208546604
C	10.39977625212440	4.35330202889613	-2.80671413540937
H	8.57820454162880	5.32056469672947	-3.42719950820003
H	11.05427717412748	5.19303900616668	-3.11926273589331
H	10.96837766582347	3.41301021780336	-2.96252426529568
H	10.20664058595633	4.45092825997077	-1.71969253872713
C	-8.58521190546216	1.23894727171118	-1.26977995864852
C	-7.14925570987487	1.63252077593683	-3.15010522952318
C	-8.30937851406718	1.96946520199528	-2.43341552864226
H	-9.50767976009028	1.46880001125135	-0.71109421146915
H	-6.91878748521165	2.18026802484029	-4.07648163367783
C	-8.17645953883576	-0.55631399724491	0.44090978165875
C	-9.51586952493790	-1.27318223155960	0.19666643758615
H	-10.34259287953092	-0.55444528943824	0.01868381157720
H	-9.79384575202651	-1.88954470438847	1.07659459925126
H	-9.45693446690454	-1.94170888373755	-0.68539823245136
C	-8.24344785691287	0.33593327566910	1.69178173901049
H	-7.42118565890557	-1.34299842791955	0.63108336042013
H	-8.49401527062195	-0.26394128883209	2.59116673262365
H	-9.01993725339389	1.12237016076791	1.58777111585888
H	-7.27878602311753	0.84958796298483	1.87889985626641
C	-5.02807446305906	0.36011674797405	-3.59128306454091
C	-4.35216973080631	1.63799700801510	-4.11843547130107
H	-3.39197130958125	1.38413803306963	-4.61096697140143
H	-4.14241603372636	2.36982960367966	-3.31389012331327
H	-4.97566229755902	2.15089053302156	-4.87900453792897
C	-5.39879245081745	-0.54920864595032	-4.77966472622363
H	-4.28478279398452	-0.18136865062385	-2.96988382143804
H	-4.49874401799518	-0.80917628207046	-5.37414343162731
H	-6.11545895850895	-0.03282735179453	-5.45181518956328
H	-5.87968903086651	-1.49229087576708	-4.45400565102856
C	-9.21557216986358	3.10516364655968	-2.88445775343646
C	-9.71984099715124	2.91548045495310	-4.32383922971305
H	-8.88303118240700	2.94274583952361	-5.05225450963261
H	-10.42812748685515	3.72208984987899	-4.60390803782300
H	-10.23749809793926	1.94297997384370	-4.44448677870631
C	-8.51430840471274	4.46458818464025	-2.71139865528115
H	-10.10205824829194	3.09608046679825	-2.21258295051686
H	-8.19161195497541	4.61897491289544	-1.66179374824624
H	-9.18751610227375	5.30090552745283	-2.99245640024518
H	-7.60985108672718	4.52880136723508	-3.35154806243649
C	-0.62772176582933	0.12571150065634	6.75974383452538
C	1.61863555968282	-0.45930912996551	7.59758278930915
C	0.63105079128305	-0.25525027218950	8.76130785134692
H	2.43653438277098	0.29084180493729	7.64173931877007
H	2.09399861857776	-1.45848960664910	7.58420475998918
C	-0.42727618520571	0.68886540583470	8.17235258709333
H	-1.23271104943315	-0.80356238826114	6.81329026987150
H	-1.14439306890871	0.81738689886918	6.06452439107974
H	-1.36518033322190	0.73350288776607	8.76153426466609
H	-0.02727568587329	1.72626596576794	8.12167522226386
H	1.11348445515957	0.13358908809401	9.68009950762666
H	0.15244847931345	-1.22163772039343	9.02667847391727
H	-5.38689735345264	-3.07081917419663	3.09362244838832
H	-5.94271794756035	-1.37331669364158	3.14941097906731
H	-4.82647553874481	-1.98708531840401	4.41156674767672
H	0.20769530983909	2.82503110838996	4.39203260658371

1d2a4a

18 conformers were generated by GOAT, all of which were used for optimization and frequency calculation at the PBE-D3(BJ)/def2-SVP level of theory.

Electronic energy (a. u.) | ωB97M-V/def2-TZVPP: –4118.27199046396
Thermochemical Corrections (a. u.) | PBE-D3(BJ)/def2-SVP: 1.380282790774
Imaginary Frequencies: none
Gibbs Free Energy (a. u.) –4116.891707673190

```
182

  C   3.62935835700265     -2.13342821955336      0.55761637614849
  C   5.50515176420166     -1.26038835149184     -0.95335982745696
  C   6.15462334714891     -0.12962368838217     -1.57017874146238
  C   5.55138502708277      1.15751759529227     -1.48162554366722
  C   4.31122257420977      1.34688977308751     -0.88636829825721
  C   3.66772226065498      0.20811714160829     -0.30269209554616
  H   6.08778151837535      2.02311351969879     -1.89859185996300
  C   4.34272258567275     -2.80413647212495      1.61683198191879
  C   2.29517861538057     -2.48715116710318      0.31906585888510
  C   3.70807162883490     -3.90282582448432      2.30346494412281
  C   2.37878598820819     -4.27011884965594      1.95284907455987
  C   1.64855670230923     -3.56804815634630      1.00191465930095
  H   1.90716293029907     -5.12022432557925      2.47025215511145
  O   2.44281572857159      0.39808661157657      0.30027705080178
  O   1.58597697372946     -1.82975592642978     -0.65982894686547
  P   1.07699757787119     -0.24595356484925     -0.44482986787233
  O   -0.01921871245469     -0.13678416995308      0.60091904977007
  O   0.91250714562516      0.31069539237170     -1.85168128161809
  N   -1.74784063421845      2.28247843307421      2.56770919972529
  C   -2.31381742160777      1.33578511871914      1.82636886210184
  C   -0.28402869187957      2.37567731336117      2.67298123784536
  C   0.11063964414819      2.13183614290715      4.13373230377380
  H   0.02971353942320      3.39531884805946      2.36496283524939
  H   0.17211856429615      1.62613791601058      1.99519786756072
  O   -0.54090212645134      3.04326929675136      5.00640479612460
  H   1.19881303319130      2.28177018017919      4.25696094480262
  H   -0.13347045059961      1.07635849806520      4.40806858074166
  C   -1.94330830016779      2.92050408001039      4.91703861228913
  C   -2.44322185235464      3.18157291266206      3.48778072993952
  H   -3.53493952731057      3.03057765537829      3.42079677944440
  H   -2.21937978728929      4.22041047058533      3.17112694770861
  C   -3.69361419490252      1.01727801809232      1.78530381655214
  H   -1.58312508651497      0.74564060063351      1.23923402992721
  C   -4.19573410828904     -0.05767508758844      1.05992207457000
  C   -3.32295942387028     -0.91349095894164      0.20461605807359
  H   -3.54227017151657     -0.70417602809798     -0.86689833893948
  H   -3.52806191837798     -1.98834670435884      0.36405336647491
  H   -2.24044220864439     -0.75276527187080      0.34756556325645
  C   -5.63683060396123     -0.38962225344695      1.10891881185623
  C   -6.49945953208247      0.12393586124717      2.11064717699425
  C   -6.20127920172985     -1.26029999165467      0.14127463201932
  H   -4.40174881993256      1.64809619103535      2.33953844447478
  C   -7.56268328244871     -1.58348405042284      0.16376445509376
  C   -7.85923996986763     -0.20107677309372      2.13391661813510
  C   -8.40088505839391     -1.05443029415802      1.15762981003458
```

H	-5.57008659197294	-1.68007226528760	-0.65314333238781
H	-7.97162859294939	-2.25652410173335	-0.60484333830035
H	-9.47037525757680	-1.31254110323976	1.17856679701764
H	-6.09540042575695	0.76824449483464	2.90456328873460
H	-8.50266744642609	0.20613109457241	2.92841479356347
H	-2.27451891297512	1.89994138363531	5.23554502129647
H	-2.38583575192781	3.66325902395714	5.61051988000486
N	-1.58124170922406	1.38056851699483	-2.21434439897033
C	-2.57192226043336	0.65982842121098	-2.85248917969934
C	-3.89587562501607	0.91141454244001	-2.53965741514117
C	-4.27292153895448	1.97093523256996	-1.52620951420613
C	-3.09720001196467	2.72114291334301	-0.94729208240802
H	-4.86137319628900	1.50297623003436	-0.69843108901945
H	-5.00585297130029	2.68079398250766	-1.96742413219466
C	-1.80064993860772	2.39044285284540	-1.30753248579798
H	-0.59505290320997	1.00706388265829	-2.24743089381651
C	-2.08706817202336	-0.39183492374001	-3.83380497073668
C	-0.56263579907417	3.09422452283961	-0.84436041733665
H	-0.44761327846526	4.04652820377753	-1.40095680411414
H	0.33786023345654	2.48621596710811	-1.03469335154705
H	-0.62906470176062	3.37673846225716	0.21585793909246
C	-3.36919263020180	3.79873259163010	0.00482226261868
O	-2.57064334305175	4.51651944088035	0.61090384778452
O	-4.72502848132771	3.93213343594870	0.20118047146921
C	-5.11780107433158	4.97846865852705	1.08283304567914
C	-5.00019510167969	0.13023745001583	-3.12449011290883
O	-6.20933247810535	0.65888609510653	-2.74622195894220
C	-7.35440815529935	-0.04627774451642	-3.21812330773596
O	-4.94000740298123	-0.88310173176226	-3.81660324132762
H	-4.81927129557804	4.76019633989646	2.13068253984512
H	-6.21914167343330	5.04226480415071	1.01593937954881
H	-7.29109027558218	-1.12423417727458	-2.96525980510409
H	-7.44728683836195	0.03700607223326	-4.32083083390752
C	7.38915729622576	-0.32481715121350	-2.25671052820545
C	6.10583255292582	-2.54632771473063	-1.09874392892087
C	7.30009626781180	-2.70516432381980	-1.78699972223986
C	7.95671788500665	-1.58529599388803	-2.36204057494703
H	5.60140984379885	-3.42049078148063	-0.66430748679339
H	7.73847487427815	-3.70915546963323	-1.89246228641839
H	8.90663501320067	-1.72212607941016	-2.90058703020077
H	7.87800571902496	0.54954194386658	-2.71442344149531
C	4.26798529543467	-1.05490058198035	-0.24363488626558
C	4.41625085997191	-4.58292482046933	3.33790899999710
C	5.64381188500590	-2.40468617922990	2.04689228262011
C	6.30006831814830	-3.07713058673660	3.06809422518717
C	5.69029640625060	-4.18554775087078	3.71249961361070
H	3.92118829312504	-5.42636417170758	3.84451078216562
H	6.22330677423796	-4.71621302470151	4.51588039827432
H	6.12376856302002	-1.54290916910418	1.56357149183482
H	7.30049983740967	-2.74393401182563	3.38352912526995
C	3.67680965004967	2.69451867986692	-0.74097150930184
C	3.71505011393068	3.32870392188237	0.53036136271286
C	3.05106644538623	4.55518235998475	0.70202573065927
C	2.37577542231627	5.19031519103062	-0.35465188033073
C	2.39529281023355	4.56695925182786	-1.61290183838067
C	3.01762809326382	3.32275280212108	-1.83067953024311
H	1.88093981325697	5.06212105880245	-2.45148392851182
H	3.07517828421749	5.03717669405566	1.69306947208000
C	4.48069972982249	2.72422023279345	1.70453950320135

C	3.54065940748082	2.30085514819933	2.84369644241943
H	3.00603674773490	3.18052177646910	3.26128843841781
H	4.11035021570676	1.83159079530922	3.67239680836100
H	2.79360760273296	1.56932433970805	2.47834202406941
C	5.58274203909301	3.67332745828326	2.20479065851504
H	4.98411005694243	1.80726545290272	1.33851681699075
H	6.27248598993353	3.95612612614975	1.38445627736155
H	6.17930785629189	3.19089873200923	3.00631279931807
H	5.15788324821825	4.60954067688725	2.62336404336086
C	2.99988393312083	2.68513410874988	-3.21722105489134
C	1.67622574190969	2.87889533939274	-3.96950297953911
H	1.68675730204623	2.29449462889255	-4.91157983342683
H	1.50386248619315	3.93949911797027	-4.24784363647350
H	0.81597263587732	2.52953773107039	-3.37110413868006
C	4.17131806673127	3.20832182060883	-4.06991199196804
H	3.13342516036168	1.59391419337132	-3.06946634738128
H	4.07869896108817	4.30313370325020	-4.23035130750366
H	4.18335549618039	2.71725684602987	-5.06491443307681
H	5.15057373080370	3.02442930260251	-3.58778443002827
C	1.66648222694294	6.52229887110742	-0.14729080513949
C	0.23160509532609	-3.91707093424031	0.68447025036226
C	-0.79310145561377	-3.69278267703816	1.64199396727554
C	-0.08737404230330	-4.48476432854112	-0.57989121821952
C	-2.11778810010928	-4.02406030252846	1.30072147613480
C	-1.42961022151432	-4.77692973588632	-0.87853251490705
C	-2.46504025170607	-4.54969573291446	0.04556402703944
H	-2.91802240567029	-3.85718098571637	2.04130965075450
H	-1.66933563690533	-5.21571502701314	-1.86010106400689
C	-0.51064393798609	-3.11483945627332	3.02742442536626
C	-1.24829473755400	-1.78611809230580	3.25777089179885
H	-1.02498507066468	-1.38949906671377	4.27114340052194
H	-2.35001754620951	-1.90220101507992	3.18228343197860
H	-0.91764763738947	-1.04681189837617	2.50400407657776
C	-0.82130339997049	-4.13982004531554	4.13128501900059
H	0.57239069128397	-2.88850039498739	3.07893357447604
H	-0.55896358423750	-3.73448760153699	5.13049314811147
H	-0.25272259732049	-5.08013625724848	3.98242335346750
H	-1.89942191461889	-4.40377393061476	4.14849520179512
C	1.00534866708391	-4.86886562460862	-1.57451545946725
C	1.08423607922652	-6.40012299950745	-1.71343514894406
H	0.15154131040309	-6.81559266659867	-2.14900118770143
H	1.23893316906693	-6.88496519773371	-0.72837476067155
H	1.92419292532011	-6.69179004636637	-2.37726336645444
C	0.85399201140107	-4.18098771523917	-2.93854394543542
H	1.97280963732262	-4.53505182982506	-1.14944494129410
H	1.68231070825560	-4.47929032599697	-3.61365139669807
H	0.88059276535575	-3.08095608097062	-2.82656837286445
H	-0.09747465775097	-4.46134625384175	-3.43678406064168
C	-3.91197625399140	-4.92128955914760	-0.25726002388084
C	2.67149020549686	7.64137826215051	0.17738988729746
H	2.16043264945333	8.62291187248076	0.26059292496206
H	3.45176261916533	7.72224863292872	-0.60564717927325
H	3.18569877853771	7.44882484428766	1.14225719328103
C	0.56372866581342	6.43703501860428	0.92304576855564
H	1.17800804764786	6.78003704149626	-1.11285728475924
H	0.98532576026413	6.14098854794394	1.90697135284859
H	-0.22641160424491	5.70914027593823	0.65299700687779
H	0.07523823885908	7.42399791246738	1.06033151000993
C	-4.39996835581795	-4.42639408089133	-1.62683382617836

H	-5.47129636336383	-4.67416430983533	-1.77290148338058
H	-3.83853333066149	-4.89901820587058	-2.45849608408360
H	-4.28619965264088	-3.33026256368436	-1.74167288062585
C	-4.11507570151622	-6.44115290325240	-0.11581018823909
H	-4.53785877084989	-4.42800952158107	0.52049585031723
H	-3.80848204838657	-6.79484474236116	0.88870030646105
H	-3.50363221366127	-6.98805245152553	-0.86345135258322
H	-5.17732103732316	-6.72125333325277	-0.27418870862790
H	-8.22732158918834	0.41626760738197	-2.72318848364267
H	-4.65305563624843	5.94352084765194	0.79613432242371
C	-0.85271560121408	0.04702386180657	-4.63686447349165
H	-0.65793445822287	-0.69980690541942	-5.43229855132930
H	0.05728493409602	0.10190454141085	-4.00752855441167
H	-1.00700419024530	1.02977960953383	-5.12652951256613
C	-1.84044249110691	-1.72369196456128	-3.10117510993452
H	-2.92784857102435	-0.56673744173212	-4.52949297673008
H	-2.76864835970688	-2.09176097522472	-2.62777554289072
H	-1.05564209743089	-1.62955438066616	-2.32579680864207
H	-1.50754018472772	-2.49401667978155	-3.82375348515256

1d2b6e

58 conformers were generated by GOAT, all of which were used for optimization and frequency calculation at the PBE-D3(BJ)/def2-SVP level of theory.

Electronic energy (a. u.) | ωB97M-V/def2-TZVPP: –5208.11047695836
Thermochemical Corrections (a. u.) | PBE-D3(BJ)/def2-SVP: 1.921211795535
Imaginary Frequencies: none
Gibbs Free Energy (a. u.) –5206.189265162820

248

C	0.29066841687917	-4.97917973257130	0.34454334559928
C	2.24621991497280	-5.93144703545335	-0.99245329036550
C	3.58141136982728	-5.76687063763398	-1.51247206372363
C	4.22865532409499	-4.50663519233551	-1.38481471947124
C	3.59910945542827	-3.40806626317804	-0.81177066286132
C	2.29833068464425	-3.60190829143589	-0.24963283712834
H	5.25050853043394	-4.39152307631644	-1.77727670937504
C	0.00066758701430	-6.02453821069646	1.29292840769056
C	-0.73972798641153	-4.09751239985943	-0.00978957606752
C	-1.36299457517030	-6.22813426107086	1.71817295106490
C	-2.38414185754670	-5.36786116712318	1.22506541770537
C	-2.09839114639061	-4.28650655051493	0.39935748699766
H	-3.42645593869528	-5.55175964619505	1.52902675550255
O	1.71353520940388	-2.54040919700760	0.40310716350760
O	-0.47053249259591	-3.04813702940392	-0.85399768381837
P	0.41916802308127	-1.73709370293898	-0.30740345748692
O	-0.26591179828610	-1.03746164153741	0.85678323270002
O	0.84012142646416	-1.00759930110689	-1.57130243172547
N	-0.11166455984912	1.55472024947426	3.29781898359733
C	-0.83620069061453	1.53128996864189	2.19105950482351
C	0.76793246835313	0.43460420557851	3.65702474010961
C	0.29034740083143	-0.14053672597190	4.99589221495983
H	1.80041650690681	0.83117206755636	3.73158426539298
H	0.71077663355938	-0.33226070040124	2.85877839986948

O	0.18082970972523	0.85386244103967	6.00357965727935
H	1.02847466505798	-0.88300073543176	5.35767129743231
H	-0.68069213529794	-0.66663910306156	4.84353884357357
C	-0.68870638752324	1.93042049066976	5.65903910196732
C	-0.17679385282506	2.56685628784214	4.34600783459954
H	-0.79736283929218	3.41837694147687	4.01936412671831
H	0.85884149431914	2.92831318546093	4.50367331279019
C	-1.88813244662065	2.43055550706708	1.86376675725672
H	-0.62150575010579	0.66347866942792	1.53377383099116
C	-2.74932385879434	2.21016398807391	0.80121299662198
C	-2.61314576154364	1.07355258321180	-0.15793459887040
H	-2.63883236550374	1.46655894636398	-1.19308161752825
H	-3.47620557913996	0.37884462336988	-0.07203511061761
H	-1.70528647006312	0.46489252916662	-0.01324458857766
C	-3.89725179151424	3.11911089781906	0.60892697589035
C	-5.11580606765332	2.62466132420072	0.08280822222143
C	-3.81585142188443	4.49473491644864	0.93917654092624
H	-2.10476869812874	3.27215752230604	2.53696329301792
C	-4.91520007064740	5.33999467503207	0.75241940107846
C	-6.21820838900618	3.46958892155029	-0.08815825331612
C	-6.12228196696647	4.83006466419751	0.24287471119352
H	-2.86117272530309	4.91361243511652	1.28949230697529
H	-4.82593760844676	6.41017366019205	0.99309233661522
H	-6.98592811429025	5.49640313208458	0.09532966273098
H	-5.21828052106461	1.56310131108180	-0.18116425462560
H	-7.15044106678668	3.05449618630779	-0.49599782701813
N	1.00696801296095	1.67538436667570	-1.18297462515930
C	0.01527482554699	2.39570148484478	-1.82861876358476
C	-0.36763536981568	3.62563683589940	-1.33902936631903
C	0.34354316948695	4.23457292226891	-0.14214754148344
C	1.24583119773106	3.25528470471947	0.58582253548219
H	-0.40674108914016	4.64531782478964	0.56528754386822
H	0.92160703723204	5.13687303663413	-0.45476352207965
C	1.57253823622553	2.03649116612675	0.01681398391234
H	1.07629074712782	0.65530795846440	-1.43998612806550
C	-0.51491707225278	1.69258540326504	-3.04357662066615
H	-1.37015139766984	2.23353426028442	-3.47555380682859
H	-0.78508077817896	0.64743820339858	-2.78685765530219
H	0.29501209589588	1.61875950870582	-3.80037745377615
C	2.50994973345995	1.01255676855514	0.58163450803708
H	3.30152821643028	1.47412830027560	1.19242395938152
H	2.94882798119561	0.40200428269352	-0.22820362772352
H	1.95848055334670	0.31100749809540	1.23509185164020
C	1.81815816831200	3.66797603732948	1.86423638191525
O	2.49557540980796	3.00261001885498	2.65386641502554
O	1.47669096177247	4.97296654297840	2.14401300984220
C	2.05453959822162	5.55610726011891	3.32241365693039
C	-1.45779519990226	4.40201585039688	-1.94958927122499
O	-1.58399164278643	5.62006243251528	-1.32323849485517
C	-2.62901384781003	6.47635030863933	-1.81669300566807
O	-2.21003033023042	4.07931268618005	-2.86791680073143
C	3.46055296061538	6.07466109969444	3.06217905361266
H	2.06493978918463	4.80476592940998	4.13987855628094
H	1.36446691606253	6.37874253073305	3.59815901202724
H	3.84789768615035	6.60009720705674	3.95852579964814
H	3.46761261264053	6.78430122071542	2.21110281709910
H	4.14521265141373	5.23686039284118	2.82890648054540
C	-2.19789472152869	7.23966734972775	-3.05966326427878
H	-2.84690177692989	7.16415568600205	-0.97513282900102

H	-3.53043661003303	5.86294532964475	-2.02098475432232
H	-2.99036616606548	7.95376026705432	-3.36308281403175
H	-2.02289851156467	6.53987891954852	-3.89916706968907
H	-1.26815504581994	7.81286520371941	-2.87072765906620
C	4.21327078277498	-6.85747620443751	-2.18003147765428
C	1.58198669831784	-7.17195380803697	-1.23308610263766
C	2.21728935509460	-8.20811936417422	-1.90244460415424
C	3.55040408464200	-8.06001736204144	-2.36775712040743
H	0.54694776542366	-7.29990407512260	-0.88891952782544
H	1.67841195687067	-9.15130679872920	-2.07956062391505
H	4.04619957741266	-8.89156467489459	-2.89095493856206
H	5.23722008116551	-6.71576600551486	-2.55997627759660
C	1.63109132907374	-4.83410917204313	-0.28631704289171
C	-1.65741752062477	-7.27862985320058	2.63716947668975
C	1.01385002091547	-6.85668633752473	1.85619784454544
C	0.69671425758402	-7.85875326978116	2.76185624953867
C	-0.65125509636352	-8.08375864988331	3.14795637548124
H	-2.70472529652704	-7.42877041374713	2.94322524424143
H	-0.89206686288952	-8.88707420555217	3.86053730403016
H	2.06063449455655	-6.68655486838294	1.56883241017073
H	1.49816631368326	-8.48168919368119	3.18718888501991
C	4.29216854432072	-2.07961217809084	-0.79226200165743
C	4.95551131206891	-1.63221003999774	0.38051663206166
C	5.72877687464626	-0.45774511564108	0.32168870363443
C	5.89049762516639	0.26552482744524	-0.87406667136311
C	5.17352093883212	-0.16279715459963	-2.00570826298721
C	4.35898831702627	-1.30901602811708	-1.98696708936374
H	5.27906059036936	0.40896043129923	-2.93950305569738
H	6.25798309684368	-0.11032868645797	1.22234852356055
C	4.87031372959175	-2.40821599577998	1.69159167700661
C	4.01658456153158	-1.65750870382655	2.72960129397985
H	3.96227234838808	-2.22928559698062	3.67947066648578
H	2.98698876046711	-1.51941702652390	2.34841240821975
H	4.44429180174827	-0.65854132221655	2.95717757233215
C	6.26097020160467	-2.75857094847332	2.24577990102960
H	4.35432615360963	-3.36540439935389	1.47318814119402
H	6.82954837985722	-1.85111332683790	2.53650387386089
H	6.86670276319843	-3.30677583177766	1.49611499942694
H	6.17180846958287	-3.39646933406180	3.14900525283198
C	3.59219903845207	-1.70490689042218	-3.24646612261363
C	2.95723851332443	-0.49359127396454	-3.94869881754139
H	2.25077233839637	-0.83498829712528	-4.73204854386361
H	3.71635567782253	0.14511366970846	-4.44670254993003
H	2.39207364674895	0.13015175382505	-3.23271643407776
C	4.48222004247378	-2.48549880669270	-4.22973606259785
H	2.76098432552475	-2.36312675137213	-2.92267925751832
H	3.91103731183244	-2.76110731727584	-5.14020272280306
H	4.87344479328422	-3.41879519876960	-3.78144526315118
H	5.35120703151694	-1.87060279769906	-4.54547773969940
C	6.86153655337473	1.39954912157250	-0.97761609389564
C	-3.16961579353905	-3.35157874003802	-0.06409819385312
C	-3.83707505320096	-2.50950673269978	0.86700439209416
C	-3.55075919127446	-3.32312236180255	-1.43388827789080
C	-4.84902076781859	-1.64797933399037	0.40513932220663
C	-4.60785390247483	-2.48776854569413	-1.83350489217147
C	-5.27294564724711	-1.62969184526361	-0.93947332114215
H	-5.34613013775155	-0.97639866264612	1.12228429623476
H	-4.93957073296730	-2.52159740189171	-2.87996857935950
C	-3.51138292749503	-2.51788408279333	2.35734385162790

C	-3.02715437497649	-1.14649859046672	2.84685367743470
H	-3.79806884195804	-0.35713910354813	2.72109225227484
H	-2.12072565792244	-0.85603719390526	2.28347607517607
H	-2.77800548859363	-1.19336179805067	3.92755939090060
C	-4.70327051157132	-3.03145072038677	3.18306776200694
H	-2.66963125922224	-3.22101096446026	2.51011843646257
H	-4.44066168780554	-3.08977470798042	4.25965331339415
H	-5.01985067766900	-4.04180672418698	2.85304112055607
H	-5.58294611733615	-2.36121396244978	3.08719978702274
C	-2.87959950720775	-4.19545679678400	-2.48937872472572
C	-3.87250486404129	-5.19403259349729	-3.10878677724998
H	-4.35151589812913	-5.82336121293073	-2.33171334515482
H	-3.35304822655211	-5.86483378202884	-3.82343862020518
H	-4.67972337323409	-4.67605233231760	-3.66762348765029
C	-2.18741772640349	-3.33877981114475	-3.56504312189535
H	-2.09425594456971	-4.79220739053010	-1.98494331075772
H	-2.92084755416777	-2.71865356213740	-4.12258582342646
H	-1.67054950545211	-3.98753708004128	-4.30185305519197
H	-1.43311897411535	-2.66930178338696	-3.10895492279246
C	-6.40989138706474	-0.75757809498112	-1.37084880805595
C	6.42083367395307	2.73837409368960	-1.17190122578152
C	8.25579193798409	1.11873303258137	-0.92702473376271
C	-7.67591456907555	-0.88206765394055	-0.72458191578047
C	-6.25574503123993	0.20448123417419	-2.41829589944935
C	7.37826246985838	3.75826430118402	-1.31171608205813
C	9.17168931593770	2.17927792492294	-1.05749259813142
C	8.75911946080715	3.50556065221310	-1.25411127161071
H	7.02929289068797	4.79198869229971	-1.46563089121735
H	10.25248066527106	1.96469515456792	-1.01734691792278
C	8.79372805088292	-0.30200560358388	-0.76700464243837
C	9.70743017254967	-0.70154009225241	-1.93769108625353
H	10.62520629830652	-0.07878655089223	-1.97748801488626
H	9.18429835103217	-0.59057041090320	-2.90864798673330
H	10.02830026406827	-1.75922829867393	-1.83912504636251
C	9.50034545275124	-0.48889777549181	0.58618376161049
H	7.92670725836919	-0.99130620326126	-0.78333448465563
H	8.82336198673605	-0.24835353454458	1.43055138078697
H	10.38880018017182	0.17085232085764	0.67345175834295
H	9.84425984978443	-1.53658637553037	0.71165214489897
C	4.94120288464101	3.10855129503416	-1.22347393882300
C	4.50420859412210	3.52010024718277	-2.64038957840069
H	5.03528358587246	4.43504045962257	-2.97674231418748
H	3.41433363803224	3.72824902735257	-2.66721298574317
H	4.71603847349598	2.72056130718438	-3.37818549753065
C	4.59349237935387	4.20120610733883	-0.19946180891200
H	4.36125225397478	2.20398719341788	-0.95755919286034
H	3.51079581086179	4.43472238183344	-0.23185974015882
H	5.14408289876047	5.14332224777384	-0.40073212729170
H	4.84123515868343	3.87827534549824	0.83136275672120
C	9.77444832312237	4.62951385614930	-1.40595053856022
C	9.62909089687478	5.68221928944366	-0.29410903039535
H	8.64380572402201	6.19015918886934	-0.35027076323686
H	10.41248436486460	6.46330658848752	-0.38133317576140
H	9.71043191891704	5.22049300469926	0.71016285342399
C	9.69781383945603	5.27494598205154	-2.79999057191505
H	10.78140309842410	4.16837861769861	-1.30340681053782
H	9.83519809166236	4.52084133391207	-3.60046278140853
H	10.47755908074220	6.05524164787958	-2.92107661084151
H	8.71219250543159	5.75887901213274	-2.96229264259000

C	-8.73117865900704	-0.03310179006322	-1.10799500046193
C	-7.34677568154852	1.02377213496711	-2.75916157160866
C	-8.59440186316306	0.92802278228112	-2.11954700031652
H	-9.70984892480285	-0.13807362402561	-0.61140540789723
H	-7.21452535087944	1.76364776949239	-3.56366466426798
C	-7.96651036638419	-1.94931919320998	0.33074566528984
C	-9.10714746492184	-2.88285718619545	-0.11062295626526
H	-9.24986293963487	-3.69842247635287	0.62805218400617
H	-8.88824748812478	-3.34480149045810	-1.09395271827126
H	-10.07291493798059	-2.34355718015041	-0.20005755733837
C	-8.25795952136558	-1.32765747216215	1.70690689779160
H	-7.06089676069973	-2.57722557816222	0.43436346722251
H	-9.17501601565695	-0.70265083120542	1.68329060877811
H	-7.42577861936440	-0.67723926947331	2.04496918153426
H	-8.40894758860309	-2.11614369136841	2.47292557834850
C	-4.96390574030181	0.35628667433393	-3.22166737973284
C	-4.53482735301989	1.81580572415660	-3.43326928980961
H	-5.25649288487465	2.37455572617897	-4.06395576039829
H	-3.55957218619980	1.85716640240382	-3.95470289914455
H	-4.42122850703719	2.37520820291187	-2.48593362648801
C	-5.09440553989177	-0.34316172658535	-4.58980947999976
H	-4.15451827108609	-0.15127863888449	-2.65721635750149
H	-4.13124908136611	-0.31030295414509	-5.13934513091077
H	-5.86006264178381	0.16341127644130	-5.21362869318391
H	-5.40013674063916	-1.40368518013493	-4.49529835280910
C	-9.76229210161027	1.82138241738114	-2.51591900304667
C	-10.10122694492940	1.69236132177899	-4.01003040905723
H	-9.27138167393940	2.06401653355299	-4.64615666511524
H	-11.00419051345191	2.28493465179444	-4.26365013481500
H	-10.29076544750261	0.63685876109596	-4.28931960092742
C	-9.51408457711896	3.28891801875139	-2.12623753140913
H	-10.64498615096873	1.46728196911308	-1.93938323165350
H	-8.61859584963616	3.69347467886394	-2.64266237842053
H	-9.35193289263140	3.39299776876109	-1.03417267159102
H	-10.37900629173141	3.92644152820471	-2.40303987174499
C	-2.18384462543234	1.54559826398694	5.56557550803429
C	-0.68045819825484	2.95925188445390	6.79774423650726
C	-1.96515128557879	3.78666984448176	6.60019001443501
H	0.24841306439707	3.56358951125569	6.81518494516013
H	-0.71081397309883	2.37758801307775	7.74169199040049
C	-2.94389624301644	2.86601571390228	5.80802669353763
H	-2.37864320015073	0.81441216788334	6.37731062007442
H	-2.46063737351894	1.05772476274773	4.61089381477696
H	-3.89148856638765	2.69030563065314	6.35335794411057
H	-3.23232941773809	3.33652555237909	4.84525093735226
H	-1.75226078614126	4.71339640992410	6.02862848073910
H	-2.38837888262851	4.11603804046336	7.56890041385430

9. Comparison of catalysts 4a, 4e, 6a and 6e

Relative Rate Experiments by NMR Spectroscopy

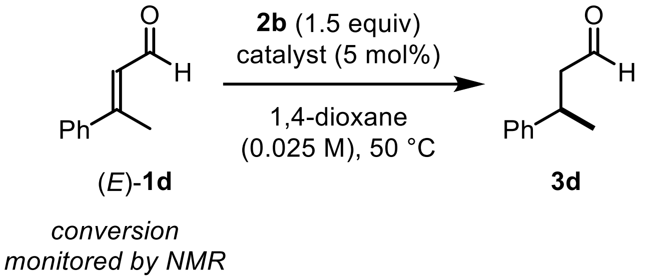

A flame-dried NMR tube under argon was filled with 5 mol% of the respective catalyst salt **4a**, **4e**, **6a** or **6e**, 1.5 equiv. Hantzsch ester **2b** (5.5 mg, 21.7 µmol), 1.0 equiv. mesitylene (2 µL, 14.4 µmol) as internal standard and 0.58 mL 1,4-dioxane-h_8. The mixture was sonicated for approximately 20 min until complete dissolution. Subsequently, 1.0 equiv. (*E*)-enal **1d** (2 µL, 14.4 µmol) was added, and the tube was inverted one time to ensure thorough mixing. The reaction was monitored by single scan ^{1}H NMR spectroscopy at 50 °C. The data was then imported into MNOVA 15.0.0 with the Reaction Monitoring plugin and processed therein (phasing, multi point baseline correction, integration and referencing).

Note: The reaction concentration was adjusted from 0.1 M to 0.025 M to ensure complete dissolution of all reagents yielding a homogeneous solution. Furthermore, 1,4-dioxane-h_8 was used as a solvent instead of the d_8 variant, as deuteration at the acidic α-position had been observed (likely due to the presence of D_2O). The solvent signal was suppressed using a WET pulse sequence (Bruker pulse sequence: wet) to enhance the signal-to-noise ratio of the reactants for the single scan measurements. (See the following review as a reference[52]).

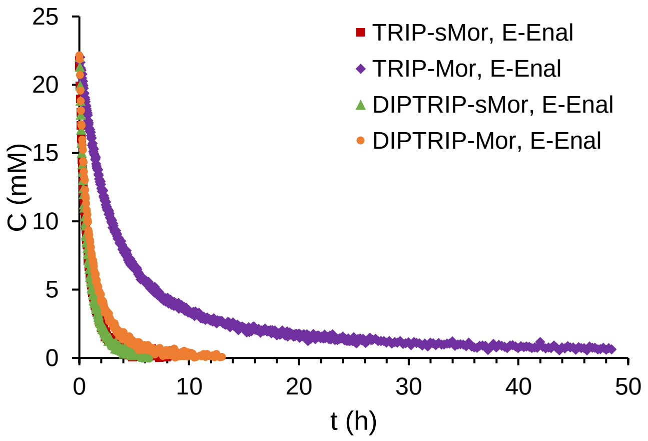

In order to compare the reactions without investigating reaction orders of this multiple component reaction in detail, we determined the relative rates of the reaction by using a variable-time-normalization approach.[53] Therefore, the time was multiplied with a relative rate ($k_{rel} = k_x/k_{ref}$), whereas the slowest reaction was used as reference reaction (**4a**). The value was obtained by visually searching the best graphical overlap to the reference reaction. This data was then converted to the respective relative energies using the following equation:

$$k = A \times e^{-\frac{\Delta E_A}{RT}}$$ Arrhenius Equation (1)

$$k_{rel} = \frac{k_x}{k_{ref}} = \frac{A_x \times e^{-\frac{E_{A,x}}{RT}}}{A_{ref} \times e^{-\frac{E_{A,ref}}{RT}}} \qquad (2)$$

Assuming that A_x and A_{ref} are similar we obtain:

$$k_{rel} = e^{-\frac{(E_{A,x} - E_{A,ref})}{RT}} = e^{-\frac{\Delta E_A}{RT}} \qquad (3)$$

$$\Delta E_A = -\ln(k_{rel}) \times R \times T \qquad (4)$$

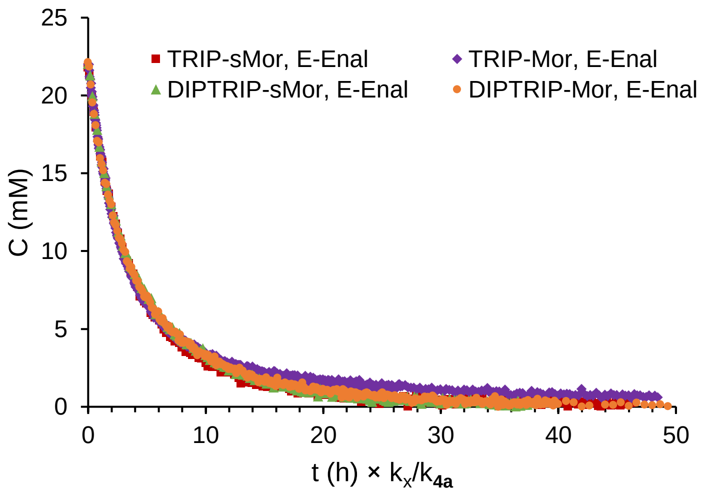

CPA	Amine	Relative Rate k_x/k_{4a}	Relative Energy ΔEA (kcal/mol)
DIPTRIP **6**	Mor **a**	4.0	−0.89
DIPTRIP **6**	sMor **e**	6.0	−1.15
TRIP **4**	sMor **e**	5.6	−1.11
TRIP **4**	Mor **a**	1.0	0

We performed a second kinetic analysis using the initial rates method, where the first 13–15% of conversion was approximated to follow zero-order kinetics, and the slope of the curve was taken as the rate constant. To achieve this, we monitored both the consumption of the starting material (enal) and the formation of the product over time, determining the slope for each reaction (**4a**, **4e**, **6a**, **6e**). After averaging the values and calculating the standard deviation, we extracted the relative rates with respect to catalyst **4a**. As previously described, relative energies were then obtained using the Arrhenius equation.

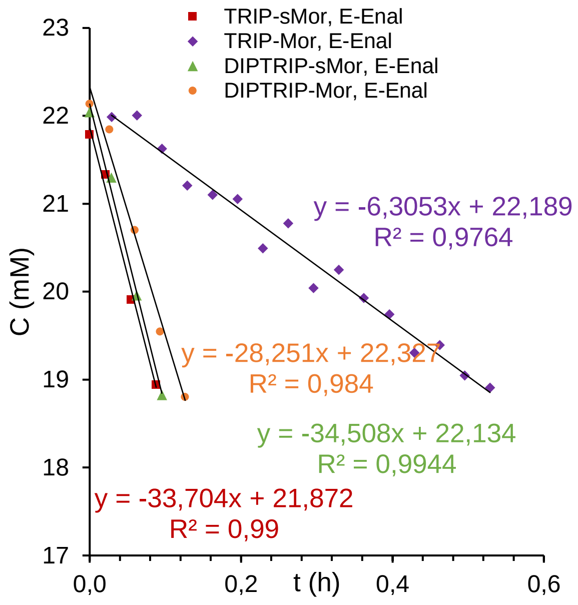

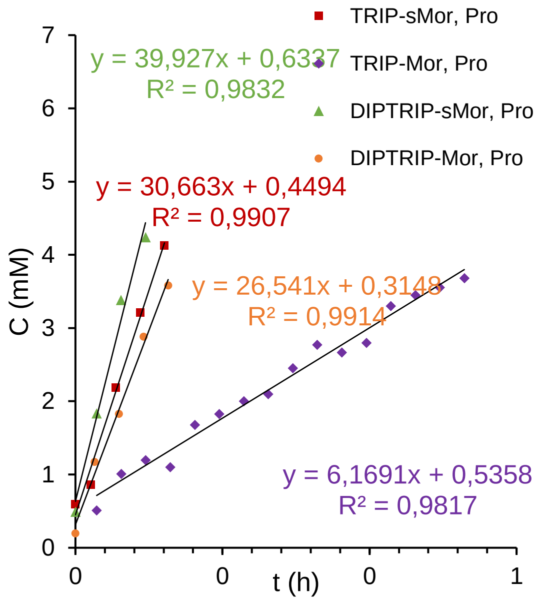

CPA	Amine	Enal Conv. (mM/h)	Product Formation (mM/h)	Average Rate (mM/h)	Relative Rate k_x/k_{4a} (initial rate)	Relative Energy ΔEA (kcal/mol)
DIPTRIP **6**	Mor **a**	28.3	26.5	27.4	4.5 ± 0.2	−0.96 ± 0.04
DIPTRIP **6**	sMor **e**	35.5	40.0	37.7	5.6 ± 0.4	−1.11 ± 0.08
TRIP **4**	sMor **e**	33.7	30.7	32.2	5.4 ± 0.3	−1.08 ± 0.06
TRIP **4**	Mor **a**	6.3	6.2	6.2	1.0	0.0

The data obtained from the two methods (VTNA and initial rates) show that the relative rates follow the same trend and remain within the same order of magnitude. The results indicate that the choice of amine source has the most pronounced effect on the reaction rate. Specifically, switching from morpholine **a** to **e** increases the rate by a factor of ~5.4–5.6 when TRIP **4** is used as the catalyst, corresponding to a reduction in activation energy of approximately 1.1 kcal/mol at 323 K. In contrast, variations in the phosphoric acid catalyst with amine **a** lead to a relative rate increase of ~4.0–4.5, translating to a reduction in the activation barrier of about 0.89–0.96 kcal/mol. When comparing the two reactions with amine **e** only subtle differences between the phosphoric acids are observed. As the changes are not additive, this comparison also suggests that the two components influence different steps to varying extents and may even result in a change of the rate-determining step in the reaction.

With regard to these experimental observations, we have calculated the activation energies of both transfer hydrogenations with catalyst **4a** and **6e** (see Section 8f, 8k). For catalyst **4a**, a barrier of 10.1 kcal/mol (with dispersion correction) and 12.1 kcal/mol (without dispersion correction) were obtained. Accordingly, this reaction experiences a transition state stabilization by dispersion interactions of 2.0 kcal/mol.

In contrast, barriers of 11.0 kcal/mol (with dispersion correction) and 6.5 kcal/mol (without dispersion correction) were obtained for catalyst **6e**. We believe this to be the result of dominating stabilizing dispersion interactions within the pre-transition state complex, which might experimentally manifest as a stronger kinetic preference for iminium ion formation. The kinetic data clearly indicates a significant influence of morpholine **e**, and could be a consequence of a stronger contribution of a different elementary step to the overall reaction rate.

10. NMR Spectra

^{1}H NMR spectrum of 1a

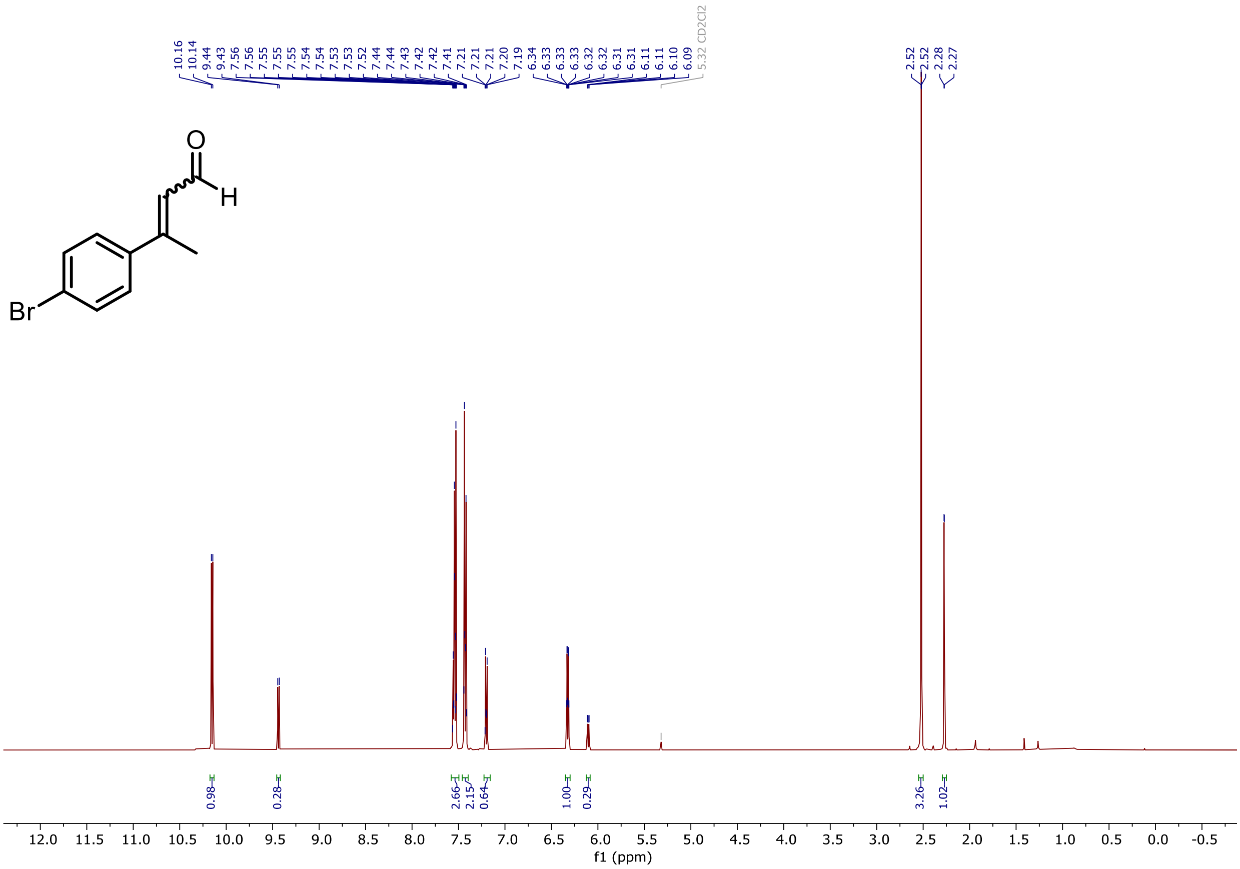

^{13}C NMR spectrum of 1a

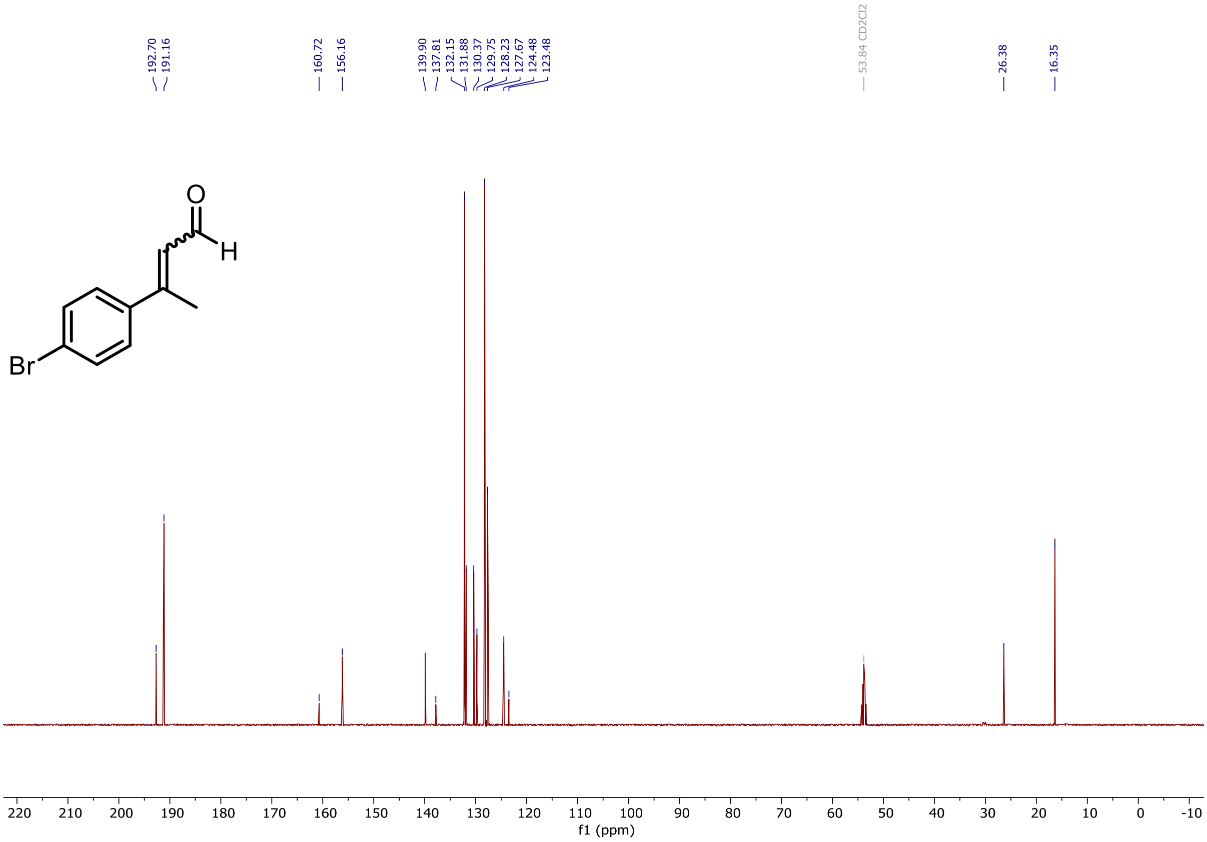

^{1}H NMR spectrum of 1b

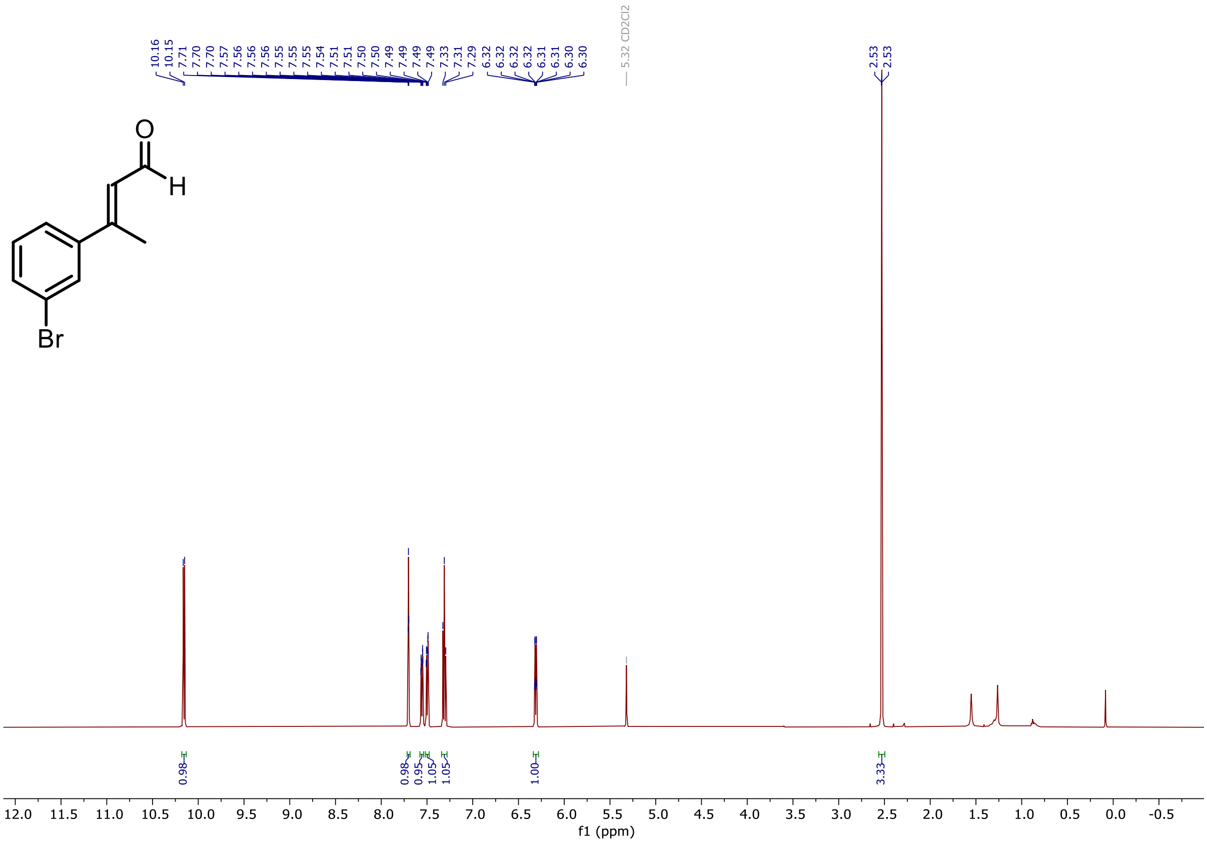

^{13}C NMR spectrum of 1b

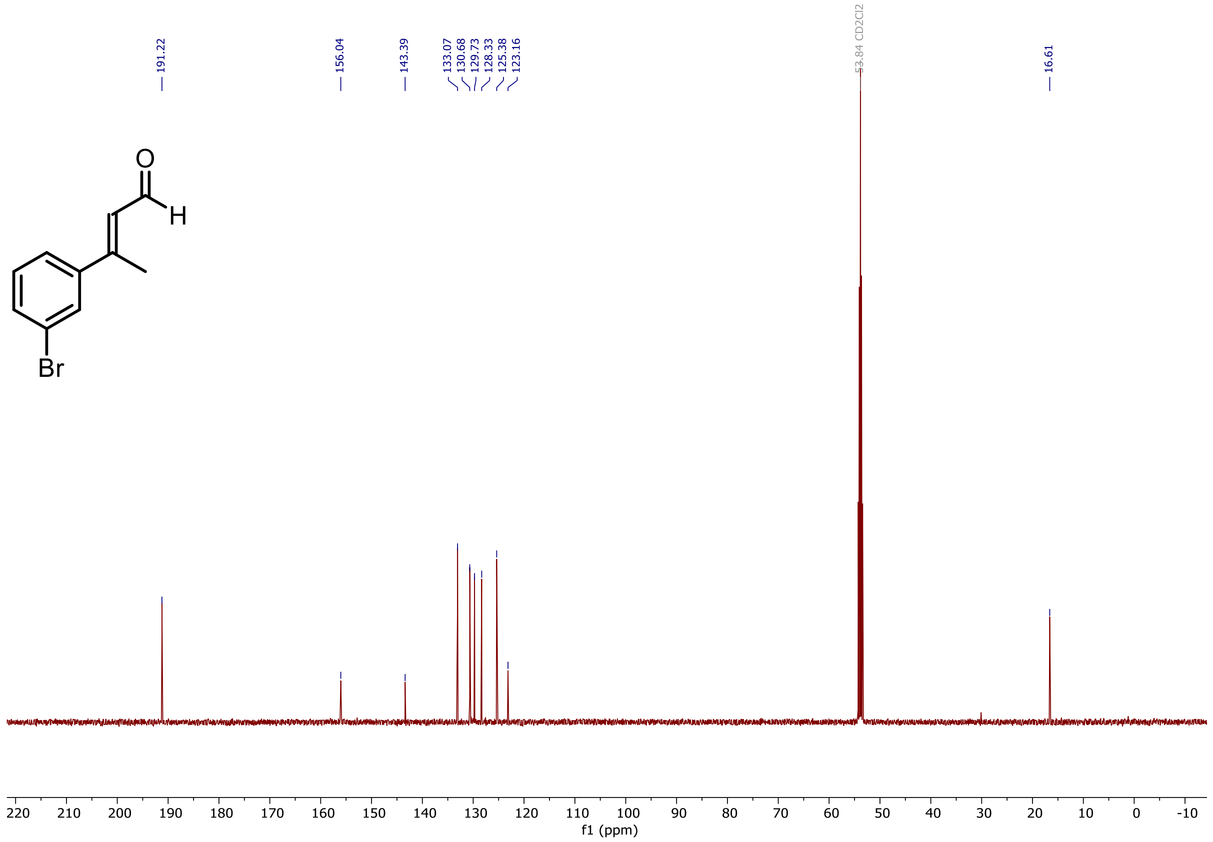

^{1}H NMR spectrum of 1c

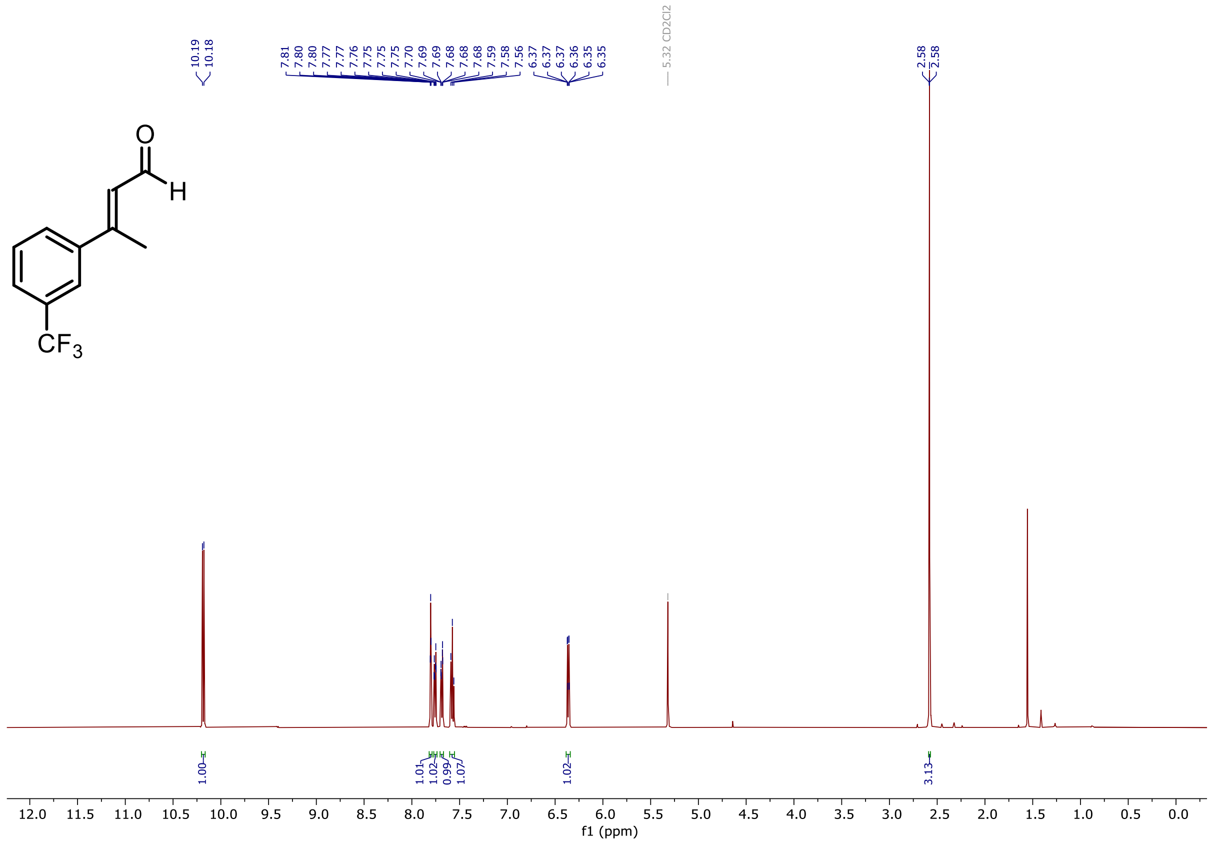

^{13}C NMR spectrum of 1c

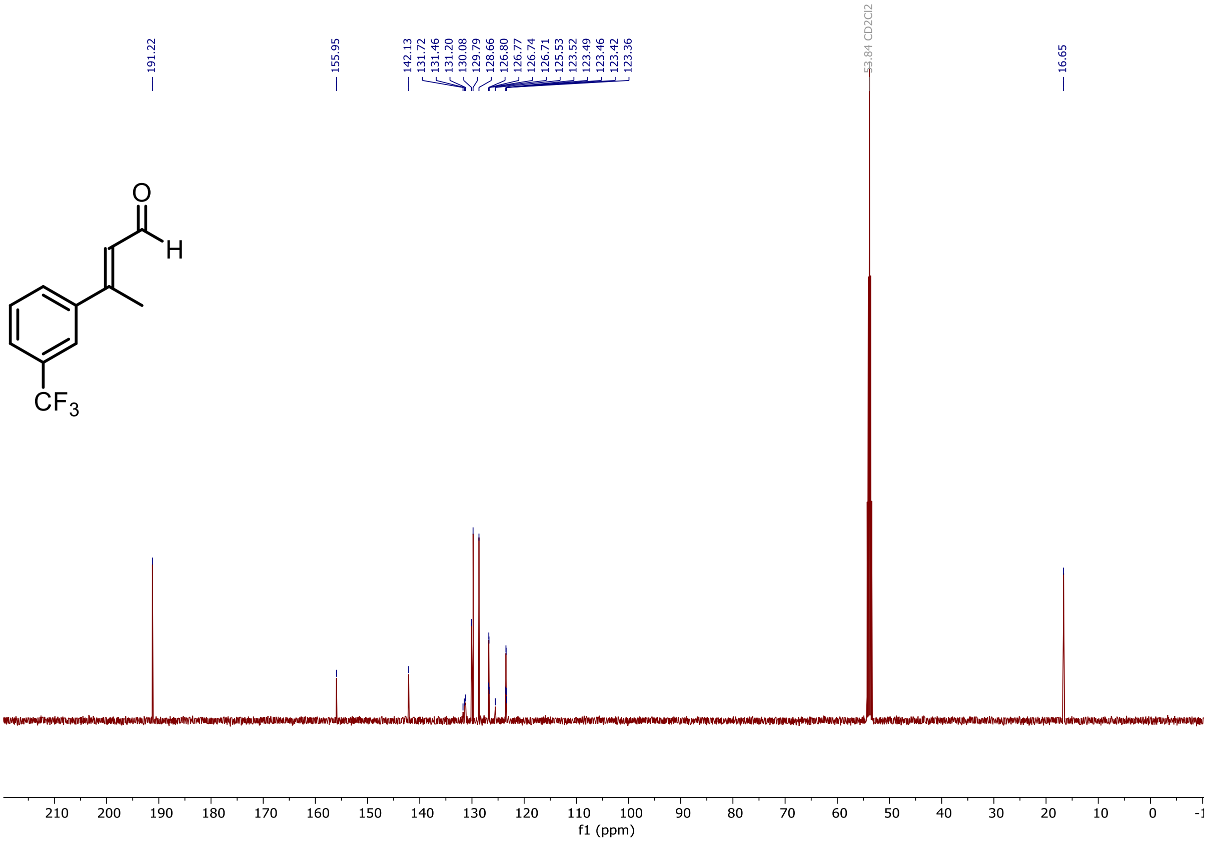

^{19}F{^{1}H} NMR spectrum of 1c

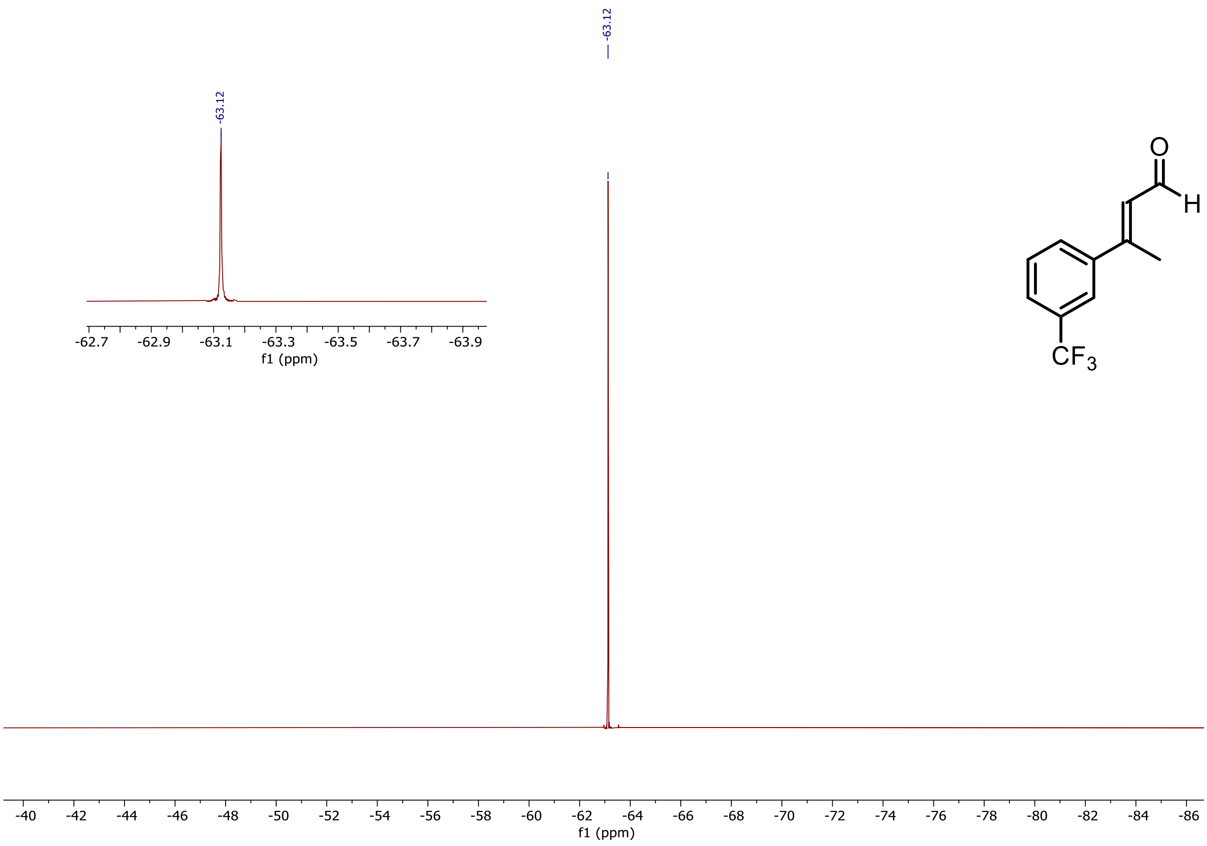

^{1}H NMR spectrum of (*E*)-1d

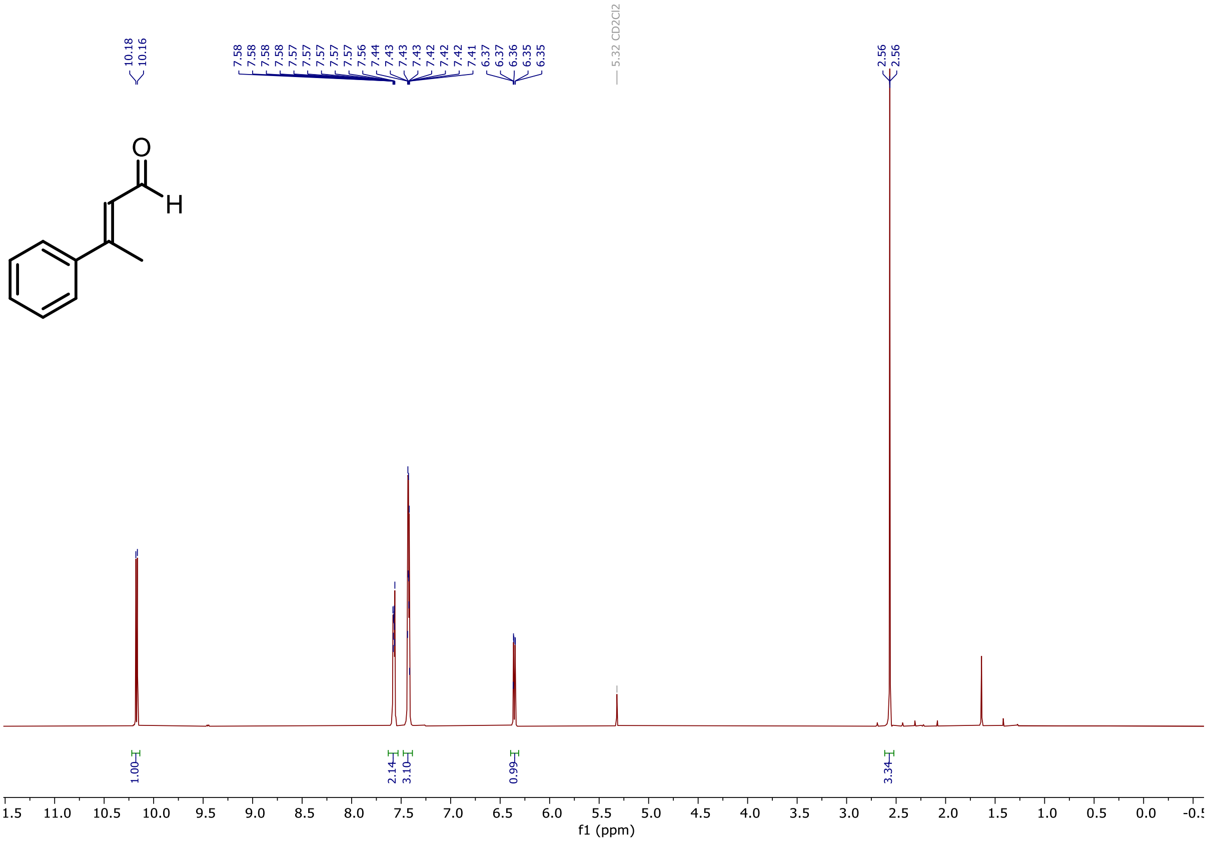

^{13}C NMR spectrum of (*E*)-1d

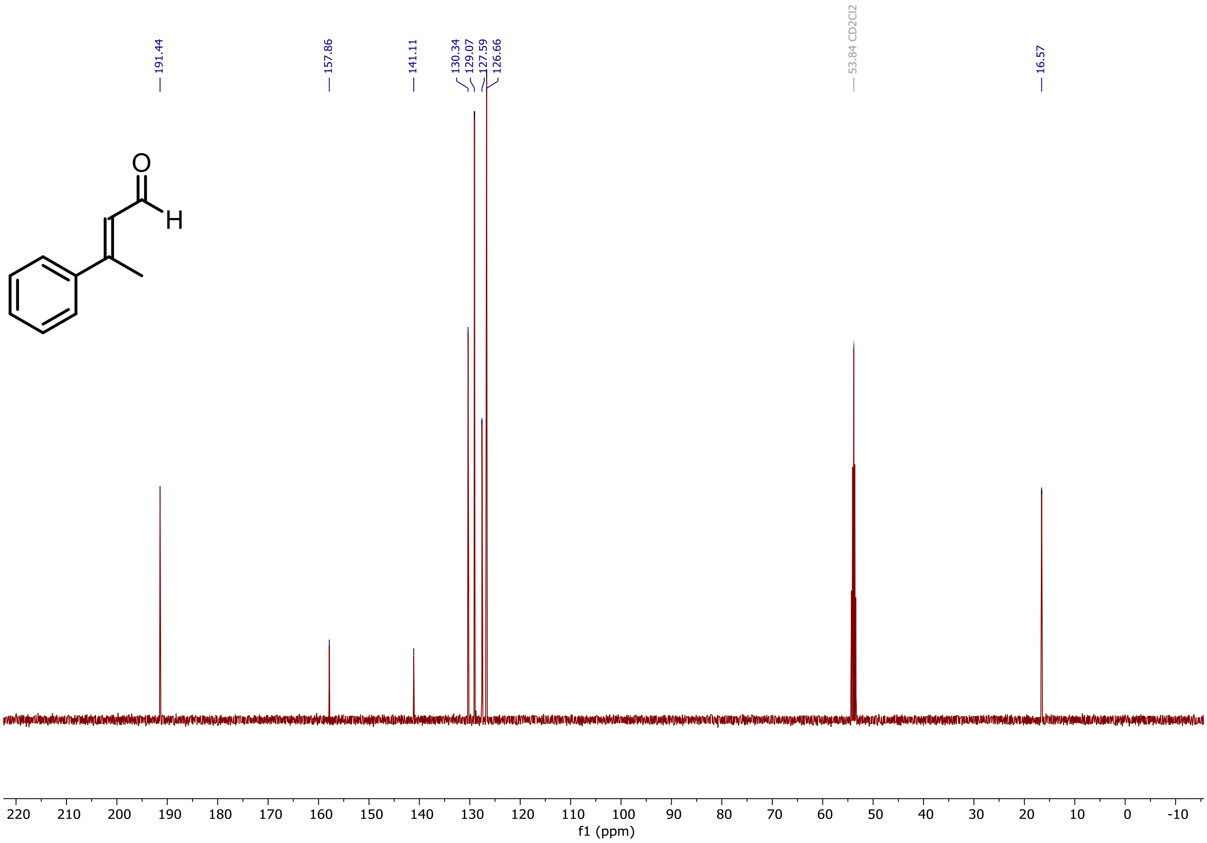

^{1}H NMR spectrum of (*Z*)-1d

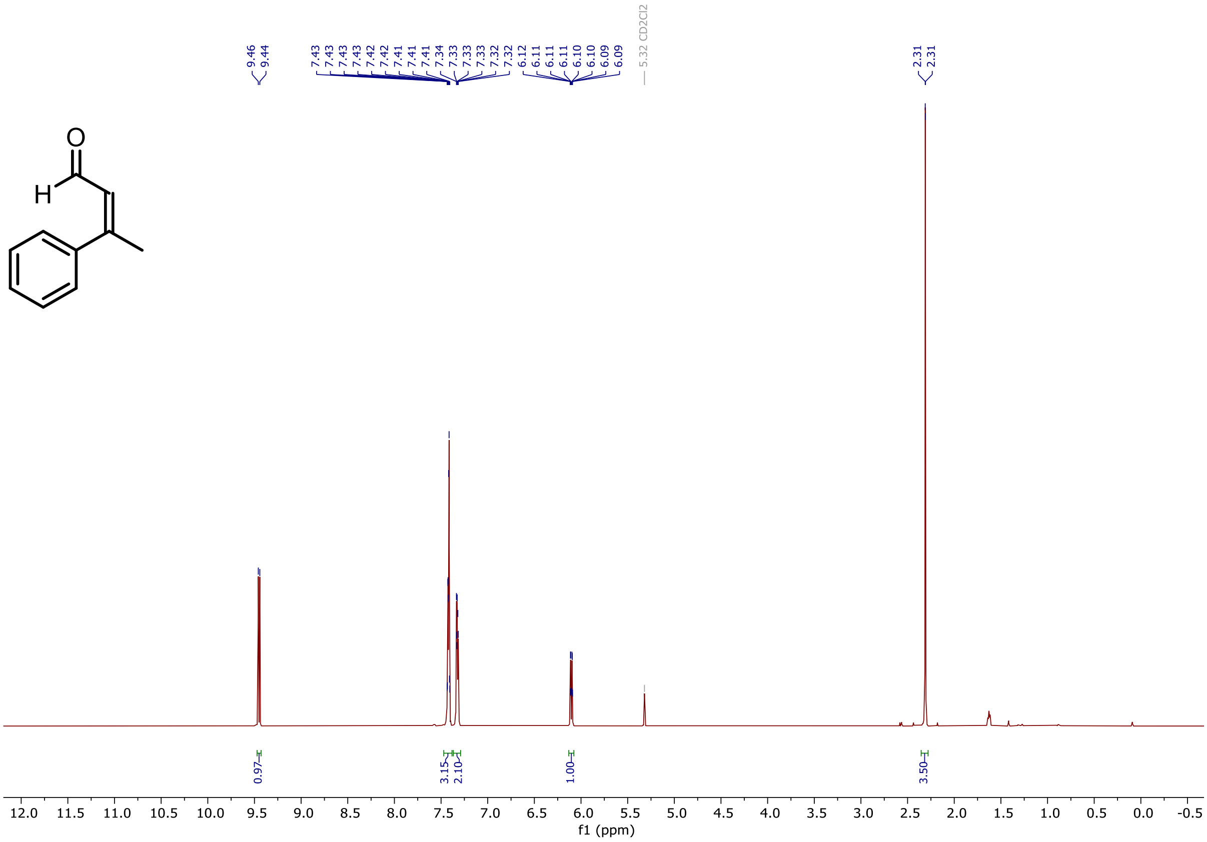

^{13}C NMR spectrum of (*Z*)-1d

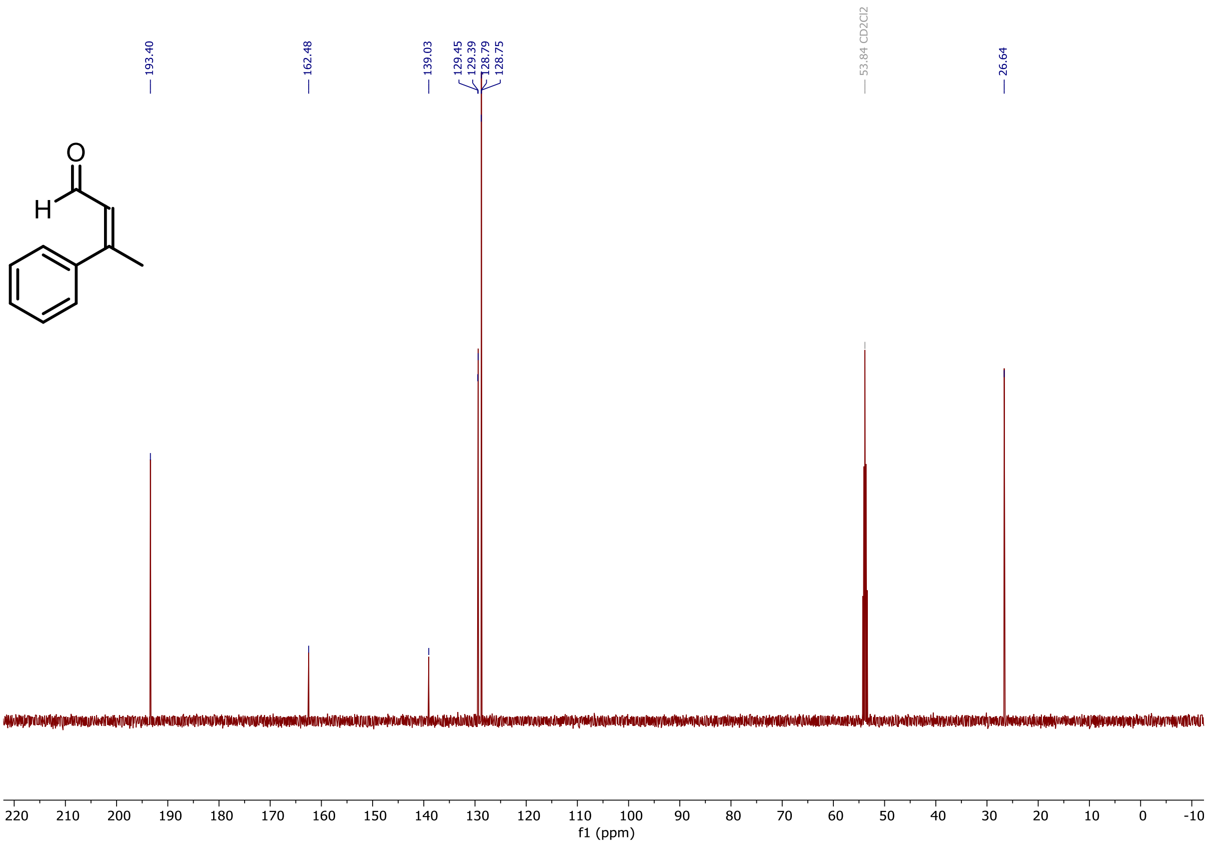

^{1}H NMR spectrum of 1e

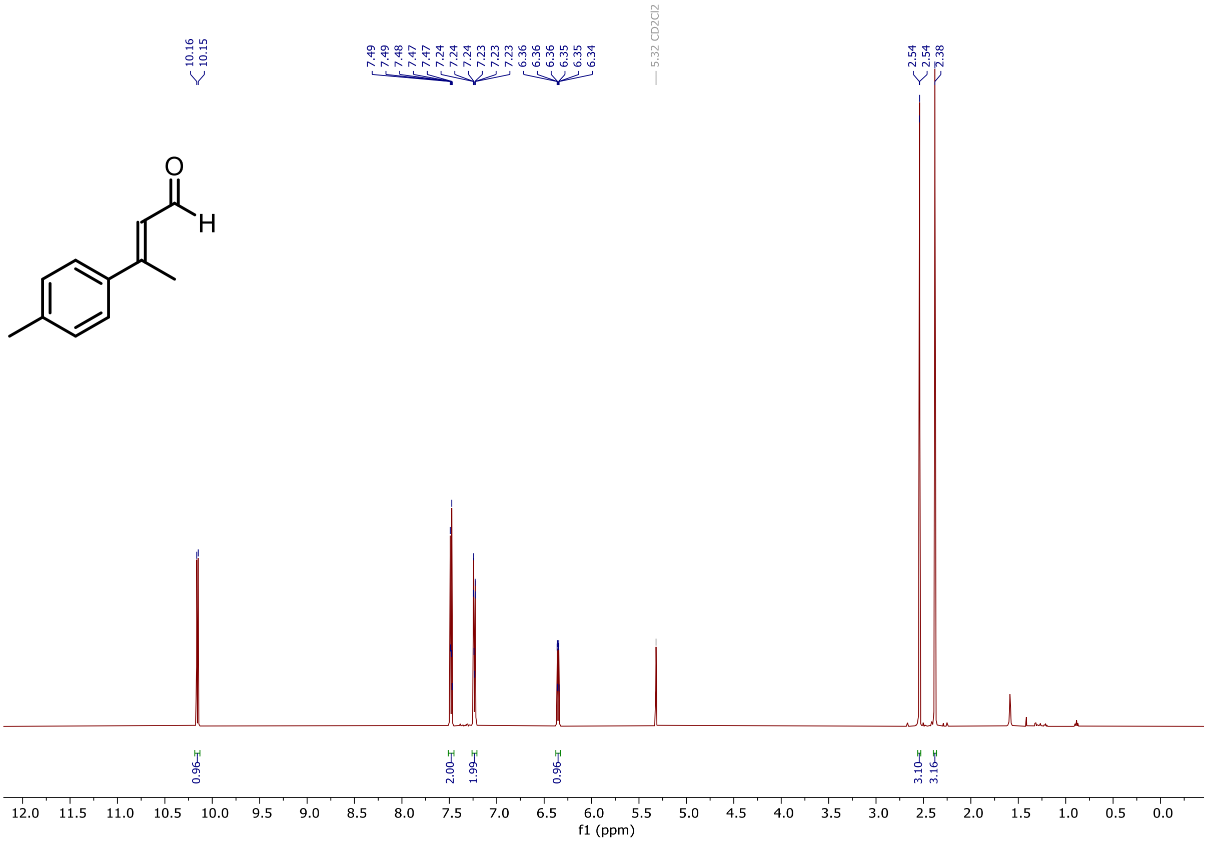

^{13}C NMR spectrum of 1e

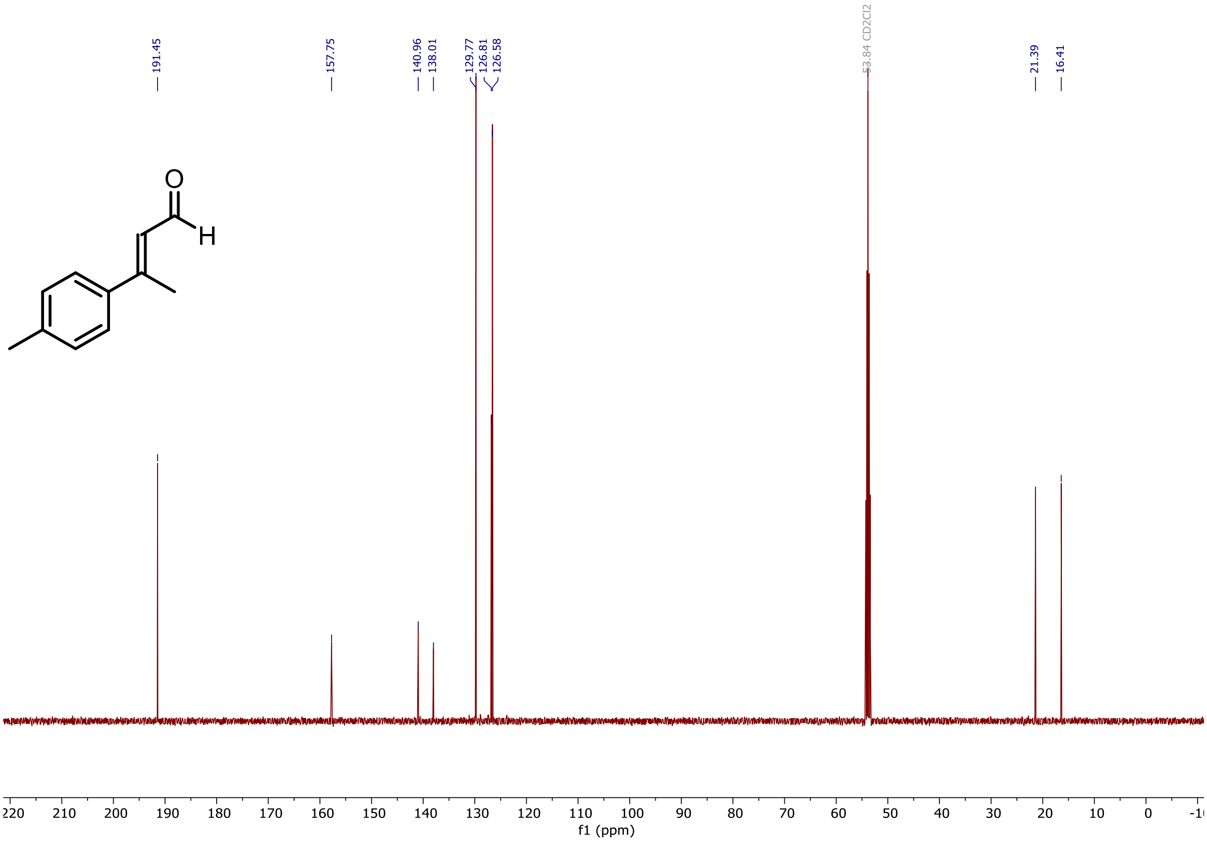

^{1}H NMR spectrum of 1f

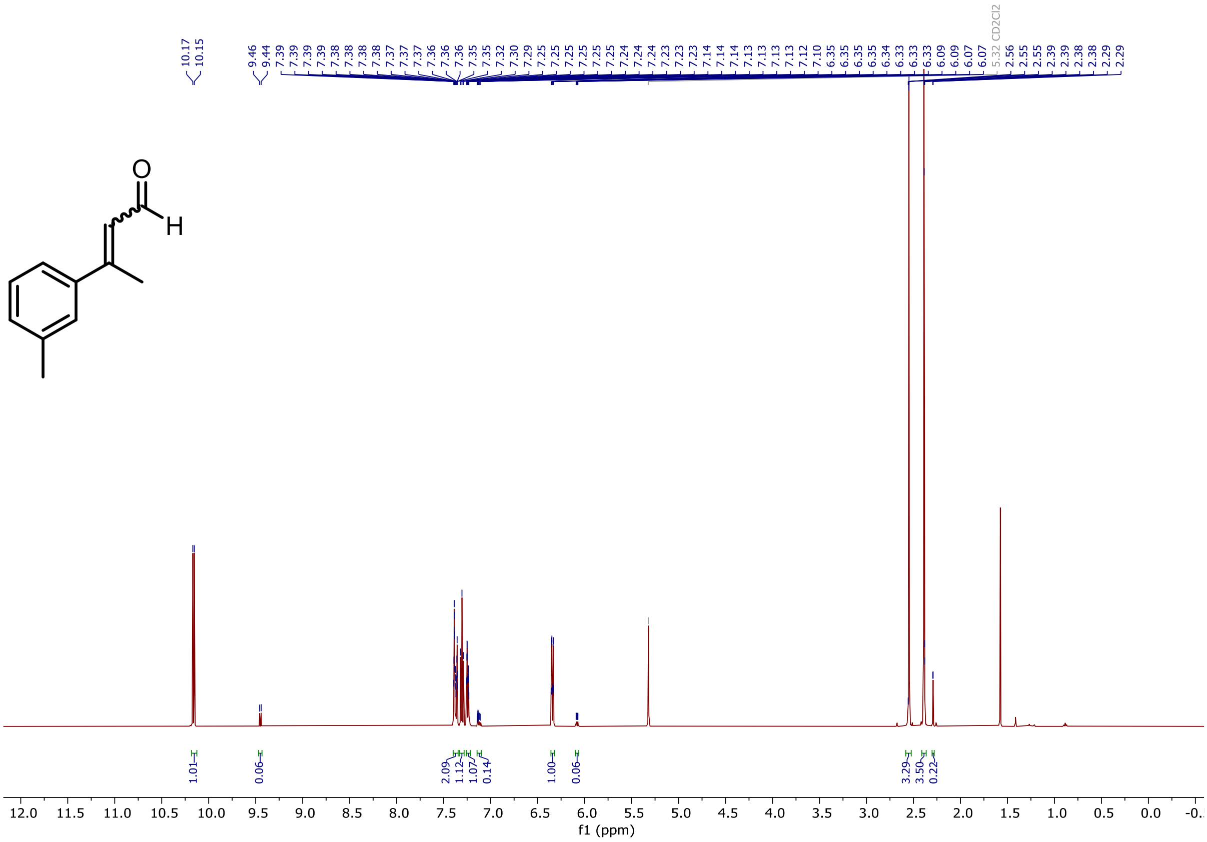

^{13}C NMR spectrum of 1f

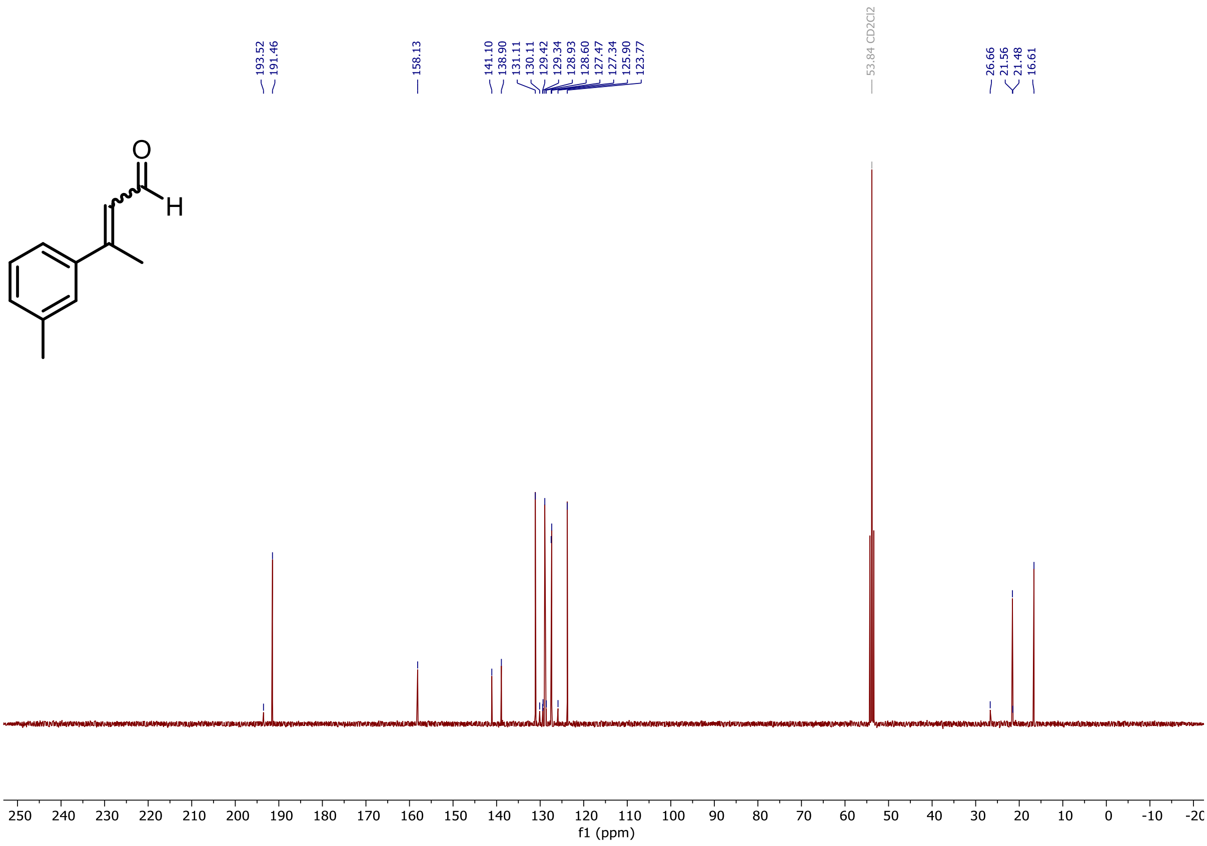

^1H NMR spectrum of 1g

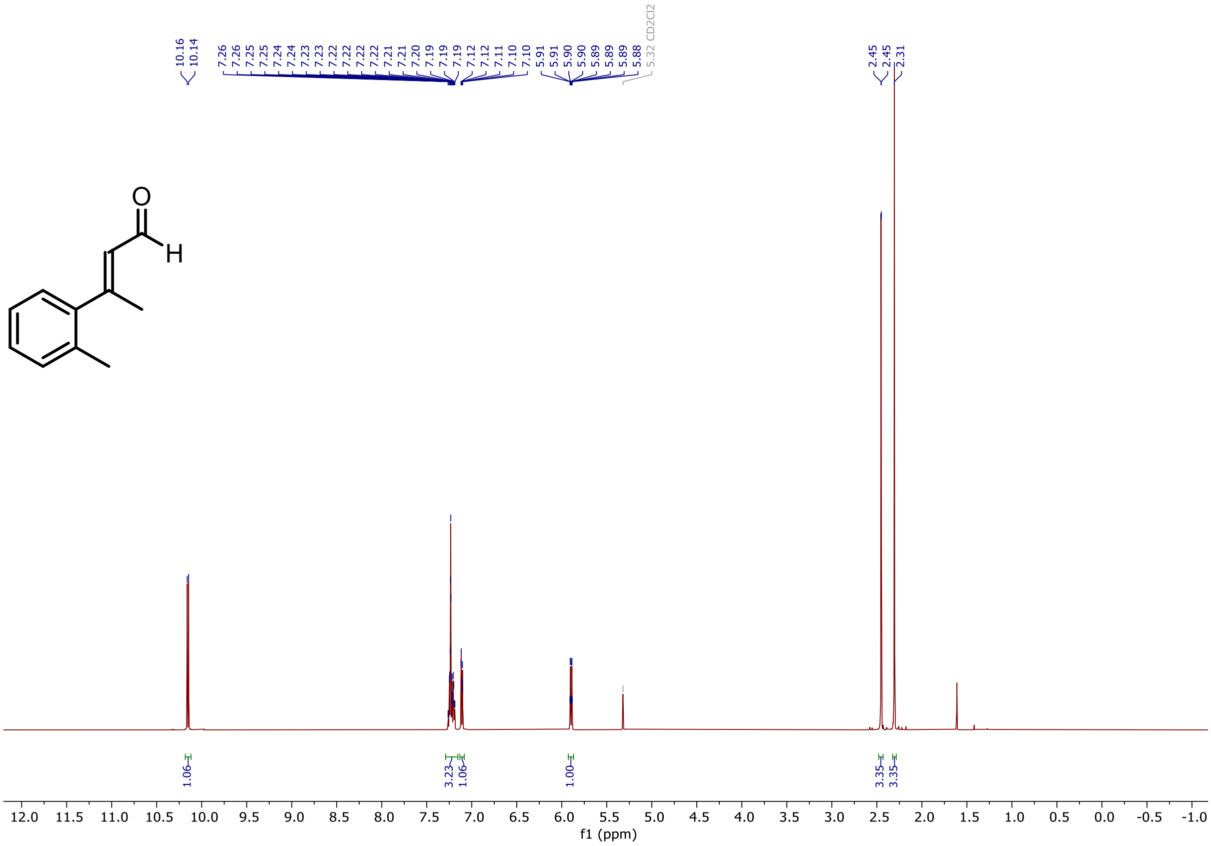

^{13}C NMR spectrum of 1g

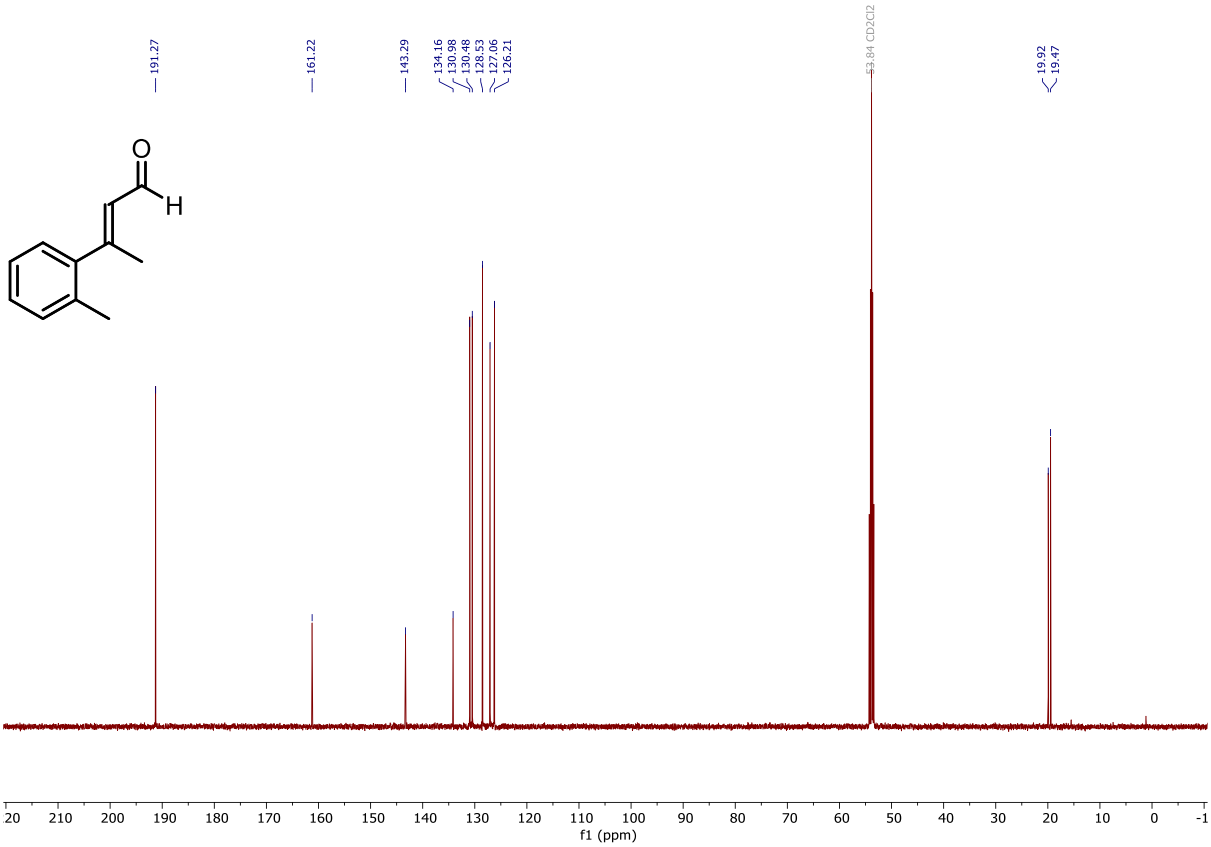

^{1}H NMR spectrum of 1h

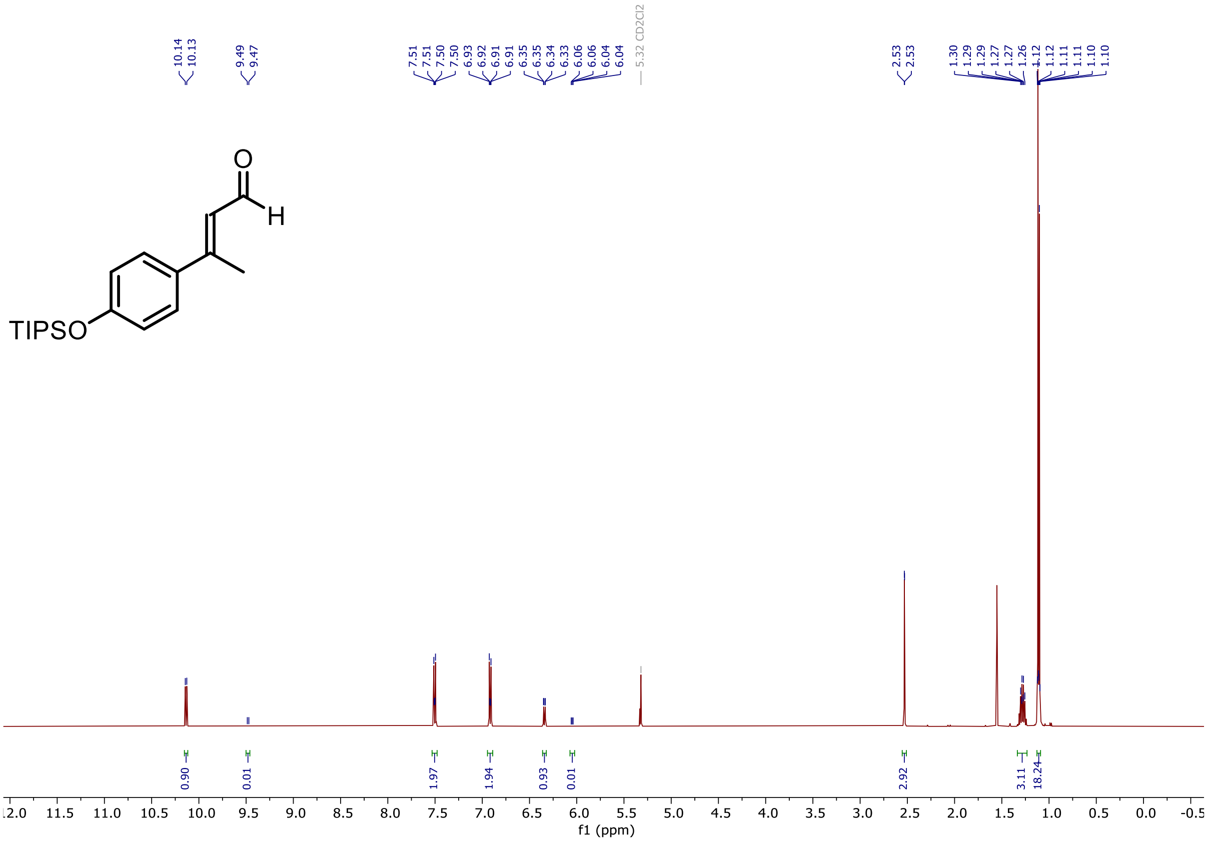

^{13}C NMR spectrum of 1h

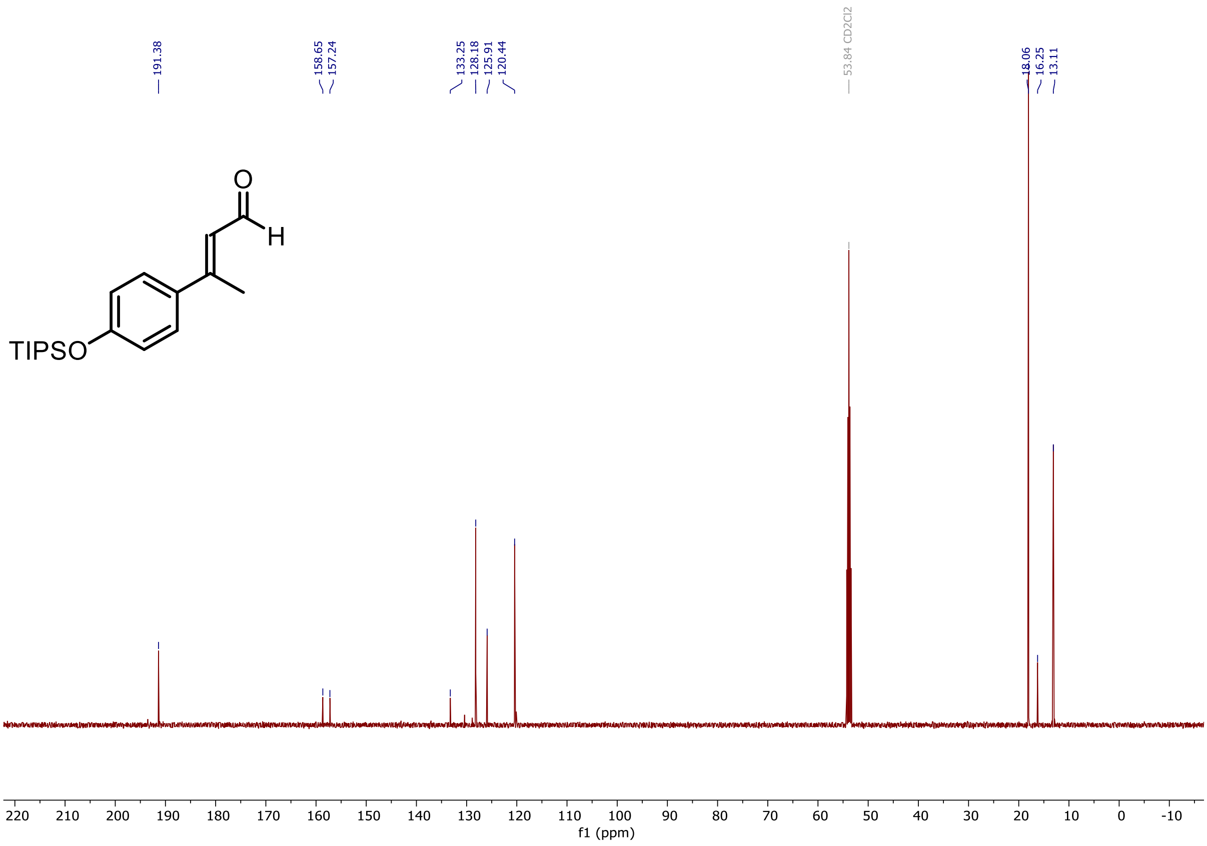

^{1}H NMR spectrum of 1i

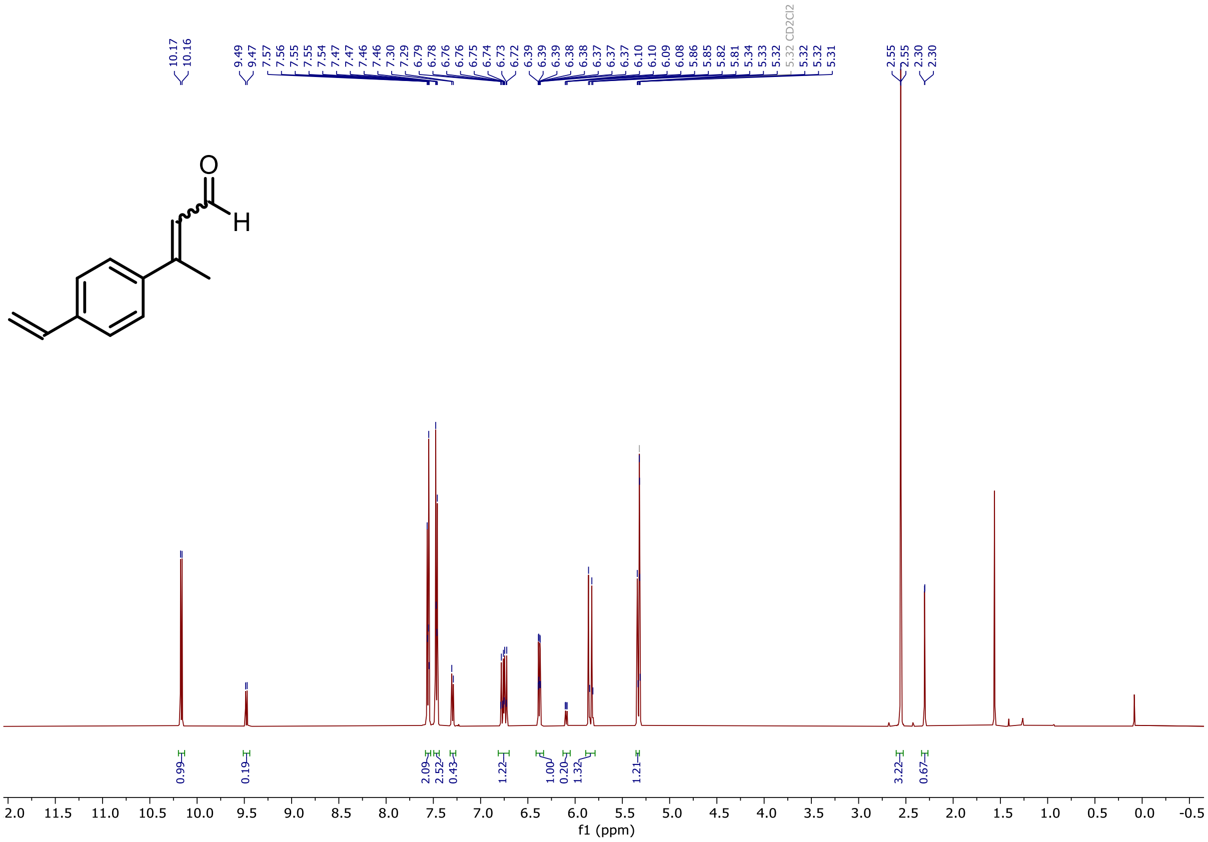

^{13}C NMR spectrum of 1i

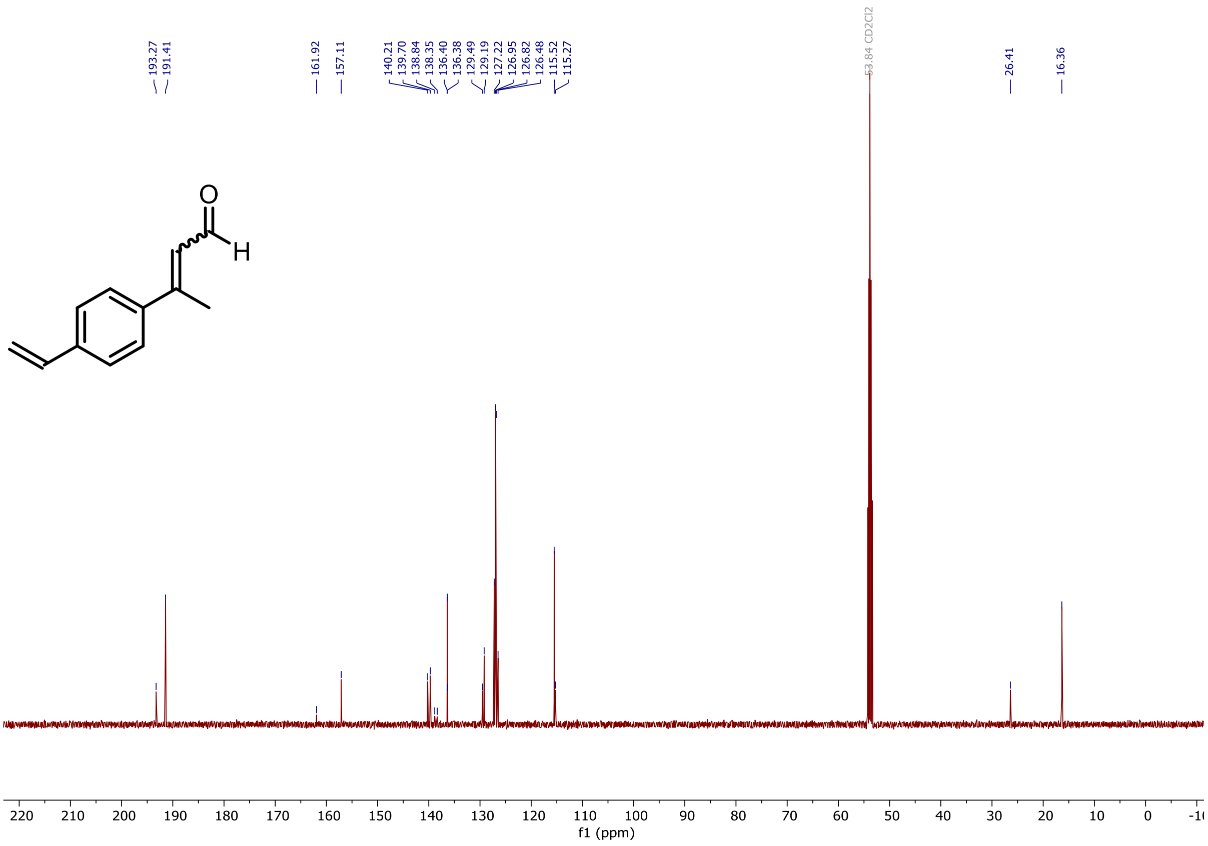

^{1}H NMR spectrum of 1j

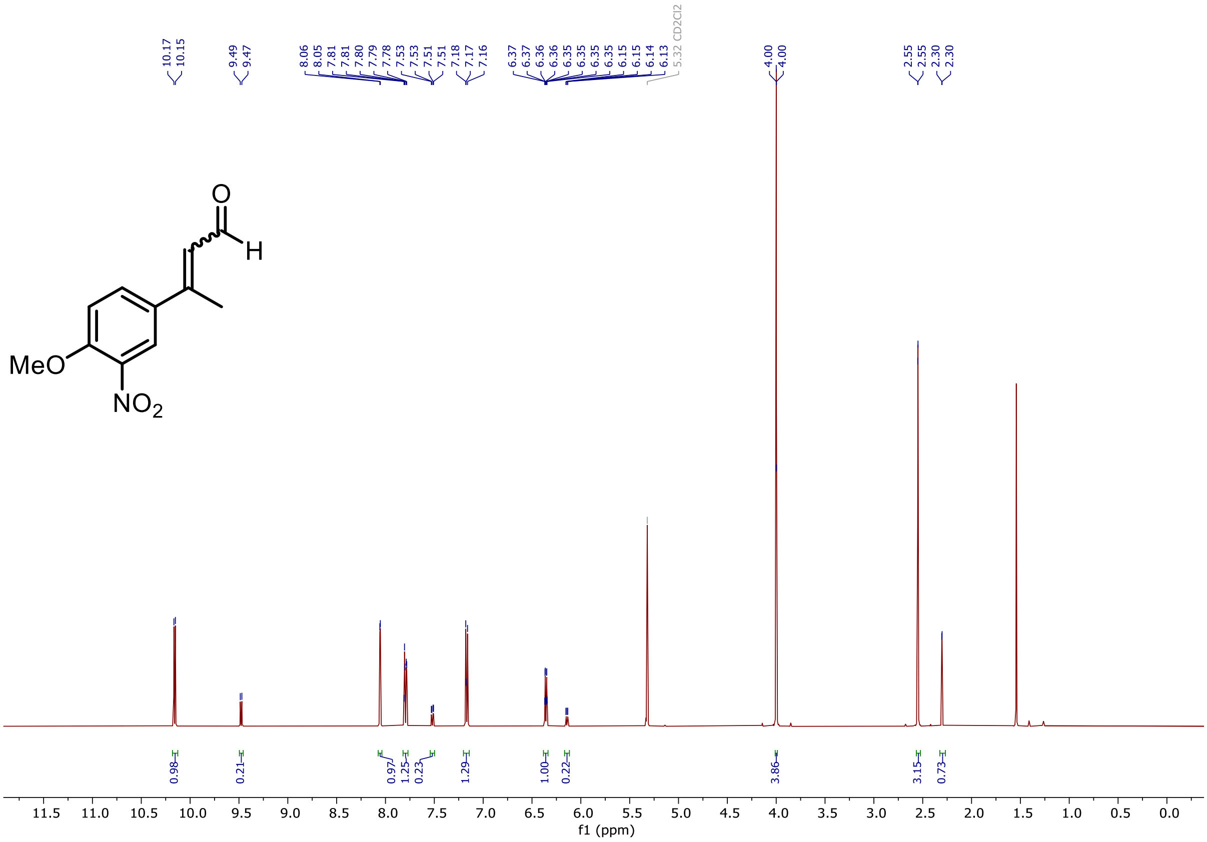

^{13}C NMR spectrum of 1j

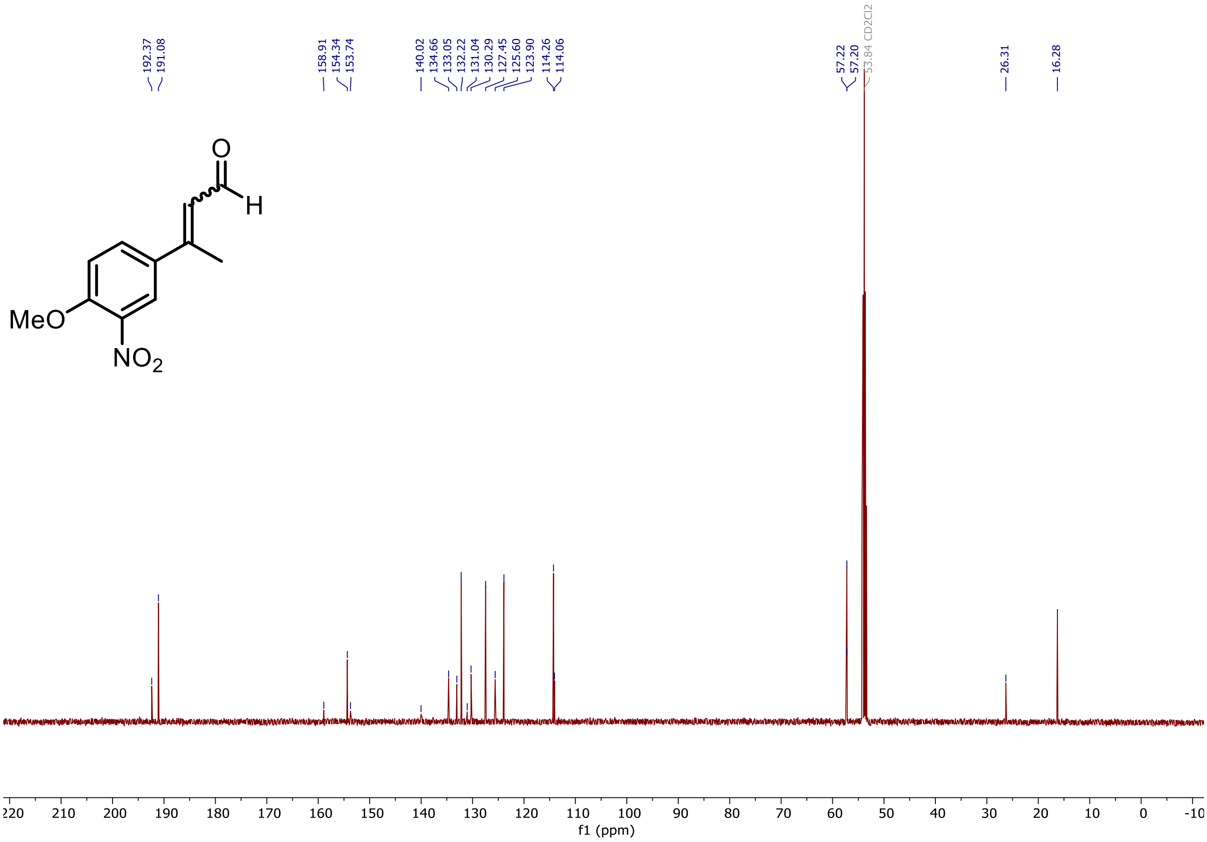

^{1}H NMR spectrum of 1k

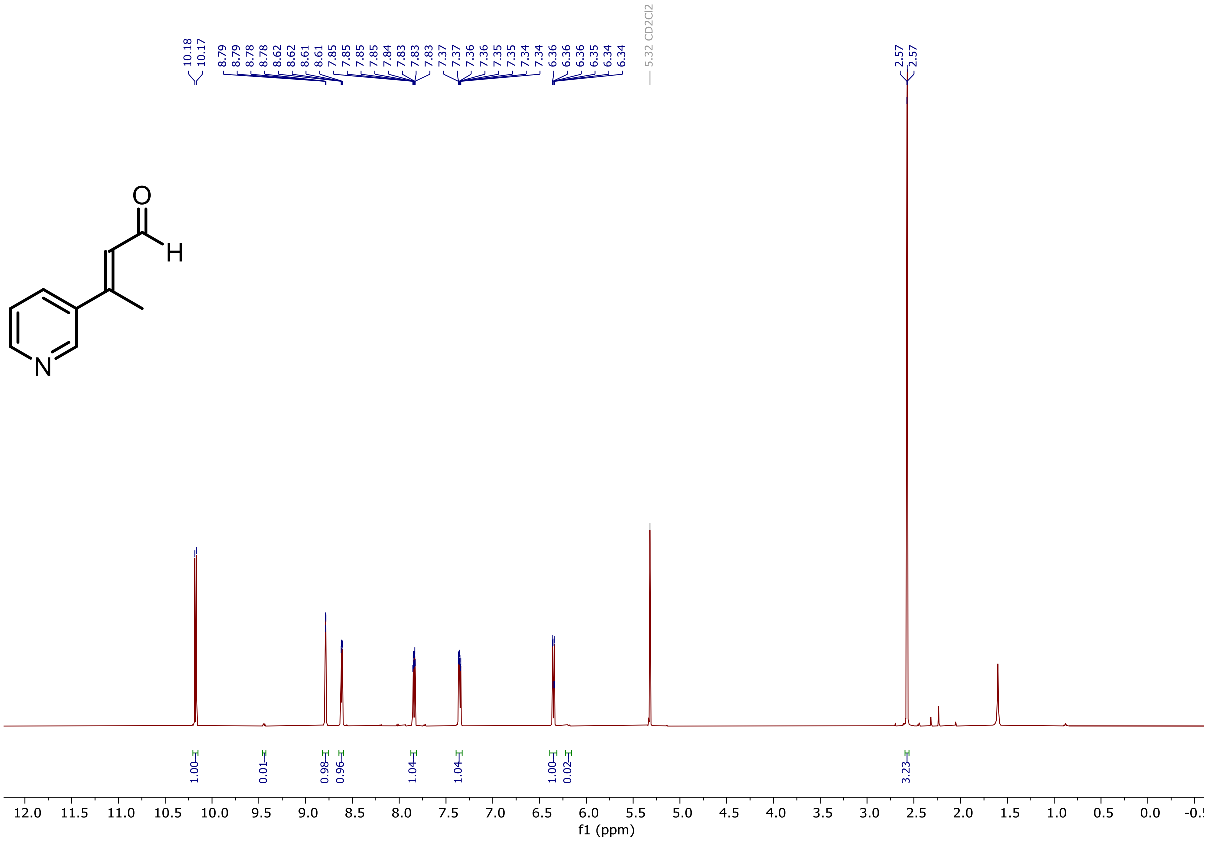

^{13}C NMR spectrum of 1k

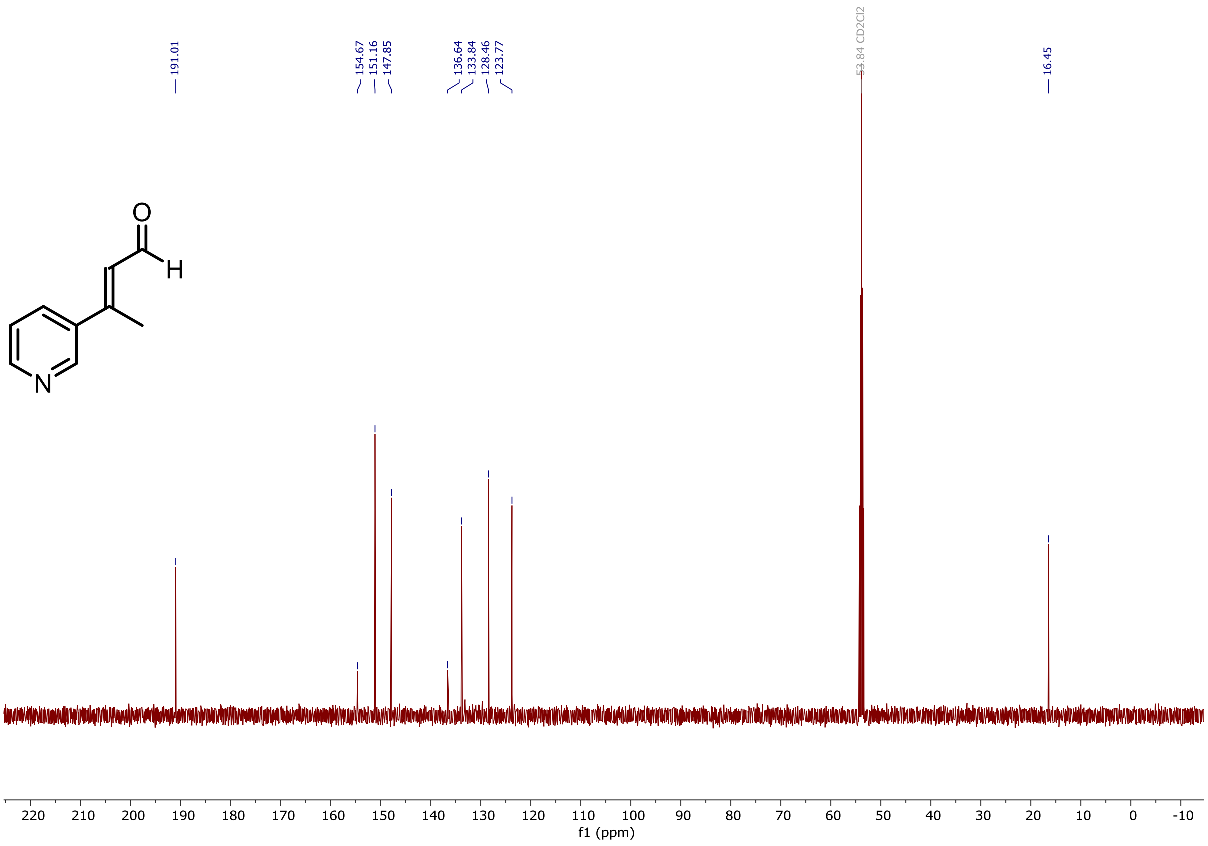

^{1}H NMR spectrum of 1l

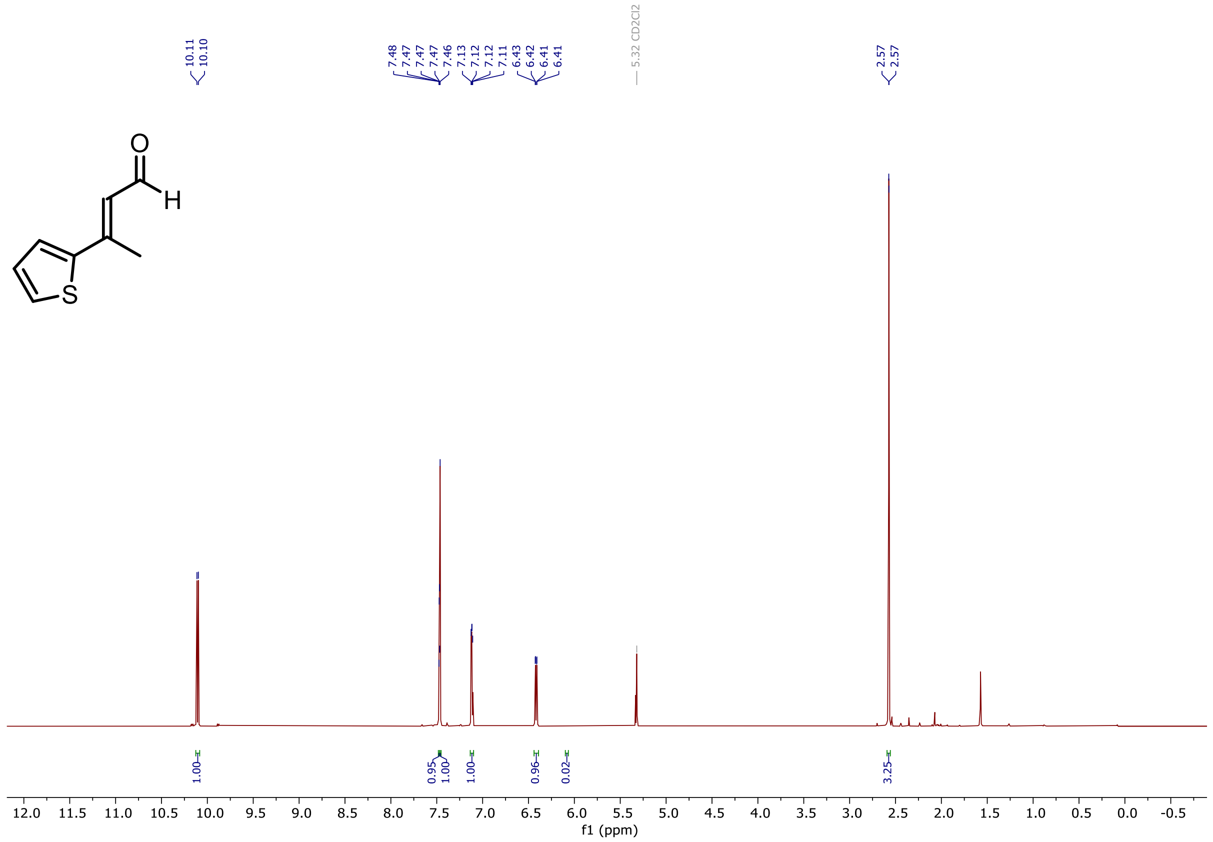

^{13}C NMR spectrum of 1l

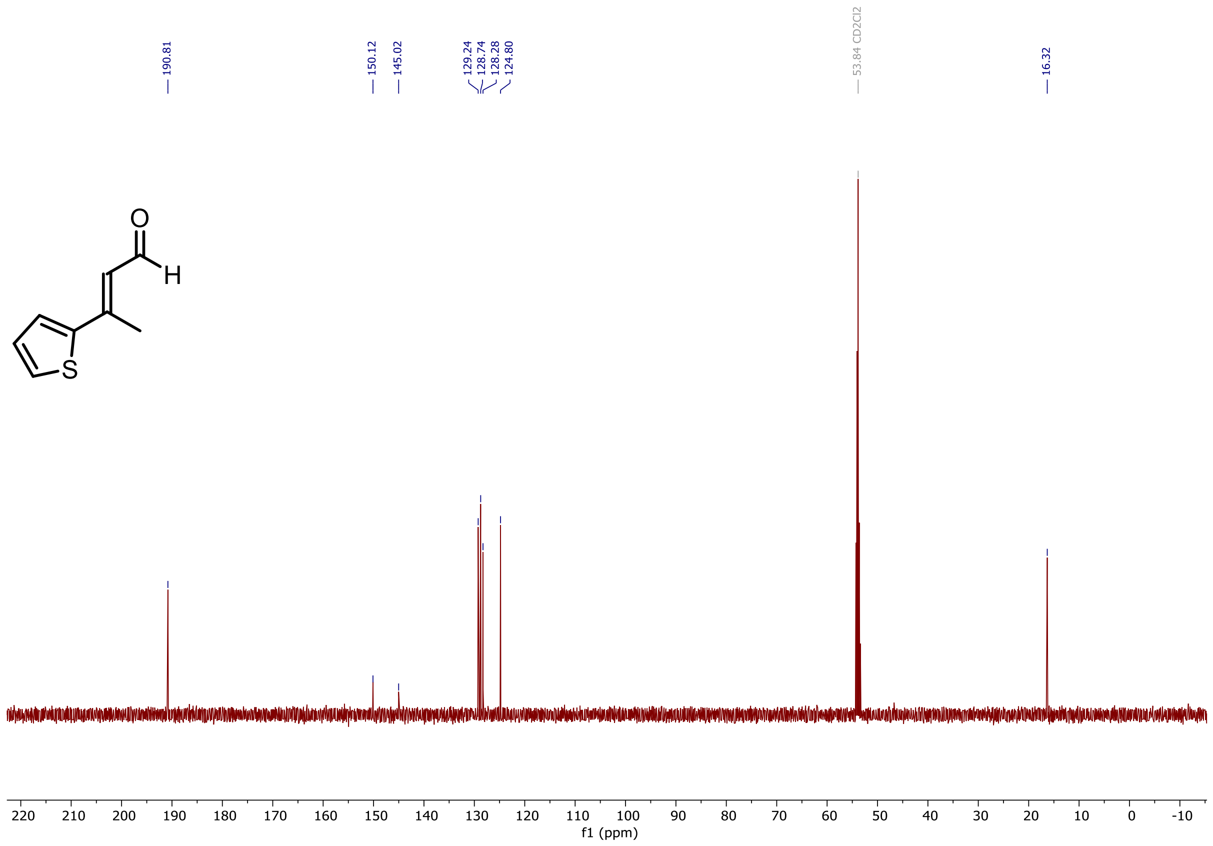

^{1}H NMR spectrum of 1m

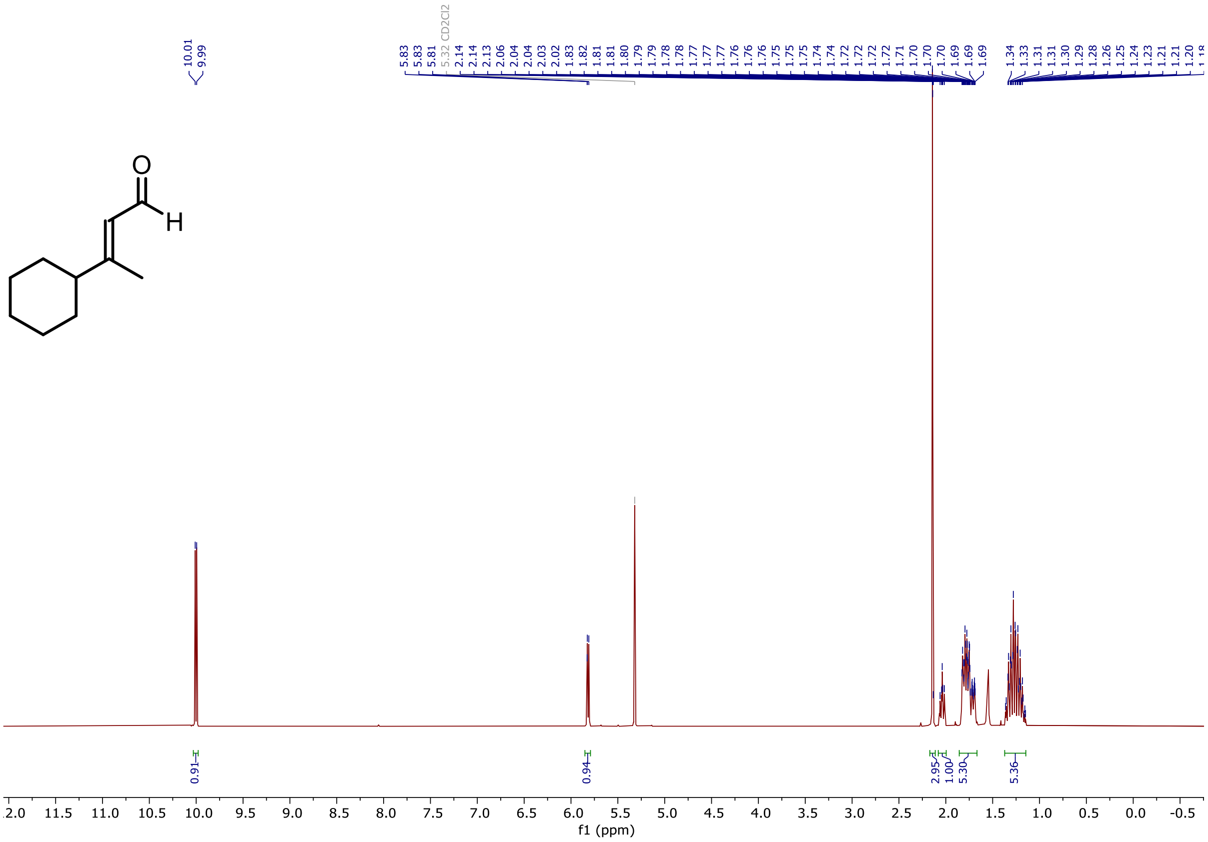

^{13}C NMR spectrum of 1m

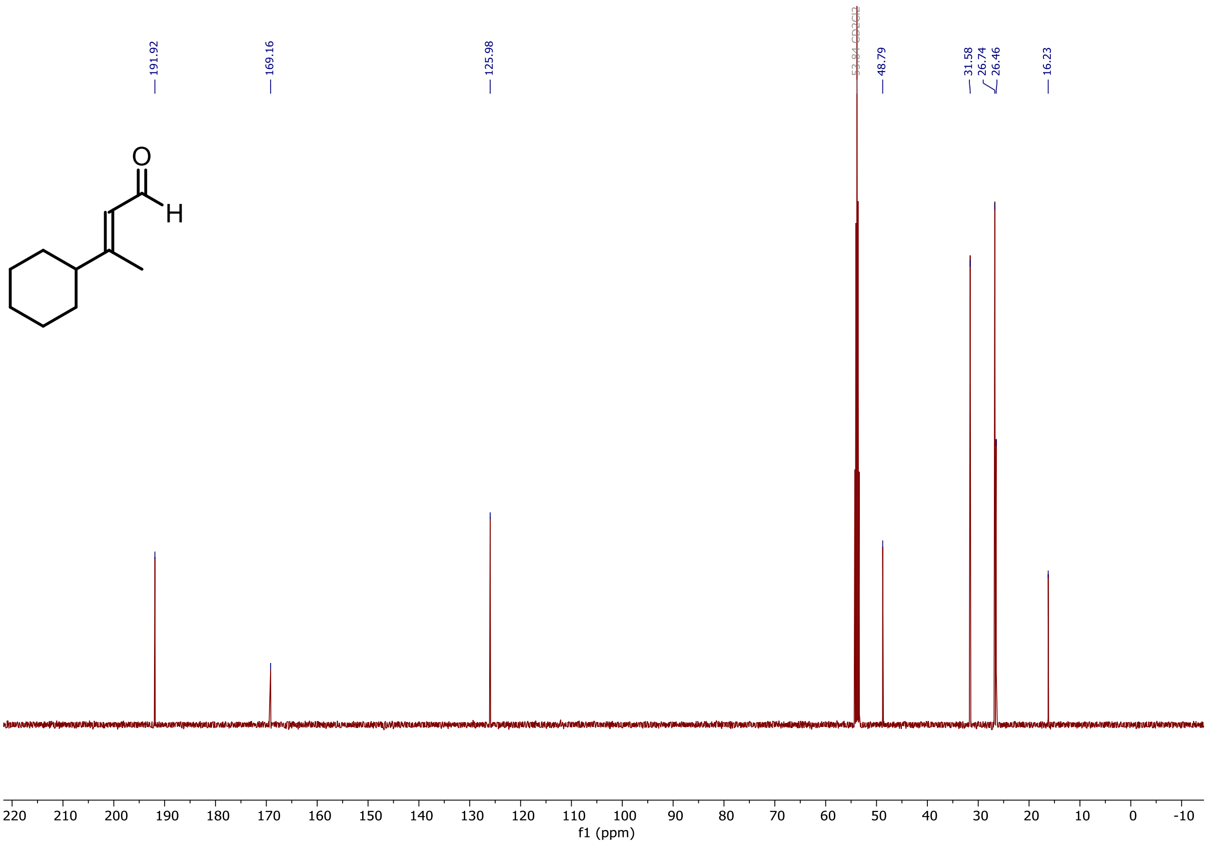

^{1}H NMR spectrum of 1n

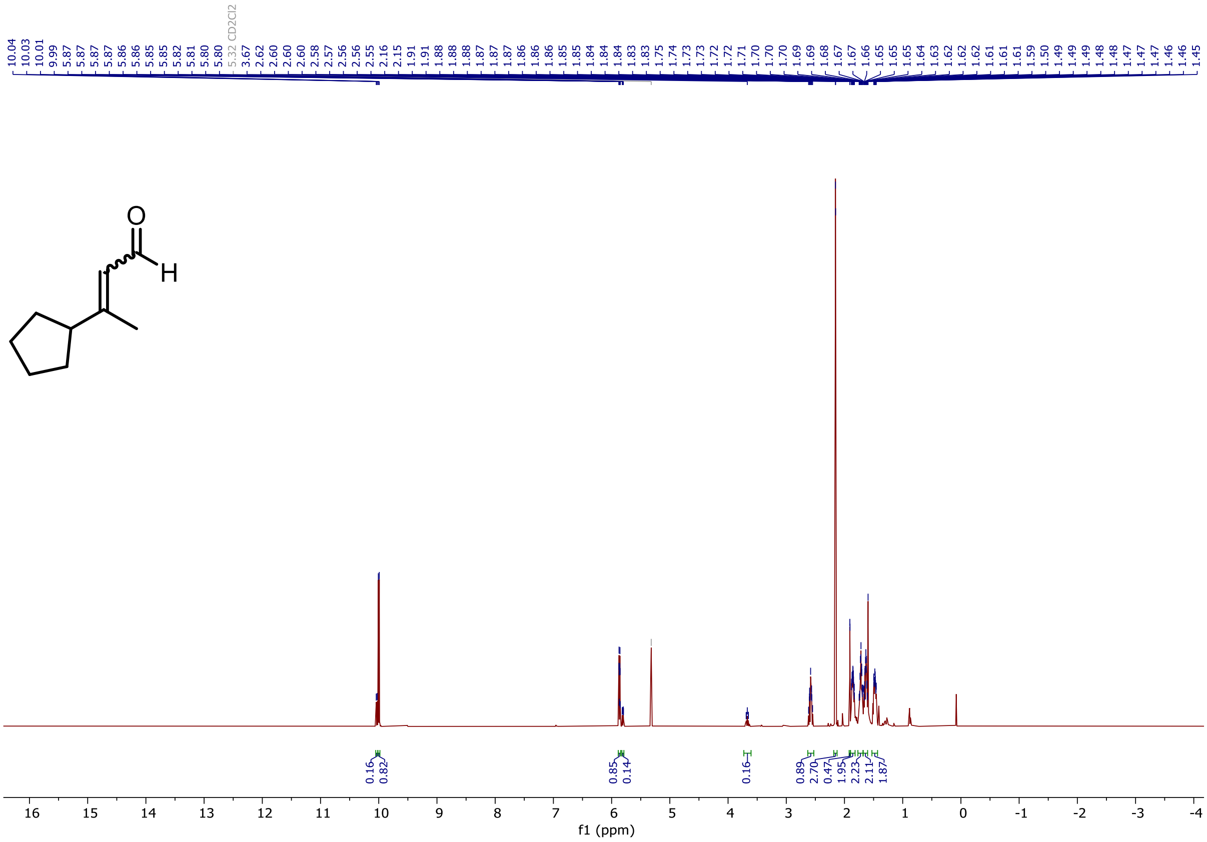

^{13}C NMR spectrum of 1n

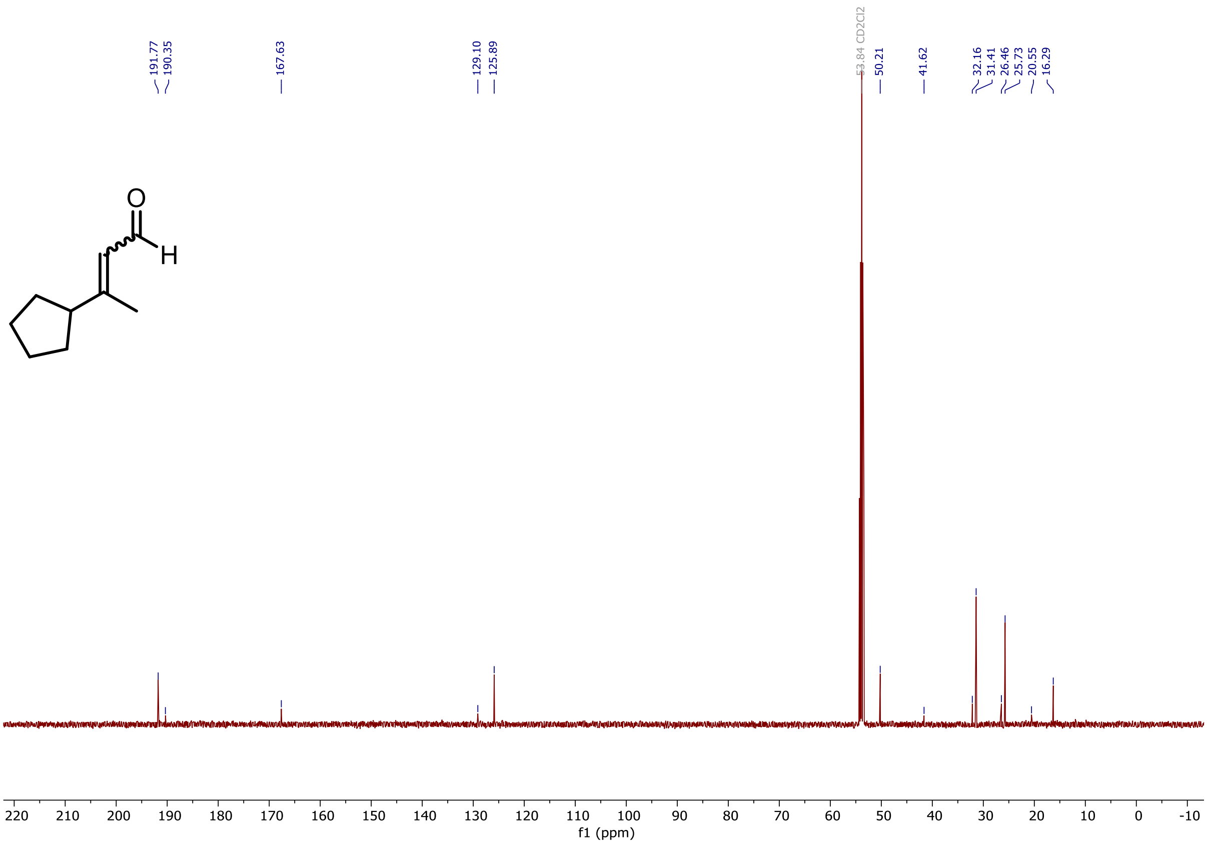

^{1}H NMR spectrum of 1o

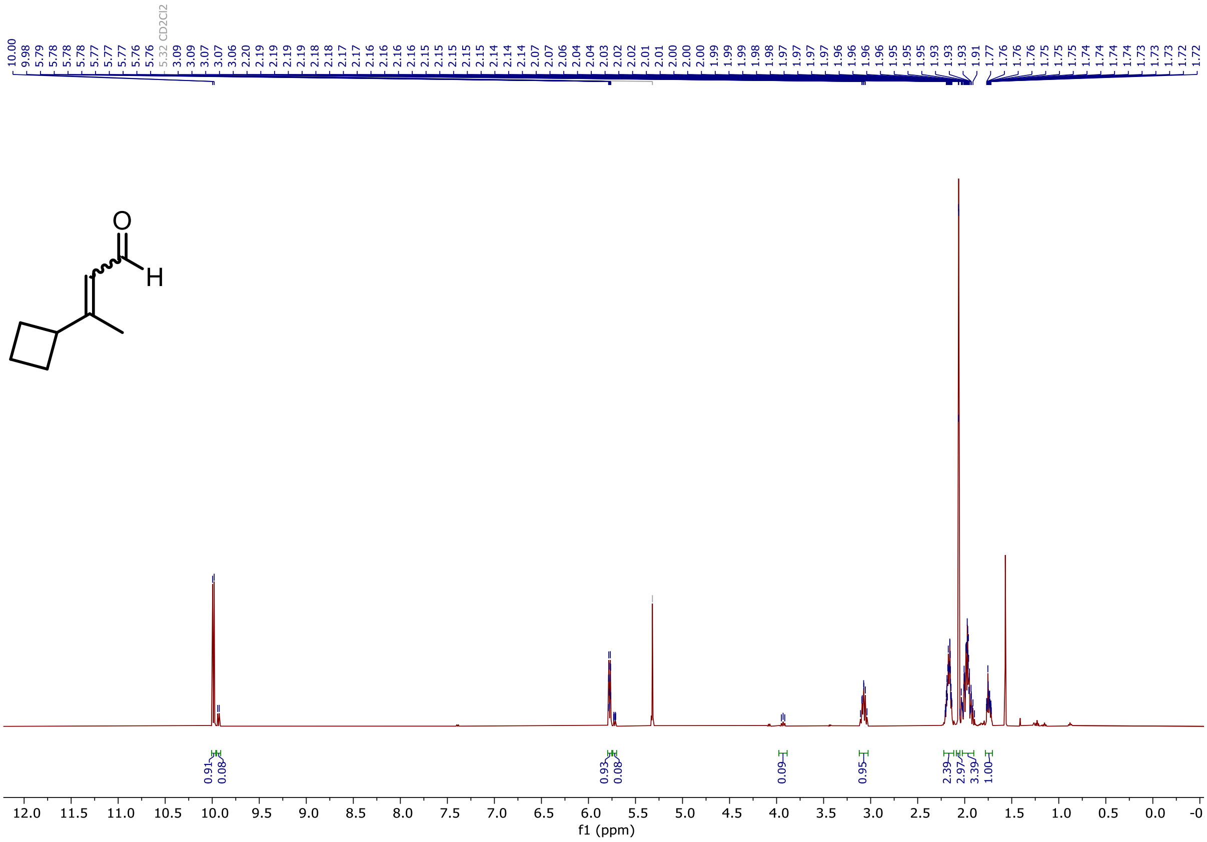

^{13}C NMR spectrum of 1o

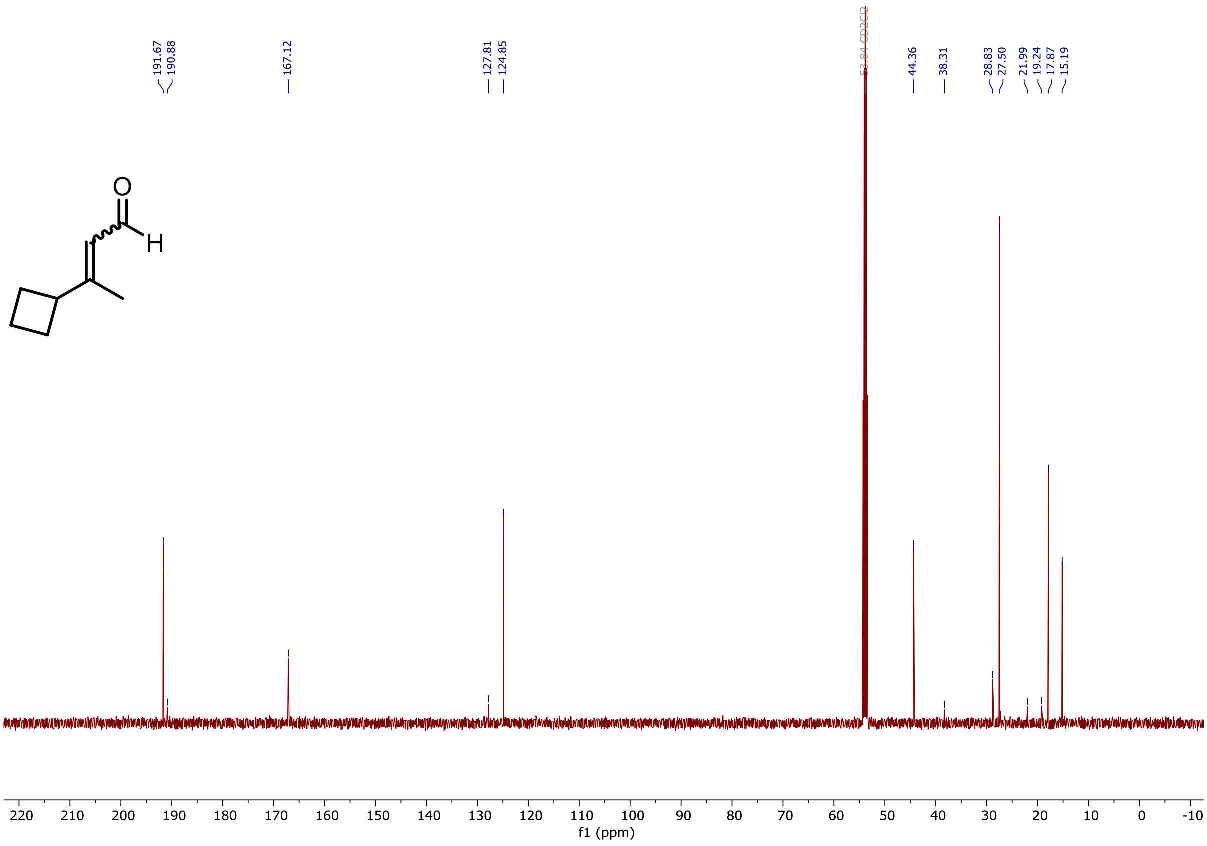

^{1}H NMR spectrum of 1p

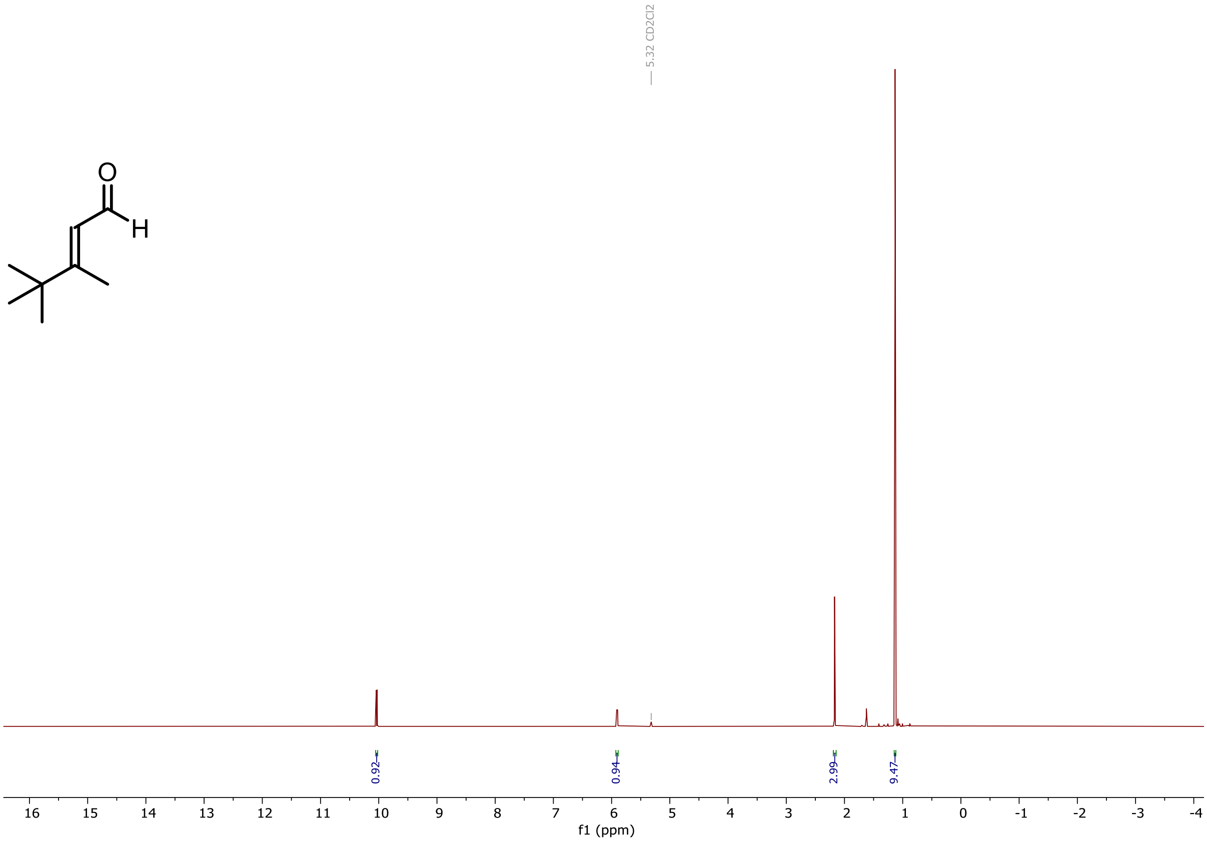

^{13}C NMR spectrum of 1p

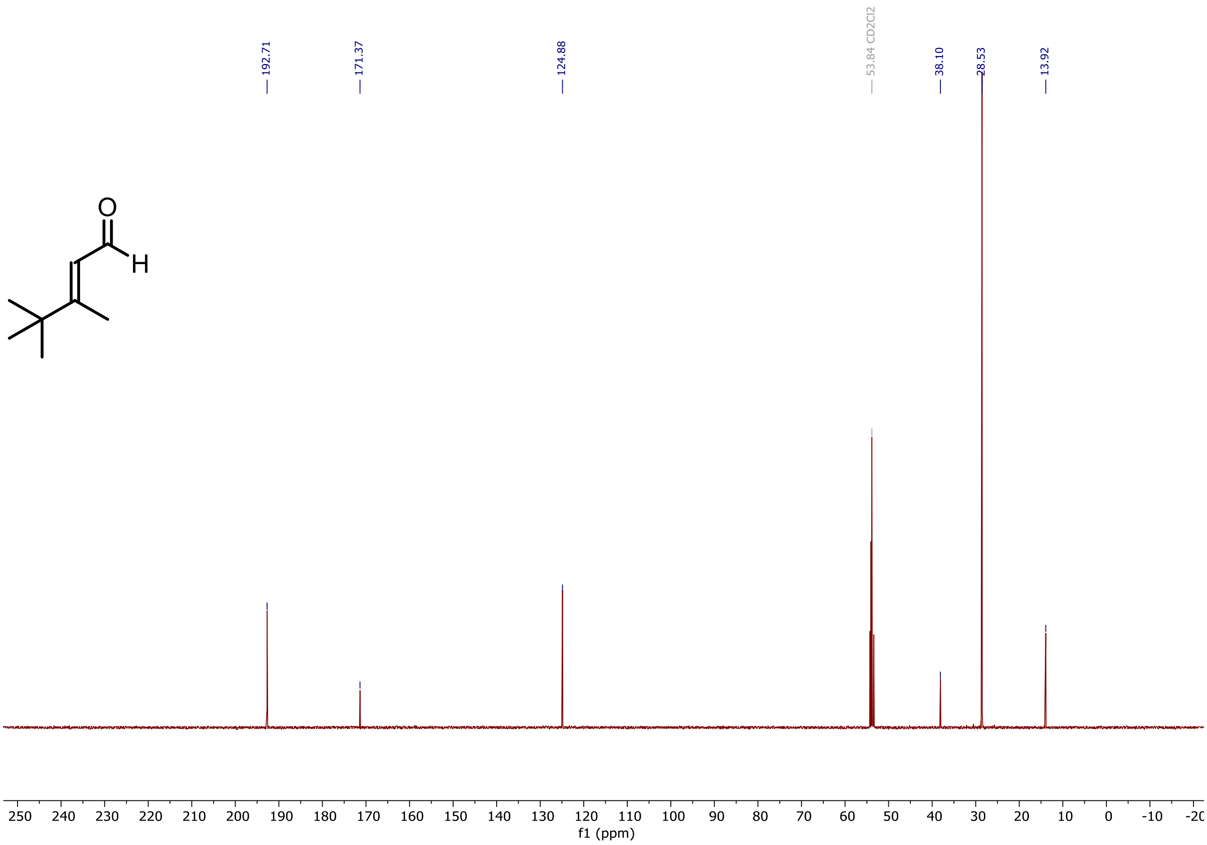

^{1}H NMR spectrum of 1q

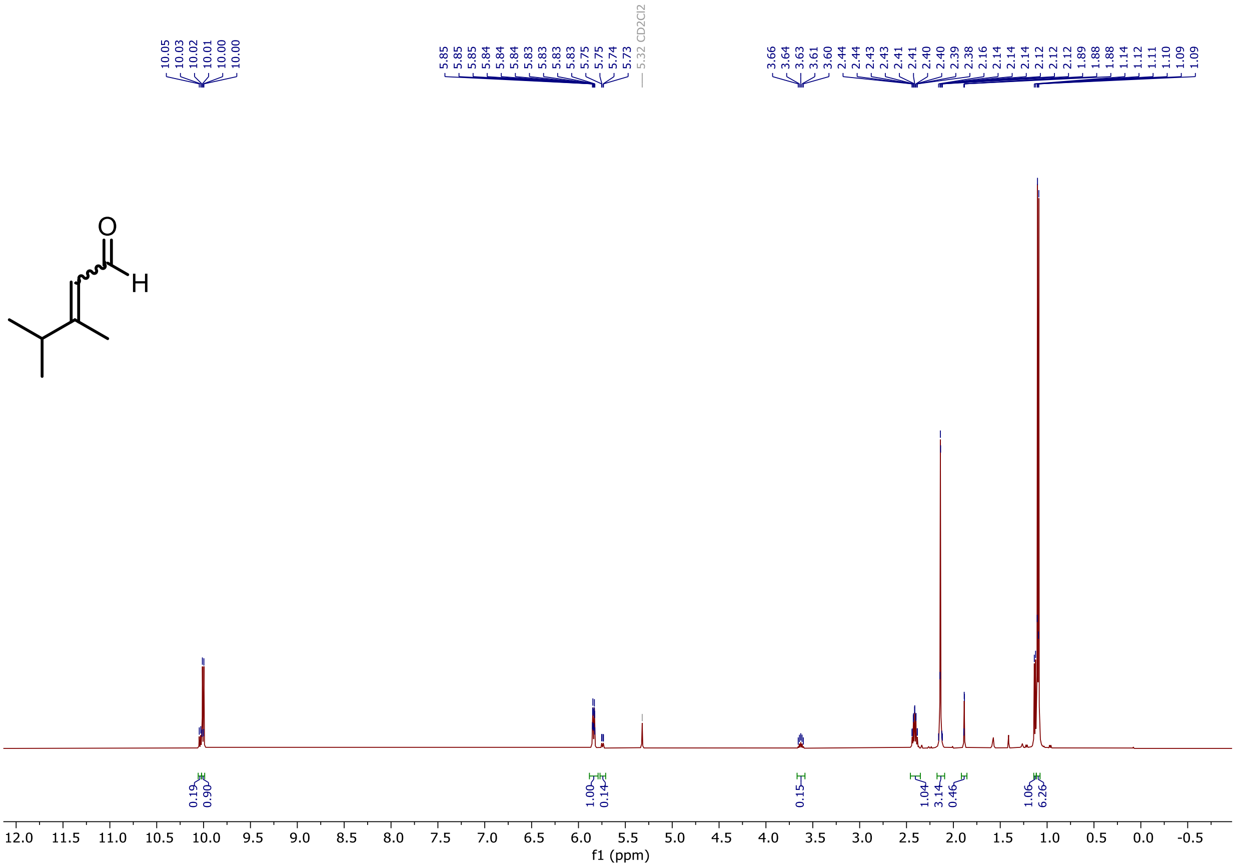

^{13}C NMR spectrum of 1q

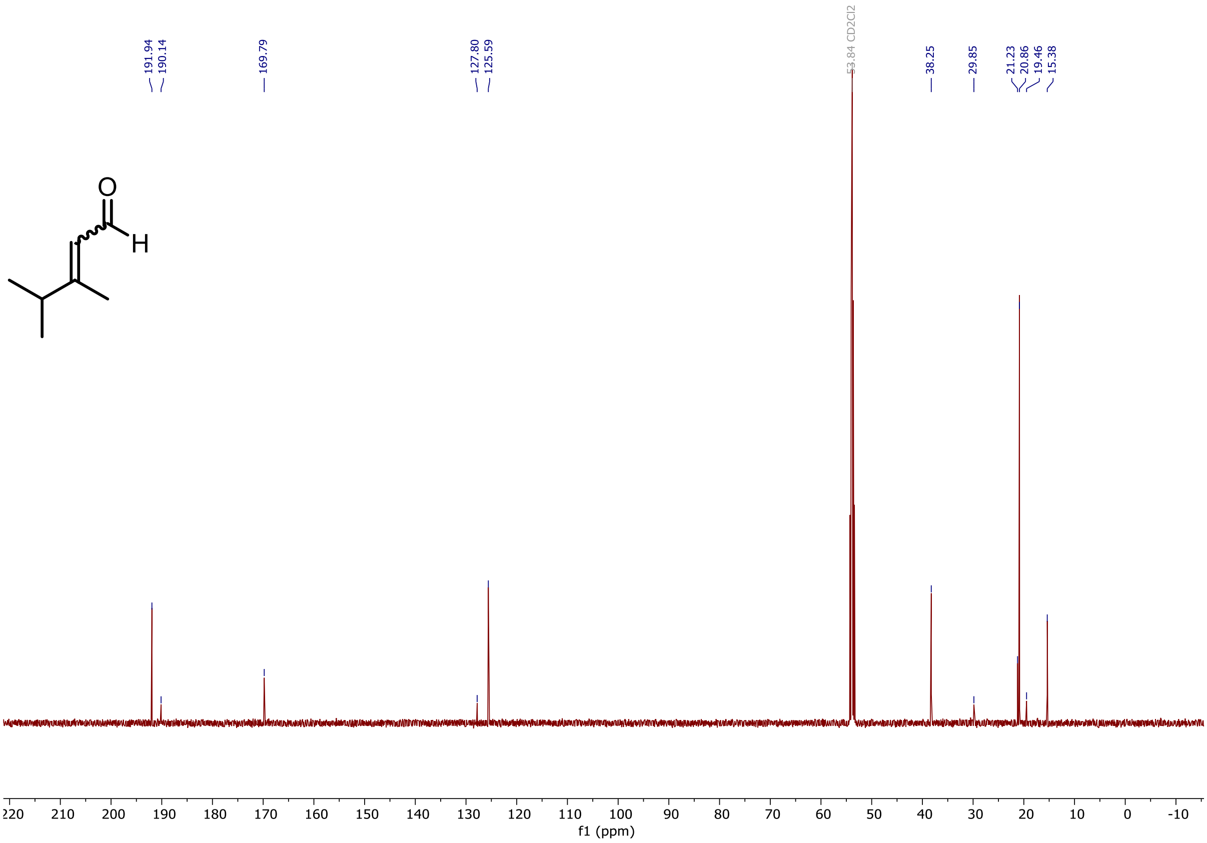

^{1}H NMR spectrum of 1r

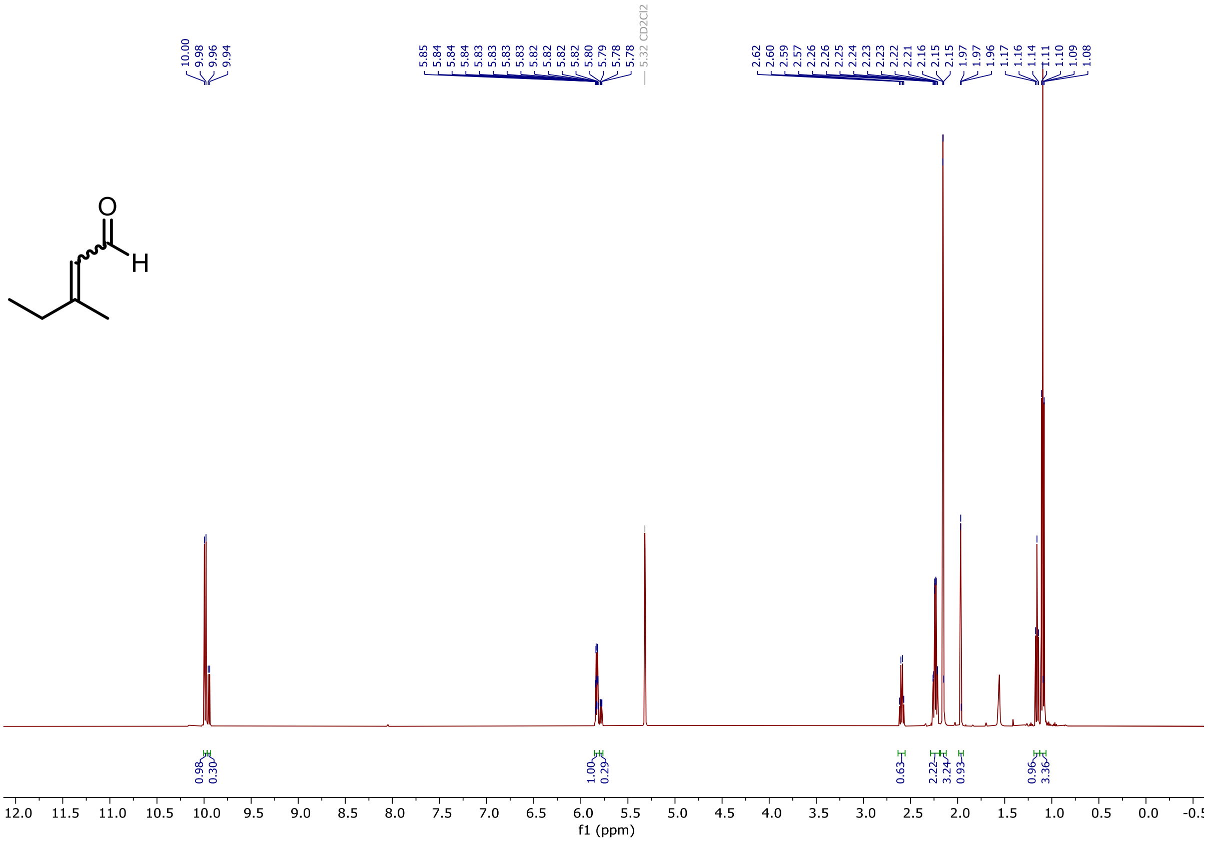

^{13}C NMR spectrum of 1r

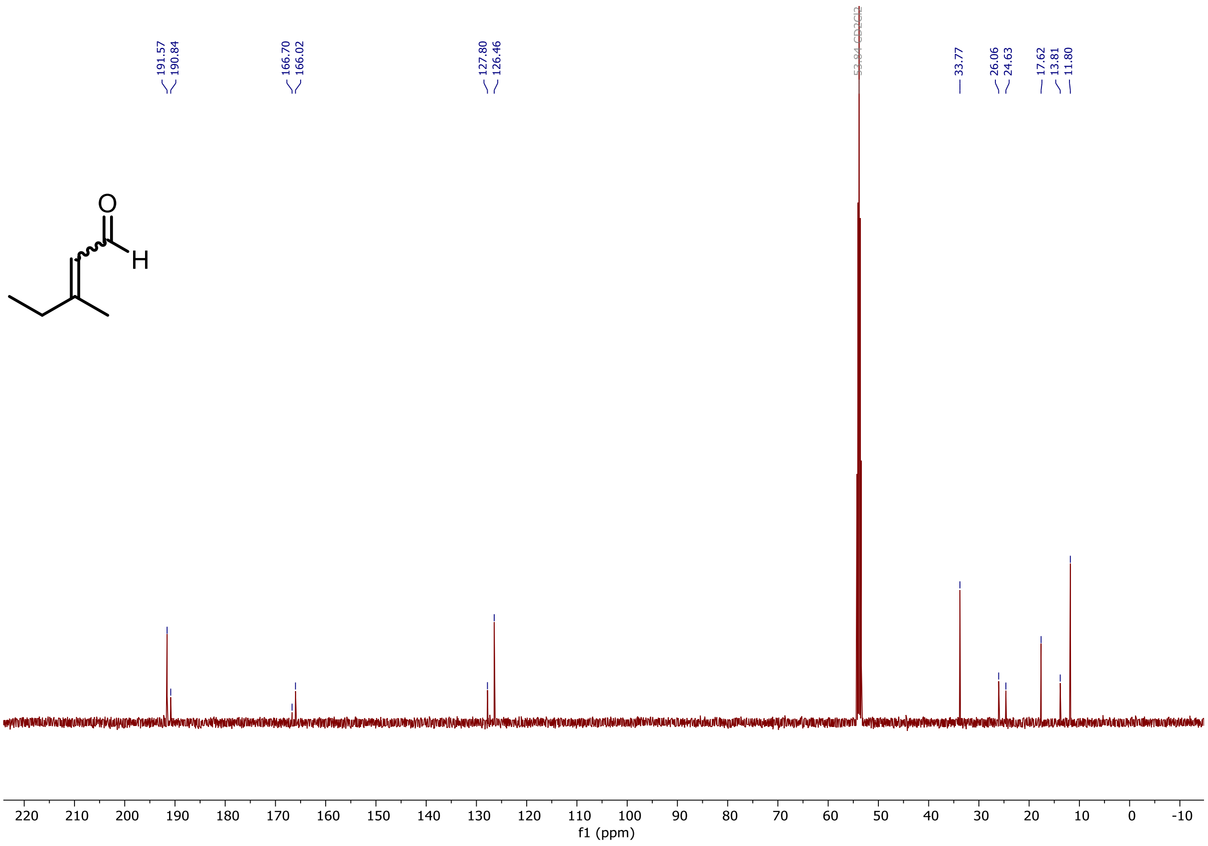

^{1}H NMR spectrum of 1s

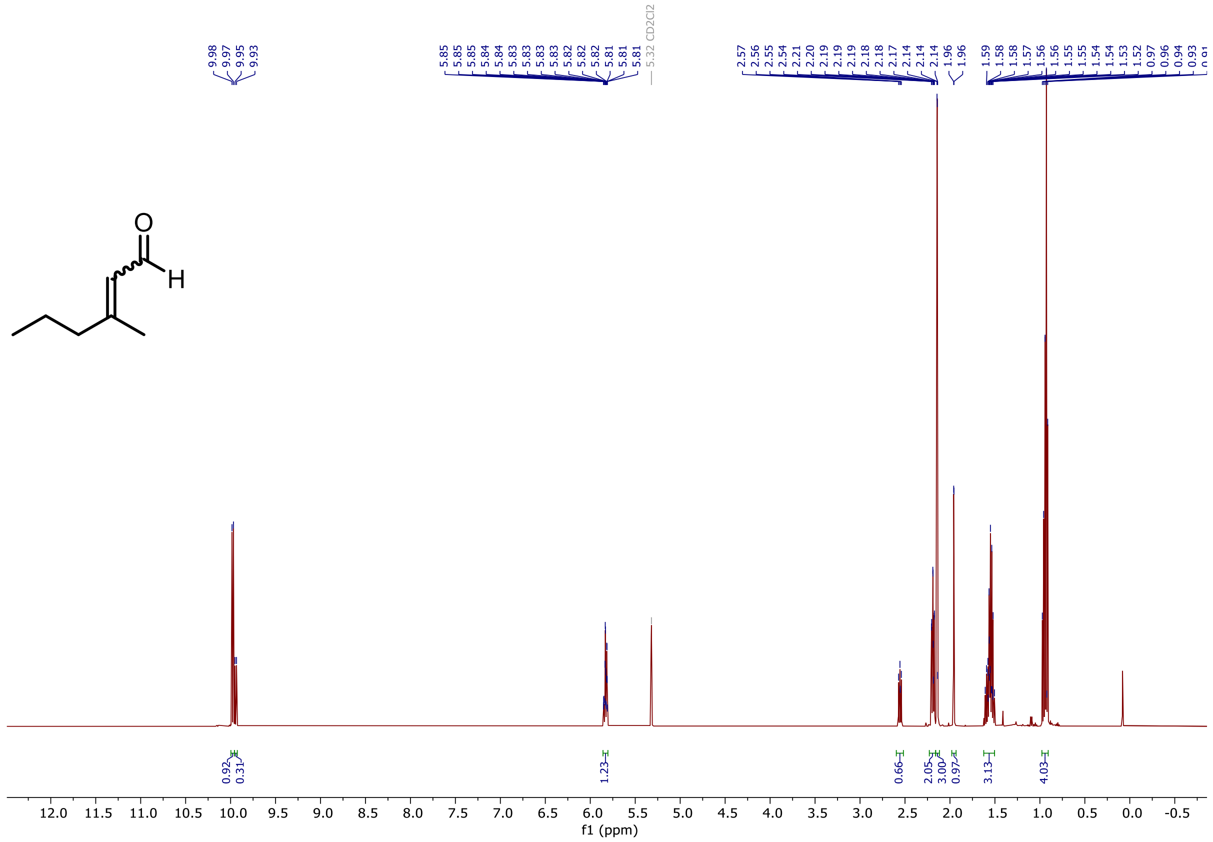

^{13}C NMR spectrum of 1s

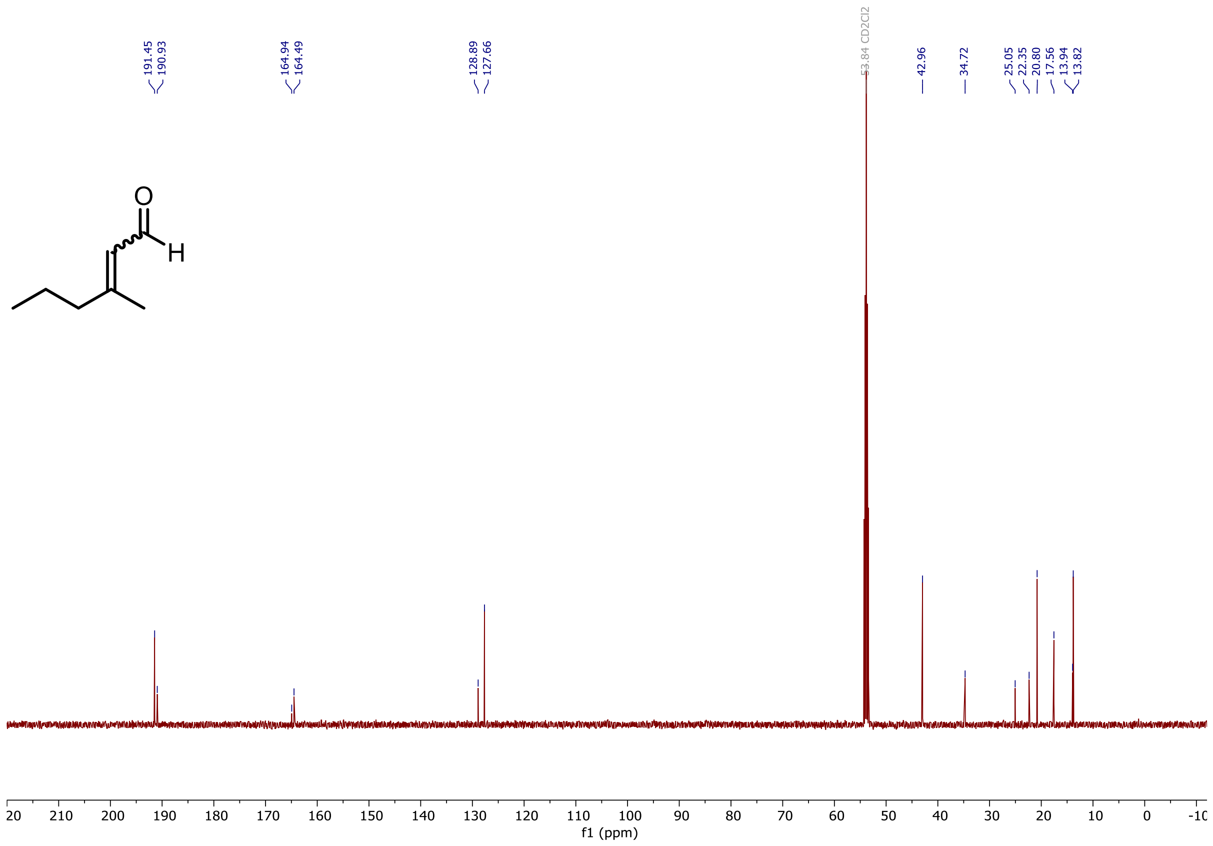

^{1}H NMR spectrum of 1t

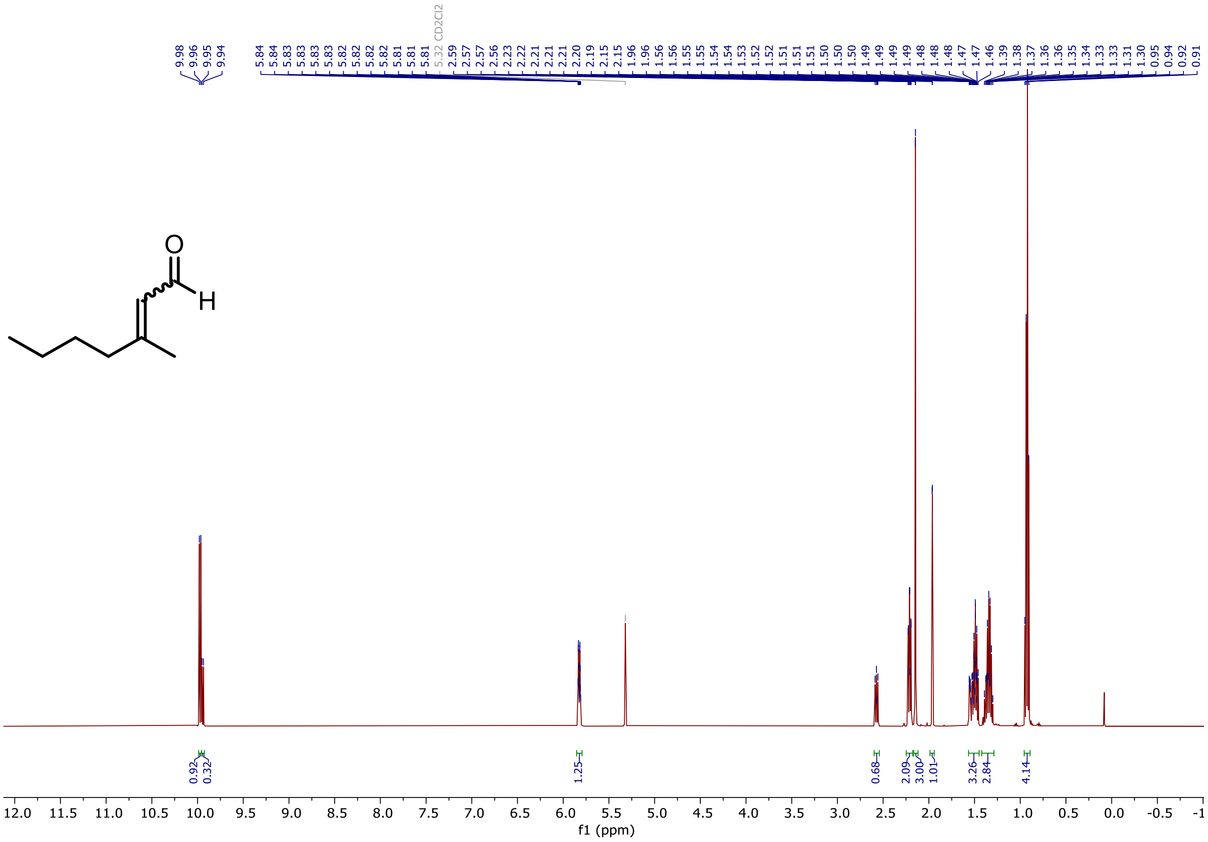

^{13}C NMR spectrum of 1t

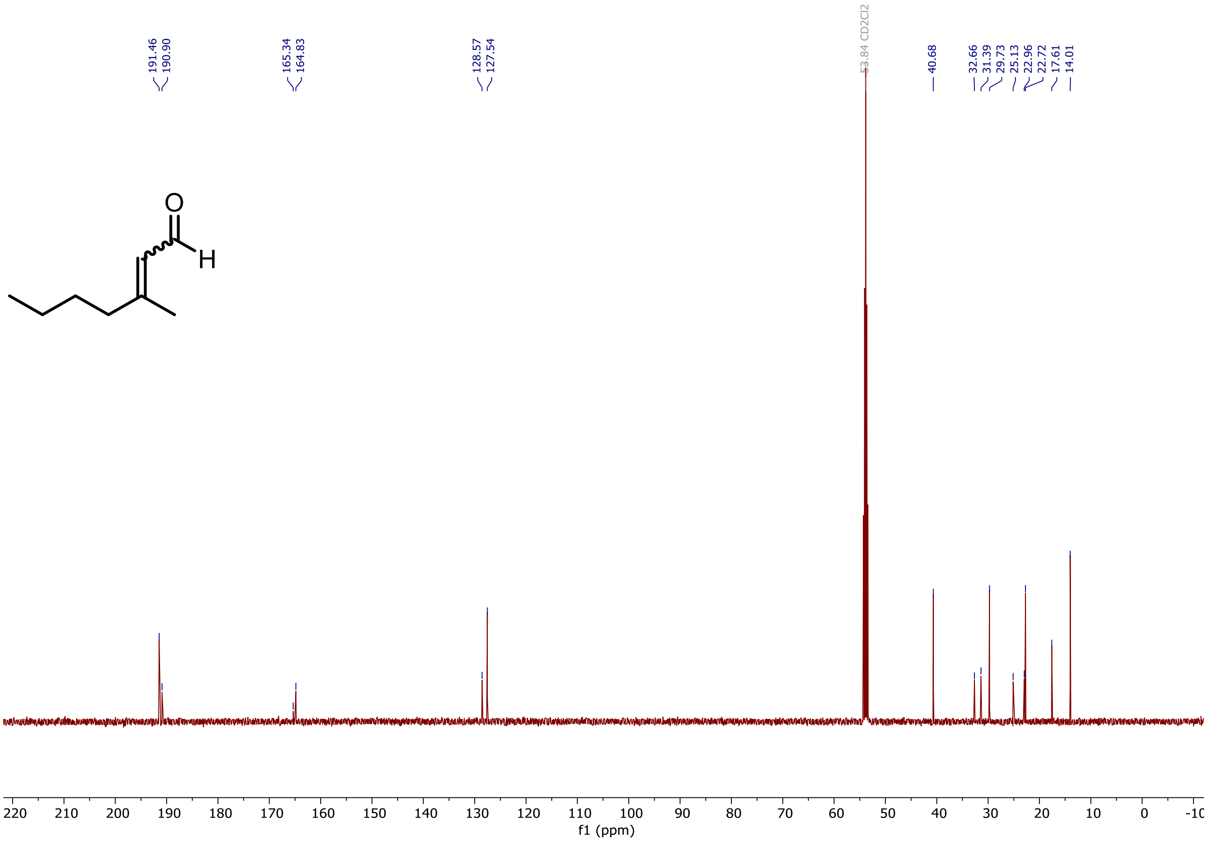

^{1}H NMR spectrum of 3a

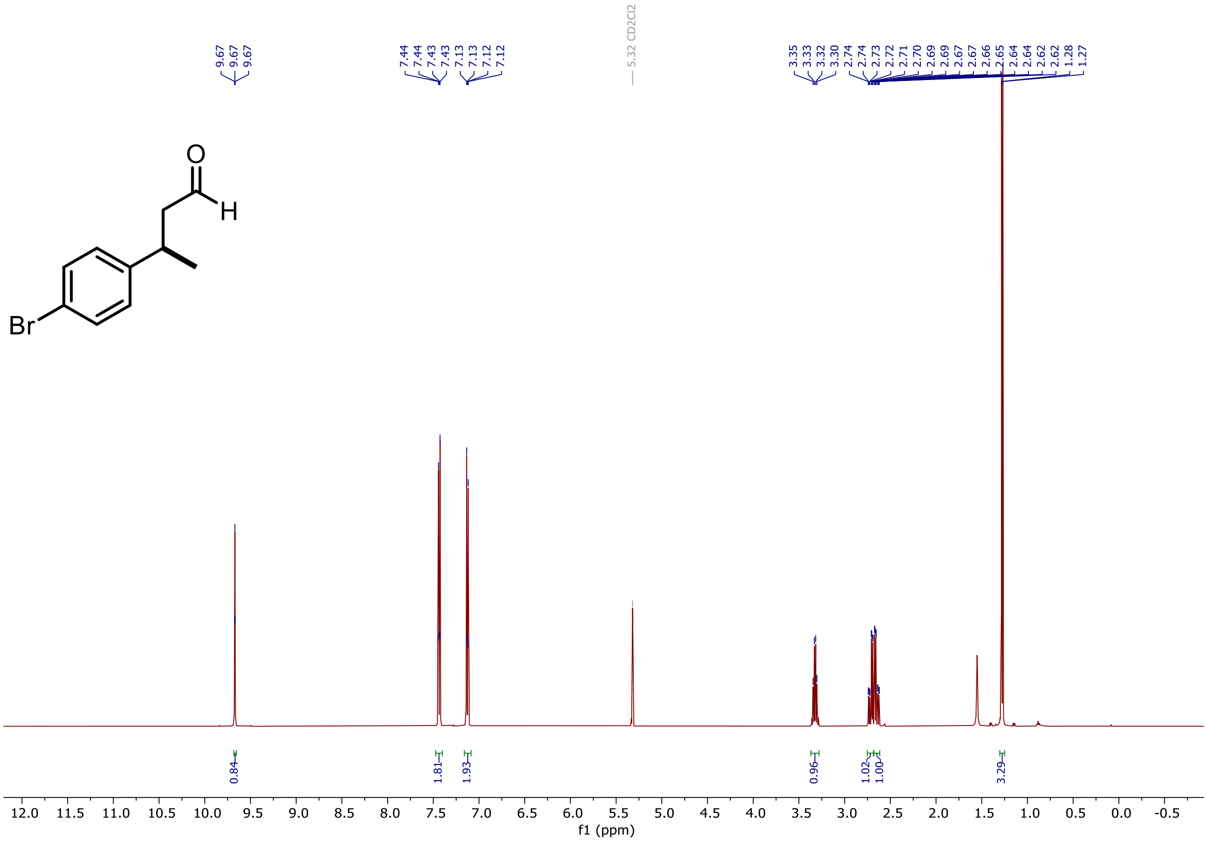

^{13}C NMR spectrum of 3a

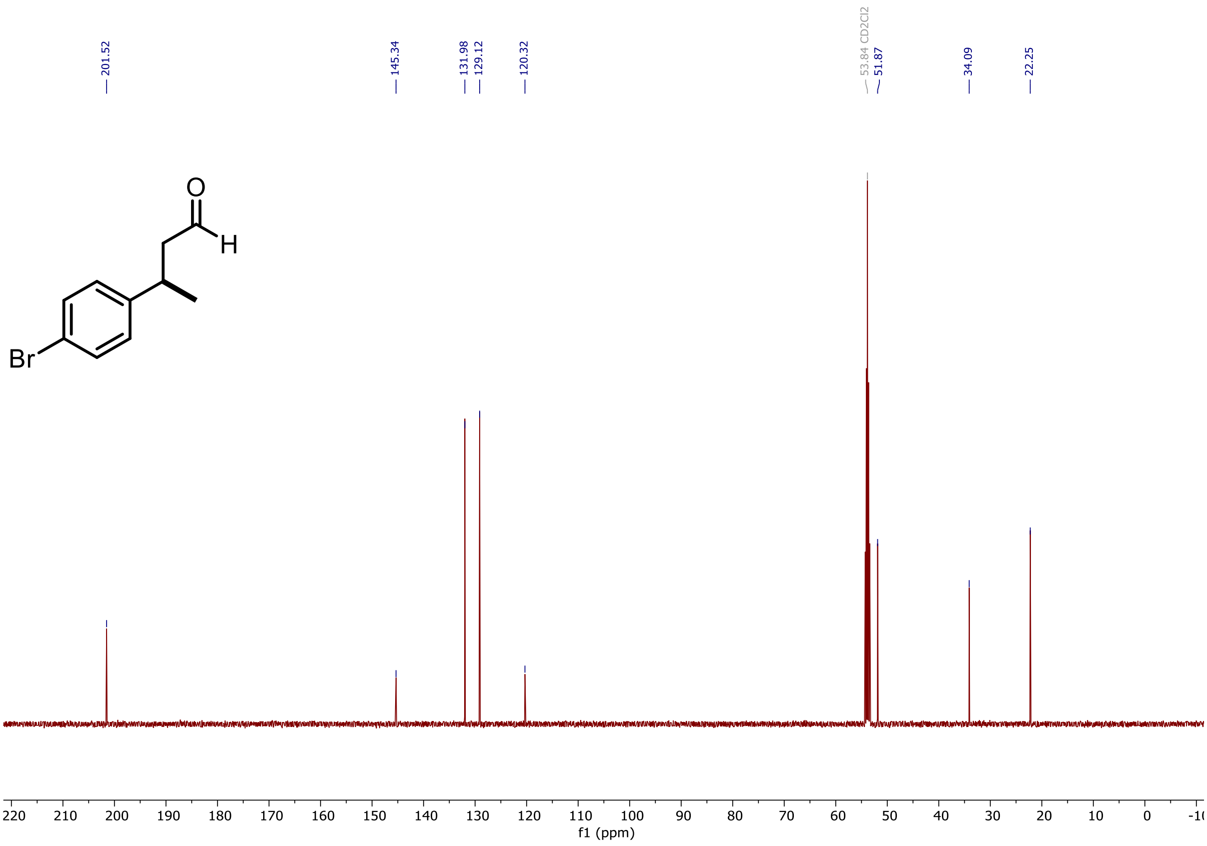

^{1}H NMR spectrum of 3b

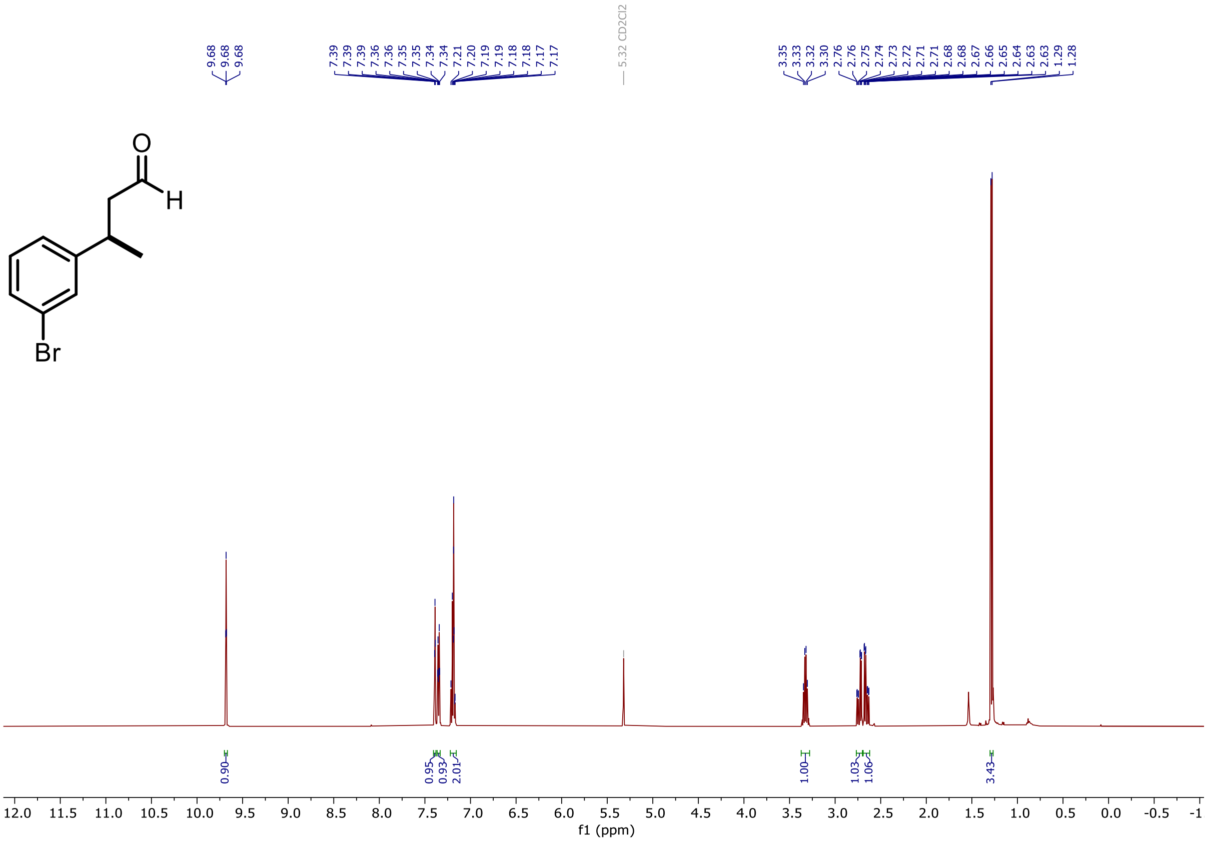

^{13}C NMR spectrum of 3b

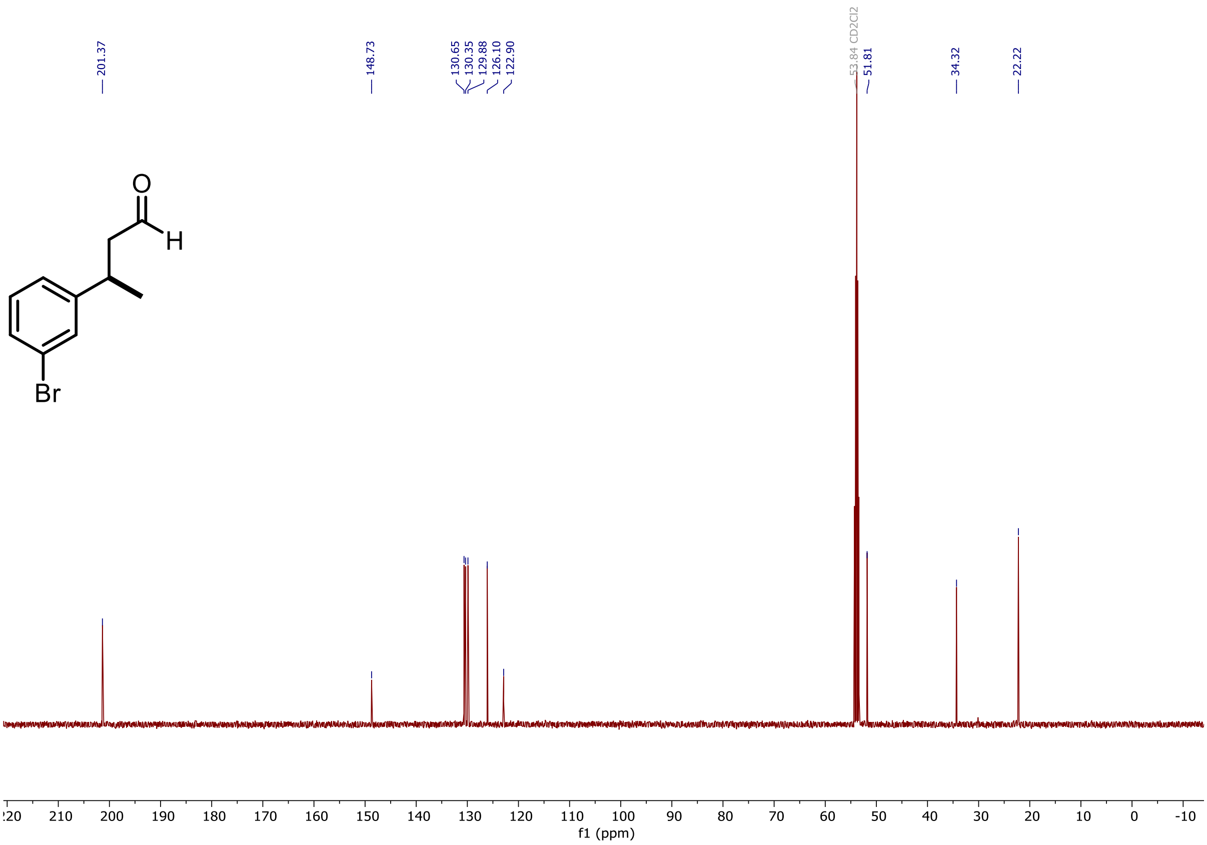

^{1}H NMR spectrum of 3c

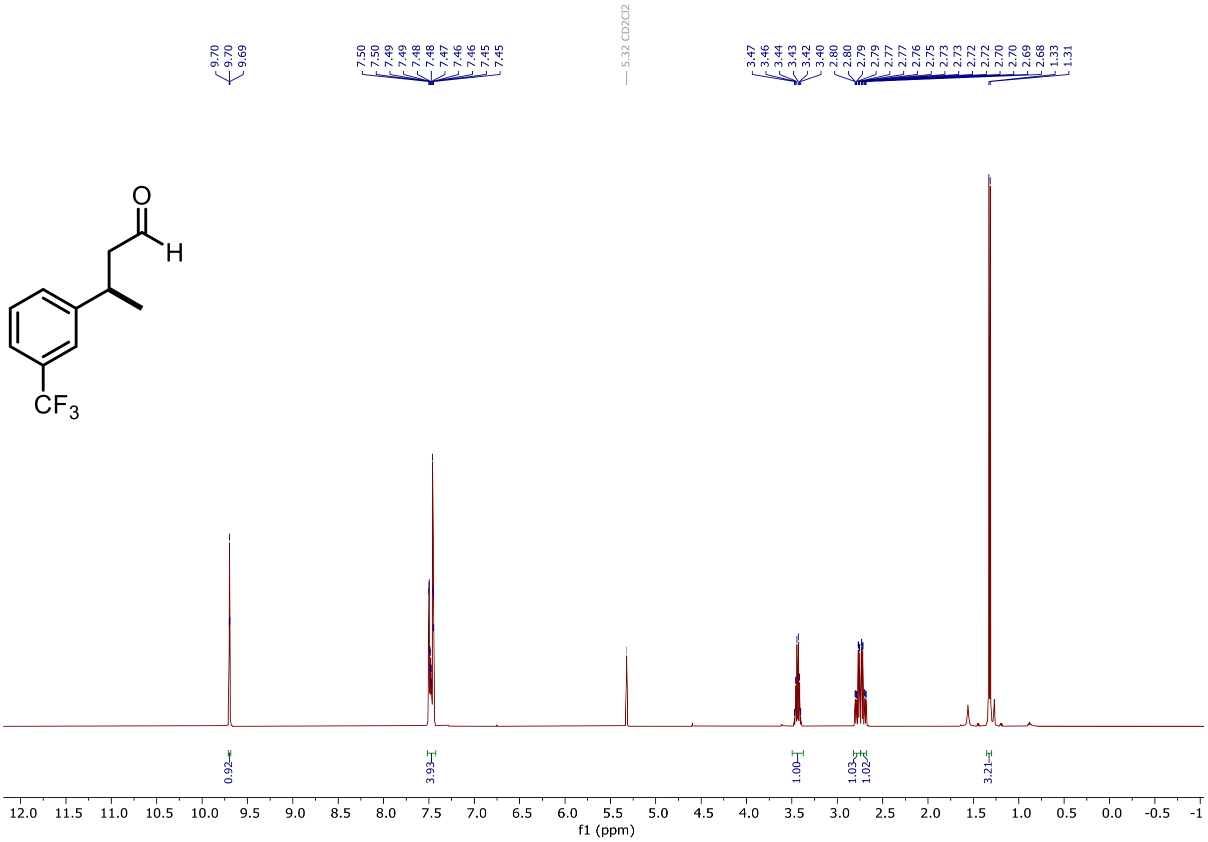

^{19}F{^{1}H} NMR spectrum of 3c

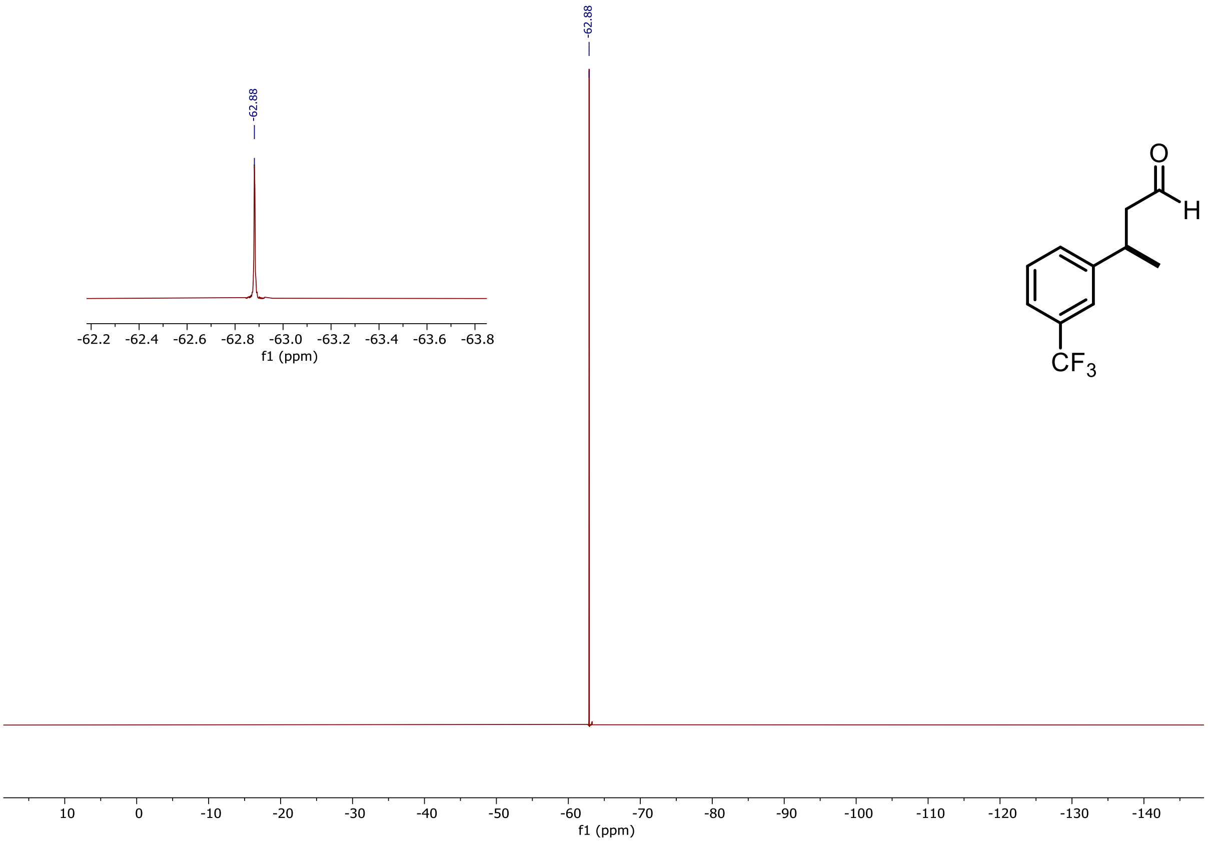

^{13}C NMR spectrum of 3c

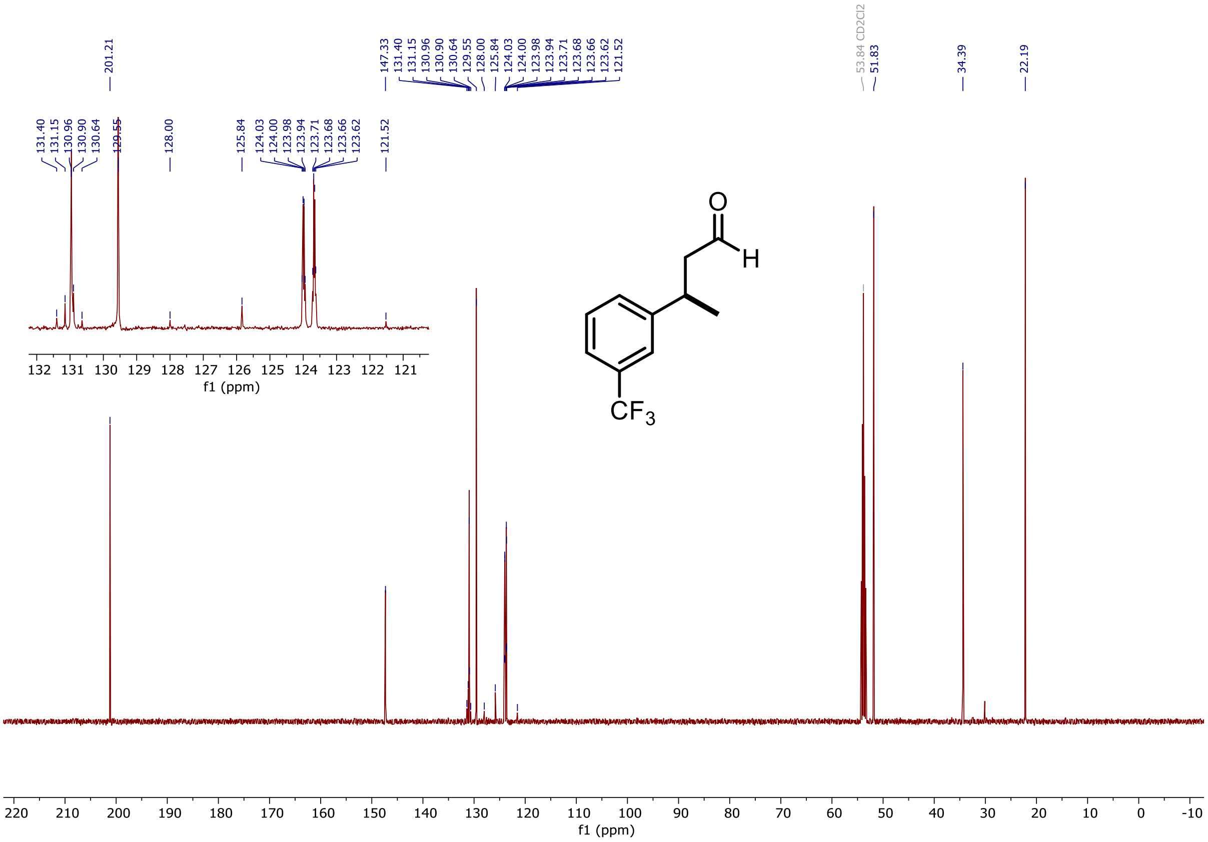

^{1}H NMR spectrum of 3d

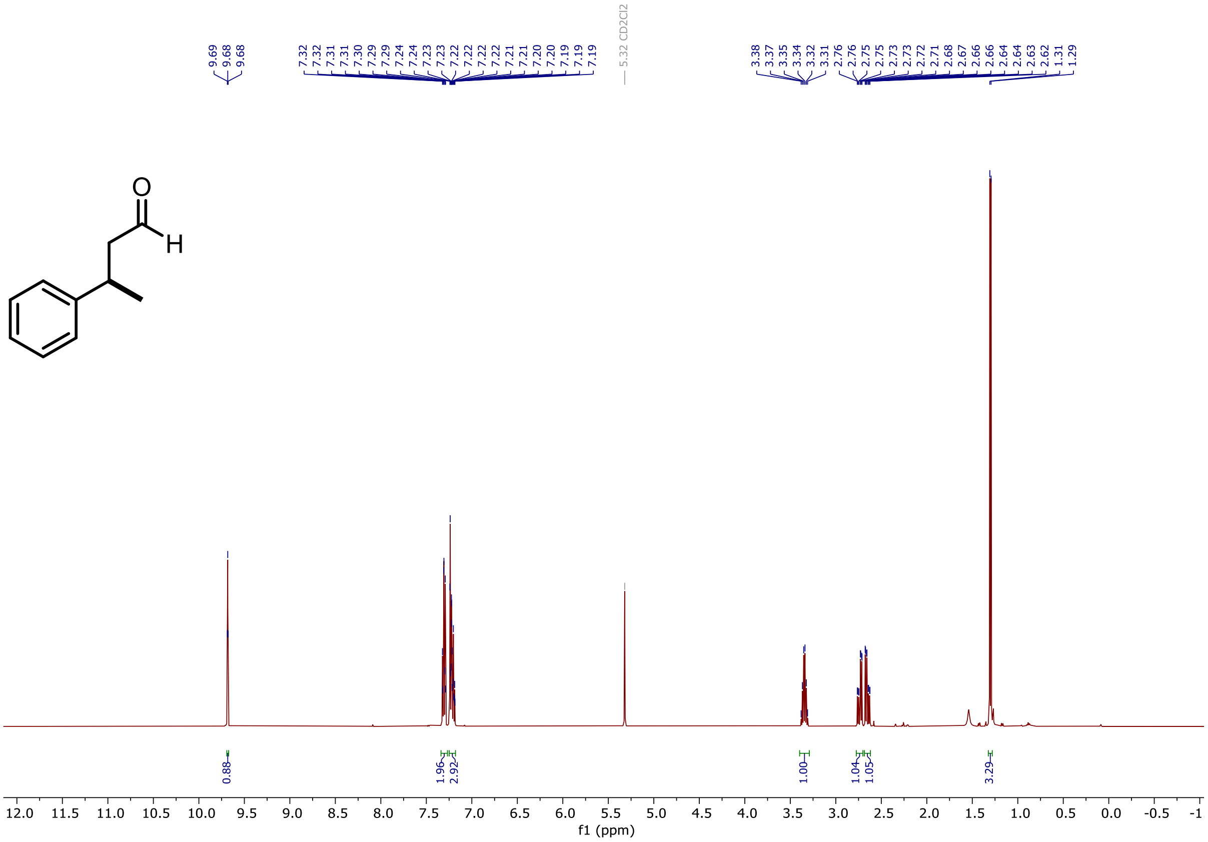

^{13}C NMR spectrum of 3d

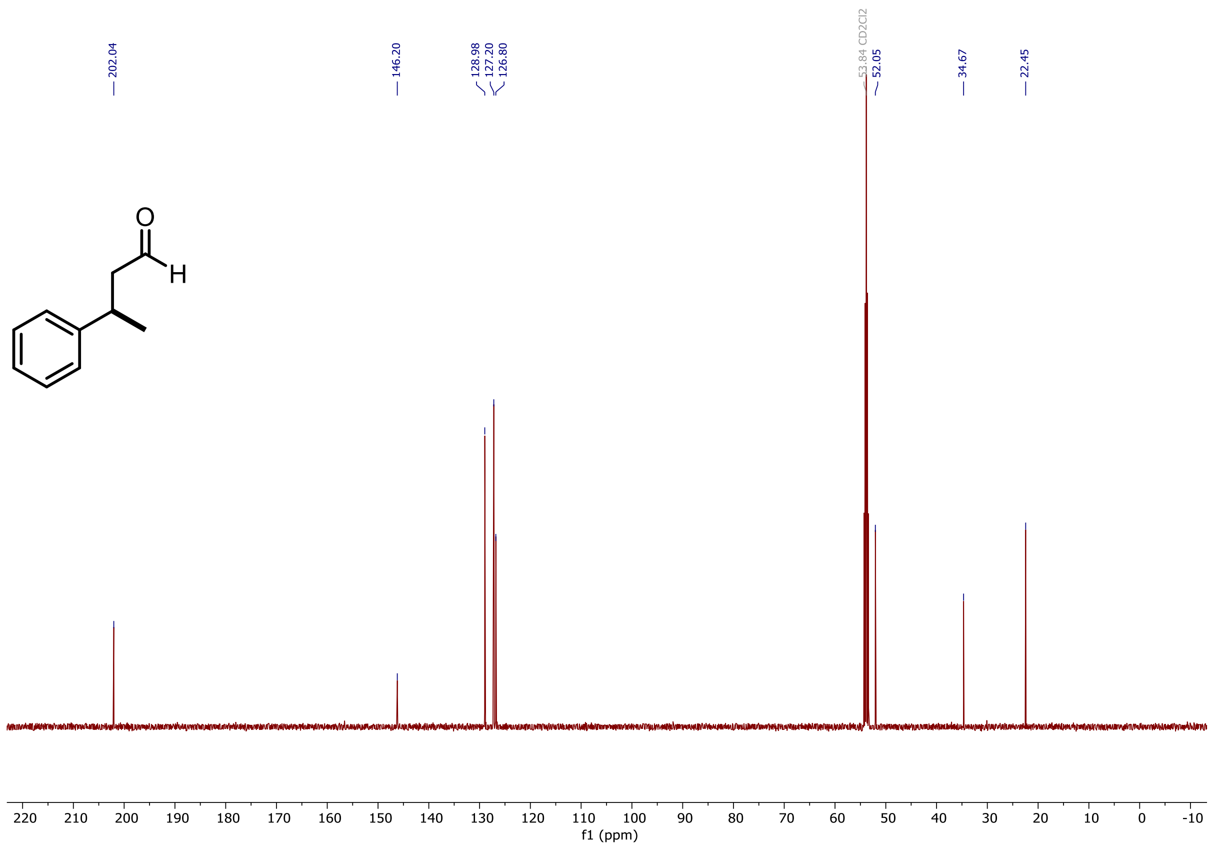

^{1}H NMR spectrum of 3e

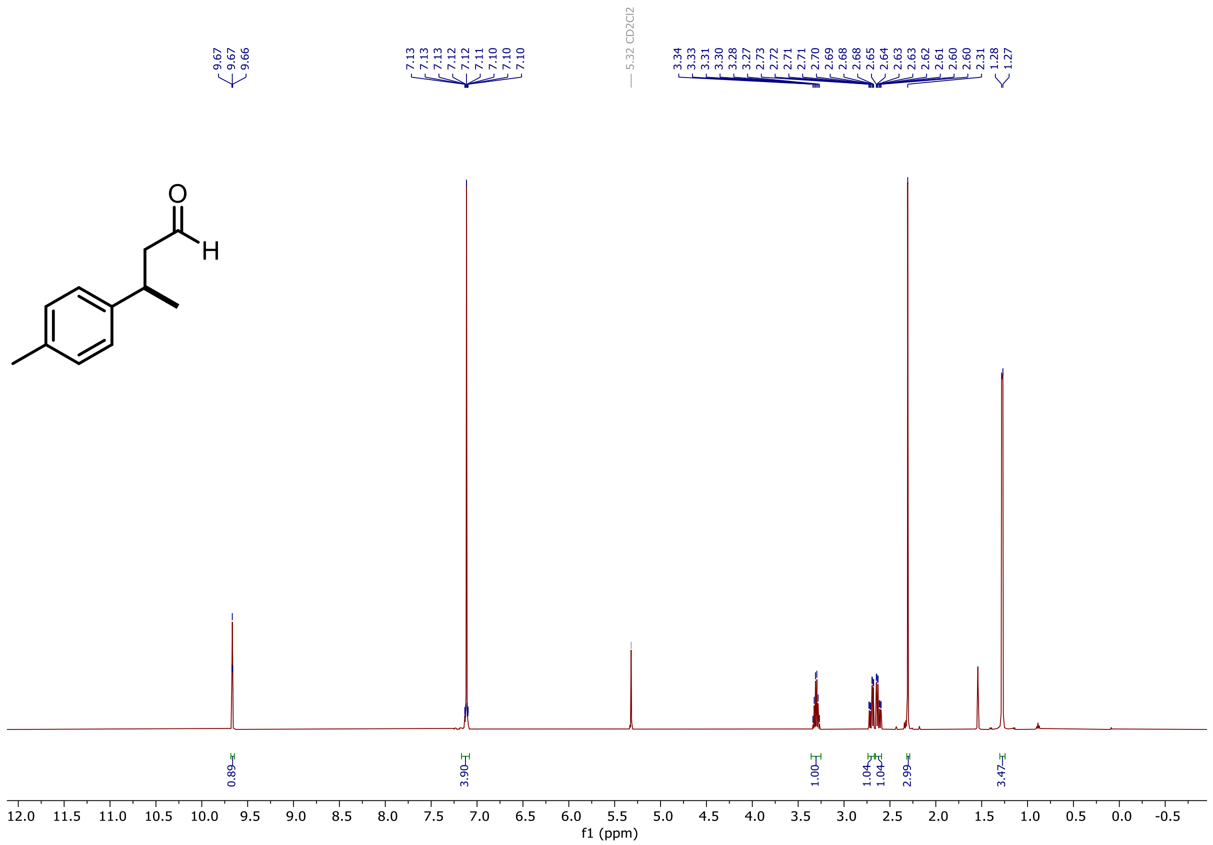

^{13}C NMR spectrum of 3e

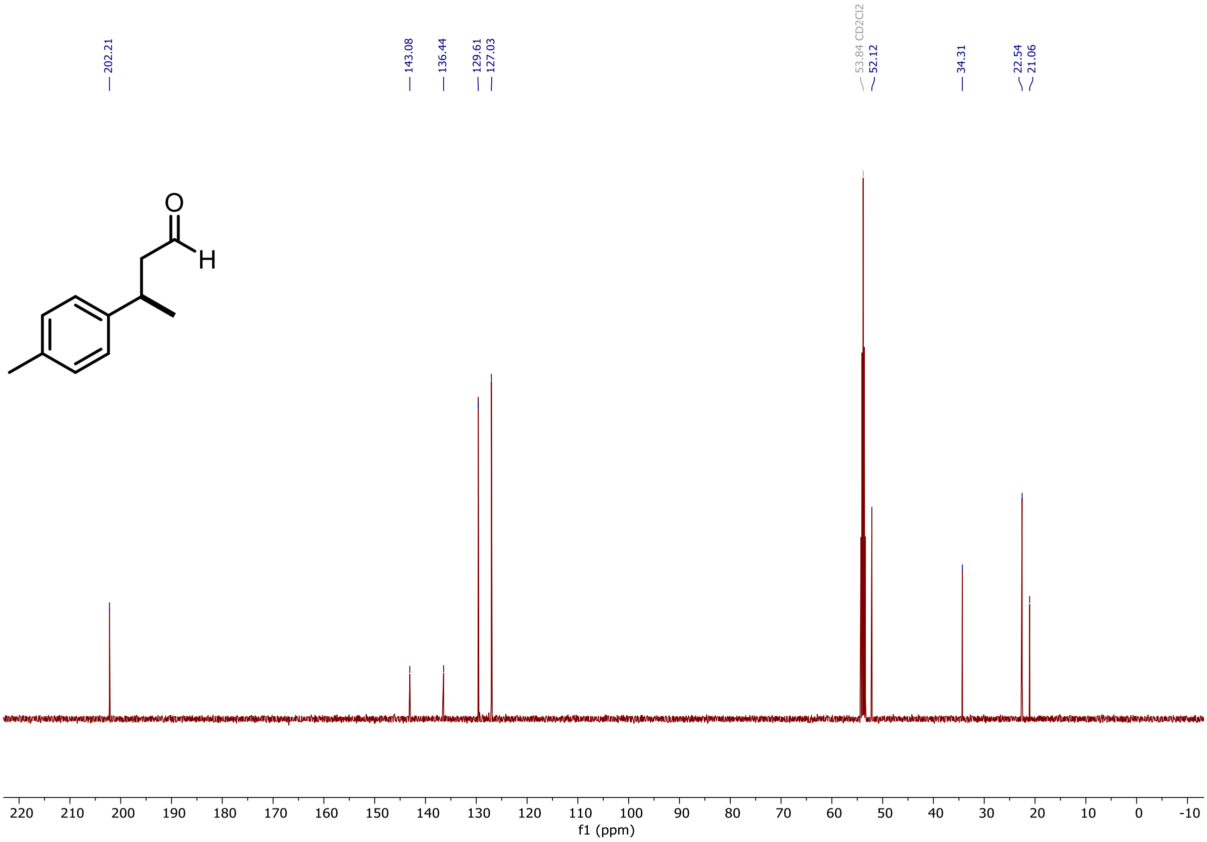

^{1}H NMR spectrum of 3f

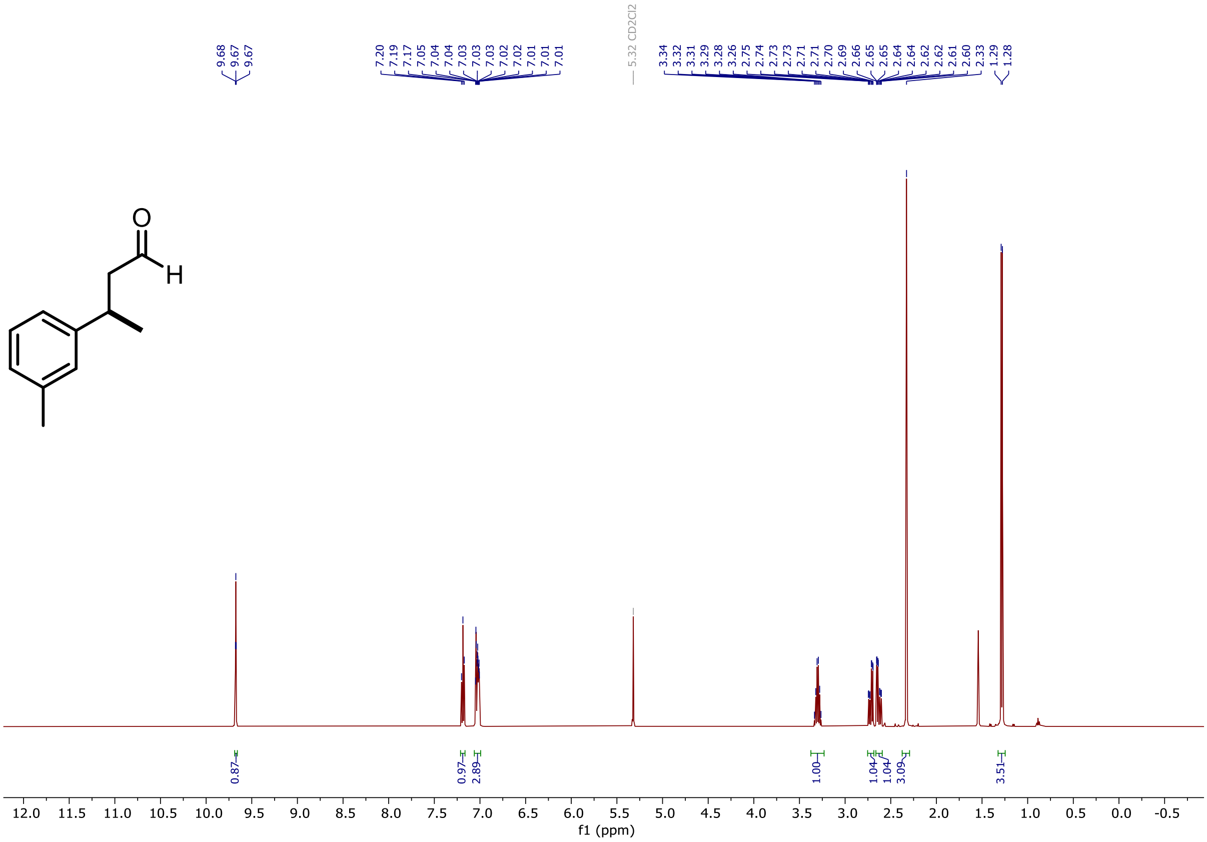

^{13}C NMR spectrum of 3f

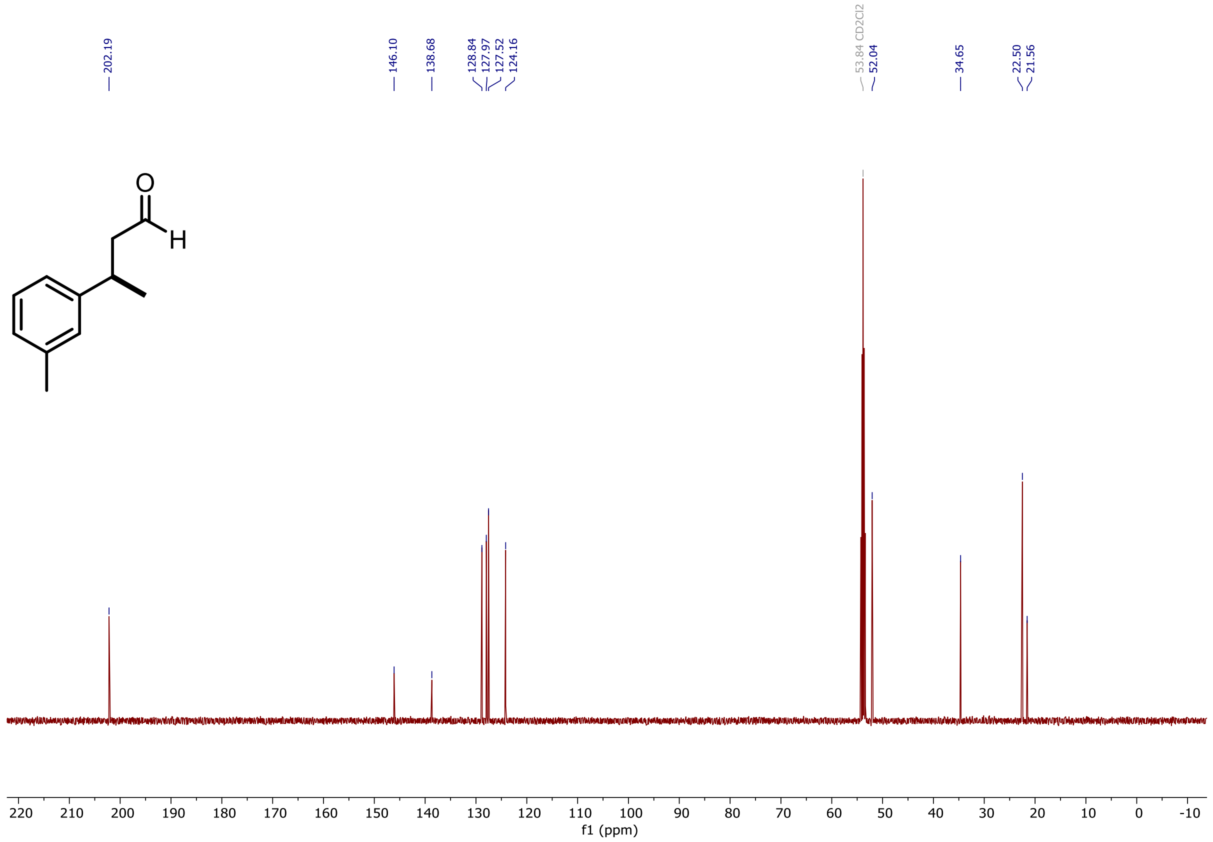

^{1}H NMR spectrum of 3g

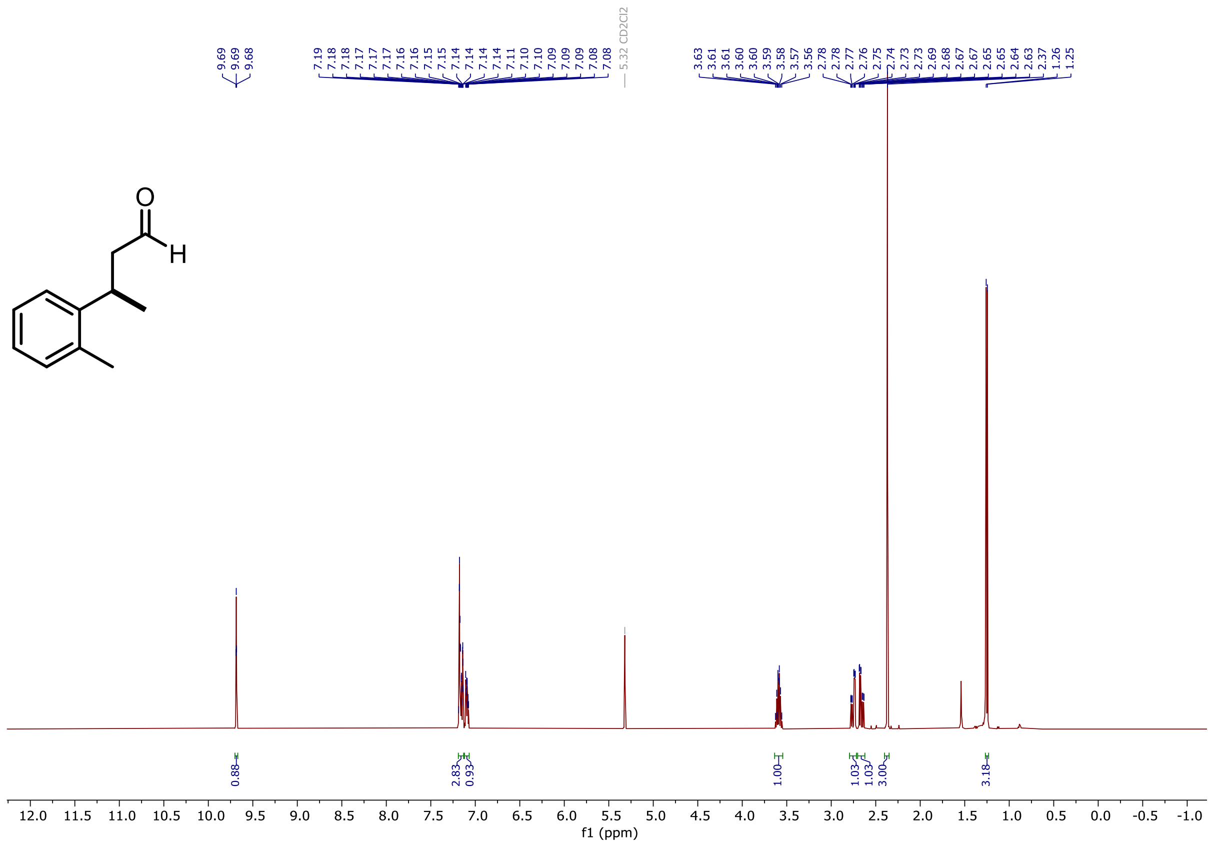

^{13}C NMR spectrum of 3g

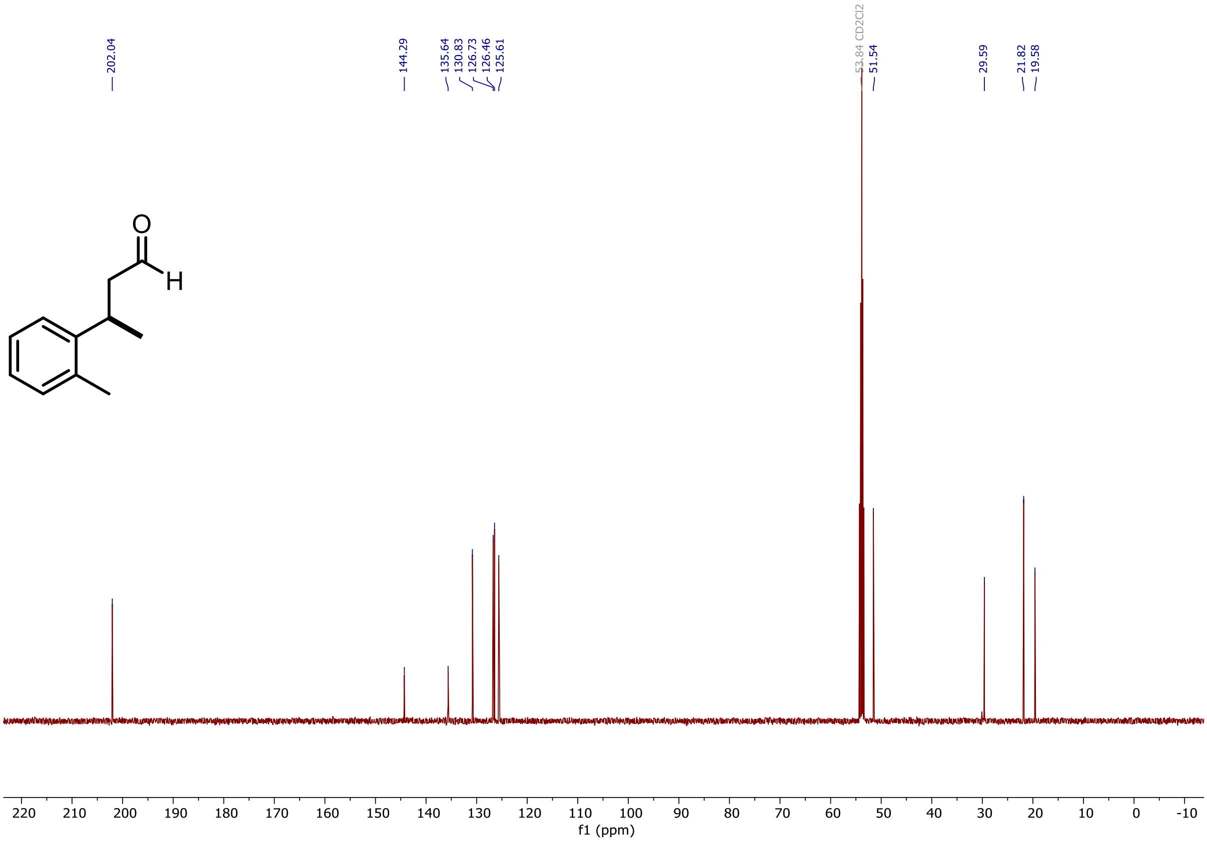

^{1}H NMR spectrum of 3h

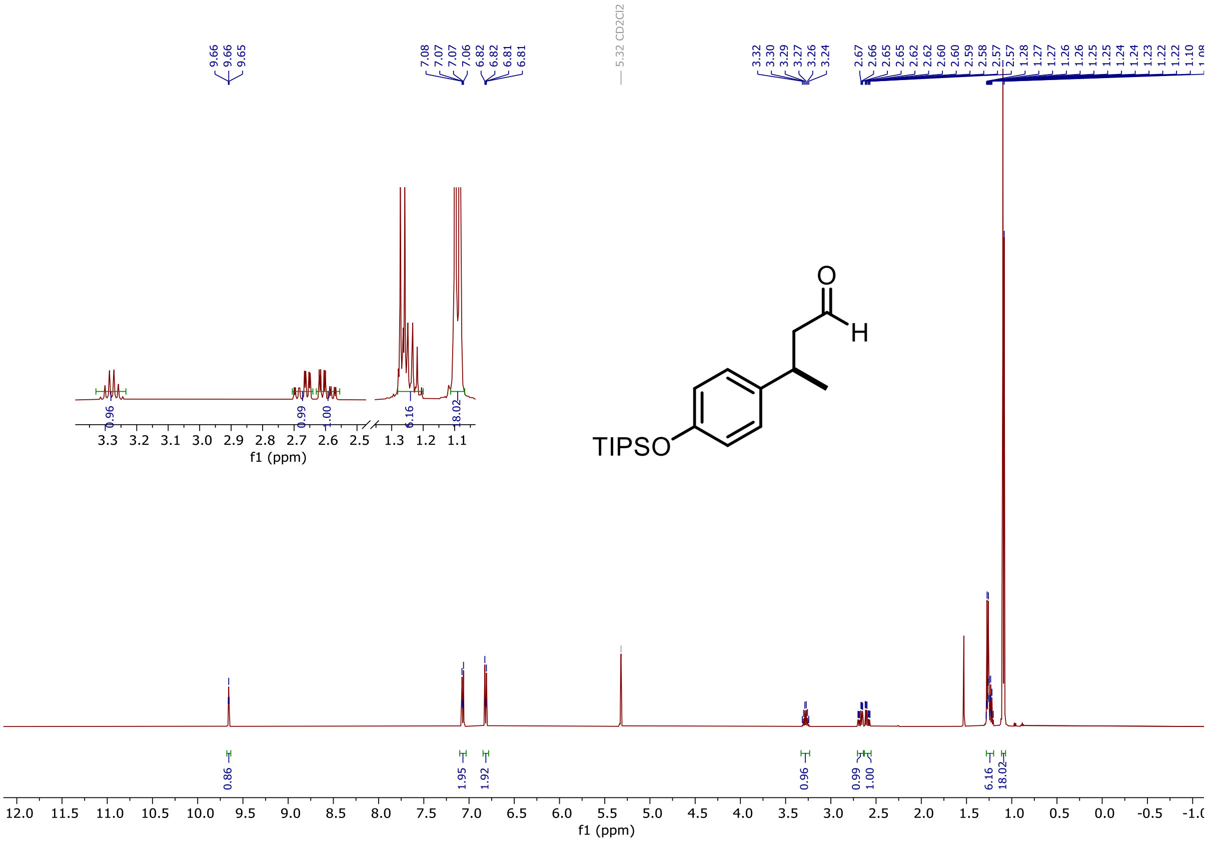

^{13}C NMR spectrum of 3h

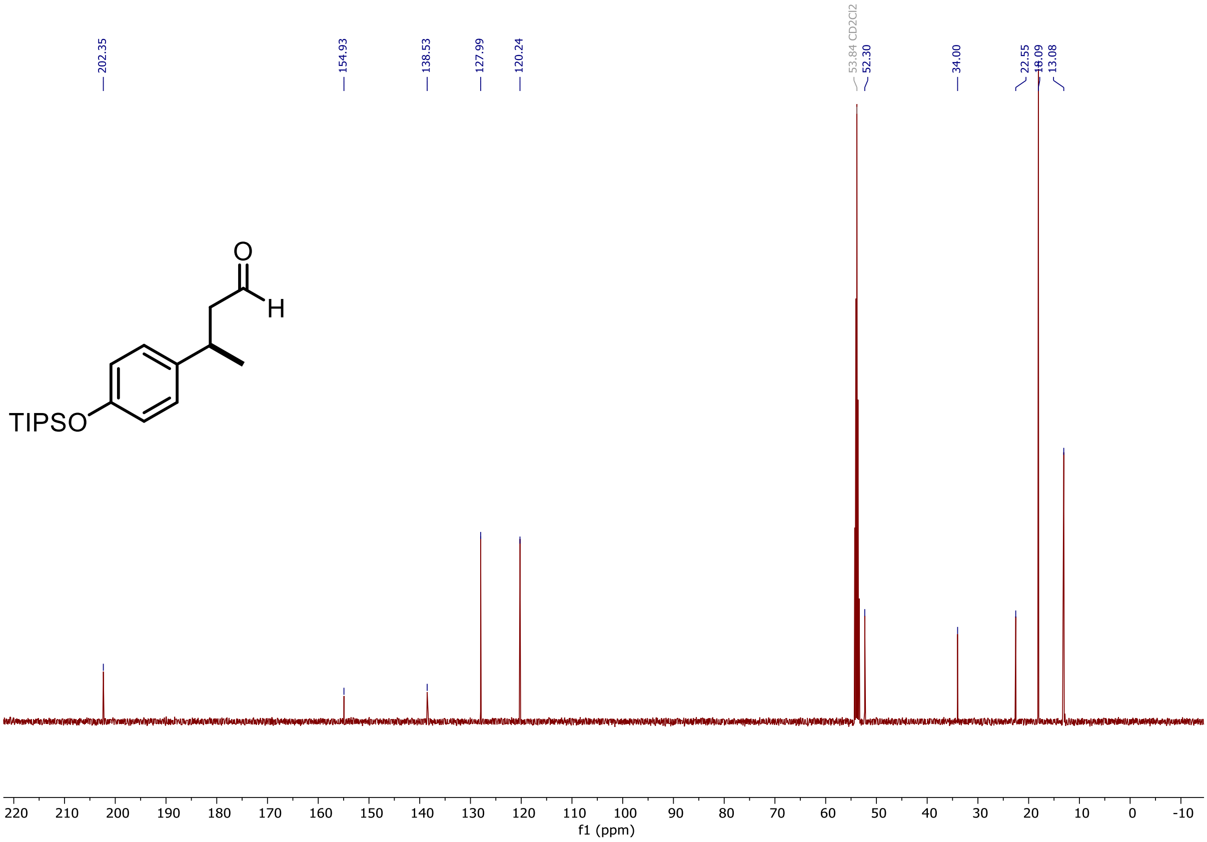

^{1}H NMR spectrum of 3i

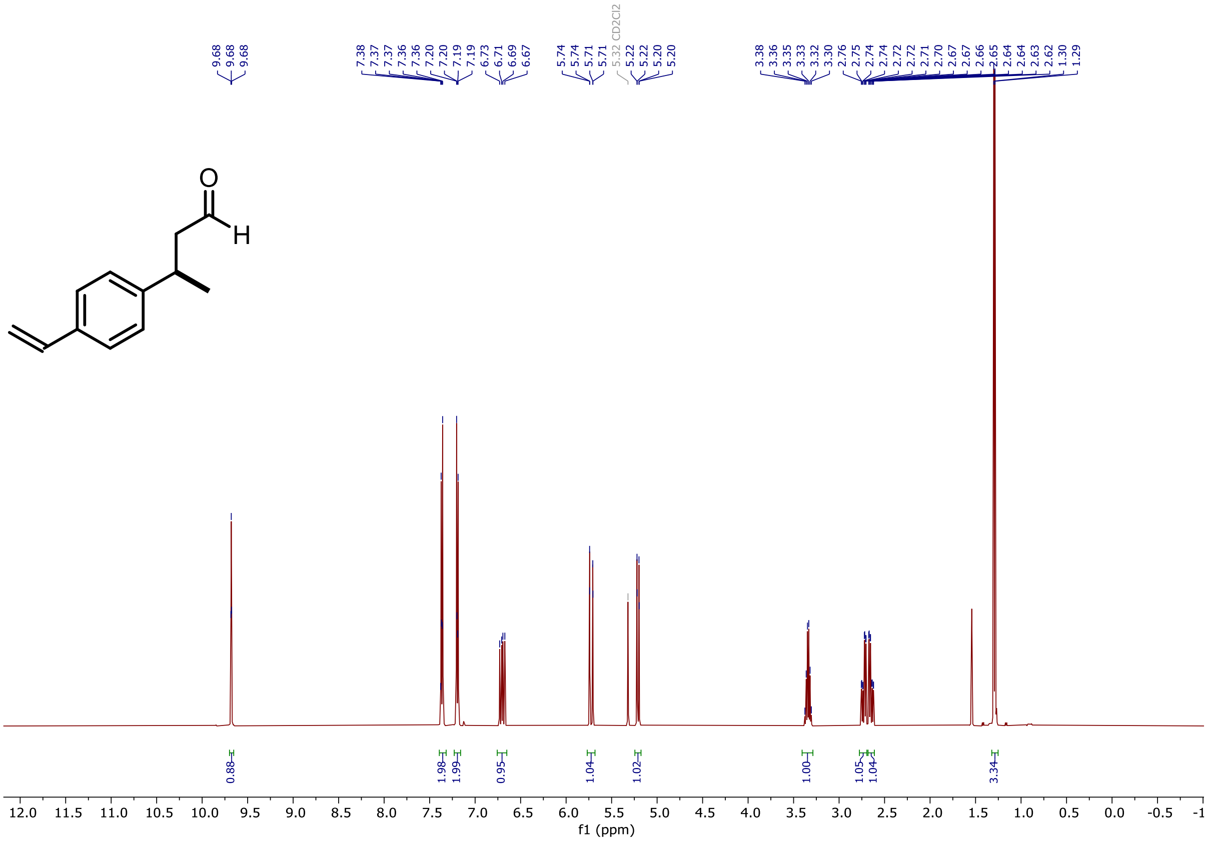

^{13}C NMR spectrum of 3i

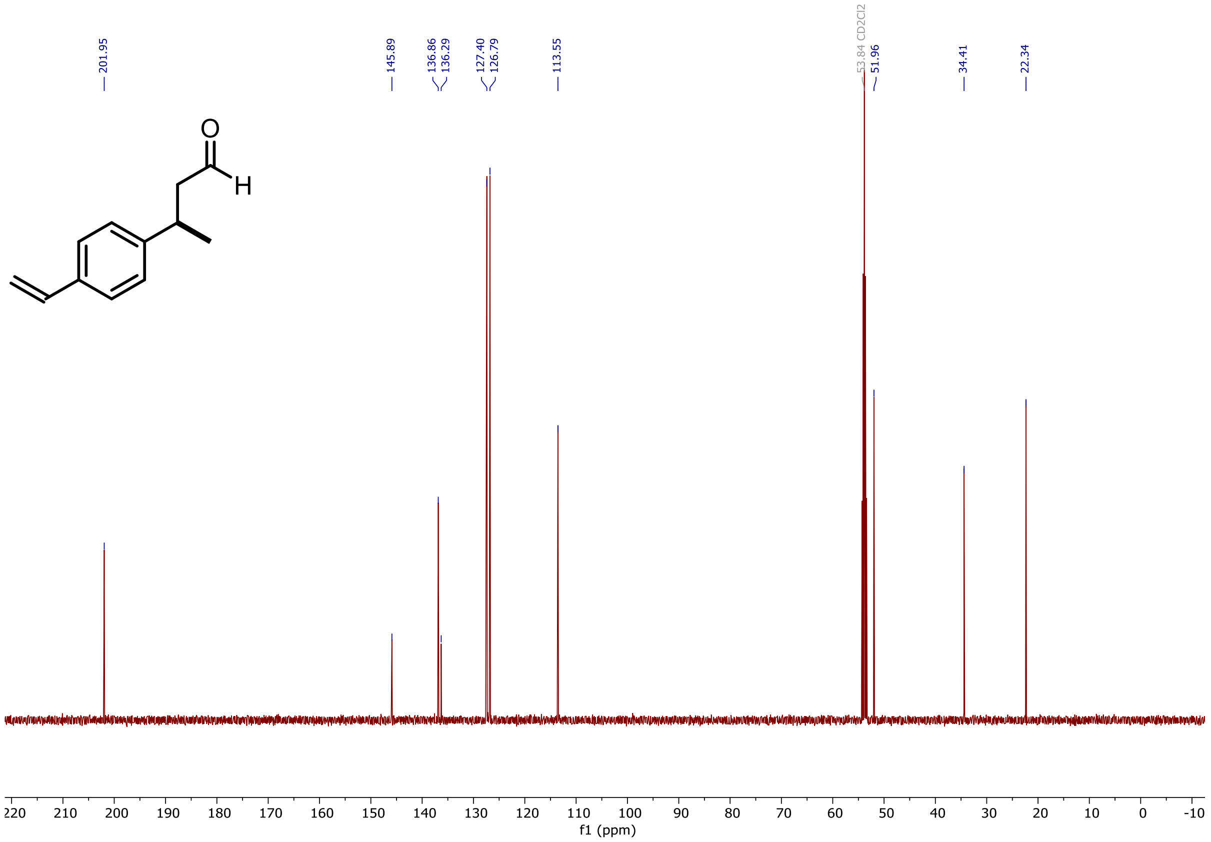

^{1}H NMR spectrum of 3j

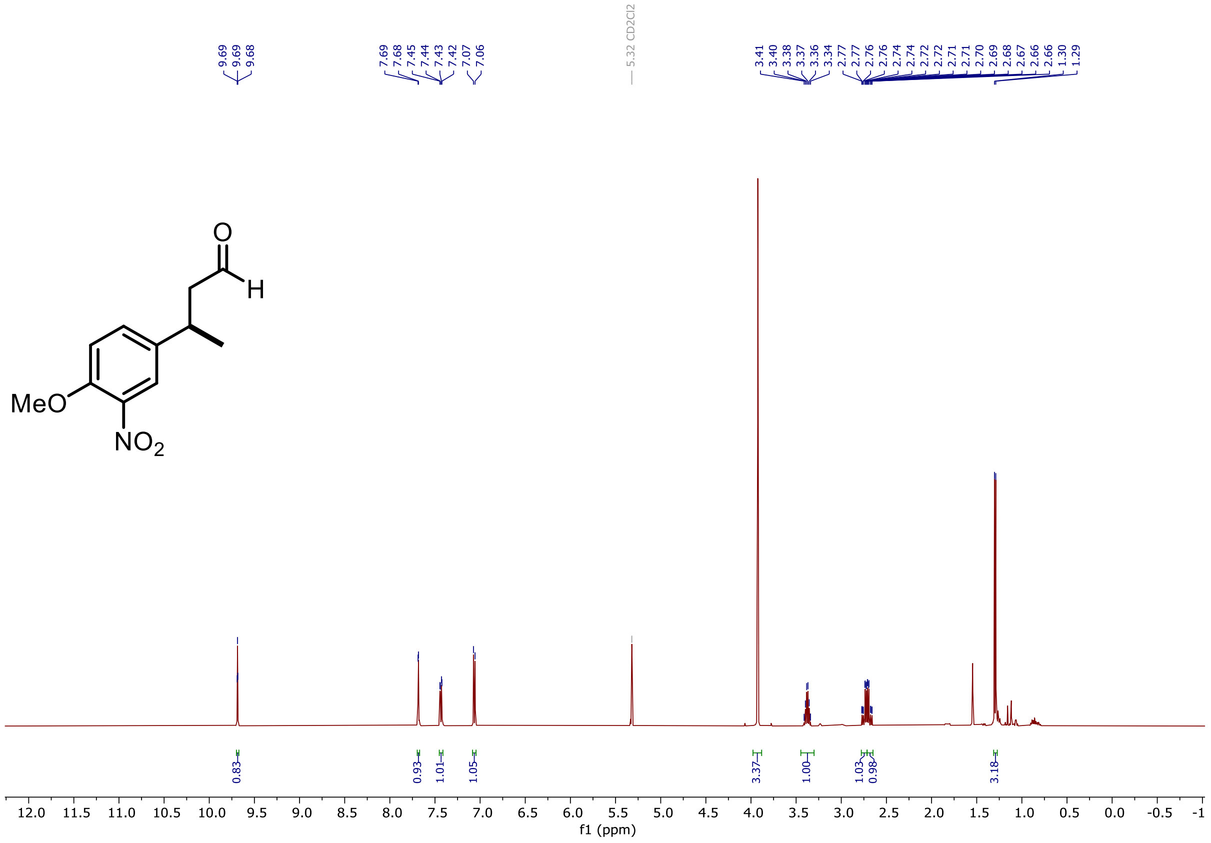

^{13}C NMR spectrum of 3j

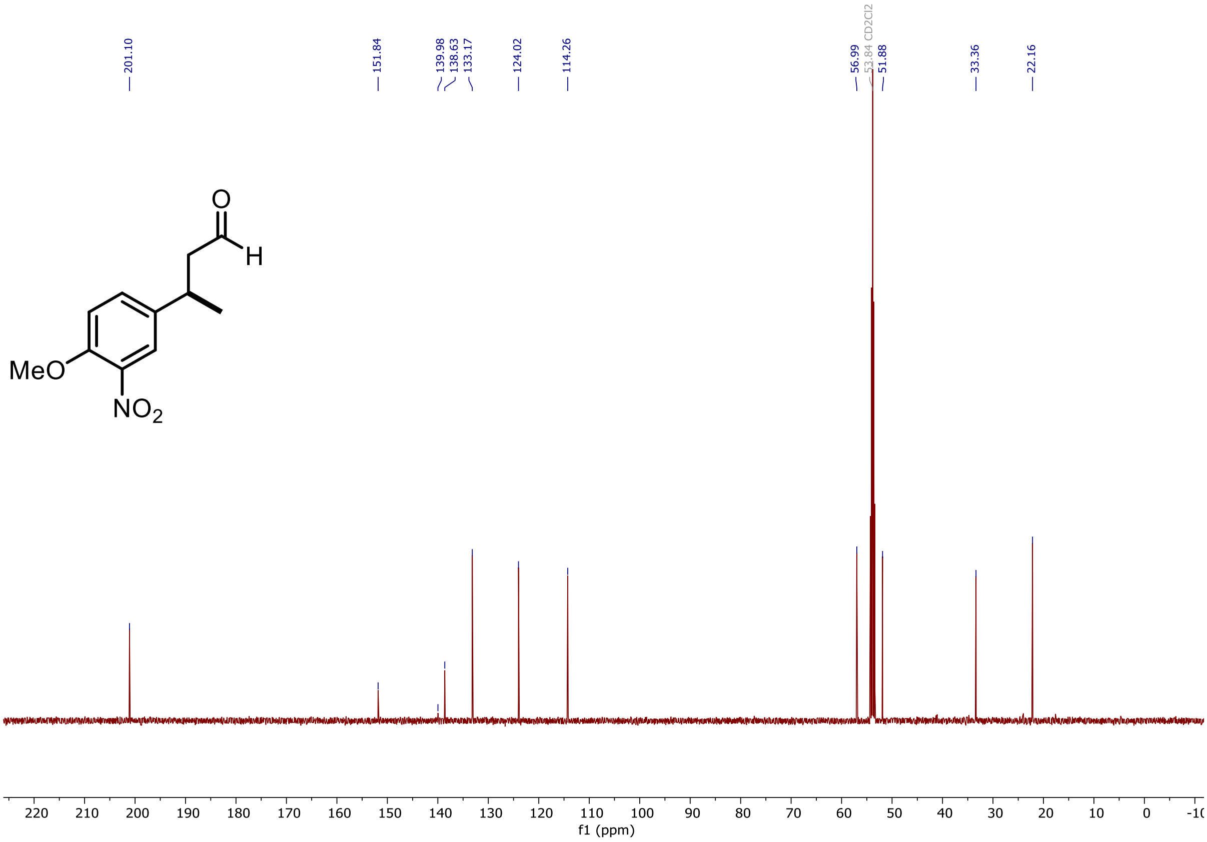

^{1}H NMR spectrum of 3k

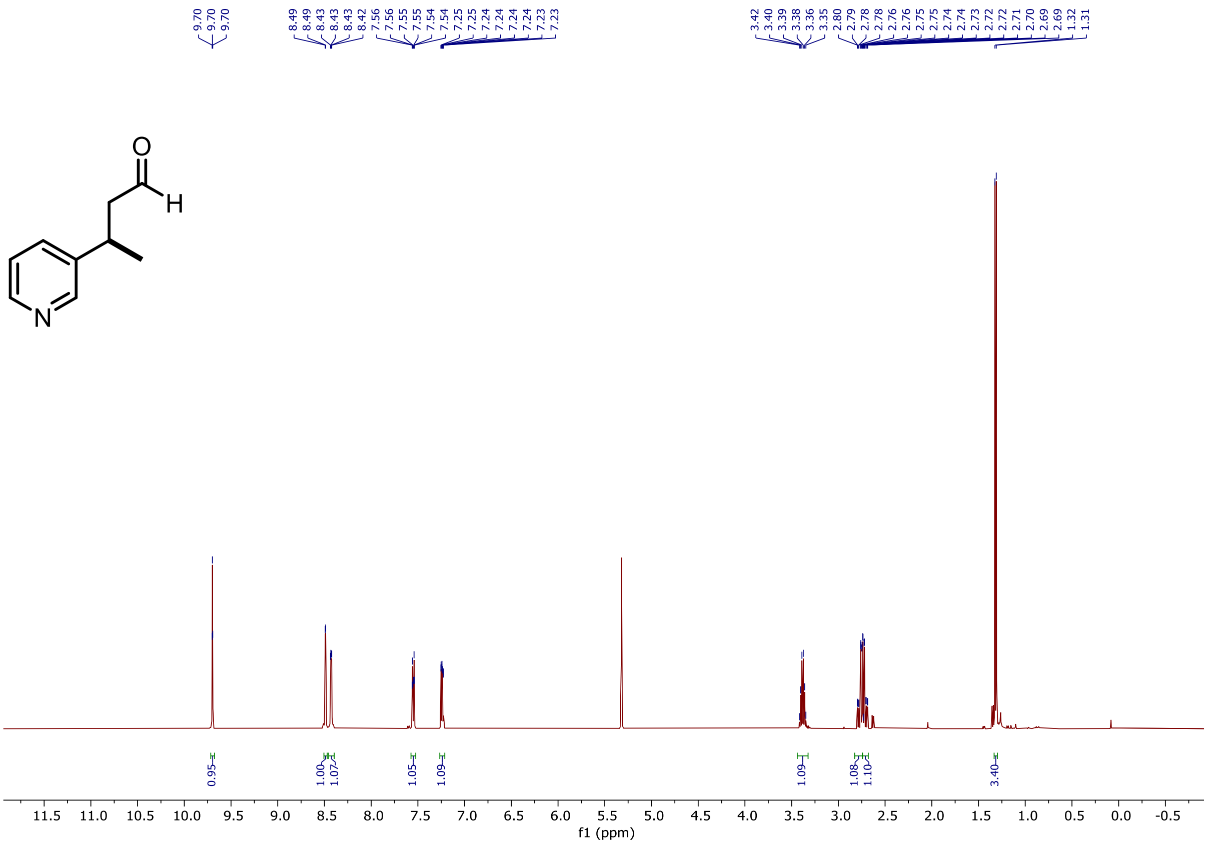

^{13}C NMR spectrum of 3k

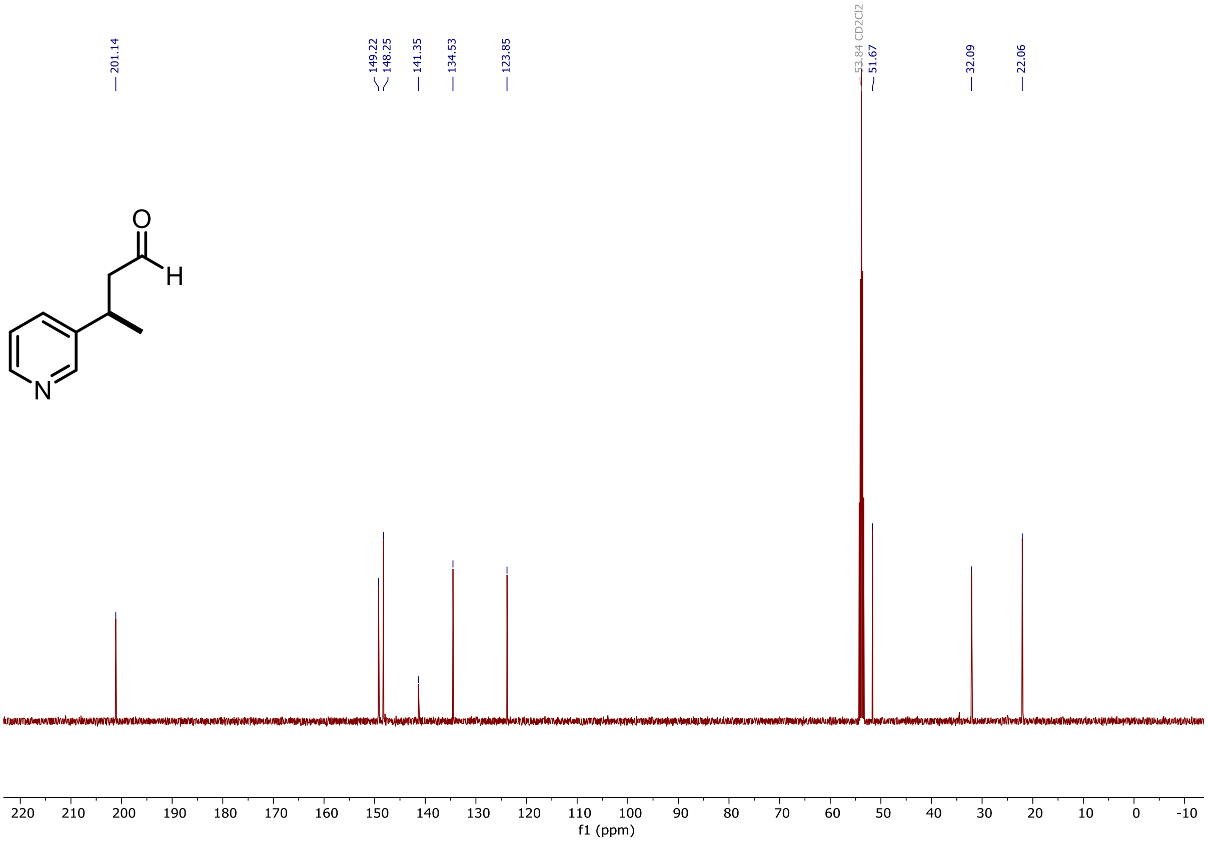

^{1}H NMR spectrum of 3l

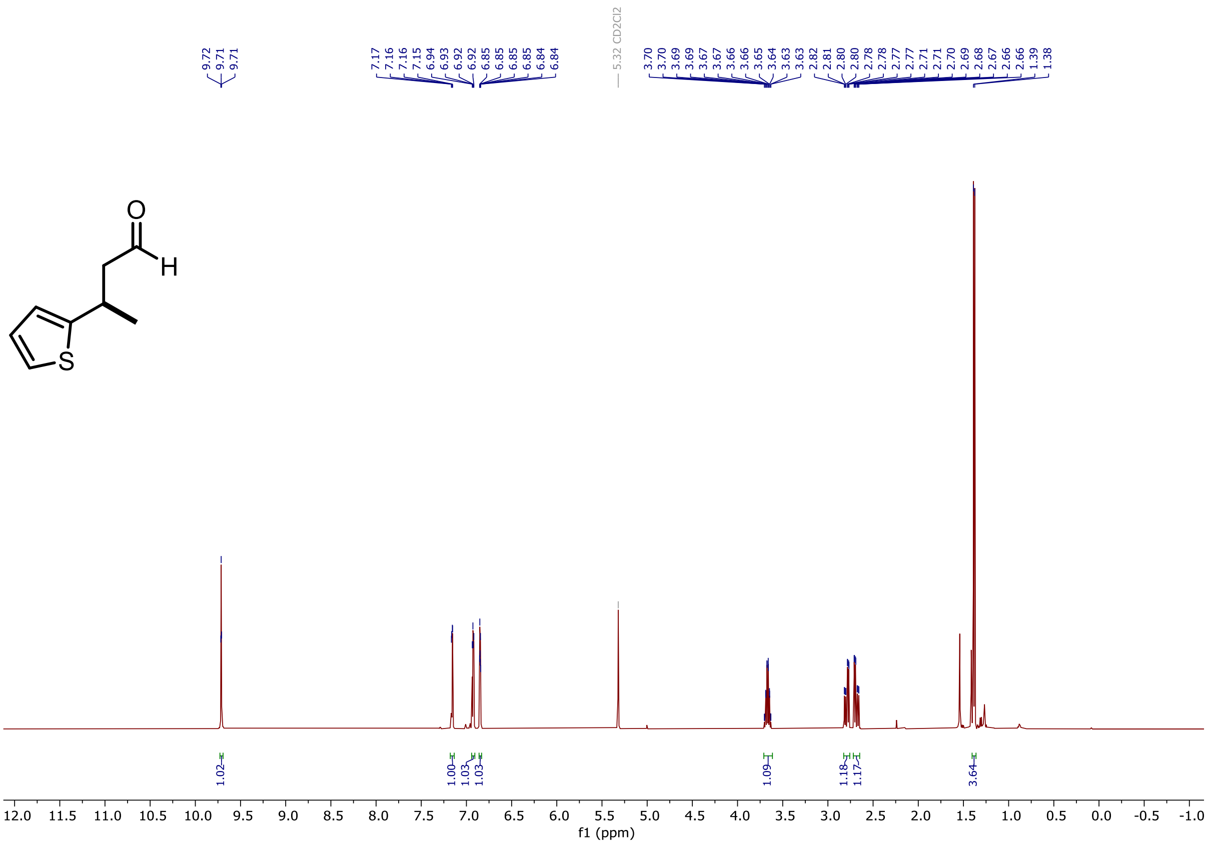

^{13}C NMR spectrum of 3l

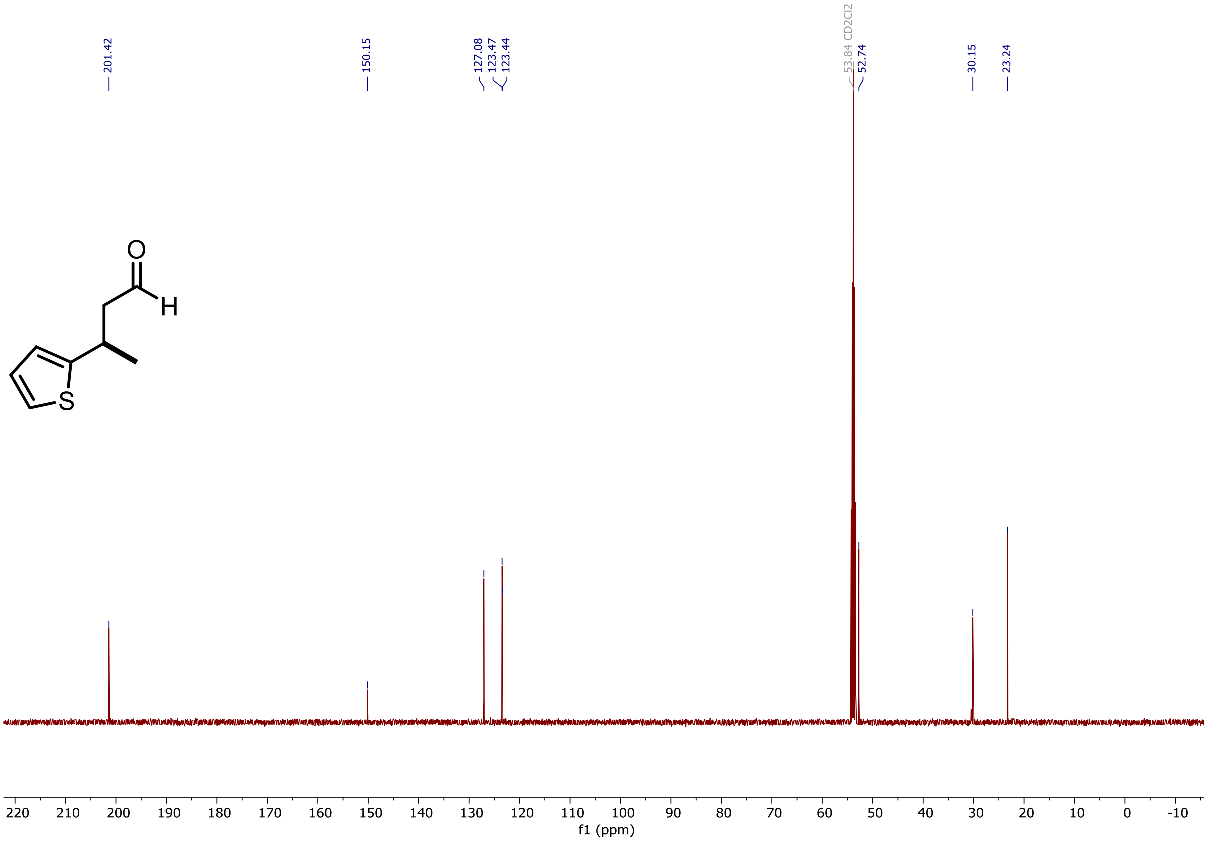

^{1}H NMR spectrum of 3m

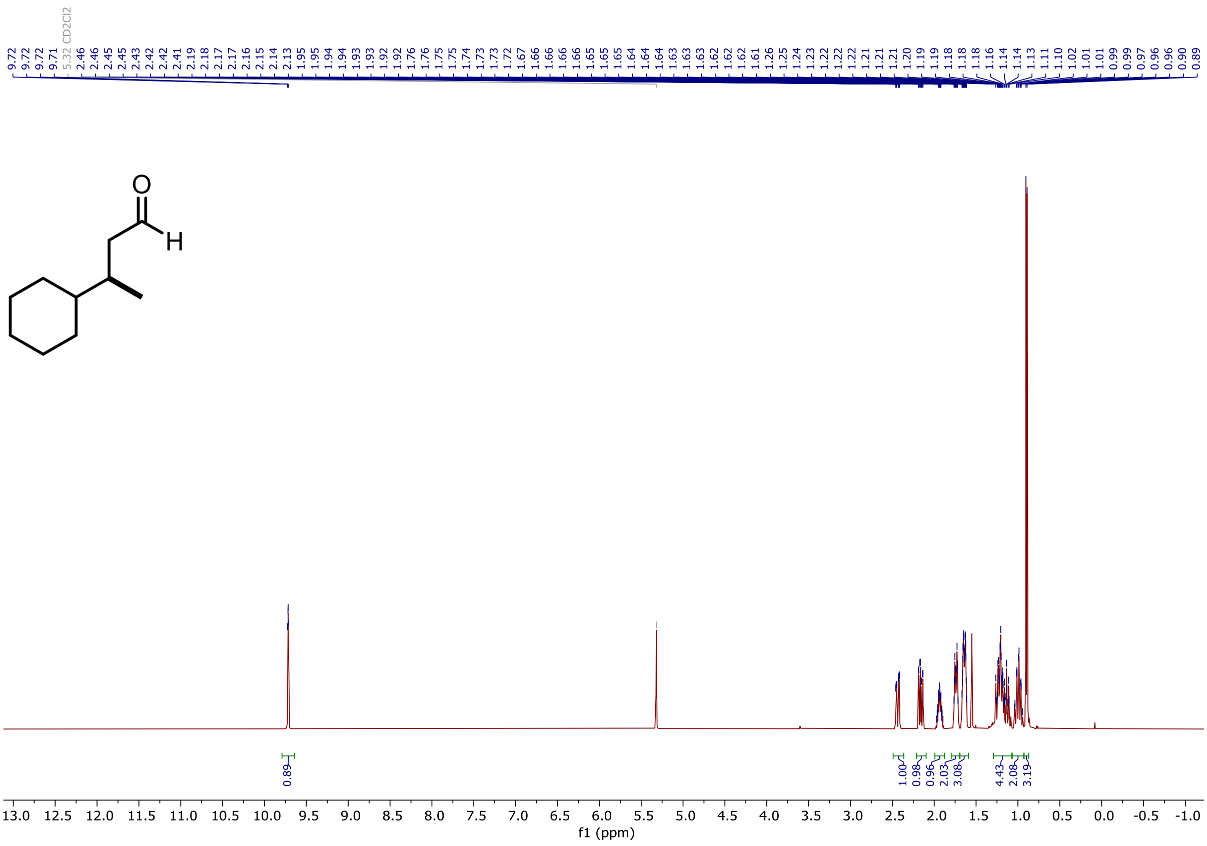

^{13}C NMR spectrum of 3m

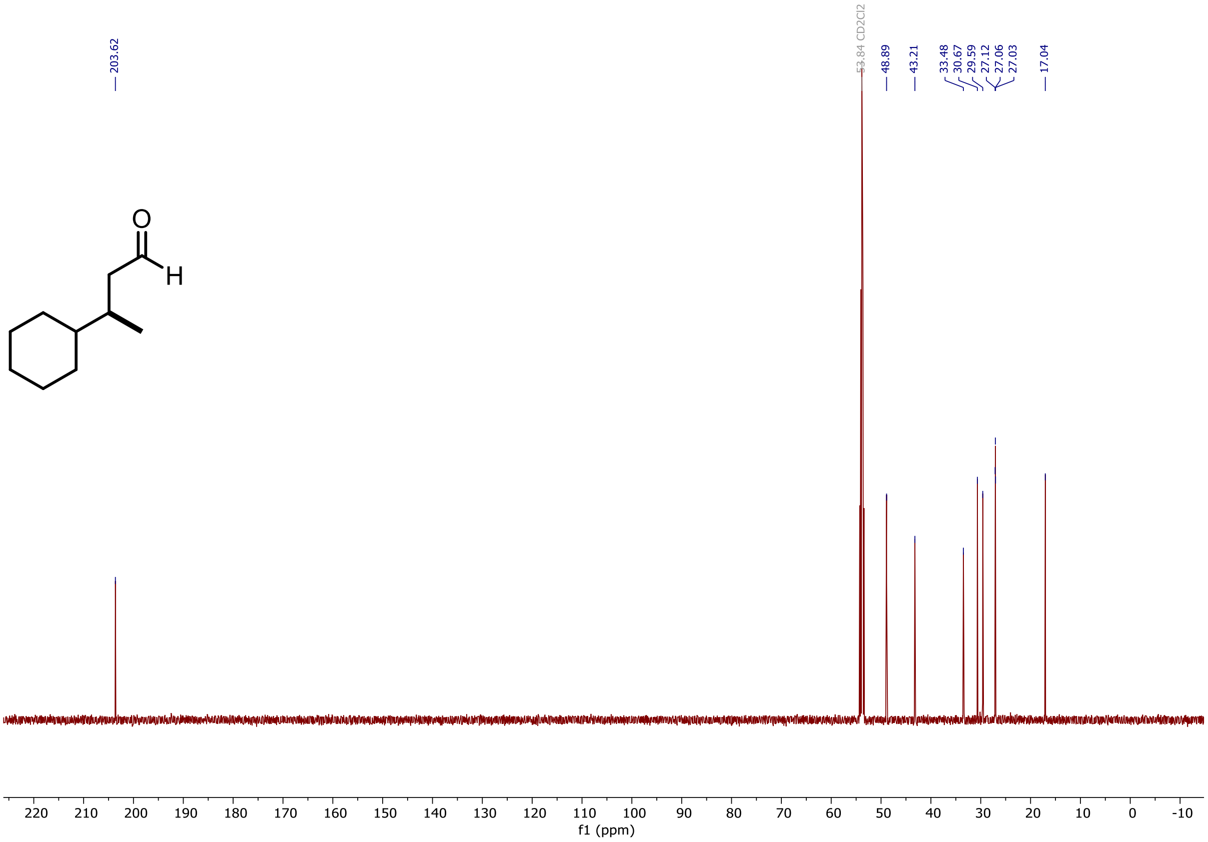

^{1}H NMR spectrum of 3n

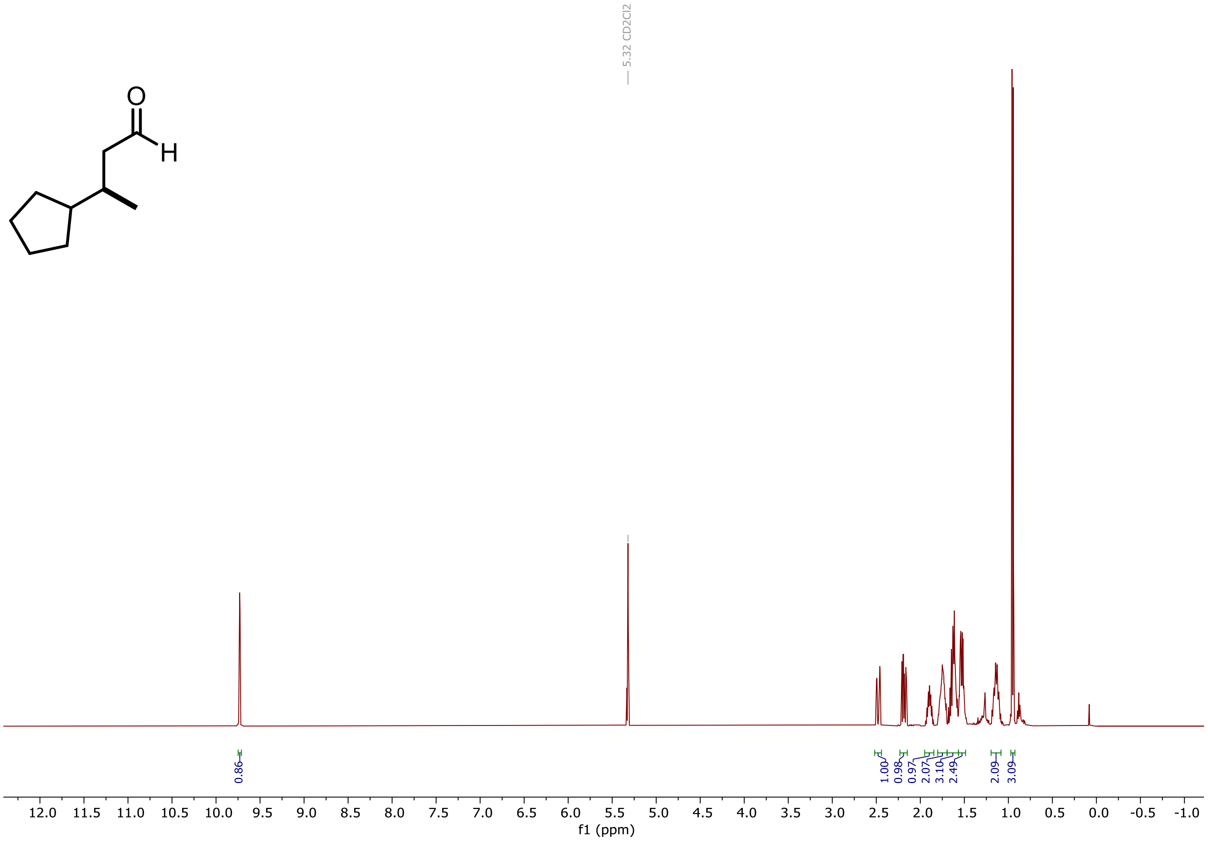

^{13}C NMR spectrum of 3n

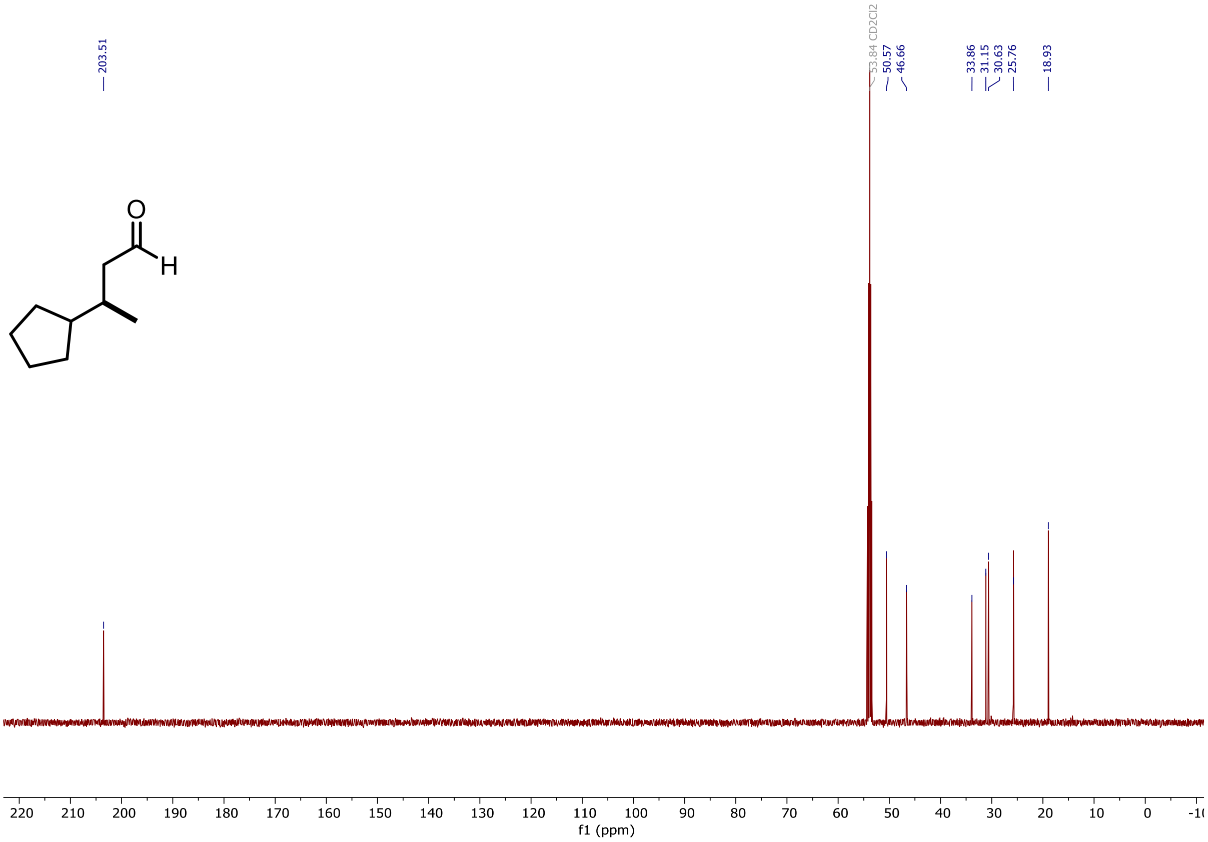

^{1}H NMR spectrum of 3o

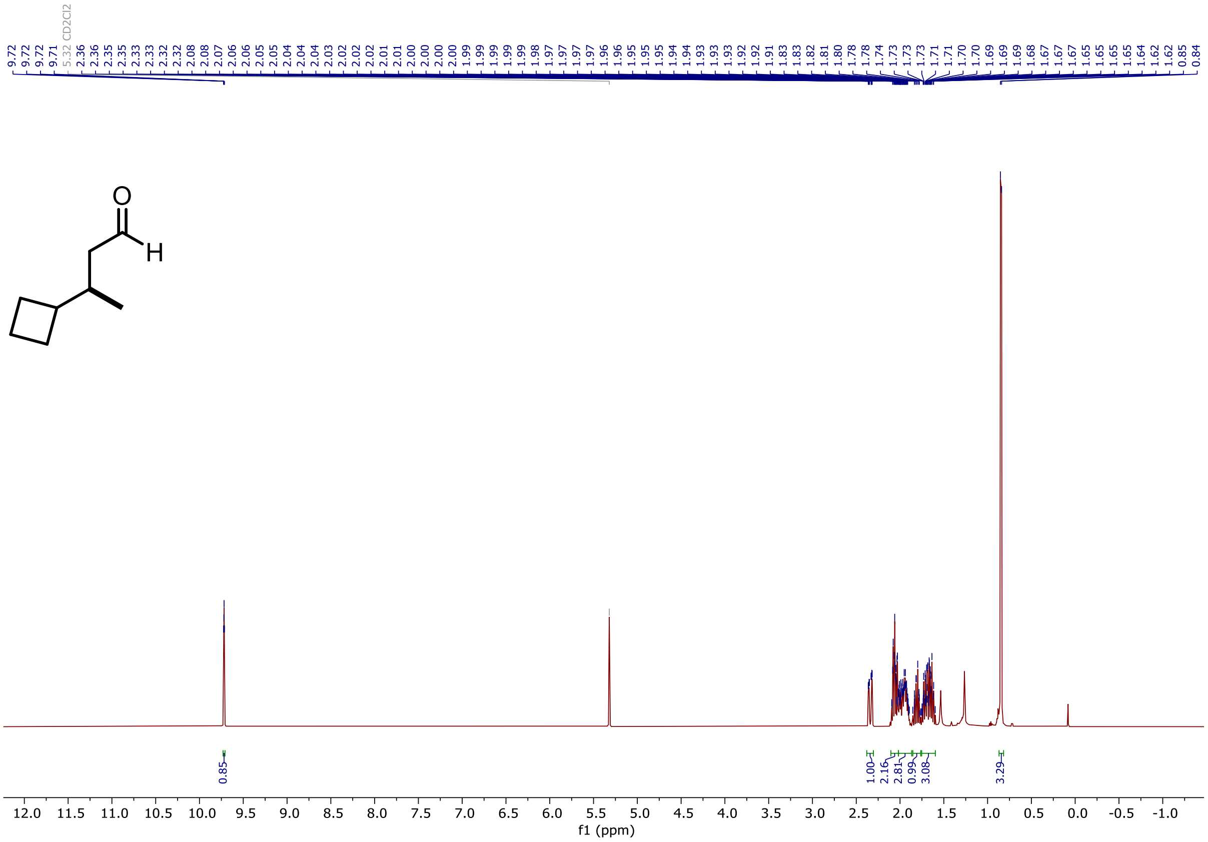

^{13}C NMR spectrum of 3o

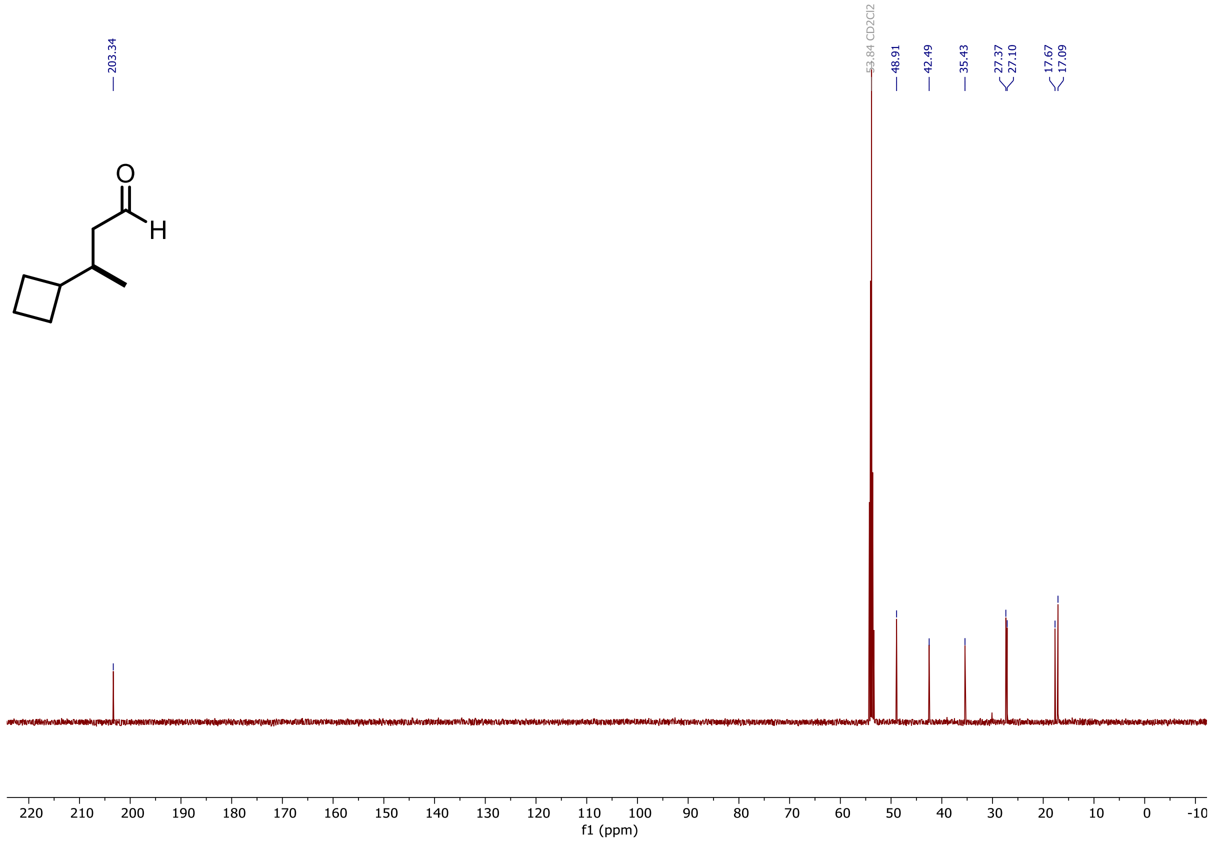

^{1}H NMR spectrum of 3p

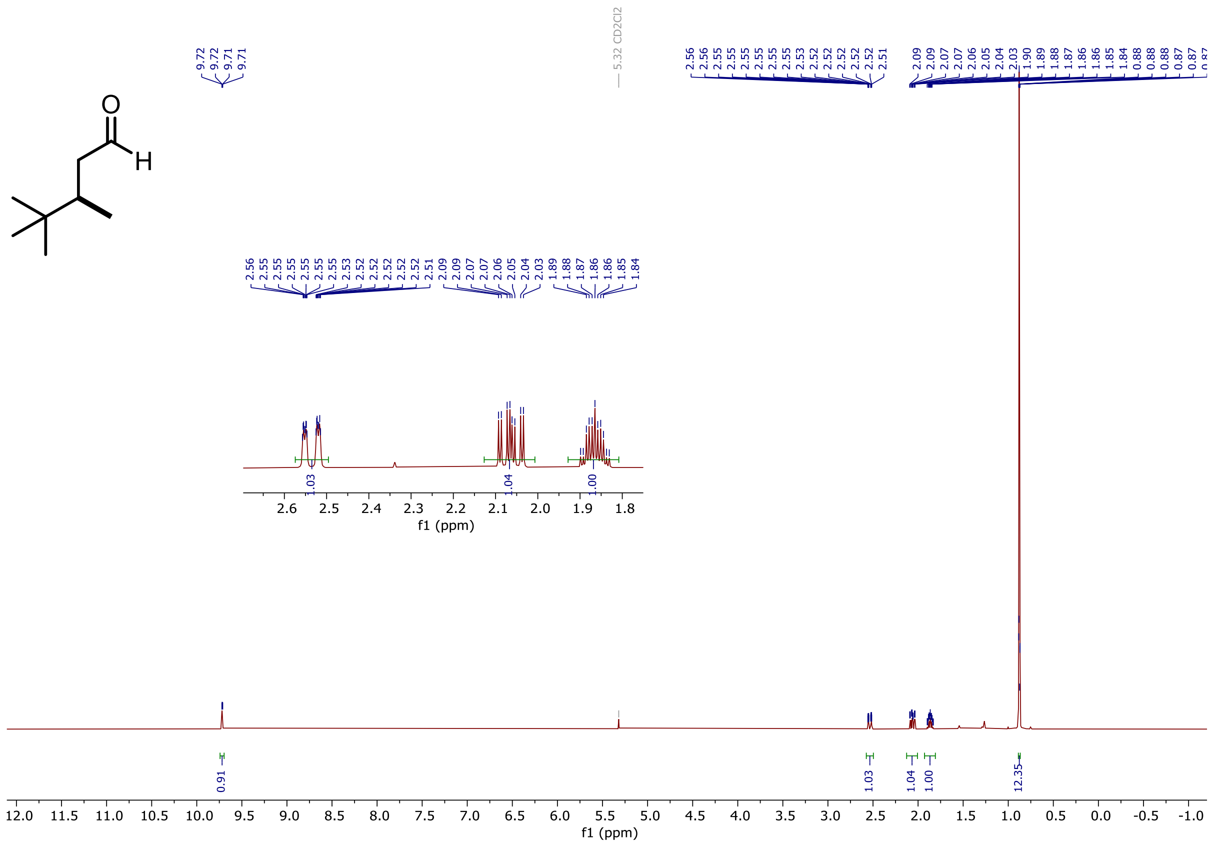

^{13}C NMR spectrum of 3p

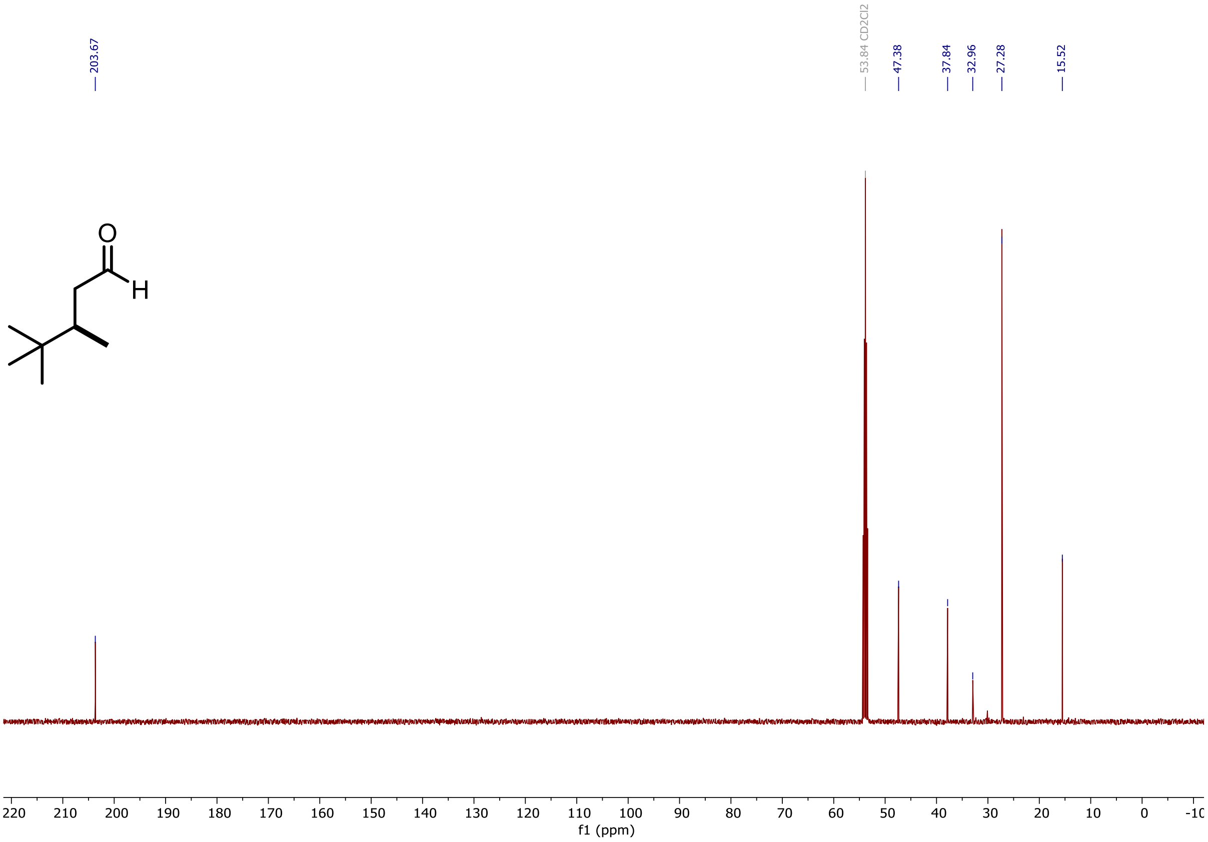

^{1}H NMR spectrum of 3q

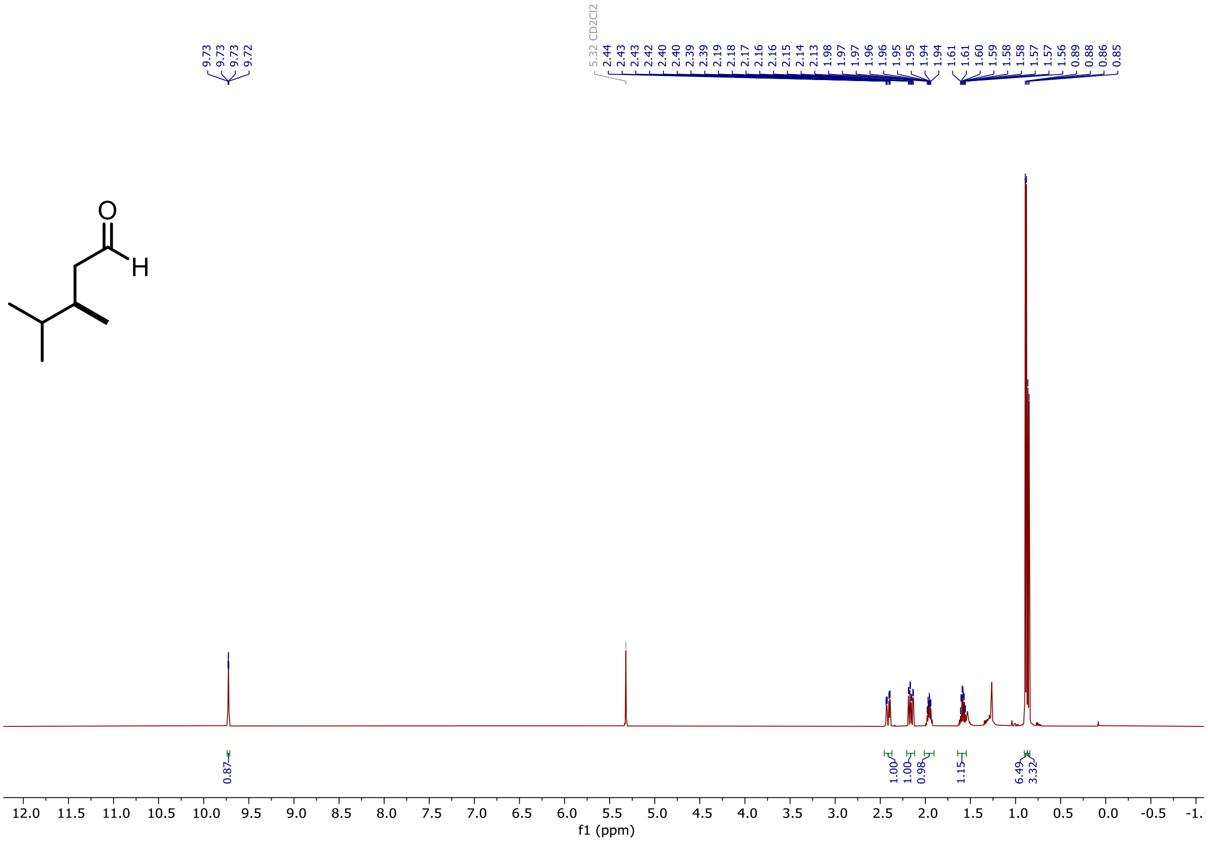

^{13}C NMR spectrum of 3q

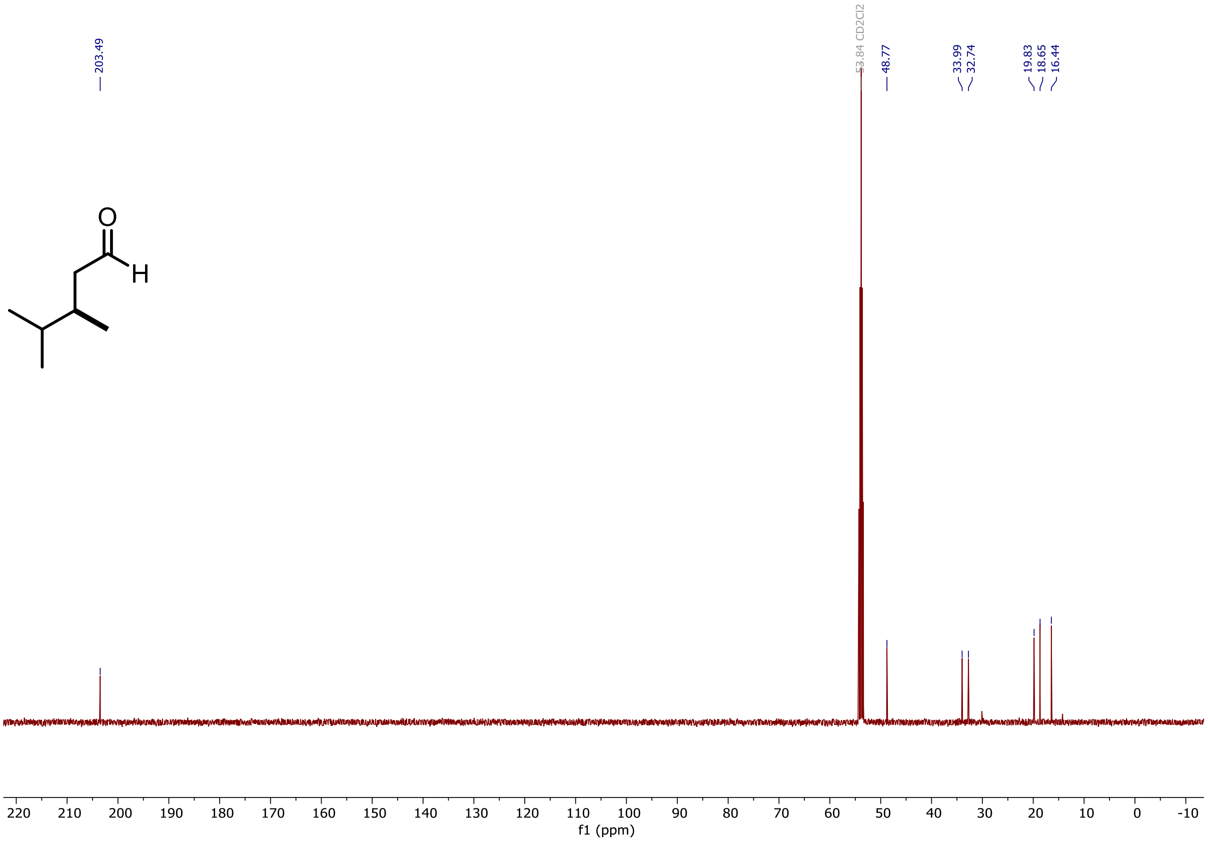

^{1}H NMR spectrum of 3ra

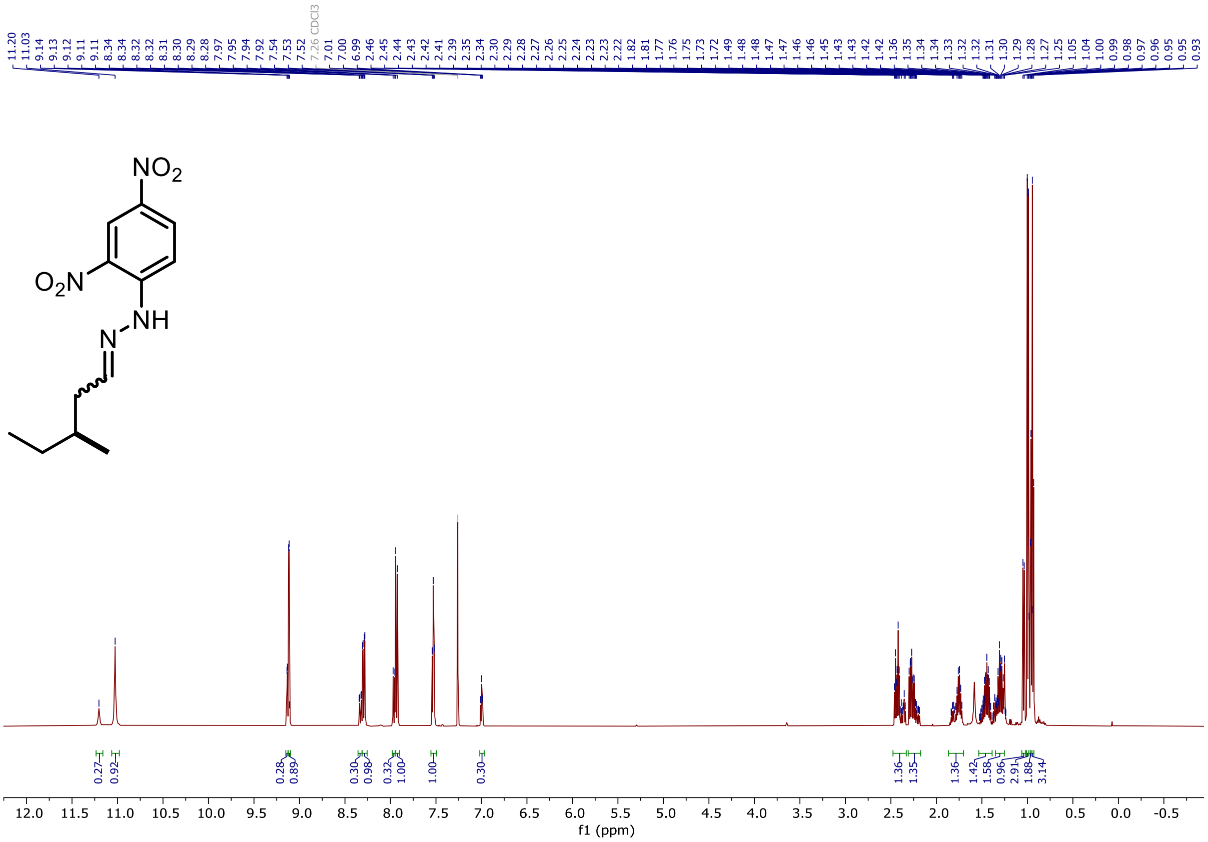

^{13}C NMR spectrum of 3ra

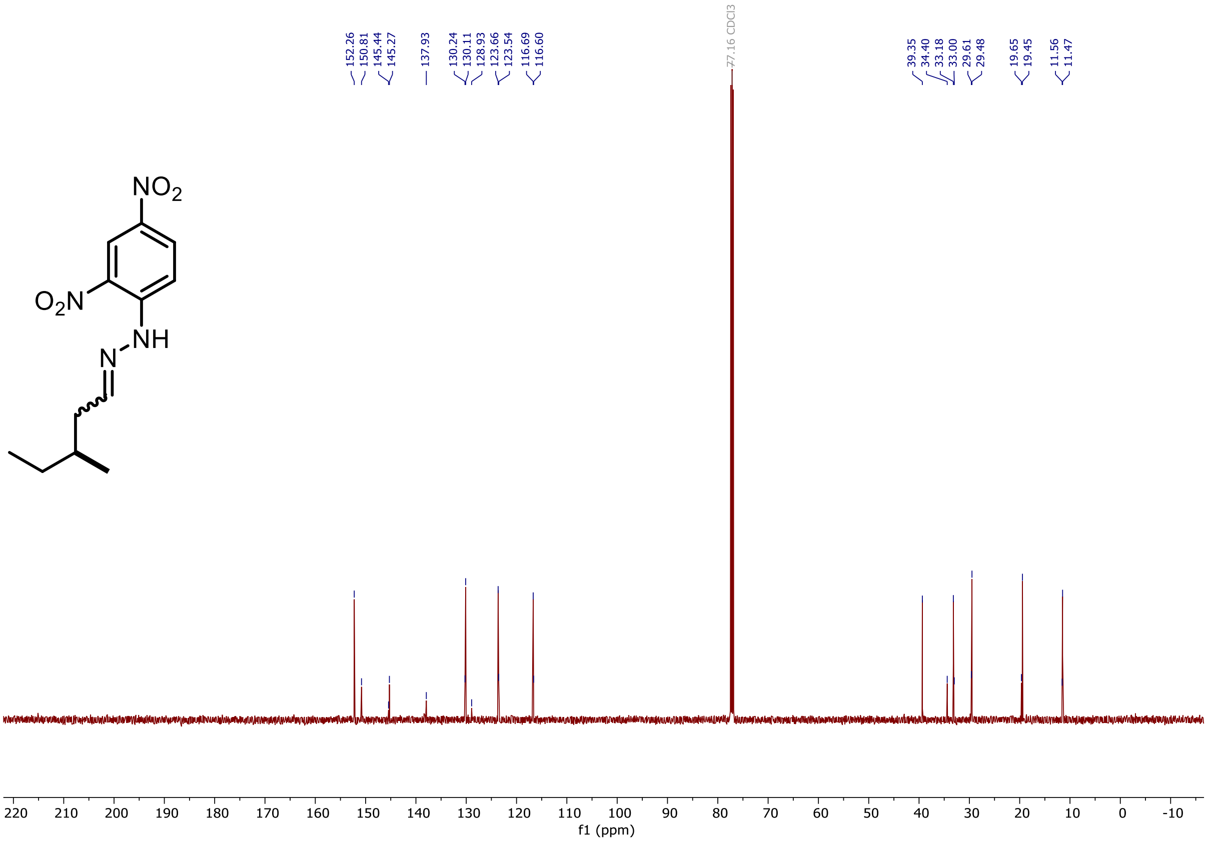

^{1}H NMR spectrum of 3sa

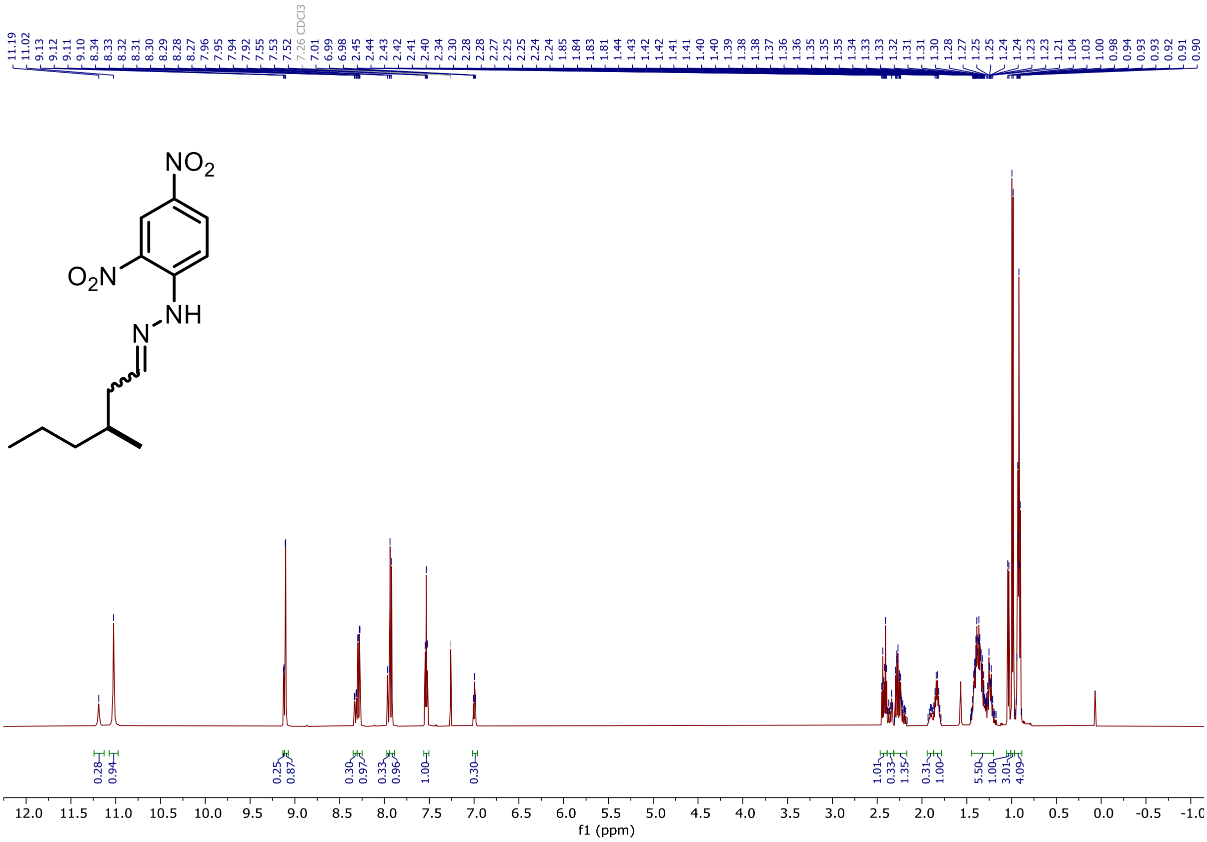

^{13}C NMR spectrum of 3sa

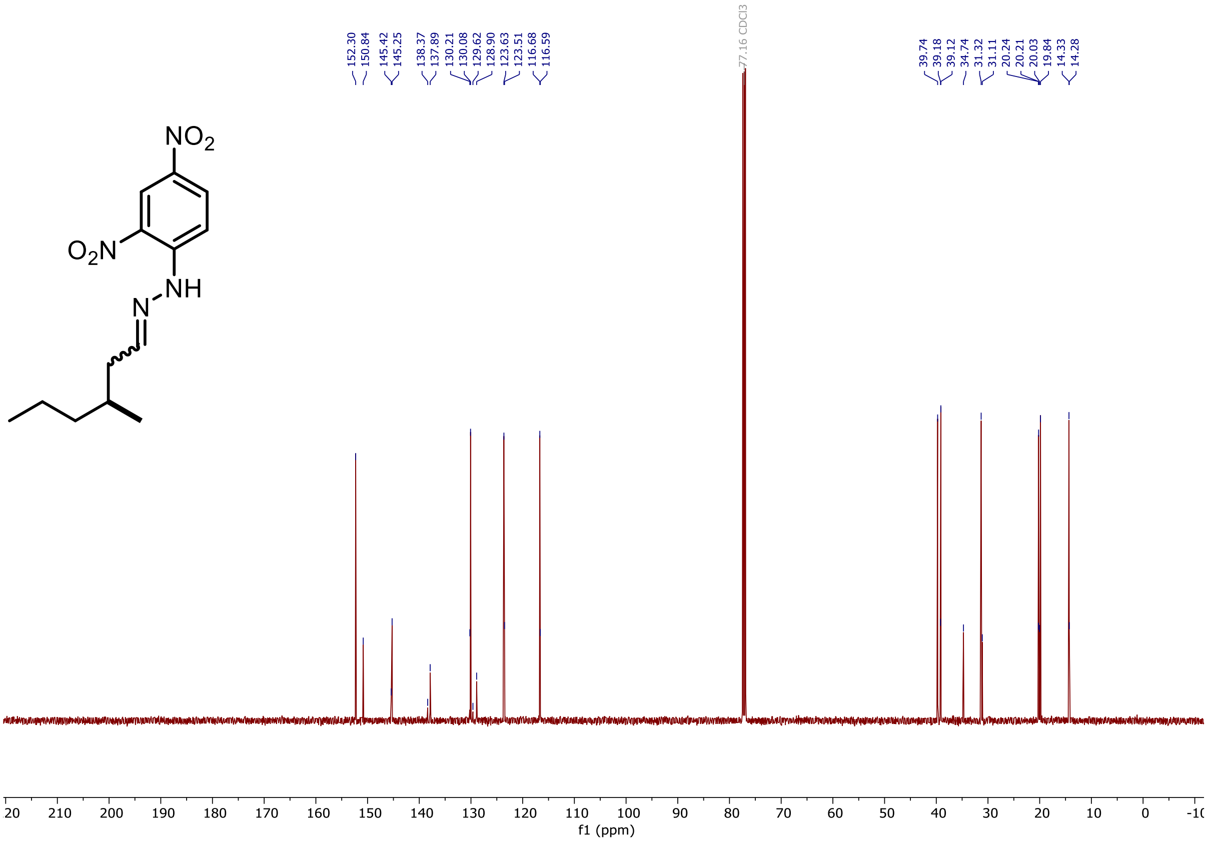

^{1}H NMR spectrum of 3t

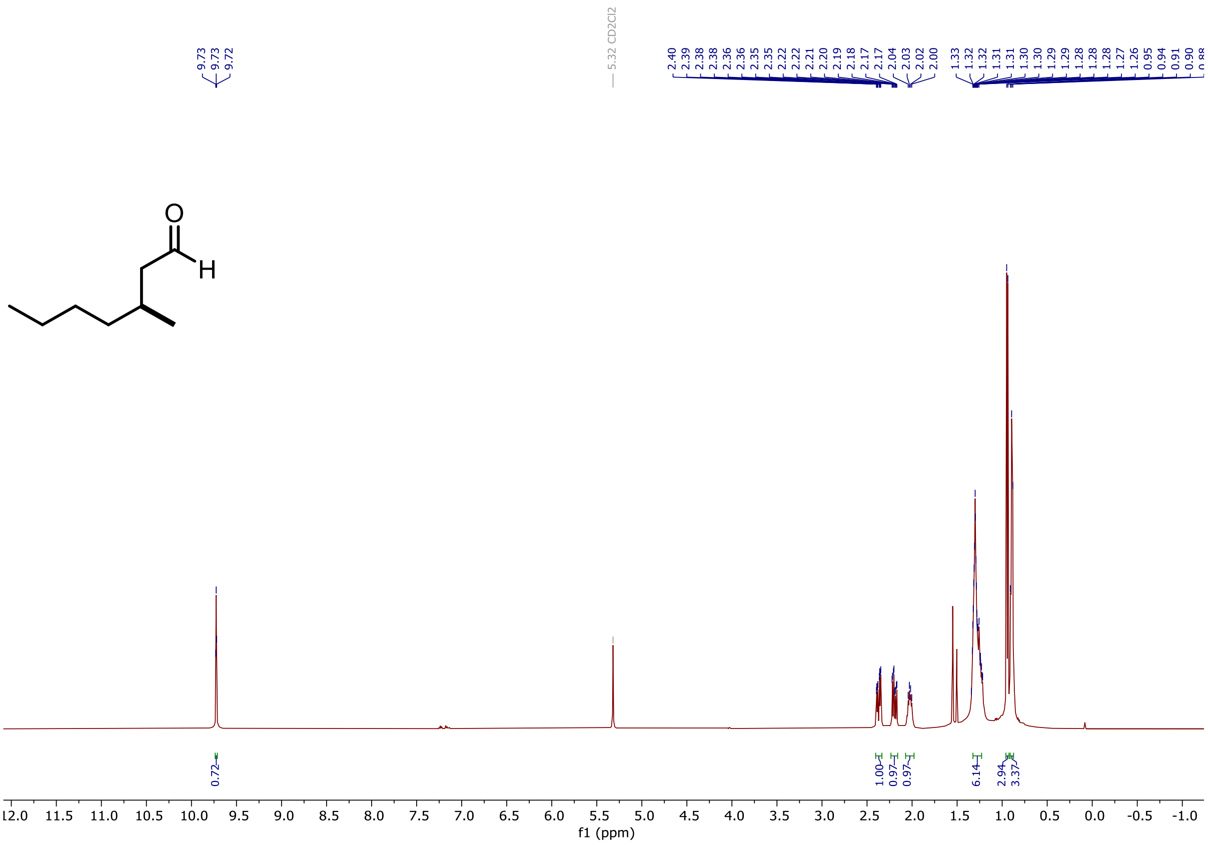

^{13}C NMR spectrum of 3t

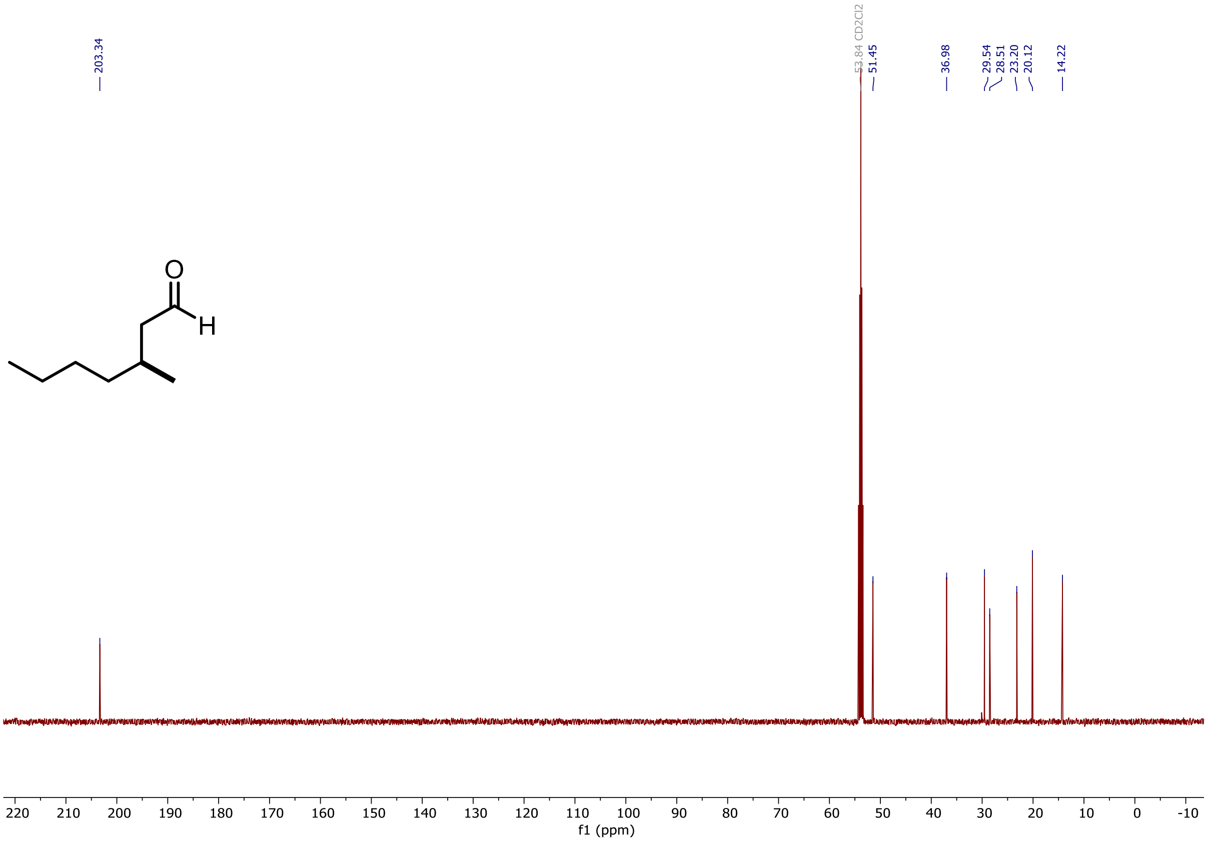

^{1}H NMR spectrum of 3u

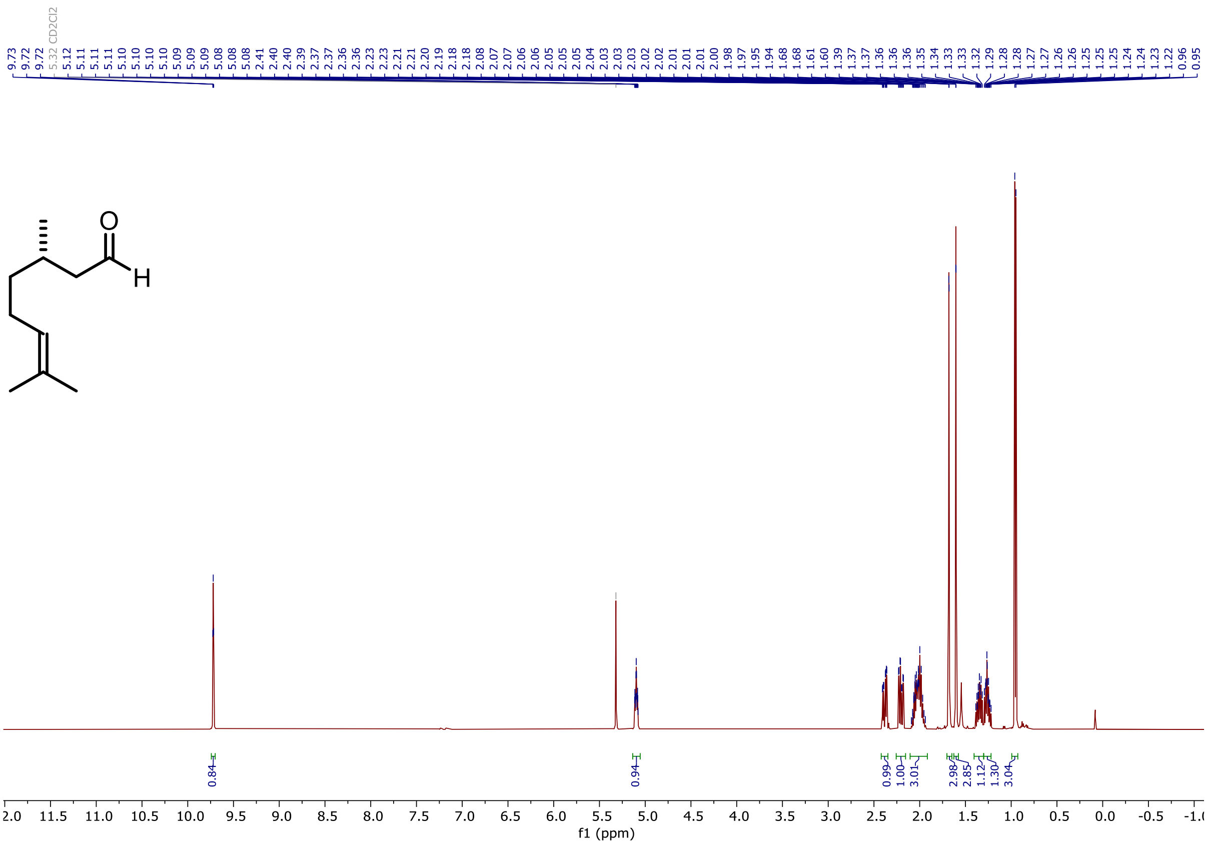

^{13}C NMR spectrum of 3u

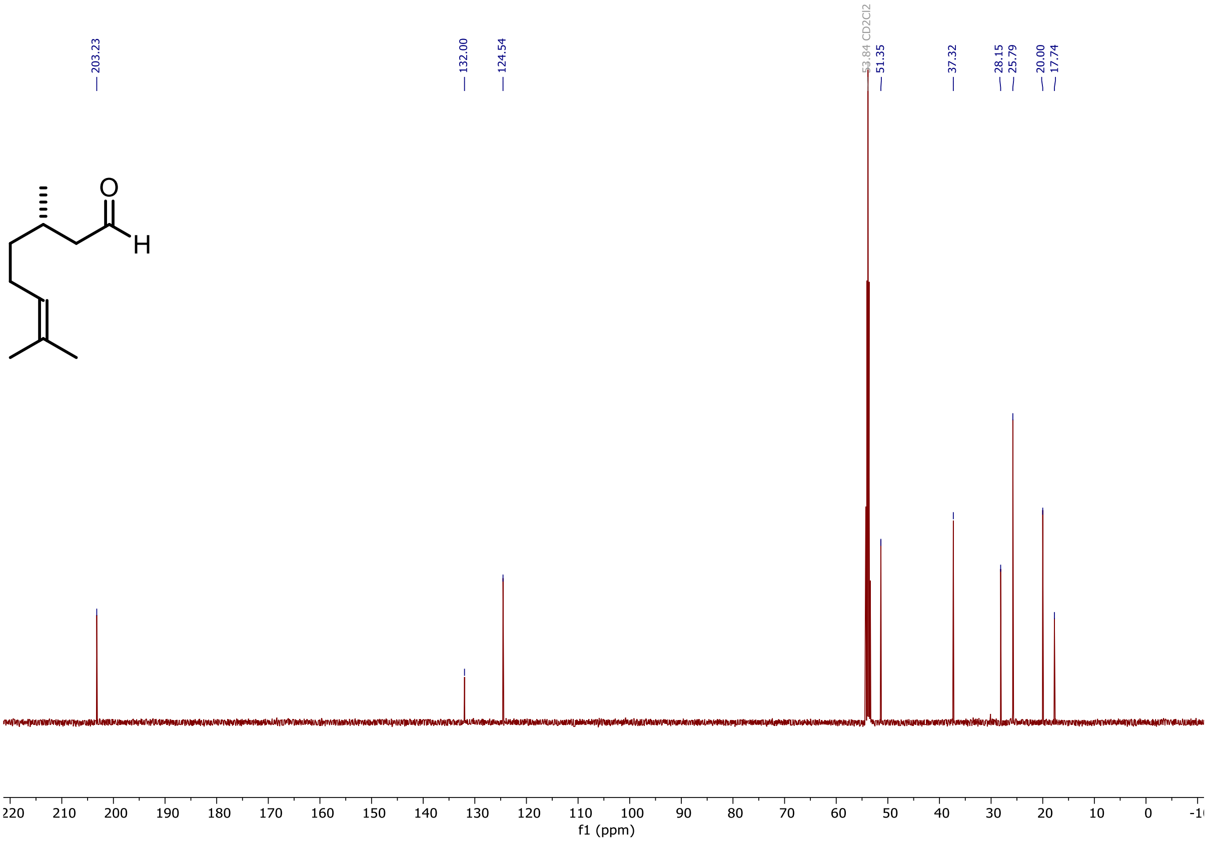

^{1}H NMR spectrum of 3v

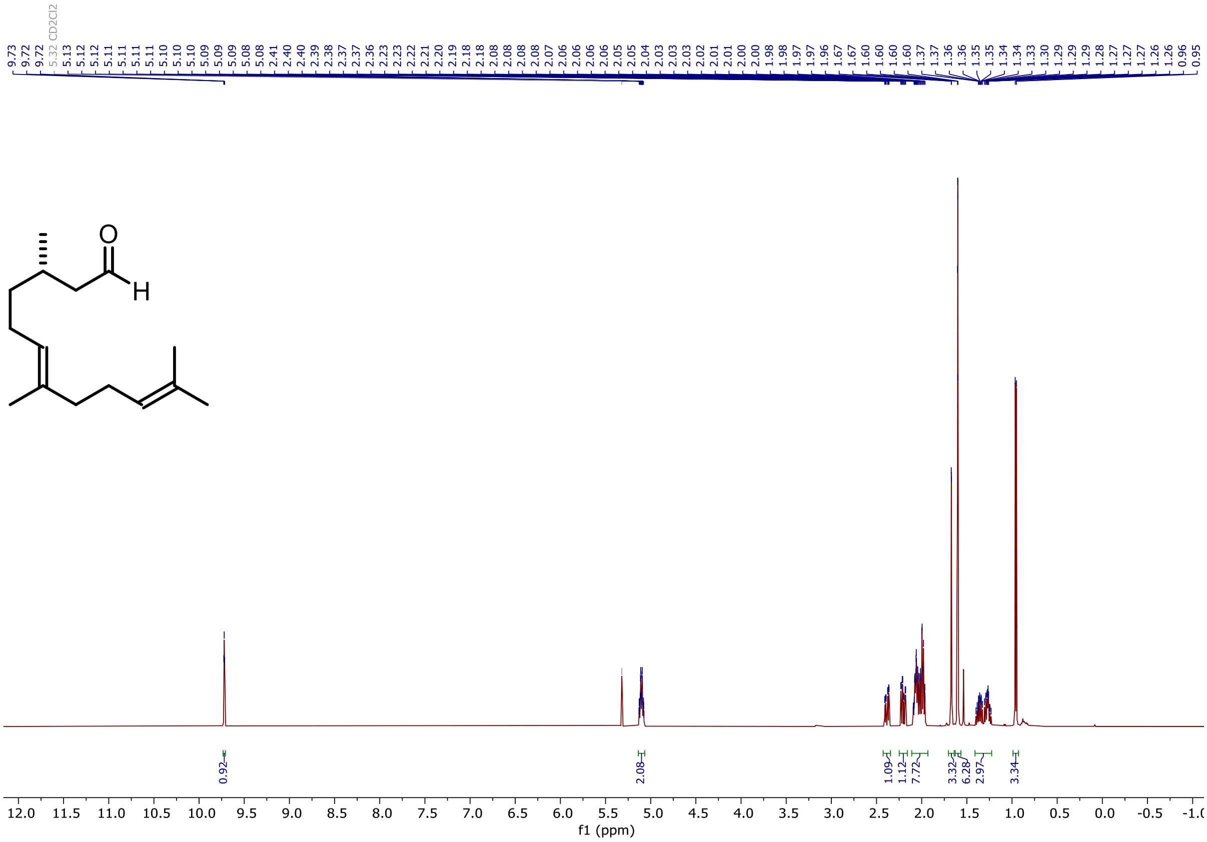

^{13}C NMR spectrum of 3v

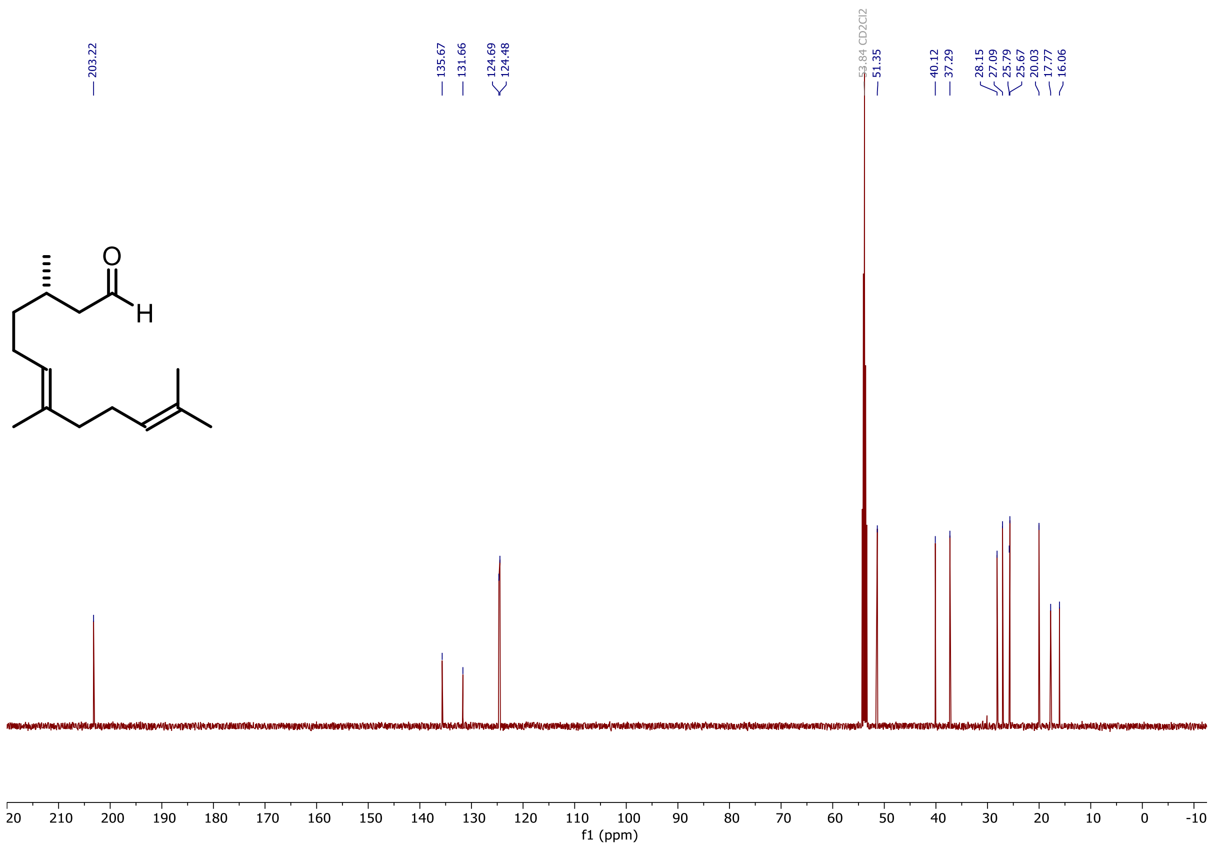

^{1}H NMR spectrum of 6e

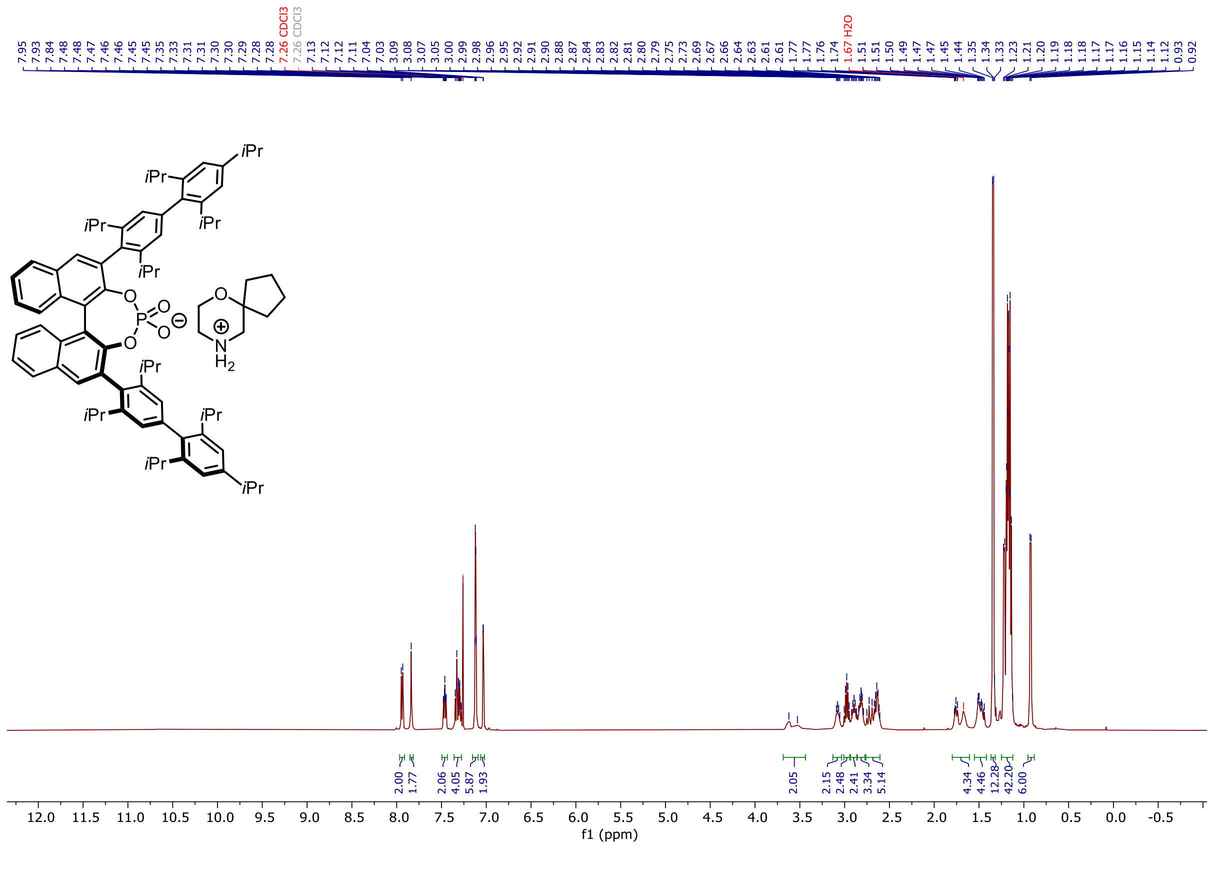

^{31}P NMR spectrum of 6e

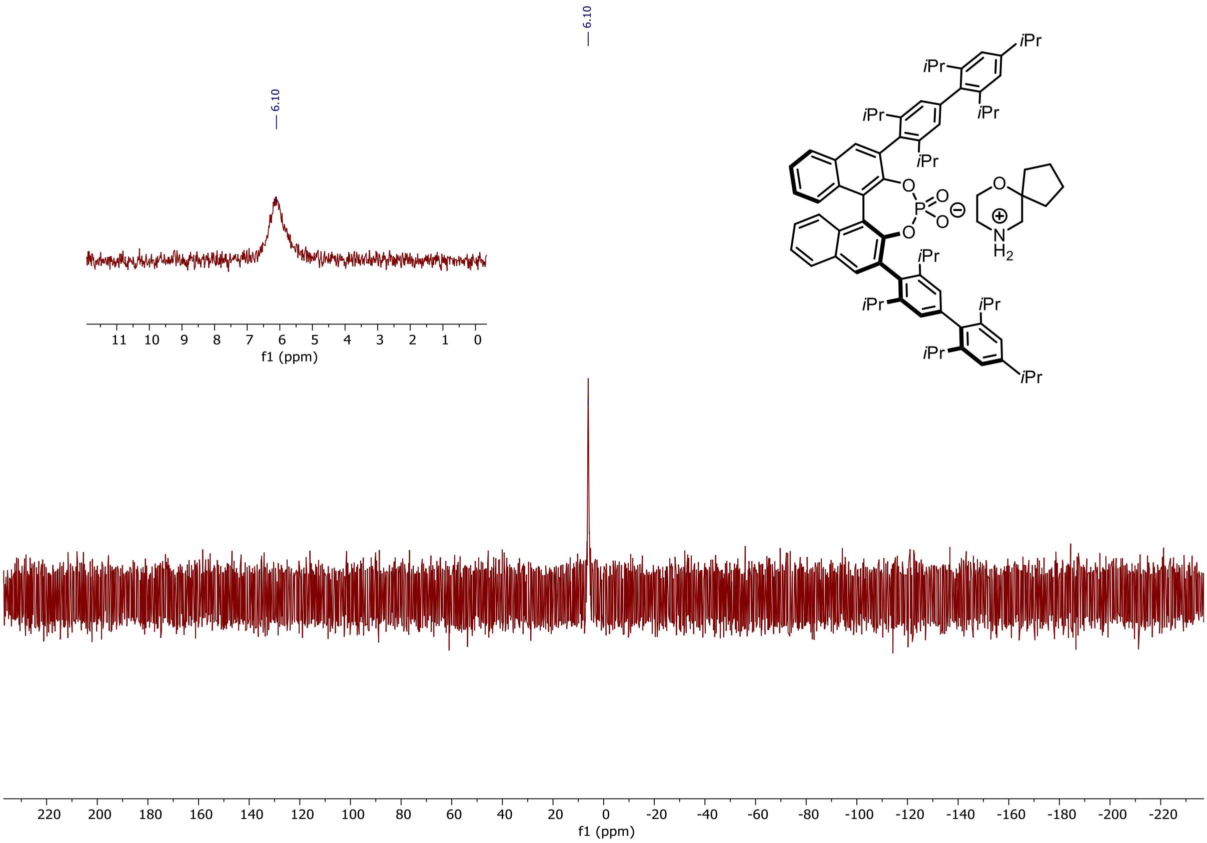

^{13}C NMR spectrum of 6e

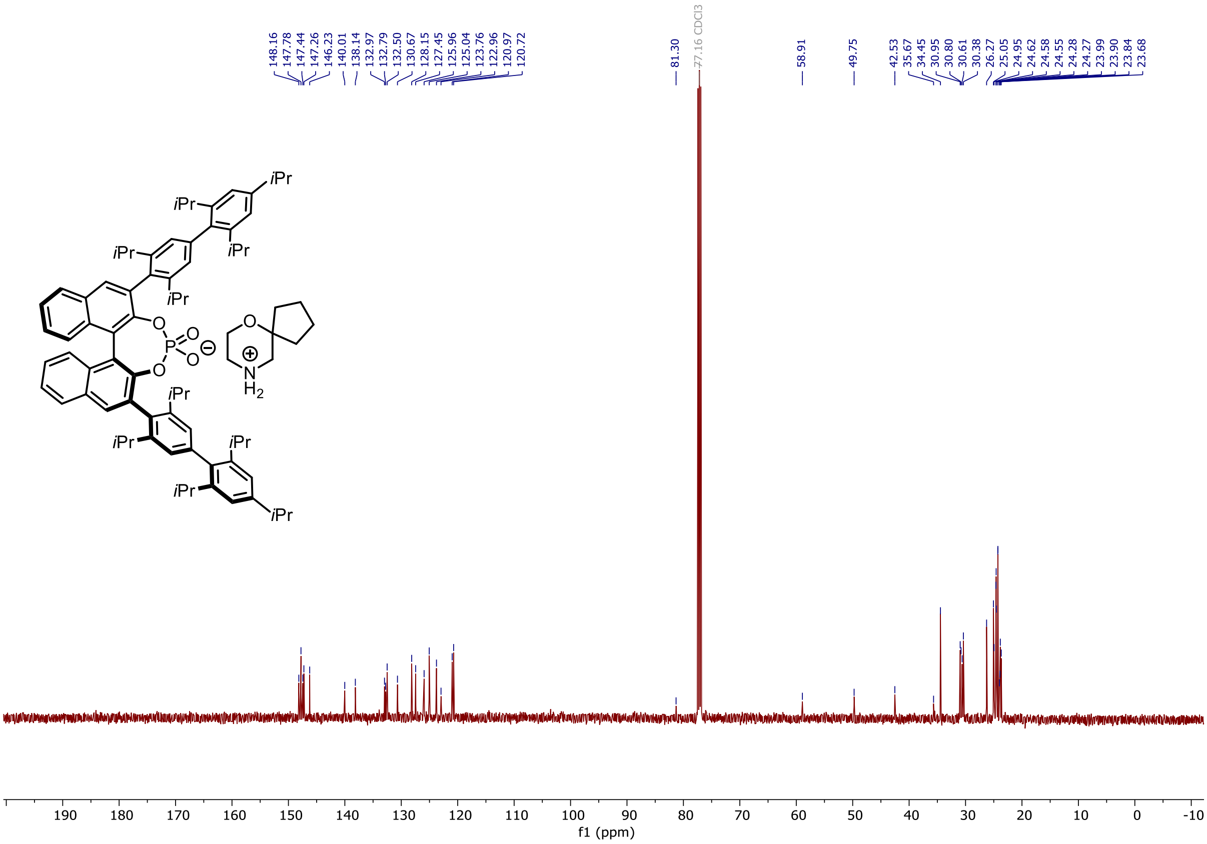

11. GC Traces

GC (30 m Ivadex-1, injection temperature: 220 °C, 110 °C iso 5 min, 2 °C/min, 150 °C iso 5 min, 5 °C/min, 200 °C iso 30 min, 5 °C/min, 220 °C iso 5 min, 0.5 bar H_2)

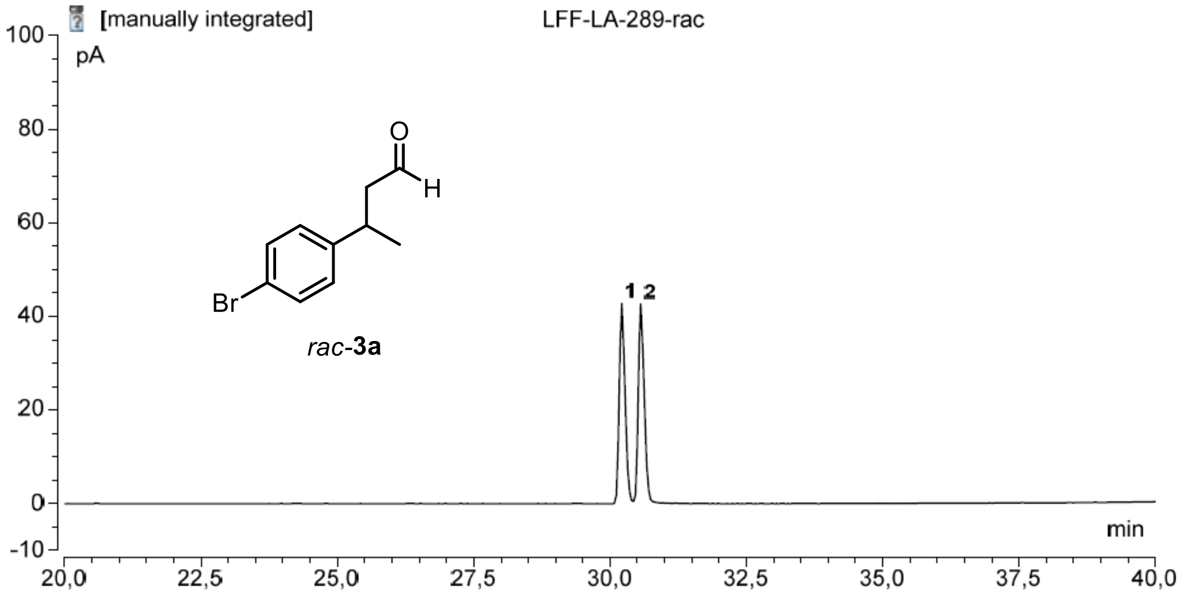

No.	Ret.Time min	Rel.Area %
1	30,22	49,92
2	30,56	50,08

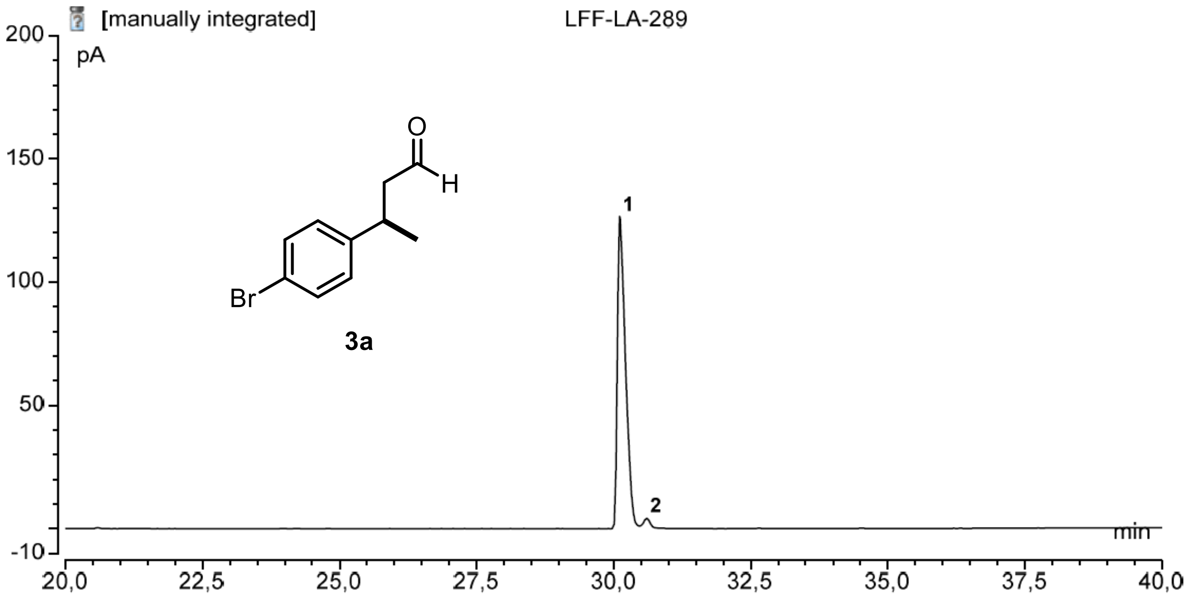

No.	Ret.Time min	Rel.Area %
1	30,11	97,33
2	30,60	2,67

GC (30 m Ivadex-1, injection temperature: 220 °C, 110 °C iso 5 min, 2 °C/min, 150 °C iso 5 min, 8 °C/min, 220 °C iso 5 min, 0.5 bar H_2)

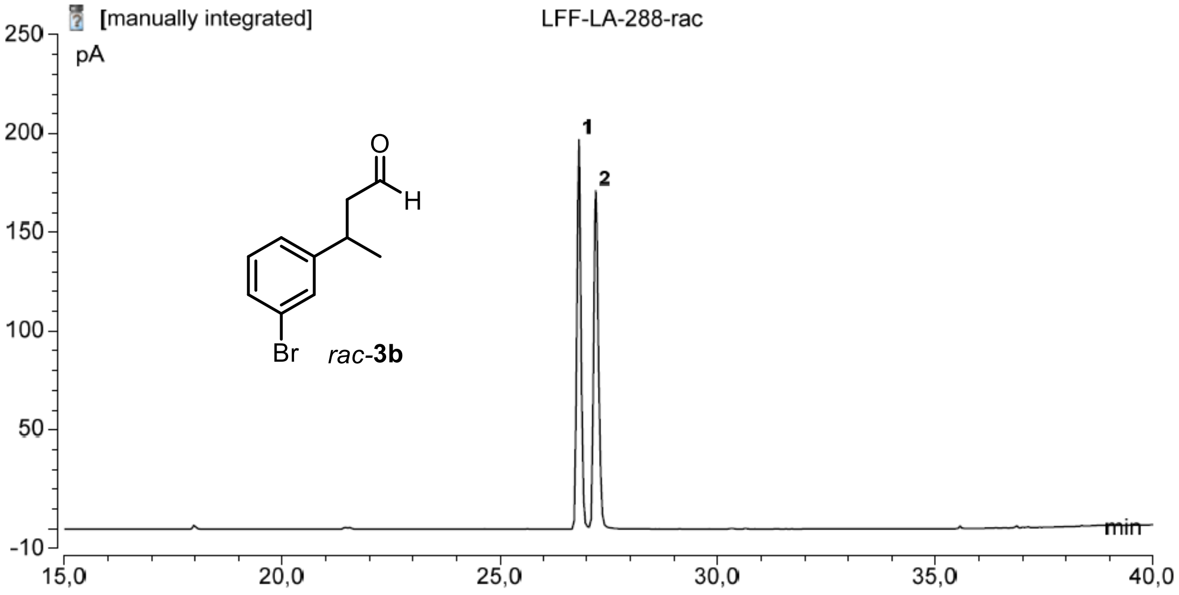

No.	Ret.Time min	Rel.Area %
1	26,82	49,80
2	27,20	50,20

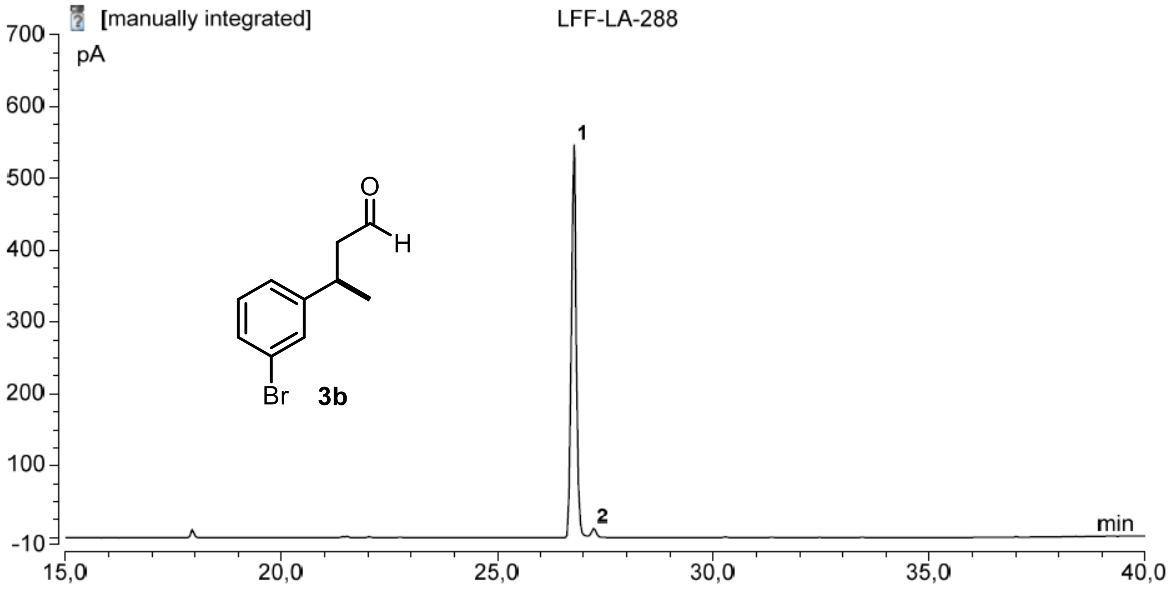

No.	Ret.Time min	Rel.Area %
1	26,78	97,64
2	27,24	2,36

GC (30 m Ivadex-1, injection temperature: 220 °C, 110 °C iso 5 min, 2 °C/min, 150 °C iso 5 min, 8 °C/min, 220 °C iso 5 min, 0.5 bar H_2)

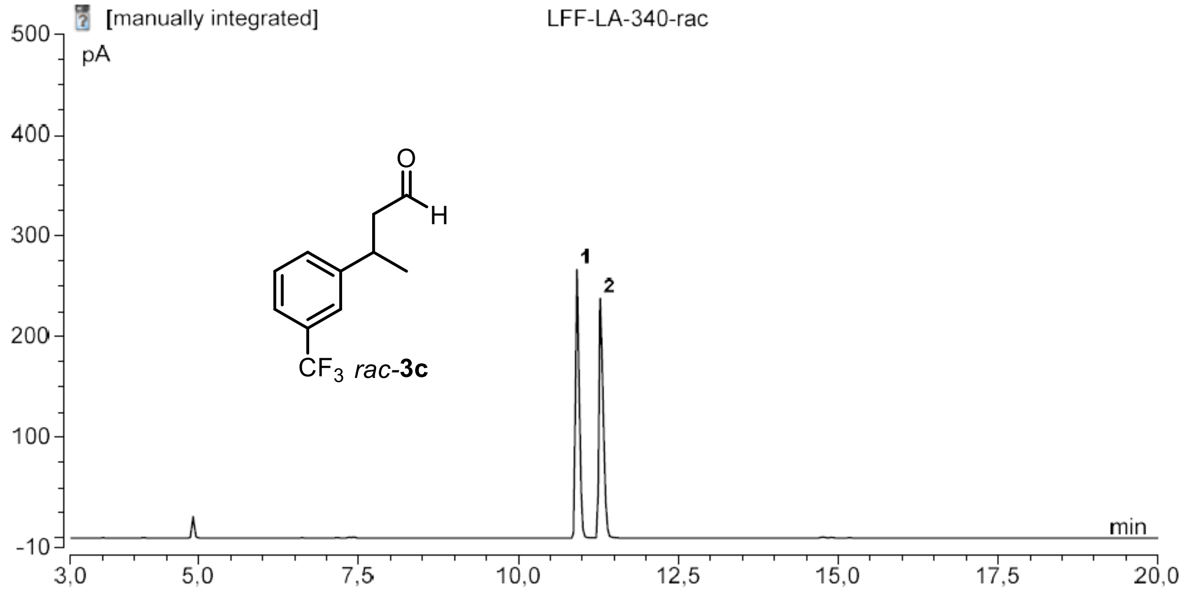

No.	Ret.Time min	Rel.Area %
1	10,92	49,99
2	11,28	50,01

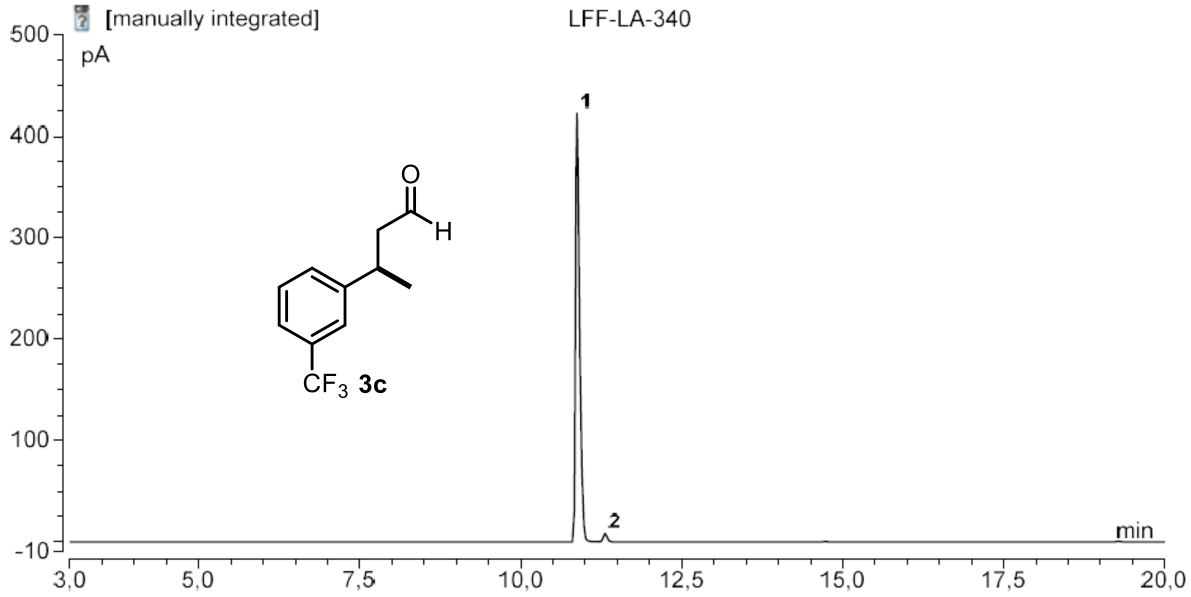

No.	Ret.Time min	Rel.Area %
1	10,87	98,23
2	11,31	1,77

GC (30 m Ivadex-1, injection temperature: 220 °C, 110 °C iso 5 min, 2 °C/min, 150 °C iso 5 min, 8 °C/min, 220 °C iso 5 min, 0.5 bar H_2)

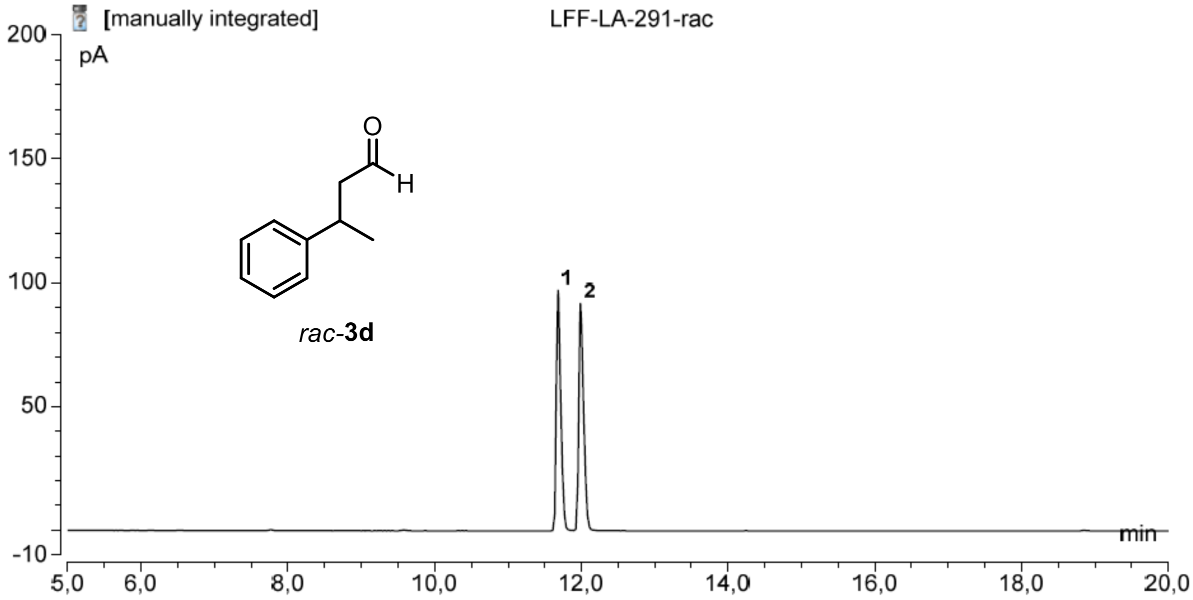

No.	Ret.Time min	Rel.Area %
1	11,68	49,87
2	11,99	50,13

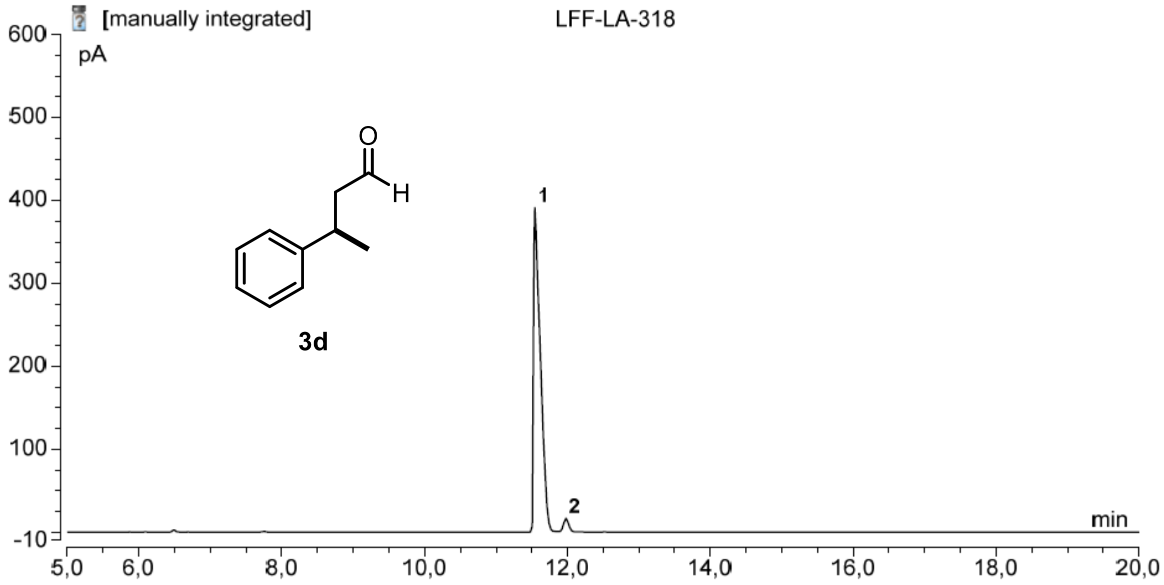

No.	Ret.Time min	Rel.Area %
1	11,54	97,04
2	11,98	2,96

GC (30 m Ivadex-1, injection temperature: 220 °C, 110 °C iso 5 min, 2 °C/min, 150 °C iso 5 min, 8 °C/min, 220 °C iso 5 min, 0.5 bar H_2)

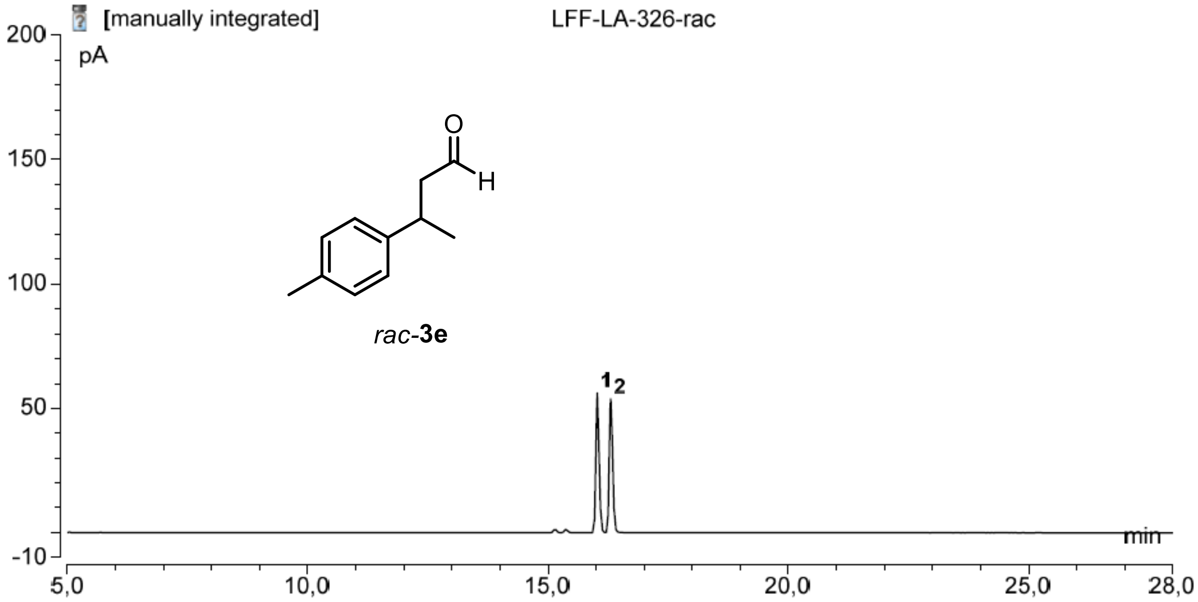

No.	Ret.Time min	Rel.Area %
1	16,03	49,97
2	16,31	50,03

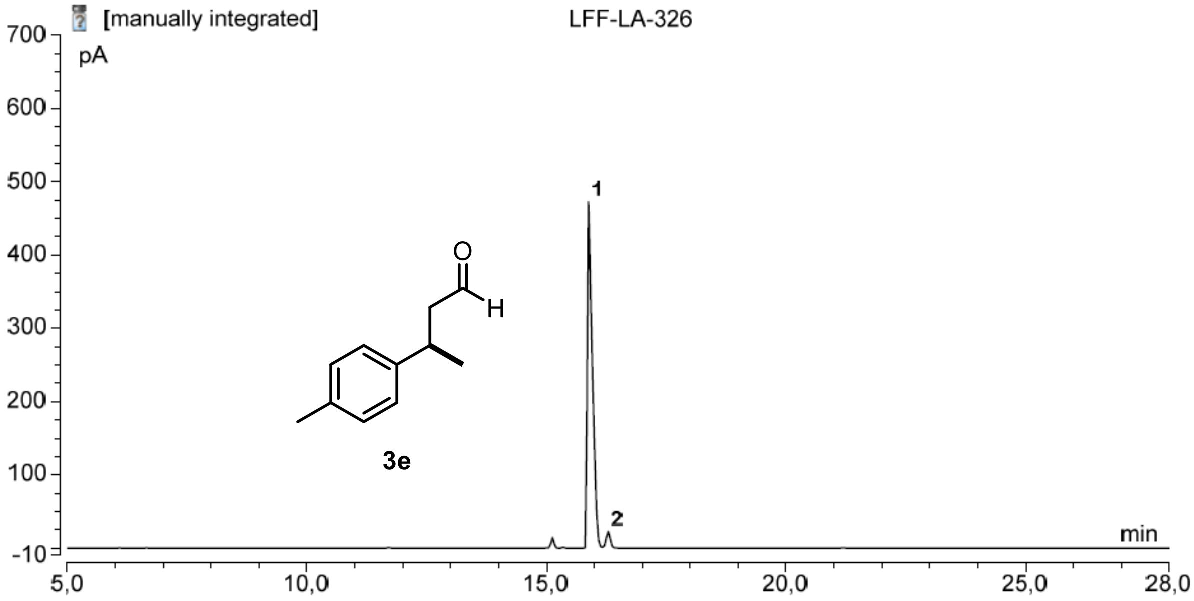

No.	Ret.Time min	Rel.Area %
1	15,88	96,50
2	16,29	3,50

GC (30 m Ivadex-1, injection temperature: 220 °C, 110 °C iso 5 min, 2 °C/min, 150 °C iso 5 min, 8 °C/min, 220 °C iso 5 min, 0.5 bar H_2)

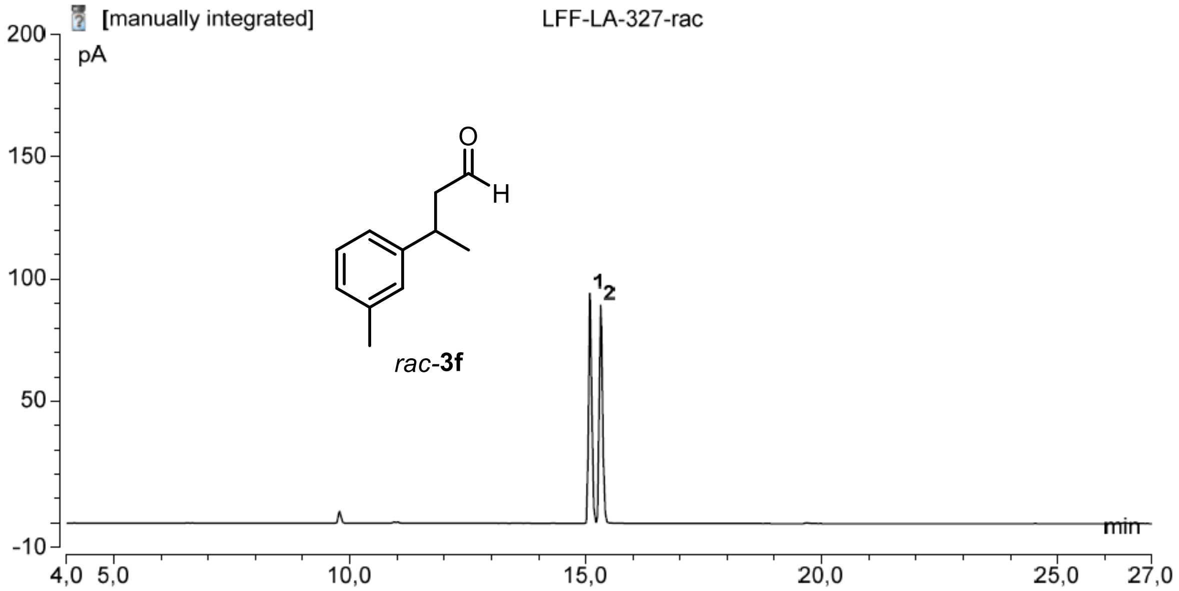

No.	Ret.Time min	Rel.Area %
1	15,09	50,00
2	15,32	50,00

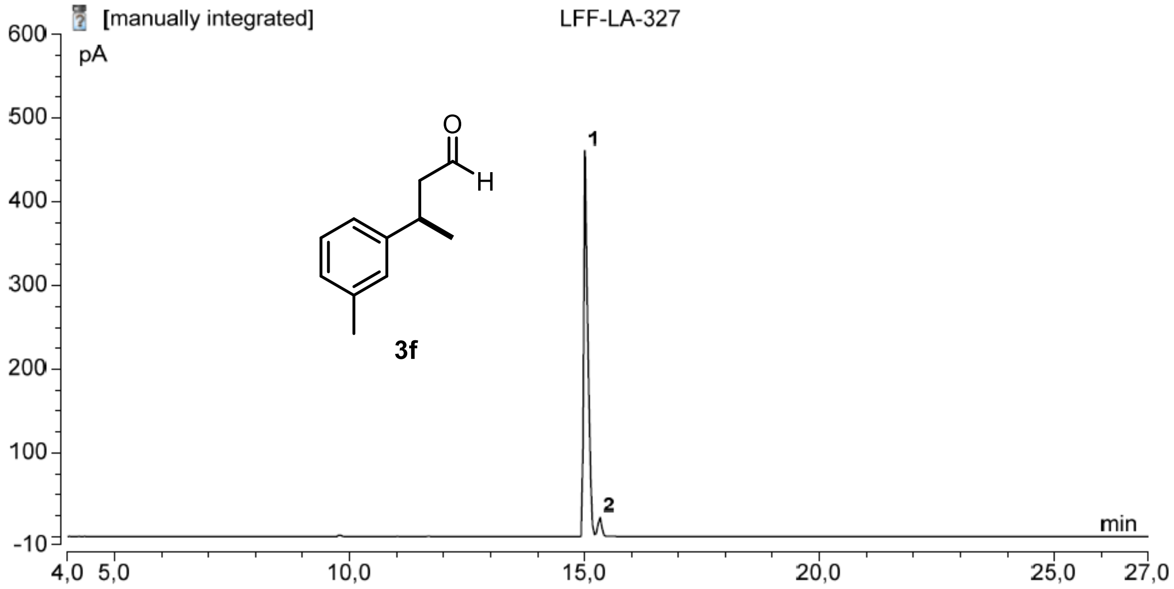

No.	Ret.Time min	Rel.Area %
1	15,01	95,55
2	15,33	4,45

GC (30 m Ivadex-1, injection temperature: 220 °C, 110 °C iso 30 min, 2 °C/min, 130 °C iso 2 min, 8 °C/min, 220 °C iso 5 min, 0.5 bar H_2)

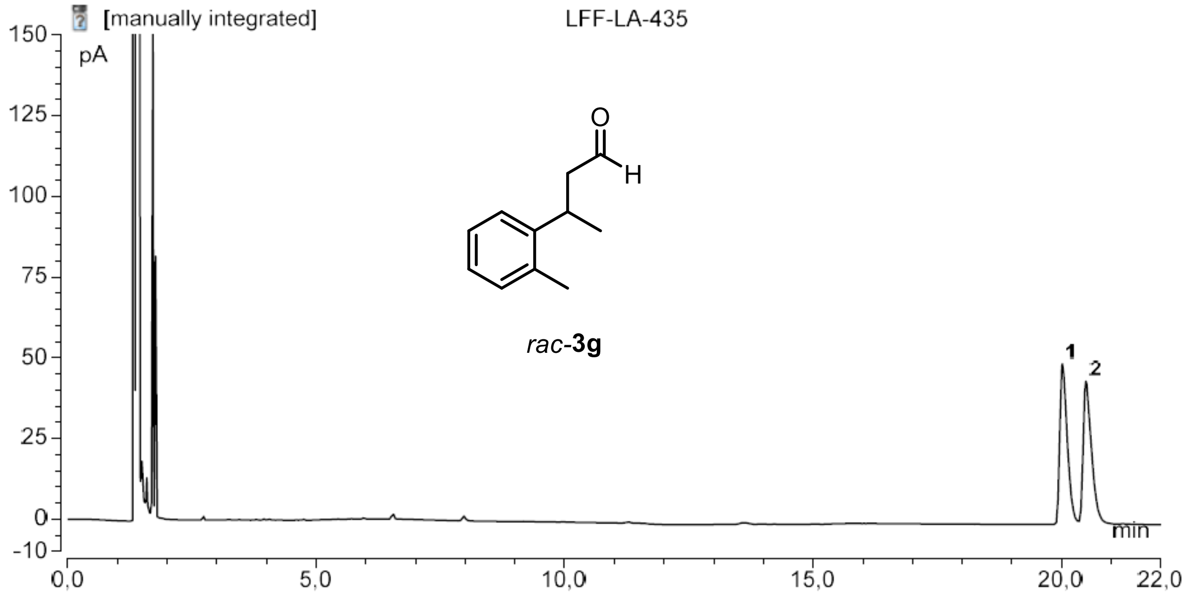

No.	Ret.Time min	Rel.Area %
1	20,02	50,14
2	20,49	49,86

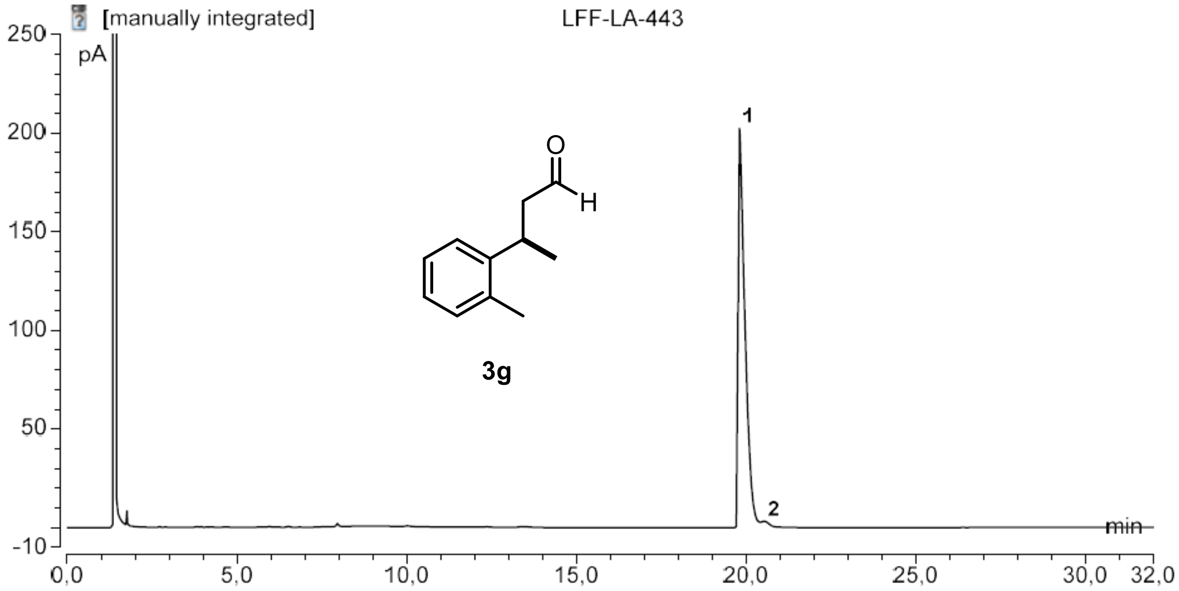

No.	Ret.Time min	Rel.Area %
1	19,80	98,29
2	20,53	1,71

GC (25 m Ivadex-1, injection temperature: 220 °C, 140 °C iso 150 min, 8 °C/min, 220 °C iso 3 min, 0.5 bar H_2)

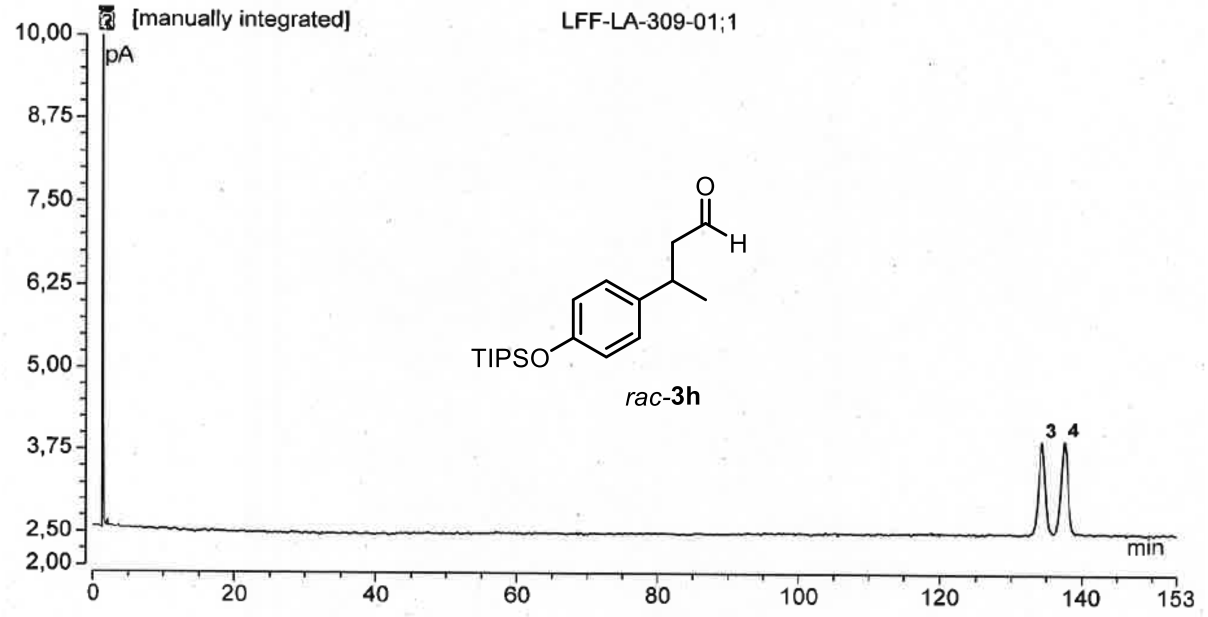

No.	Ret.Time min	Rel.Area %	Peak Name
3	134,37	50,20 .	
4	137,53	49,80 .	

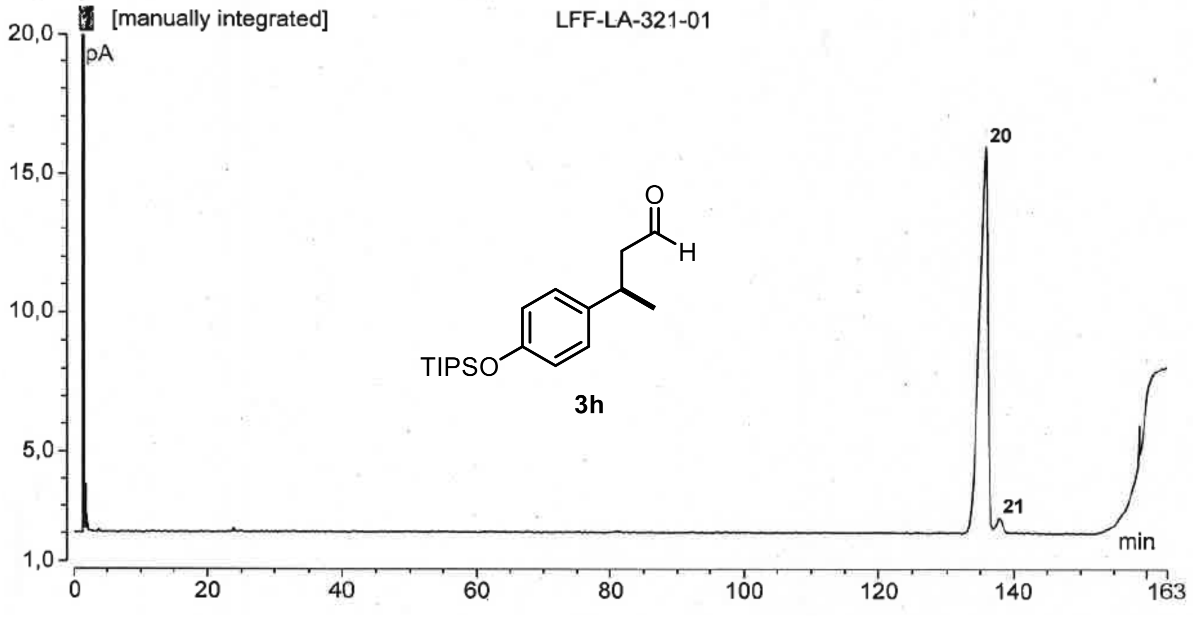

No.	Ret.Time min	Rel.Area %	Peak Name
20	135,90	97,27 .	
21	137,78	2,73 .	

GC (30 m Ivadex-1, injection temperature: 220 °C, 110 °C iso 5 min, 2 °C/min, 150 °C iso 5 min, 8 °C/min, 220 °C iso 5 min, 0.5 bar H_2)

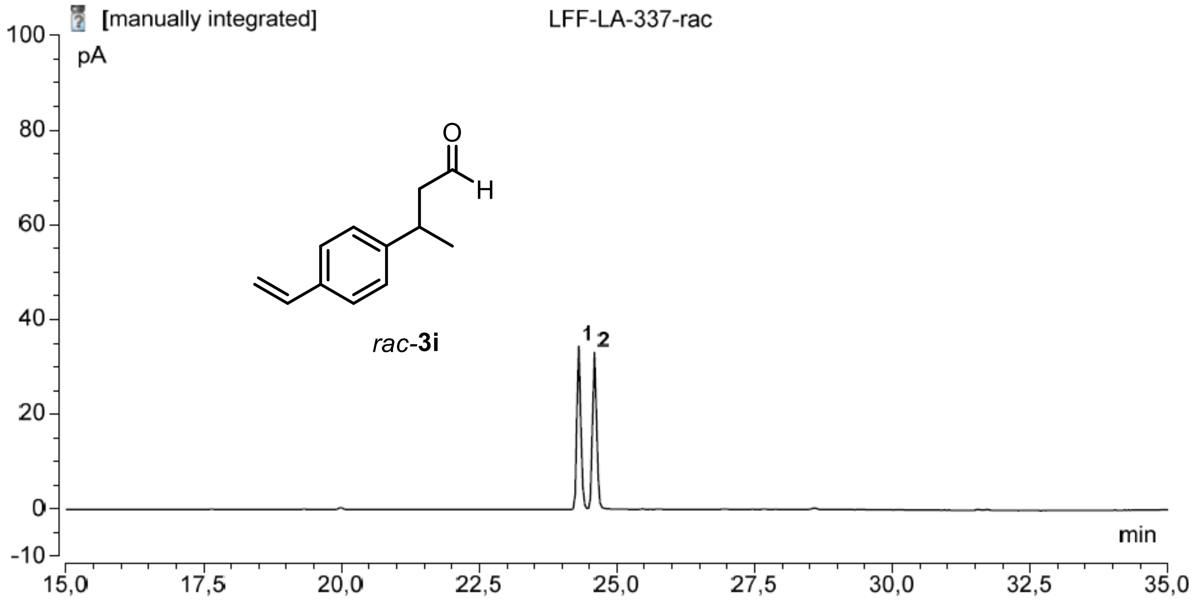

No.	Ret.Time min	Rel.Area %
1	24,31	49,98
2	24,59	50,02

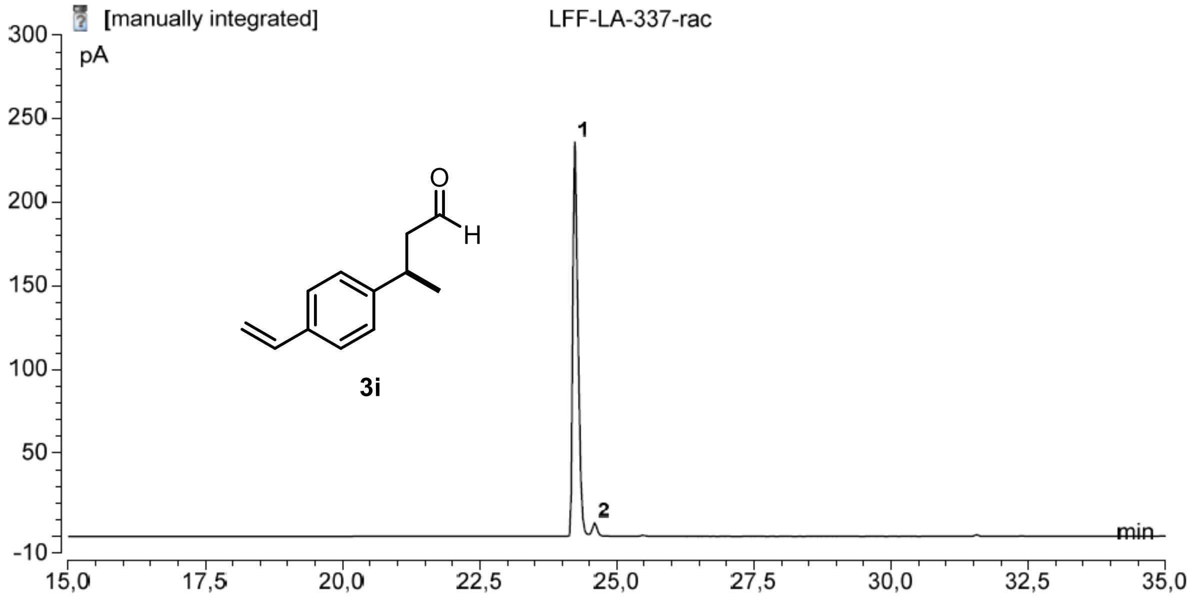

No.	Ret.Time min	Rel.Area %
1	24,23	96,98
2	24,60	3,02

GC (30 m BGB-176, injection temperature: 220 °C, 130 °C iso 410 min, 8 °C/min, 240 °C, 0.6 bar H_2)

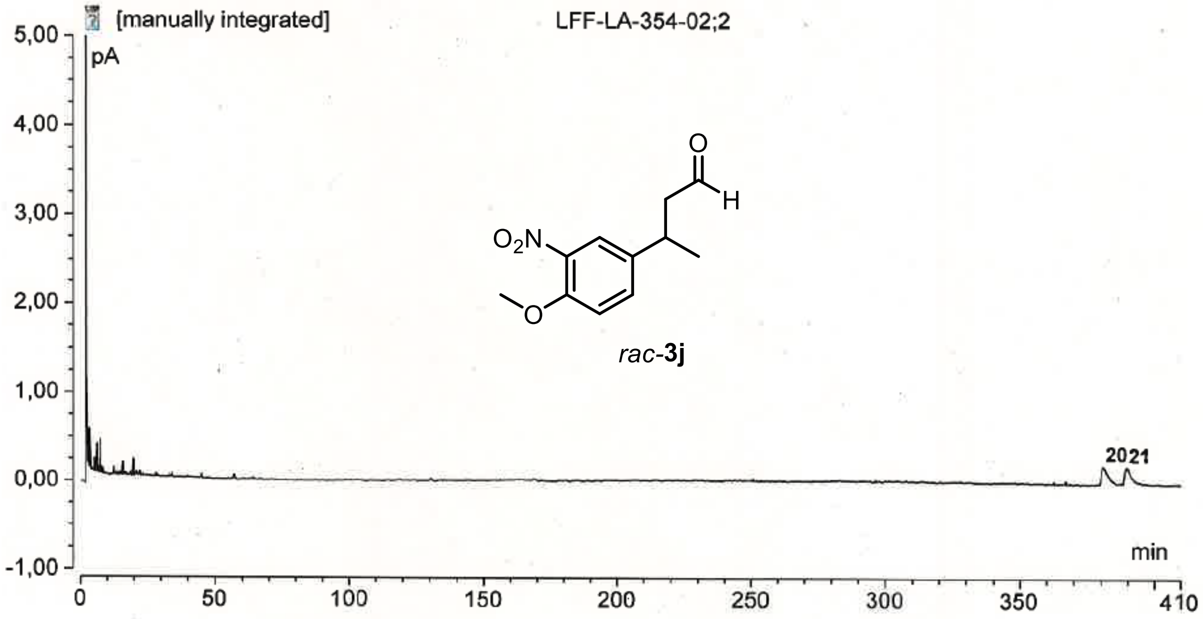

No.	Ret.Time min	Rel.Area %	Peak Name
20	380,81	49,43	.
21	389,51	50,57	.

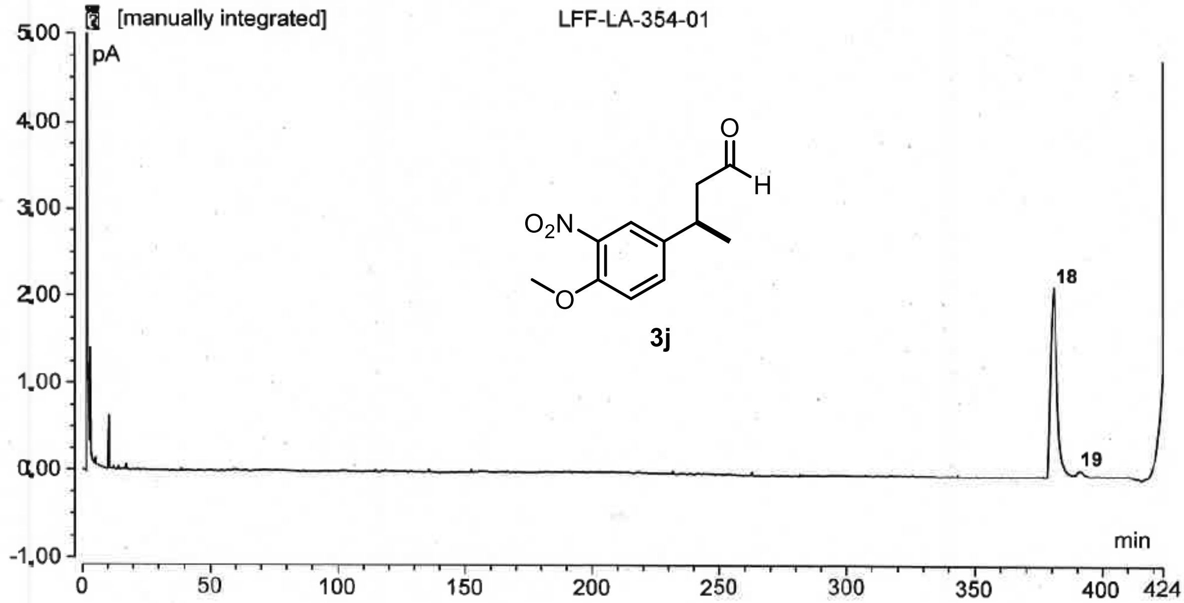

No.	Ret.Time min	Rel.Area %	Peak Name
18	380,81	96,67	.
19	390,47	3,33	.

GC (30 m Ivadex-1, injection temperature: 220 °C, 110 °C iso 5 min, 2 °C/min, 150 °C iso 5 min, 8 °C/min, 22 0 °C iso 5 min, 0.5 bar H_2)

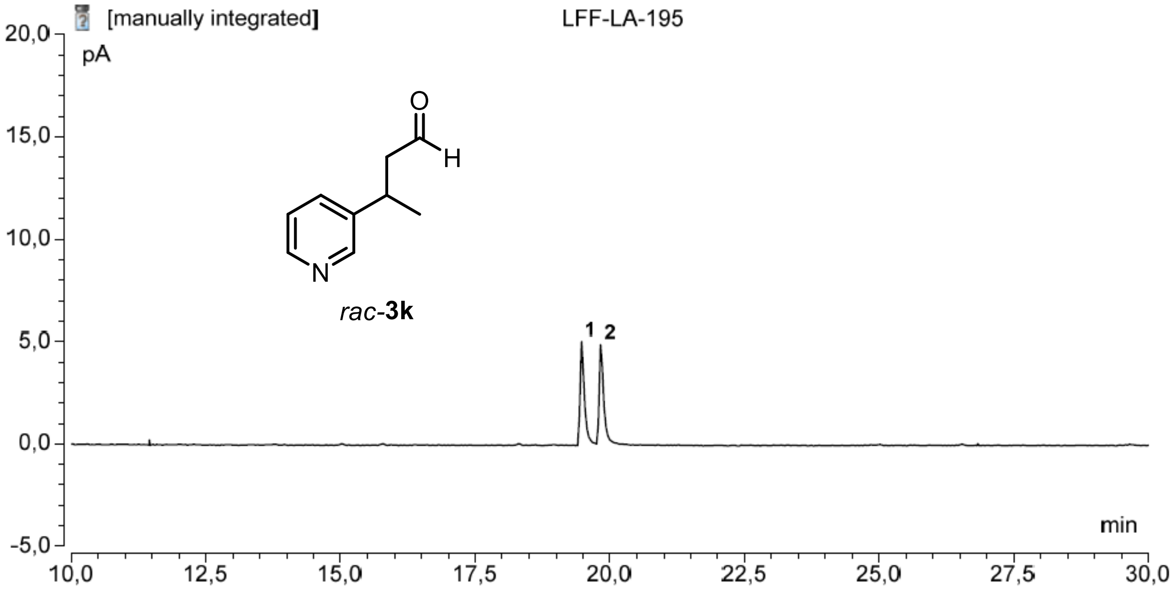

No.	Ret.Time min	Rel.Area %
1	19,47	49,89
2	19,83	50,11

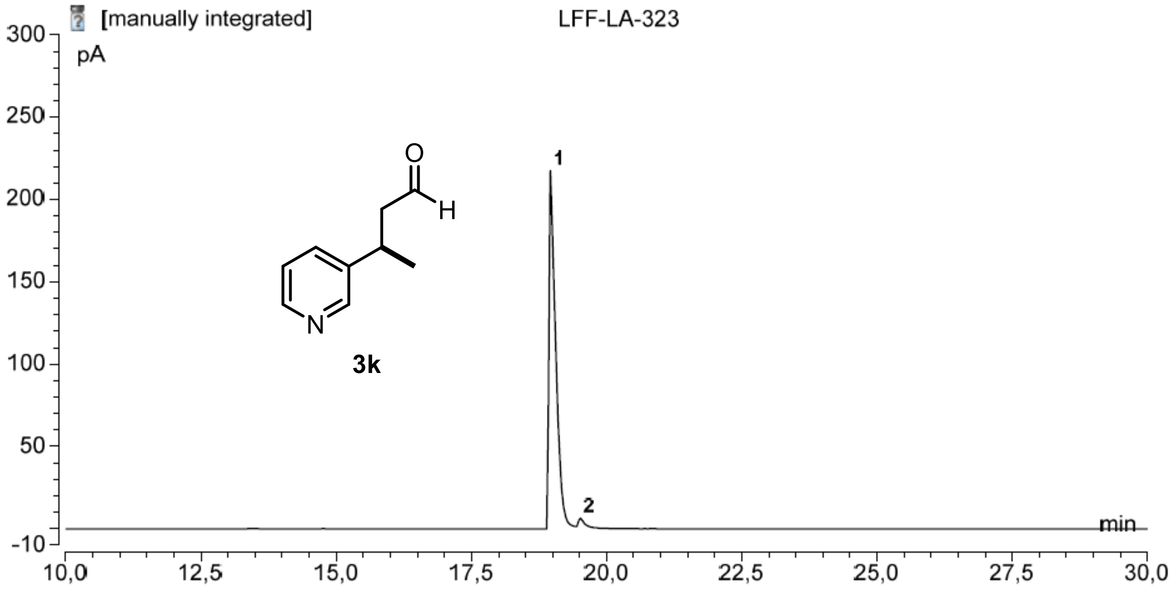

No.	Ret.Time min	Rel.Area %
1	18,96	97,00
2	19,51	3,00

GC (30 m Ivadex-1, injection temperature: 220 °C, 110 °C iso 5 min, 2 °C/min, 150 °C iso 5 min, 8 °C/min, 220 °C iso 5 min, 0.5 bar H_2)

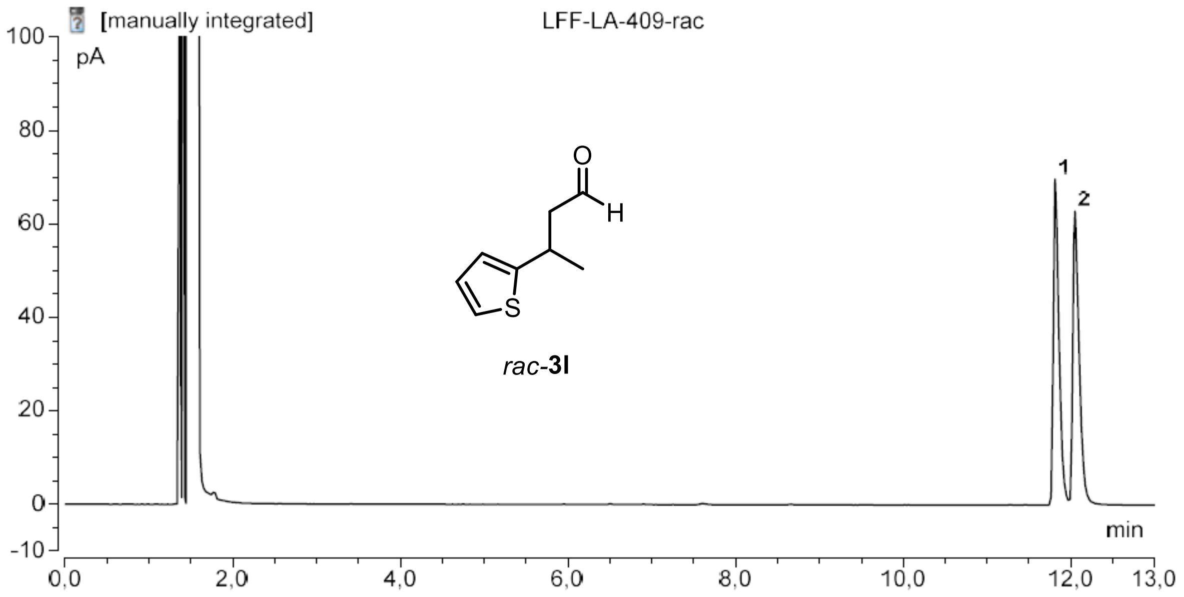

No.	Ret.Time min	Rel.Area %
1	11,81	50,01
2	12,05	49,99

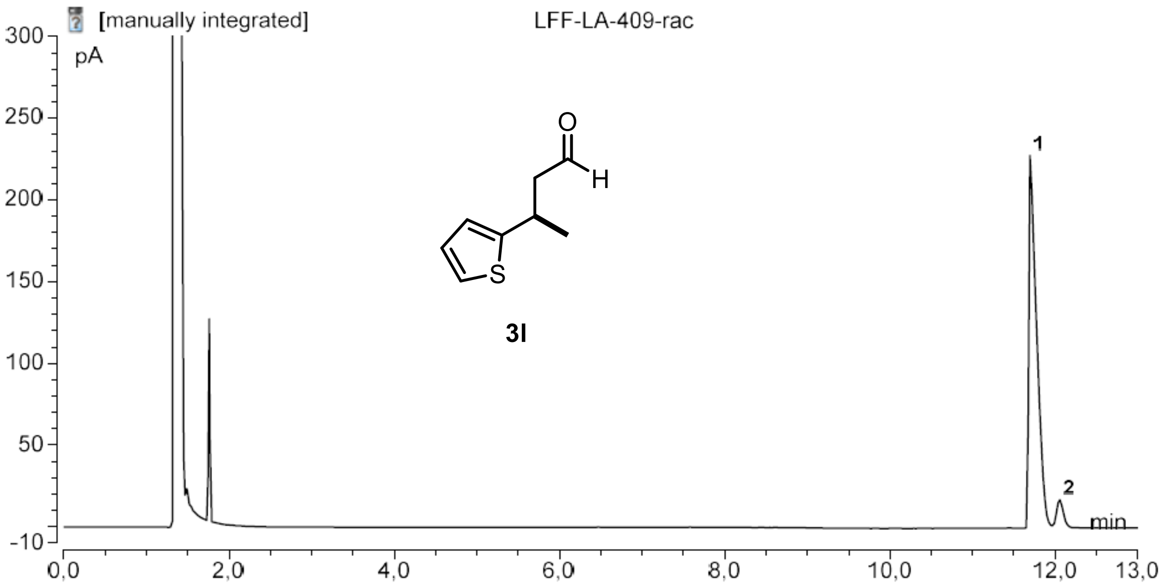

No.	Ret.Time min	Rel.Area %
1	11,70	93,91
2	12,06	6,09

GC (30 m Ivadex-1, injection temperature: 220 °C, 110 °C iso 5 min, 2 °C/min, 150 °C iso 5 min, 8 °C/min, 220 °C iso 5 min, 0.5 bar H_2)

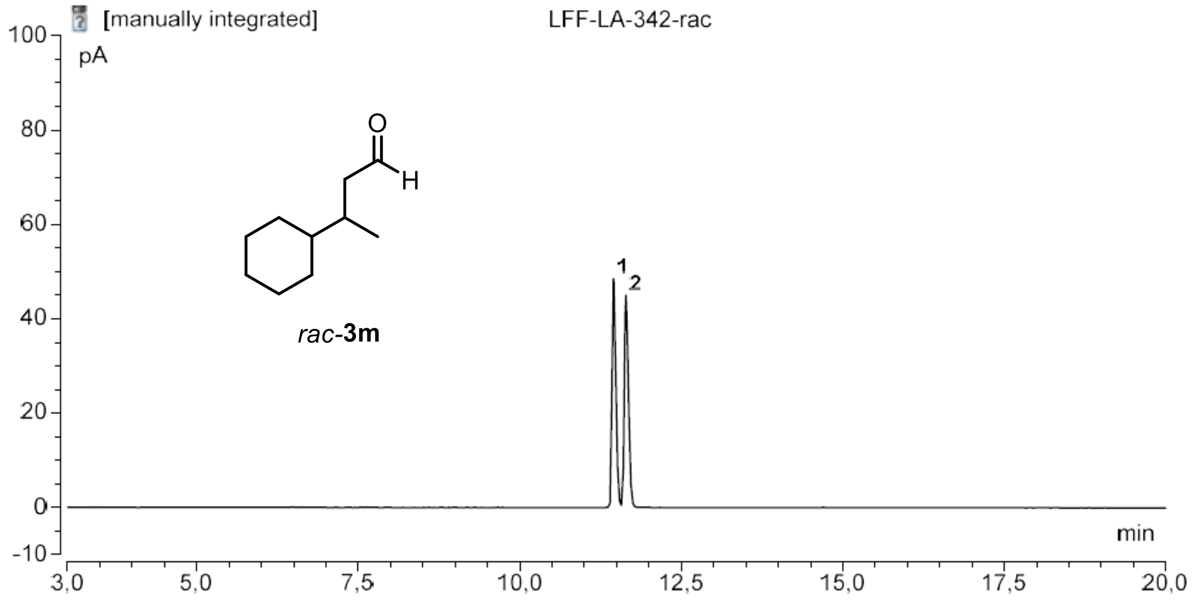

No.	Ret.Time min	Rel.Area %
1	11,45	49,94
2	11,64	50,06

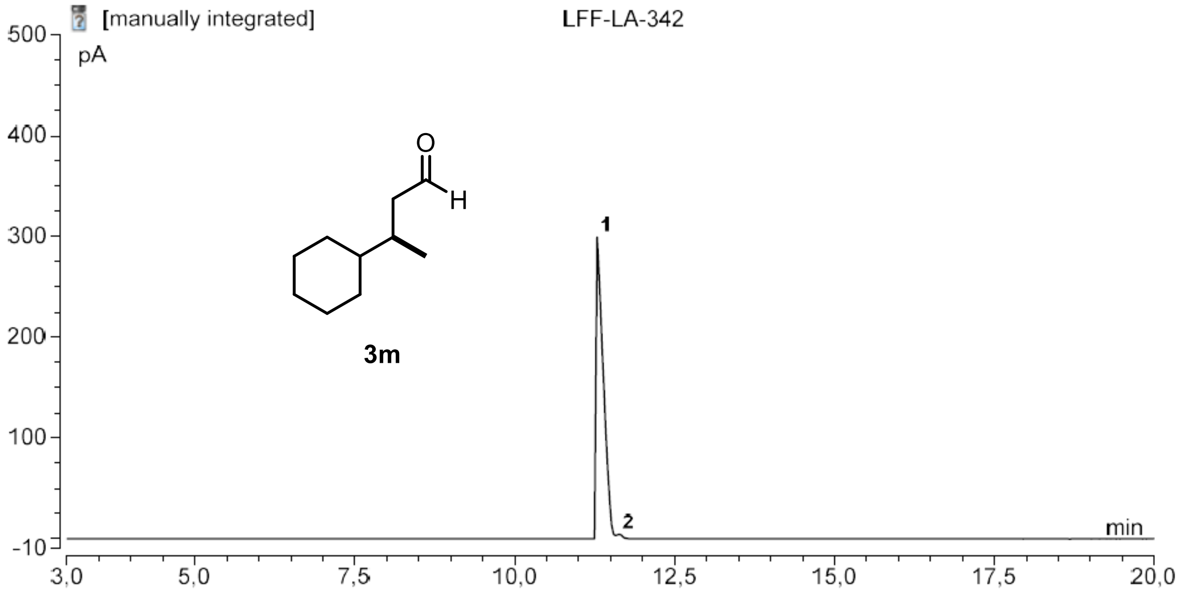

No.	Ret.Time min	Rel.Area %
1	11,29	98,74
2	11,63	1,26

GC (30 m Ivadex-1, injection temperature: 220 °C, 40 °C, 1 °C/min, 80 °C iso 5 min, 8 °C/min, 220 °C iso 5 min, 0.5 bar H_2)

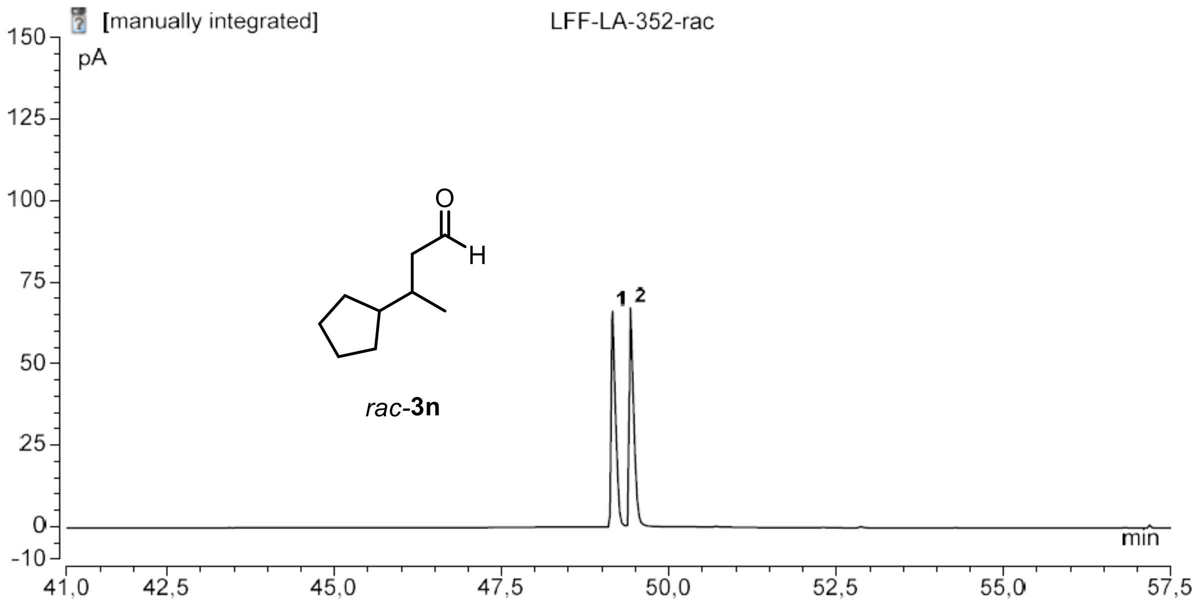

No.	Ret.Time min	Rel.Area %
1	49,16	49,93
2	49,43	50,07

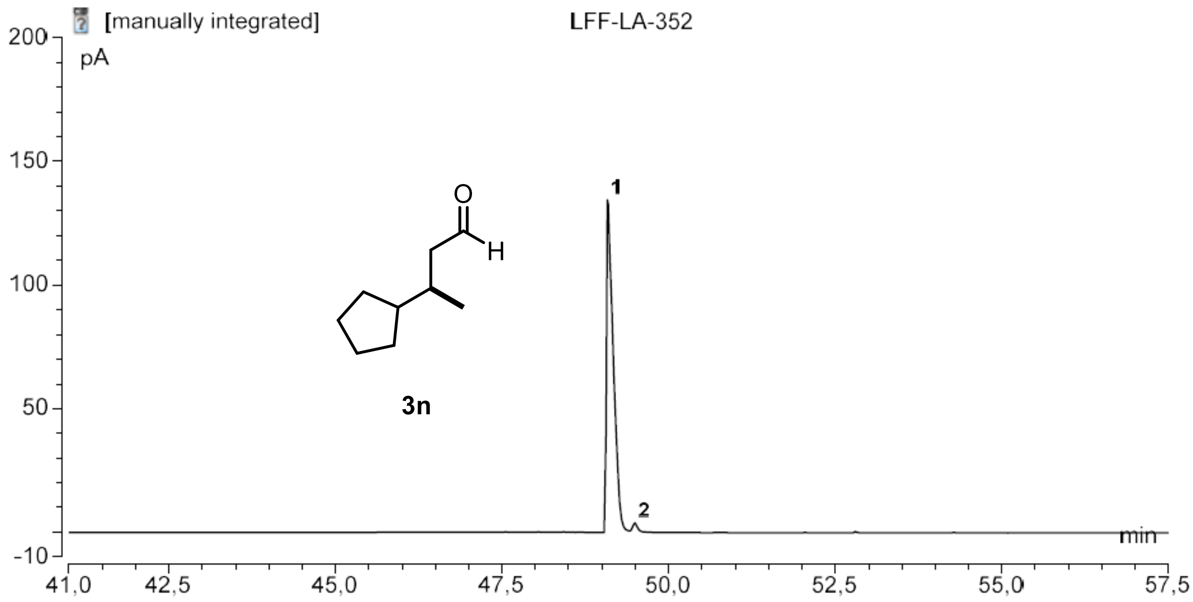

No.	Ret.Time min	Rel.Area %
1	49,08	98,37
2	49,49	1,63

GC (30 m Ivadex-1, injection temperature: 220 °C, 40 °C, 1 °C/min, 80 °C iso 5 min, 8 °C/min, 220 °C iso 5 min, 0.5 bar H_2)

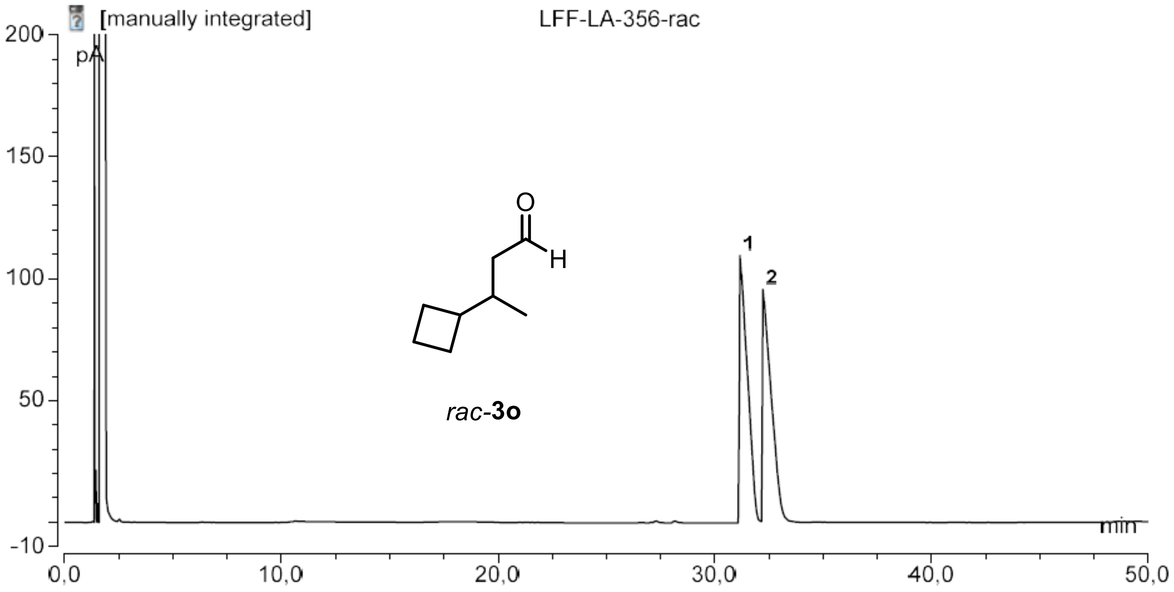

No.	Ret.Time min	Rel.Area %
1	31,17	50,26
2	32,22	49,74

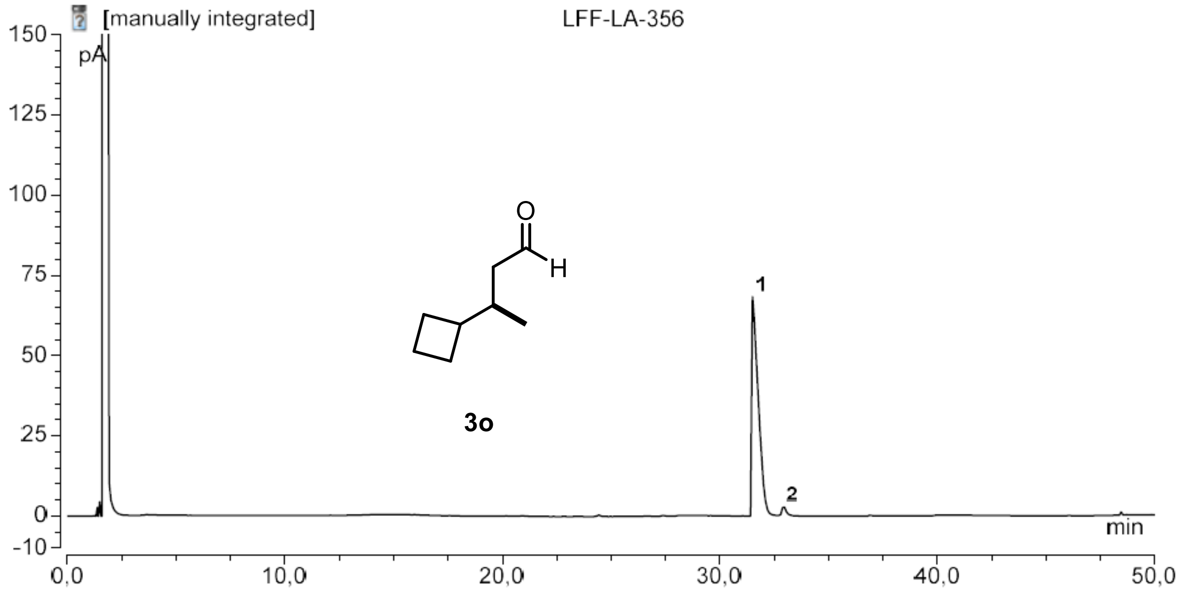

No.	Ret.Time min	Rel.Area %
1	31,51	97,16
2	32,92	2,84

GC (30 m Ivadex-1, injection temperature: 220 °C, 40 °C, 1 °C/min, 80 °C iso 5 min, 8 °C/min, 220 °C iso 5 min, 0.5 bar H_2)

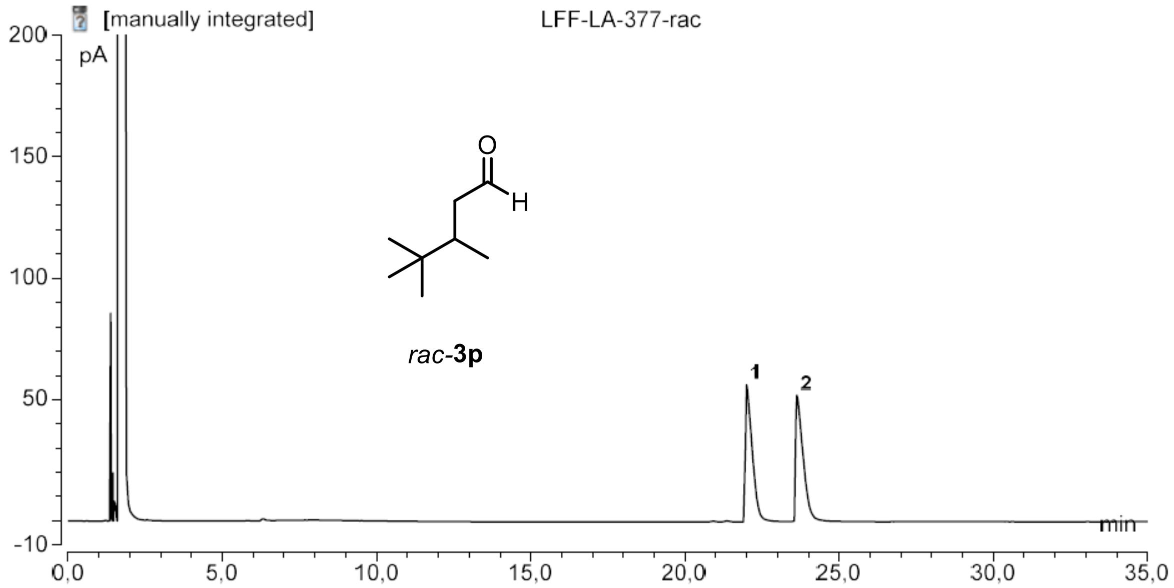

No.	Ret.Time min	Rel.Area %
1	22,00	49,99
2	23,63	50,01

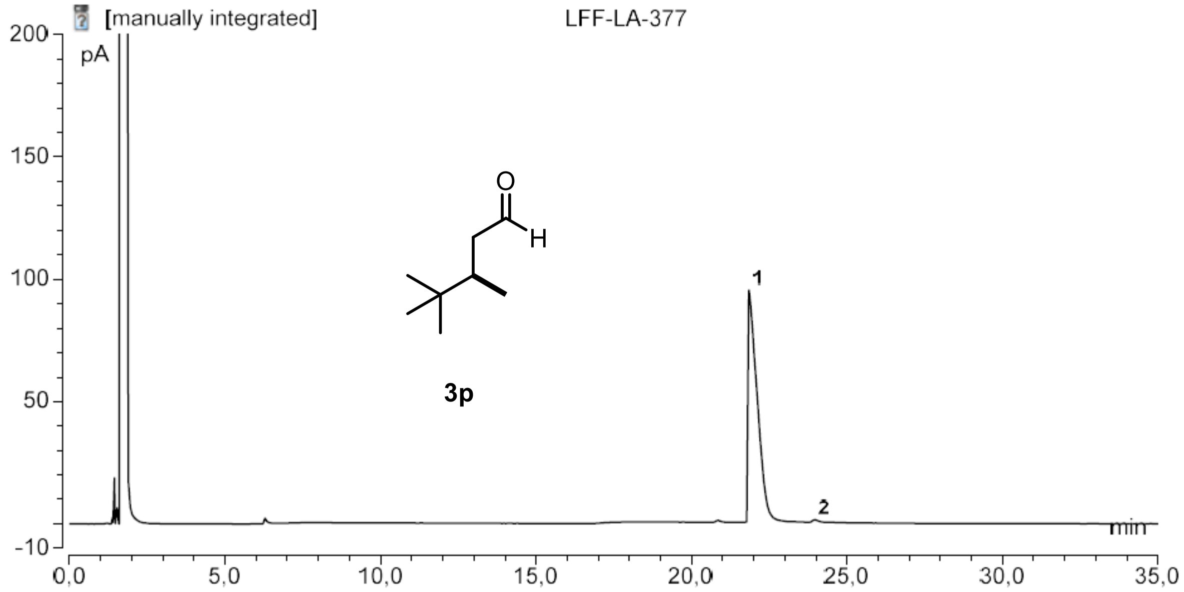

No.	Ret.Time min	Rel.Area %
1	21,85	99,33
2	23,97	0,67

GC (30 m Ivadex-1, injection temperature: 220 °C, 40 °C, 1 °C/min, 80 °C iso 5 min, 8 °C/min, 220 °C iso 5 min, 0.5 bar H_2)

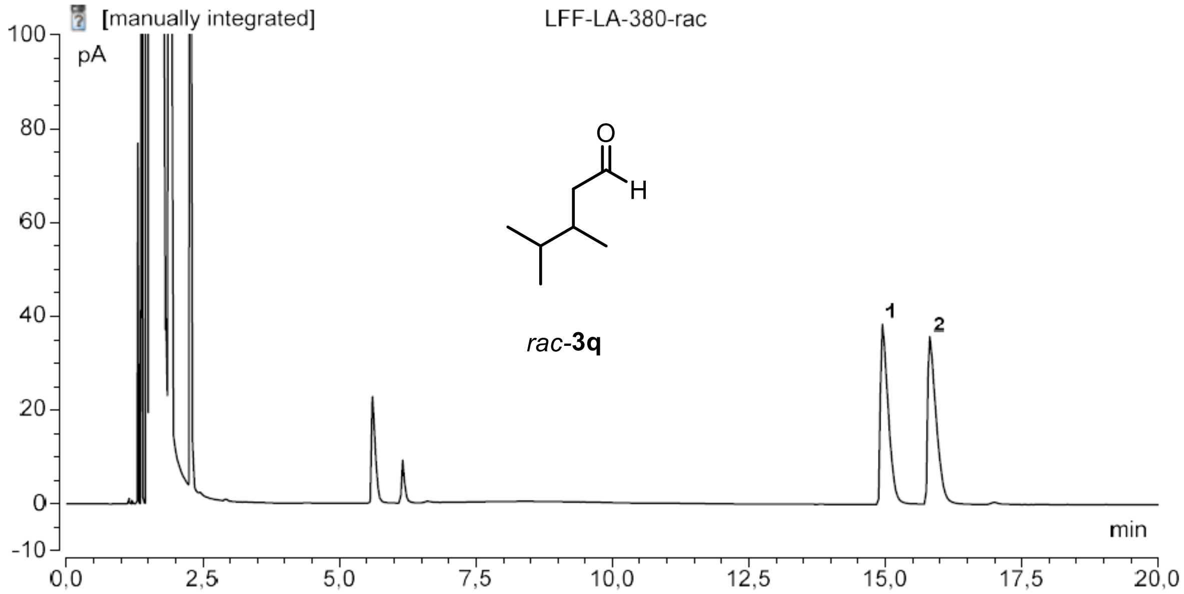

No.	Ret.Time min	Rel.Area %
1	14,95	49,92
2	15,81	50,08

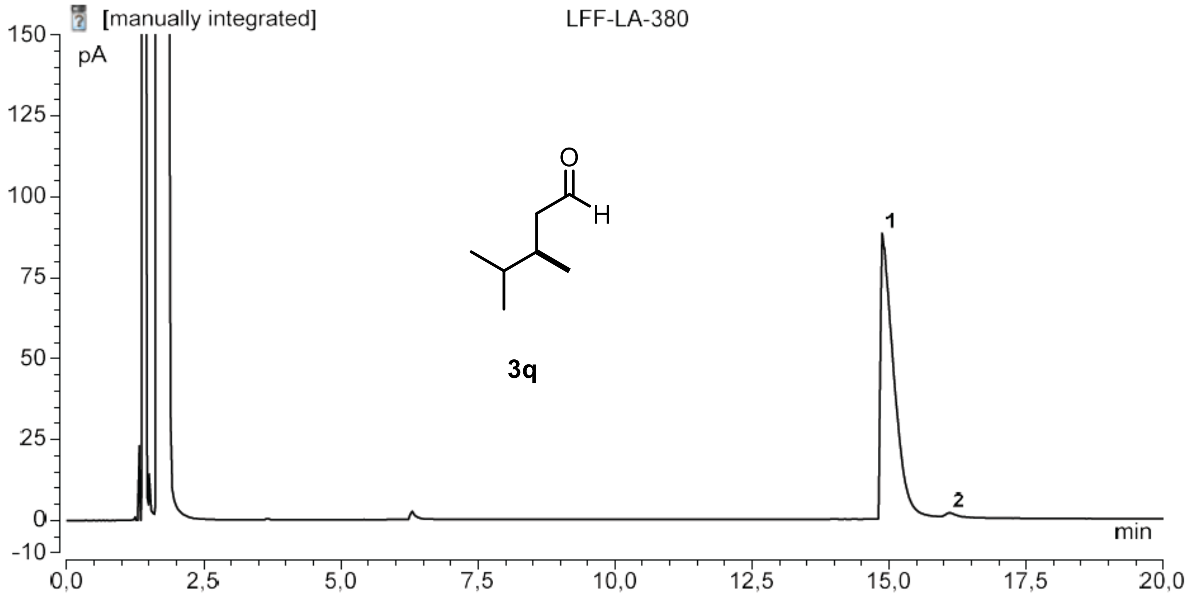

No.	Ret.Time min	Rel.Area %
1	14,87	96,95
2	16,10	3,05

HPLC (OD-3, *n*-heptane/ *i*PrOH 95:5, 298 K, 340 nm)

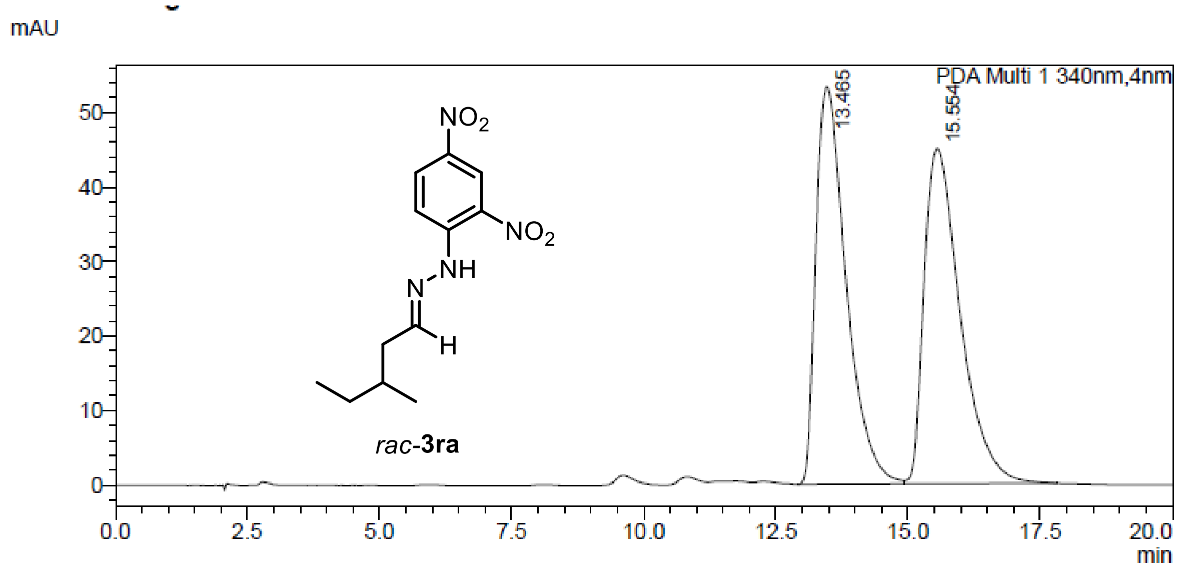

peak #	t_R / min	area / %
1	13.465	49.827
2	15.554	50.173

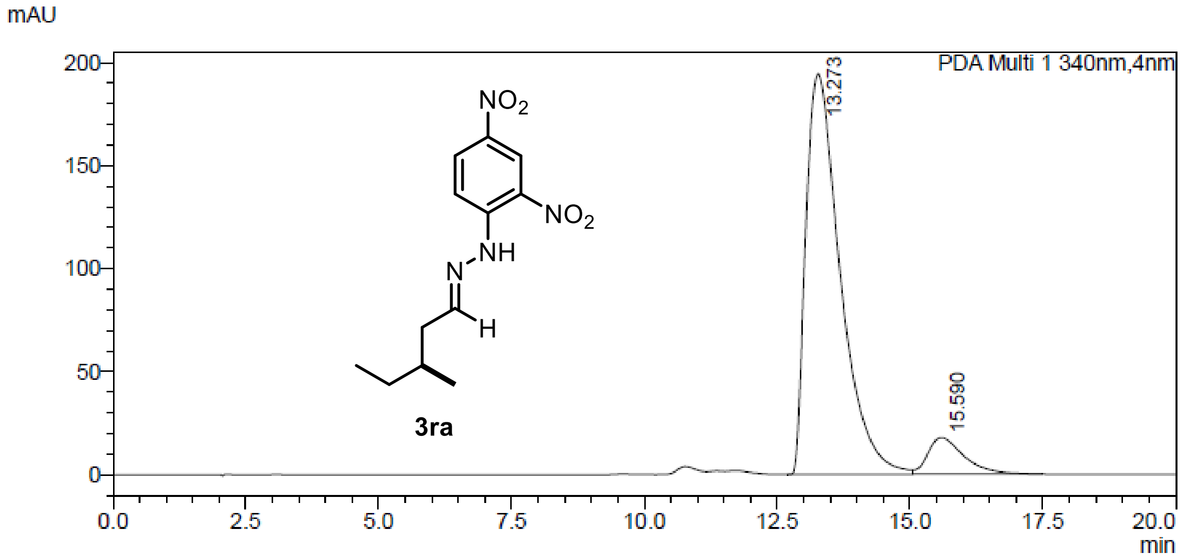

peak #	t_R / min	area / %
1	13.273	91.243
2	15.590	8.757

HPLC (OD-3, *n*-heptane/ *i*PrOH 95:5, 298 K, 340 nm)

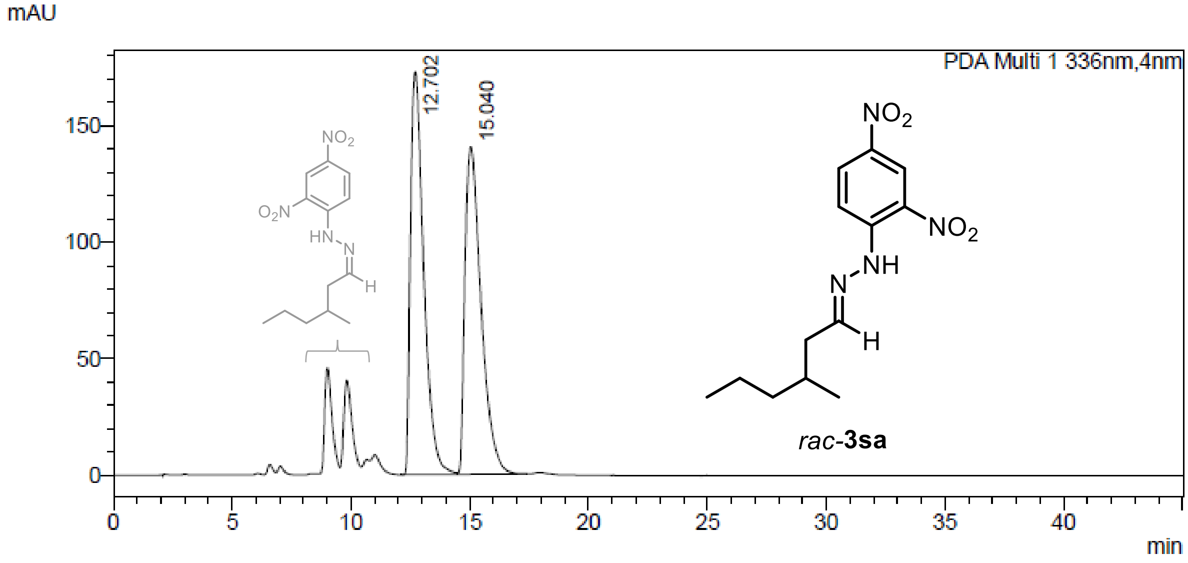

peak #	t_R / min	area / %
1	12.702	50.183
2	15.040	49.817

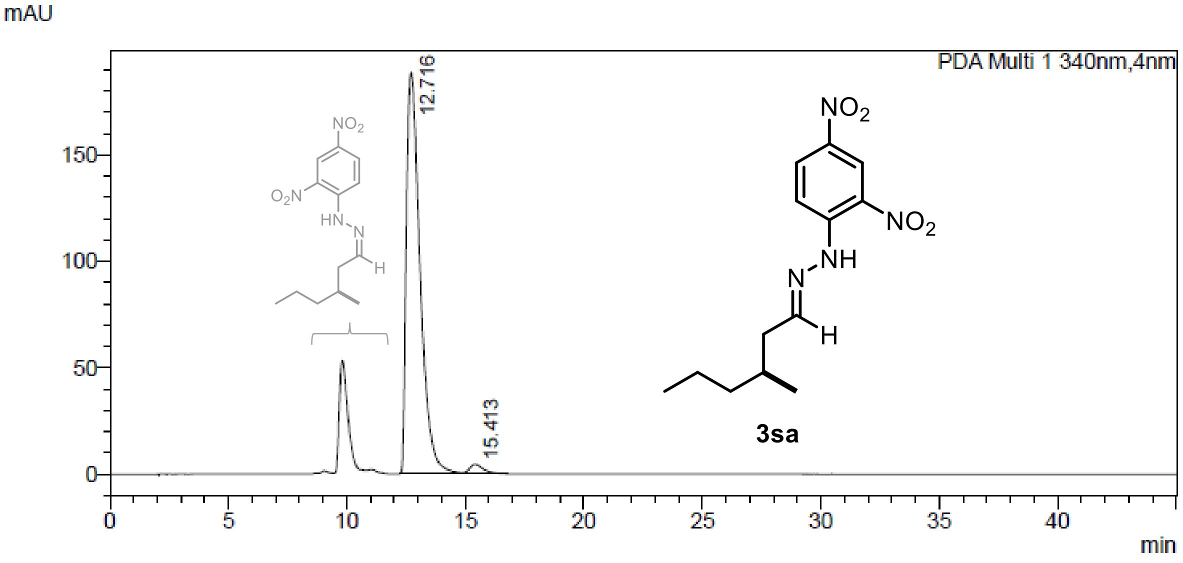

peak #	t_R / min	area / %
1	12.716	97.667
2	15.413	2.333

GC (30 m Ivadex-1, injection temperature: 220 °C, 45 °C iso 40 min, 1 °C/min, 65 °C, 8 °C/min, 220 °C iso 5 min, 0.5 bar H_2)

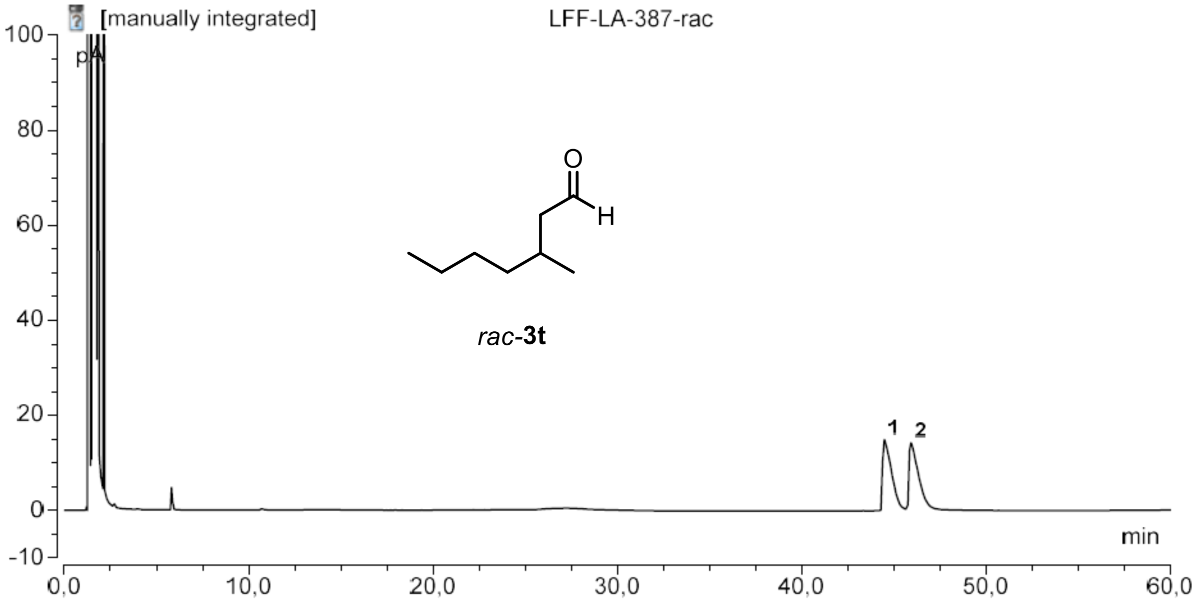

No.	Ret.Time min	Rel.Area %
1	44,47	49,76
2	45,91	50,24

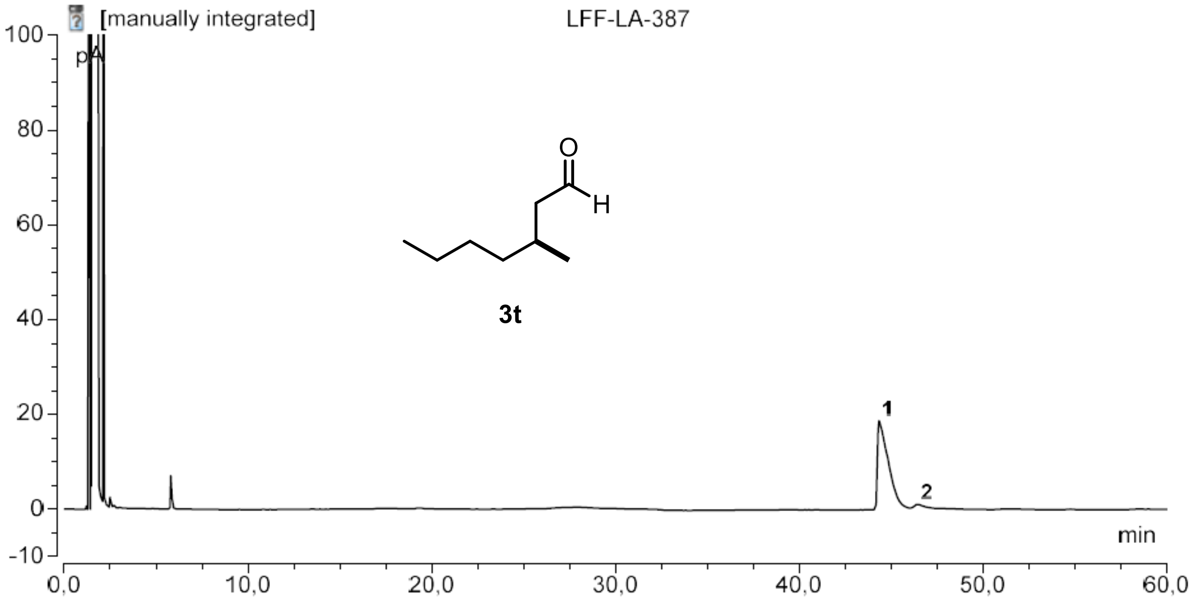

No.	Ret.Time min	Rel.Area %
1	44,33	95,05
2	46,43	4,95

GC (25 m Hydrodex-beta-TBDAc-CD, injection temperature: 220 °C, 70 °C iso 44 min, 8 °C/min, 220 °C, 0.5 bar H_2)

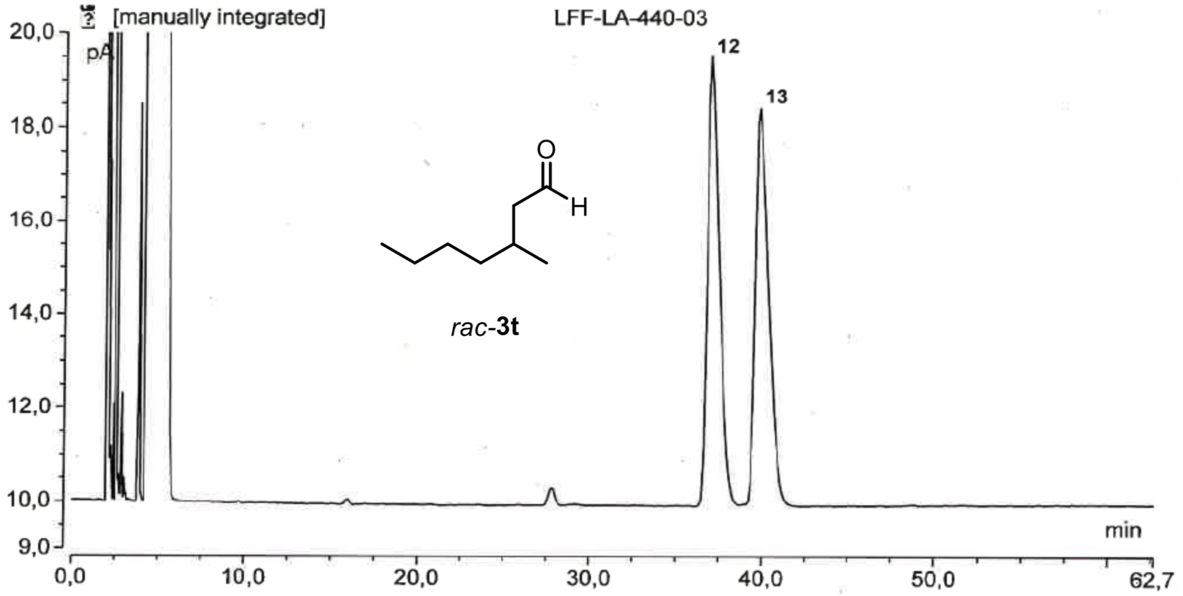

No.	Ret.Time min	Rel.Area %	Peak Name
12	36,95	49,91	.
13	39,76	50,09	.

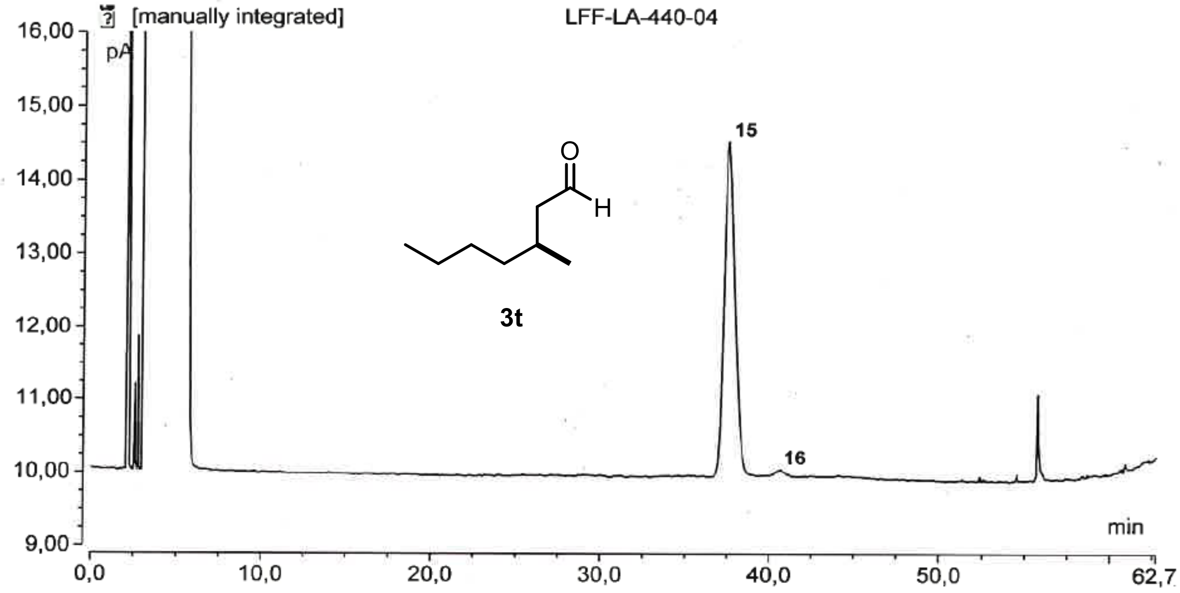

No.	Ret.Time min	Rel.Area %	Peak Name
15	37,51	97,59	.
16	40,56	2,41	.

GC (30 m BGB-174, injection temperature: 220 °C, 70 °C iso 120 min, 8 °C/min, 240 °C iso 3 min, 0.6 bar H_2)

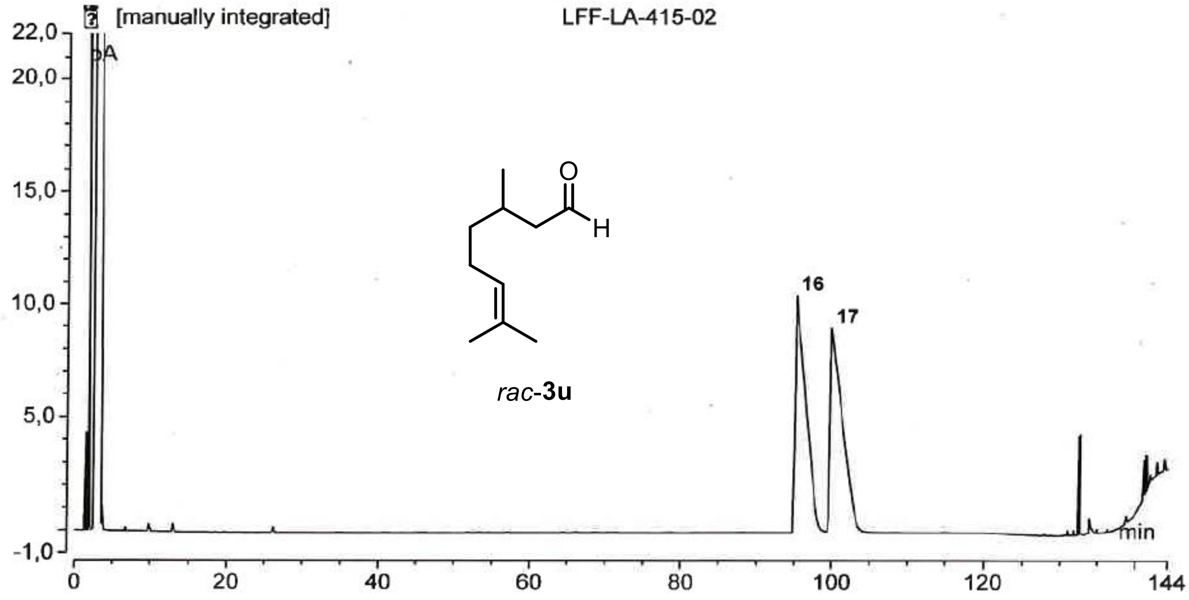

No.	Ret.Time min	Rel.Area %	Peak Name
16	95,16	49,99 .	
17	99,80	50,01 .	

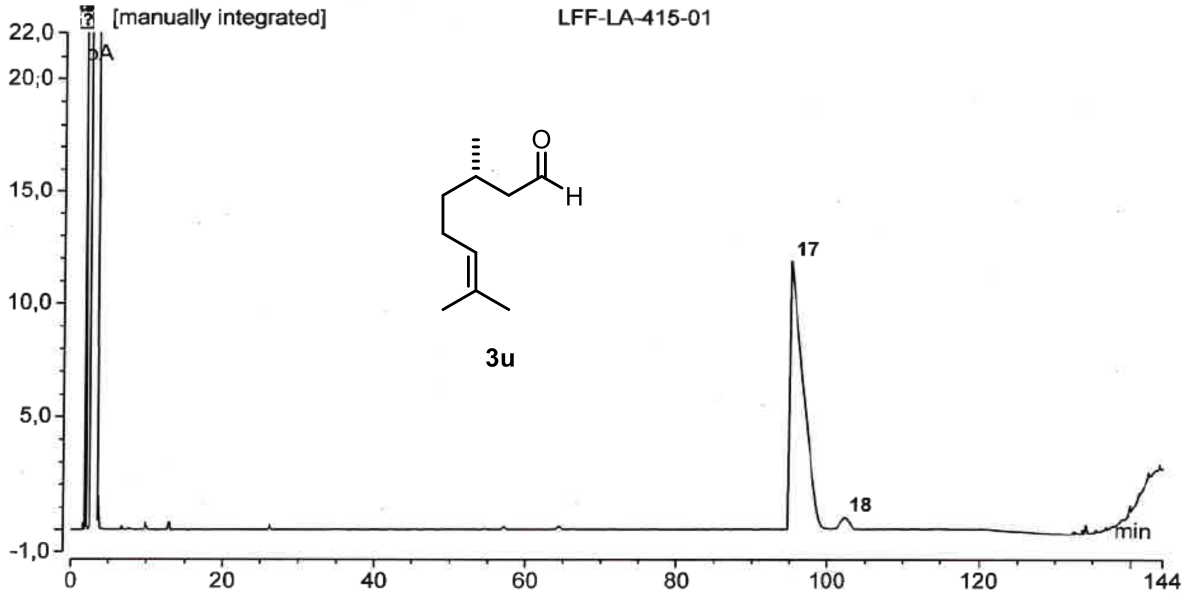

No.	Ret.Time min	Rel.Area %	Peak Name
17	95,03	97,24 .	
18	102,32	2,76 .	

GC (30 m BGB-174, injection temperature: 220 °C, 110 °C iso 160 min, 8 °C/min, 220 °C iso 3 min, 0.6 bar H_2)

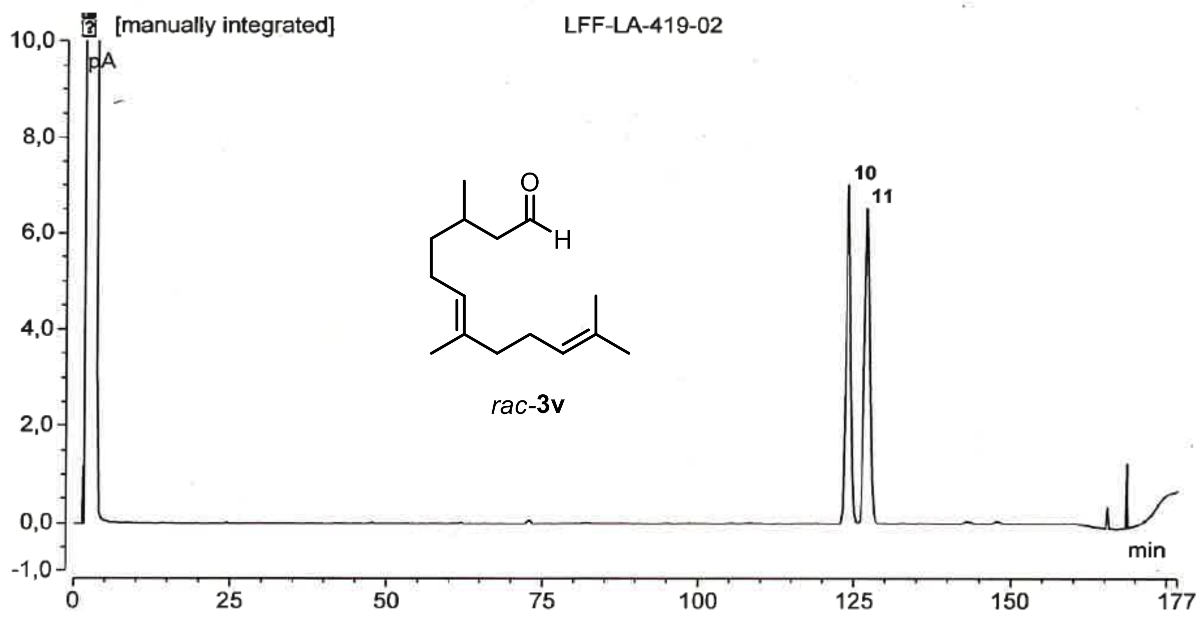

No.	Ret.Time min	Rel.Area %	Peak Name
10	123,86	50,03	.
11	126,85	49,97	.

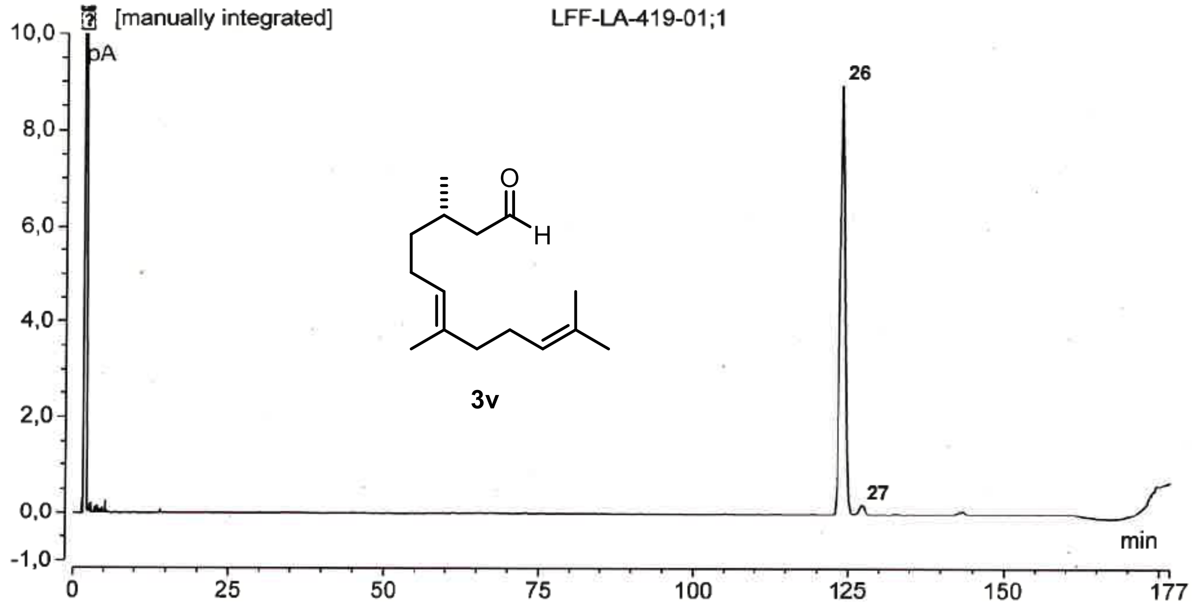

No.	Ret.Time min	Rel.Area %	Peak Name
26	123,78	97,47	.
27	127,22	2,53	.

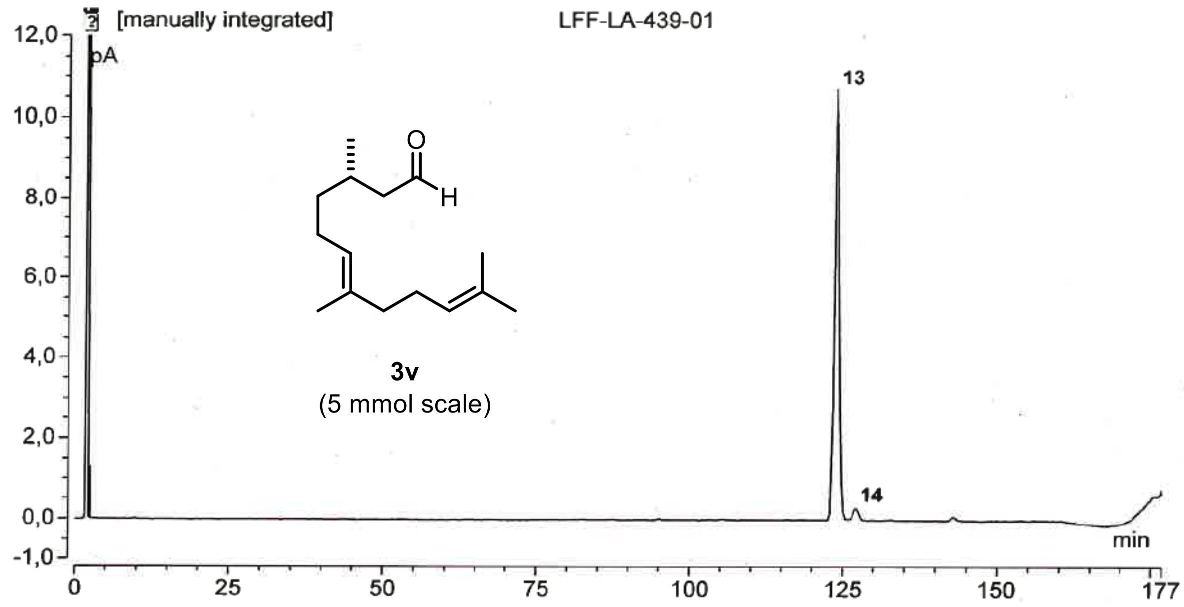

No.	Ret.Time min	Rel.Area %	Peak Name
13	123,59	96,97	.
14	127,08	3,03	.

12. References

(1) Metternich, J. B.; Gilmour, R. A Bio-Inspired, Catalytic *E* → *Z* Isomerization of Activated Olefins. *J. Am. Chem. Soc.* **2015**, *137*, 11254–11257.

(2) Obrien, J. M.; Lee, K. S.; Hoveyda, A. H. Enantioselective Synthesis of Boron-Substituted Quaternary Carbons by NHC-Cu-Catalyzed Boronate Conjugate Additions to Unsaturated Carboxylic Esters, Ketones, or Thioesters. *J. Am. Chem. Soc.* **2010**, *132*, 10630–10633.

(3) Bush, A. G.; Jiang, J. L.; Payne, P. R.; Ogilvie, W. W. Development of a Palladium Catalyzed Addition of Boronic Acids to Alkynyl Esters: Synthesis of Trisubstituted Olefins as Single Isomers. *Tetrahedron* **2009**, *65*, 8502–8506.

(4) Shevlin, M.; Friedfeld, M. R.; Sheng, H.; Pierson, N. A.; Hoyt, J. M.; Campeau, L. C.; Chirik, P. J. Nickel-Catalyzed Asymmetric Alkene Hydrogenation of α,β-Unsaturated Esters: High-Throughput Experimentation-Enabled Reaction Discovery, Optimization, and Mechanistic Elucidation. *J. Am. Chem. Soc.* **2016**, *138*, 3562–3569.

(5) Zheng, J.; Margarita, C.; Krajangsri, S.; Andersson, P. G. Asymmetric Total Synthesis of (-)-Juvabione via Sequential Ir-Catalyzed Hydrogenations. *Org. Lett.* **2018**, *20*, 5676–5679.

(6) Denmark, S. E.; Marble, L. K. Asymmetric Construction of Quaternary Stereogenic Centers via Auxiliary-Based SN2′ Reactions: A Case of 1,7-Relative Stereogenesis. *Heterocycles* **2014**, *88*, 559–590.

(7) Rivera, A. C. P.; Still, R.; Frantz, D. E. Iron-Catalyzed Stereoselective Cross-Coupling Reactions of Stereodefined Enol Carbamates with Grignard Reagents. *Angew. Chem., Int. Ed.* **2016**, *55*, 6689–6693.

(8) Engel, D. A.; Dudley, G. B. Olefination of Ketones Using a Gold(III)-Catalyzed Meyer-Schuster Rearrangement. *Org. Lett.* **2006**, *8*, 4027–4029.

(9) Narczyk, A.; Stecko, S. An Entry to Non-Racemic β-Tertiary-β-Amino Alcohols, Building Blocks for the Synthesis of Aziridine, Piperazine, and Morpholine Scaffolds. *Org. Biomol. Chem.* **2020**, *18*, 5972–5981.

(10) Xiao, Y.; Huang, Y.; Zeng, Z.; Luo, X.; Qian, X.; Yang, Y. Harnessing Thorpe–Ingold Dialkylation to Access High-Hill-Percentage PH Probes. *J. Org. Chem.* **2022**, *87*, 85–93.

(11) Sahner, J. H.; Sucipto, H.; Wenzel, S. C.; Groh, M.; Hartmann, R. W.; Müller, R. Advanced Mutasynthesis Studies on the Natural α-Pyrone Antibiotic Myxopyronin from Myxococcus Fulvus. *ChemBioChem* **2015**, *16*, 946–953.

(12) Castelani, P.; Comasseto, J. V. Diastereoselective Synthesis of α,β-Unsaturated Systems. *Tetrahedron* **2005**, *61*, 2319–2326.

(13) Brégent, T.; Bouillon, J. P.; Poisson, T. Copper-Photocatalyzed Contra-Thermodynamic Isomerization of Polarized Alkenes. *Org. Lett.* **2020**, *22*, 7688–7693.

(14) Asikainen, M.; Lewis, W.; Blake, A. J.; Woodward, S. An SN2′ Displacement Approach to Allenyl Acetates. *Tetrahedron Lett.* **2010**, *51*, 6454–6456.

(15) Næsborg, L.; Corti, V.; Leth, L. A.; Poulsen, P. H.; Jørgensen, K. A. Catalytic Asymmetric Oxidative γ-Coupling of α,β-Unsaturated Aldehydes with Air as the Terminal Oxidant. *Angew. Chemie Int. Ed.* **2018**, *57*, 1606–1610.

(16) Tummatorn, J.; Dudley, G. B. Generation of Medium-Ring Cycloalkynes by Ring Expansion of Vinylogous Acyl Triflates. *Org. Lett.* **2011**, *13*, 1572–1575.

(17) Neel, A. J.; Milo, A.; Sigman, M. S.; Toste, F. D. Enantiodivergent Fluorination of Allylic Alcohols: Data Set Design Reveals Structural Interplay between Achiral Directing Group and Chiral Anion. *J. Am. Chem. Soc.* **2016**, *138*, 3863–3875.

(18) Bench, B. J.; Liu, C.; Evett, C. R.; Watanabe, C. M. H. Proline Promoted Synthesis of Ring-Fused Homodimers: Self-Condensation of α,β-Unsaturated Aldehydes. *J. Org. Chem.* **2006**, *71*, 9458–9463.

(19) Shintani, R.; Kimura, T.; Hayashi, T. Rhodium/Diene-Catalyzed Asymmetric 1,4-Addition of Arylboronic Acids to α,β-Unsaturated Weinreb Amides. *Chem. Commun.* **2005**, No. 25, 3213–3214.

(20) Jung, W. Y.; Hechavarria Fonseca, M. T.; Vignola, N.; List, B. Metal-Free, Organocatalytic Asymmetric Transfer Hydrogenation of α,β-Unsaturated Aldehydes. *Angew. Chem., Int. Ed.* **2004**, *44*, 108–110.

(21) Ouellet, S. G.; Tuttle, J. B.; MacMillan, D. W. C. Enantioselective Organocatalytic Hydride Reduction. *J. Am. Chem. Soc.* **2005**, *127*, 32–33.

(22) Bannwarth, C.; Ehlert, S.; Grimme, S. GFN2-XTB - An Accurate and Broadly Parametrized Self-Consistent Tight-Binding Quantum Chemical Method with Multipole Electrostatics and Density-Dependent Dispersion Contributions. *J. Chem. Theory Comput.* **2019**, *15*, 1652–1671.

(23) Pracht, P.; Bohle, F.; Grimme, S. Automated Exploration of the Low-Energy Chemical Space with Fast Quantum Chemical Methods. *Phys. Chem. Chem. Phys.* **2020**, *22*, 7169–7192.

(24) Neese, F. The ORCA Program System. *Wiley Interdiscip. Rev. Comput. Mol. Sci.* **2012**, *2*, 73–78.

(25) Perdew, J. P.; Burke, K.; Ernzerhof, M. Generalized Gradient Approximation Made Simple. *Phys. Rev. Lett.* **1996**, *77*, 3865–3868.

(26) Grimme, S.; Antony, J.; Ehrlich, S.; Krieg, H. A Consistent and Accurate Ab Initio Parametrization of Density Functional Dispersion Correction (DFT-D) for the 94 Elements H-Pu. *J. Chem. Phys.* **2010**, *132*.

(27) Grimme, S.; Ehrlich, S.; Goerigk, L. Effect of the Damping Function in Dispersion Corrected Density Functional Theory. *J. Comput. Chem.* **2011**, *32*, 1456–1465.

(28) Weigend, F.; Ahlrichs, R. Balanced Basis Sets of Split Valence, Triple Zeta Valence and Quadruple Zeta Valence Quality for H to Rn: Design and Assessment of Accuracy. *Phys. Chem. Chem. Phys.* **2005**, *7*, 3297–3305.

(29) Eichkorn, K.; Treutler, O.; Öhm, H.; Häser, M.; Ahlrichs, R. Auxiliary Basis Sets to Approximate Coulomb Potentials (Chem. Phys. Letters 240 (1995) 283) (PII:0009-2614(95)00621-4). *Chemical Physics Letters*. ELSEVIER 1995, pp 652–660.

(30) Neese, F. An Improvement of the Resolution of the Identity Approximation for the Formation of the Coulomb Matrix. *J. Comput. Chem.* **2003**, *24*, 1740–1747.

(31) Weigend, F. Accurate Coulomb-Fitting Basis Sets for H to Rn. *Phys. Chem. Chem. Phys.* **2006**, *8*, 1057–1065.

(32) Barone, V.; Cossi, M. Quantum Calculation of Molecular Energies and Energy Gradients in Solution by a Conductor Solvent Model. *J. Phys. Chem. A* **1998**, *102*, 1995–2001.

(33) GitHub - duartegroup/otherm: thermochemical contributions from ORCA calculations https://github.com/duartegroup/otherm (accessed 2024 -03 -05).

(34) Lu, T.; Chen, Q. Independent Gradient Model Based on Hirshfeld Partition: A New Method for Visual Study of Interactions in Chemical Systems. *J. Comput. Chem.* **2022**, *43*, 539–555.

(35) Lu, T.; Chen, F. Multiwfn: A Multifunctional Wavefunction Analyzer. *J. Comput. Chem.* **2012**, *33*, 580–592.

(36) Lu, T. A Comprehensive Electron Wavefunction Analysis Toolbox for Chemists, Multiwfn. *J. Chem. Phys.* **2024**, *161*, 82503.

(37) Becke, A. D. Density-Functional Thermochemistry. III. The Role of Exact Exchange. *J. Chem. Phys.* **1993**, *98*, 5648–5652.

(38) Lee, C.; Yang, W.; Parr, R. G. Development of the Colle-Salvetti Correlation-Energy Formula into a Functional of the Electron Density. *Phys. Rev. B* **1988**, *37*, 785–789.

(39) Zhao, Y.; Truhlar, D. G. The M06 Suite of Density Functionals for Main Group Thermochemistry, Thermochemical Kinetics, Noncovalent Interactions, Excited States, and Transition Elements: Two New Functionals and Systematic Testing of Four M06-Class Functionals and 12 Other Function. *Theor. Chem. Acc.* **2008**, *120*, 215–241.

(40) Grimme, S.; Neese, F. Double-Hybrid Density Functional Theory for Excited Electronic States of Molecules. *J. Chem. Phys.* **2007**, *127*, 154116.

(41) Mardirossian, N.; Head-Gordon, M. Ωb97X-V: A 10-Parameter, Range-Separated Hybrid, Generalized Gradient Approximation Density Functional with Nonlocal Correlation, Designed by a Survival-of-the-Fittest Strategy. *Phys. Chem. Chem. Phys.* **2014**, *16*, 9904–9924.

(42) Mardirossian, N.; Head-Gordon, M. ω B97M-V: A Combinatorially Optimized, Range-Separated Hybrid, Meta-GGA Density Functional with VV10 Nonlocal Correlation. *J. Chem. Phys.* **2016**, *144*, 214110.

(43) Bistoni, G. Finding Chemical Concepts in the Hilbert Space: Coupled Cluster Analyses of Noncovalent Interactions. *Wiley Interdisciplinary Reviews: Computational Molecular Science*. 2020.

(44) Riplinger, C.; Sandhoefer, B.; Hansen, A.; Neese, F. Natural Triple Excitations in Local Coupled Cluster Calculations with Pair Natural Orbitals. *J. Chem. Phys.* **2013**, *139*, 134101.

(45) Riplinger, C.; Neese, F. An Efficient and near Linear Scaling Pair Natural Orbital Based Local Coupled Cluster Method. *J. Chem. Phys.* **2013**, *138*, 34106.

(46) Hansen, A.; Liakos, D. G.; Neese, F. Efficient and Accurate Local Single Reference Correlation Methods for High-Spin Open-Shell Molecules Using Pair Natural Orbitals. *J. Chem. Phys.* **2011**, *135*, 214102.

(47) Liakos, D. G.; Hansen, A.; Neese, F. Weak Molecular Interactions Studied with Parallel Implementations of the Local Pair Natural Orbital Coupled Pair and Coupled Cluster Methods. *J. Chem. Theory Comput.* **2011**, *7*, 76–87.

(48) Neese, F.; Hansen, A.; Wennmohs, F.; Grimme, S. Accurate Theoretical Chemistry with Coupled Pair Models. *Acc. Chem. Res.* **2009**, *42*, 641–648.

(49) Neese, F.; Hansen, A.; Liakos, D. G. Efficient and Accurate Approximations to the Local Coupled Cluster Singles Doubles Method Using a Truncated Pair Natural Orbital Basis. *J. Chem. Phys.* **2009**, *131*, 64103.

(50) Riplinger, C.; Pinski, P.; Becker, U.; Valeev, E. F.; Neese, F. Sparse Maps - A Systematic Infrastructure for Reduced-Scaling Electronic Structure Methods. II. Linear Scaling Domain Based Pair Natural Orbital Coupled Cluster Theory. *J. Chem. Phys.* **2016**, *144*, 24109.

(51) Bickelhaupt, F. M.; Houk, K. N. Analyzing Reaction Rates with the Distortion/Interaction-Activation Strain Model. *Angew. Chemie Int. Ed.* **2017**, *56*, 10070–10086.

(52) Smallcombe, S. H.; Patt, S. L.; Keifer, P. A. WET Solvent Suppression and Its Applications to

LC NMR and High-Resolution NMR Spectroscopy. *J. Magn. Reson. Ser. A* **1995**, *117*, 295–303.

(53) Burés, J. Variable Time Normalization Analysis: General Graphical Elucidation of Reaction Orders from Concentration Profiles. *Angew. Chemie Int. Ed.* **2016**, *55*, 16084–16087.
